# Photocatalytic Synthesis of Pentafluorosulfanyl Ketones, Acetals, and BCP Motifs Utilizing SF_6_

**DOI:** 10.1002/advs.75663

**Published:** 2026-05-15

**Authors:** Chen‐Hui Jiang, Haoran Xu, Yi‐Gang Yang, Xuan Nie, Meng‐Meng Zheng, Yue Zhao, Ya‐Wen Zuo, Wei‐Ran Ren, Shan Zhu, Ruo‐Xing Jin, Xiao‐Song Xue, Xi‐Sheng Wang

**Affiliations:** ^1^ Department of Pharmacy Division of Life Sciences and Medicine The First Affiliated Hospital of USTC University of Science and Technology of China Hefei China; ^2^ Department of Chemistry University of Science and Technology of China Hefei China; ^3^ State Key Laboratory of Fluorine and Nitrogen Chemistry and Advanced Materials Shanghai Institute of Organic Chemistry University of Chinese Academy of Sciences Chinese Academy of Sciences Shanghai China; ^4^ State Grid Anhui electric Power Research Institute Hefei Anhui China; ^5^ School of Chemistry and Materials Science Hangzhou Institute for Advanced Study University of Chinese Academy of Sciences Hangzhou China

**Keywords:** bicyclo[1.1.1]pentane motifs, DFT calculations, pentafluorosulfanylation, photoredox catalysis, sulfur hexafluoride

## Abstract

Sulfur hexafluoride (SF_6_), a chemically inert and stable gas essential to the electric power industry, poses severe environmental risks due to its persistence and high global warming potential. While traditional degradation methods are inefficient, photocatalytic single electron reduction (SER) enables SF_6_ activation into SF_5_ radicals, creating opportunities for both degradation and reutilization. The SF_5_ group, renowned for its strong electron‐withdrawing, lipophilic, and bioisosteric features, has great potential in drug discovery, while efficient methods for synthesizing alkyl‐ or BCP‐SF_5_ motifs remain scarce. As a safe, inexpensive, and atom‐economical alternative to conventional SF_5_ reagents, SF_6_ serves as an ideal yet underexplored SF_5_ source. Herein, we present a photocatalytic strategy for the direct synthesis of diverse SF_5_‐containing scaffolds, including ketones, acetals, and bicyclo[1.1.1]pentane derivatives. Derivatization studies demonstrate its synthetic versatility, particularly in accessing α‐SF_5_‐substituted acetaldehydes. Mechanistic and density functional theory (DFT) studies confirm the single electron reduction of SF_6_, highlighting a mild, efficient, and broadly applicable route for constructing SF_5_‐functionalized architectures in fluorinated drug development.

## Graphical abstract

A photocatalytic single electron reduction strategy is reported for the activation and valorization of sulfur hexafluoride (SF_6_), a potent greenhouse gas. Under mild conditions, SF_6_ is reduced to generate pentafluorosulfanyl (SF_5_) radicals, enabling direct access to pentafluorosulfanyl ketones, acetals, and BCP motifs. Mechanistic and DFT studies elucidate the reductive activation pathway of SF_6_, establishing a platform for constructing SF_5_‐functionalized architectures.

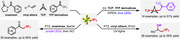

## 1 Introduction

**FIGURE 1:**
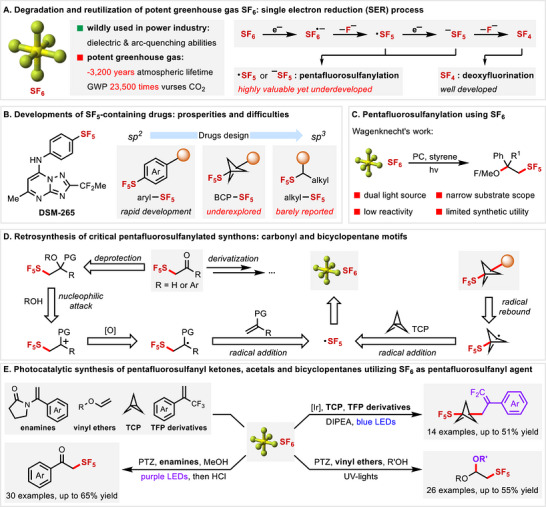
Development of diverse synthesis of pentafluorosulfanyl scaffolds. (A) Degradation and reutilization of potent greenhouse gas SF_6_: single electron reduction (SER) process. (B) Developments of SF_5_‐containing drugs: prosperities and difficulties. (C) Pentafluorosulfanylation using SF_6_. (D) Retrosynthesis of critical pentafluorosulfanylated synthons: carbonyl and bicyclopentane motifs. (E) Photocatalytic synthesis of pentafluorosulfanyl ketones, acetals and bicyclopentanes utilizing SF_6_ as pentafluorosulfanyl agent.

Sulfur hexafluoride (SF_6_) is a chemically inert and thermally stable gas renowned for its exceptional dielectric and arc‐quenching properties, making it indispensable in high‐voltage power transmission systems. However, SF_6_ poses significant environmental concerns due to its extremely long atmospheric lifetime (∼3200 years) and a global warming potential approximately 23 500 times higher than that of CO_2_ [1]. Therefore, developing strategies for the valorization and reutilization of SF_6_ is critically important for advancing sustainable and environmentally responsible technologies. Traditional degradation approaches, including pyrolysis and plasma discharge [2, 3, 4, 5, 6, 7], have been applied to the decomposition of SF_6_. However, these methods generally suffer from high energy demands and considerable chemical waste generation. Furthermore, the byproducts formed during SF_6_ degradation are rarely harnessed for subsequent chemical valorization. Despite its pronounced chemical inertness, SF_6_ can be activated via single electron reduction (SER) through photocatalytic or electrochemical pathways, generating sulfur hexafluoride radical anions [8, 9, 10]. These species can undergo continuous fragmentation to yield several active species such as SF_5_ radicals and SF_4_, offering an opportunity to exploit SF_6_ as a pentafluorosulfanylation or fluorination reagent (Figure 1A). This strategy not only enables the degradation of SF_6_ under mild reaction conditions but also facilitates its effective reutilization. Nevertheless, while SF_6_ has been extensively explored as a fluorinating reagent [11, 12, 13, 14], its direct application as a pentafluorosulfanylation reagent remains underdeveloped, underscoring the significance and potential impact of this transformation [7].

Among the various fluorinated functional groups, the pentafluorosulfanyl (SF_5_) group stands out as a unique bioelectronic isostere of motifs such as nitro (NO_2_), tert‐butyl (^t^Bu), and trifluoromethyl (CF_3_) groups [15, 16, 17, 18, 19, 20, 21]. Sterically, the SF_5_ group occupies a size range between ^t^Bu and CF_3_. Its distinct octahedral geometry, high electronegativity, and pronounced lipophilicity have earned it the moniker “super‐trifluoromethyl” [22]. Relative to the CF_3_ group, the SF_5_ group exhibits stronger electron‐withdrawing capability (σ_p_ = +0.68 vs +0.54) and increased lipophilicity (π = 1.51 vs 1.09) [23, 24, 25, 26]. Owing to the high bond dissociation energy of the S─F bonds, the SF_5_ group exhibits exceptional stability and is generally regarded as a terminal functional motif in synthetic chemistry. To date, only limited examples of transformations involving aryl‐SF_5_ groups have been reported, while analogous studies on alkyl‐SF_5_ derivatives remain unexplored [27, 28, 29, 30]. Such chemical inertness provides a strong rationale for the incorporation of SF_5_ groups to improve the metabolic stability of drug molecules. These attributes make SF_5_‐containing compounds highly attractive scaffolds in the design of biologically active molecules and next‐generation fluorinated drugs. While several drug candidates, such as the antimalarial agent DSM‐265 [31], feature aromatic SF_5_ groups, the synthesis of alkyl‐SF_5_ analogs remains challenging due to the limited accessibility of aliphatic substrates for oxidative fluorination [32]. In parallel, bicyclo[1.1.1]pentane (BCP) has gained prominence as the *para*‐disubstituted phenyl bioisostere with improved pharmacokinetic properties [33, 34, 35, 36, 37, 38]. Incorporating the SF_5_ group into BCP frameworks (BCP‐SF_5_) would further expand the utility of SF_5_ chemistry in medicinal applications. Therefore, developing efficient and versatile synthetic routes for BCP‐SF_5_ and alkyl‐SF_5_ motifs is of great importance (Figure 1B). Compared with conventional reagents such as SF_5_Cl or N‐SF_5_ imines [39, 40, 41, 42, 43, 44, 45, 46, 47, 48, 49, 50, 51, 52], SF_6_ provides significant advantages in terms of cost (∼$51–62 per kg), safety and atom economy, positioning it as a compelling alternative for industrial applications [53]. To date, the only reported example, demonstrated by Wagenknecht and co‐workers, achieves SF_5_‐functionalization of 1,1‐disubstituted styrenes via a dual etherification/fluorination strategy, yielding rigid structures with limited downstream synthetic flexibility (Figure 1C) [54, 55, 56, 57]. Given our ongoing efforts in SF_6_ activation and transformation chemistry [58, 59, 60, 61], we sought to address this limitation by designing substrates that could facilitate the synthesis of structurally simpler, more synthetically versatile, and higher‐value SF_5_‐containing building blocks. Such advances would significantly enhance the synthetic applicability and practical relevance of SF_6_‐based pentafluorosulfanylation strategies.

Ketones and aldehydes are fundamental synthetic building blocks that participate in a wide range of chemical transformations, including oxidation, reduction, Michael addition, and Wittig reactions [62, 63, 64, 65, 66]. Their versatility and reactivity place them at the core of synthetic methodology. In this context, the development of α‐pentafluorosulfanyl ketones and aldehydes holds significant promise for the design of new SF_5_‐containing pharmaceuticals. Based on retrosynthetic analysis, we strategically targeted the synthesis of pentafluorosulfanylated acetals using SF_6_ as an SF_5_ source (Figure 1D). This strategy relies on the photocatalytic single electron reduction of SF_6_ to generate pentafluorosulfanyl radicals, followed by radical addition to vinyl ethers or enamines to afford the desired adducts, which can then be transformed into the corresponding carbonyl compounds. Moreover, given the emerging importance of SF_5_‐BCP motifs in drug discovery, we further envisioned accessing these high‐value scaffolds directly via radical rearrangement of SF_5_ intermediates with tricyclo[1.1.1.0^1,3^]pentane (TCP), providing a one‐step route to structurally advanced SF_5_‐functionalized scaffolds [67, 68].

Given the broad synthetic utility of α‐pentafluorosulfanyl ketones and aldehydes, along with the growing importance of BCP‐SF_5_ motifs in fluorinated pharmaceuticals discovery, herein, we report a practical and sustainable method for constructing a range of pentafluorosulfanyl‐containing building blocks using sulfur hexafluoride (SF_6_) as the SF_5_ source (Figure 1E). This strategy enables the synthesis of α‐pentafluorosulfanyl ketones and acetals from enamines and vinyl ethers, respectively, with subsequent derivatization affording compounds such as α‐SF_5_‐substituted aldehydes. In parallel, the use of DIPEA as reductants facilitates the direct synthesis of BCP‐SF_5_ compounds from trifluoropropylene derivatives under mild conditions. This method not only offers an efficient route to both BCP‐SF_5_ and alkyl‐SF_5_ frameworks but also advances the valorization of SF_6_, the potent greenhouse gas, by incorporating it into high‐value organofluorine scaffolds relevant to medicinal chemistry. Furthermore, mechanistic investigations supported by density functional theory (DFT) calculations confirm the generation of SF_5_ radicals under the reaction conditions, providing strong evidence for a reductive activation pathway of SF_6_. These findings offer valuable insight into the molecular‐level transformation of SF_6_ and establish a theoretical foundation for its future application in green fluorine chemistry.

## 2 Results and Discussion

Building on our strategy for photocatalytic pentafluorosulfanylation using sulfur hexafluoride, we envisioned that α‐pentafluorosulfanyl ketones could be accessed by exploiting the hydrolytic lability of *N*,*O*‐acetal intermediates. Specifically, we proposed that 1‐aryl enamines could undergo dual functionalization across the C═C bond to furnish the desired products. Guided by this rationale, we systematically investigated and identified the optimal reaction conditions: enamine 1‐(1‐phenylvinyl)pyrrolidin‐2‐one **1a** as the substrate, 10‐phenyl‐10H‐phenothiazine (PTZ) as the photocatalyst, tetrabutylammonium chloride (TBAC) as an additive, methanol as the nucleophile, and ethyl acetate as the solvent, under purple LEDs irradiation followed by a one‐pot acidic hydrolysis. Under these conditions, the target α‐pentafluorosulfanyl ketone was obtained in 68% yield (65% for isolating). Variation of individual parameters revealed several factors critical to the reaction outcome. The omission of TBAC significantly diminished the yield, likely due to reduced substrate solubility and increased system acidity, which favored the formation of hydrolyzed byproducts such as acetophenone (Table 1, **entry 2**). Notably, the absence of methanol completely suppressed product formation, underscoring its essential role as a nucleophile (**entry 2**). Substituting PTZ with Ir‐based photocatalysts possessing lower reduction potentials failed to yield the desired product, indicating that a strongly reducing photocatalyst is required (**entry 3**). The use of etheric or highly polar solvents also resulted in diminished yields (**entries 5**–**7**). Furthermore, irradiation with 365 or 460 nm LEDs led to direct decomposition of the enamine substrate, producing acetophenone as the major byproduct (**entries 8**–**9**).

**TABLE 1:** Initial attempt and optimization of the reaction conditions.

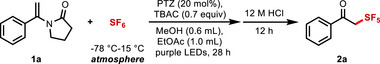		
Entry	Deviation from the standard condition^a^	yield^b^
1	None	68(65)^c^
2	No TBAC	45%
3	No MeOH	N.R.
4	Ir(ppy)_3_ instead of PTZ	N.R.
5	Dioxane instead of EtOAc	52%
6	MeCN instead of EtOAc	39%
7	DMF instead of EtOAc	trace
8	365 nm LEDs instead of 380‐400 nm LEDs	N.D.
9	460 nm LEDs instead of 380‐400 nm LEDs	trace

^a^ Unless otherwise noted, the reaction conditions were as follows: 1a (0.1 mmol, 1 equiv), SF_6_ (atmosphere, charged at −78°C), PTZ (20 mol%), MeOH (0.6 mL), EtOAc (1.0 mL), 380–400 nm purple LEDs (24 W), 15°C, 28 h; HCl (12 M, 0.6 mL) for 12 h.

^b^ Yield was determined by crude ^19^F NMR spectroscopy using PhCF_3_ as an internal standard.

^c^ Isolated yield was given.

**FIGURE 2:**
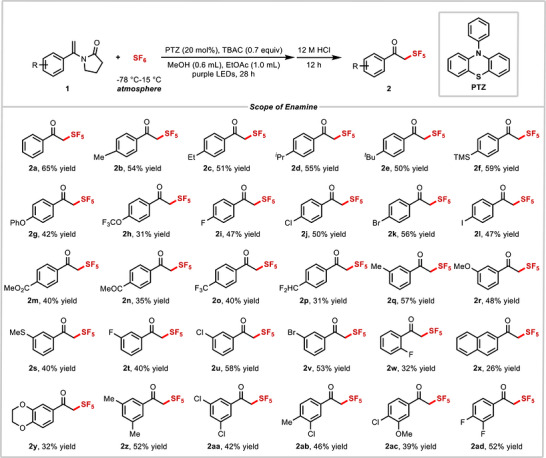
Photocatalytic pentafluorosulfanylation of enamines with SF_6_. Reaction conditions: *^a^* Isolated yields are given, **1** (0.1 mmol, 1.0 equiv), SF_6_ (atmosphere, charged at −78°C), PTZ (20 mol%), TBAC (0.07 mmol, 0.7 equiv), MeOH (0.6 mL), EtOAc (1.0 mL), under 380–400 nm purple LEDs (24 W) irradiation at 15°C for 28 h, then HCl (12 M, 0.6 mL) for 12 h. Yield was obtained by flash column chromatography.

**FIGURE 3:**
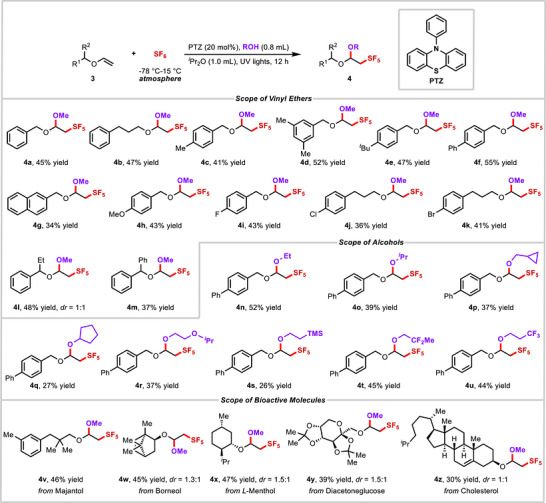
Photocatalytic pentafluorosulfanylation of vinyl ethers with SF_6_. Reaction conditions: Isolated yields are given, **3** (0.2 mmol, 1.0 equiv), SF_6_ (atmosphere, charged at −78°C), PTZ (20 mol%), MeOH (0.8 mL), *^i^* Pr_2_O (1.0 mL), under 365 nm ultraviolet lights (24 W) irradiation at 15°C for 12 h. Yield was obtained by flash column chromatography.

Following the identification of optimal reaction conditions, we explored the substrate scope using various aryl enamine derivatives to evaluate the functional group tolerance of this catalytic system as shown in Figure 2. Electron‐donating substituents, including methyl (**2b**), ethyl (**2c**), isopropyl (**2d**), *tert*‐butyl (**2e**), trimethylsilyl (**2f**) and phenoxy (**2** **g**) groups, were well tolerated under the reaction conditions, affording the desired products in moderate to good yields (31%–59%). Notably, *para*‐alkoxy enamines exhibited diminished reactivity, likely due to increased electron density promoting hydrolytic degradation. Electron‐withdrawing substituents, such as fluoro (**2i**), chloro (**2j**), bromo (**2k**), iodine (**2l**), ester (**2m**), acyl (**2n**), and fluoroalkyl groups (**2o**, **2p**), also proved compatible with the transformation, affording the pentafluorosulfanyl product in 31%–56% yield. In addition to *para*‐substituents, *meta*‐positioned electron‐donating groups such as methyl (**2q**), alkoxy (**2r**), and thiomethyl (**2s**), as well as electron‐withdrawing groups, including halogens (**2t‐2v**), were well accommodated with moderate yield. In contrast, the *ortho*‐fluorinated substrate (**2w**) led to notably reduced yields (32% yield), likely due to steric hindrance. The fused ring substrate (**2x**) was also tolerated under the photocatalytic system, obtaining the target product with a yield of 26%. Moreover, the reaction also proceeded smoothly with multi‐substituted arenes (**2y‐2ad**), delivering the corresponding products in moderate yields ranging from 32% to 52%. We then explored the pentafluorosulfanylation of vinyl ether substrates, aiming to yield products that could serve as valuable synthons for α‐pentafluorosulfanyl aldehydes. Following systematic optimization, reaction conditions were slightly modified from those optimized for enamine substrates. Under these conditions, we subsequently examined the substrate scope of vinyl ethers (Figure 3). First, ((vinyloxy)methyl)benzene (**4a**) was chosen as the model substrate, affording the dual‐functionalized product with 45% yield. Extending the alkyl chain had minimal effect on reactivity, while (3‐(vinyloxy)propyl)benzene (**4b**) yielded the desired product moderately. A variety of alkyl‐substituted aromatic vinyl ethers, such as methyl (**4c**, **4d**), *tert*‐butyl (**4e**), and phenyl (**4f**), underwent smooth pentafluorosulfanylation to afford the desired products in 41%–55% yields. A slight reduction in efficiency was observed for the fused‐ring derivative (**4** **g**), which gave a 34% yield. Notably, in contrast to the enamines, the *para*‐methoxy substrate (**4** **h**) performed well, likely due to the better stability of the vinyl ether scaffold.

Halogenated substrates, including fluoro (**4i**), chloro (**4j**), and bromo (**4k**) derivatives, also reacted effectively, delivering products in 36%–43% yield. Substrates bearing increased steric bulk at the benzylic position, such as ethyl (**4l**) and phenyl (**4m**) analogs, were also well tolerated. The scope of alcohol nucleophiles was subsequently evaluated. Both linear and branched aliphatic alcohols, including ethanol (**4n**), isopropanol (**4o**), cyclopropylmethanol (**4p**) and cyclopentanol (**4q**), participated successfully, affording products in 27%–52% yields. Additionally, heteroatom‐containing nucleophiles, such as isopropoxyethanol (**4r**), silanol derivatives (**4s**), and fluorinated alcohols (**4t**, **4u**), also proved compatible, further highlighting the broad applicability of this photocatalytic protocol. To further demonstrate the potential of this pentafluorosulfanylation method for drug modification, we applied the reaction system to vinyl ethers derived from biopharmaceutical molecules and macromolecular fragments. The results showed that various vinyl ether derivatives incorporating biologically active building blocks, such as majantol (**4v**), borneol(**4w**), *L*‐menthol (**4x**), diacetoneglucose (**4y**), and cholesterol(**4z**) successfully afforded the target products in 30%–47% yields, which demonstrates application potential of incorporating pentafluorosulfanyl group in commercially available drugs and natural products.

**FIGURE 4:**
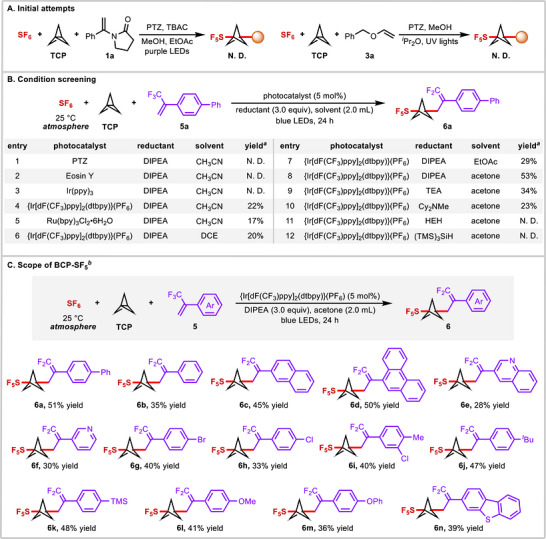
Direct construction of pentafluorosulfanylated bicyclo[1.1.1]pentane Motifs with SF_6_. (A) Initial attempts. (B) Condition screening. (C) Scope of BCP‐SF_5_. *^a^* The yields were determined by crude ^1^H NMR spectroscopy using PhOMe as internal standard. *^b^* Isolated yields are given, **5** (0.3 mmol, 3.0 equiv), SF_6_ (atmosphere, charged at 25°C), {Ir[dF(CF_3_)ppy]_2_(dtbpy)}(PF_6_) (5 mol%), TCP (1.0 M in Et_2_O, 0.1 mmol, 1.0 equiv), DIPEA (0.3 mmol, 3.0 equiv), acetone (2.0 mL), under 450–465 nm blue LEDs irradiation (40 W) at 25°C for 24 h. Yield was obtained by HPLC Thermo Scientific UltiMate 3000 (Shimadzu Shim‐pack PRC‐ODS).

Encouraged by the broad substrate scope and high efficiency of the pentafluorosulfanylation protocol, we next extended this strategy to access BCP‐SF_5_ scaffolds directly. Given the well‐documented radical‐trapping ability of tricyclo[1.1.1.0^1,3^]pentane (TCP), we designed the cascade reaction by introducing TCP intothe pentafluorosulfanylation systems of enamines or vinyl ethers. However, no desired SF_5_‐substituted bicyclopentane products were observed under these conditions (Figure 4A). This failure was likely attributable to the enamines or vinyl ethers acting as sacrificial reductants within the photocatalytic cycle, thereby attenuating the generation rate and concentration of pentafluorosulfanyl radicals. To overcome this limitation, we proposed a modified approach that decouples the formation of SF_5_ radicals from substrate oxidation by introducing an external reductant into the photocatalyst successfully enabled the formation of the desired three‐component product in 22% yield (Figure 4B, **entries 1**–**5**). No by‐products arising from the direct addition of SF_5_ radicals to trifluoropropene were detected, as confirmed by ^19^F NMR analysis of the crude reaction mixture and high‐resolution mass spectrometry (HRMS). Subsequent solvent screening revealed that acetone provided the highest compatibility for this transformation (Figure 4B **, entries 6–8**). Final optimization of the reducing agent identified DIPEA as the most effective (Figure 4B, **entries 9**–**12**), delivering the desired product in 53% yield (51% isolated). With the optimized conditions established, we next explored the substrate scope using a series of 2‐aryl trifluoropropenes (Figure 4C). The reaction exhibited good tolerance toward substrates bearing a fused ring (**6b**–**6d**), affording the corresponding three‐component adducts in yields of up to 50%. Heteroaryl‐substituted olefins, including 2‐quinolyl (**6e**) and 2‐pyridyl (**6f**) derivatives, also transformed successfully, albeit with slightly diminished yields. Substrates bearing electron‐withdrawing groups on the aryl ring, such as bromine (**6g**) and chlorine (**6h**, **6i**), were well tolerated. Moreover, electron‐rich aryl substituents, including *tert*‐butyl (**6j**), trimethylsilyl (**6k**), methoxy (**6l**), phenoxy (**6m**), and thioether (**6n**) groups, were compatible under the reaction conditions, delivering the desired products in moderate yields (36%–48%). These findings underscore the versatility of the developed methodology, offering a concise and efficient strategy for the direct synthesis of BCP‐SF_5_ scaffolds.

**FIGURE 5:**
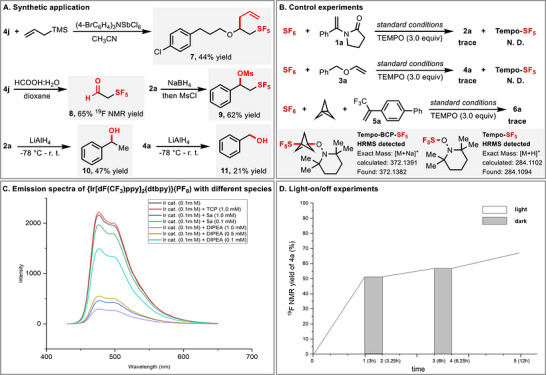
Synthetic application and mechanism investigation. (A) Synthetic application. (B) Control experiments. (C) Emission spectra of {Ir[dF(CF_3_)ppy]_2_(dtbpy)}(PF_6_) with different species. (D) Light‐on/off experiments.

To evaluate the practicability of the strategy, we then carried out multiple derivatization probes of the pentafluorosulfanylated product (Figure 5A). The nucleophilic substitution of pentafluorosulfanylated acetal **4j** with allylsilane under ammonium salt catalysis furnished the corresponding homoallyl ether in 44% yield. Additionally, treatment under acidic conditions afforded α‐pentafluorosulfanyl acetaldehyde, a valuable synthon, with a ^19^F NMR yield of 65%, offering a new route to access pentafluorosulfanyl motifs. Furthermore, α‐pentafluorosulfanylated ketone **2a** underwent sequential reduction and protection to deliver β‐pentafluorosulfanyl alcohol in a total yield of 62%. However, in the presence of the strong reducing agent LiAlH_4_, both **2a** and **4a** suffered from depentafluorosulfanylation, affording the corresponding benzyl alcohols **10** and **11**. Overall, these derivatizations highlight the versatility of sulfur hexafluoride as a viable reagent for the construction of diverse pentafluorosulfanyl‐containing compounds.

To gain more insights into the reaction mechanism, control experiments were conducted (Figure 5B). The addition of stoichiometric TEMPO to the reaction mixture resulted in only trace product formation for both enamines and vinyl ethers, consistent with the involvement of radical intermediates. However, none SF_5_‐abstraction product was detected, possibly due to the relatively low concentration of SF_5_ radicals. This mechanistic proposal was further supported by high resolution mass spectrometry (HRMS), which confirmed the formation of TEMPO‐SF_5_ and TEMPO‐BCP‐SF_5_ adducts in the reaction with external reductant DIPEA, aligning with our hypothesis. Additional fluorescence quenching studies revealed that both DIPEA and substrate **5a** could quench the excited‐state photocatalyst, with DIPEA identified as the more efficient electron donor based on Stern‐Volmer analysis (Figure 5C and Figure S4). Finally, light on/off experiments confirmed that continuous irradiation is required for product formation, thereby affirming that the transformation proceeds through a photoinduced pathway rather than a radical chain mechanism (Figure 5D).

**FIGURE 6:**
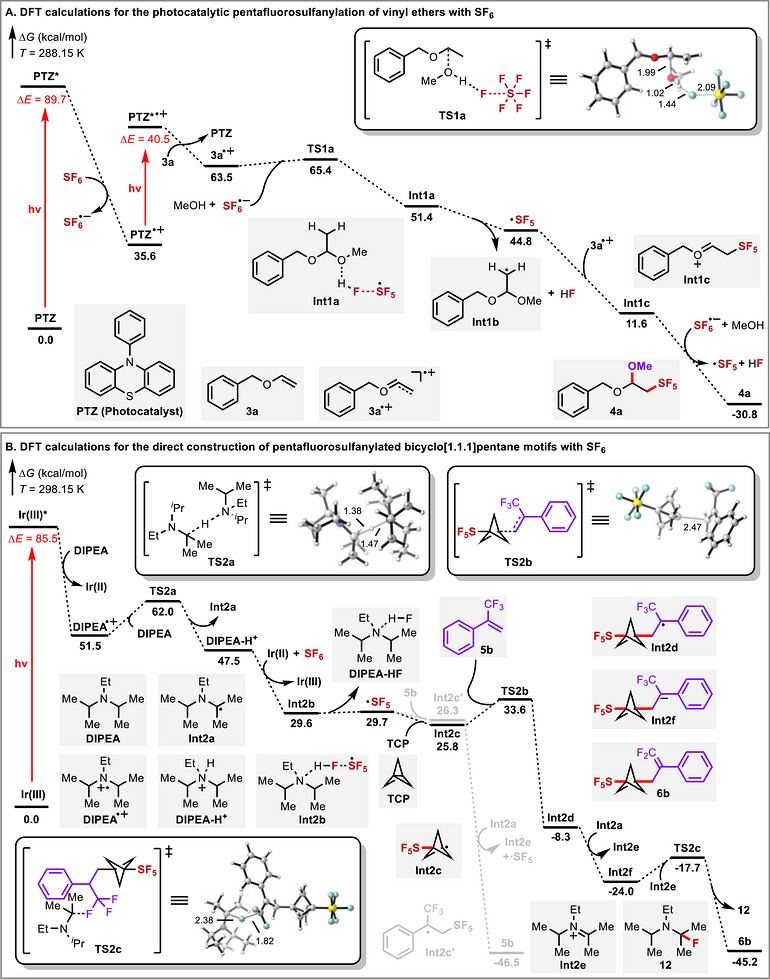
Potential energy surface for the pentafluorosulfanylation reactions. (A) DFT calculations for the photocatalytic pentafluorosulfanylation of vinyl ethers with SF_6_. (B) DFT calculations for the direct construction of pentafluorosulfanylated bicyclo[1.1.1]pentane motifs with SF_6_. Ground states was calculated at ωB97XD/def2‐TZVPP‐PCM//ωB97XD/def2‐SVP‐PCM level, and single‐point energy of excited state was calculated at ωB97XD/def2‐TZVPP‐PCM level using time‐dependent density functional theory (TD‐DFT).

Based on experimental results and previous literature, we hypothesize a coupling mechanism between the SF_5_ radical and the radical cation of enamines or vinyl ethers. Using DFT calculations, we gained insights into the mechanism for the synthesis of SF_5_ synthons, with the potential energy surface of vinyl ether **3a** depicted in Figure 6A. The calculation results reveal that the photocatalyst PTZ is sensitized to its excited state and then oxidized by SF_6_ to generate SF_6_^•–^. PTZ^•+^ is then further sensitized to a doublet excited state, which oxidizes substrate **3a** to its radical cation **3a** ^•+^ [69]. Next, **3a** ^•+^ undergoes transition state **TS1a** with a low energy barrier of 1.9 kcal/mol to form an open‐shell singlet intermediate **Int1a**, which decomposes to yield SF_5_^•^. This key intermediate SF_5_^•^ undergoes a barrierless combination with **3a** ^•+^ to generate intermediate **Int1c**. Through a nucleophilic addition step involving MeOH and SF_6_^•–^, **Int1c** transforms into the final product **4a**, while the intermediate SF_5_^•^ is regenerated simultaneously. The DFT calculations support our hypothesis of SF_5_^•^‐**3a** ^•+^ coupling and align with experimental results.

The reaction involving trifluoropropylene derivatives and TCP is also considered, as illustrated in Figure 6B. At the start of the potential energy surface, the excited photocatalyst oxidizes DIPEA to its radical cation DIPEA^•+^. Proton transfer (ΔG^‡^ = 10.5 kcal/mol) follows, assisted by another molecule of DIPEA acting as a base, yielding **Int2a** and DIPEA‐H^+^. This facilitates the generation of SF_5_^•^ from SF_6_^•–^. TCP captures SF_5_^•^ to form intermediate **Int2c**, which then undergoes an addition transition state **TS2b** with an energy barrier of 7.8 kcal/mol. An alternative pathway involves the formation of **Int2c′** following the addition of the SF_5_^•^ radical to **5b**. However, **Int2c′** would subsequently undergo reduction by **Int2a**, leading to the regeneration of **5b**, thereby rendering the overall transformation unsuccessful. In the favored pathway, the resulting radical intermediate **Int2d** is reduced by **Int2a** to form the anion intermediate **Int2f**. After a fluoride transfer step with an energy barrier of 6.3 kcal/mol, **Int2f** finally transforms into the pentafluorosulfanylation product **6b**, with the generation of by‐product **12** observed in the experiment (Page **S24**). The DFT calculation results confirm the crucial role of DIPEA in producing SF_5_^•^, deepening our understanding of the mechanism.

## 3 Conclusion

This study introduces a novel pentafluorosulfanylation strategy that leverages SF_6_ as an SF_5_ source for the synthesis of structurally diverse pentafluorosulfanyl scaffolds, including ketones, acetals, aldehydes and BCP derivatives, addressing longstanding challenges in both SF_5_ group incorporation and SF_6_ remediation. Utilizing a photocatalytic single electron reduction approach, the method proceeds under mild conditions with broad functional group tolerance and high efficiency, thereby enriching the synthetic repertoire for SF_5_‐containing pharmaceutical analogs. Mechanistic studies, corroborated by DFT calculations, reveal the in situ generation of SF_5_ radicals, offering compelling insights into the reductive activation pathway of SF_6_. Beyond demonstrating the synthetic utility of SF_5_‐functionalized motifs in medicinal chemistry, this work highlights a promising avenue for the valorization of SF_6_, a potent greenhouse gas, by transforming it into valuable organofluorine frameworks.

## Conflicts of Interest

Partial of this study has been submitted for a patent application by the authors and the same institute: Y. Zhao, X.‐S. Wang, R.‐X. Jin, etc. State grid Anhui electric power Research Institute, CN119504535 A, 2025.

## Supporting information




**Supporting File**: advs75663‐sup‐0001‐SuppMat.pdf.

## Data Availability

The data that support the findings of this study are available in the Supporting Information of this article.

## References

[1] O. Hodnebrog , M. Etminan , J. S. Fuglestvedt , et al., “Global Warming Potentials and Radiative Efficiencies of Halocarbons and Related Compounds: A Comprehensive Review,” Reviews of Geophysics 51 (2013): 300–378.

[2] L. Zamostna , T. Braun , and B. Braun , “S─F and S─C Activation of SF_6_ and SF_5_ Derivatives at Rhodium: Conversion of SF_6_ Into H_2_S,” Angewandte Chemie International Edition 53 (2014): 2745–2749, 10.1002/anie.201308254. 24453166

[3] F. Buss , C. Mueck‐Lichtenfeld , P. Mehlmann , and F. Dielmann , “Nucleophilic Activation of Sulfur Hexafluoride: Metal‐Free, Selective Degradation by Phosphines,” Angewandte Chemie International Edition 57 (2018): 4951–4955. 29437280 10.1002/anie.201713206

[4] Y. Li , X. Zhang , S. Xiao , et al., “Decomposition Properties of C4F7N/N_2_ Gas Mixture: An Environmentally Friendly Gas to Replace SF_6_ ,” Industrial & Engineering Chemistry Research 57 (2018): 5173–5182.

[5] P. Tomar , T. Braun , and E. Kemnitz , “Photochemical Activation of SF_6_ by N‐heterocyclic Carbenes to Provide a Deoxyfluorinating Reagent,” Chemical Communications 54 (2018): 9753–9756, 10.1039/C8CC05494K. 30109328

[6] P. Rotering , C. Mueck‐Lichtenfeld , and F. Dielmann , “Solvent‐free Photochemical Decomposition of Sulfur Hexafluoride by Phosphines: Formation of Difluorophosphoranes as Versatile Fluorination Reagents,” Green Chemistry 24 (2022): 8054–8061, 10.1039/D2GC02172B.

[7] A. Taponard , T. Jarrosson , L. Khrouz , M. Médebielle , J. Broggi , and A. Tlili , “New SF_5_‐Based Reagent Enables Deoxyfluorination and Pentafluorosulfanylation Reactions,” Angewandte Chemie International Edition 61 (2022): 202204623. 10.1002/anie.202204623 35471641

[8] C.‐L. Chen and P. J. Chantry , “Photon‐Enhanced Dissociative Electron Attachment in SF_6_ and Its Isotopic Selectivity,” The Journal of Chemical Physics 71 (1979): 3897–3907, 10.1063/1.438158.

[9] G. E. Streit , “Negative Ion Chemistry and the Electron Affinity of SF_6_ ,” The Journal of Chemical Physics 77 (1982): 826–833, 10.1063/1.443898.

[10] J. Troe , T. M. Miller , and A. A. Viggiano , “Low‐Energy Electron Attachment to SF_6_. II. Temperature and Pressure Dependences of Dissociative Attachment,” The Journal of Chemical Physics 127 (2007): 244304, 10.1063/1.2804762. 18163672

[11] T. A. McTeague and T. F. Jamison , “Photoredox Activation of SF_6_ for Fluorination,” Angewandte Chemie International Edition 55 (2016): 15072–15075, 10.1002/anie.201608792. 27813242

[12] S. J. Kim , Y. Khomutnyk , A. Bannykh , and P. Nagorny , “Synthesis of Glycosyl Fluorides by Photochemical Fluorination With Sulfur(VI) Hexafluoride,” Organic Letters 23 (2021): 190–194, 10.1021/acs.orglett.0c03915. 33354969 PMC7783729

[13] S. Kim and P. Nagorny , “Electrochemical Synthesis of Glycosyl Fluorides Using Sulfur(VI) Hexafluoride as the Fluorinating Agent,” Organic Letters 24 (2022): 2294–2298, 10.1021/acs.orglett.2c00431. 35298181 PMC10543653

[14] Y. Zhao , F. Ma , Y. Chen , et al., “Photoinduced SF_6_ Degradation for Deoxyfluorination of Propargyl Alcohols,” Organic & Biomolecular Chemistry 23 (2025): 1094–1097, 10.1039/D4OB01839G. 39699173

[15] W. A. Sheppard , “The Electrical Effect of the Sulfur Pentafluoride Group,” Journal of the American Chemical Society 84 (1962): 3072–3076, 10.1021/ja00875a007.

[16] K. Müller , C. Faeh , and F. Diederich , “Fluorine in Pharmaceuticals: Looking Beyond Intuition,” Science 317 (2007): 1881–1886. 17901324 10.1126/science.1131943

[17] K. L. Kirk , “Fluorination in Medicinal Chemistry: Methods, Strategies, and Recent Developments,” Organic Process Research & Development 12 (2008): 305–321, 10.1021/op700134j.

[18] Y. Zhou , J. Wang , Z. Gu , et al., “Next Generation of Fluorine‐Containing Pharmaceuticals, Compounds Currently in Phase II‐III Clinical Trials of Major Pharmaceutical Companies: New Structural Trends and Therapeutic Areas,” Chemical Reviews 116 (2016): 422–518. 26756377 10.1021/acs.chemrev.5b00392

[19] S. Codony , E. Pujol , J. Pizarro , et al., “2‐Oxaadamant‐1‐yl Ureas as Soluble Epoxide Hydrolase Inhibitors: In Vivo Evaluation in a Murine Model of Acute Pancreatitis,” Journal of Medicinal Chemistry 63 (2020): 9237–9257, 10.1021/acs.jmedchem.0c00310. 32787085 PMC7755424

[20] J. M. Salamoun , C. J. Garcia , S. R. Hargett , et al., “6‐Amino[1,2,5]oxadiazolo[3,4‐b] pyrazin‐5‐ol Derivatives as Efficacious Mitochondrial Uncouplers in STAM Mouse Model of Nonalcoholic Steatohepatitis,” Journal of Medicinal Chemistry 63 (2020): 6203–6224. 32392051 10.1021/acs.jmedchem.0c00542 PMC11042500

[21] H. Tang , K. Jensen , E. Houang , et al., “Discovery of a Novel Class of _D_‐Amino Acid Oxidase Inhibitors Usingthe Schrödinger Computational Platform,” Journal of Medicinal Chemistry 65 (2022): 6775–6802, 10.1021/acs.jmedchem.2c00118. 35482677

[22] M. Magre , S. Ni , and J. Cornella , “(Hetero)aryl‐S^VI^ Fluorides: Synthetic Development and Opportunities,” Angewandte Chemie International Edition 61 (2022): 202200904. 10.1002/anie.202200904 PMC9322316 35303387

[23] H. Ladenheim , E. M. Loebl , and H. Morawetz , “Reactions of Polymers With Reagents Carrying Two Interacting Groups. I. The Quaternization of Poly‐(4‐vinylpyridine) With Bromoacetic Acid^1,2^ ,” Journal of the American Chemical Society 81 (1959): 20–23, 10.1021/ja01510a006.

[24] J. O. Edwards and R. G. Pearson , “The Factors Determining Nucleophilic Reactivities,” Journal of the American Chemical Society 84 (1962): 16–24, 10.1021/ja00860a005.

[25] M. F. Sowaileh , R. A. Hazlitt , and D. A. Colby , “Application of the Pentafluorosulfanyl Group as a Bioisosteric Replacement,” Chemmedchem 12 (2017): 1481–1490, 10.1002/cmdc.201700356. 28782186

[26] Y. Xie , J. Iwata , T. Matsumoto , et al., “Hydrophobicity of the Pentafluorosulfanyl Group in Side Chains of Polymethacrylates by Evaluation With Surface Free Energy and Neutron Reflectivity,” Langmuir 38 (2022): 6472–6480, 10.1021/acs.langmuir.2c00690. 35544954

[27] J.‐Y. Li , Y. Liu , D. Sun , et al., “Radical 1,3‐Difunctionalization of β,γ‐Unsaturated Ketones via Concomitant 1,2‐Carbonyl Migration: An Entrance to β‐SF_5_‐/β‐CF_3_SF_4_ Ketones,” Journal of the American Chemical Society 147 (2025): 26124–26132, 10.1021/jacs.5c09085. 40670305

[28] T. Uchikura , F. Akutsu , and T. Akiyama , “Electron Donor–Acceptor (EDA) Complex Mediated Visible‐Light Driven Sulfur–Fluorine Bond Reduction of Pentafluorosulfanyl Arenes Using Potassium Iodide,” Chemical Communications 61 (2025): 6328–6331, 10.1039/D5CC00764J. 40167480

[29] T. M. Nguyen , F. René , V. Bizet , and D. Cahard , “Understanding the Pentafluorosulfanyl Group and Property‐Driven Design of SF_5_‐Containing Compounds,” Chemical Society Reviews 55 (2026): 1333–1351, 10.1039/D5CS00566C. 41493022

[30] V. A. Béland , N. Nöthling , M. Leutzsch , and J. Cornella , “Activation and Catalytic Degradation of SF_6_ and PhSF_5_ at a Bismuth Center,” Journal of the American Chemical Society 146 (2024): 25409–25415. 39226694 10.1021/jacs.4c07044 PMC11421020

[31] J. D. Macdonald , S. C. Simon , C. Han , et al., “Discovery and Optimization of Salicylic Acid‐Derived Sulfonamide Inhibitors of the WD Repeat‐Containing Protein 5–MYC Protein–Protein Interaction,” Journal of Medicinal Chemistry 62 (2019): 11232–11259, 10.1021/acs.jmedchem.9b01411. 31724864 PMC6933084

[32] R. Kordnezhadian , B.‐Y. Li , A. Zogu , J. Demaerel , W. M. De Borggraeve , and E. Ismalaj , “Chemistry of Pentafluorosulfanyl Derivatives and Related Analogs: From Synthesis to Applications,” Chemistry: A European Journal 28 (2022): 202201491. 10.1002/chem.202201491 35781717

[33] A. F. Stepan , C. Subramanyam , I. V. Efremov , et al., “Application of the Bicyclo[1.1.1]Pentane Motif as a Nonclassical Phenyl Ring Bioisostere in the Design of a Potent and Orally Active γ‐Secretase Inhibitor,” Journal of Medicinal Chemistry 55 (2012): 3414–3424, 10.1021/jm300094u. 22420884

[34] G. M. Locke , S. S. R. Bernhard , and M. O. Senge , “Nonconjugated Hydrocarbons as Rigid‐Linear Motifs: Isosteres for Material Sciences and Bioorganic and Medicinal Chemistry,” Chemistry: A European Journal 25 (2019): 4590–4647. 30387906 10.1002/chem.201804225

[35] P. K. Mykhailiuk , “Saturated Bioisosteres of Benzene: Where to Go Next?,” Organic & Biomolecular Chemistry 17 (2019): 2839–2849, 10.1039/C8OB02812E. 30672560

[36] A. Fawcett , “Recent Advances in the Chemistry of Bicyclo‐ and 1‐Azabicyclo[1.1.0]butanes,” Pure and Applied Chemistry 92 (2020): 751–765, 10.1515/pac-2019-1007.

[37] E. G. Tse , S. D. Houston , C. M. Williams , et al., “Nonclassical Phenyl Bioisosteres as Effective Replacements in a Series of Novel Open‐Source Antimalarials,” Journal of Medicinal Chemistry 63 (2020): 11585–11601. 32678591 10.1021/acs.jmedchem.0c00746

[38] M. Golfmann and J. C. L. Walker , “Bicyclobutanes as Unusual Building Blocks for Complexity Generation in Organic Synthesis,” Communications Chemistry 6 (2023): 9. 36697911 10.1038/s42004-022-00811-3 PMC9837078

[39] G. Lefebvre , O. Charron , J. Cossy , and C. Meyer , “Radical Addition of SF_5_Cl to Cyclopropenes: Synthesis of (Pentafluorosulfanyl)Cyclopropanes,” Organic Letters 23 (2021): 5491–5495, 10.1021/acs.orglett.1c01840. 34170712

[40] J.‐Y. Shou , X.‐H. Xu , and F.‐L. Qing , “Chemoselective Hydro(Chloro)Pentafluorosulfanylation of Diazo Compounds With Pentafluorosulfanyl Chloride,” Angewandte Chemie International Edition 60 (2021): 15271–15275, 10.1002/anie.202103606. 33928731

[41] M. Birepinte , P. A. Champagne , and J. F. Paquin , “Photoinitiated *Anti*‐Hydropentafluorosulfanylation of Terminal Alkynes,” Angewandte Chemie International Edition 61 (2022): 202112575. 10.1002/anie.202112575 34716642

[42] Y. Kraemer , C. Ghiazza , A. N. Ragan , et al., “Strain‐Release Pentafluorosulfanylation and Tetrafluoro(aryl)Sulfanylation of 1.1.1 Propellane: Reactivity and Structural Insight,” Angewandte Chemie International Edition 61 (2022): 202211892. 10.1002/anie.202211892 PMC9828730 36137228

[43] J.‐Y. Shou and F.‐L. Qing , “Three‐Component Reaction of Pentafluorosulfanyl Chloride, Alkenes and Diazo Compounds and Synthesis of Pentafluorosulfanylfurans,” Angewandte Chemie International Edition 61 (2022): 202208860. 10.1002/anie.202208860 35942876

[44] J.‐Y. Shou , X.‐H. Xu , and F.‐L. Qing , “The Radical Reaction of Ethynylbenziodoxolone (EBX) Reagents With Pentafluorosulfanyl Chloride: New Approach to SF_5_‐substituted Alkynes,” Journal of Fluorine Chemistry 261–262 (2022): 110018, 10.1016/j.jfluchem.2022.110018.

[45] J. E. Erchinger , R. Hoogesteger , R. Laskar , et al., “EnT‐Mediated N–S Bond Homolysis of a Bifunctional Reagent Leading to Aliphatic Sulfonyl Fluorides,” Journal of the American Chemical Society 145 (2023): 2364–2374, 10.1021/jacs.2c11295. 36652725

[46] X. Zhao , J.‐Y. Shou , and F.‐L. Qing , “Iodopentafluorosulfanylation of[1.1.1]Propellane and Further Functionalizations,” Science China Chemistry 66 (2023): 2871–2877, 10.1007/s11426-023-1715-2.

[47] Y. Jiang , X. Meng , J. Zhang , G. Wu , X. Lin , and S. Guo , “Photo‐Induced Hydroxypentafluorosulfanylation of Alkenes With SF_5_Cl and Oxygen Gas and Their Further Derivatization,” Nature Communications 15 (2024): 9705, 10.1038/s41467-024-54015-5. PMC11550833 39521769

[48] Y. Kraemer , J. A. Buldt , W. Y. Kong , et al., “Overcoming a Radical Polarity Mismatch in Strain‐Release Pentafluorosulfanylation of [1.1.0]Bicyclobutanes: An Entryway to Sulfone‐ and Carbonyl‐Containing SF 5 ‐Cyclobutanes,” Angewandte Chemie International Edition 63 (2024): 202319930, 10.1002/anie.202319930. PMC11045327 38237059

[49] L. Wang and W. Qin , “Copper‐Initiated Regiodivergent Chloropentafluorosulfanylation of 1,3‐Enynes Under Substrate Control,” Organic Letters 26 (2024): 5049–5054, 10.1021/acs.orglett.4c01768. 38833632

[50] R. Li , C. Hu , C. Liu , et al., “Modular Access to N‐SF_5_ Azetidines,” Journal of the American Chemical Society 147 (2025): 34218–34224, 10.1021/jacs.5c10954. 40939176 PMC12443333

[51] Y. Yang , L. Han , L. Canavero , et al., “Strain‐Release‐Driven Synthesis of Pentafluorosulfanylated Four‐Membered Rings Under Energy Transfer Photocatalysis,” Journal of the American Chemical Society 147 (2025): 27905–27911, 10.1021/jacs.5c07138. 40698566

[52] Y. Yang , L. Han , L. Brettnacher , L. Canavero , and A. Tlili , “Synthetic Access to Persulfuranyl Scaffolds via Direct and Indirect Methods,” Chemical Reviews 125 (2025): 8426–8476, 10.1021/acs.chemrev.5c00131. 40608488

[53] D. Rombach and H. A. Wagenknecht , “Photochemical Activation of Sulfur Hexafluoride: A Tool for Fluorination and Pentafluorosulfanylation Reactions,” Synthesis‐Stuttgart 54 (2022): 4883–4894.

[54] D. Rombach and H.‐A. Wagenknecht , “Photoredox Catalytic Activation of Sulfur Hexafluoride for Pentafluorosulfanylation of α‐Methyl‐ and α‐Phenyl Styrene,” Chemcatchem 10 (2018): 2955–2961, 10.1002/cctc.201800501.

[55] D. Rombach and H. A. Wagenknecht , “Photoredox Catalytic α‐Alkoxypentafluorosulfanylation of α‐Methyl‐ and α‐Phenylstyrene Using SF 6,” Angewandte Chemie International Edition 59 (2020): 300–303, 10.1002/anie.201910830. 31680388 PMC6973110

[56] S. Klehenz , H. Kucher , and H. A. Wagenknecht , “Redox‐Convertible Groups to Expand the Substrate Scope for Pentafluorosulfanylation of Styrenes by Photocatalytic Activation of Sulfur Hexafluoride,” European Journal of Organic Chemistry 28 (2025): 202500478, 10.1002/ejoc.202500478.

[57] M. Flügge , S. Klehenz , S. Leidenheimer , D. Rombach , and H.‐A. Wagenknecht , “Photoredox Catalytic Hydropentafluorosulfanylation of Alkynes by Sulfur Hexafluoride,” JACS Au 6 (2026): 965–972. 41755855 10.1021/jacsau.5c01407 PMC12933376

[58] Y.‐X. Bi , Y. Zhao , Q.‐Q. Zhang , et al., “Visible‐Light‐Induced Oxidation of Silanes With SF_6_ for Efficient Synthesis of Siloxanes,” European Journal of Organic Chemistry 27 (2024): 202400451, 10.1002/ejoc.202400451.

[59] Y.‐L. Huang , Q.‐Q. Zhang , C.‐Y. Wang , Y. Zhao , and X.‐S. Wang , “Development of SF_6_ as a Formal Electrophilic Fluorinating Reagent for Photocatalyzed Oxidative Fluorination of Phosphine Oxides,” Organic Letters 26 (2024): 5776–5781, 10.1021/acs.orglett.4c01953. 38934518

[60] Y.‐F. Zhang , S. Zhu , Y.‐W. Zuo , H. Liu , R.‐X. Jin , and X.‐S. Wang , “Visible Light‐Induced Photocatalytic Deoxyfluorination of Benzyl Alcohol Using SF_6_ as a Fluorinating Reagent,” Green Chemistry 26 (2024): 10324–10329.

[61] Y.‐W. Zuo , Y. Zhao , Y.‐F. Zhang , et al., “Visible‐Light‐Induced Oxidative Decarboxylative Coupling of Phenylacetic Acid Derivatives Using SF_6_ as an Oxidant,” Organic Letters 26 (2024): 5652–5656, 10.1021/acs.orglett.4c01609. 38941116

[62] Z.‐Y. Liu , Y. He , Y.‐G. Yang , R.‐X. Jin , S. Zhu , and X.‐S. Wang , “Visible‐Light‐Induced Synthesis of Azlactone Monomers and Dimers Utilizing SF_6_ as both Condensation Agent and Oxidant,” Organic Letters 27 (2024): 41–45, 10.1021/acs.orglett.4c03700. 39721991

[63] S. G. Levine , “A New Aldehyde Synthesis,” Journal of the American Chemical Society 80 (1958): 6150–6151, 10.1021/ja01555a068.

[64] B. E. Maryanoff and A. B. Reitz , “The Wittig Olefination Reaction and Modifications Involving Phosphoryl‐Stabilized Carbanions. Stereochemistry, Mechanism, and Selected Synthetic Aspects,” Chemical Reviews 89 (1989): 863–927, 10.1021/cr00094a007.

[65] S. Chakraborty , P. Bhattacharya , H. Dai , and H. Guan , “Nickel and Iron Pincer Complexes as Catalysts for the Reduction of Carbonyl Compounds,” Accounts of Chemical Research 48 (2015): 1995–2003, 10.1021/acs.accounts.5b00055. 26098431

[66] X. Huang and S. Ma , “Allenation of Terminal Alkynes With Aldehydes and Ketones,” Accounts of Chemical Research 52 (2019): 1301–1312, 10.1021/acs.accounts.9b00023. 30985104

[67] E. Yen‐Pon , L. Li , G. Levitre , et al., “On‐DNA Hydroalkylation to Introduce Diverse Bicyclo [1.1.1]Pentanes and Abundant Alkyls via Halogen Atom Transfer,” Journal of the American Chemical Society 144 (2022): 12184–12191, 10.1021/jacs.2c03025. 35759692 PMC10412002

[68] X.‐T. Feng , Q.‐Q. Min , X. Zeng , H.‐Y. Zhao , and X. A. Zhang , “Controllable Diverse Construction of Gem‐Difluoroallylated Bicyclo [1.1.1]Pentanes and Cyclobutanes From [1.1.1]Propellane via Copper Catalysis,” ACS Catalysis 14 (2024): 5879–5887, 10.1021/acscatal.4c00281.

[69] F. Glaser , C. Kerzig , and O. S. Wenger , “Multi‐Photon Excitation in Photoredox Catalysis: Concepts, Applications, Methods,” Angewandte Chemie International Edition 59 (2020): 10266–10284, 10.1002/anie.201915762. 31945241

