# Supplementary material for: Photocatalytic Synthesis of Pentafluorosulfanyl Ketones, Acetals, and BCP Motifs Utilizing SF6
Source: Adv Sci (Weinh). 2026 May 15;13(44):e75663. doi: 10.1002/advs.75663 (PMC13336085; doi:10.1002/advs.75663)
Supplement: Supplementary file 1 [file ADVS-13-e75663-s001.pdf]

Supplementary Materials for

Photocatalytic Synthesis of Pentafluorosulfanyl Ketones, Acetals and BCP Motifs Utilizing SF_6

Chen-Hui Jiang,† Haoran Xu,† Yi-Gang Yang,† Xuan Nie,† Meng-Meng Zheng,† Yue Zhao, Ya-Wen Zuo, Wei-Ran Ren, Shan Zhu, Ruo-Xing Jin,* Xiao-Song Xue,* Xi-Sheng Wang*

[a] Chen-Hui Jiang, Xuan Nie, Xi-Sheng Wang
Department of Pharmacy, The First Affiliated Hospital of USTC, Division of Life Sciences and Medicine, University of Science and Technology of China, Hefei 230026, China.
E-mail: xswang77@ustc.edu.cn

[b] Chen-Hui Jiang, Yi-Gang Yang, Ya-Wen Zuo, Wei-Ran Ren, Ruo-Xing Jin, Xi-Sheng Wang.
Department of Chemistry, University of Science and Technology of China, Hefei 230026, China.
E-mail: xx520@mail.ustc.edu.cn

[c] Haoran Xu, Meng-Meng Zheng, Xiao-Song Xue
State Key Laboratory of Fluorine and Nitrogen Chemistry and Advanced Materials, Shanghai Institute of Organic Chemistry, University of Chinese Academy of Sciences, Chinese Academy of Sciences, 345 Lingling Road, Shanghai 200032, China
E-mail: xuexs@sioc.ac.cn

[d] Xiao-Song Xue
School of Chemistry and Materials Science, Hangzhou Institute for Advanced Study, University of Chinese Academy of Sciences, 1 Sub-Lane Xiangshan, Hangzhou 310024, China

[e] Yue Zhao, Shan Zhu
State grid Anhui electric power Research Institute, Hefei 230601, Anhui, China

†These authors contributed equally to this work.

*Correspondence to: xx520@mail.ustc.edu.cn, xuexs@sioc.ac.cn, xswang77@ustc.edu.cn.

This PDF file includes:

Supplementary Text
Figs. S1 to S222
Tables S1 to S13
References (1 to 9)

Tabel of Contents

General Information 3

Optimization of reaction conditions 4

Substrates synthesis 11

General procedure for the pentafluorosulfanylation reactions 15

Synthetic applications 17

Mechanism investigations 22

DFT calculations 28

References 29

NMR data for pentafluorosulfanyl compounds 101

Supplementary Figures 139

Supplementary Notes

General Information

NMR spectra were recorded on Bruker-400 MHz NMR spectrometer (400 MHz for ^{1}H; 101 MHz for ^{13}C and 376 MHz for ^{19}F), Bruker-500 MHz NMR spectrometer (500 MHz for ^{1}H; 126 MHz for ^{13}C and 470 MHz for ^{19}F), Bruker-600 MHz NMR spectrometer (600 MHz for ^{1}H; 151 MHz for ^{13}C and 565 MHz for ^{19}F). ^{1}H NMR chemical shifts were determined relative to internal $(CH_3)_4Si$ at δ 0.0 ppm or at the signal of a residual protonated solvent: $CDCl_3$ δ 7.26 ppm, DMSO δ 2.50 ppm. ^{13}C NMR chemical shifts were determined relative to $CDCl_3$ δ 77.16 ppm, DMSO δ 39.60 ppm. ^{19}F NMR chemical shifts were determined relative to $CFCl_3$ at δ 0.0 ppm. Data for ^{1}H, ^{13}C, ^{19}F NMR are recorded as follows: chemical shift (δ, ppm), multiplicity (s = singlet, d = doublet, t = triplet, m = multiplet, q = quartet et al.), integration, and coupling constant (Hz). High resolution mass spectra were recorded on P-SIMS-Gly of BrukerDaltonics Inc. using ESI-TOF (electrospray ionization-time of flight). DIPEA, Anhydrous EA, *i*Pr_2O and MeOH were purchased from J&K Chemicals. {Ir[dF(CF_3)ppy]$_2$(dtbpy)}(PF_6) and TBAC were purchased from leyan. Acetone were purchased from sinopharm. Trifluoromethyl alkenes, tricyclo[1.1.1.0^{1,3}]pentane (TCP), Vinyl amine derivatives and vinyl ether derivatives were synthesized via following methods described in this supporting information. Unless otherwise noted, all other reagents and starting materials were purchased from commercial sources and used without further purification.

Optimization of reaction conditions

Condition optimization for enamines

Table S1. Solvents screening

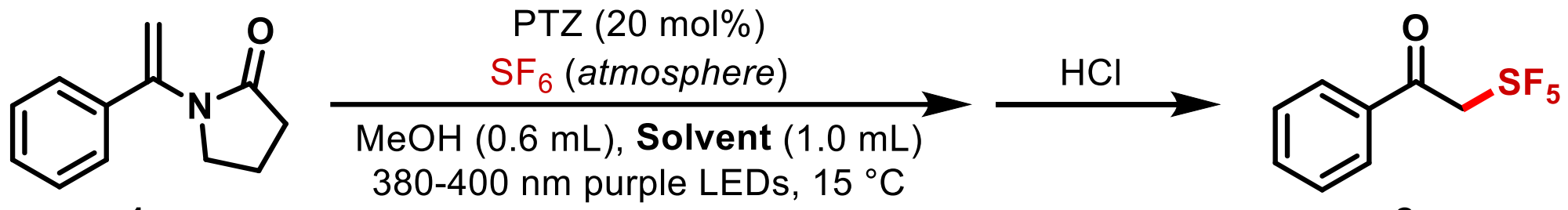

Entry[a]	solvent	yield[b]
1	MeCN	39%
2	CPME	21%
3	THF	33%
4	dioxane	38%
5	DMF	trace
6	toluene	39%
7	EA	45%

[a]Unless otherwise noted ,the reaction conditions were as follows: **1a** (0.1 mmol, 1 equiv), SF_6 (atmosphere charged at -78 °C), PTZ (20 mol%), MeOH (0.6 mL), **Solvent** (1.0 mL), 380-400 nm purple LEDs (24 W), 15 °C, 28 h, then HCl (12 M, 0.6 mL) for 12 h.
[b]Yield was determined by ^{19}F NMR spectroscropy using $PhCF_3$ as an internal standard.

Table S2. Photocatalysts screening

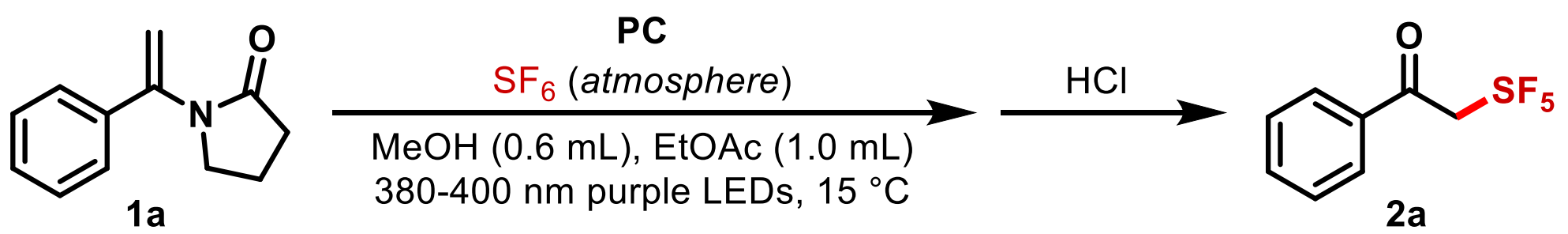

Entry[a]	PC	yield[b]
1	4-CZIPN	N. D.
2	$Ir(ppy)_3$	N. R.
3	Ir(*p*-CF_3-ppy)$_3$	N. D.
4	di-*p*-tolylmethanone	N. D.
5	$Ru(phen)_3(PF_6)_2$	N. D.
6	PTZ	45%

[a]Unless otherwise noted ,the reaction conditions were as follows: **1** (0.1 mmol, 1 equiv), SF_6 (atmosphere charged at -78 °C), **PC**, MeOH (0.6 mL), EtOAc (1.0 mL), 380-400 nm purple LEDs (24 W), 15 °C, 28 h, then HCl (12 M, 0.6 mL) for 12 h.
[b]Yield was determined by ^{19}F NMR spectroscropy using $PhCF_3$ as an internal standard.

Table S3. LEDs screening

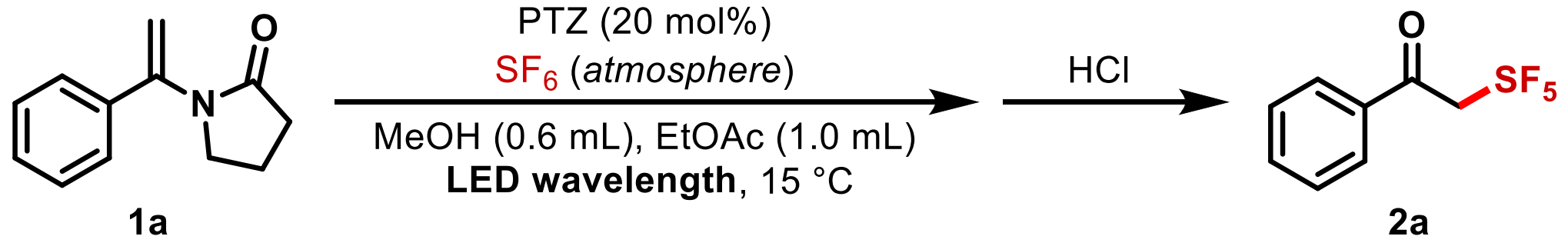

Entry[a]	LED wavelength	yield[b]
1	365 nm	N. D.
2	375 nm	N. D.
3	380-400 nm	45%
4	420 nm	16%
5	440 nm	trace

[a]Unless otherwise noted ,the reaction conditions were as follows: **1a** (0.1 mmol, 1 equiv), SF_6 (atmosphere, charged at -78 °C), PTZ (20 mol%), MeOH (0.6 mL), EtOAc (1.0 mL), LEDs (24 W), 15 °C, 28 h, then HCl (12 M, 0.6 mL) for 12 h.
[b]Yield was determined by ^{19}F NMR spectroscropy using $PhCF_3$ as an internal standard.

Table S4. Temperature screening

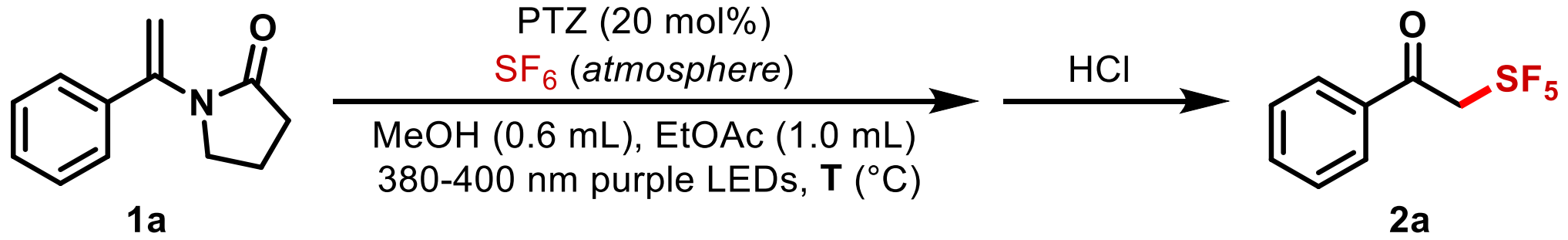

Entry[a]	T	yield[b]
1	0	43%
2	15	45%
3	30	24%
4	50	N. D.

[a]Unless otherwise noted ,the reaction conditions were as follows: **1a** (0.1 mmol, 1 equiv), SF_6 (atmosphere, charged at -78 °C), PTZ (20 mol%), MeOH (0.6 mL), EtOAc (1.0 mL), 380-400 nm purple LEDs (24 W), **T** °C, 28 h, then HCl (12 M, 0.6 mL) for 12 h.
[b]Yield was determined by ^{19}F NMR spectroscropy using $PhCF_3$ as an internal standard.

Table S5. Additives screening

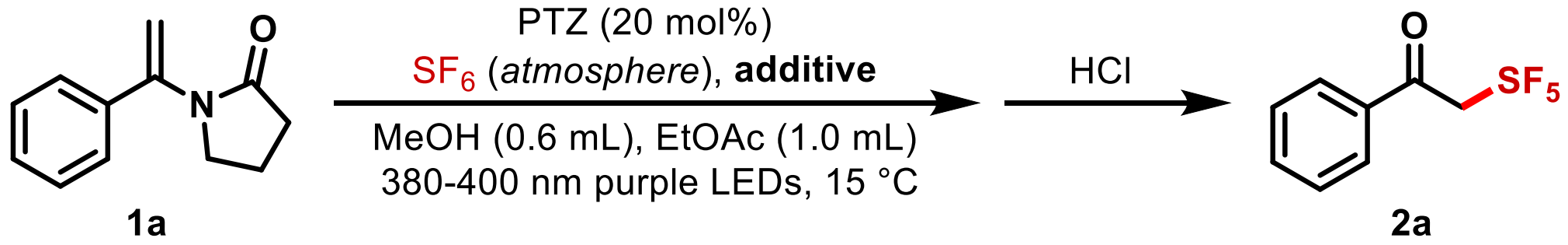

Entry[a]	additive	yield[b]
1	TBAB	trace
2[c]	TBAC	55%
3[d]	TBAC	68%(65%)[e]
4	K_2CO_3	38%
5	$NaHCO_3$	trace

[a]Unless otherwise noted ,the reaction conditions were as follows: **1a** (0.1 mmol, 1 equiv), SF_6 (atmosphere, charged at -78 °C), PTZ (20 mol%), MeOH (0.6 mL), EtOAc (1.0 mL), **additive**, 380-400 nm LEDs (24 W), 15 °C, 28 h, then HCl (12 M, 0.6 mL) for 12 h.
[b]Yield was determined by ^{19}F NMR spectroscropy using $PhCF_3$ as an internal standard.
[c]0.5 equiv TBAC was used.
[d]0.7 equiv TBAC was used.
[e]Isolated yield.

Condition optimizations for vinyl ethers

Table S6. Solvents screening

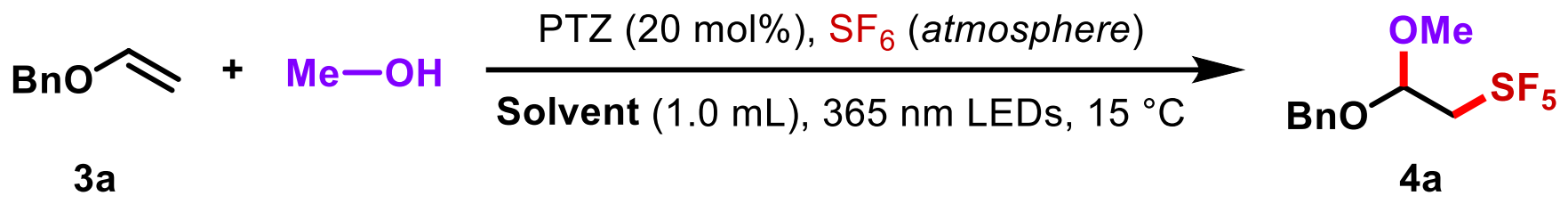

Entry[a]	solvent	yield[b]
1	MeCN	51%
2	MeOH	58%
3	THF	60%
4	dioxane	58%
5	DMF	3%
6	toluene	46%
7	$^{i}Pr_2O$	65%

[a]Unless otherwise noted ,the reaction conditions were as follows: **3a** (0.2 mmol, 1 equiv), SF_6 (atmosphere, charged at -78 °C), PTZ (20 mol%), MeOH (0.8 mL), **Solvent** (1.0 mL), 365 nm LEDs (24 W), 15 °C, 12 h.
[b]Yield was determined by ^{19}F NMR spectroscropy using $PhCF_3$ as an internal standard.

Table S7. The equivalent of MeOH screening

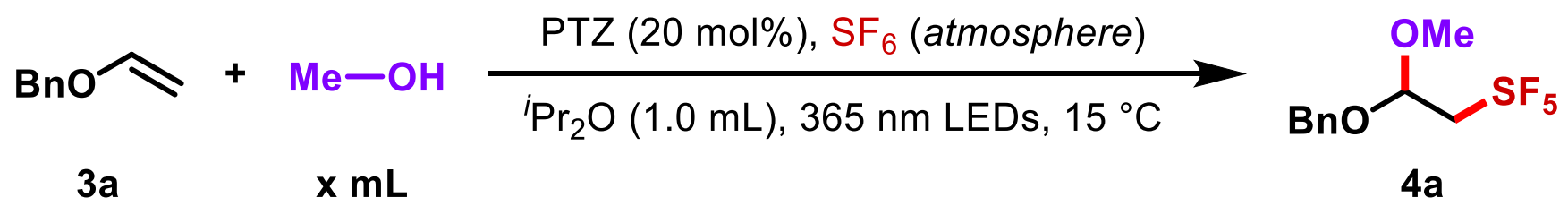

Entry[a]	x	yield[b]
1	0.2	26%
2	0.4	51%
3	0.6	62%
4	0.8	65%
5	1.0	65%
6	1.2	66%

[a]Unless otherwise noted ,the reaction conditions were as follows: **3a** (0.2 mmol, 1 equiv), SF_6 (atmosphere, charged at -78 °C), PTZ (20 mol%), **MeOH (x mL)**, $^{i}Pr_2O$ (1.0 mL), 365 nm LEDs (24 W), 15 °C, 12 h.
[b]Yield was determined by ^{19}F NMR spectroscropy using $PhCF_3$ as an internal standard.

Table S8. LEDs screening

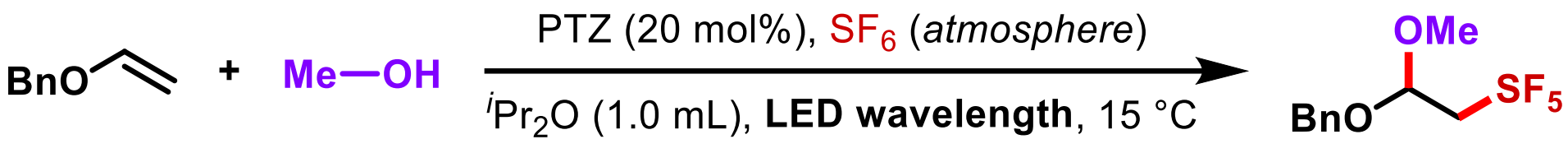

3a 4a

Entry[a]	LED wavelength	yield[b]
1	365 nm	65%
2	390 nm	34%
3	400 nm	14%
4	420 nm	6%
5	440 nm	4%

[a]Unless otherwise noted ,the reaction conditions were as follows: **3a** (0.2 mmol, 1 equiv), SF_6 (atmosphere, charged at -78 °C), PTZ (20 mol%), MeOH (0.8 mL), $^{i}Pr_2O$ (1.0 mL), **LEDs** (24 W), 15 °C, 12 h.
[b]Yield was determined by ^{19}F NMR spectroscropy using $PhCF_3$ as an internal standard.

Table S9. Temperature screening

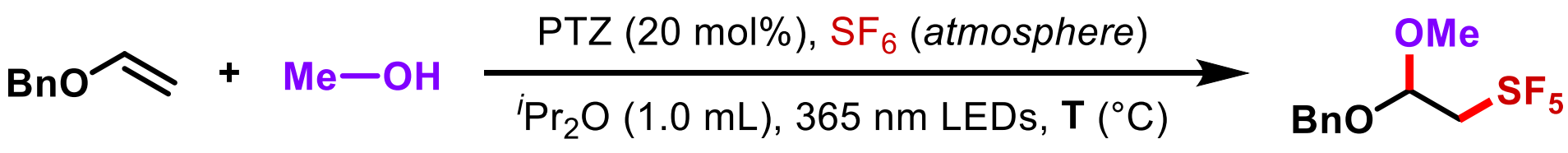

3a 4a

Entry[a]	T	yield[b]
1	0	65%
2	10	65%
3	15	65%(45%)[c]
4	35	31%
5	50	2%

[a]Unless otherwise noted ,the reaction conditions were as follows: **3a** (0.2 mmol, 1 equiv), SF_6 (atmosphere, charged at -78 °C), PTZ (20 mol%), MeOH (0.8 mL), $^{i}Pr_2O$ (1.0 mL), 365 nm LEDs (24 W), **T** °C, 12 h.
[b]Yield was determined by ^{19}F NMR spectroscropy using $PhCF_3$ as an internal standard.
[c]Isolated yield.

Condition optimizations for pentafluorosulfanylated bicyclo[1.1.1]pentane motifs

Table S10. Photocatalysts screening

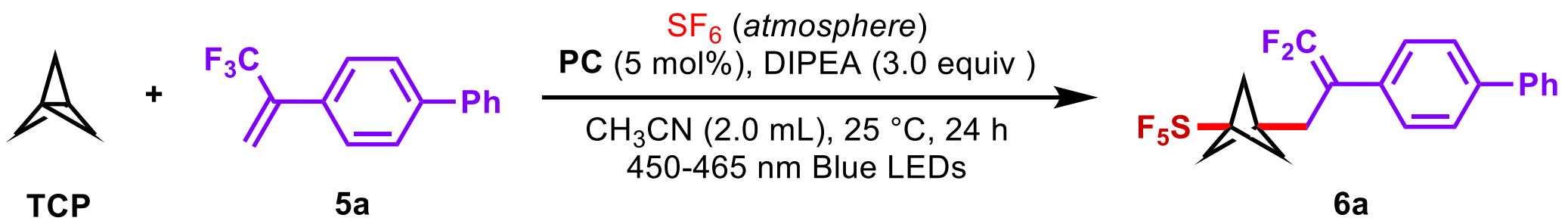

Entry[a]	PC	yield[b]
1	Acid Red 94	N. D.
2	Eosin Y	N. D.
3	$Ir(ppy)_3$	N. D.
4	$\{Ir[dF(CF_3)ppy]_2(dtbpy)\}(PF_6)$	22%
5	$[Ir(dtbbpy)(ppy)_2](PF_6)$	20%
6	$[Ru(4,7\text{-}dPh\text{-}1,10\text{-}phen)_3](PF_6)$	16%
7	$Ru(bpy)_3Cl_2{\bullet}6H_2O$	17%
8	4CzIPN	16%

[a]Unless otherwise noted ,the reaction conditions were as follows: **5a** (0.3 mmol, 3 equiv), **TCP** (0.1 mmol, 1 equiv), SF_6 (atmosphere), **PC** (5 mol%), CH_3CN (2.0 mL), 450-465 nm Blue LEDs (40 W), 25 °C, 24 h.
[b]Yield was determined by ^{1}H NMR spectroscropy using PhOMe as an internal standard.

Table S11. Solvents screening

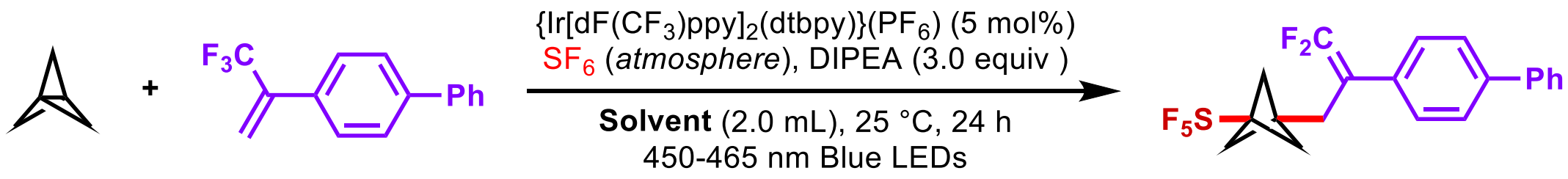

Entry[a]	Solvent	yield[b]
1	DCE	20%
2	DMF	18%
3	CH_3CN	22%
4	THF	21%
5	EA	29%
6	PhCl	11%
7	Acetone	53%
8	^{i}PrOH	15%

[a]Unless otherwise noted ,the reaction conditions were as follows: **5a** (0.3 mmol, 3 equiv), **TCP** (0.1 mmol, 1 equiv), SF_6 (atmosphere), $\{Ir[dF(CF_3)ppy]_2(dtbpy)\}(PF_6)$ (5 mol%), **Solvent** (2.0 mL), 450-465 nm Blue LEDs (40 W), 25 °C , 24 h.
[b]Yield was determined by ^{1}H NMR spectroscropy using PhOMe as an internal standard.

Table S12. Reductants screening

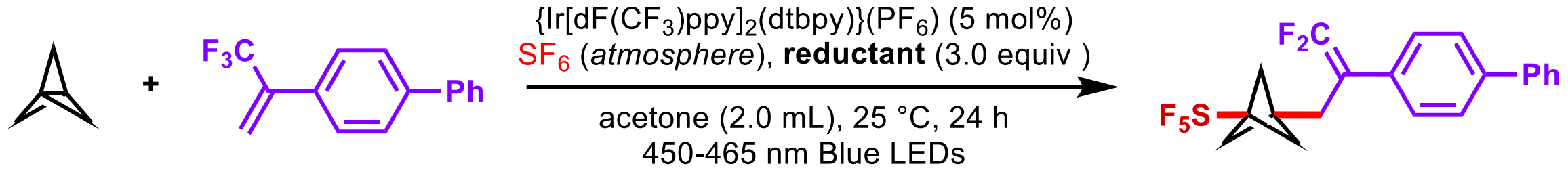

TCP 5a 6a

Entry[a]	reducant	yield[b]
1	DIPEA	53%
2	TMEDA	N. D.
3	Et_3N	34%
4	Cy_2NMe	23%
5	DIPA	16%
6	BnSH	N. D.
7	HEH	N. D.
8	$(TMS)_3SiH$	N. D.

[a]Unless otherwise noted ,the reaction conditions were as follows: **5a** (0.3 mmol, 3 equiv), **TCP** (0.1 mmol, 1 equiv), SF_6 (atmosphere), {Ir[dF(CF_3)ppy]$_2$(dtbpy)}(PF_6) (5 mol%), acetone (2.0 mL), **reducant** (0.3 mmol, 3 equiv), 450-465 nm Blue LEDs (40 W), 25 °C, 24 h.
[b]Yield was determined by ^{1}H NMR spectroscropy using PhOMe as an internal standard.

Table S13. LEDs Screening

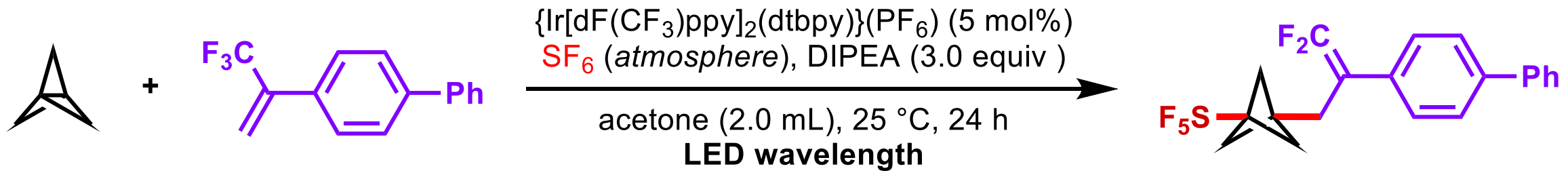

TCP 5a 6a

Entry[a]	LED wavelength	yield[b]
1	365	29%
2	390	31%
3	410	30%
4	430	39%
5	450	42%
6	460	53%(51%)[c]
7	480	39%

[a]Unless otherwise noted ,the reaction conditions were as follows: **5a** (0.3 mmol, 3 equiv), **TCP** (0.1 mmol, 1 equiv), SF_6 (atmosphere), {Ir[dF(CF_3)ppy]$_2$(dtbpy)}(PF_6) (5 mol%), acetone (2.0 mL), DIPEA (0.3 mmol, 3 equiv), **LEDs** (40 W), 25 °C, 24 h.
[b]Yield was determined by ^{1}H NMR spectroscropy using PhOMe as an internal standard.
[c]Isolated yield.

Substrates synthesis

General procedures for synthesizing enamines

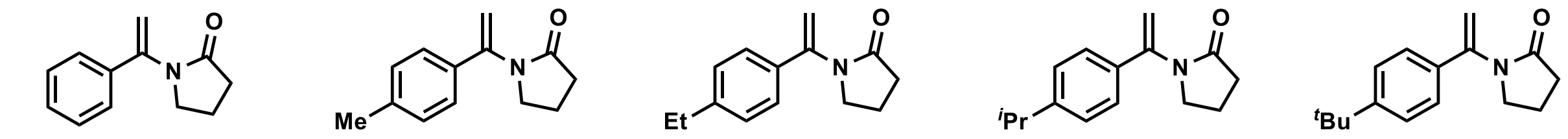

1a **1b** **1c** **1d** **1e**

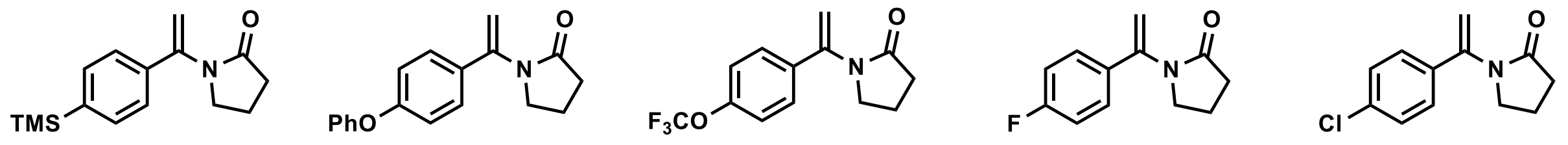

1f **1g** **1h** **1i** **1j**

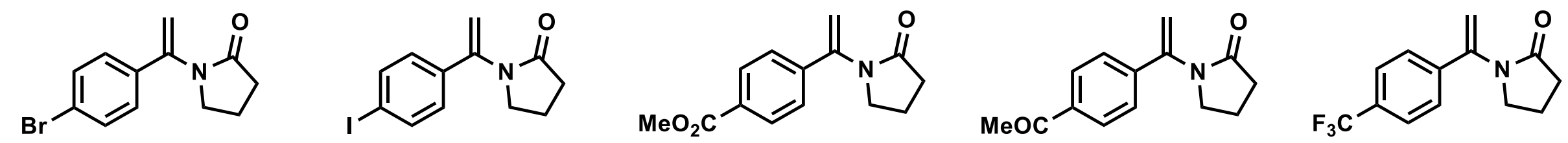

1k **1l** **1m** **1n** **1o**

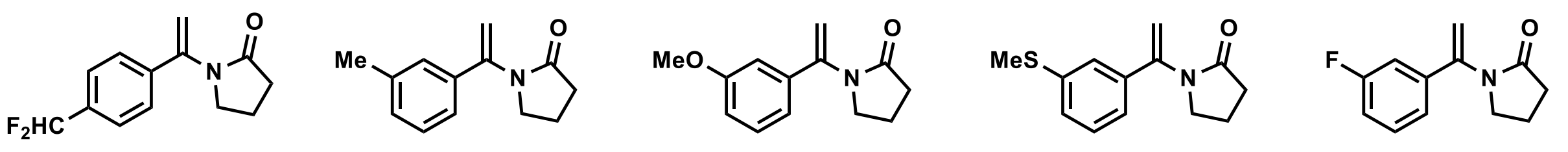

1p **1q** **1r** **1s** **1t**

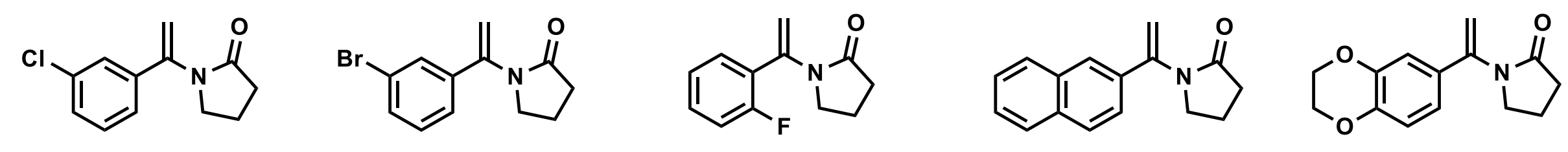

1u **1v** **1w** **1x** **1y**

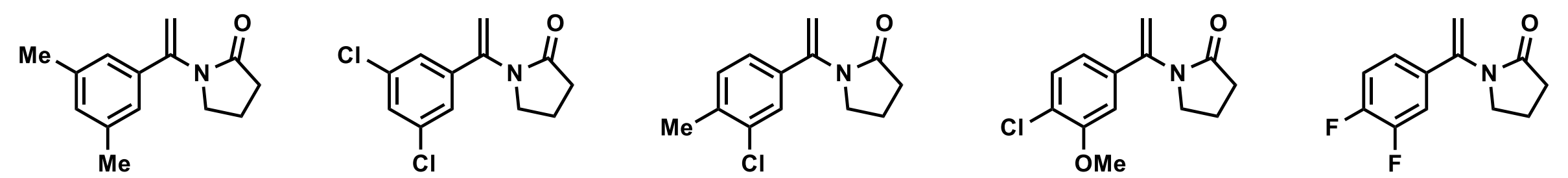

1z **1aa** **1ab** **1ac** **1ad**

General procedure A for the synthesis of substrates 1a-1ad:

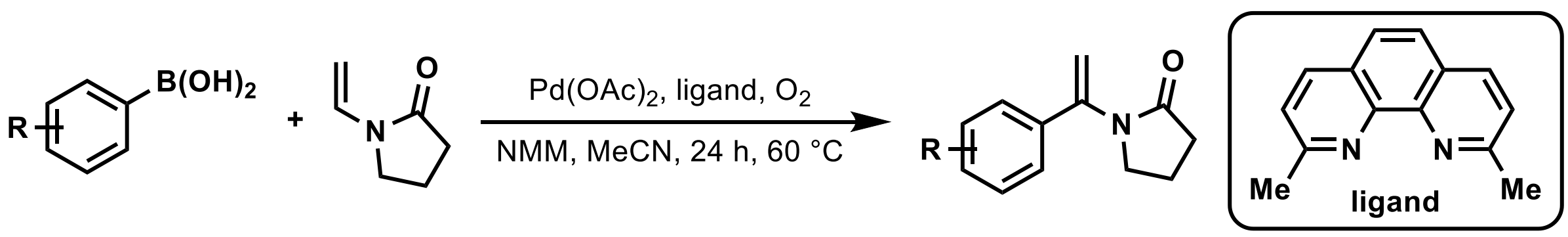

A 100 mL round-bottomed flask, fitted with a glass tube as cooler was charged with boronic acid (10 mmol), enamide (5 mmol), N-methylmorpholine (1.1 mL, 10 mmol) and acetonitrile (15 mL). The reaction mixture was stirred until all the reagents were thoroughly dissolved. $Pd(OAc)_2$ (0.2 mmol) and ligand (0.24 mmol) dissolved in 10 mL acetonitrile were then added

to the reaction mixture. The mixture was vigorously stirred with O_2 balloon at 60 °C for 24 h. Then, the mixture was filtrated through diatomite and the filtrate was evaporated under vacuum and the residue was purified by flash column chromatography on silica gel to give the desired product **1a-1ad.** (*J. Org. Chem.* **2004**, *69*, 5212–5218)

General procedures for synthesizing vinyl ethers

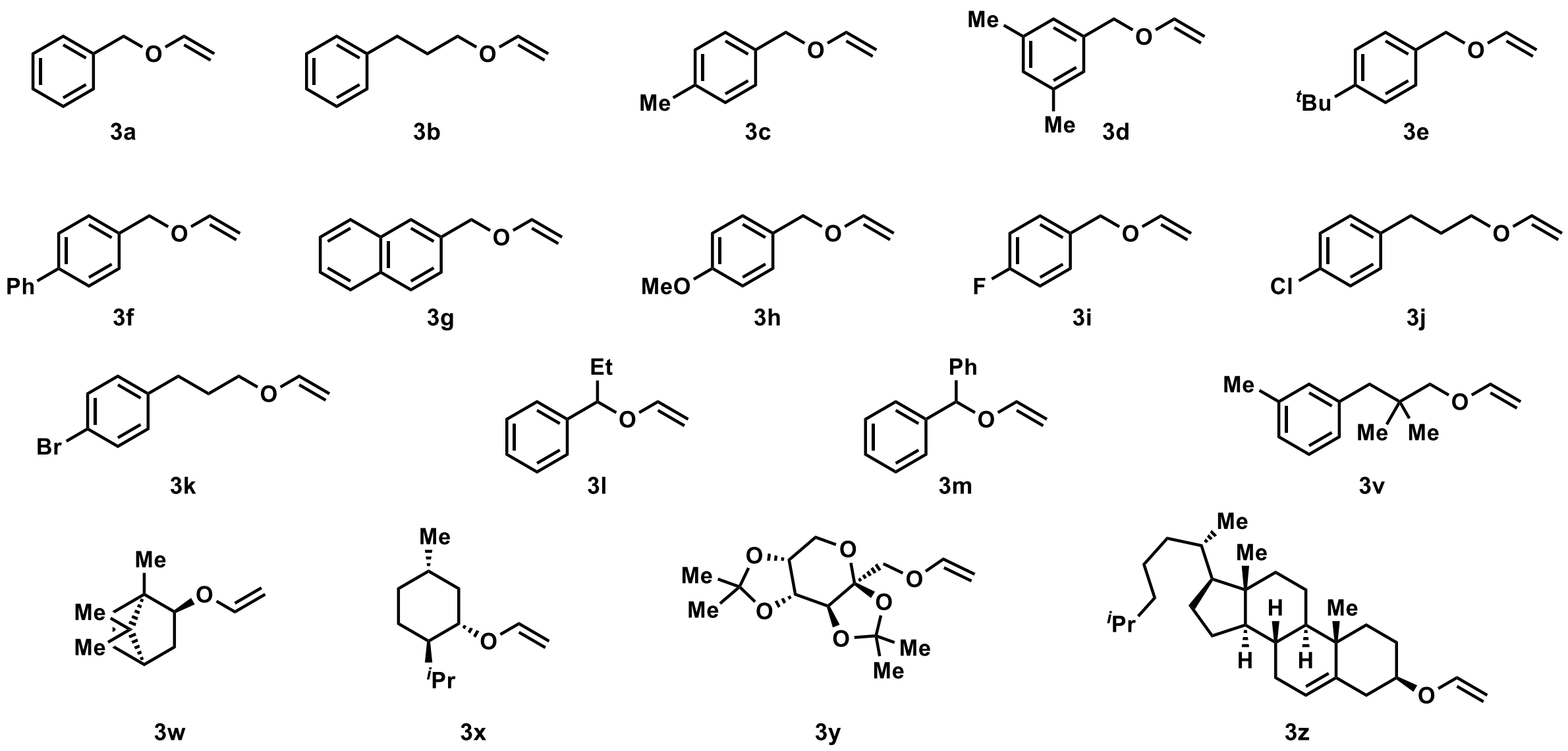

General procedure B for the synthesis of substrates 3a-3v:

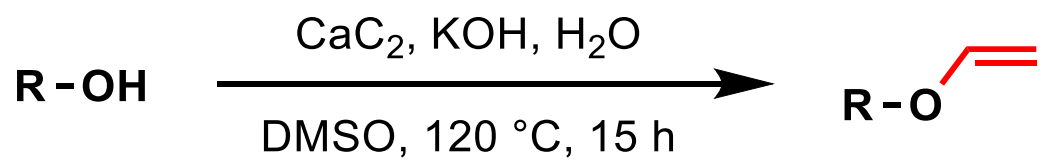

In a 200 mL two-necked round-bottom flask with a reflux con denser, a mixture of alcohol (26 mmol), KOH (1.1 g, 20 mmol), and water (2.2 g, 120 mmol) in DMSO (40 mL) was prepared. After stirring at room temperature for 30 min, calcium carbide (5.2 g, 81.4 mmol) was added, the flask was then sealed with a rubber septum and attached to a balloon to avoid excess pressure due to generated acetylene. The mixture was gradually heated to 120 °C under vigorous stirring for 15 h. Then, the mixture was filtered through Aluminum oxide and the mixture was extracted with ethyl acetate. The reaction mixture was evaporated under vacuum and the residue was purified by flash column chromatography on silica gel with pentane to give the desired product **3a-3v**. (*Green Chem.*, **2016**, *18*, 2614–2618)

General procedure C for the synthesis of substrates 3v-3z:

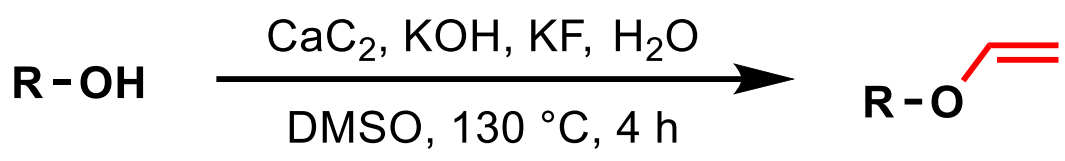

KOH (20 mmol, 1.1 g), alcohol (10 mmol), KF (40 mmol, 2.3 g) and freshly powdered calcium carbide (20 mmol, 1.3 g) were added to a reaction tube with 20 mL of DMSO. After stirring the mixture at room temp erature for 5 min, water (40.0 mmol, 720 μL) was added. The tube was sealed, and the mixture was gradually heated at 130 °C for 4 h with vigorous stirring. Then, the mixture was filtered through Aluminum oxide and the mixture was extracted with extracted with ethyl acetate, the reaction mixture was evaporated under vacuum and the residue was purified by flash column chromatography on silica gel with pentane to give the desired product **3v-3z**. (*Green Chem.,* **2017**, *19*, 3032–3041)

General procedures for synthesizing trifluoropropylene derivatives.

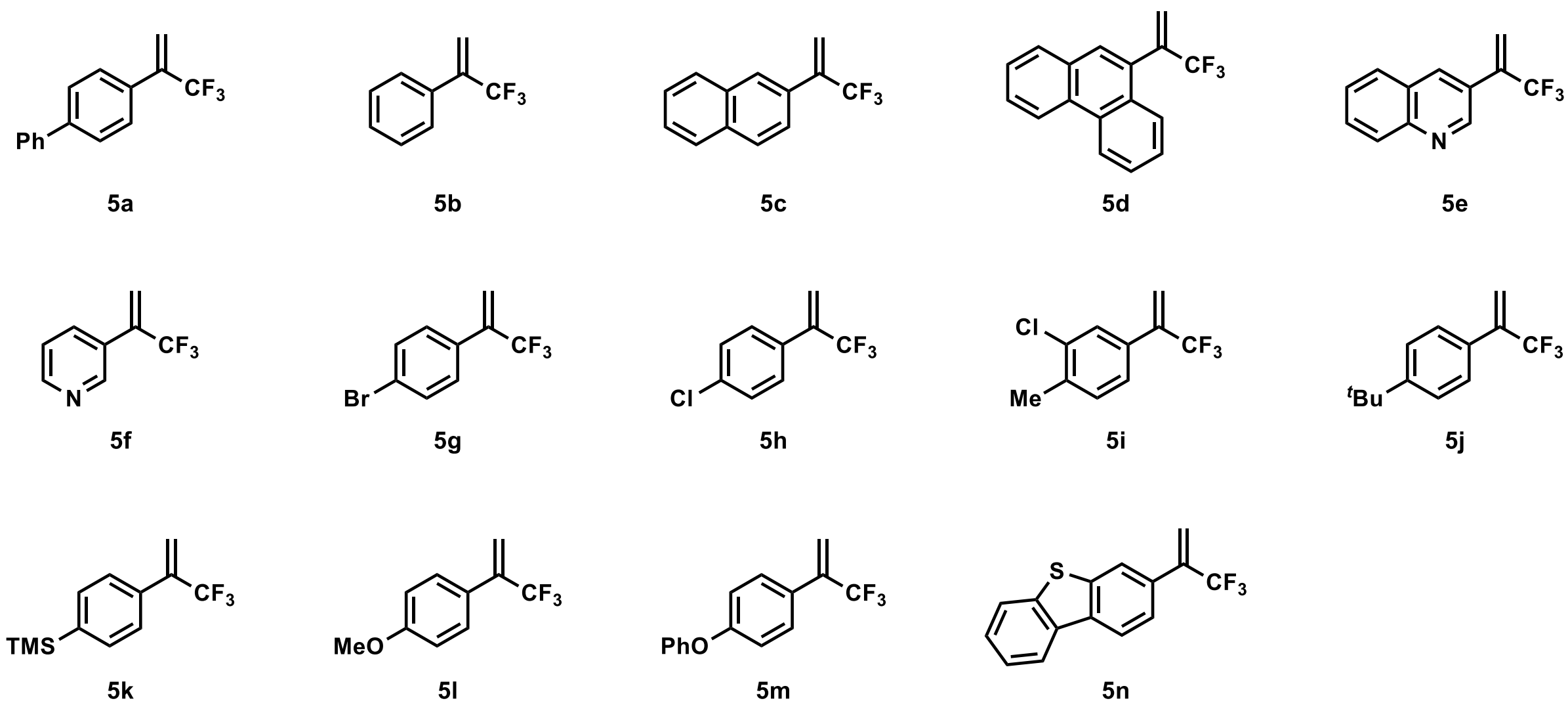

General procedure D of synthesis of the substrates 5a-5n

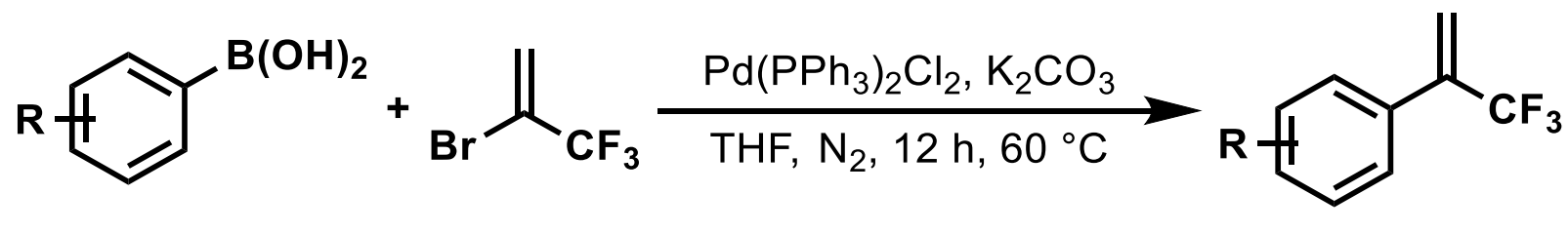

To a Schlenk tube equipped with stir bar, arylboronic acid (1.0 equiv., 10 mmol) and $Pd(PPh_3)_2Cl_2$ (3 mol%, 0.3 mmol, 210.6 mg) were added. The vessel was evacuated and filled with argon (three times), and then aqueous K_2CO_3 (2.0 M, 20 mL) and THF (30 mL) were added. After addition of 2-bromo-3,3,3-trifluoropropene (2.0 equiv., 20 mmol, 2.1 mL), the

solution was stirred at 60 °C for 12 hours (TLC tracking detection). The solvent was removed under reduced pressure and the residue was purified by column chromatography to afford the corresponding trifluoromethyl alkene (PE - PE/EA=100:1). (*Org. Lett.* **2019**, *21*, 2658−2662)

General procedure E for the synthesis of tricyclo[1.1.1.0^{1,3}]pentane (TCP):

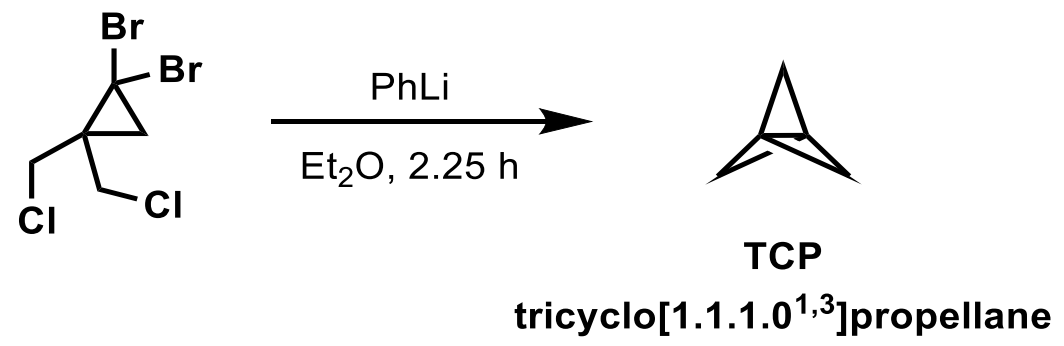

1,1-Dibromo-2,2-bis(chloromethyl)cyclopropane (5.94 g, 20 mmol, 1 equiv.) was added to a heat-gun dried 100 mL two-neck round bottom flask equipped with a stir bar. The flask was evacuated and backfilled with N_2. Next, 20 mL of anhydrous Et_2O was added via syringe. The solution was cooled to –78 °C. Phenyllithium (PhLi) (1.5 M in *Dibutyl Ether* 27 mL, 40 mmol, 2 equiv.) was added dropwise. Upon addition of PhLi, the reaction mixture turned brown, and a precipitate formed. The reaction was allowed to stir at –78 °C for 20 min and was then transferred to an ice bath and stirred at 0 °C for 2 h. After 2 h, a short path condenser connected to a 100 mL 2-neck receiver flask was introduced. The system was placed under vacuum for 2 min, then static vacuum was maintained. (Care should be taken not to pull the crude solution over through the distillation head, vigorous bubbling was observed.) Vacuum may be reapplied periodically to increase the rate of the distillation. After the distillation is complete, the resulting solution of tricyclo[1.1.1.0^{1,3}]pentane (TCP) was transferred via cannula to a Teflon-capped 25 mL Schlenk tube. (*Sci. China Chem*. **2023**, *66*, 2871–2877)

General procedure for the pentafluorosulfanylation reactions

General procedure F for the photocatalytic pentafluorosulfanylation of enamines with SF_6 (2a-2ad):

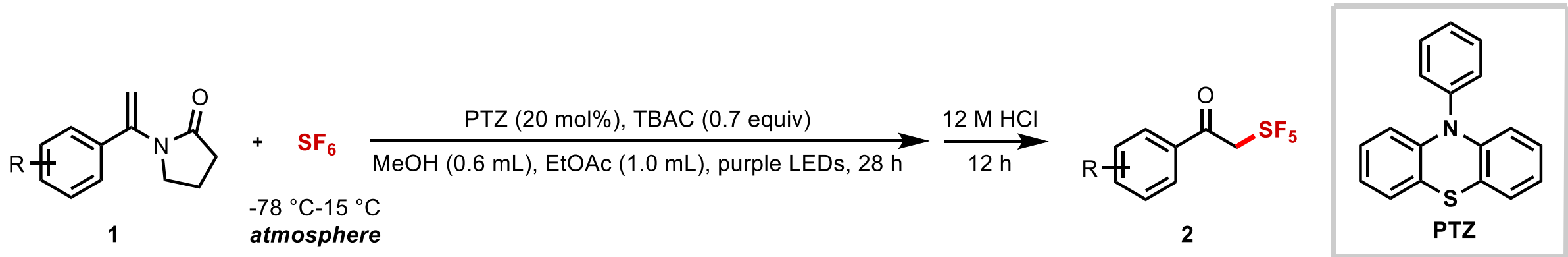

In a 10 mL oven-dried Schlenk tube, PTZ (20 mol%) was added, the tube was evacuated and backfilled with N_2 (repeated for 3 times), quickly added TBAC (0.07 mmol, 0.7 equiv). Then, the reaction mixture was frozen to -78 °C. After that, the tube was evacuated and backfilled with SF_6 (repeated for 3 times) while enamines **1** (0.1 mmol, 1.0 equiv), dry EtOAc (1.0 mL) and dry MeOH (0.6 mL) were added quickly at -78 °C. After completion, the reaction was moved out, waiting for the sublimation of SF_6, and irradiated by 380-400 nm purple LEDs (24 W) at 15 °C for 28 h. The reaction tube should be opened carefully until the bubbles disappear. After that, HCl (12 M, 0.6 mL) was added slowly and stirred continuously for 12 h. The products **2a**-**2ad** were purified by column chromatography with dichloromethane in petroleum ether as eluent.

General procedure G for the photocatalytic pentafluorosulfanylation of vinyl ethers with SF_6 (4a-4z):

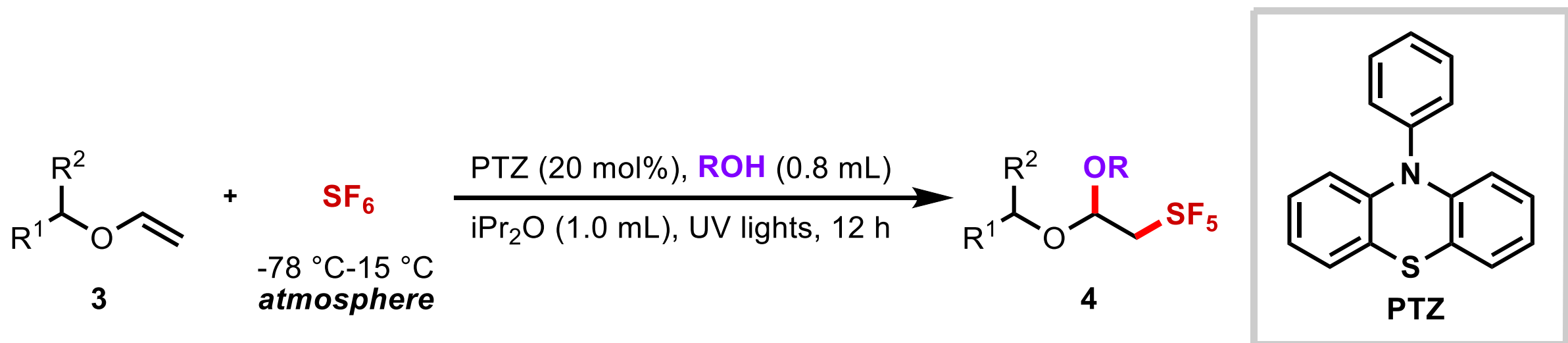

In a 10 mL oven-dried Schlenk tube, PTZ (20 mol%) was added, the tube was evacuated and backfilled with N_2 (repeated for 3 times). Then, the reaction mixture was frozen to -78 °C. After that, the tube was evacuated and backfilled with SF_6 (repeated for 3 times) while vinyl

ethers **3** (0.2 mmol, 1.0 equiv), dry $^{i}Pr_2O$ (1.0 mL) and dry MeOH (0.8 mL) were added quickly at -78 °C. After completion, the reaction was moved out, waiting for the sublimation of SF_6, and irradiated by 365 nm UV lights (24 W) at 15 ℃ for 12 h. The reaction tube should be opened carefully until the bubbles disappear. The products **4a**-**4z** were purified by column chromatography with dichloromethane in hexane as eluent.

General procedure H for the direct construction of pentafluorosulfanylated bicyclo[1.1.1]pentane motifs with SF_6 (6a-6n):

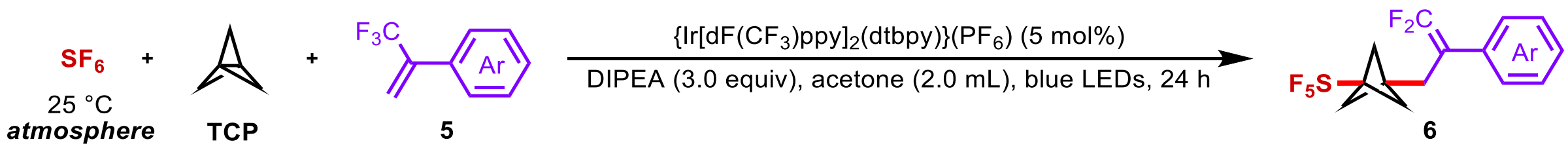

In a 10 mL oven-dried Schlenk tube, {Ir[dF(CF_3)ppy]$_2$(dtbpy)}(PF_6) (5 mol%) was added. The tube was evacuated and backfilled with SF_6 (repeated for 3 times), while 2-aryl trifluoropropenes **5** (0.3 mmol, 3.0 equiv), DIPEA (0.3 mmol, 3.0 equiv), acetone (2.0 mL) and TCP (1.0 M in Et_2O, 0.1 mmol, 1.0 equiv) were added quickly under SF_6 atmosphere. The reaction was irradiated by 450-465 nm blue LEDs (40 W) at 25 ℃ for 24 h. After completion, the reaction mixture was extracted with ethyl acetate, water and brine, dried over Na_2SO_4, filtrated and concentrated in vacuo. The products **6a**-**6n** were purified with HPLC Thermo Scientific UltiMate 3000 (Shimadzu Shim-pack PRC-ODS).

Synthetic applications

Preparation of (2-(3-(4-chlorophenyl)propoxy)pent-4-en-1-yl)pentafluoro-λ^6-sulfane

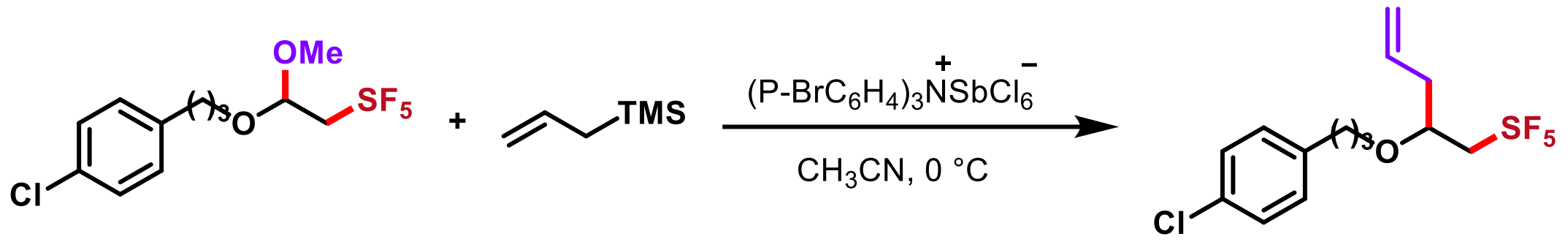

Modified based on a literature report Tetrahedron Letters, Volume 37, Issue 20, 1996, Pages 3483-3486, (DOI: 10.1016/0040-4039(96)00596-5), when tris(p-bromophenyl)aminium hexachloroantimonate (0.02 mmol) was added to a dry acetonitrile solution containing SF_5 products (0.2 mmol) and allyltrimethylsilane (0.2 mmol) at 0 °C under argon atmosphere, after one hour of reaction, tris(p-bromophenyl)aminium hexachloroantimonate (0.02 mmol) and allyltrimethylsilane (0.2 mmol) was added again, with a total of 6 times, then the reaction proceeded for 12 hours. After completion, the crude mixture was purified by column chromatography on silica gel to give the corresponding product with 44% yield.

(2-(3-(4-chlorophenyl)propoxy)pent-4-en-1-yl)pentafluoro-λ^6-sulfane

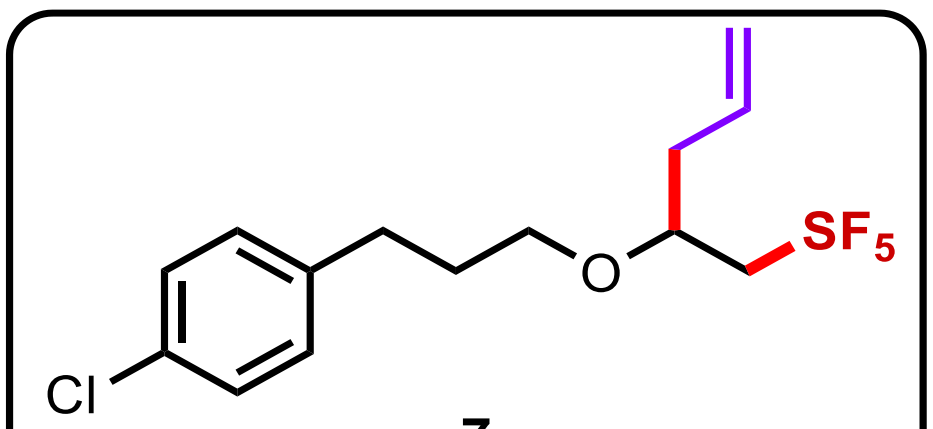

7

^{1}H NMR (500 MHz, $CDCl_3$) δ 7.30 – 7.21 (m, 2H), 7.17 – 7.05 (m, 2H), 5.86 – 5.60 (m, 1H), 5.23 – 5.02 (m, 2H), 3.91 (tt, J = 7.4, 4.1 Hz, 1H), 3.78 – 3.54 (m, 3H), 3.47 – 3.33 (m, 1H), 2.75 – 2.56 (m, 2H), 2.45 – 2.25 (m, 2H), 1.95 – 1.80 (m, 2H).

^{13}C NMR (126 MHz, $CDCl_3$) δ 140.43, 132.63, 131.65, 129.93, 128.56, 119.25, 75.03 (p, J = 4.0 Hz), 74.23 (p, J = 11.4 Hz), 68.52, 37.64, 31.75, 31.56.

^{19}F NMR (471 MHz, $CDCl_3$) δ 86.06 – 84.20 (m, 1F), 66.53 (dt, J = 146.4, 8.5 Hz, 4F).

HRMS (ESI) (m/z): $[M+H]^+$ calcd. for $C_{14}H_{19}ClF_5OS$: 365.0760, found: 365.0789.

Preparation of 2-(pentafluoro-λ6-sulfaneyl)acetaldehyde

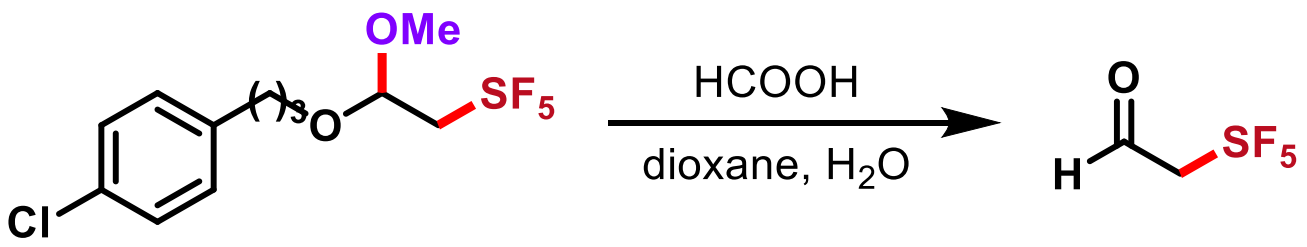

Acetal (0.2 mmol, 1 equiv) was dissolved in 1,4-dioxane (1 mL) and an 80% (w/w) aqueous solution of formic acid (1.1 mL) was added. The reaction mixture was stirred for 12 h at ambient temperature. The reaction was quenched by the addition of water (2 mL). The aqueous layer was extracted with 2 mL DCM and obtain the crude product with 65% ^{19}F NMR yield. All spectroscopic data were in agreement with those reported in the literature. (*Beilstein J. Org. Chem.* **2013,** *9,* 2675–2680.)

^{19}F NMR (376 MHz, $CDCl_3$) δ 81.97 – 79.61 (m, 1F), 73.66 – 72.25 (m, 4F).

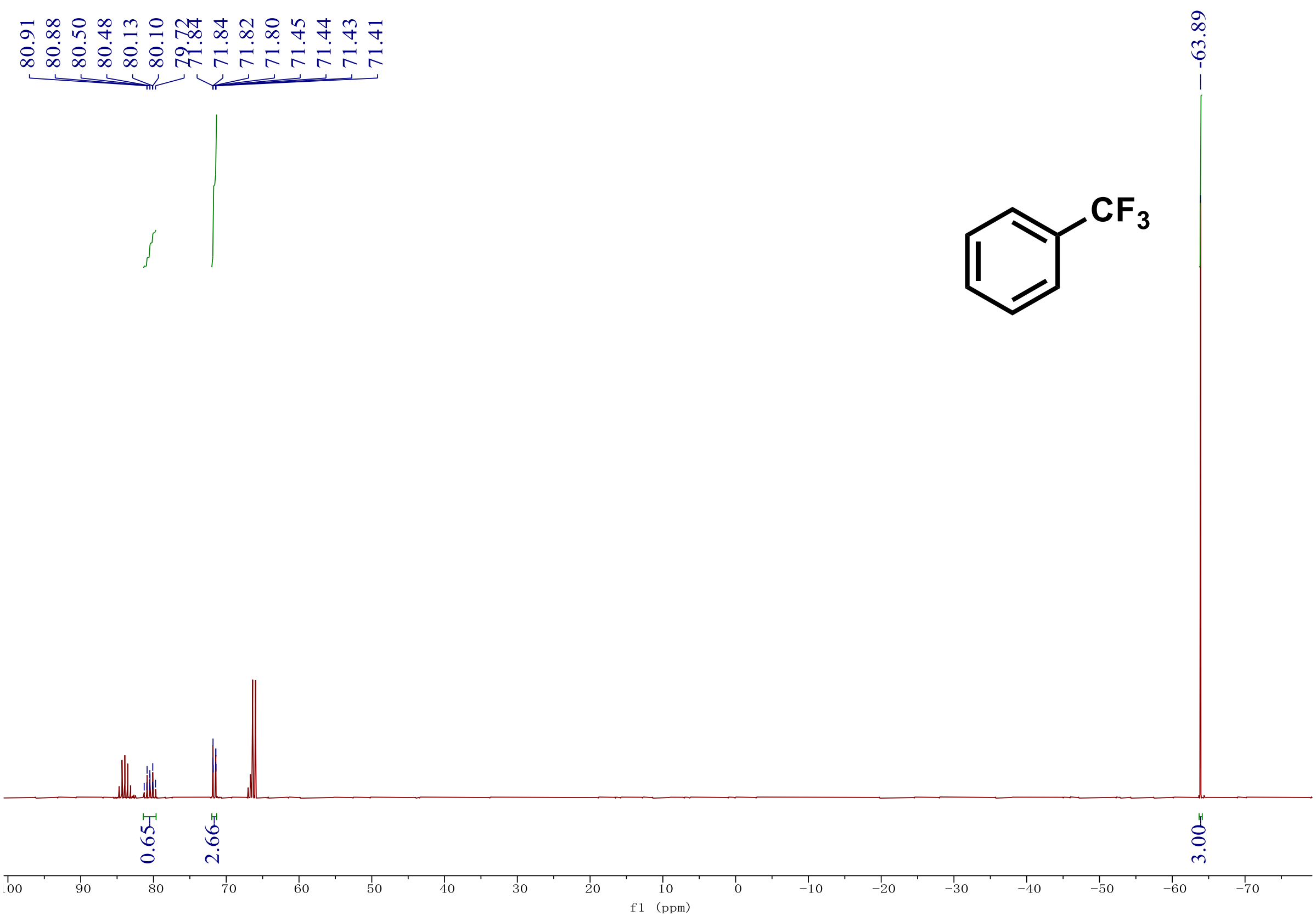

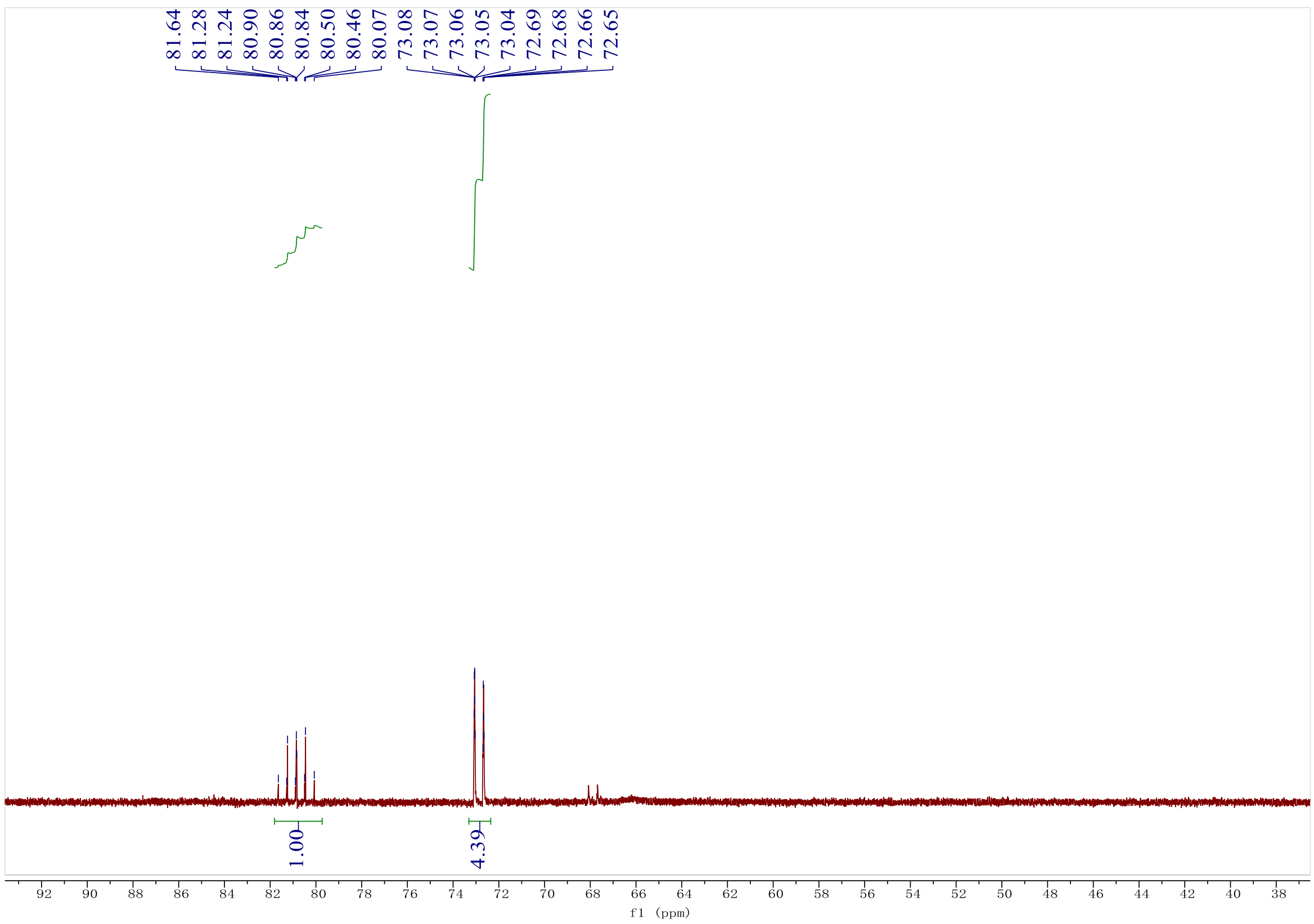

Preparation of 2-(pentafluoro-λ^6-sulfaneyl)-1-phenylethan-1-ol

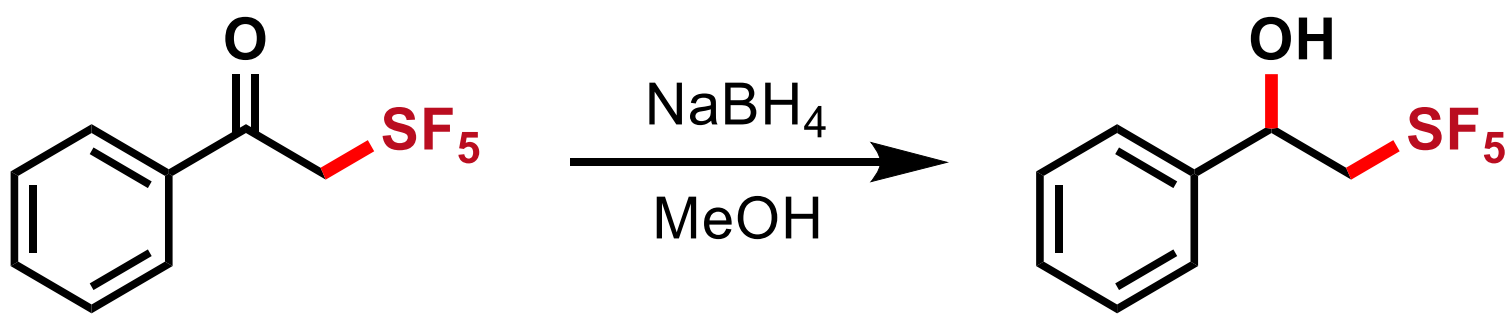

To a solution of **2a** (0.2 mmol) in MeOH (1.0 mL) at 0 °C was slowly added $NaBH_4$ (2 mmol, 10 eq). After 12 h, water was added and the mixture was stirred for 1 h at room temperature. The reaction mixture was extracted with EtOAc, and the combined organic layers were dried with $MgSO_4$ and concentrated under vacuum. The residue was purified by column chromatography to provide compound as white oil.

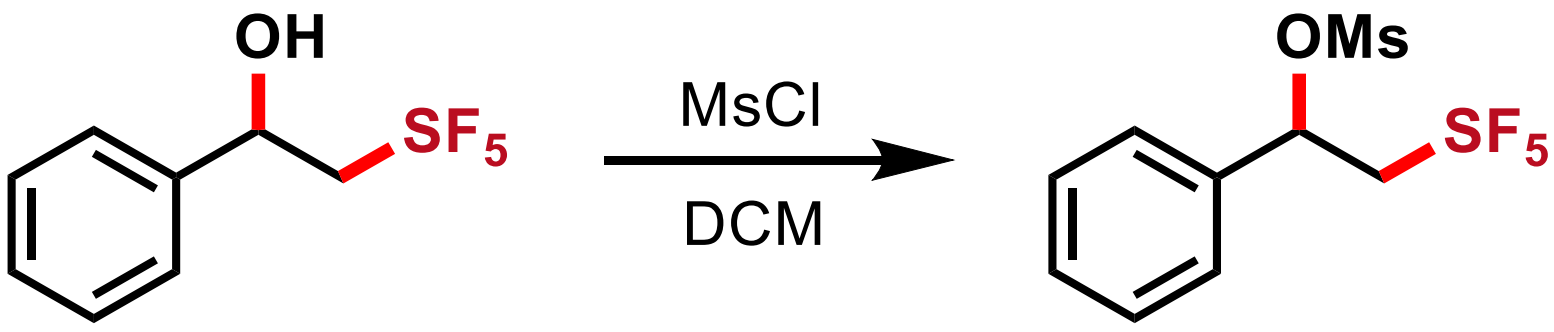

The alcohol (0.15 mmol), NEt_3 (0.23 mmol), and dry CH_2Cl_2 (1.0 mL) were combined in a 10-mL Schlenk tube. The Tube was then flushed with nitrogen and capped. The mixture was stirred for 5 min, and then mesyl chloride (0.18 mmol) was added in one portion by syringe,

the mixture was stirred at 0 °C for 30 min, and then return to r.t. overnight. The compounds were purified by means of column chromatography with 62% yield.

2-(pentafluoro-λ⁶-sulfaneyl)- λ⁶-phenylethyl methanesulfonate

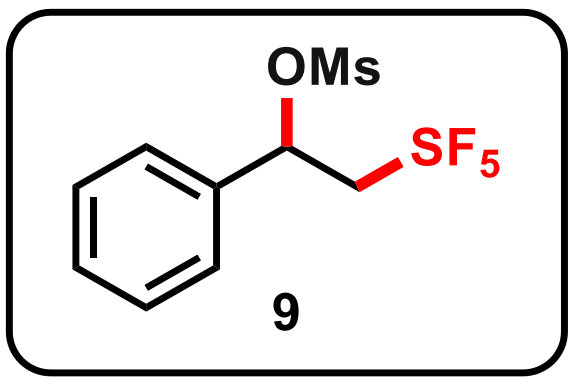

^{1}H NMR (400 MHz, $CDCl_3$) δ 7.51 – 7.38 (m, 5H), 6.09 (dd, J = 9.6, 2.7 Hz, 1H), 4.33 – 4.15 (m, 1H), 3.95 – 3.78 (m, 1H), 2.80 (s, 3H).

^{13}C NMR (101 MHz, $CDCl_3$) δ 135.58, 130.39, 129.66, 126.68, 78.71 (p, J = 4.1 Hz), 74.24 (p, J = 14.1 Hz), 39.60, 29.84.

^{19}F NMR (376 MHz, $CDCl_3$) δ 83.20 – 81.23 (m, 1F), 66.50 (dt, J = 146.6, 7.7 Hz, 4F)).

HRMS (ESI) (*m/z*): $[M+Na]^+$ calcd. for $C_9H_{11}F_5O_3S_2Na$: 348.9962, found: 348.9931.

The conversion of the SF_5 group

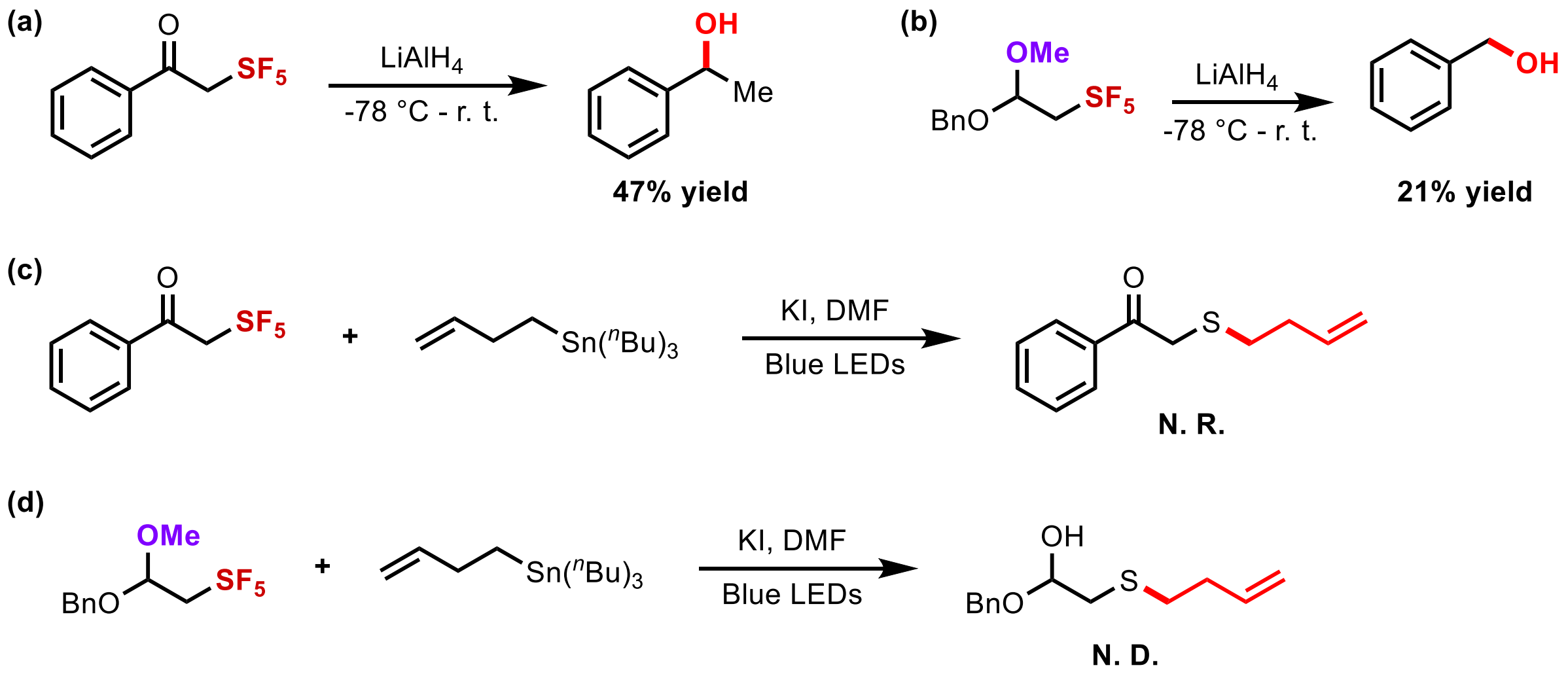

(a) To a solution of **2a** (0.2 mmol) in THF (2.0 mL) at -78 °C was slowly added $LiAlH_4$ (1.0 M in THF) (0.4 mmol, 2 eq). After that, the reaction was proceeded at room temperature overnight. Water was added and the mixture was stirred for 1 h. The reaction mixture was extracted with ethyl acetate, and the combined organic layers were dried with $MgSO_4$ and concentrated under vacuum. The residue was purified by column chromatography to

provide benzyl alcohol with 47% yield. Unfortunately, no fluorine signals were observed with the ^{19}F NMR result.

(b) To a solution of **4a** (0.2 mmol) in THF (2.0 mL) at -78 °C was slowly added $LiAlH_4$ (1.0 M in THF) (0.4 mmol, 2 eq). After that, the reaction was proceeded at room temperature overnight. Water was added and the mixture was stirred for 1 h. The reaction mixture was extracted with ethyl acetate, and the combined organic layers were dried with $MgSO_4$ and concentrated under vacuum. The residue was purified by column chromatography to provide benzyl alcohol with 21% yield. Unfortunately, no fluorine signals were observed with the ^{19}F NMR result.

(c) In a dried test tube, **2a** (0.10 mmol), but-3-en-1-yltributylstannane (0.30 mmol) and KI (0.60 mmol) were mixed in DMF (2 mL) under N_2 atmosphere. The mixture was irradiated with 460 nm blue LED for 24 h. After the irradiation, water was added to the mixture and extracted with ethyl acetate for 3 times. The reaction was monitored by TLC, but the desired product was not obtained.

(d) In a dried test tube, **4a** (0.10 mmol), but-3-en-1-yltributylstannane (0.30 mmol) and KI (0.60 mmol) were mixed in DMF (2 mL) under N_2 atmosphere. The mixture was irradiated with 460 nm blue LED for 24 h. After the irradiation, water was added to the mixture and extracted with ethyl acetate for 3 times. The reaction was monitored by TLC, but the desired product was not obtained.

Mechanism investigations

Light on/off experiment

PTZ (0.2 equiv) was firstly weighted in a 10 mL oven-dried Schlenk tube. The tube was evacuated and backfilled with N_2 (repeated for 3 times). Then, the reaction mixture was frozen to -78 °C. The reaction tube was evacuated and the atmosphere was exchanged against SF_6 while substrates **3a**, dry ${}^{i}Pr_2O$ (1 mL) and dry MeOH (0.8 mL) were added quickly. After completion, the reaction tube was charged with SF_6 for additional 4 mins. The reaction tube was moved to room temperature until SF_6 was resublimed to the gas phase (about 2 mins). The reaction was irradiated at 365 nm, 15 °C with 5 parallel experiments. After 3 h, the light was turned off and tube #1 was opened to measure ^{19}F NMR yield with $PhCF_3$ as the internal standard substance. After 15 mins, tube #2 was subject to yield measurement and the light was turned on for another 3 h, and the rest of experiments were done in the same manner. The results showed obvious suspension of reaction process when light source was removed.

Radical Trapping Experiments

The reactions were proceeded under standard conditions with additional 3.0 equiv of TEMPO. After completion, the products were identified with ^{19}F crude NMR. Corresponding products **2a**, **4a**, **6a** were detected only trace amount. The radical abstract product TEMPO-SF_5 and TEMPO-BCP-SF_5 were found in the three-component reaction by HRMS (ESI) analysis. However, reactions using enamines or vinyl ethers failed to provide the same result, possibly due to the relative low concentration of SF_5 radical.

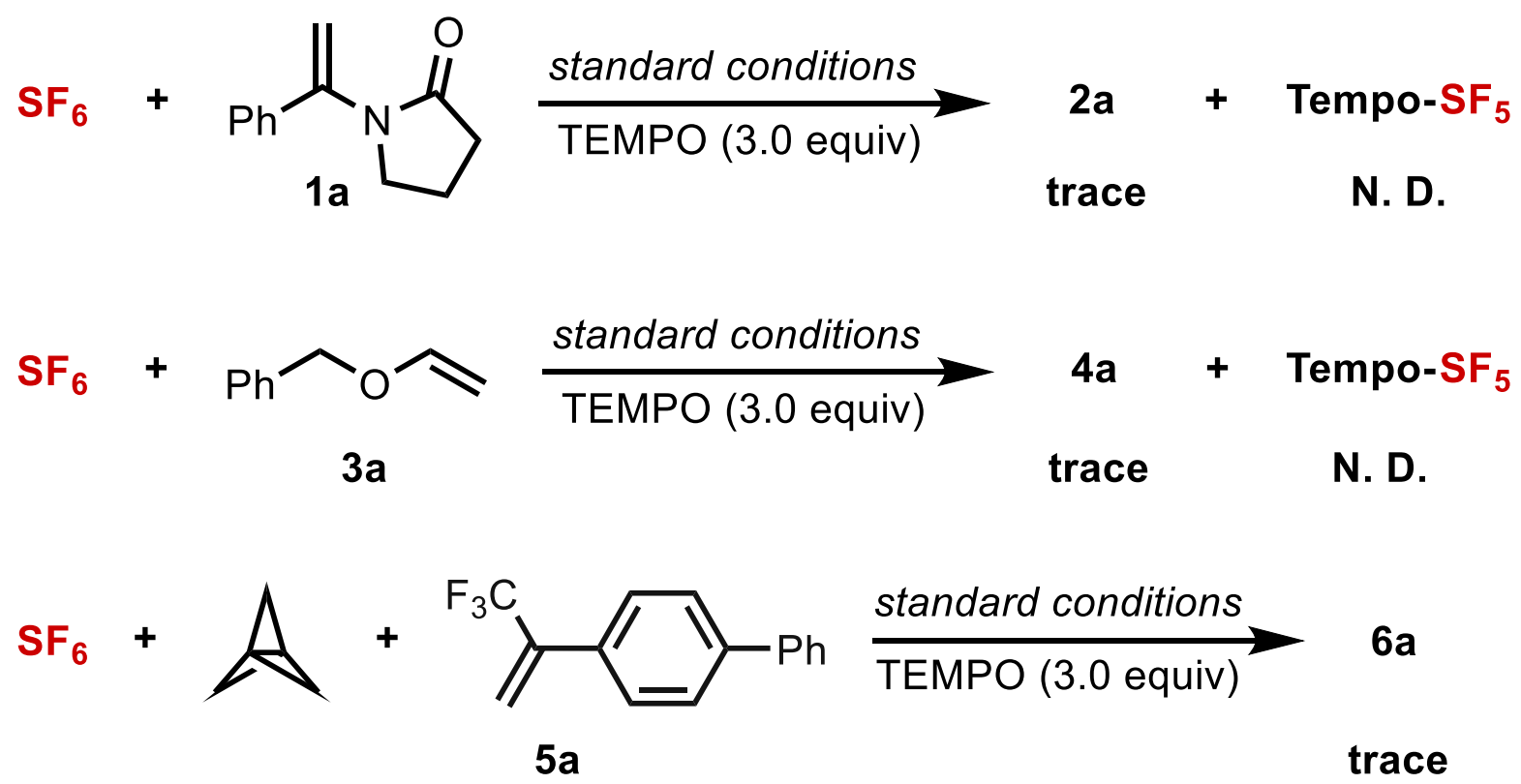

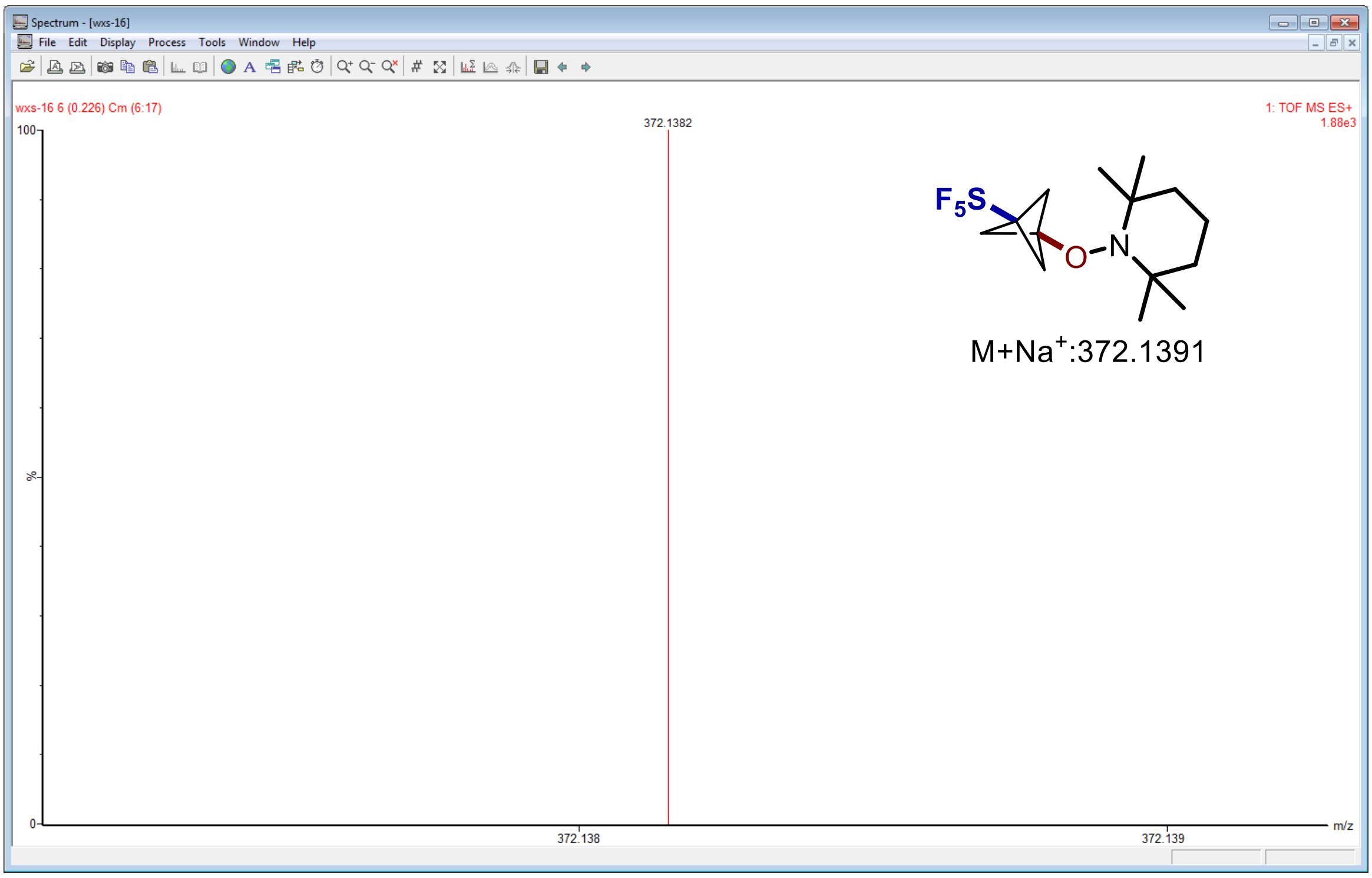
Spectrum - [wxs-16]
File Edit Display Process Tools Window Help
wxs-16 6 (0.226) Cm (6:17)
1: TOF MS ES+
1.88e3
372.1382
100
%
0
372.138
372.139
m/z
F5S
O
N
M+Na+:372.1391

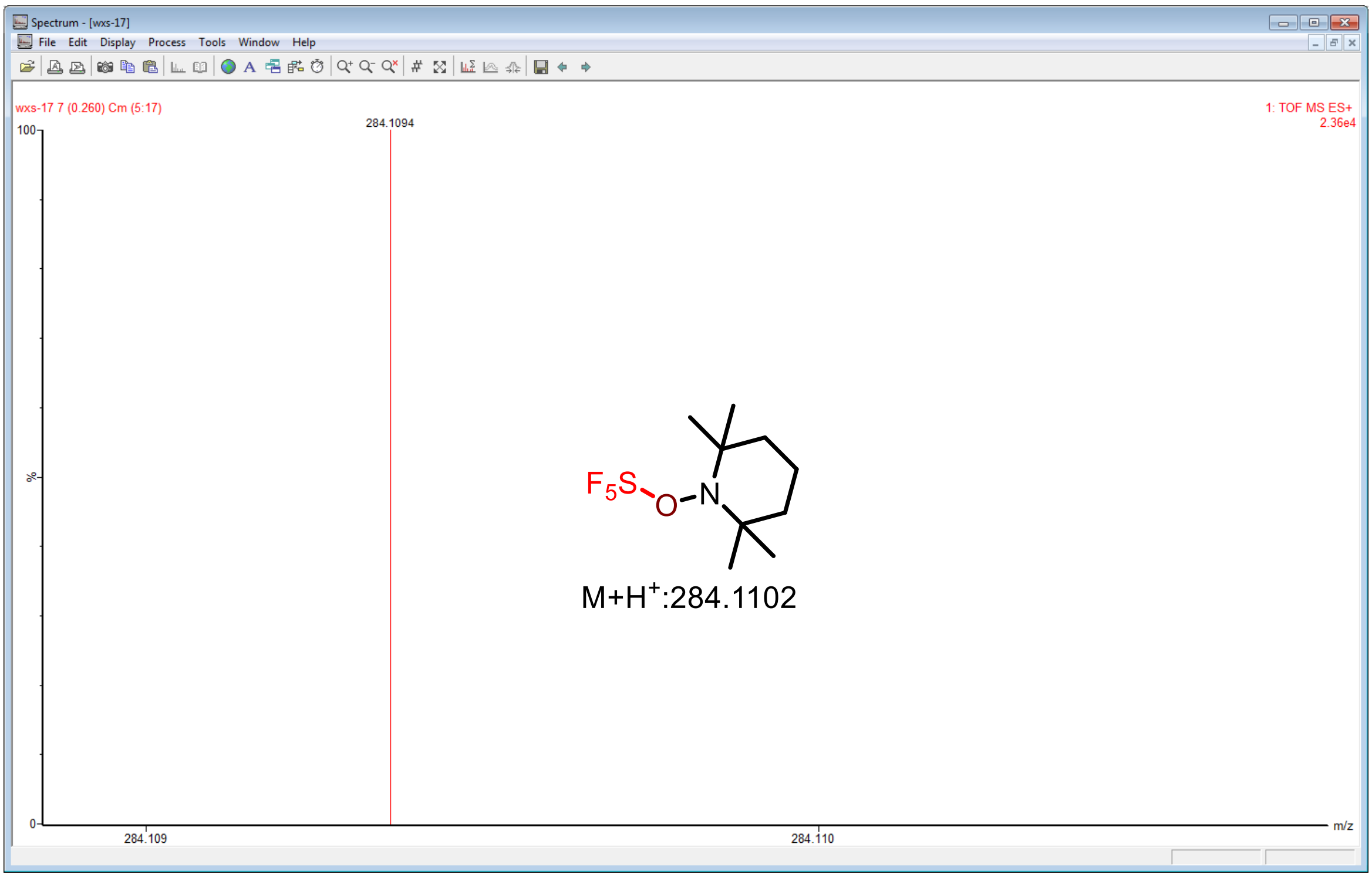
Spectrum - [wxs-17]
File Edit Display Process Tools Window Help
wxs-17 7 (0.260) Cm (5:17)
1: TOF MS ES+
2.36e4
284.1094
100
%
0
284.109
284.110
m/z
F5S
O
N
M+H+:284.1102

By-product 12 analysis

{Ir[dF(CF_3)ppy]$_2$(dtbpy)}(PF_6) (5 mol%) were firstly combined in a 10 mL oven-dried Schlenk tube, The tube was evacuated and backfilled with SF_6 (repeated for 3 times), and then the substrates 5a (0.3 mmol, 3.0 equiv), DIPEA(0.3 mmol, 3.0 equiv) , acetone (2.0 mL) and tricyclo[1.1.1.0^{1,3}]pentane (TCP) (1.0 M in Et_2O, 0.1 mmol, 1.0 equiv), were added quickly under SF_6 atmosphere. The reaction was reacted under visible light source (450-465 nm, 40 W) at 25 °C for 24 h. Then concentrated under vacuum and added 0.7 mL D_2O, by-product **12** was detected by ^{19}F NMR as shown below. The fluorine spectrum signal (-146.65 ppm) was basically same as prediction software (https://fluobase.cstspace.cn/fnmr) referred to *Artificial Intelligence Chemistry*. **2024**, *2*, 100043.

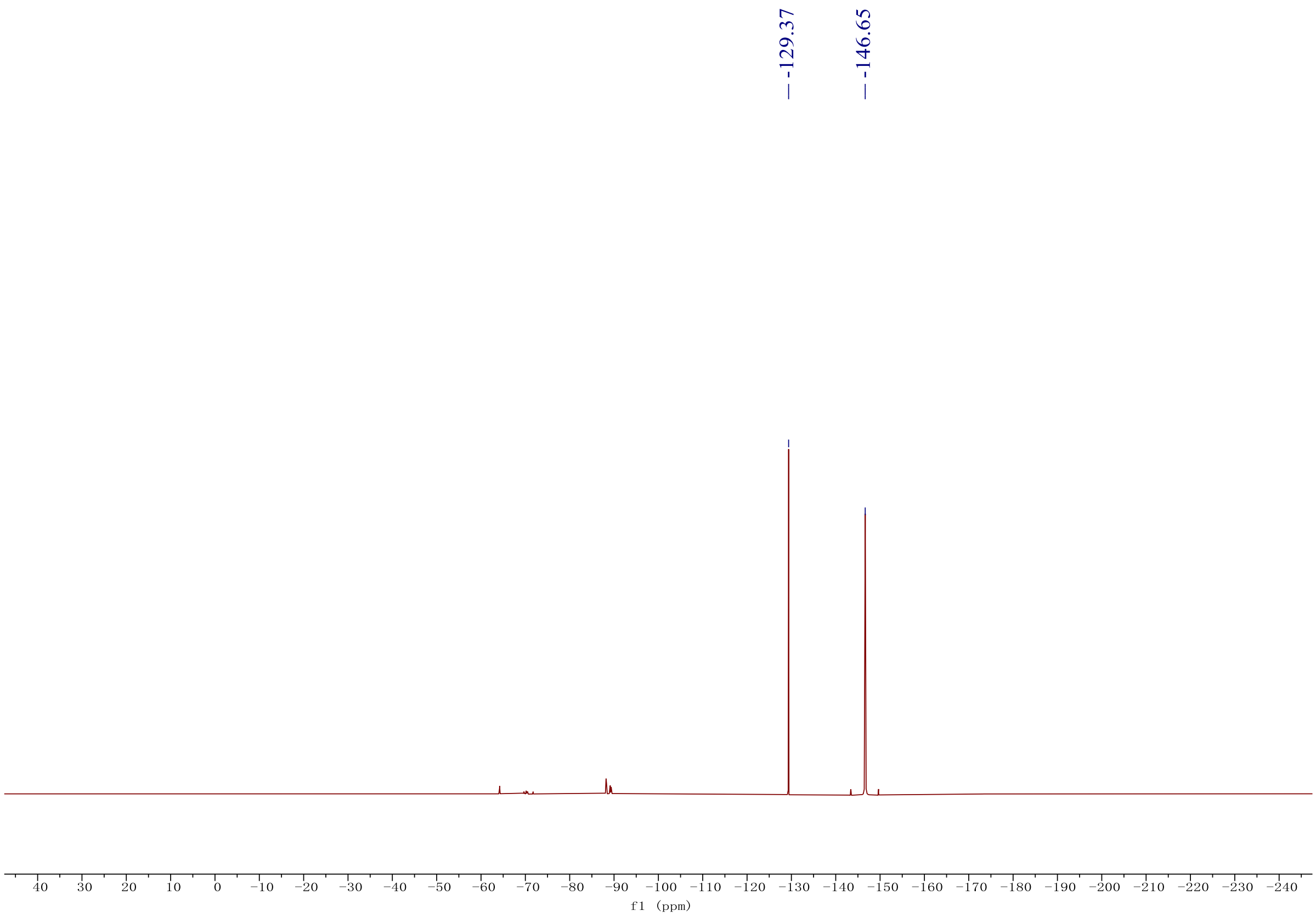

Spectra measurements: emission spectra measurements

Emission intensities were recorded using a Hitachi Fluorescence Spectrophotometer F-4600. Emission spectra measurements were carried out using 0.1 mM solution of {Ir[dF(CF_3)ppy]$_2$(dtbpy)}(PF_6) and different species: **5a** (1.0, 0.1 mM), TCP (tricyclo[1.1.1.0^{1,3}]propellane) (1.0 mM), and DIPEA (1.0, 0.7, 0.5, 0.3, 0.1 mM) in acetone. The samples were prepared in 3 mL quartz cuvettes, equipped with PTFE stoppers. Each sample was irradiated at 365 nm and the emission spectrum was recorded. (Figure S1 and S2)

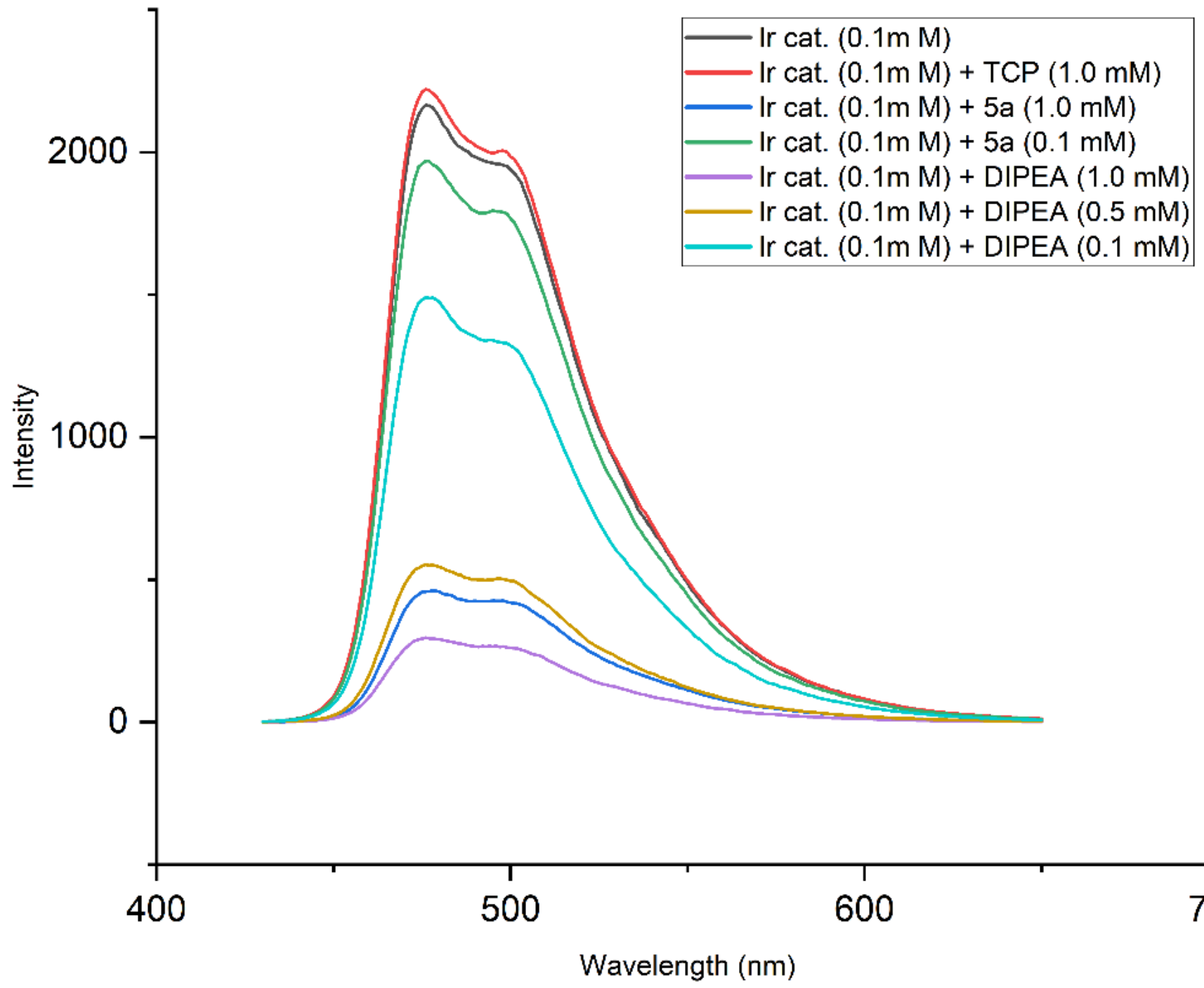

Figure S1 Emission spectra measurements with different species.

Stern-Volmer studies were conducted sperately with DIPEA and trifluoromethyl alkenes (**5a**). Standard solutions of quencher and {Ir[dF(CF_3)ppy]$_2$(dtbpy)} were prepared in acetone. The samples contained 0.1 mM Ir photocatalyst solution and varying quencher concentrations (0.1 mM, 0.3 mM, 0.5 mM, 0.7 mM, 1.0 mM) were prepared in 3 mL quartz cuvettes, equipped with PTFE stoppers. The cuvettes were irradiated at 365 nm and the emission intensity was measured at 476 nm. (Figure S3)

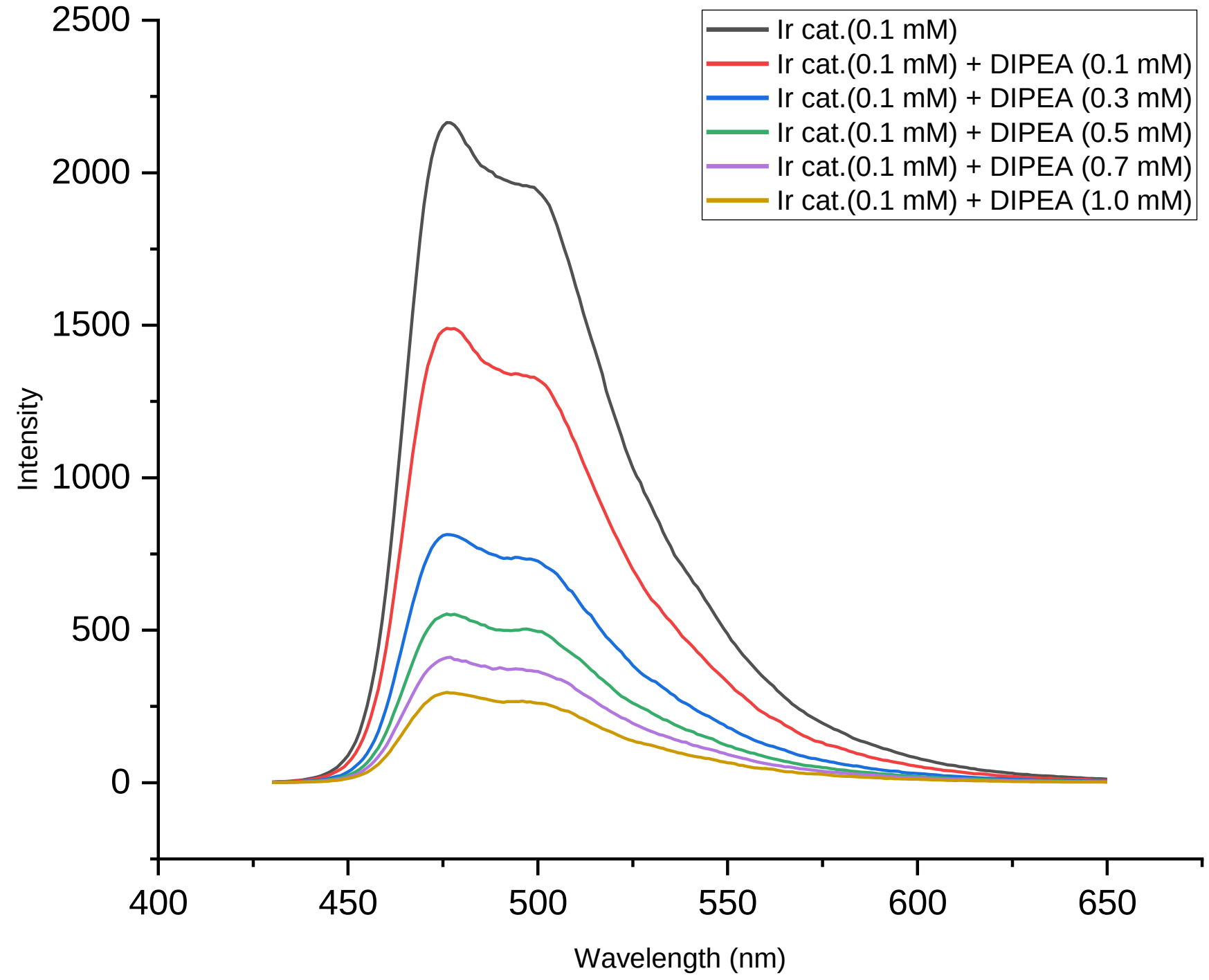

Figure S2 Emission spectra measurements with DIPEA.

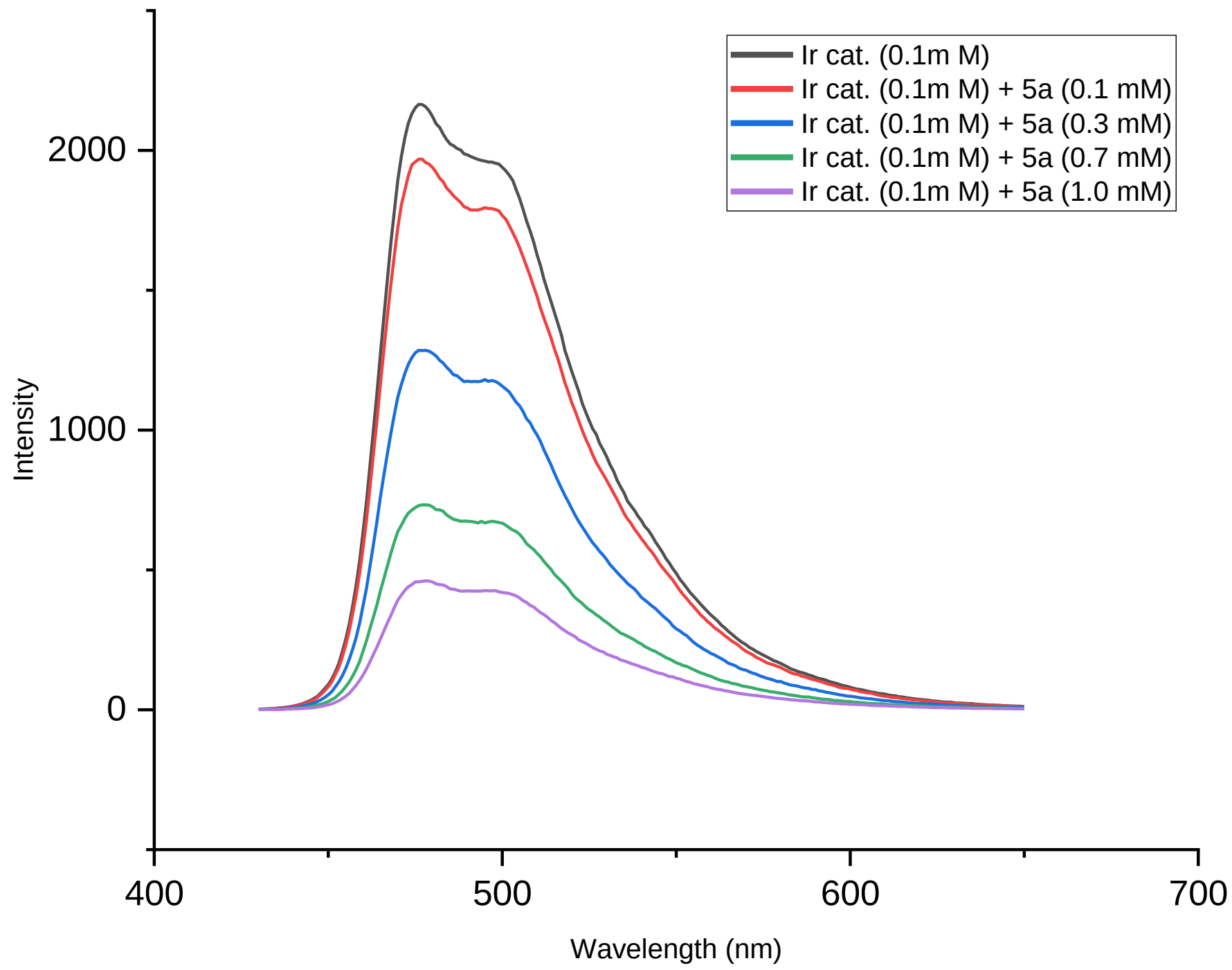

Figure S3 Emission spectra measurements with substrates **5a**.

The ratio of I_0/I was plotted as a function of the quencher concentration (I_0 = emission intensity of the photocatalyst in isolation at the specified wavelength; I = observed intensity as a function of the quencher concentration). The emission spectra and fitted Stern-Volmer equations were shown as Figure S4.

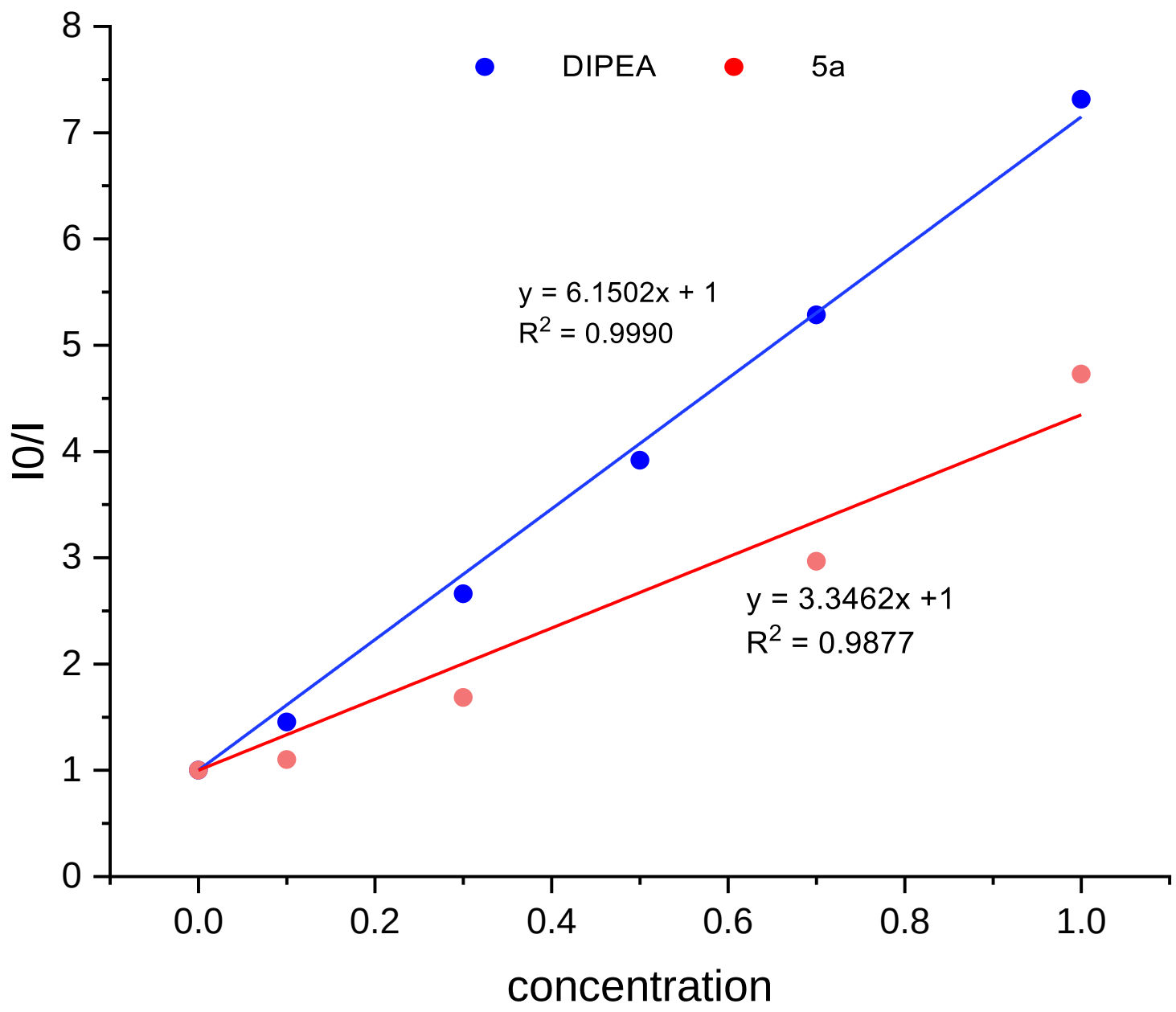

Figure S4 The fitted Stern-Volmer equations.

From the Stern-Volmer plot it was confirmed that DIPEA could quench the excited state of the photocatalyst more efficiently than trifluoromethyl alkenes (**5a**).

DFT calculations

Computational details

Density functional theory (DFT) calculations were performed with the Gaussian 16[1] programs. Geometry optimization and frequency calculations were performed using ωB97XD[2] functional together with the def2-SVP[3-4] basis set. The polarizable continuum model (PCM)[5] implicit solvation model was used to account for the solvation effects. The frequency calculations confirmed that the optimized structures corresponded to either energy minima (no imaginary frequency) or transition structures (one imaginary frequency). Intrinsic reaction coordinates (IRC) calculations at the same level verified the connectivity of located intermediates and transition states. To enhance the accuracy of electronic energies, single-point energy was calculated at ωB97XD/def2-TZVPP[3-4]-PCM level of theory using the optimized structures. The single-point energy of excited state was investigaed using the time-dependent density functional theory (TD-DFT) at ωB97XD/def2-TZVPP-PCM level. The determination of standard enthalpies and Gibbs free energies (at a concentration of 1 $mol \cdot L^{-1}$ and a temperature of 298.15 K/288.15 K) involved the use of Grimme's quasi-harmonic approximation and Head-Gordon's method[6-7], implemented in GoodVibes version 3.0.1[8]. Three-dimensional molecular structures were visualized using CYLview[9].

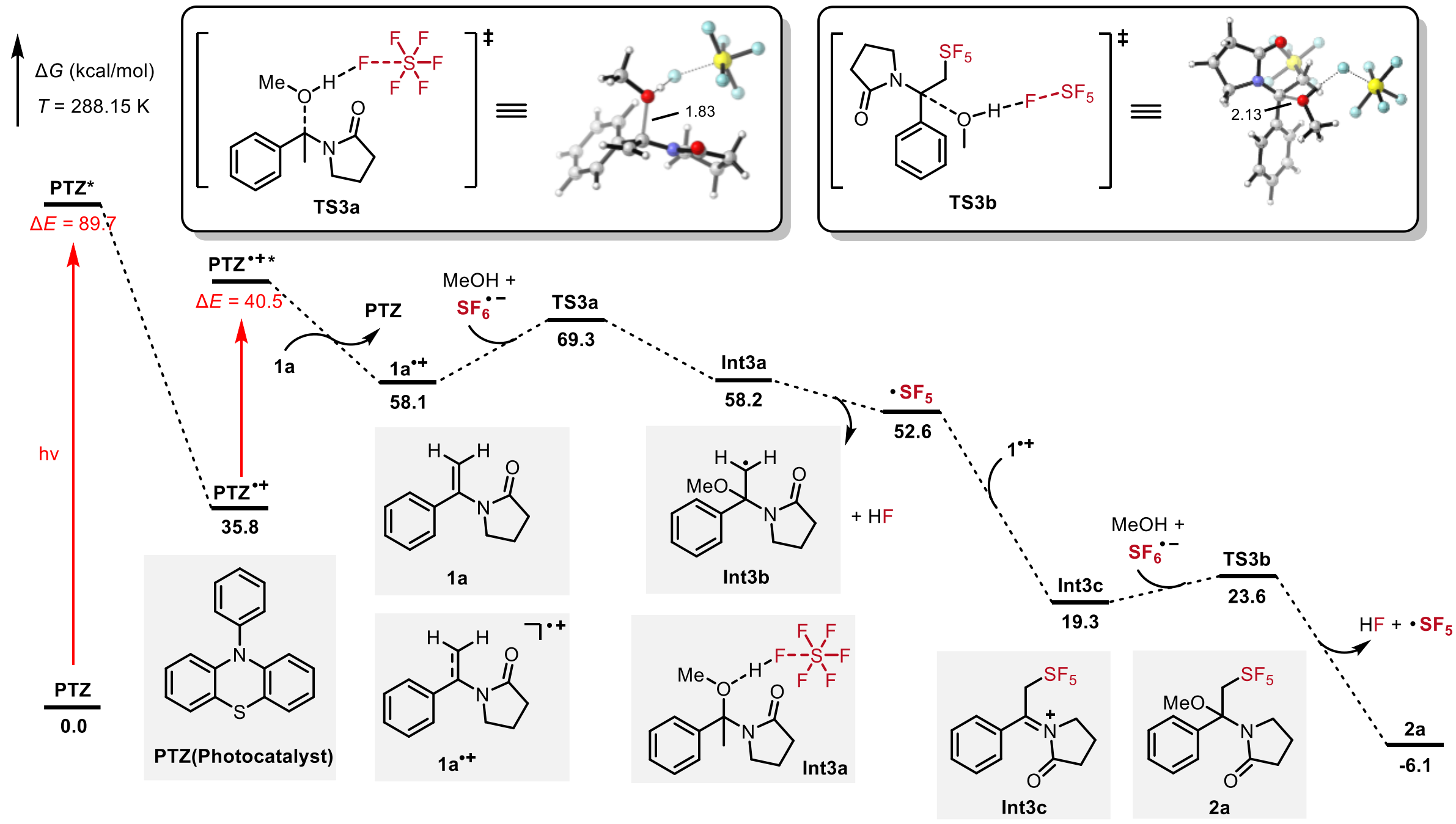

Figure S5 Potential energy surface for the reaction of 1.

References

[1] Frisch, M. J.; Trucks, G. W.; Schlegel, H. B.; Scuseria, G. E.; Robb, M. A.; Cheeseman, J. R.; Scalmani, G.; Barone, V.; Petersson, G. A.; Nakatsuji, H.; Li, X.; Caricato, M.; Marenich, A. V.; Bloino, J.; Janesko, B. G.; Gomperts, R.; Mennucci, B.; Hratchian, H. P.; Ortiz, J. V.; Izmaylov, A. F.; Sonnenberg, J. L.; Williams-Young, D.; Ding, F.; Lipparini, F.; Egidi, F.; Goings, J.; Peng, B.; Petrone, A.; Henderson, T.; Ranasinghe, D.; Zakrzewski, V. G.; Gao, J.; Rega, N.; Zheng, G.; Liang, W.; Hada, M.; Ehara, M.; Toyota, K.; Fukuda, R.; Hasegawa, J.; Ishida, M.; Nakajima, T.; Honda, Y.; Kitao, O.; Nakai, H.; Vreven, T.; Throssell, K.; Montgomery, J. A. Jr.; Peralta, J. E.; Ogliaro, F.; Bearpark, M. J.; Heyd, J. J.; Brothers, E. N.; Kudin, K. N.; Staroverov, V. N.; Keith, T. A.; Kobayashi, R.; Normand, J.; Raghavachari, K.; Rendell, A. P.; Burant, J. C.; Iyengar, S. S.; Tomasi, J.; Cossi, M.; Millam, J. M.; Klene, M.; Adamo, C.; Cammi, R.; Ochterski, J. W.; Martin, R. L.; Morokuma, K.; Farkas, O.; Foresman, J. B.; Fox, D. J. Gaussian, Inc., Wallingford CT, **2019**.

[2] Chai, J.-D.; Head-Gordon M. Long-range corrected hybrid density functionals with damped atom-atom dispersion corrections. *Phys. Chem. Chem. Phys.* **2008**, 10, 6615-6620.

[3] Weigend, F.; Ahlrichs R. Balanced basis sets of split valence, triple zeta valence and quadruple zeta valence quality for H to Rn: design and assessment of accuracy. *Phys. Chem. Chem. Phys.* **2005**, 7, 3297-

3305.

[4] Weigend, F. Accurate Coulomb-fitting basis sets for H to Rn. *Phys. Chem. Chem. Phys.* **2006**, 8, 1057-1065.

[5] Tomasi, J.; Mennucci, B.; Cammi, R. Quantum Mechanical Continuum Solvation Models. *Chem. Rev.* **2005**, 105, 2999-3094.

[6] Grimme, S. Supramolecular Binding Thermodynamics by Dispersion-Corrected Density Functional Theory. *Chem. Eur. J.* **2012**, 18, 9955-9964.

[7] Li, Y.; Gomes, J.; Sharada, S. M.; Bell, A. T.; Head-Gordon, M. Improved Force-Field Parameters for QM/MM Simulations of the Energies of Adsorption for Molecules in Zeolites and a Free Rotor Correction to the Rigid Rotor Harmonic Oscillator Model for Adsorption Enthalpies. *J. Phys. Chem. C.* **2015**, 119, 1840-1850.

[8] Luchini, G.; Alegre-Requena, J. V.; Guan, Y.; Funes-Ardoiz, I.; Paton, R. S. GoodVibes: GoodVibes 3.0.1. **2019**.

[9] Legault, C. Y. CYLview., 1.0 b; Universitee de Sherbrooke: Sherbrooke (Queebec) Canada. **2009**.

Cartesian coordinates and energies for all optimized compounds and transition states:

In reaction of vinyl ether 3a:

PTZ

C	-1.76447500	3.68018900	-0.48548800
C	-2.46315600	2.55111000	-0.06137000
C	-1.79595600	1.34825000	0.16770800
C	-0.41378600	1.23157600	-0.07276700
C	0.27514800	2.37625100	-0.50162600
C	-0.39135600	3.58559400	-0.69256300
C	-0.41336600	-1.23162800	-0.07272600
C	-1.79549300	-1.34878100	0.16774300
C	-2.46226200	-2.55188300	-0.06131800
H	-3.53869300	-2.60379800	0.11811800
C	-1.76317400	-3.68071600	-0.48542400

C	-0.39008800	-3.58563000	-0.69251100
C	0.27598100	-2.37604700	-0.50158600
H	-2.29083700	4.62273600	-0.64521200
H	-3.53960400	2.60264200	0.11807000
H	1.34850000	2.32698300	-0.68039100
H	0.17826100	4.45875600	-1.01730800
H	-2.28919700	-4.62345200	-0.64514200
H	0.17984100	-4.45858700	-1.01725800
H	1.34931500	-2.32637600	-0.68037000
S	-2.68396600	-0.00041100	0.89649100
N	0.24735200	0.00008300	0.11987300
C	1.67913800	0.00027300	0.15599700
C	2.43101500	0.00018400	-1.02278800
C	2.32141700	0.00047400	1.39411900
C	3.82265400	0.00035400	-0.95869900
H	1.91806800	-0.00001000	-1.98707500
C	3.71492600	0.00063800	1.45543200
H	1.71818100	0.00051100	2.30395300
C	4.46595000	0.00058700	0.28038800
H	4.40776200	0.00029800	-1.88056300
H	4.21484700	0.00080900	2.42617900
H	5.55684500	0.00071600	0.32891200

Zero-point correction= 0.261757 (Hartree/Particle)

Thermal correction to Energy= 0.275568

Thermal correction to Enthalpy= 0.276480

Thermal correction to Gibbs Free Energy= 0.220609

sp-E = -1146.71002963 hartree

Thermal correction to Gibbs free energy (ZPG) from GoodVibes: 0.000156 hartree

PTZ*

C	-1.76447500	3.68018900	-0.48548800
C	-2.46315600	2.55111000	-0.06137000
C	-1.79595600	1.34825000	0.16770800
C	-0.41378600	1.23157600	-0.07276700
C	0.27514800	2.37625100	-0.50162600
C	-0.39135600	3.58559400	-0.69256300
C	-0.41336600	-1.23162800	-0.07272600
C	-1.79549300	-1.34878100	0.16774300
C	-2.46226200	-2.55188300	-0.06131800
H	-3.53869300	-2.60379800	0.11811800
C	-1.76317400	-3.68071600	-0.48542400
C	-0.39008800	-3.58563000	-0.69251100
C	0.27598100	-2.37604700	-0.50158600
H	-2.29083700	4.62273600	-0.64521200
H	-3.53960400	2.60264200	0.11807000
H	1.34850000	2.32698300	-0.68039100
H	0.17826100	4.45875600	-1.01730800
H	-2.28919700	-4.62345200	-0.64514200
H	0.17984100	-4.45858700	-1.01725800
H	1.34931500	-2.32637600	-0.68037000
S	-2.68396600	-0.00041100	0.89649100
N	0.24735200	0.00008300	0.11987300
C	1.67913800	0.00027300	0.15599700
C	2.43101500	0.00018400	-1.02278800
C	2.32141700	0.00047400	1.39411900
C	3.82265400	0.00035400	-0.95869900
H	1.91806800	-0.00001000	-1.98707500
C	3.71492600	0.00063800	1.45543200

H	1.71818100	0.00051100	2.30395300
C	4.46595000	0.00058700	0.28038800
H	4.40776200	0.00029800	-1.88056300
H	4.21484700	0.00080900	2.42617900
H	5.55684500	0.00071600	0.32891200

sp-E = -1146.56701532 hartree

SF_6

S	0.00000300	-0.00000600	0.00000600
F	1.18445000	0.34218100	-0.98946500
F	0.29527100	-1.54264100	-0.18001600
F	1.00488800	0.04996700	1.21918100
F	-1.18446000	-0.34218100	0.98946500
F	-1.00488200	-0.04994100	-1.21916000
F	-0.29527200	1.54262600	0.17998400

Zero-point correction= 0.021710 (Hartree/Particle)

Thermal correction to Energy= 0.026872

Thermal correction to Enthalpy= 0.027784

Thermal correction to Gibbs Free Energy= -0.006776

sp-E = -997.306898433 hartree

Thermal correction to Gibbs free energy (ZPG) from GoodVibes: -0.000029 hartree

$SF_6^{\bullet -}$

S	-0.00000500	0.00000100	0.00000000
F	-0.48421100	1.58251200	-0.46468900
F	-1.32550500	-0.08493900	1.09126300
F	-0.98195600	-0.66569200	-1.24390700
F	0.48420700	-1.58250900	0.46468400
F	0.98196200	0.66569600	1.24389500

F	1.32551200	0.08493100	-1.09124400

Zero-point correction= 0.012070 (Hartree/Particle)

Thermal correction to Energy= 0.018197

Thermal correction to Enthalpy= 0.019109

Thermal correction to Gibbs Free Energy= -0.018151

sp-E = -997.425610108 hartree

Thermal correction to Gibbs free energy (ZPG) from GoodVibes: -0.000086 hartree

$PTZ^{\bullet+}$

C	1.66364000	-3.76494300	0.00009800
C	2.43381900	-2.62188700	0.00015000
C	1.82385900	-1.35314300	0.00008800
C	0.41166100	-1.23203800	0.00008000
C	-0.35333000	-2.42362300	-0.00000200
C	0.26165500	-3.65675300	-0.00001400
C	0.41243700	1.23192900	0.00006200
C	1.82472600	1.35210900	0.00006200
C	2.43553600	2.62044400	0.00011800
H	3.52538200	2.68492400	0.00019700
C	1.66613200	3.76401700	0.00006900
C	0.26407800	3.65676900	-0.00002100
C	-0.35173900	2.42405500	0.00000000
H	2.14140700	-4.74521600	0.00011700
H	3.52362400	-2.68710100	0.00023500
H	-1.43959200	-2.36690300	-0.00005600
H	-0.35420400	-4.55708400	-0.00010500
H	2.14455900	4.74396900	0.00008200
H	-0.35116800	4.55751800	-0.00009900
H	-1.43803200	2.36811500	-0.00002700

S	2.89756300	-0.00086400	-0.00024800
N	-0.22606000	0.00013200	0.00002800
C	-1.67258300	0.00047700	-0.00002200
C	-2.35058400	0.00056800	-1.21649800
C	-2.35057800	0.00065000	1.21644200
C	-3.74388800	0.00091900	-1.21010700
H	-1.78999100	0.00040200	-2.15295500
C	-3.74388400	0.00099900	1.21008000
H	-1.78999300	0.00054900	2.15289600
C	-4.43852000	0.00113900	-0.00000200
H	-4.28759400	0.00102200	-2.15624500
H	-4.28757800	0.00116500	2.15622700
H	-5.53003200	0.00141200	0.00001500

Zero-point correction= 0.262905 (Hartree/Particle)

Thermal correction to Energy= 0.276580

Thermal correction to Enthalpy= 0.277492

Thermal correction to Gibbs Free Energy= 0.221860

sp-E = -1146.52397955 hartree

Thermal correction to Gibbs free energy (ZPG) from GoodVibes: -0.000222 hartree

$PTZ^{\bullet+}$*

C	1.66364000	-3.76494300	0.00009800
C	2.43381900	-2.62188700	0.00015000
C	1.82385900	-1.35314300	0.00008800
C	0.41166100	-1.23203800	0.00008000
C	-0.35333000	-2.42362300	-0.00000200
C	0.26165500	-3.65675300	-0.00001400
C	0.41243700	1.23192900	0.00006200
C	1.82472600	1.35210900	0.00006200

C	2.43553600	2.62044400	0.00011800
H	3.52538200	2.68492400	0.00019700
C	1.66613200	3.76401700	0.00006900
C	0.26407800	3.65676900	-0.00002100
C	-0.35173900	2.42405500	0.00000000
H	2.14140700	-4.74521600	0.00011700
H	3.52362400	-2.68710100	0.00023500
H	-1.43959200	-2.36690300	-0.00005600
H	-0.35420400	-4.55708400	-0.00010500
H	2.14455900	4.74396900	0.00008200
H	-0.35116800	4.55751800	-0.00009900
H	-1.43803200	2.36811500	-0.00002700
S	2.89756300	-0.00086400	-0.00024800
N	-0.22606000	0.00013200	0.00002800
C	-1.67258300	0.00047700	-0.00002200
C	-2.35058400	0.00056800	-1.21649800
C	-2.35057800	0.00065000	1.21644200
C	-3.74388800	0.00091900	-1.21010700
H	-1.78999100	0.00040200	-2.15295500
C	-3.74388400	0.00099900	1.21008000
H	-1.78999300	0.00054900	2.15289600
C	-4.43852000	0.00113900	-0.00000200
H	-4.28759400	0.00102200	-2.15624500
H	-4.28757800	0.00116500	2.15622700
H	-5.53003200	0.00141200	0.00001500

sp-E = -1146.45944871 hartree

3a

C	-2.46450800	-0.03890900	-0.56079500

C	-3.37671300	-1.00623600	-0.65987200
H	-3.72606300	-1.31994700	-1.64364000
H	-3.79139100	-1.48768000	0.22887400
C	-0.89231500	1.27165200	0.58777500
H	-1.04843100	2.09007000	-0.13729500
H	-0.83144700	1.72971700	1.58625000
O	-2.02136300	0.42557800	0.62903300
C	0.39521200	0.53988900	0.27593900
C	1.44754500	1.20751400	-0.35855500
C	0.56346400	-0.79895600	0.64468800
C	2.65289100	0.55267500	-0.61099400
H	1.32288700	2.25083600	-0.66174700
C	1.76541700	-1.45689700	0.38593700
H	-0.25835400	-1.32796400	1.13215700
C	2.81452400	-0.78253000	-0.23997600
H	3.46661200	1.08579400	-1.10780400
H	1.88347600	-2.50354400	0.67565100
H	3.75559700	-1.29850500	-0.44278700
H	-2.03507900	0.44738300	-1.44680600

Zero-point correction= 0.167599 (Hartree/Particle)

Thermal correction to Energy= 0.176149

Thermal correction to Enthalpy= 0.177062

Thermal correction to Gibbs Free Energy= 0.133337

sp-E = -424.184551810 hartree

Thermal correction to Gibbs free energy (ZPG) from GoodVibes: 0.000266 hartree

$3a^{\bullet+}$

C	-2.81987400	0.32589000	0.03478600
C	-3.70892300	0.36012700	-1.05030500

H	-4.53787200	1.06870900	-1.04462600
H	-3.56673300	-0.32170900	-1.89165500
C	-0.87361100	-0.58008800	1.10299200
H	-1.18920200	0.11064700	1.89544200
H	-0.94061100	-1.61624300	1.45406800
O	-1.86510800	-0.51342600	0.01805000
C	0.47161600	-0.24890700	0.53388500
C	0.92994800	1.07346800	0.53960900
C	1.26139000	-1.25545000	-0.03328200
C	2.16934700	1.38493600	-0.01558800
H	0.32010300	1.86397000	0.98539100
C	2.49918600	-0.94162100	-0.58959200
H	0.90707100	-2.28907400	-0.03621400
C	2.95260700	0.37819100	-0.58124900
H	2.52625200	2.41648100	-0.00426300
H	3.11366200	-1.73004400	-1.02828200
H	3.92432700	0.62300700	-1.01514400
H	-2.92624700	1.00238600	0.89335500

Zero-point correction= 0.167529 (Hartree/Particle)

Thermal correction to Energy= 0.176105

Thermal correction to Enthalpy= 0.177018

Thermal correction to Gibbs Free Energy= 0.133300

sp-E = -423.952824901 hartree

Thermal correction to Gibbs free energy (ZPG) from GoodVibes: -0.000065 hartree

MeOH

O	1.50783900	-0.12565200	-0.72616500
H	1.01286000	0.68180400	-0.55797000
C	1.76542800	-0.74301600	0.50864400

H	0.84265300	-1.02672800	1.04761700
H	2.33175000	-1.66469300	0.31175200
H	2.37450200	-0.11415300	1.18389500

Zero-point correction= 0.051677 (Hartree/Particle)

Thermal correction to Energy= 0.054851

Thermal correction to Enthalpy= 0.055764

Thermal correction to Gibbs Free Energy= 0.029849

sp-E = -115.742168532 hartree

Thermal correction to Gibbs free energy (ZPG) from GoodVibes: -0.000008 hartree

TS1a

C	-0.69580000	1.86437700	0.15818200
C	0.41487400	1.81928500	1.04179800
H	1.38765900	2.17575600	0.70764600
H	0.30711500	1.33298200	2.01127400
C	-3.01665800	1.61451300	-0.20450300
H	-3.55351400	2.51716100	0.11708800
H	-2.69273200	1.74192200	-1.24748400
O	-1.84517600	1.49579200	0.63068700
C	-3.86605900	0.38492900	-0.04581700
C	-5.15027000	0.47251400	0.49665800
C	-3.37125100	-0.86216300	-0.44827000
C	-5.93828400	-0.67191400	0.62984100
H	-5.54022300	1.44208800	0.81705500
C	-4.15432100	-2.00467400	-0.30721600
H	-2.36298100	-0.92670500	-0.86507300
C	-5.44060600	-1.91085300	0.22991200
H	-6.94221000	-0.59390800	1.05236100
H	-3.76179300	-2.97434000	-0.62081600

H	-6.05497200	-2.80750500	0.33708800
H	-0.67802400	2.53703500	-0.70755400
O	-0.26014500	0.34046400	-1.04478800
C	0.43096700	0.65797900	-2.24034100
H	1.37967800	1.17222100	-2.03063100
H	0.64728100	-0.26906900	-2.79093200
H	-0.21321700	1.29511400	-2.86076100
H	0.35414000	-0.21089700	-0.44624200
F	1.28430300	-0.78731100	0.48470500
S	3.31755300	-0.41149000	0.19387700
F	3.08950700	1.16931400	-0.43134900
F	3.44396300	0.22532700	1.72752900
F	4.89009800	-0.19113100	0.00155200
F	3.62545300	-1.91498100	0.78356900
F	3.24556000	-1.02588800	-1.35753000

Zero-point correction= 0.238003 (Hartree/Particle)

Thermal correction to Energy= 0.258204

Thermal correction to Enthalpy= 0.259116

Thermal correction to Gibbs Free Energy= 0.186939

sp-E = -1537.15490877 hartree

Thermal correction to Gibbs free energy (ZPG) from GoodVibes: 0.001036 hartree

Int1a

C	-0.61540400	1.44902200	0.74964600
C	0.22427900	0.97773900	1.87924400
H	0.79966500	1.68469800	2.47702900
H	0.41164900	-0.09410000	1.97063100
C	-2.65750800	1.02054900	-0.34113300
H	-2.90912300	2.08281600	-0.15270500

H	-2.26028900	0.95896600	-1.36723300
O	-1.68255200	0.59894700	0.59178200
C	-3.89439500	0.17100400	-0.20494800
C	-4.29147900	-0.32246600	1.04231000
C	-4.68414600	-0.10231800	-1.32608600
C	-5.45996500	-1.07311900	1.16510800
H	-3.67172000	-0.12133000	1.91808300
C	-5.85791200	-0.84599100	-1.20297500
H	-4.37677300	0.26754600	-2.30811700
C	-6.24858000	-1.33460800	0.04374800
H	-5.75726900	-1.45637900	2.14398900
H	-6.46479700	-1.05198900	-2.08749200
H	-7.16402200	-1.92247600	0.14081100
H	-0.94335500	2.49916400	0.88710600
O	0.12667000	1.42025900	-0.49163600
C	0.97215900	2.52883200	-0.74411200
H	1.76305900	2.61408600	0.01595700
H	1.43468000	2.36095600	-1.72337300
H	0.38294400	3.45847600	-0.77366200
H	0.68380300	0.04605600	-0.72796900
F	1.07657100	-0.84117900	-0.78873600
S	3.48979900	-0.58696000	-0.05871400
F	3.18451400	0.70839800	0.90925900
F	3.23384700	-1.58556700	1.20621000
F	5.02350100	-0.49525600	0.28931700
F	3.76155000	-1.86580300	-1.02467600
F	3.69329600	0.42377900	-1.32911000

Zero-point correction= 0.238558 (Hartree/Particle)

Thermal correction to Energy= 0.259177

Thermal correction to Enthalpy= 0.260089

Thermal correction to Gibbs Free Energy= 0.184953

sp-E = -1537.17617243 hartree

Thermal correction to Gibbs free energy (ZPG) from GoodVibes: 0.001882 hartree

Int1b

C	-2.31125500	0.48074700	0.20433300
C	-2.94848700	1.76959500	-0.18120300
H	-3.87797300	2.08787900	0.29372800
H	-2.60114100	2.27597300	-1.08473200
C	-0.20758900	-0.56198300	0.31670400
H	-0.41752700	-0.75021400	1.38948500
H	-0.49152500	-1.47755600	-0.22809000
O	-0.96854800	0.53648000	-0.12627700
C	1.26228700	-0.28096600	0.13386700
C	1.75841900	1.02574700	0.18001600
C	2.15831300	-1.33997300	-0.04761600
C	3.12598200	1.26771100	0.05020600
H	1.06089600	1.85500300	0.31007900
C	3.52664000	-1.10011600	-0.16941700
H	1.78079700	-2.36511300	-0.09786700
C	4.01495400	0.20613500	-0.12172600
H	3.49989200	2.29373900	0.08414400
H	4.21384000	-1.93741500	-0.31091900
H	5.08577400	0.39643100	-0.22300100
H	-2.43462900	0.28932500	1.29508200
O	-2.88571700	-0.61045600	-0.49394700
C	-4.03470600	-1.15431300	0.09835500
H	-4.87778200	-0.44052700	0.11384300

H	-4.33242300	-2.02683600	-0.49743500
H	-3.84142800	-1.48438400	1.13635900

Zero-point correction= 0.209153 (Hartree/Particle)

Thermal correction to Energy= 0.220886

Thermal correction to Enthalpy= 0.221799

Thermal correction to Gibbs Free Energy= 0.169505

sp-E = -539.281666126 hartree

Thermal correction to Gibbs free energy (ZPG) from GoodVibes: 0.000564 hartree

HF

F	1.89002900	-0.41187000	0.00000000
H	0.96766500	-0.41187000	0.00000000

Zero-point correction= 0.009411 (Hartree/Particle)

Thermal correction to Energy= 0.011692

Thermal correction to Enthalpy= 0.012604

Thermal correction to Gibbs Free Energy= -0.006337

sp-E = -100.470308492 hartree

Thermal correction to Gibbs free energy (ZPG) from GoodVibes: 0.000000 hartree

$SF_5^{\bullet}$

S	0.00059300	-0.00019600	-0.20904400
F	-1.58750600	0.33907500	-0.24670100
F	0.00180000	-0.00050000	1.36160500
F	-0.34090600	-1.58717600	-0.24740500
F	1.58805300	-0.33930600	-0.24857500
F	0.33750400	1.58825500	-0.24729000

Zero-point correction= 0.015322 (Hartree/Particle)

Thermal correction to Energy= 0.020493

Thermal correction to Enthalpy= 0.021406

Thermal correction to Gibbs Free Energy= -0.013515

sp-E = -897.403778573 hartree

Thermal correction to Gibbs free energy (ZPG) from GoodVibes: -0.000044 hartree

Int1c

C	-0.36313800	1.44402700	-0.34867700
C	-1.56936900	1.28044500	0.49069000
H	-2.26358000	2.10188700	0.25752200
H	-1.31927300	1.28367800	1.55643900
S	-2.44696700	-0.27974800	0.09463100
F	-1.06948800	-0.94924600	-0.43775200
F	-3.19977700	-1.63275200	-0.27785700
F	-2.74748700	0.25027900	-1.39307300
F	-2.15792200	-0.87563900	1.54763100
F	-3.82993700	0.32887300	0.60799400
C	1.99435200	1.86909700	-0.56392700
H	1.71943600	1.98317400	-1.61982500
H	2.39081800	2.80498200	-0.15851400
O	0.71155700	1.70252400	0.20398400
C	2.84929200	0.67963600	-0.28272000
C	2.79223100	-0.43798300	-1.12457000
C	3.68662100	0.66594500	0.83941500
C	3.56660100	-1.56098400	-0.84395500
H	2.14561300	-0.42966900	-2.00575700
C	4.45814600	-0.45915400	1.11799400
H	3.73549200	1.53879600	1.49456700
C	4.39712400	-1.57158200	0.27731500
H	3.52439800	-2.42910900	-1.50406100
H	5.11152900	-0.46746800	1.99225000

H	5.00449300	-2.45230600	0.49578100
H	-0.41956000	1.37148400	-1.44626300

Zero-point correction= 0.190020 (Hartree/Particle)

Thermal correction to Energy= 0.203507

Thermal correction to Enthalpy= 0.204420

Thermal correction to Gibbs Free Energy= 0.148295

sp-E = -1321.43595953 hartree

Thermal correction to Gibbs free energy (ZPG) from GoodVibes: 0.000675 hartree

4a

C	0.69803500	1.54787200	-0.03516800
C	1.83775100	0.74781000	-0.65667900
H	2.78979000	1.27415200	-0.53525900
H	1.63360300	0.59320200	-1.72172700
S	2.14532900	-0.91513800	0.02479000
F	1.01918100	-0.81677900	1.17782000
F	2.47602800	-2.39207600	0.58085000
F	3.23655000	-0.35172400	1.07434500
F	1.07009800	-1.60274800	-0.96179100
F	3.31013500	-1.14867100	-1.06987900
C	-1.62436900	1.39183300	0.29843400
H	-1.38452500	1.49613700	1.37497200
H	-1.92777900	2.38839800	-0.06378100
O	-0.48149700	0.94021900	-0.39270200
C	-2.74650400	0.40073300	0.12428700
C	-2.47520900	-0.96017100	-0.05135400
C	-4.07660000	0.82932300	0.17620700
C	-3.51993800	-1.87638600	-0.16949300
H	-1.43772000	-1.29287100	-0.10426600

C	-5.12140000	-0.08808900	0.06622000
H	-4.29870200	1.89276700	0.30121900
C	-4.84549400	-1.44458700	-0.10811000
H	-3.29630800	-2.93643100	-0.31048600
H	-6.15593000	0.26006400	0.10841900
H	-5.66259500	-2.16355900	-0.20053100
O	0.73482700	2.84035900	-0.56259100
C	1.47063600	3.77830300	0.18590000
H	1.36053200	4.74993300	-0.31061400
H	1.08861200	3.85802900	1.21887200
H	2.54548800	3.52819100	0.22707500
H	0.80426100	1.59770700	1.06824800

Zero-point correction= 0.233864 (Hartree/Particle)

Thermal correction to Energy= 0.250051

Thermal correction to Enthalpy= 0.250963

Thermal correction to Gibbs Free Energy= 0.188519

sp-E = -1436.80595013 hartree

Thermal correction to Gibbs free energy (ZPG) from GoodVibes: 0.000658 hartree

In reaction of 5b:

Ir(III)

Ir	0.00000100	-0.25413900	0.00000100
C	1.22601100	-1.99399800	1.96979500
C	-1.17127300	-2.37778900	1.86080700
C	-1.03792100	-3.32335500	2.86831200
C	0.18593500	-3.63463300	3.45073600
C	1.30077600	-2.95859000	2.98503800
C	2.35812300	-1.23877100	1.42831800
C	2.94154800	0.40331800	-0.14016200

C	4.28055800	0.32139200	0.19481400
C	4.66601100	-0.57921200	1.19061300
C	3.70368700	-1.35902800	1.80637200
H	-2.16612900	-2.19067600	1.45362300
H	0.27197600	-4.37913500	4.24120400
H	2.59493800	1.08637000	-0.91479600
H	5.71330700	-0.67403800	1.48260200
H	3.98550200	-2.06696500	2.57963800
N	2.01185200	-0.35049100	0.45887600
C	-0.05519800	2.68018600	0.74221400
C	-0.21257300	1.38832000	2.66253600
C	-0.25236300	2.51158600	3.47343300
C	-0.19579200	3.78258600	2.89456000
C	-0.09254200	3.84604600	1.50148100
C	0.05517100	2.68018800	-0.74220500
C	0.21255800	1.38832900	-2.66253000
C	0.25233800	2.51159700	-3.47342400
C	0.19575600	3.78259600	-2.89454700
C	0.09250400	3.84605100	-1.50146900
H	-0.25817400	0.38316600	3.08635000
H	-0.32629600	2.38882100	4.55461100
H	-0.03884700	4.82023700	1.01711700
H	0.25816800	0.38317600	-3.08634700
H	0.32627300	2.38883600	-4.55460200
H	0.03880000	4.82024100	-1.01710200
N	-0.12070700	1.46709700	1.33020600
N	0.12069200	1.46710200	-1.33020000
C	-1.22599400	-1.99400700	-1.96979500
C	1.17129400	-2.37777700	-1.86080600

C	1.03795000	-3.32334700	-2.86831100
C	-0.18590400	-3.63463700	-3.45073300
C	-1.30075000	-2.95860200	-2.98503600
C	-2.35811100	-1.23878800	-1.42832000
C	-2.94155200	0.40329600	0.14016100
C	-4.28055900	0.32136300	-0.19482100
C	-4.66600400	-0.57924100	-1.19062200
C	-3.70367300	-1.35905100	-1.80637800
H	2.16614900	-2.19065600	-1.45362300
H	-0.27193900	-4.37914000	-4.24120000
H	-2.59494800	1.08634800	0.91479700
H	-5.71329800	-0.67407200	-1.48261600
H	-3.98548200	-2.06698900	-2.57964700
N	-2.01184900	-0.35050700	-0.45887500
C	0.26991700	5.02852500	-3.72520400
H	-0.26817800	5.85961700	-3.25068900
H	1.32204800	5.33383500	-3.83982000
H	-0.13948200	4.86249900	-4.73021300
C	-0.26996200	5.02851700	3.72521400
H	0.26827200	5.85956300	3.25077600
H	-1.32208200	5.33392100	3.83967800
H	0.13928200	4.86245300	4.73028000
C	-5.30199900	1.18822300	0.49197200
F	-5.88453000	2.02582800	-0.37168800
F	-6.27385800	0.45128800	1.03702400
F	-4.75921000	1.92521300	1.46314500
F	-2.47368500	-3.25934100	-3.54841800
F	2.12209500	-3.96380900	-3.30167200
F	-2.12206100	-3.96382600	3.30167400

F	2.47371300	-3.25931700	3.54842200
C	-0.04096300	-1.69621200	1.39752300
C	0.04097800	-1.69621000	-1.39752300
C	5.30199100	1.18825500	-0.49198400
F	4.75919200	1.92525800	-1.46314100
F	5.88454000	2.02584600	0.37167700
F	6.27383900	0.45132100	-1.03705900

Zero-point correction= 0.520216 (Hartree/Particle)

Thermal correction to Energy= 0.563286

Thermal correction to Enthalpy= 0.564230

Thermal correction to Gibbs Free Energy= 0.438327

sp-E = -2707.19099236 hartree

Thermal correction to Gibbs free energy (ZPG) from GoodVibes: 0.003624 hartree

Ir(III)*

Ir	0.00000100	-0.25413900	0.00000100
C	1.22601100	-1.99399800	1.96979500
C	-1.17127300	-2.37778900	1.86080700
C	-1.03792100	-3.32335500	2.86831200
C	0.18593500	-3.63463300	3.45073600
C	1.30077600	-2.95859000	2.98503800
C	2.35812300	-1.23877100	1.42831800
C	2.94154800	0.40331800	-0.14016200
C	4.28055800	0.32139200	0.19481400
C	4.66601100	-0.57921200	1.19061300
C	3.70368700	-1.35902800	1.80637200
H	-2.16612900	-2.19067600	1.45362300
H	0.27197600	-4.37913500	4.24120400
H	2.59493800	1.08637000	-0.91479600

H	5.71330700	-0.67403800	1.48260200
H	3.98550200	-2.06696500	2.57963800
N	2.01185200	-0.35049100	0.45887600
C	-0.05519800	2.68018600	0.74221400
C	-0.21257300	1.38832000	2.66253600
C	-0.25236300	2.51158600	3.47343300
C	-0.19579200	3.78258600	2.89456000
C	-0.09254200	3.84604600	1.50148100
C	0.05517100	2.68018800	-0.74220500
C	0.21255800	1.38832900	-2.66253000
C	0.25233800	2.51159700	-3.47342400
C	0.19575600	3.78259600	-2.89454700
C	0.09250400	3.84605100	-1.50146900
H	-0.25817400	0.38316600	3.08635000
H	-0.32629600	2.38882100	4.55461100
H	-0.03884700	4.82023700	1.01711700
H	0.25816800	0.38317600	-3.08634700
H	0.32627300	2.38883600	-4.55460200
H	0.03880000	4.82024100	-1.01710200
N	-0.12070700	1.46709700	1.33020600
N	0.12069200	1.46710200	-1.33020000
C	-1.22599400	-1.99400700	-1.96979500
C	1.17129400	-2.37777700	-1.86080600
C	1.03795000	-3.32334700	-2.86831100
C	-0.18590400	-3.63463700	-3.45073300
C	-1.30075000	-2.95860200	-2.98503600
C	-2.35811100	-1.23878800	-1.42832000
C	-2.94155200	0.40329600	0.14016100
C	-4.28055900	0.32136300	-0.19482100

C	-4.66600400	-0.57924100	-1.19062200
C	-3.70367300	-1.35905100	-1.80637800
H	2.16614900	-2.19065600	-1.45362300
H	-0.27193900	-4.37914000	-4.24120000
H	-2.59494800	1.08634800	0.91479700
H	-5.71329800	-0.67407200	-1.48261600
H	-3.98548200	-2.06698900	-2.57964700
N	-2.01184900	-0.35050700	-0.45887500
C	0.26991700	5.02852500	-3.72520400
H	-0.26817800	5.85961700	-3.25068900
H	1.32204800	5.33383500	-3.83982000
H	-0.13948200	4.86249900	-4.73021300
C	-0.26996200	5.02851700	3.72521400
H	0.26827200	5.85956300	3.25077600
H	-1.32208200	5.33392100	3.83967800
H	0.13928200	4.86245300	4.73028000
C	-5.30199900	1.18822300	0.49197200
F	-5.88453000	2.02582800	-0.37168800
F	-6.27385800	0.45128800	1.03702400
F	-4.75921000	1.92521300	1.46314500
F	-2.47368500	-3.25934100	-3.54841800
F	2.12209500	-3.96380900	-3.30167200
F	-2.12206100	-3.96382600	3.30167400
F	2.47371300	-3.25931700	3.54842200
C	-0.04096300	-1.69621200	1.39752300
C	0.04097800	-1.69621000	-1.39752300
C	5.30199100	1.18825500	-0.49198400
F	4.75919200	1.92525800	-1.46314100
F	5.88454000	2.02584600	0.37167700

F	6.27383900	0.45132100	-1.03705900

sp-E = -2707.05469732 hartree

Ir(II)

Ir	0.00000700	-0.27345600	0.00000300
C	1.25417300	-2.00900100	1.96396400
C	-1.13997700	-2.41309600	1.88371600
C	-0.98644500	-3.36297900	2.88244500
C	0.24801000	-3.66922300	3.44736100
C	1.35032600	-2.98003700	2.97082300
C	2.37276100	-1.23584100	1.41795500
C	2.91734100	0.43262600	-0.13749300
C	4.25892200	0.37691300	0.19552100
C	4.66661600	-0.52656700	1.18013900
C	3.72238900	-1.33185100	1.79119700
H	-2.14136200	-2.22873200	1.48963900
H	0.35056300	-4.41850000	4.23133700
H	2.54282300	1.11645700	-0.89917300
H	5.71658200	-0.60188900	1.46860400
H	4.02053300	-2.03910300	2.55906600
N	2.00770300	-0.35050400	0.45335100
C	-0.07224700	2.64206900	0.70830200
C	-0.27078700	1.35129800	2.66840400
C	-0.34873100	2.46384100	3.47730100
C	-0.29113000	3.75478200	2.87441800
C	-0.15381500	3.82285300	1.50868900
C	0.07215100	2.64207400	-0.70828700
C	0.27075500	1.35131700	-2.66839200
C	0.34864800	2.46386500	-3.47728500

C	0.29098500	3.75480300	-2.87440000
C	0.15366400	3.82286400	-1.50867100
H	-0.31625900	0.34560500	3.09527700
H	-0.45426400	2.34821600	4.55681300
H	-0.10477600	4.79979800	1.02608800
H	0.31627600	0.34562800	-3.09526700
H	0.45418900	2.34824900	-4.55679700
H	0.10457700	4.79980600	-1.02606900
N	-0.14525000	1.40959700	1.33272400
N	0.14521200	1.40960700	-1.33271200
C	-1.25411000	-2.00901500	-1.96397700
C	1.14004900	-2.41305800	-1.88371800
C	0.98654300	-3.36293700	-2.88245600
C	-0.24790200	-3.66920400	-3.44738100
C	-1.35023500	-2.98004600	-2.97084400
C	-2.37271800	-1.23588400	-1.41796800
C	-2.91734600	0.43254800	0.13750000
C	-4.25892400	0.37681100	-0.19552500
C	-4.66659000	-0.52666200	-1.18016100
C	-3.72234100	-1.33191700	-1.79122200
H	2.14142800	-2.22867600	-1.48963400
H	-0.35043400	-4.41847800	-4.23136300
H	-2.54285000	1.11637600	0.89919400
H	-5.71655200	-0.60200000	-1.46863700
H	-4.02046400	-2.03916400	-2.55910500
N	-2.00768700	-0.35055200	-0.45334900
C	0.37888000	4.98690500	-3.72946100
H	0.32204800	5.90502000	-3.12897600
H	1.32366700	5.00110300	-4.29550800

H	-0.43674900	5.00592500	-4.46951700
C	-0.37907800	4.98687900	3.72948100
H	-0.32230700	5.90499700	3.12899400
H	-1.32385400	5.00102700	4.29554600
H	0.43656600	5.00594600	4.46952000
C	-5.25856500	1.27716000	0.47744100
F	-5.80739100	2.13159500	-0.39357000
F	-6.26165400	0.57364000	1.01386800
F	-4.70608700	2.00007800	1.45350500
F	-2.53457700	-3.27756500	-3.51759200
F	2.06039400	-4.01730000	-3.32825800
F	-2.06027900	-4.01736800	3.32824900
F	2.53467800	-3.27753400	3.51756200
C	-0.02239000	-1.71565100	1.40632400
C	0.02244500	-1.71564200	-1.40632800
C	5.25853900	1.27729400	-0.47743800
F	4.70603600	2.00022000	-1.45348300
F	5.80735700	2.13172400	0.39358400
F	6.26163600	0.57380500	-1.01388900

Zero-point correction= 0.516927 (Hartree/Particle)

Thermal correction to Energy= 0.560051

Thermal correction to Enthalpy= 0.560995

Thermal correction to Gibbs Free Energy= 0.435420

sp-E = -2707.29963350 hartree

Thermal correction to Gibbs free energy (ZPG) from GoodVibes: 0.003190 hartree

DIPEA

N	0.04284700	-0.12448800	-0.09647600
C	1.30920700	0.53408900	0.24150200

H	1.33792000	0.77904600	1.32918200
C	-1.12740100	0.74408600	-0.25488800
H	-0.82802800	1.54630700	-0.94390400
C	-0.20816400	-1.33390800	0.68662600
H	-1.20113800	-1.28195000	1.16260100
H	0.49837900	-1.39075900	1.53369300
C	-0.12847700	-2.61896700	-0.13177400
H	0.86179800	-2.73180000	-0.59693500
H	-0.31452100	-3.50143000	0.50093100
H	-0.87717100	-2.61188900	-0.93784800
C	2.49066700	-0.39788400	-0.04208400
H	2.43136400	-1.33837700	0.52290800
H	2.52687600	-0.64598700	-1.11489600
H	3.43631200	0.09151800	0.23516100
C	1.52294200	1.84247500	-0.51926100
H	1.42783300	1.68072400	-1.60519300
H	0.82055400	2.63334900	-0.22207000
H	2.53715000	2.21771800	-0.32003400
C	-1.59986900	1.41346800	1.04395500
H	-2.42359700	2.11514000	0.84195200
H	-1.96596300	0.67306900	1.77289700
H	-0.78532000	1.98058800	1.52031200
C	-2.27176500	0.01127100	-0.95526800
H	-1.92073600	-0.43689100	-1.89649700
H	-2.69940700	-0.78990900	-0.33247300
H	-3.08507300	0.71517300	-1.18729700

Zero-point correction= 0.263014 (Hartree/Particle)

Thermal correction to Energy= 0.274613

Thermal correction to Enthalpy= 0.275557

Thermal correction to Gibbs Free Energy= 0.227163

sp-E = -371.075308480 hartree

Thermal correction to Gibbs free energy (ZPG) from GoodVibes: -0.000427 hartree

DIPEA$^{\bullet+}$

N	0.03132500	-0.05832300	0.18369600
C	1.39499600	0.48117100	0.27073700
H	1.56404700	0.57147700	1.36203800
C	-1.08256200	0.74628500	-0.31283800
H	-0.65132600	1.46623800	-1.01651900
C	-0.23945700	-1.32636300	0.84273500
H	-1.21913200	-1.23826600	1.33338800
H	0.51769300	-1.45651200	1.62708800
C	-0.23984800	-2.54217900	-0.09222100
H	0.71417400	-2.65156200	-0.62043600
H	-0.39403500	-3.43232600	0.53163500
H	-1.04922600	-2.49483500	-0.82849000
C	2.41707300	-0.52890100	-0.27117400
H	2.41881900	-1.47400200	0.28525500
H	2.23640800	-0.73070000	-1.33635200
H	3.41468200	-0.08384100	-0.16685600
C	1.57697700	1.84984300	-0.36682000
H	1.45509600	1.81071600	-1.45918900
H	0.90422600	2.61034700	0.04884300
H	2.60409000	2.17712100	-0.15971100
C	-1.67609300	1.52871800	0.87627400
H	-2.43371300	2.21753800	0.47970300
H	-2.16151300	0.84989100	1.59143700
H	-0.91385800	2.11694900	1.40385100

C	-2.14314200	-0.06285800	-1.05057900
H	-1.71690100	-0.59650800	-1.90997000
H	-2.66652600	-0.77420900	-0.39684600
H	-2.88993600	0.64644700	-1.43142500

Zero-point correction= 0.264456 (Hartree/Particle)

Thermal correction to Energy= 0.276118

Thermal correction to Enthalpy= 0.277062

Thermal correction to Gibbs Free Energy= 0.227586

sp-E = -370.881910376 hartree

Thermal correction to Gibbs free energy (ZPG) from GoodVibes: -0.000214 hartree

TS2a

N	-1.65481100	-0.17929900	0.12596600
C	-0.75829300	-0.94650600	-0.63014100
H	0.47969400	-0.38292600	-0.41407100
C	-2.69642800	0.65014900	-0.50909800
H	-2.27708400	0.98480000	-1.46611600
C	-1.63218200	-0.29144500	1.58125000
H	-1.66156000	0.71978600	2.01182100
H	-0.65900600	-0.70877100	1.86567300
C	-2.75001600	-1.13528500	2.18750600
H	-2.80622500	-2.12686300	1.71763300
H	-2.55234800	-1.27131400	3.26000700
H	-3.72959400	-0.64950400	2.08857900
C	-0.60669100	-2.39675200	-0.15974700
H	-0.47769400	-2.51267600	0.92185500
H	-1.51406000	-2.95286800	-0.44818700
H	0.24688000	-2.86995500	-0.66070600
C	-0.95998200	-0.91944600	-2.13957300

H	-1.95487300	-1.29984900	-2.41402000
H	-0.83723900	0.06932900	-2.59392900
H	-0.23101400	-1.58940600	-2.60892900
C	-3.05470200	1.90219400	0.28472500
H	-3.75414600	2.50034800	-0.31534300
H	-3.55832200	1.66689700	1.23358600
H	-2.17801900	2.52938100	0.49356300
C	-3.95521200	-0.17529200	-0.81811300
H	-3.71650200	-1.12696100	-1.31041700
H	-4.52032100	-0.39875900	0.09646300
H	-4.60929100	0.40085900	-1.48820300
N	1.77713400	0.25493600	-0.17282400
C	1.54032400	1.72585000	-0.39044500
H	2.53940800	2.19382300	-0.41603400
C	2.13317200	-0.06081800	1.25355600
H	1.25386400	0.24014800	1.83776600
C	2.82523400	-0.23197400	-1.11917500
H	3.78110500	-0.27213100	-0.57808300
H	2.96386600	0.52789100	-1.89675100
C	2.56316500	-1.56785500	-1.79018400
H	1.70065100	-1.51697100	-2.46472400
H	3.44022700	-1.83027200	-2.39825100
H	2.40454300	-2.38097300	-1.07140500
C	0.85816700	1.99668100	-1.72991000
H	1.26400700	1.40341400	-2.55958700
H	-0.22112700	1.80728800	-1.66123400
H	0.98708600	3.05649900	-1.98768700
C	0.72844300	2.38493300	0.71918600
H	-0.22252200	1.86091000	0.86735300

H	1.24879900	2.44022100	1.68212800
H	0.49466000	3.41408200	0.41429500
C	3.34275800	0.71926300	1.77948900
H	3.43806000	0.52000800	2.85594200
H	4.27960700	0.39344300	1.30514600
H	3.25590400	1.80512400	1.65259200
C	2.35439500	-1.55047300	1.49011100
H	1.53342400	-2.16543000	1.10955200
H	3.29260000	-1.90364000	1.03856100
H	2.42738900	-1.72377100	2.57254700

Zero-point correction= 0.527755 (Hartree/Particle)

Thermal correction to Energy= 0.551434

Thermal correction to Enthalpy= 0.552379

Thermal correction to Gibbs Free Energy= 0.477781

sp-E = -741.960544221 hartree

Thermal correction to Gibbs free energy (ZPG) from GoodVibes: -0.000632 hartree

Int2a

N	0.05901700	0.07722400	-0.00646000
C	1.26374300	-0.63846900	-0.10988700
C	-1.19487600	-0.64182900	0.22529200
H	-0.95518000	-1.47523900	0.89837700
C	-0.04740700	1.35544800	-0.70185700
H	-1.05153100	1.43981400	-1.14458700
H	0.64226600	1.36866700	-1.56129800
C	0.20552500	2.56871300	0.18823100
H	1.21263900	2.53587600	0.62855600
H	0.11619500	3.49970700	-0.39296400
H	-0.52121500	2.60898900	1.01274300

C	2.54274800	0.14899400	-0.07389700
H	2.54759700	1.01545000	-0.75160200
H	2.76377800	0.54107900	0.94323600
H	3.39011300	-0.49097400	-0.36117600
C	1.32960500	-2.01743200	0.47779200
H	1.20202000	-2.02445600	1.58216300
H	0.57728100	-2.70836200	0.06524700
H	2.31664200	-2.45634700	0.27206100
C	-1.78184600	-1.23960800	-1.05900100
H	-2.66961300	-1.85056100	-0.83540600
H	-2.08787600	-0.45172900	-1.76550100
H	-1.04050200	-1.87809500	-1.56251300
C	-2.21735500	0.21517600	0.97240000
H	-1.78754600	0.60891900	1.90507300
H	-2.57258400	1.06542800	0.37002600
H	-3.09642800	-0.39469200	1.22834600

Zero-point correction= 0.249726 (Hartree/Particle)

Thermal correction to Energy= 0.261643

Thermal correction to Enthalpy= 0.262587

Thermal correction to Gibbs Free Energy= 0.212621

sp-E = -370.422938119 hartree

Thermal correction to Gibbs free energy (ZPG) from GoodVibes: -0.000393 hartree

DIPEA-H^+

N	-0.03270700	0.13659700	-0.14920100
C	-1.36162900	-0.50342600	0.23778600
H	-1.27837300	-0.70515300	1.31534200
C	1.14156800	-0.82781300	-0.25242300
H	0.79509300	-1.60296000	-0.94546100

C	0.27846500	1.35496800	0.69478300
H	1.26257200	1.20380900	1.14910100
H	-0.44597300	1.38169300	1.51654500
C	0.25605100	2.63514300	-0.11497400
H	-0.72816100	2.81510500	-0.57024300
H	0.48018100	3.47998500	0.54951100
H	1.01639100	2.62098200	-0.90894800
C	-2.48802500	0.49198200	-0.01347900
H	-2.37312200	1.42691900	0.54936100
H	-2.57292200	0.72963500	-1.08539100
H	-3.43436700	0.03595900	0.30421000
C	-1.60569800	-1.80436600	-0.51345300
H	-1.54897900	-1.65958500	-1.60332000
H	-0.91874200	-2.60827800	-0.22190300
H	-2.62211100	-2.14602500	-0.27798500
C	1.46798300	-1.46711300	1.08678800
H	2.26906600	-2.20107500	0.92749800
H	1.83188800	-0.73642100	1.82279500
H	0.61121500	-2.00254200	1.51739600
C	2.33064700	-0.13424600	-0.90200200
H	2.05966400	0.32005400	-1.86657700
H	2.77989500	0.63519500	-0.25834900
H	3.10205500	-0.89033500	-1.09928900
H	-0.17250000	0.47608000	-1.10803500

Zero-point correction= 0.279729 (Hartree/Particle)

Thermal correction to Energy= 0.291247

Thermal correction to Enthalpy= 0.292191

Thermal correction to Gibbs Free Energy= 0.244036

sp-E = -371.542439523 hartree

Thermal correction to Gibbs free energy (ZPG) from GoodVibes: -0.000417 hartree

SF_6

F	0.11254200	1.28891800	-0.90881600
S	-0.00007700	0.00011100	0.00001900
F	-1.03527600	-0.62697300	-1.01702800
F	-1.18971400	0.66725500	0.79953800
F	-0.11225800	-1.28869700	0.90828800
F	1.03538000	0.62667400	1.01714100
F	1.18946400	-0.66737400	-0.79915700

Zero-point correction= 0.021680 (Hartree/Particle)

Thermal correction to Energy= 0.027178

Thermal correction to Enthalpy= 0.028122

Thermal correction to Gibbs Free Energy= -0.008015

sp-E = -997.306893240 hartree

Thermal correction to Gibbs free energy (ZPG) from GoodVibes: -0.000030 hartree

Int2b

N	-1.93766700	0.05157500	0.20305100
C	-1.92310500	1.56640500	0.20531200
H	-2.94375800	1.88194200	0.47004900
C	-2.72121700	-0.57067700	-0.93338000
H	-2.28373100	-0.13059300	-1.83698300
C	-2.28181400	-0.53342300	1.54462000
H	-3.09943500	-1.24933500	1.40818900
H	-2.67995900	0.26968500	2.17609900
C	-1.08615000	-1.21335100	2.18503200
H	-0.24032400	-0.52366300	2.30306000
H	-1.37172900	-1.59538800	3.17443800

H	-0.74058000	-2.05811600	1.57323400
C	-0.94368500	2.07803800	1.25687100
H	-1.19591700	1.75799700	2.27590500
H	0.07974300	1.75329100	1.02751700
H	-0.96615000	3.17581700	1.24646600
C	-1.54153000	2.10363200	-1.16961500
H	-0.59640200	1.64789600	-1.50056200
H	-2.31465900	1.93824600	-1.93072700
H	-1.38741100	3.18785000	-1.09176900
C	-4.19995300	-0.22420700	-0.87668100
H	-4.70130900	-0.68817300	-1.73690200
H	-4.68144800	-0.60815600	0.03471800
H	-4.37727000	0.85860500	-0.93546700
C	-2.44256100	-2.06762400	-0.99932000
H	-1.35883000	-2.24789200	-1.00491800
H	-2.90156200	-2.62599200	-0.17077300
H	-2.86287200	-2.46019600	-1.93515900
H	-0.94150500	-0.22494800	-0.02171600
F	0.38334300	-0.70363900	-0.66360300
S	2.29022100	-0.12430700	-0.16489800
F	2.65622000	-1.67210900	0.31580700
F	1.87820600	0.27123600	1.44170100
F	1.99027800	1.47277100	-0.63138200
F	2.77663100	-0.48193700	-1.70501400
F	3.79591400	0.30659500	0.17752400

Zero-point correction= 0.295731 (Hartree/Particle)

Thermal correction to Energy= 0.316652

Thermal correction to Enthalpy= 0.317596

Thermal correction to Gibbs Free Energy= 0.245361

sp-E = -1368.99677421 hartree

Thermal correction to Gibbs free energy (ZPG) from GoodVibes: 0.000198 hartree

DIPEA-HF

N	-0.02533300	0.02661000	-0.15695500
C	1.08320900	-0.90375700	-0.50082800
H	0.88260800	-1.35181100	-1.49427900
C	-1.29780400	-0.62769600	0.24200100
H	-1.03232300	-1.31654500	1.05508100
C	-0.20998900	1.08534300	-1.17535600
H	-1.25943900	1.10305100	-1.50097300
H	0.36476300	0.83088900	-2.07963500
C	0.17712000	2.47074400	-0.67706100
H	1.23227600	2.50824300	-0.37212700
H	0.02012800	3.21755800	-1.46954400
H	-0.43121100	2.75765700	0.19237700
C	2.40343600	-0.13436700	-0.57982200
H	2.39345100	0.64930600	-1.34938200
H	2.61856800	0.33584800	0.39137300
H	3.22230700	-0.82484500	-0.82593300
C	1.22033100	-2.03119700	0.52110800
H	1.28965300	-1.61062400	1.53604500
H	0.38754700	-2.74640900	0.48931100
H	2.14181000	-2.59438200	0.31824400
C	-1.95356400	-1.44613900	-0.87001200
H	-2.85029600	-1.95099600	-0.48198100
H	-2.26841300	-0.81332800	-1.71403400
H	-1.27712600	-2.22128500	-1.25947900
C	-2.25615100	0.39053300	0.85733500

H	-1.74775900	0.95000000	1.65485400
H	-2.64430500	1.10683200	0.11745900
H	-3.11921900	-0.13213700	1.29401200
H	0.44918000	0.62306700	1.16395900
F	0.75617700	0.94142800	2.05879400

Zero-point correction= 0.277065 (Hartree/Particle)

Thermal correction to Energy= 0.290439

Thermal correction to Enthalpy= 0.291383

Thermal correction to Gibbs Free Energy= 0.238836

sp-E = -471.573641347 hartree

Thermal correction to Gibbs free energy (ZPG) from GoodVibes: -0.000619 hartree

$SF_5^{\bullet}$

F	-0.00062900	-0.00043100	1.36141700
S	0.00021800	0.00012300	-0.20928300
F	-1.25908900	1.02491900	-0.24742000
F	-1.02497700	-1.25882700	-0.24772200
F	1.25911300	-1.02524600	-0.24721000
F	1.02519500	1.25936500	-0.24700700

Zero-point correction= 0.015330 (Hartree/Particle)

Thermal correction to Energy= 0.020805

Thermal correction to Enthalpy= 0.021749

Thermal correction to Gibbs Free Energy= -0.014717

sp-E = -897.403838381 hartree

Thermal correction to Gibbs free energy (ZPG) from GoodVibes: -0.000044 hartree

TCP

C	-0.00005100	0.00005000	0.77149200
C	0.00002100	0.00005500	-0.77140900

C	0.08430400	-1.29786200	-0.00002100
H	-0.79860900	-1.94343400	-0.00006100
H	1.04327800	-1.82379000	0.00002500
C	-1.16625600	0.57591700	-0.00007300
C	1.08197700	0.72189700	0.00002900
H	2.08249900	0.27998400	0.00007800
H	1.05806000	1.81536300	0.00003400
H	-2.10111800	0.00823600	-0.00011500
H	-1.28407900	1.66330200	-0.00007300

Zero-point correction= 0.093803 (Hartree/Particle)

Thermal correction to Energy= 0.097740

Thermal correction to Enthalpy= 0.098684

Thermal correction to Gibbs Free Energy= 0.067519

sp-E = -194.018724779 hartree

Thermal correction to Gibbs free energy (ZPG) from GoodVibes: -0.000007 hartree

Int2c

C	-1.02436100	0.01511400	-0.00293100
C	-2.75596000	-0.00234800	0.00026100
C	-1.88383700	-0.79785100	-0.98501000
H	-1.85656000	-1.88715100	-0.86762200
H	-1.87435300	-0.46599300	-2.02912400
C	-1.88349500	-0.44502700	1.18639100
C	-1.89959500	1.26024100	-0.20484400
H	-1.88969500	1.70477200	-1.20658800
H	-1.88958700	1.99852000	0.60513500
H	-1.85624000	-1.51544300	1.41995300
H	-1.87364200	0.20051900	2.07155000
S	0.84011000	0.00282400	-0.00055400

F	0.85239800	-1.59698400	0.27434000
F	0.90163500	0.28299600	1.59545700
F	0.94836900	1.59598300	-0.27463300
F	0.90405300	-0.26631300	-1.59825300
F	2.44708000	-0.04470200	0.00890600

Zero-point correction= 0.112887 (Hartree/Particle)

Thermal correction to Energy= 0.121536

Thermal correction to Enthalpy= 0.122481

Thermal correction to Gibbs Free Energy= 0.078829

sp-E = -1091.45163346 hartree

Thermal correction to Gibbs free energy (ZPG) from GoodVibes: -0.000176 hartree

5b

C	2.41514400	-1.33492300	0.35943500
C	1.07086900	-0.98594100	0.47340800
C	0.63919000	0.30783300	0.14300300
C	1.58741100	1.23403200	-0.31601500
C	2.93144100	0.88442200	-0.42586800
C	3.35053300	-0.40205200	-0.08753300
H	2.73297800	-2.34496600	0.62669000
H	0.35622900	-1.72622200	0.83463300
H	1.26476700	2.23599900	-0.60740900
H	3.65301900	1.61965600	-0.78829600
H	4.40310600	-0.67880800	-0.17738000
C	-0.78453400	0.72246700	0.27822700
C	-1.84108800	-0.27095400	-0.14903300
F	-1.94427500	-1.29555000	0.71624700
F	-3.05532500	0.27535700	-0.23811400
F	-1.55682900	-0.80374700	-1.34354300

C	-1.16310000	1.92087300	0.73187000
H	-2.21432200	2.20713600	0.78348400
H	-0.42311000	2.64812500	1.07201400

Zero-point correction= 0.140268 (Hartree/Particle)

Thermal correction to Energy= 0.150081

Thermal correction to Enthalpy= 0.151025

Thermal correction to Gibbs Free Energy= 0.103757

sp-E = -646.754789385 hartree

Thermal correction to Gibbs free energy (ZPG) from GoodVibes: 0.000113 hartree

Int2c'

C	3.94556900	-0.62347600	0.48464900
C	2.88270800	0.24054000	0.27227400
C	1.65129800	-0.22980200	-0.26416700
C	1.55924400	-1.61772000	-0.55965400
C	2.62879000	-2.47160600	-0.34374700
C	3.83076200	-1.98249600	0.17691600
H	4.87831200	-0.23344100	0.89627300
H	3.01066100	1.29217600	0.52312400
H	0.63046400	-2.03914100	-0.94252800
H	2.52471300	-3.53239200	-0.57887400
H	4.67184600	-2.65751700	0.34595700
C	0.53770300	0.63917700	-0.49824400
C	0.61689400	2.09389300	-0.10251400
F	1.63989300	2.72238100	-0.70269000
F	-0.48749500	2.76823900	-0.42902900
F	0.79303200	2.24434600	1.21795000
C	-0.72568100	0.17564400	-1.10912300
H	-1.28446800	0.99756900	-1.56439800

H	-0.57942900	-0.61099400	-1.85534300
S	-1.98543800	-0.55226400	0.06334700
F	-2.19261600	0.87238900	0.79788200
F	-0.89869000	-0.96864700	1.18202100
F	-3.11637500	-1.14994500	1.03818700
F	-3.17174200	-0.20365500	-0.97986100
F	-1.86031900	-2.03232600	-0.58847200

Zero-point correction= 0.159592 (Hartree/Particle)

Thermal correction to Energy= 0.175209

Thermal correction to Enthalpy= 0.176153

Thermal correction to Gibbs Free Energy= 0.114606

sp-E = -1544.18675857 hartree

Thermal correction to Gibbs free energy (ZPG) from GoodVibes: 0.000308 hartree

TS2b

C	-1.85529300	-0.05746700	0.52609100
C	-0.33305500	-0.39064000	1.31107400
C	-1.12374900	-1.40712000	0.47021700
H	-0.70003200	-1.69004800	-0.49987900
H	-1.59290900	-2.24147100	1.00379300
C	-0.54271000	0.69004700	0.23858300
C	-1.61937700	0.04543500	2.04099600
H	-2.12190400	-0.70365100	2.66381800
H	-1.63556800	1.05446700	2.46910800
H	-0.08099600	0.51335200	-0.73994800
H	-0.49727100	1.73645700	0.56101100
S	-3.45920100	0.29506100	-0.33823100
F	-2.78271200	-0.02832400	-1.77789100
F	-3.09321700	1.86516800	-0.51272400

F	-4.24072700	0.64047100	1.03828600
F	-3.94174600	-1.24774600	-0.21310000
F	-4.83226700	0.59391900	-1.12088300
C	4.20463000	2.98985400	-0.44678900
C	4.65506100	1.82526200	-1.06692900
C	4.13149700	0.58541700	-0.70723400
C	3.14195000	0.48261400	0.28571300
C	2.69368300	1.66661200	0.89682900
C	3.22011000	2.90394700	0.53773300
H	4.61507600	3.96056200	-0.73250600
H	5.42578300	1.87834900	-1.83876800
H	4.51061200	-0.30921600	-1.20104600
H	1.90590200	1.62550800	1.65093200
H	2.84988900	3.80844500	1.02490400
C	2.56658200	-0.82100700	0.69387500
C	1.98230400	-1.03878900	1.89242700
H	1.58132500	-2.01653900	2.15598800
H	2.03848200	-0.29459700	2.68730900
C	2.51125000	-1.92306500	-0.33510400
F	1.96086300	-1.49229100	-1.48280000
F	1.78594900	-2.96890700	0.07114500
F	3.72735800	-2.40109000	-0.64846600

Zero-point correction= 0.254021 (Hartree/Particle)

Thermal correction to Energy= 0.274548

Thermal correction to Enthalpy= 0.275492

Thermal correction to Gibbs Free Energy= 0.200045

sp-E = -1738.21026662 hartree

Thermal correction to Gibbs free energy (ZPG) from GoodVibes: 0.001778 hartree

Int2d

C	-1.80055700	-0.06448100	0.39763600
C	-0.14458000	-0.44119100	1.07968100
C	-0.88495200	-1.21165900	-0.05336000
H	-0.45906400	-1.13174900	-1.06228100
H	-1.21927300	-2.22997700	0.18326800
C	-0.59032000	0.87978500	0.38383400
C	-1.49155200	-0.41576500	1.85981800
H	-1.86980500	-1.38128800	2.22172100
H	-1.62005500	0.38992100	2.59492600
H	-0.14613000	1.09518400	-0.59738100
H	-0.66116200	1.77312900	1.01849900
S	-3.43034100	0.30362200	-0.30312900
F	-2.81352000	0.35633100	-1.79812100
F	-3.22097300	1.89984900	-0.15916200
F	-4.16571300	0.28302900	1.13511200
F	-3.77030700	-1.26621000	-0.49000400
F	-4.86753000	0.62134300	-0.95849000
C	4.30015300	2.93471900	-0.41634800
C	4.62056200	1.74944400	-1.08560700
C	3.97939400	0.56138300	-0.76866600
C	2.97875600	0.51093200	0.24325300
C	2.67671100	1.73025100	0.91032400
C	3.32337600	2.91305300	0.58320800
H	4.80795100	3.86680700	-0.67164200
H	5.38369700	1.75301900	-1.86664000
H	4.26032400	-0.34034100	-1.31096900
H	1.91790000	1.75270500	1.69219700
H	3.06250800	3.83115200	1.11360800

C	2.29143300	-0.69779400	0.58764900
C	1.22589700	-0.76137400	1.64183000
H	1.18893100	-1.76569700	2.08517500
H	1.44775600	-0.06483000	2.46146800
C	2.52527100	-1.94414400	-0.21976600
F	2.25965500	-1.76446300	-1.52797400
F	1.75296000	-2.95912400	0.18473800
F	3.79590600	-2.37797000	-0.14974200

Zero-point correction= 0.257835 (Hartree/Particle)

Thermal correction to Energy= 0.277736

Thermal correction to Enthalpy= 0.278680

Thermal correction to Gibbs Free Energy= 0.205981

sp-E = -1738.28236385 hartree

Thermal correction to Gibbs free energy (ZPG) from GoodVibes: 0.001156 hartree

Int2e

N	-0.07605300	0.01235300	0.13990000
C	-1.20664400	-0.59875500	-0.04145500
C	1.23358500	-0.64434700	-0.14715100
H	1.00255600	-1.57387500	-0.67355700
C	-0.00445200	1.38804600	0.68505300
H	1.01153300	1.52336600	1.07075100
H	-0.66955200	1.44841900	1.55484900
C	-0.32623300	2.45959500	-0.34537900
H	-1.34481200	2.35522400	-0.74333300
H	-0.24761400	3.44477100	0.13383200
H	0.37699500	2.43176500	-1.18754700
C	-2.51858900	0.02336900	0.28652000
H	-2.47384800	1.00956300	0.75277400

H	-3.10227300	0.09007500	-0.64514200
H	-3.06468200	-0.66790900	0.94571500
C	-1.26917900	-1.98820900	-0.59438400
H	-0.80950200	-2.03456200	-1.59186200
H	-0.73975600	-2.69524700	0.06066400
H	-2.31217700	-2.31000100	-0.67901700
C	1.93098600	-1.00418800	1.16144400
H	2.83396200	-1.58554600	0.93067600
H	2.24268900	-0.11311500	1.72516000
H	1.28176200	-1.61700100	1.80230400
C	2.09021200	0.20055100	-1.08197400
H	1.55580500	0.43081800	-2.01414400
H	2.42574500	1.13862300	-0.61739100
H	2.98743800	-0.37821000	-1.34007400

Zero-point correction= 0.253368 (Hartree/Particle)

Thermal correction to Energy= 0.265167

Thermal correction to Enthalpy= 0.266111

Thermal correction to Gibbs Free Energy= 0.216656

sp-E = -370.326414031 hartree

Thermal correction to Gibbs free energy (ZPG) from GoodVibes: -0.000245 hartree

Int2f

C	-1.79000100	-0.06703700	0.41399800
C	-0.14014300	-0.44352100	1.11594700
C	-0.89908100	-1.25033900	0.01966000
H	-0.45948600	-1.22924600	-0.98567200
H	-1.25508100	-2.24961100	0.30434900
C	-0.56289400	0.85059400	0.35625400
C	-1.49531700	-0.35114600	1.89040400

H	-1.89812400	-1.28890900	2.29802800
H	-1.61274900	0.49276000	2.58437700
H	-0.10873800	1.00677600	-0.63107500
H	-0.61548000	1.77543000	0.94616100
S	-3.41532900	0.30207500	-0.30981200
F	-2.78714300	0.33331900	-1.80371900
F	-3.20174800	1.90259500	-0.18311600
F	-4.17490000	0.30387400	1.12008100
F	-3.77296700	-1.26862000	-0.48530700
F	-4.84590500	0.62391400	-0.98597600
C	4.26264600	2.98857000	-0.42514100
C	4.56273900	1.79941500	-1.10214300
C	3.94850700	0.59773600	-0.77877800
C	2.96688400	0.49000800	0.26302700
C	2.67793700	1.72706500	0.92636700
C	3.30830200	2.92105200	0.59438000
H	4.75063700	3.93010300	-0.68644300
H	5.30237800	1.80830600	-1.90972700
H	4.23174100	-0.29339900	-1.34016500
H	1.92903800	1.74905600	1.72032500
H	3.03761900	3.82774300	1.14559200
C	2.33104800	-0.73271800	0.62394700
C	1.23447600	-0.77704700	1.65884400
H	1.16544000	-1.77402500	2.12178700
H	1.44951500	-0.08426600	2.48963300
C	2.52373800	-1.91948300	-0.19361700
F	2.16498200	-1.80734600	-1.53047100
F	1.81655400	-2.98797000	0.24446400
F	3.81840500	-2.37785600	-0.29585200

Zero-point correction= 0.255625 (Hartree/Particle)

Thermal correction to Energy= 0.275640

Thermal correction to Enthalpy= 0.276585

Thermal correction to Gibbs Free Energy= 0.204497

sp-E = -1738.40662543 hartree

Thermal correction to Gibbs free energy (ZPG) from GoodVibes: 0.001070 hartree

TS2c

C	3.43896900	-0.49649800	-0.22429000
C	1.64754100	-0.25113700	-0.52225800
C	2.31298500	-0.86223200	0.74812200
H	2.24582400	-0.26780300	1.66899500
H	2.14403200	-1.93288600	0.92504400
C	2.75039800	0.84962400	-0.48615800
C	2.62993200	-1.11396400	-1.37264500
H	2.48551200	-2.20227800	-1.33890100
H	2.85988300	-0.75111900	-2.38395800
H	2.71008400	1.55497900	0.35426300
H	2.99211300	1.34205300	-1.43826700
S	5.21860800	-0.71790300	0.04949600
F	5.33023500	0.67322700	0.86903300
F	5.56606100	0.05786500	-1.32985900
F	5.24946700	-2.13170100	-0.73710600
F	5.01299200	-1.51226500	1.44281600
F	6.80759000	-0.88878500	0.26825600
C	-1.21352200	4.98809900	-0.63416700
C	-1.94365100	4.34700600	0.36909600
C	-1.71386900	3.00999100	0.67957200
C	-0.72840600	2.24783800	0.00522900

C	-0.01029200	2.91707200	-1.01182600
C	-0.24726800	4.25478800	-1.32253800
H	-1.39790800	6.03681200	-0.87686400
H	-2.71738500	4.89378800	0.91507500
H	-2.32323300	2.53366700	1.44666500
H	0.76058800	2.38417300	-1.57077000
H	0.33791600	4.72942100	-2.11474600
C	-0.49361600	0.83431200	0.28524200
C	0.16484700	-0.05990400	-0.74050200
H	-0.29874600	-1.06026100	-0.73418300
H	0.00818200	0.33692700	-1.75697100
C	-1.03010700	0.22186100	1.38951100
F	-1.24007000	0.87077000	2.52820900
F	-0.67667200	-1.02851300	1.66770500
F	-2.79367800	-0.17499000	1.18318500
N	-4.87913600	-1.15129100	-0.53020400
C	-4.37059200	0.04803400	-0.58709700
C	-4.26639900	-2.27090700	-1.30191800
H	-3.88209500	-1.81189700	-2.21917700
C	-5.93545300	-1.47217100	0.44499800
H	-6.57809800	-2.24159400	0.00510000
H	-6.56864200	-0.58700900	0.56085600
C	-5.36697000	-1.91815800	1.78875100
H	-4.52367900	-1.27046800	2.06801400
H	-6.15234200	-1.86998400	2.55564600
H	-5.00048900	-2.95290900	1.74491400
C	-4.97826500	1.17747000	0.18076600
H	-5.17141300	0.91266900	1.22470800
H	-4.30634800	2.04041500	0.16002000

H	-5.93022100	1.46496600	-0.29607200
C	-3.30370500	0.38741700	-1.57680500
H	-2.49356700	-0.34772400	-1.58475100
H	-3.75189200	0.43347900	-2.58338800
H	-2.87264900	1.36434300	-1.33947700
C	-5.28617200	-3.31413400	-1.73661800
H	-4.79215000	-3.99732600	-2.44152700
H	-5.65245000	-3.92226400	-0.89679300
H	-6.14288800	-2.85826100	-2.25363000
C	-3.09377600	-2.87505700	-0.52667100
H	-2.50734000	-2.08632000	-0.03679800
H	-3.44880900	-3.56242300	0.25427000
H	-2.45455300	-3.44626200	-1.21473600

Zero-point correction= 0.510528 (Hartree/Particle)

Thermal correction to Energy= 0.543713

Thermal correction to Enthalpy= 0.544657

Thermal correction to Gibbs Free Energy= 0.442872

sp-E = -2108.74282460 hartree

Thermal correction to Gibbs free energy (ZPG) from GoodVibes: 0.001987 hartree

10

N	-0.14019600	0.17348500	0.14962200
C	1.23054800	-0.20115200	0.11741600
C	-1.16073800	-0.86825100	0.32366000
H	-0.79589800	-1.53495100	1.11590000
C	-0.51792100	1.28618700	-0.72330500
H	-1.40871200	1.00453500	-1.30593700
H	0.26321200	1.44475800	-1.48226300
C	-0.80080900	2.58822700	0.01861500

H	0.08190500	2.92653200	0.58050900
H	-1.08087000	3.38424700	-0.68884300
H	-1.62565300	2.46158500	0.73467300
C	2.16574900	0.97395700	0.38490800
H	2.00131500	1.79893300	-0.31953500
H	2.01228800	1.34922900	1.40619000
H	3.20857900	0.64534500	0.28198100
C	1.57833400	-1.36830600	1.03190100
H	1.28261500	-1.15260300	2.06801800
H	1.09651800	-2.29876200	0.70636000
H	2.66400400	-1.52901400	1.00649500
C	-1.39153100	-1.72292900	-0.92870400
H	-2.11061600	-2.52970600	-0.71986700
H	-1.79896000	-1.11970400	-1.75563000
H	-0.45016500	-2.17212600	-1.27376000
C	-2.46894100	-0.28307800	0.85523300
H	-2.29030700	0.30111500	1.76979200
H	-2.96504700	0.36922400	0.12005500
H	-3.16911200	-1.09662100	1.09590500
F	1.58423600	-0.65492800	-1.20730500

Zero-point correction= 0.255539 (Hartree/Particle)

Thermal correction to Energy= 0.267759

Thermal correction to Enthalpy= 0.268703

Thermal correction to Gibbs Free Energy= 0.219064

sp-E = -470.348491474 hartree

Thermal correction to Gibbs free energy (ZPG) from GoodVibes: -0.000442 hartree

6b

C	1.73660000	0.05250800	0.34211800

C	0.06014200	0.41743400	0.98682900
C	0.90877800	1.30160200	0.02142500
H	0.54656800	1.39447600	-1.01133700
H	1.27870700	2.25366800	0.42443500
C	0.48547100	-0.81043700	0.12547500
C	1.35554000	0.19882800	1.82238700
H	1.75608600	1.07681600	2.34608800
H	1.40064900	-0.71335400	2.43261700
H	0.09013400	-0.85509700	-0.89813600
H	0.48221700	-1.79096600	0.62126700
S	3.39090600	-0.33715400	-0.28577900
F	2.79304000	-1.01997400	-1.62414600
F	3.37366300	-1.77256800	0.46211000
F	4.11694100	0.32573000	0.99741500
F	3.53356800	1.06432100	-1.07621200
F	4.85858800	-0.71716200	-0.83202200
C	-5.03116900	-2.39612000	-0.37176600
C	-5.45995000	-1.08371100	-0.56982800
C	-4.60760300	-0.01453500	-0.30459900
C	-3.29866200	-0.23184000	0.15878100
C	-2.88829000	-1.55801400	0.37028100
C	-3.74149000	-2.62715100	0.10396700
H	-5.70118800	-3.23304600	-0.57940100
H	-6.47314200	-0.88630100	-0.92689100
H	-4.97365100	1.00229700	-0.44888100
H	-1.88581500	-1.76811400	0.74470800
H	-3.39214800	-3.64829200	0.27131400
C	-2.37069700	0.88907400	0.45817200
C	-1.31117900	0.74420700	1.53300700

H	-1.23045100	1.67696000	2.11114800
H	-1.61320900	-0.03791700	2.24358100
C	-2.40242500	2.03596300	-0.22398300
F	-3.19689500	2.33256700	-1.22628900
F	-1.60664500	3.05558400	0.02451500

Zero-point correction= 0.255431 (Hartree/Particle)

Thermal correction to Energy= 0.274396

Thermal correction to Enthalpy= 0.275340

Thermal correction to Gibbs Free Energy= 0.205644

sp-E = -1638.42155290 hartree

Thermal correction to Gibbs free energy (ZPG) from GoodVibes: 0.000953 hartree

In reaction of 1a:

PTZ

C	1.74519700	3.68022400	0.29505400
C	2.42784200	2.55107200	-0.15415800
C	1.75267500	1.34820200	-0.35868300
C	0.38017600	1.23160500	-0.06799400
C	-0.29254900	2.37634600	0.38570900
C	0.38053900	3.58567300	0.55208800
C	0.37983600	-1.23172000	-0.06810000
C	1.75230400	-1.34867000	-0.35879800
C	2.42714000	-2.55174300	-0.15437600
H	3.49633100	-2.60341900	-0.37295600
C	1.74418300	-3.68074700	0.29473400
C	0.37955100	-3.58584400	0.55177500
C	-0.29320500	-2.37631700	0.38550000
H	2.27706900	4.62276200	0.43538300
H	3.49704700	2.60247400	-0.37273500

H	-1.35861600	2.32752800	0.60373100
H	-0.17682500	4.45885700	0.89739000
H	2.27579700	-4.62344300	0.43498200
H	-0.17805300	-4.45890600	0.89699700
H	-1.35925800	-2.32722100	0.60352000
S	2.61367400	-0.00031900	-1.11895900
N	-0.28749600	0.00004200	-0.23639600
C	-1.71969700	0.00024200	-0.22270600
C	-2.43030900	0.00031800	0.98137100
C	-2.40433400	0.00035900	-1.43792800
C	-3.82337800	0.00050800	0.96540200
H	-1.88431200	0.00022700	1.92732500
C	-3.79912500	0.00054900	-1.45103500
H	-1.83291200	0.00029800	-2.36806300
C	-4.50909100	0.00062200	-0.25071500
H	-4.37625900	0.00056500	1.90695400
H	-4.33235100	0.00063800	-2.40389300
H	-5.60101300	0.00076800	-0.26152100

Zero-point correction= 0.261690 (Hartree/Particle)

Thermal correction to Energy= 0.275506

Thermal correction to Enthalpy= 0.276418

Thermal correction to Gibbs Free Energy= 0.220543

sp-E = -1146.71001154 hartree

Thermal correction to Gibbs free energy (ZPG) from GoodVibes: 0.000149 hartree

PTZ*

C	1.74519700	3.68022400	0.29505400
C	2.42784200	2.55107200	-0.15415800
C	1.75267500	1.34820200	-0.35868300

C	0.38017600	1.23160500	-0.06799400
C	-0.29254900	2.37634600	0.38570900
C	0.38053900	3.58567300	0.55208800
C	0.37983600	-1.23172000	-0.06810000
C	1.75230400	-1.34867000	-0.35879800
C	2.42714000	-2.55174300	-0.15437600
H	3.49633100	-2.60341900	-0.37295600
C	1.74418300	-3.68074700	0.29473400
C	0.37955100	-3.58584400	0.55177500
C	-0.29320500	-2.37631700	0.38550000
H	2.27706900	4.62276200	0.43538300
H	3.49704700	2.60247400	-0.37273500
H	-1.35861600	2.32752800	0.60373100
H	-0.17682500	4.45885700	0.89739000
H	2.27579700	-4.62344300	0.43498200
H	-0.17805300	-4.45890600	0.89699700
H	-1.35925800	-2.32722100	0.60352000
S	2.61367400	-0.00031900	-1.11895900
N	-0.28749600	0.00004200	-0.23639600
C	-1.71969700	0.00024200	-0.22270600
C	-2.43030900	0.00031800	0.98137100
C	-2.40433400	0.00035900	-1.43792800
C	-3.82337800	0.00050800	0.96540200
H	-1.88431200	0.00022700	1.92732500
C	-3.79912500	0.00054900	-1.45103500
H	-1.83291200	0.00029800	-2.36806300
C	-4.50909100	0.00062200	-0.25071500
H	-4.37625900	0.00056500	1.90695400
H	-4.33235100	0.00063800	-2.40389300

H	-5.60101300	0.00076800	-0.26152100

sp-E = -1146.56702448 hartree

SF_6

S	0.00000000	-0.00000300	0.00000300
F	-0.74932700	-0.06681500	1.39040800
F	1.08817700	-1.01297700	0.53769300
F	-0.86823000	-1.21192400	-0.52608600
F	0.74932200	0.06681600	-1.39040300
F	0.86822300	1.21192700	0.52608200
F	-1.08816500	1.01297800	-0.53769900

Zero-point correction= 0.021700 (Hartree/Particle)

Thermal correction to Energy= 0.026864

Thermal correction to Enthalpy= 0.027777

Thermal correction to Gibbs Free Energy= -0.006787

sp-E = -997.306894883 hartree

Thermal correction to Gibbs free energy (ZPG) from GoodVibes: -0.000029 hartree

$SF_6^{\bullet-}$

S	-0.00000500	0.00000100	0.00000000
F	-0.48367500	1.58309400	-0.46326400
F	-1.32542400	-0.08539900	1.09132500
F	-0.98232900	-0.66424900	-1.24438400
F	0.48367100	-1.58309100	0.46325900
F	0.98233500	0.66425200	1.24437200
F	1.32543100	0.08539000	-1.09130600

Zero-point correction= 0.012071 (Hartree/Particle)

Thermal correction to Energy= 0.018198

Thermal correction to Enthalpy= 0.019110

Thermal correction to Gibbs Free Energy= -0.018150

sp-E = -997.425480054 hartree

Thermal correction to Gibbs free energy (ZPG) from GoodVibes: -0.000086 hartree

PTZ$^{•+}$

C	1.66336300	3.76419900	0.02617200
C	2.43300600	2.62074100	0.02913700
C	1.82252600	1.35225500	0.02504700
C	0.41028400	1.23177900	0.01802400
C	-0.35410700	2.42367600	0.01490700
C	0.26138700	3.65659500	0.01891000
C	0.41001900	-1.23223300	0.01792900
C	1.82226600	-1.35294100	0.02494000
C	2.43257600	-2.62151100	0.02891600
H	3.52238400	-2.68648000	0.03449200
C	1.66279900	-3.76487000	0.02585500
C	0.26084500	-3.65708200	0.01860400
C	-0.35449800	-2.42408800	0.01470600
H	2.14163200	4.74422300	0.02944000
H	3.52282400	2.68557000	0.03472600
H	-1.44039300	2.36744600	0.00940800
H	-0.35413600	4.55715500	0.01644900
H	2.14094500	-4.74495500	0.02903500
H	-0.35478400	-4.55756800	0.01606400
H	-1.44076500	-2.36780900	0.00920600
S	2.89564000	-0.00044200	0.02850900
N	-0.22788900	-0.00013300	0.01410900
C	-1.67442900	0.00024100	0.00624900
C	-2.35894700	0.00048100	1.21904700

C	-2.34588600	0.00051100	-1.21383600
C	-3.75219900	0.00084100	1.20520500
H	-1.80339600	0.00032700	2.15850700
C	-3.73921700	0.00087200	-1.21493700
H	-1.78030400	0.00037700	-2.14727700
C	-4.44033800	0.00102000	-0.00859900
H	-4.30098300	0.00098200	2.14840700
H	-4.27781700	0.00103600	-2.16399600
H	-5.53183400	0.00131000	-0.01445900

Zero-point correction= 0.262904 (Hartree/Particle)

Thermal correction to Energy= 0.276579

Thermal correction to Enthalpy= 0.277492

Thermal correction to Gibbs Free Energy= 0.221858

sp-E = -1146.52388514 hartree

Thermal correction to Gibbs free energy (ZPG) from GoodVibes: -0.000221 hartree

PTZ$^{\bullet+}$*

C	1.66336300	3.76419900	0.02617200
C	2.43300600	2.62074100	0.02913700
C	1.82252600	1.35225500	0.02504700
C	0.41028400	1.23177900	0.01802400
C	-0.35410700	2.42367600	0.01490700
C	0.26138700	3.65659500	0.01891000
C	0.41001900	-1.23223300	0.01792900
C	1.82226600	-1.35294100	0.02494000
C	2.43257600	-2.62151100	0.02891600
H	3.52238400	-2.68648000	0.03449200
C	1.66279900	-3.76487000	0.02585500
C	0.26084500	-3.65708200	0.01860400

C	-0.35449800	-2.42408800	0.01470600
H	2.14163200	4.74422300	0.02944000
H	3.52282400	2.68557000	0.03472600
H	-1.44039300	2.36744600	0.00940800
H	-0.35413600	4.55715500	0.01644900
H	2.14094500	-4.74495500	0.02903500
H	-0.35478400	-4.55756800	0.01606400
H	-1.44076500	-2.36780900	0.00920600
S	2.89564000	-0.00044200	0.02850900
N	-0.22788900	-0.00013300	0.01410900
C	-1.67442900	0.00024100	0.00624900
C	-2.35894700	0.00048100	1.21904700
C	-2.34588600	0.00051100	-1.21383600
C	-3.75219900	0.00084100	1.20520500
H	-1.80339600	0.00032700	2.15850700
C	-3.73921700	0.00087200	-1.21493700
H	-1.78030400	0.00037700	-2.14727700
C	-4.44033800	0.00102000	-0.00859900
H	-4.30098300	0.00098200	2.14840700
H	-4.27781700	0.00103600	-2.16399600
H	-5.53183400	0.00131000	-0.01445900

sp-E = -1146.45937073 hartree

1a

C	0.10535200	1.02577000	0.19188700
C	0.25363900	2.35464700	0.27235900
H	-0.61372800	3.01030000	0.18079000
H	1.23681300	2.81026700	0.39105000
C	-1.22594300	0.37556500	0.03805000

C	-1.40149700	-0.66777500	-0.88277800
C	-2.32856800	0.80971700	0.78469600
C	-2.65239500	-1.25399900	-1.06001500
H	-0.54949000	-1.01229600	-1.47445100
C	-3.58101500	0.22340100	0.60660300
H	-2.19775700	1.60578300	1.52133800
C	-3.74660500	-0.80969400	-0.31566400
H	-2.77502200	-2.06030900	-1.78642900
H	-4.43055300	0.57058500	1.19874700
H	-4.72669100	-1.27188700	-0.45198800
N	1.21709700	0.15279600	0.20612100
C	1.22912700	-1.07216900	1.00666700
C	2.36112300	0.31927000	-0.54206800
C	2.71097800	-1.45977000	1.03196700
H	0.81939100	-0.87729100	2.00792700
C	3.25084600	-0.88319400	-0.27371000
H	2.85217000	-2.54377400	1.12608100
H	3.12715900	-1.58223900	-1.11775200
O	2.59548900	1.24079100	-1.29577700
H	0.61152900	-1.85611800	0.53762000
H	3.20682600	-0.97840400	1.88801000
H	4.30551400	-0.58113900	-0.24554100

Zero-point correction= 0.227189 (Hartree/Particle)

Thermal correction to Energy= 0.238245

Thermal correction to Enthalpy= 0.239158

Thermal correction to Gibbs Free Energy= 0.190110

sp-E = -595.115703391 hartree

Thermal correction to Gibbs free energy (ZPG) from GoodVibes: -0.000136 hartree

1a$^{\bullet+}$

C	0.14193200	0.89213000	-0.05754600
C	0.31940800	2.29170200	-0.06021800
H	-0.57079800	2.92028900	-0.09239800
H	1.30251400	2.75481800	-0.03408400
C	-1.23144400	0.35091600	-0.04891700
C	-1.61656500	-0.63482100	-0.97238400
C	-2.17364500	0.86567300	0.85584300
C	-2.92568800	-1.10191000	-0.98114100
H	-0.90337500	-1.01224300	-1.70702900
C	-3.47440500	0.37369900	0.85743900
H	-1.88253500	1.63242600	1.57680800
C	-3.85152700	-0.60667000	-0.06124800
H	-3.22569300	-1.85489200	-1.71152200
H	-4.19798500	0.76099700	1.57639500
H	-4.87615800	-0.98325700	-0.06476900
N	1.18121900	0.04438400	-0.03778300
C	1.08164500	-1.39371200	0.27624800
C	2.58571500	0.39798900	-0.24470800
C	2.45127500	-1.72560700	0.86214000
H	0.24089800	-1.56664300	0.95863100
C	3.39626800	-0.82484700	0.07626900
H	2.68217600	-2.79127400	0.75655900
H	3.69126700	-1.27734800	-0.88563100
O	2.94505000	1.46978500	-0.62444500
H	0.90123000	-1.95549900	-0.65206200
H	2.47555800	-1.47109500	1.93111900
H	4.31615900	-0.53250800	0.59736200

Zero-point correction= 0.226584 (Hartree/Particle)

Thermal correction to Energy= 0.237714

Thermal correction to Enthalpy= 0.238626

Thermal correction to Gibbs Free Energy= 0.189169

sp-E = -594.892060135 hartree

Thermal correction to Gibbs free energy (ZPG) from GoodVibes: -0.000275 hartree

MeOH

O	1.50783900	-0.12565200	-0.72616500
H	1.01286000	0.68180400	-0.55797000
C	1.76542800	-0.74301600	0.50864400
H	0.84265300	-1.02672800	1.04761700
H	2.33175000	-1.66469300	0.31175200
H	2.37450200	-0.11415300	1.18389500

Zero-point correction= 0.051677 (Hartree/Particle)

Thermal correction to Energy= 0.054851

Thermal correction to Enthalpy= 0.055764

Thermal correction to Gibbs Free Energy= 0.029850

sp-E = -115.742156587 hartree

Thermal correction to Gibbs free energy (ZPG) from GoodVibes: -0.000008 hartree

TS3a

C	-0.81165400	2.81045000	-1.00930400
C	-0.08425300	2.15605800	-2.17624300
C	0.63369200	0.97277100	-1.53136500
H	-1.79254900	2.34340600	-0.83285000
H	-0.95872600	3.89465200	-1.09069000
H	-0.75911300	1.82636300	-2.97486900
H	0.64930000	2.85118000	-2.61053700
H	-0.01001100	0.08359400	-1.48012400

H	1.56875000	0.71335500	-2.04099400
C	0.03164600	2.48717700	0.19937100
O	-0.03422800	3.00238200	1.28705200
N	0.90927600	1.44247000	-0.16030500
C	1.74636200	0.77744700	0.71408800
C	2.29424100	1.51001000	1.84515100
H	3.08206800	1.03581000	2.43027700
C	2.69104000	-0.21896900	0.08512600
C	4.04080800	0.13887300	-0.01657800
C	2.26647200	-1.44633000	-0.44039700
C	4.95396700	-0.71564500	-0.63220100
H	4.38533900	1.09911800	0.37349400
C	3.18462800	-2.30112900	-1.04343200
H	1.21574700	-1.73555000	-0.38537300
C	4.52865500	-1.94018200	-1.14222400
H	6.00190300	-0.41990400	-0.70878400
H	2.84277800	-3.25827200	-1.44179700
H	5.24379600	-2.61414300	-1.61818300
O	0.52432600	-0.26216100	1.59937400
H	1.85744000	2.45230500	2.16228300
C	0.94628300	-1.18776700	2.58672400
H	1.38328800	-0.63601300	3.42833000
H	0.06231600	-1.73654500	2.93907500
H	1.68316600	-1.90104600	2.18564100
H	-0.08119400	-0.73693900	0.90261400
F	-0.80790800	-1.45402000	-0.02956400
S	-2.84828000	-0.75250200	-0.08977400
F	-2.59852900	0.05636200	-1.53915800
F	-3.32466100	-2.09499500	-0.91828500

F	-4.35168100	-0.22813500	-0.09495800
F	-2.40394700	0.63102800	0.75143500
F	-3.13758900	-1.52101600	1.34134100

Zero-point correction= 0.296463 (Hartree/Particle)

Thermal correction to Energy= 0.319182

Thermal correction to Enthalpy= 0.320094

Thermal correction to Gibbs Free Energy= 0.244391

sp-E = -1708.07997637 hartree

Thermal correction to Gibbs free energy (ZPG) from GoodVibes: 0.000070 hartree

Int3a

C	-0.27339400	2.97891500	-0.54311200
C	0.96075900	2.98681400	-1.43608500
C	1.59439600	1.62078800	-1.16993200
H	-1.13009600	2.48777600	-1.03285300
H	-0.60507900	3.96128500	-0.18418400
H	0.73531900	3.13324300	-2.49974800
H	1.65042400	3.78449900	-1.12160300
H	1.17983600	0.84926200	-1.83911900
H	2.68493700	1.63021800	-1.28861300
C	0.11413300	2.07911700	0.61214400
O	-0.46409400	1.98746800	1.67503800
N	1.21808400	1.34605400	0.22082700
C	1.63519600	0.14634100	0.92834900
C	1.89951900	0.41709800	2.37834100
H	2.64993600	-0.17204400	2.90696600
C	2.85896900	-0.46380100	0.23911900
C	4.14164600	-0.00409400	0.55216200
C	2.70637600	-1.43710900	-0.75170300

C	5.25603400	-0.51354500	-0.11183700
H	4.27215200	0.76820600	1.31391100
C	3.82135700	-1.95144100	-1.41205100
H	1.70763200	-1.78801900	-1.01537100
C	5.09857700	-1.49153200	-1.09412000
H	6.25222600	-0.14439500	0.14055200
H	3.68849400	-2.71441500	-2.18182300
H	5.97155400	-1.89395300	-1.61231300
O	0.50818600	-0.75627200	0.77884100
H	1.22697000	1.07765500	2.92201200
C	0.42566700	-1.89555000	1.61498800
H	0.25941100	-1.60152100	2.66160400
H	-0.43496400	-2.47569000	1.26268000
H	1.33588400	-2.51167400	1.54215000
H	-0.35765400	-0.53529700	-0.40339200
F	-0.91469000	-0.35003100	-1.18104700
S	-3.29171400	-0.54338400	-0.22314700
F	-3.45433900	1.08142100	-0.15770400
F	-3.82871900	-0.53824200	-1.76090800
F	-4.75935100	-0.73441100	0.31492900
F	-2.70831000	-0.54778700	1.30452200
F	-3.08284400	-2.16261100	-0.29922600

Zero-point correction= 0.297597 (Hartree/Particle)

Thermal correction to Energy= 0.320669

Thermal correction to Enthalpy= 0.321582

Thermal correction to Gibbs Free Energy= 0.243529

sp-E = -1708.09756395 hartree

Thermal correction to Gibbs free energy (ZPG) from GoodVibes: 0.000801 hartree

Int3b

C	3.23201500	-1.32763100	-0.32338700
C	2.13630600	-2.38237400	-0.41149900
C	0.86704900	-1.55029600	-0.59777200
H	3.61967900	-1.05221300	-1.31867400
H	4.09259900	-1.59443900	0.30371300
H	2.28007700	-3.10445700	-1.22510600
H	2.07411600	-2.94338900	0.53306500
H	0.66927600	-1.34034900	-1.66391000
H	-0.02091400	-2.04268800	-0.18282700
C	2.52527000	-0.10101900	0.22673300
O	3.07427500	0.89634100	0.65404300
N	1.17005200	-0.31113500	0.12136000
C	0.19532900	0.76950400	0.26053400
C	0.37649200	1.49272500	1.56732900
H	-0.49165600	1.93269500	2.06074200
C	-1.21965600	0.17578300	0.17365900
C	-1.80024000	-0.42982000	1.29269800
C	-1.91962800	0.17969100	-1.03478100
C	-3.06021600	-1.01929500	1.20626100
H	-1.25629200	-0.45222000	2.24033500
C	-3.18368300	-0.40370000	-1.12173900
H	-1.46357900	0.63858600	-1.91327300
C	-3.75741800	-1.00534800	-0.00247800
H	-3.49960300	-1.49143200	2.08766700
H	-3.72164200	-0.38999700	-2.07224400
H	-4.74644800	-1.46360700	-0.07109600
O	0.43982500	1.62012600	-0.85710700
H	1.38310400	1.73247900	1.90465000

C	-0.12544100	2.90715400	-0.81076500
H	0.34077000	3.53593400	-0.03341100
H	0.05576100	3.36808700	-1.79055300
H	-1.21549500	2.88096200	-0.63283400

Zero-point correction= 0.268378 (Hartree/Particle)

Thermal correction to Energy= 0.282414

Thermal correction to Enthalpy= 0.283326

Thermal correction to Gibbs Free Energy= 0.227580

sp-E = -710.201309267 hartree

Thermal correction to Gibbs free energy (ZPG) from GoodVibes: -0.000234 hartree

HF

F	0.00000000	0.00000000	0.09223600
H	0.00000000	0.00000000	-0.83012000

Zero-point correction= 0.009411 (Hartree/Particle)

Thermal correction to Energy= 0.011692

Thermal correction to Enthalpy= 0.012605

Thermal correction to Gibbs Free Energy= -0.006337

sp-E = -100.470296285 hartree

Thermal correction to Gibbs free energy (ZPG) from GoodVibes: 0.000000 hartree

$SF_5^{\bullet}$

S	0.00059300	-0.00019600	-0.20904400
F	-1.58744400	0.33936300	-0.24670100
F	0.00180000	-0.00050000	1.36160500
F	-0.34119400	-1.58711400	-0.24740500
F	1.58799100	-0.33959500	-0.24857500
F	0.33779300	1.58819400	-0.24729000

Zero-point correction= 0.015323 (Hartree/Particle)

Thermal correction to Energy= 0.020494

Thermal correction to Enthalpy= 0.021407

Thermal correction to Gibbs Free Energy= -0.013514

sp-E = -897.403774023 hartree

Thermal correction to Gibbs free energy (ZPG) from GoodVibes: -0.000045 hartree

Int3c

C	-0.56402600	0.33159400	0.51706700
C	0.47737000	-0.57136400	1.12628600
H	0.01516000	-1.43670200	1.60624400
H	1.07968900	-0.02663500	1.85963300
C	-1.89827300	-0.19489600	0.27781600
C	-3.03192300	0.62482900	0.46066100
C	-2.07586400	-1.55212500	-0.06485600
C	-4.30487700	0.10215400	0.28610200
H	-2.92254300	1.65699600	0.79155800
C	-3.35125700	-2.05520000	-0.27095800
H	-1.21987600	-2.21116000	-0.20310600
C	-4.46528500	-1.23132600	-0.09419700
H	-5.17667900	0.73614300	0.45173100
H	-3.47904700	-3.09833500	-0.56219300
H	-5.46805600	-1.63636200	-0.24230300
N	-0.24752100	1.57090200	0.21961100
C	-0.95936100	2.44064800	-0.74509500
C	0.94784800	2.32185600	0.69754000
C	0.18607400	3.20401500	-1.40287900
H	-1.54956300	1.82313200	-1.43127200
C	1.13785300	3.47221600	-0.24333200
H	-0.17326700	4.11934800	-1.88565100

H	0.87628500	4.38935000	0.30984000
O	1.53226400	2.03514100	1.69042400
H	-1.62801000	3.12260900	-0.20147900
H	0.66750100	2.57325200	-2.16331800
H	2.20019500	3.54461100	-0.51081700
S	1.68967600	-1.25071500	-0.08988100
F	2.34995500	0.20037300	-0.32022000
F	2.76209100	-1.83987700	-1.11632100
F	2.71817400	-1.54982300	1.09913400
F	1.08521300	-2.73471300	0.05113700
F	0.70993000	-0.98447600	-1.34777100

Zero-point correction= 0.249279 (Hartree/Particle)

Thermal correction to Energy= 0.265262

Thermal correction to Enthalpy= 0.266174

Thermal correction to Gibbs Free Energy= 0.206344

sp-E = -1492.37668834 hartree

Thermal correction to Gibbs free energy (ZPG) from GoodVibes: -0.000289 hartree

TS3b

C	3.35050900	-1.39236000	2.63539500
C	4.31408800	-0.75211500	1.64429000
C	3.55835600	0.49460600	1.19514800
H	3.44350500	-0.95534400	3.64337000
H	3.43037200	-2.48177500	2.73851600
H	5.28594700	-0.49748100	2.08216000
H	4.48119800	-1.41733600	0.78505800
H	3.65910800	1.29931400	1.93717700
H	3.86220600	0.87072300	0.21204900
C	1.98422900	-1.03548200	2.11554600

O	0.92167100	-1.47026900	2.44740400
N	2.16056400	0.01620600	1.14017300
C	1.20325200	0.45914100	0.31449300
C	0.17746000	-0.54145100	-0.17048900
H	-0.63588800	-0.05005300	-0.70845700
C	1.40484200	1.70717300	-0.43935800
C	0.93451800	1.83675900	-1.75568800
C	1.99272000	2.82304300	0.18386900
C	1.09248600	3.03523400	-2.44421400
H	0.45917800	1.00320700	-2.26804700
C	2.13437300	4.02099700	-0.50108600
H	2.29491000	2.77261700	1.22917100
C	1.69357400	4.12707100	-1.82168400
H	0.73544200	3.11437600	-3.47196300
H	2.58029700	4.88061400	0.00136600
H	1.80784100	5.06909500	-2.36138100
O	-0.18575600	1.23853900	1.73270400
H	-0.25653000	-1.10123400	0.66200500
S	0.83398000	-1.83055500	-1.30990000
F	0.04305100	-1.21346300	-2.57514900
F	1.37182100	-2.97821600	-2.29444100
F	2.14195900	-0.96657300	-1.71653000
F	-0.42104100	-2.77203200	-0.98262100
F	1.68542800	-2.53909600	-0.13499100
C	-0.82056900	2.48180600	1.50614300
H	-0.90645300	2.70213600	0.43004900
H	-1.83686600	2.45660400	1.92400100
H	-0.24505900	3.28084400	1.99196800
H	-0.90769900	0.54897000	1.81046600

F	-2.07601400	-0.40572400	1.74606600
S	-3.50385100	0.00886200	0.31219300
F	-4.02316700	1.36553900	1.14547200
F	-2.45164900	0.99912500	-0.64142400
F	-4.56199800	-0.92154300	1.15378100
F	-3.02830100	-1.32190400	-0.57548400
F	-4.64274200	0.28785100	-0.77695200

Zero-point correction= 0.320037 (Hartree/Particle)

Thermal correction to Energy= 0.347774

Thermal correction to Enthalpy= 0.348686

Thermal correction to Gibbs Free Energy= 0.262084

sp-E = -2605.57589730 hartree

Thermal correction to Gibbs free energy (ZPG) from GoodVibes: -0.000208 hartree

2a

C	0.51779900	-0.14446300	0.38920000
C	-0.46602400	-0.93507100	-0.49144000
H	-0.28046400	-2.00583800	-0.38635100
H	-0.34053900	-0.62861600	-1.53425900
S	-2.26742200	-0.81545900	-0.19479600
F	-2.24254500	-2.01274000	0.88559600
F	-3.87372200	-0.79134300	-0.02263500
F	-2.49952800	-1.88662600	-1.38661000
F	-2.20326400	0.25774500	0.99942400
F	-2.43953100	0.39782300	-1.25055700
N	0.30381500	1.30112800	0.22898100
C	0.03749200	2.20062100	1.35168200
C	0.19087700	1.91388300	-0.98910700
C	0.14498900	3.59081600	0.71511600

H	-0.96217100	2.01126800	1.76833600
C	-0.22054400	3.35155000	-0.74584900
H	-0.50152800	4.32127500	1.21724900
H	-1.30886000	3.40514900	-0.91593400
H	0.76996800	2.06511600	2.15664200
H	1.18150800	3.95334200	0.78665500
H	0.26446000	4.02352700	-1.46482600
O	0.36962000	1.38299100	-2.07170200
C	1.93891200	-0.54780800	-0.05364600
C	2.29251400	-1.90291200	-0.09253500
C	2.91624400	0.40767600	-0.33824900
C	3.58670100	-2.29219000	-0.42909300
H	1.56355100	-2.67486800	0.16257000
C	4.21273000	0.02017100	-0.67527500
H	2.67162700	1.46851200	-0.28892600
C	4.55156200	-1.33019700	-0.72715900
H	3.84180900	-3.35351900	-0.45337500
H	4.96179000	0.78307000	-0.89705300
H	5.56632800	-1.63337300	-0.99316900
O	0.26920900	-0.50707300	1.71455600
C	1.32408900	-0.49767300	2.65532300
H	0.85198400	-0.46428300	3.64520300
H	1.94161000	-1.40596600	2.58055700
H	1.98360900	0.37716000	2.54694600

Zero-point correction= 0.292570 (Hartree/Particle)

Thermal correction to Energy= 0.311393

Thermal correction to Enthalpy= 0.312306

Thermal correction to Gibbs Free Energy= 0.245955

sp-E = -1607.71915894 hartree

Thermal correction to Gibbs free energy (ZPG) from GoodVibes: -0.000045 hartree

NMR data for pentafluorosulfanyl compounds

2-(pentafluoro-λ⁶-sulfaneyl)-1-(p-tolyl)ethan-1-one

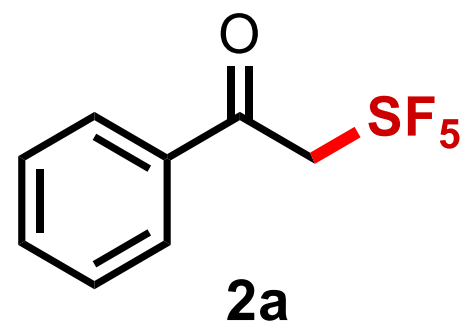

The product **2a** was purified with silica gel chromatography (PE: DCM = 10:1) as a colorless oil (65% yield)

^{1}H NMR (400 MHz, $CDCl_3$) δ 8.10 – 7.90 (m, 2H), 7.71 – 7.62 (m, 1H), 7.58 – 7.40 (m, 2H), 4.88 (p, J = 7.8 Hz, 2H).

^{13}C NMR (101 MHz, $CDCl_3$) δ 187.20 (t, J = 3.7 Hz), 135.61, 134.63, 129.26, 129.22, 71.79 (p, J = 13.0 Hz).

^{19}F NMR (376 MHz, CDC13) δ 81.96 – 80.09 (m, 1F), 73.46 – 71.69 (m, 4F).

HRMS (ESI) (*m/z*): $[M+Na]^+$ calcd. for $C_8H_7F_5OSNa$: 269.0030, found: 269..0042.

2-(pentafluoro-λ⁶-sulfaneyl)-1-(p-tolyl)ethan-1-one

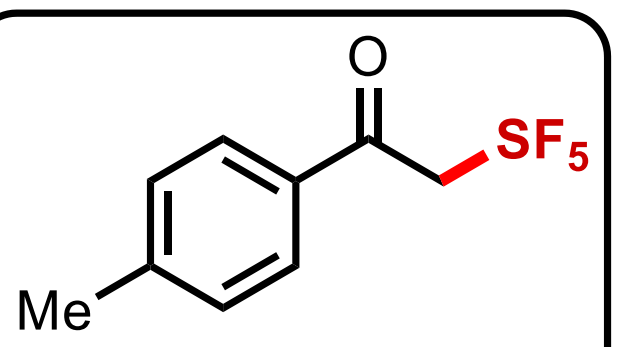

The product **2b** was purified with silica gel chromatography (PE: DCM = 10:1) as a colorless oil (54% yield)

^{1}H NMR (600 MHz, $CDCl_3$) δ 7.97 – 7.86 (m, 2H), 7.33 (d, J = 8.1 Hz, 2H), 4.85 (p, J = 7.8 Hz, 2H), 2.44 (s, 3H).

^{13}C NMR (151 MHz, $CDCl_3$) δ 186.77 (t, J = 3.6 Hz), 145.94, 133.16, 129.94, 129.44, 71.83 (p, J = 12.8 Hz), 21.92.

^{19}F NMR (565 MHz, $CDCl_3$) δ 81.98 – 80.62 (m, 1F), 73.04 – 72.27 (m, 4F).

HRMS (ESI) (*m/z*): $[M+H]^+$ calcd. for $C_9H_{10}F_5OS$: 261.0373, found: 261.0378.

1-(4-ethylphenyl)-2-(pentafluoro-λ^6-sulfaneyl)ethan-1-one

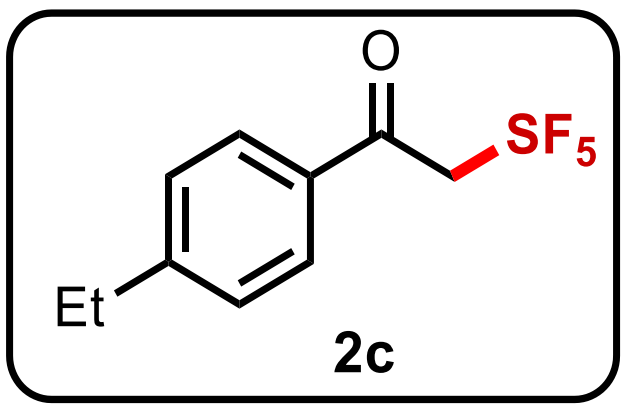

The product **2c** was purified with silica gel chromatography (PE: DCM = 10:1) as a colorless oil (51% yield)

^{1}H NMR (500 MHz, $CDCl_3$) δ 7.99 – 7.87 (m, 2H), 7.35 (d, *J* = 8.0 Hz, 2H), 4.85 (p, *J* = 7.8 Hz, 2H), 2.74 (q, *J* = 7.6 Hz, 2H), 1.27 (t, *J* = 7.7 Hz, 3H).

^{13}C NMR (126 MHz, $CDCl_3$) δ 186.78 (t, *J* = 3.6 Hz), 152.00, 133.33, 129.55, 128.75, 71.83 (p, *J* = 12.6 Hz), 29.15, 15.12.

^{19}F NMR (471 MHz, $CDCl_3$) δ 82.16 – 80.21 (m, 1F), 72.84 – 72.02 (m, 4F).

HRMS (ESI) (*m/z*): $[M+Na]^+$ calcd. for $C_{10}H_{11}F_5NaOS$: 297.0343, found: 294.0343.

1-(4-isopropylphenyl)-2-(pentafluoro-λ^6-sulfaneyl)ethan-1-one

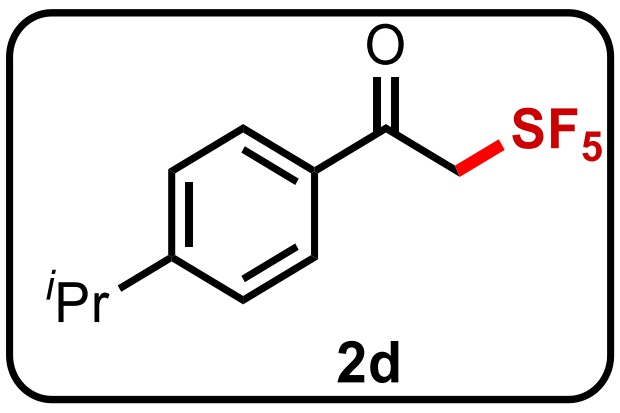

The product **2d** was purified with silica gel chromatography (PE: DCM = 10:1) as a colorless oil (55% yield)

^{1}H NMR (500 MHz, $CDCl_3$) δ 7.94 (d, *J* = 8.0 Hz, 2H), 7.38 (d, *J* = 8.1 Hz, 2H), 4.85 (p, *J* = 7.9 Hz, 2H), 3.05 – 2.93 (m, 1H), 1.29 (d, *J* = 6.9 Hz, 6H).

^{13}C NMR (126 MHz, $CDCl_3$) δ 186.73 (t, *J* = 3.6 Hz), 156.52, 133.52, 129.61, 127.36, 71.70 (p, *J* = 12.9 Hz), 34.50, 23.67.

^{19}F NMR (471 MHz, $CDCl_3$) δ 81.96 – 80.54 (m, 1F), 72.82 – 72.21 (m, 4F).

HRMS (ESI) (*m/z*): $[M+H]^+$ calcd. for $C_{11}H_{14}F_5OS$: 289.0686, found: 289.0681.

1-(4-(tert-butyl)phenyl)-2-(pentafluoro-λ^6-sulfaneyl)ethan-1-one

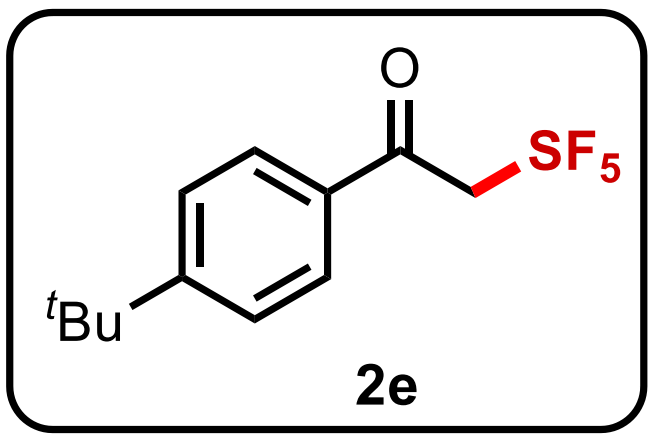

The product **2e** was purified with silica gel chromatography (PE: DCM = 10:1) as a colorless oil (50% yield)

^{1}H NMR (600 MHz, $CDCl_3$) δ 7.95 (d, J = 8.3 Hz, 2H), 7.54 (d, J = 8.5 Hz, 2H), 4.85 (p, J = 7.8 Hz, 2H), 1.35 (s, 9H).

^{13}C NMR (151 MHz, $CDCl_3$) δ 186.75, 158.75, 133.02, 129.32, 126.23, 71.81 (p, J = 12.8 Hz), 35.44, 31.09.

^{19}F NMR (565 MHz, $CDCl_3$) δ 82.10 – 80.64 (m, 1F), 73.02 – 72.28 (m, 4F).

HRMS (ESI) (*m/z*): $[M+H]^+$ calcd. for $C_{12}H_{16}F_5OS$: 303.0842, found: 303.0838.

2-(pentafluoro-λ^6-sulfaneyl)-1-(4-(trimethylsilyl)phenyl)ethan-1-one

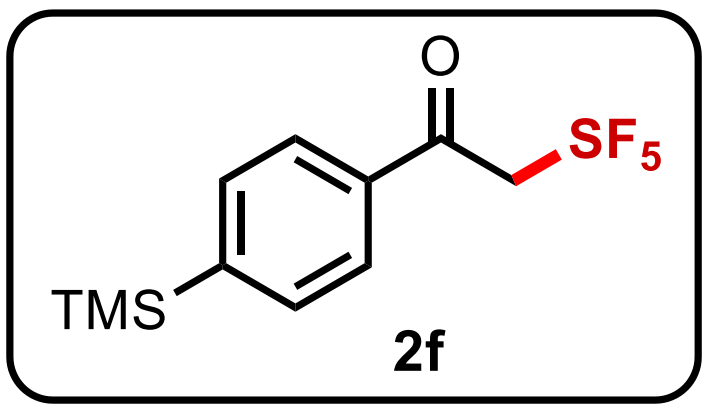

The product **2f** was purified with silica gel chromatography (PE: DCM = 10:1) as a colorless oil (59% yield)

^{1}H NMR (600 MHz, $CDCl_3$) δ 8.01 – 7.86 (m, 2H), 7.77 – 7.59 (m, 2H), 4.87 (p, J = 8.0 Hz, 2H), 0.31 (s, 9H).

^{13}C NMR (151 MHz, $CDCl_3$) δ 187.43 (t, J = 3.8 Hz), 149.61, 135.51, 134.09, 128.07, 71.77 (p, J = 12.9 Hz), -1.31.

^{19}F NMR (565 MHz, $CDCl_3$) δ 81.83 – 80.62 (m, 1F), 72.98 – 72.39 (m, 4F).

HRMS (ESI) (*m/z*): $[M+H]^+$ calcd. for $C_{11}H_{16}F_5OSiS$: 319.0611, found: 319.0620.

2-(pentafluoro-λ^6-sulfaneyl)-1-(4-phenoxyphenyl)ethan-1-one

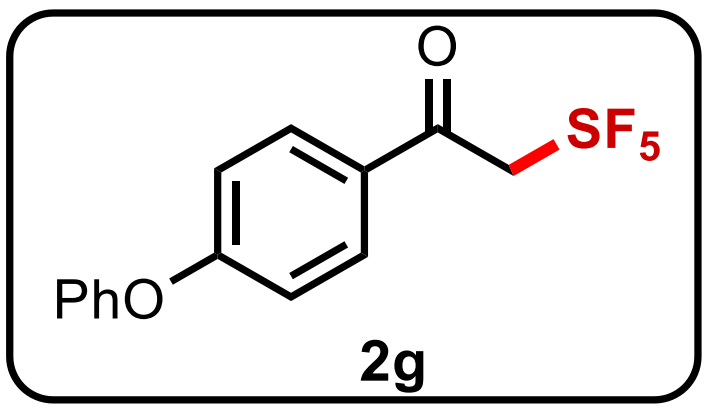

The product **2g** was purified with silica gel chromatography (PE: DCM = 3:1) as a colorless oil (42% yield)

^{1}H NMR (600 MHz, $CDCl_3$) δ 8.06 – 7.95 (m, 2H), 7.45 (dd, J = 8.5, 7.4 Hz, 2H), 7.28 (d, J = 7.9 Hz, 1H), 7.15 – 7.10 (m, 2H), 7.08 – 7.04 (m, 2H), 4.85 (p, J = 7.8 Hz, 2H).

^{13}C NMR (151 MHz, $CDCl_3$) δ 185.60 (t, J = 3.6 Hz), 163.55, 154.88, 131.79, 130.36, 129.96, 125.35, 120.75, 117.42, 71.86 (p, J = 12.9 Hz).

^{19}F NMR (565 MHz, $CDCl_3$) δ 82.00 – 80.71 (m, 1F), 72.74 – 72.27 (m, 4F).

HRMS (ESI) (*m/z*): $[M+H]^+$ calcd. for $C_{14}H_{12}F_5O_2S$: 339.0478, found: 339.0483.

2-(pentafluoro-λ^6-sulfaneyl)-1-(4-(trifluoromethoxy)phenyl)ethan-1-one

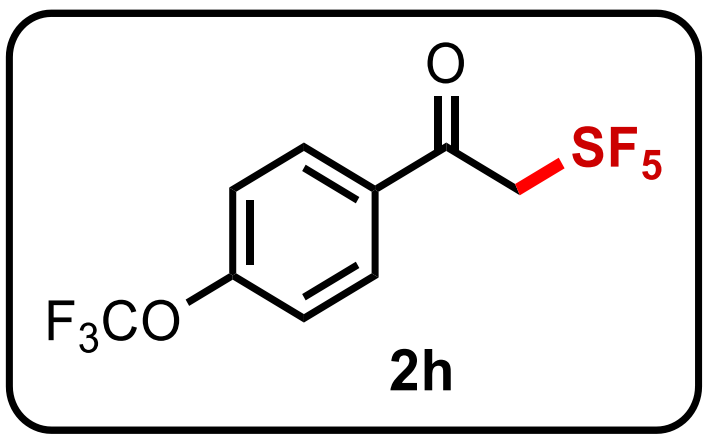

The product **2h** was purified with silica gel chromatography (PE: DCM = 7:1) as a colorless oil (31% yield)

^{1}H NMR (500 MHz, $CDCl_3$) δ 8.15 – 7.99 (m, 2H), 7.36 (d, J = 8.4 Hz, 2H), 4.86 (p, J = 7.8 Hz, 2H).

^{13}C NMR (126 MHz, $CDCl_3$) δ 185.75 (t, J = 3.8 Hz), 153.76, 133.58, 131.42, 120.73, 120.35 (q, J = 259.7 Hz), 71.80 (p, J = 12.6 Hz).

^{19}F NMR (471 MHz, $CDCl_3$) δ 81.56 – 79.59 (m, 1F), 73.21 – 72.27 (m, 4F), -57.59 (s, 3F).

HRMS (ESI) (*m/z*): $[M+H]^+$ calcd. for $C_9H_7F_8O_2S$: 331.0039, found: 331.0048.

1-(4-fluorophenyl)-2-(pentafluoro-λ^6-sulfaneyl)ethan-1-one

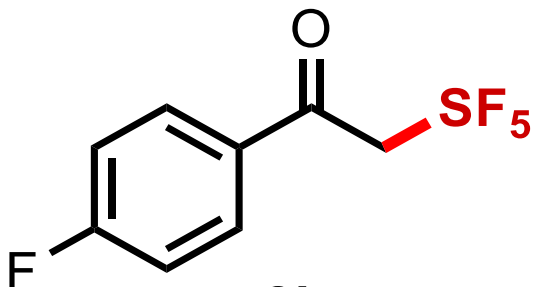

2i

The product **2i** was purified with silica gel chromatography (PE: DCM = 10:1) as a colorless oil (47% yield)

^{1}H NMR (600 MHz, $CDCl_3$) δ 8.09 – 8.00 (m, 2H), 7.21 (td, J = 8.6, 2.2 Hz, 2H), 4.85 (p, J = 7.8 Hz, 2H).

^{13}C NMR (151 MHz, $CDCl_3$) δ 185.66 (t, J = 4.5 Hz), 167.53, 165.82, 132.12 (d, J = 9.7 Hz), 116.55 (d, J = 22.2 Hz), 71.85 (p, J = 13.4 Hz).

^{19}F NMR (565 MHz, $CDCl_3$) δ 81.55 – 80.26 (m, 1F), 73.03 – 72.42 (m, 4F), -101.99 (m, 1F).

HRMS (ESI) (*m/z*): $[M+Na]^+$ calcd. for $C_8H_6F_6NaOS$: 286.9936, found: 286.9941.

1-(4-chlorophenyl)-2-(pentafluoro-λ^6-sulfaneyl)ethan-1-one

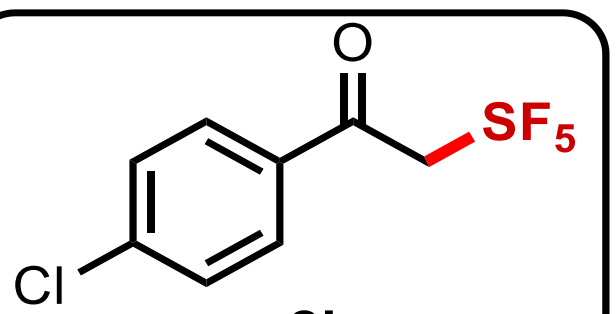

2j

The product **2j** was purified with silica gel chromatography (PE: DCM = 10:1) as a colorless oil (50% yield)

^{1}H NMR (600 MHz, $CDCl_3$) δ 7.98 – 7.91 (m, 2H), 7.56 – 7.49 (m, 2H), 4.85 (p, J = 7.8 Hz, 2H).

^{13}C NMR (151 MHz, $CDCl_3$) δ 186.09 (t, J = 4.5 Hz), 141.44, 133.87, 130.63, 129.63, 71.77 (p, J = 14.0 Hz).

^{19}F NMR (565 MHz, $CDCl_3$) δ 81.51 – 80.25 (m, 1F), 73.04 – 72.71 (m, 4F).

HRMS (ESI) (*m/z*): $[M+Na]^+$ calcd. for $C_8H_6ClF_5NaOS$: 302.9640, found: 302.9654.

1-(4-bromophenyl)-2-(pentafluoro-λ^6-sulfaneyl)ethan-1-one

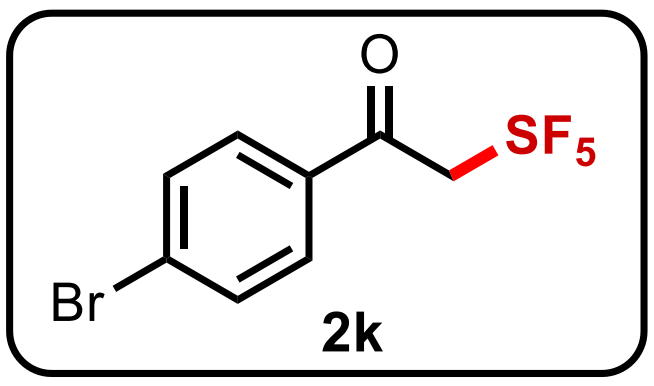

The product **2k** was purified with silica gel chromatography (PE: DCM = 10:1) as a colorless oil (56% yield)

^{1}H NMR (600 MHz, $CDCl_3$) δ 7.90 – 7.84 (m, 2H), 7.72 – 7.63 (m, 2H), 4.84 (p, *J* = 7.8 Hz, 2H).

^{13}C NMR (151 MHz, $CDCl_3$) δ 186.32, 134.27, 132.64, 130.66, 130.29, 71.75 (p, *J* = 13.6 Hz).

^{19}F NMR (471 MHz, $CDCl_3$) δ 81.41 – 80.00 (m, 1F), 73.12 – 72.51 (m, 4F).

HRMS (ESI) (*m/z*): $[M+Na]^+$ calcd. for $C_8H_6BrF_5OS$:346.9135, found: 346.9128.

1-(4-iodophenyl)-2-(pentafluoro-λ^6-sulfaneyl)ethan-1-one

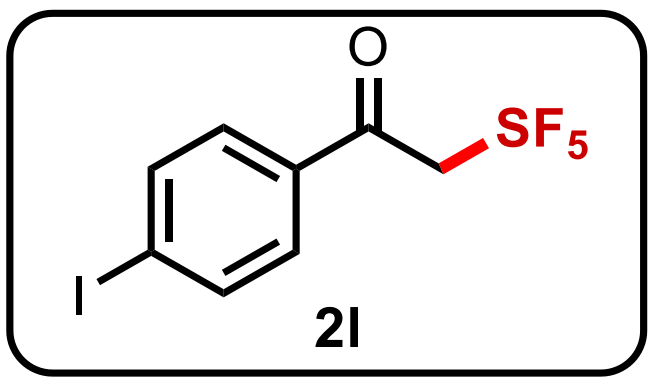

The product **2l** was purified with silica gel chromatography (PE: DCM = 10:1) as a colorless oil (47% yield)

^{1}H NMR (500 MHz, $CDCl_3$) δ 7.91 (d, *J* = 8.9 Hz, 2H), 7.70 (d, *J* = 8.8 Hz, 2H), 4.83 (p, *J* = 8.0 Hz, 2H).

^{13}C NMR (126 MHz, $CDCl_3$) δ 186.63, 138.64, 134.83, 130.41, 103.28, 72.04 – 71.06 (p, *J* = 13.4 Hz).

^{19}F NMR (471 MHz, $CDCl_3$) δ 81.48 – 79.95 (m, 1F), 73.14 – 72.42 (m, 4F).

HRMS (ESI) (*m/z*): $[M+Na]^+$ calcd. for $C_8H_6F_5IOSNa$: 394.9002, found: 394.9011.

methyl 4-(2-(pentafluoro-λ^6-sulfaneyl)acetyl)benzoate

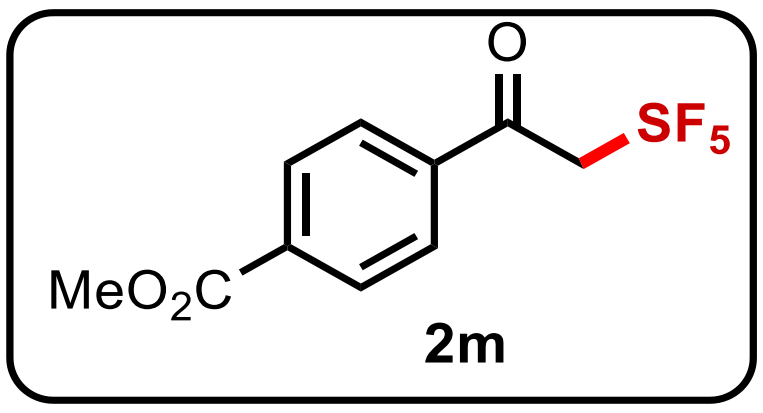

The product **2m** was purified with silica gel chromatography (PE: DCM = 1:1) as a colorless oil (40% yield)

^{1}H NMR (600 MHz, $CDCl_3$) δ 8.19 (s, 2H), 8.06 (d, J = 8.4 Hz, 2H), 4.90 (p, J = 8.0 Hz, 2H), 3.97 (s, 3H).

^{13}C NMR (151 MHz, $CDCl_3$) δ 186.85, 165.95, 138.55, 135.21, 130.35, 129.16, 71.89 (p, J = 13.6 Hz), 52.82.

^{19}F NMR (565 MHz, $CDCl_3$) δ 81.32 – 79.98 (m, 1F), 73.30 – 72.92 (m, 4F).

HRMS (ESI) (*m/z*): $[M+H]^+$ calcd. for $C_{10}H_{10}F_5O_3S$: 305.0271, found: 305.0266.

1-(4-acetylphenyl)-2-(pentafluoro-λ^6-sulfaneyl)ethan-1-one

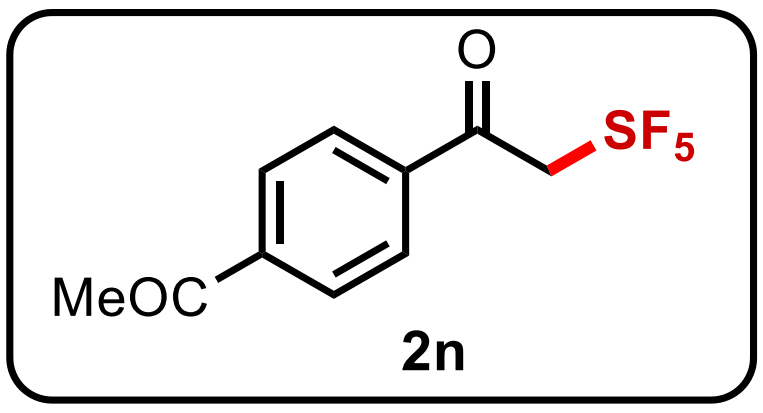

The product **2n** was purified with silica gel chromatography (PE: DCM = 3:1) as a colorless oil (35% yield)

^{1}H NMR (600 MHz, $CDCl_3$) δ 8.09 (s, 4H), 4.91 (p, J = 7.7 Hz, 2H), 2.66 (s, 3H).

^{13}C NMR (151 MHz, $CDCl_3$) δ 197.28, 186.82 (t, J = 3.8 Hz), 141.18, 138.47, 129.47, 128.96, 71.91 (p, J = 13.5 Hz), 27.09.

^{19}F NMR (565 MHz, $CDCl_3$) δ 81.30 – 80.05 (m, 1F), 73.39 – 72.85 (m, 4F).

HRMS (ESI) (*m/z*): $[M+H]^+$ calcd. for $C_{10}H_{10}F_5O_2S$: 289.0322, found: 289.0323.

2-(pentafluoro-λ^6-sulfaneyl)-1-(4-(trifluoromethyl)phenyl)ethan-1-one

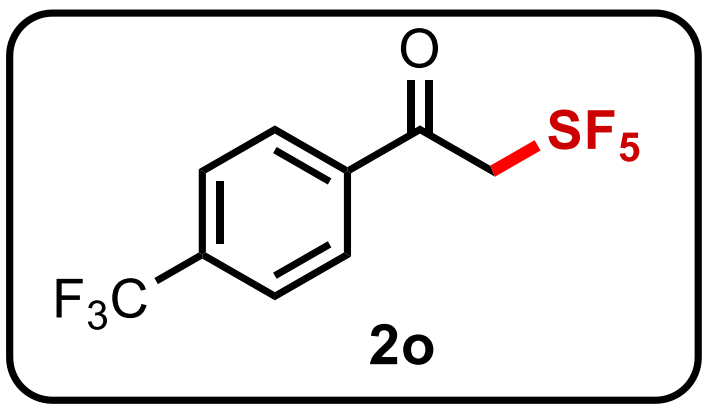

The product **2o** was purified with silica gel chromatography (PE: DCM = 10:1) as a colorless oil (40% yield)

^{1}H NMR (500 MHz, $CDCl_3$) δ 8.12 (d, *J* = 8.2 Hz, 2H), 7.81 (d, *J* = 8.3 Hz, 2H), 4.90 (p, *J* = 7.8 Hz, 2H).

^{13}C NMR (126 MHz, $CDCl_3$) δ 186.43, 138.13, 135.68 (q, *J* = 34.0 Hz), 129.58, 126.33 (q, *J* = 3.7 Hz), 123.44 (d, *J* = 273.0 Hz), 71.86 (p, *J* = 13.9 Hz).

^{19}F NMR (471 MHz, $CDCl_3$) δ 81.07 – 79.66 (m, 1F), 73.36 – 72.80 (m, 4F), -63.38 (s, 3F).

HRMS (ESI) (*m/z*): $[M+Na]^+$ calcd. for $C_9H_6F_8NaOS$: 336.9904, found: 336.9909.

1-(4-(difluoromethyl)phenyl)-2-(pentafluoro-λ^6-sulfaneyl)ethan-1-one

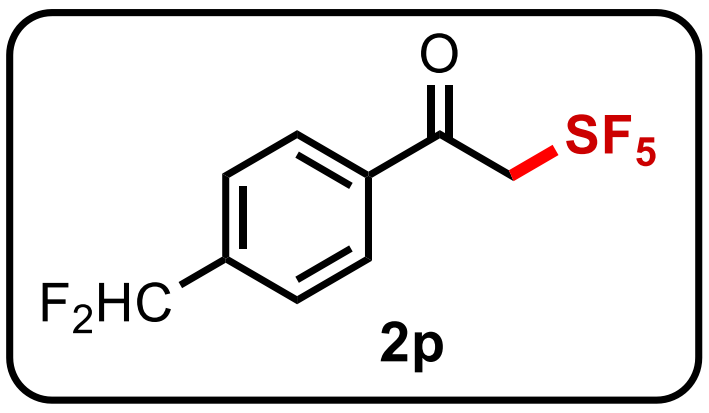

The product **2p** was purified with silica gel chromatography (PE: DCM = 10:1) as a colorless oil (31% yield)

^{1}H NMR (600 MHz, $CDCl_3$) δ 8.09 (d, *J* = 8.1 Hz, 2H), 7.69 (d, *J* = 8.0 Hz, 2H), 6.72 (t, *J* = 55.9 Hz, 1H), 4.89 (p, *J* = 7.8 Hz, 2H).

^{13}C NMR (151 MHz, $CDCl_3$) δ 186.66, 139.93 (t, *J* = 22.5 Hz), 137.25, 129.61, 126.53 (t, *J* = 6.1 Hz), 113.72 (t, *J* = 240.3 Hz), 71.85 (p, *J* = 13.6 Hz).

^{19}F NMR (565 MHz, $CDCl_3$) δ 81.38 – 80.02 (m, 1F), 73.28 – 72.77 (m, 4F), -112.98 (d, J = 56.5 Hz, 2F).

HRMS (ESI) (*m/z*): $[M+Na]^+$ calcd. for $C_9H_7F_7NaOS$:318.9998, found: 319.0004.

2-(pentafluoro-λ^6-sulfaneyl)-1-(m-tolyl)ethan-1-one

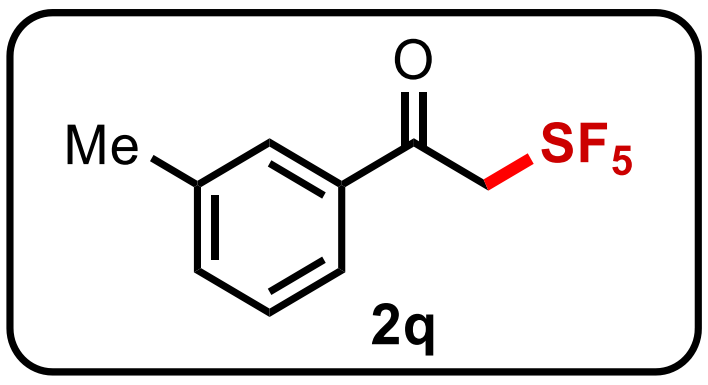

The product **2q** was purified with silica gel chromatography (PE: DCM = 10:1) as a colorless oil (57% yield)

^{1}H NMR (500 MHz, $CDCl_3$) δ 7.88 – 7.76 (m, 2H), 7.50 – 7.37 (m, 2H), 4.86 (p, *J* = 7.8 Hz, 2H), 2.44 (s, 3H).

^{13}C NMR (126 MHz, $CDCl_3$) δ 187.36 (t, *J* = 3.8 Hz), 139.22, 135.66, 135.47, 129.71, 129.06, 126.56, 71.85 (p, *J* = 12.9 Hz), 21.47.

^{19}F NMR (471 MHz, $CDCl_3$) δ 81.96 – 80.35 (m, 1F), 72.87 – 72.24 (m, 4F).

HRMS (ESI) (*m/z*): $[M+H]^+$ calcd. for $C_9H_{10}F_5OS$: 261.0373, found: 261.0383.

1-(3-methoxyphenyl)-2-(pentafluoro-λ^6-sulfaneyl)ethan-1-one

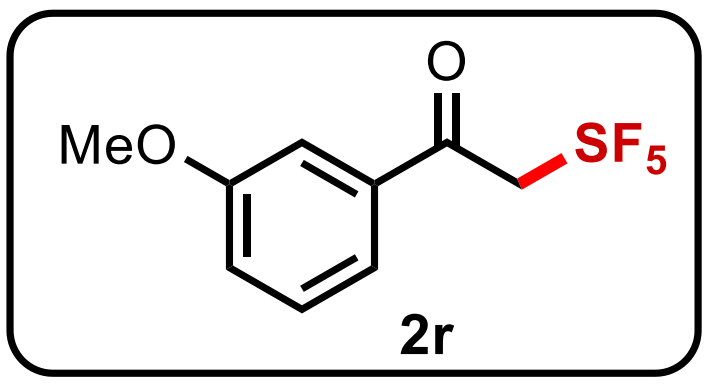

The product **2r** was purified with silica gel chromatography (PE: DCM = 8:1) as a colorless oil (48% yield)

^{1}H NMR (400 MHz, $CDCl_3$) δ 7.58 (d, *J* = 7.7 Hz, 1H), 7.54 – 7.49 (m, 1H), 7.44 (t, *J* = 8.0 Hz, 1H), 7.20 (dd, *J* = 8.4, 2.7 Hz, 1H), 4.86 (p, *J* = 7.8 Hz, 2H), 3.87 (s, 3H).

^{13}C NMR (101 MHz, $CDCl_3$) δ 187.05, 160.24, 136.84, 130.18, 121.95, 121.40, 113.14, 71.85 (p, *J* = 13.1 Hz), 55.64.

^{19}F NMR (376 MHz, $CDCl_3$) δ 81.94 – 80.10 (m, 1F), 72.93 – 72.13 (m, 4F).

HRMS (ESI) (*m/z*): $[M+H]^+$ calcd. for $C_9H_{10}F_5O_2S$: 277.0316, found: 277.0326.

1-(3-(methylthio)phenyl)-2-(pentafluoro-λ^6-sulfaneyl)ethan-1-one

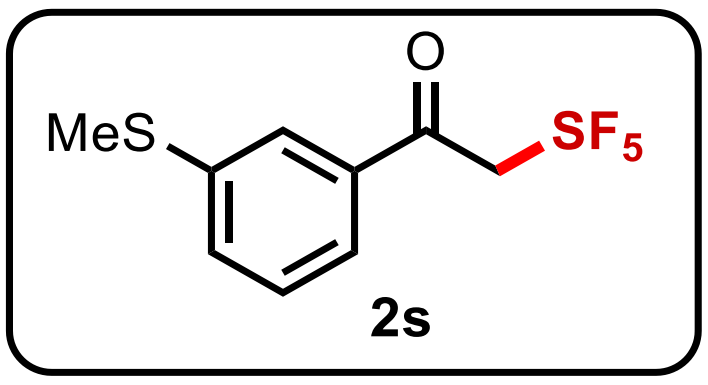

The product **2s** was purified with silica gel chromatography (PE: DCM = 10:1) as a colorless oil (40% yield)

^{1}H NMR (600 MHz, $CDCl_3$) δ 7.89 – 7.82 (m, 1H), 7.73 (d, J = 7.8 Hz, 1H), 7.60 – 7.48 (m, 1H), 7.44 (t, J = 7.6 Hz, 1H), 4.86 (p, J = 7.4 Hz, 2H), 2.54 (s, 3H).

^{13}C NMR (151 MHz, $CDCl_3$) δ 186.90, 140.89, 136.03, 132.25, 129.42, 126.11, 125.71, 71.83 (p, J = 13.0 Hz), 15.57.

^{19}F NMR (565 MHz, $CDCl_3$) δ 81.72 – 80.34 (m, 1F), 73.35 – 72.44 (m, 4F).

HRMS (ESI) (*m/z*): $[M+Na]^+$ calcd. for $C_9H_9F_5OS_2Na$: 314.9907, found: 314.9925.

1-(3-fluorophenyl)-2-(pentafluoro-λ⁶-sulfaneyl)ethan-1-one

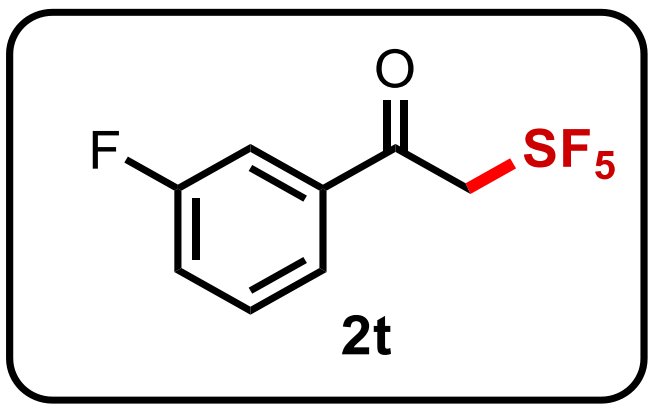

The product **2t** was purified with silica gel chromatography (PE: DCM = 10:1) as a colorless oil (40% yield)

^{1}H NMR (600 MHz, $CDCl_3$) δ 7.79 (d, J = 7.8 Hz, 1H), 7.69 (dt, J = 9.3, 2.1 Hz, 1H), 7.59 – 7.50 (m, 1H), 7.37 (td, J = 8.0, 2.4 Hz, 1H), 4.86 (p, J = 7.8 Hz, 2H).

^{13}C NMR (151 MHz, $CDCl_3$) δ 186.12, 163.09 (d, J = 249.4 Hz), 137.50 (d, J = 8.2 Hz), 130.98 (d, J = 8.3 Hz), 125.07 (d), 121.85 (d, J = 21.7 Hz), 115.94 (d, J = 23.2 Hz), 71.78 (p, J = 14.6 Hz).

^{19}F NMR (565 MHz, $CDCl_3$) δ 81.34 – 80.00 (m, 1F), 73.10 – 72.75 (m, 4F), -109.21 – -112.09 (m, 1F).

HRMS (ESI) (*m/z*): $[M+H]^+$ calcd. for $C_8H_7F_6OS$: 265.0116, found: 265.0118.

1-(3-chlorophenyl)-2-(pentafluoro-λ⁶-sulfaneyl)ethan-1-one

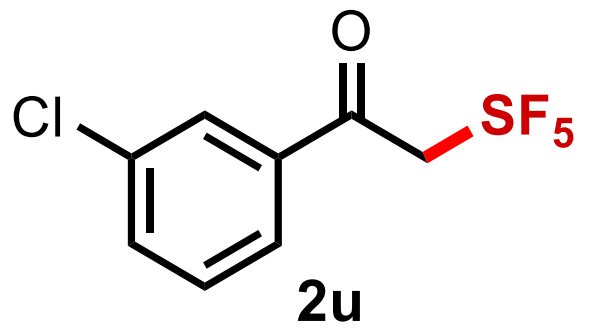

2u

The product **2u** was purified with silica gel chromatography (PE: DCM = 10:1) as a colorless oil (58% yield)

^{1}H NMR (400 MHz, $CDCl_3$) δ 7.97 (d, *J* = 2.1 Hz, 1H), 7.88 (d, *J* = 7.8 Hz, 1H), 7.63 (d, *J* = 8.0 Hz, 1H), 7.49 (t, *J* = 7.9 Hz, 1H), 4.85 (p, *J* = 7.7 Hz, 2H).

^{13}C NMR (101 MHz, $CDCl_3$) δ 186.10, 136.97, 135.70, 134.60, 130.53, 129.21, 127.32, 71.73 (p, *J* = 13.5 Hz).

^{19}F NMR (376 MHz, $CDCl_3$) δ 81.49 – 79.73 (m, 1F), 73.24 – 72.53 (m, 4F).

HRMS (ESI) (*m/z*): $[M+Na]^+$ calcd. for $C_8H_6ClF_5NaOS$: 302.9640, found: 302.9644.

1-(3-bromophenyl)-2-(pentafluoro-λ^6-sulfaneyl)ethan-1-one

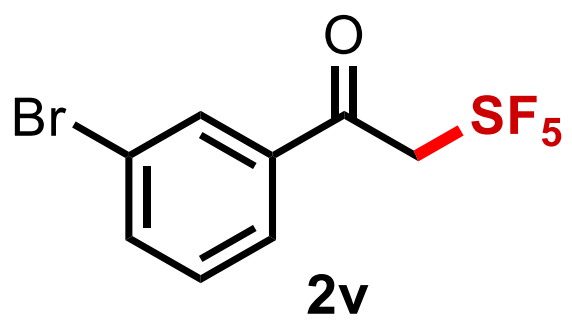

2v

The product **2v** was purified with silica gel chromatography (PE: DCM = 10:1) as a colorless oil (53% yield)

^{1}H NMR (500 MHz, $CDCl_3$) δ 8.13 (t, *J* = 1.9 Hz, 1H), 7.92 (d, *J* = 7.9 Hz, 1H), 7.84 – 7.73 (m, 1H), 7.42 (t, *J* = 7.9 Hz, 1H), 4.85 (p, *J* = 7.8 Hz, 2H).

^{13}C NMR (126 MHz, $CDCl_3$) δ 186.01 (t, *J* = 3.8 Hz), 137.50, 137.15, 132.14, 130.74, 127.75, 123.60, 71.68 (p, *J* = 13.3 Hz).

^{19}F NMR (471 MHz, $CDCl_3$) δ 81.40 – 79.74 (m, 1F), 73.25 – 72.53 (m, 4F).

HRMS (ESI) (*m/z*): $[M+Na]^+$ calcd. for $C_8H_6BrF_5NaOS$: 346.9135, found: 346.9141.

1-(2-fluorophenyl)-2-(pentafluoro-λ^6-sulfaneyl)ethan-1-one

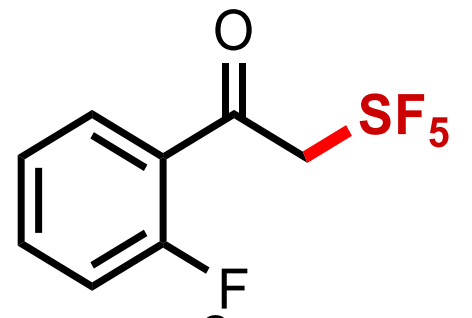

2w

The product **2w** was purified with silica gel chromatography (PE: DCM = 10:1) as a colorless oil (32% yield)

^{1}H NMR (500 MHz, $CDCl_3$) δ 7.87 (td, J = 7.7, 1.8 Hz, 1H), 7.66 – 7.57 (m, 1H), 7.32 – 7.27 (m, 1H), 7.22 – 7.16 (m, 1H), 4.96 (p, J = 7.6 Hz, 2H).

^{13}C NMR (126 MHz, $CDCl_3$) δ 185.36, 162.71, 160.68, 136.27 (d, J = 9.3 Hz), 131.74 (d, J = 1.6 Hz), 125.19 (d, J = 3.3 Hz), 117.04 (d, J = 23.8 Hz), 75.90 – 75.67 (m).

^{19}F NMR (471 MHz, $CDCl_3$) δ 81.57 – 80.11 (m, 1F), 75.00 – 69.37 (m, 4F), -110.12 – -111.95 (m, 1F).

HRMS (ESI) (m/z): $[M+Na]^+$ calcd. for $C_8H_6F_6NaOS$: 286.9936, found: 286.9947.

1-(naphthalen-2-yl)-2-(pentafluoro-λ^6-sulfaneyl)ethan-1-one

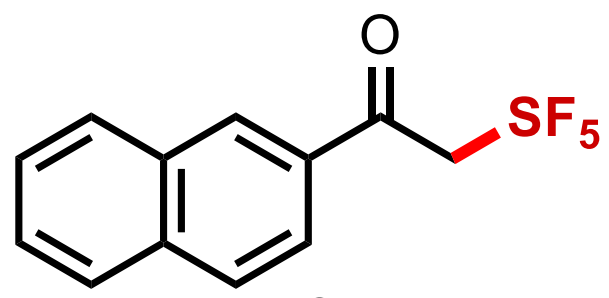

2x

The product **2x** was purified with silica gel chromatography (PE: DCM = 5:1) as a colorless oil (26% yield)

^{1}H NMR (600 MHz, $CDCl_3$) δ 8.55 – 8.49 (m, 1H), 8.09 – 8.00 (m, 2H), 7.93 (dd, J = 26.4, 8.4 Hz, 2H), 7.67 (ddd, J = 8.2, 6.9, 1.3 Hz, 1H), 7.61 (ddd, J = 8.1, 6.8, 1.2 Hz, 1H), 5.01 (p, J = 7.8 Hz, 2H).

^{13}C NMR (151 MHz, $CDCl_3$) δ 187.15, 136.21, 132.99, 132.50, 131.79, 130.09, 129.67, 129.30, 128.03, 127.45, 124.10, 71.92 (p, J = 13.0 Hz).

^{19}F NMR (565 MHz, $CDCl_3$) δ 81.97 – 80.67 (m, 1F), 72.91 – 72.18 (m, 4F).

HRMS (ESI) (m/z): $[M+H]^+$ calcd. for $C_{12}H_{10}F_5OS$: 297.0373, found: 297.0368.

1-(2,3-dihydrobenzo[*b*][1,4]dioxin-6-yl)-2-(pentafluoro-λ^6-sulfaneyl)ethan-1-one

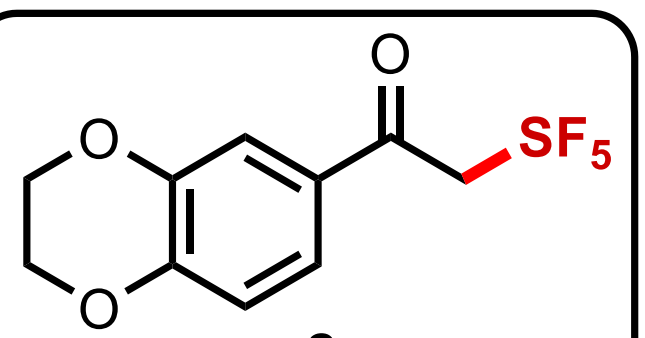

2y

The product **2y** was purified with silica gel chromatography (PE: DCM = 3:1) as a colorless oil (32% yield)

^{1}H NMR (600 MHz, $CDCl_3$) δ 7.56 – 7.52 (m, 2H), 6.98 – 6.94 (m, 1H), 4.79 (p, J = 7.8 Hz, 2H), 4.37 – 4.27 (m, 4H).

^{13}C NMR (151 MHz, $CDCl_3$) δ 185.48 (t, J = 3.0 Hz), 149.53, 143.85, 129.41, 123.70, 118.79, 117.88, 71.76 (t, J = 12.8 Hz), 64.94, 64.18.

^{19}F NMR (565 MHz, $CDCl_3$) δ 82.01 – 80.78 (m, 1F), 72.68 – 72.02 (m, 4F).

HRMS (ESI) (m/z): $[M+H]^+$ calcd. for $C_{10}H_{10}F_5O_3S$: 305.0271, found: 305.0272.

1-(3,5-dimethylphenyl)-2-(pentafluoro-λ^6-sulfaneyl)ethan-1-one

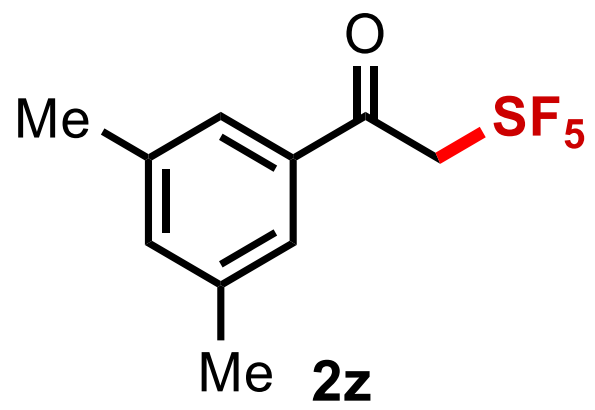

2z

The product **2z**was purified with silica gel chromatography (PE: DCM = 10:1) as a colorless oil (52% yield)

^{1}H NMR (500 MHz, $CDCl_3$) δ 7.60 (s, 2H), 7.29 (s, 1H), 4.85 (p, J = 7.8 Hz, 2H), 2.40 (s, 6H).

^{13}C NMR (126 MHz, $CDCl_3$) δ 187.49, 138.99, 136.38, 135.75, 127.07, 71.89 (p, J = 12.8 Hz), 21.36.

^{19}F NMR (471 MHz, $CDCl_3$) δ 82.18 – 80.27 (m, 1F), 72.97 – 72.00 (m, 4F).

HRMS (ESI) (m/z): $[M+Na]^+$ calcd. for $C_{12}H_{11}F_5OSNa$: 297.0348, found: 297.0352.

1-(3,5-dichlorophenyl)-2-(pentafluoro-λ^6-sulfaneyl)ethan-1-one

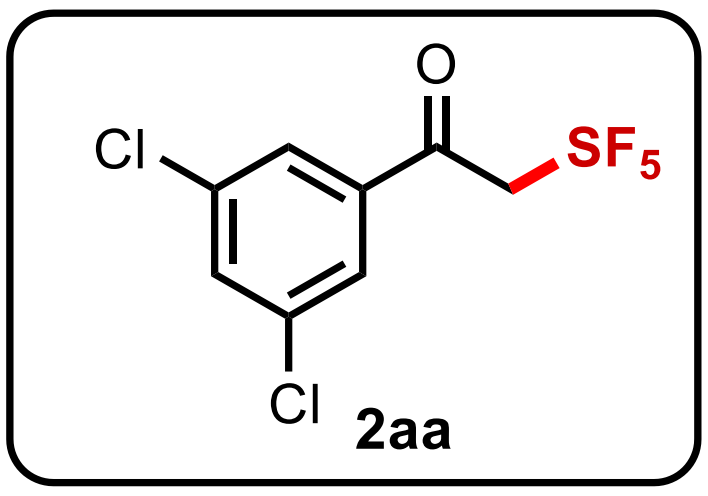

The product **2aa** was purified with silica gel chromatography (PE: DCM = 10:1) as a colorless oil (42% yield)

^{1}H NMR (500 MHz, $CDCl_3$) δ 7.87 – 7.82 (m, 2H), 7.67 – 7.62 (m, 1H), 4.83 (p, J = 7.8 Hz, 1H).

^{13}C NMR (126 MHz, $CDCl_3$) δ 185.02, 137.85, 136.41, 134.31, 127.53, 71.69 (p, J = 13.9 Hz).

^{19}F NMR (471 MHz, $CDCl_3$) δ 80.83 – 79.37 (m, 1F), 73.44 – 72.83 (m, 4F).

HRMS (ESI) (*m/z*): $[M+H]^+$ calcd. for $C_8H_6Cl_2F_5OS$: 314.9431, found: 314.9451.

1-(3-chloro-4-methylphenyl)-2-(pentafluoro-λ^6-sulfaneyl)ethan-1-one

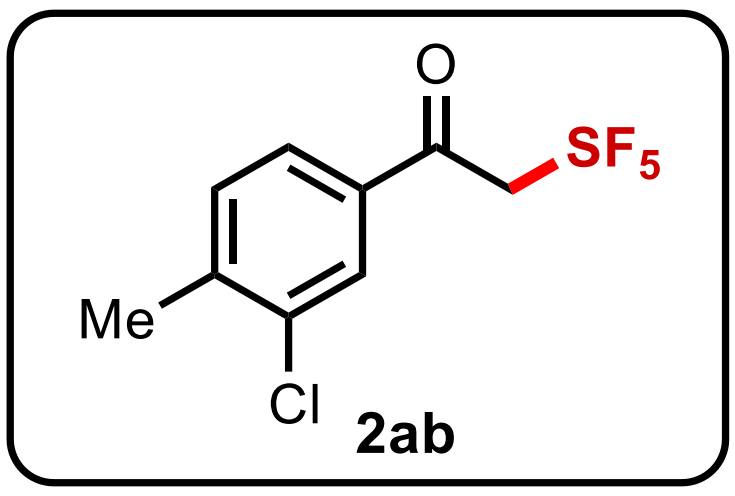

The product **2ab** was purified with silica gel chromatography (PE: DCM = 10:1) as a colorless oil (46% yield)

^{1}H NMR (500 MHz, $CDCl_3$) δ 7.98 (d, J = 1.9 Hz, 1H), 7.78 (dd, J = 8.0, 1.9 Hz, 1H), 7.40 (d, J = 8.0 Hz, 1H), 4.83 (p, J = 7.7 Hz, 2H), 2.46 (s, 3H).

^{13}C NMR (126 MHz, $CDCl_3$) δ 185.82, 143.79, 135.73, 134.79, 131.66, 129.81, 127.40, 71.74 (p, J = 13.9 Hz), 20.69.

^{19}F NMR (471 MHz, $CDCl_3$) δ 81.52 – 80.07 (m, 1F), 73.01 – 72.40 (m, 4F).

HRMS (ESI) (*m/z*): $[M+H]^+$ calcd. for $C_9H_9ClF_5OS$: 294.9977, found: 294.9974.

1-(4-chloro-3-methoxyphenyl)-2-(pentafluoro-λ^6-sulfaneyl)ethan-1-one

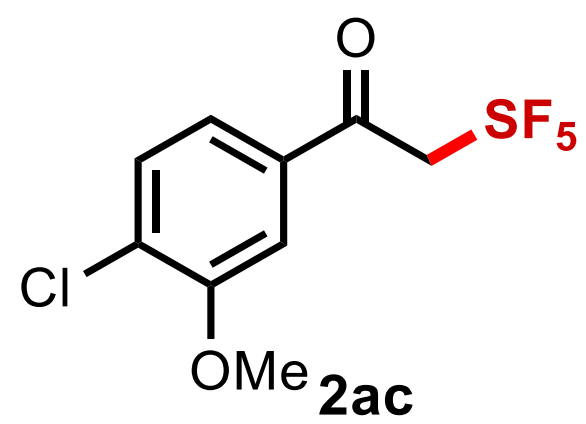

2ac

The product **2ac** was purified with silica gel chromatography (PE: DCM = 10:1) as a colorless oil (39% yield)

^{1}H NMR (500 MHz, $CDCl_3$) δ 7.61 – 7.47 (m, 3H), 4.85 (p, J = 7.8 Hz, 2H), 3.98 (s, 3H).

^{13}C NMR (126 MHz, $CDCl_3$) δ 186.16, 155.85, 135.10, 130.77, 130.22, 122.50, 111.49, 71.71 (p, J = 13.9 Hz), 56.48.

^{19}F NMR (471 MHz, $CDCl_3$) δ 81.65 – 79.98 (m, 1F), 72.93 – 72.36 (m, 4F).

HRMS (ESI) (m/z): $[M+Na]^+$ calcd. for $C_9H_8ClF_5O_2SNa$: 332.9746, found: 332.9720.

1-(3,4-difluorophenyl)-2-(pentafluoro-λ^6-sulfaneyl)ethan-1-one

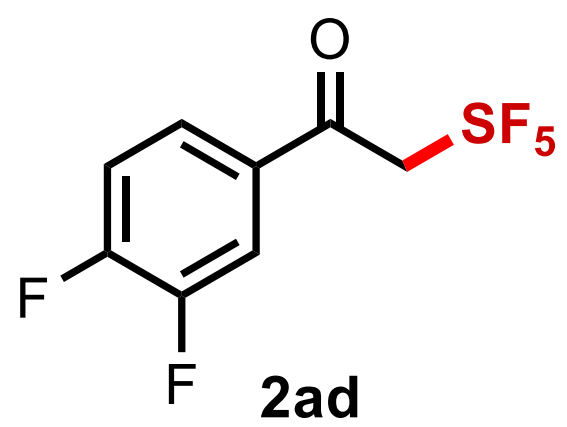

2ad

The product **2ad** was purified with silica gel chromatography (PE: DCM = 10:1) as a colorless oil (52% yield)

^{1}H NMR (500 MHz, $CDCl_3$) δ 7.95 (td, J = 8.7, 6.3 Hz, 1H), 7.03 (ddd, J = 9.4, 7.7, 2.5 Hz, 1H), 6.94 (ddd, J = 11.2, 8.4, 2.4 Hz, 2H), 4.93 (p, J = 7.5 Hz, 2H).

^{13}C NMR (126 MHz, $CDCl_3$) δ 183.92 – 183.71 (m), 166.88 (dd, J = 260.1, 12.8 Hz), 162.57 (dd, J = 257.7, 12.7 Hz), 133.86 (d, J = 3.3 Hz), 121.07 (d, J = 11.7 Hz), 105.05, 75.39 – 75.20 (m).

^{19}F NMR (471 MHz, $CDCl_3$) δ 81.26 – 79.93 (m, 1F), 73.17 – 72.76 (m, 4F), -98.25 – -98.62 (m, 1F), -105.76 – -106.17 (m, 1F).

HRMS (ESI) (m/z): $[M+H]^+$ calcd. for $C_8H_6F_7OS$: 283.0022, found: 283.0017.

(2-(benzyloxy)-2-methoxyethyl)pentafluoro-λ⁶-sulfane

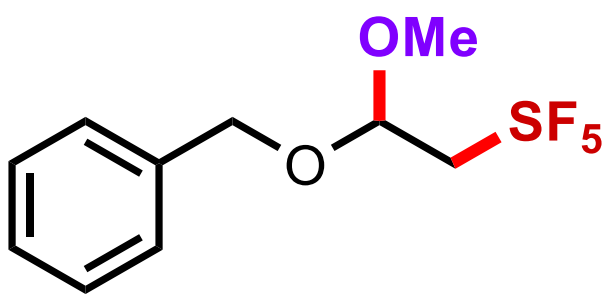

4a

The product **4a** was purified with silica gel chromatography (PE: DCM = 5:1) as a colorless oil (45% yield)

^{1}H NMR (500 MHz, $CDCl_3$) δ 7.41 – 7.31 (m, 5H), 5.06 (t, J = 5.1 Hz, 1H), 4.69 (d, J = 11.4 Hz, 1H), 4.60 (d, J = 11.4 Hz, 1H), 3.85 (pd, J = 8.3, 3.8 Hz, 2H), 3.42 (s, 3H).

^{13}C NMR (126 MHz, $CDCl_3$) δ 136.90, 128.80, 128.24, 128.02, 98.63 (p, J = 5.2 Hz), 72.07 (p, J = 12.5 Hz), 69.23, 53.96.

^{19}F NMR (471 MHz, $CDCl_3$) δ 85.23 – 82.58 (m, 1F), 67.92 (dt, J = 147.4, 8.4 Hz, 1F).

HRMS (ESI) (*m/z*): $[M+Na]^+$ calcd. for $C_{10}H_{13}F_5O_2SNa$: 315.0449, found: 315.0456.

pentafluoro(2-methoxy-2-(3-phenylpropoxy)ethyl)-λ⁶-sulfane

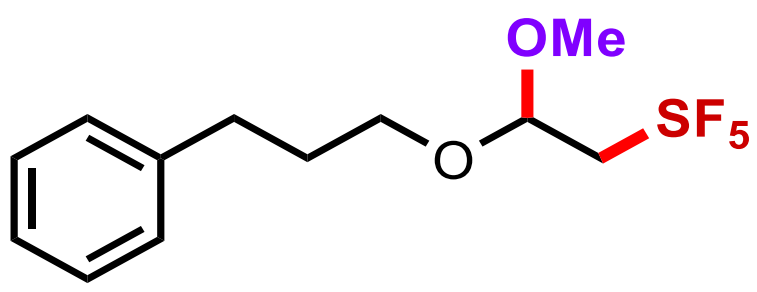

4b

The product **4b** was purified with silica gel chromatography (PE: DCM = 10:1) as a colorless oil (47% yield)

^{1}H NMR (500 MHz, $CDCl_3$) δ 7.29 (dd, *J* = 8.7, 6.7 Hz, 2H), 7.20 (t, *J* = 7.5 Hz, 3H), 4.91 (t, *J* = 5.0 Hz, 1H), 3.78 (pd, *J* = 8.4, 5.0 Hz, 2H), 3.66 (dt, *J* = 9.3, 6.2 Hz, 1H), 3.51 (dt, *J* = 9.3, 6.4 Hz, 1H), 3.39 (s, 3H), 2.71 (td, *J* = 7.4, 2.2 Hz, 2H), 1.98 – 1.87 (m, 2H).

^{13}C NMR (126 MHz, $CDCl_3$) δ 141.68, 128.57, 128.56, 126.09, 99.26 (p, *J* = 5.3 Hz), 72.28 (p, *J* = 12.4 Hz), 66.80, 54.09, 32.35, 31.32.

^{19}F NMR (471 MHz, $CDCl_3$) δ 84.95 – 83.42 (m, 1F), 67.82 (dt, *J* = 147.3, 8.5 Hz, 4F).

HRMS (ESI) (*m/z*): $[M+Na]^+$ calcd. for $C_{12}H_{17}F_5O_2SNa$: 343.0762, found: 343.0732.

pentafluoro(2-methoxy-2-((4-methylbenzyl)oxy)ethyl)-λ^6-sulfane

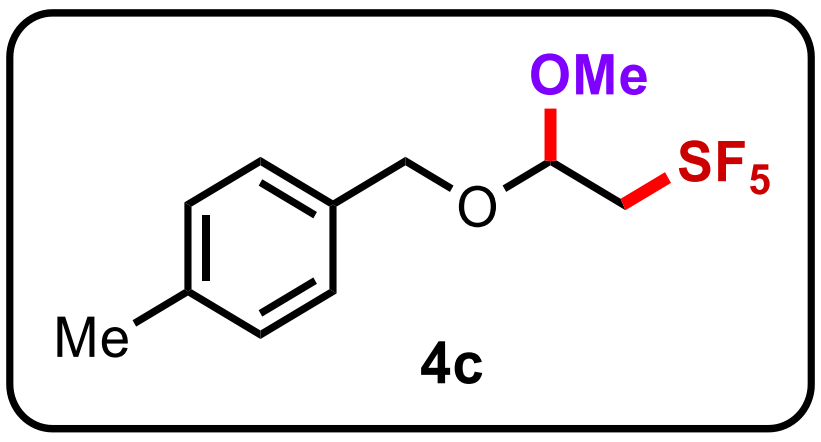

The product **4c** was purified with silica gel chromatography (PE: DCM = 10:1) as a colorless oil (41% yield)

^{1}H NMR (400 MHz, $CDCl_3$) δ 7.28 – 7.15 (m, 4H), 5.03 (t, J = 5.0 Hz, 1H), 4.64 (d, J = 11.2 Hz, 1H), 4.55 (d, J = 11.2 Hz, 1H), 3.83 (pd, J = 9.0, 8.3, 5.6 Hz, 2H), 3.41 (s, 3H), 2.36 (s, 3H).

^{13}C NMR (101 MHz, $CDCl_3$) δ 138.07, 133.82, 129.38, 128.18, 98.47 (p, J = 5.1 Hz), 72.10 (p, J = 12.6 Hz), 69.17, 53.91, 21.33.

^{19}F NMR (376 MHz, $CDCl_3$) δ 85.99 – 82.26 (m, 1F), 67.91 (dt, J = 147.7, 8.5 Hz, 4F).

HRMS (ESI) (*m/z*): $[M+H]^+$ calcd. for $C_{11}H_{16}F_5O_2S$: 307.0786, found: 307.0799.

(2-((3,5-dimethylbenzyl)oxy)-2-methoxyethyl)pentafluoro-λ^6-sulfane

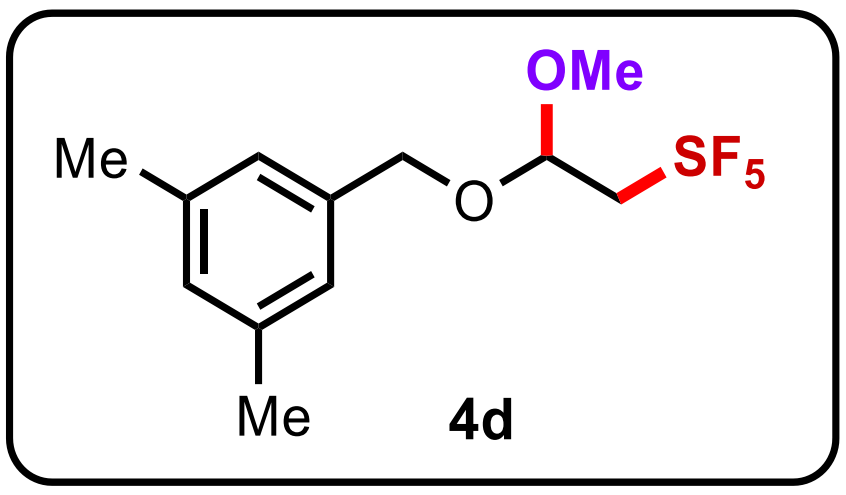

The product **4d** was purified with silica gel chromatography (PE: DCM = 10:1) as a colorless oil (52% yield)

^{1}H NMR (500 MHz, $CDCl_3$) δ 6.96 (s, 3H), 5.04 (t, J = 5.0 Hz, 1H), 4.61 (d, J = 11.2 Hz, 1H), 4.51 (d, J = 11.1 Hz, 1H), 3.84 (pd, J = 8.3, 5.0 Hz, 2H), 3.42 (s, 3H), 2.33 (s, 6H).

^{13}C NMR (126 MHz, $CDCl_3$) δ 138.32, 136.71, 129.88, 125.90, 98.61 (p, J = 5.3 Hz), 72.11 (p, J = 12.4 Hz), 69.29, 53.99, 21.38.

^{19}F NMR (471 MHz, $CDCl_3$) δ 84.77 – 83.32 (m, 1F), 67.92 (dt, J = 147.3, 8.3 Hz, 4F).

HRMS (ESI) (*m/z*): $[M+Na]^+$ calcd. for $C_{12}H_{17}F_5O_2S$: 343.0762, found: 343.0749.

(2-((4-(tert-butyl)benzyl)oxy)-2-methoxyethyl)pentafluoro-λ^6-sulfane

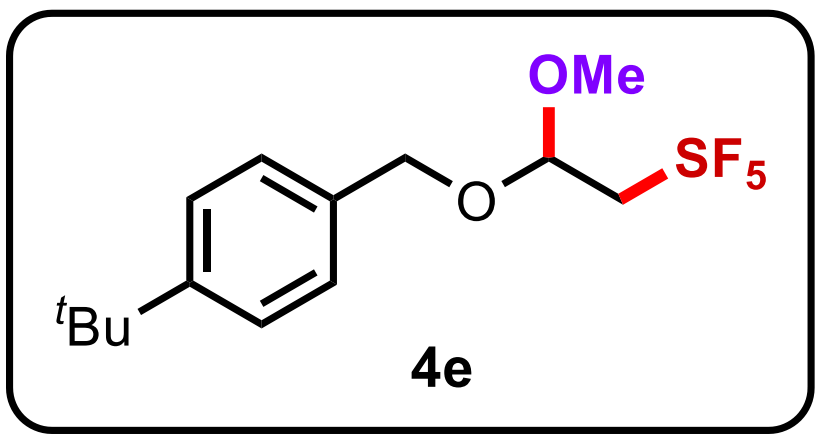

The product **4e** was purified with silica gel chromatography (PE: DCM = 10:1) as a colorless oil (47% yield)

^{1}H NMR (400 MHz, CDCl$_3$) δ 7.41 (d, *J* = 7.9 Hz, 2H), 7.30 (d, *J* = 7.9 Hz, 2H), 5.06 (t, *J* = 5.0 Hz, 1H), 4.66 (d, *J* = 11.2 Hz, 1H), 4.56 (d, *J* = 11.0 Hz, 1H), 3.95 – 3.73 (m, 2H), 3.42 (s, 3H), 1.33 (s, 9H).

^{13}C NMR (101 MHz, CDCl$_3$) δ 151.32, 133.90, 127.89, 125.65, 98.64 (p, *J* = 5.0 Hz), 72.13 (p, *J* = 12.1 Hz), 69.11, 53.93, 34.73, 31.45.

^{19}F NMR (376 MHz, CDCl$_3$) δ 85.00 – 82.97 (m, 1F), 67.94 (dt, *J* = 147.4, 8.4 Hz, 4F).

HRMS (ESI) (*m/z*): $[M+Na]^+$ calcd. for $C_{14}H_{21}F_5O_2S$: 371.1075, found: 371.1058.

(2-([1,1'-biphenyl]-4-ylmethoxy)-2-methoxyethyl)pentafluoro-λ^6-sulfane

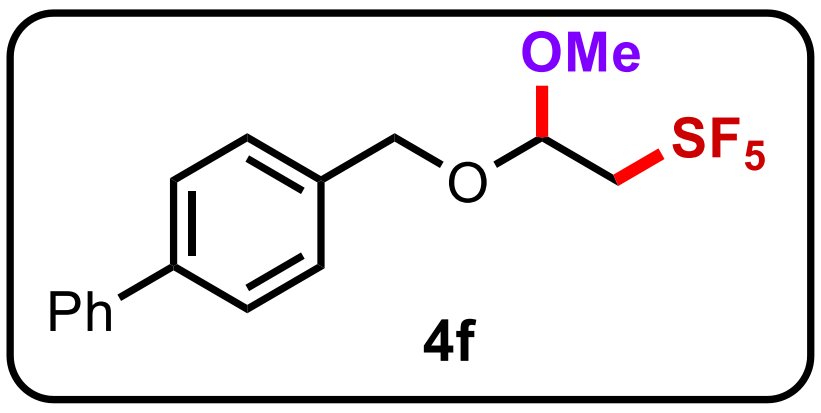

The product **4f** was purified with silica gel chromatography (PE: DCM = 10:1) as a colorless oil (55% yield)

^{1}H NMR (500 MHz, CDCl$_3$) δ 7.65 – 7.58 (m, 4H), 7.45 (q, *J* = 7.7 Hz, 4H), 7.40 – 7.33 (m, 1H), 5.09 (t, *J* = 5.0 Hz, 1H), 4.74 (d, *J* = 11.4 Hz, 1H), 4.64 (d, *J* = 11.4 Hz, 1H), 3.96 – 3.80 (m, 2H), 3.44 (s, 3H).

^{13}C NMR (126 MHz, CDCl$_3$) δ 141.22, 140.86, 135.94, 128.95, 128.47, 127.56, 127.46, 127.26, 98.71 (p, *J* = 5.3 Hz), 72.12 (p, *J* = 12.4 Hz), 68.98, 54.00.

^{19}F NMR (471 MHz, CDCl$_3$) δ 84.79 – 83.23 (m, 1F), 68.01 (dt, *J* = 147.3, 8.4 Hz, 4F).

HRMS (ESI) (*m/z*): $[M+Na]^+$ calcd. for $C_{16}H_{17}F_5O_2SNa$: 391.0762, found: 391.0744.

pentafluoro(2-methoxy-2-(naphthalen-2-ylmethoxy)ethyl)-λ⁶-sulfane

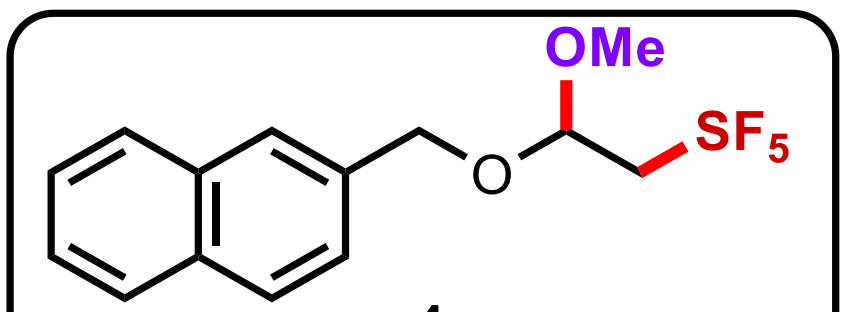

4g

The product **4g** was purified with silica gel chromatography (PE: DCM = 10:1) as a colorless oil (34% yield)

^{1}H NMR (500 MHz, $CDCl_3$) δ 7.88 – 7.79 (m, 4H), 7.53 – 7.45 (m, 3H), 5.10 (t, J = 5.1 Hz, 1H), 4.85 (d, J = 11.6 Hz, 1H), 4.76 (d, J = 11.6 Hz, 1H), 3.94 – 3.83 (m, 2H), 3.44 (s, 3H).

^{13}C NMR (126 MHz, $CDCl_3$) δ 134.37, 133.35, 133.23, 128.54, 128.07, 127.87, 126.88, 126.42, 126.30, 125.81, 98.67 (p, J = 5.0 Hz), 72.12 (p, J = 12.6 Hz), 69.34, 54.04.

^{19}F NMR (471 MHz, $CDCl_3$) δ 84.75 – 83.15 (m, 1F), 68.01 (dt, J = 147.4, 8.3 Hz, 4F).

HRMS (ESI) (m/z): $[M+Na]^+$ calcd. for $C_{14}H_{15}F_5O_2SNa$: 365.0605, found: 365.0589.

pentafluoro(2-methoxy-2-((4-methoxybenzyl)oxy)ethyl)-λ⁶-sulfane

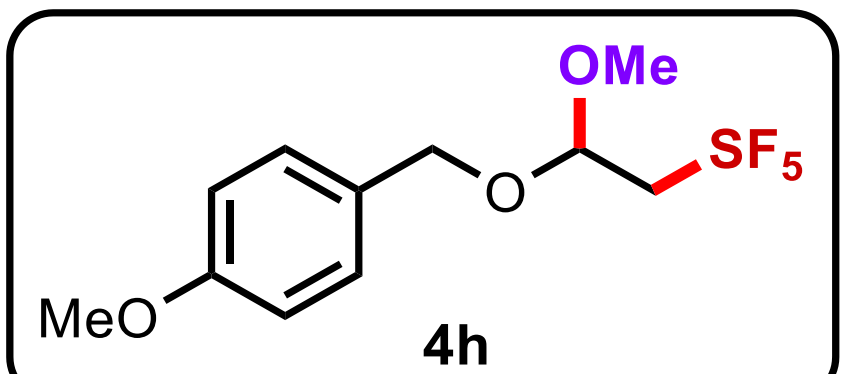

4h

The product **4h** was purified with silica gel chromatography (PE: DCM = 10:1) as a colorless oil (43% yield)

^{1}H NMR (500 MHz, $CDCl_3$) δ 7.29 – 7.26 (m, 2H), 6.93 – 6.88 (m, 2H), 5.01 (t, J = 5.0 Hz, 1H), 4.61 (d, J = 11.1 Hz, 1H), 4.52 (d, J = 11.1 Hz, 1H), 3.81 (s, 5H), 3.39 (s, 3H).

^{13}C NMR (126 MHz, $CDCl_3$) δ 159.66, 129.76, 128.95, 114.09, 98.37 (p, J = 5.0 Hz), 72.14 (p, J = 12.6 Hz), 69.06, 55.43, 53.89.

^{19}F NMR (471 MHz, $CDCl_3$) δ 84.74 – 83.32 (m, 1F), 67.91 (dt, J = 147.5, 8.4 Hz, 4F).

HRMS (ESI) (m/z): $[M+H]^+$ calcd. for $C_{11}H_{16}F_5O_3S$: 323.0735, found: 323.0727.

pentafluoro(2-((4-fluorobenzyl)oxy)-2-methoxyethyl)-λ⁶-sulfane

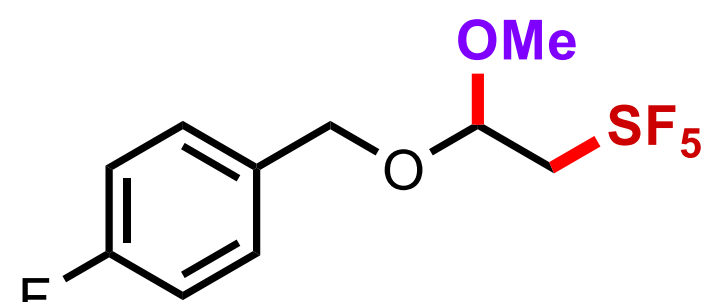

4i

The product **4i** was purified with silica gel chromatography (PE: DCM = 10:1) as a colorless oil (43% yield)

^{1}H NMR (500 MHz, $CDCl_3$) δ 7.35 – 7.29 (m, 2H), 7.09 – 7.02 (m, 2H), 5.03 (t, *J* = 5.1 Hz, 1H), 4.65 (d, *J* = 11.4 Hz, 1H), 4.55 (d, *J* = 11.3 Hz, 1H), 3.83 (pd, *J* = 9.1, 8.3, 3.4 Hz, 2H), 3.40 (s, 3H).

^{13}C NMR (126 MHz, $CDCl_3$) δ 162.68 (d, *J* = 246.3 Hz), 132.72 (d, *J* = 3.2 Hz), 129.82 (d, *J* = 8.3 Hz), 115.59 (d, *J* = 21.6 Hz), 98.59 (p, 5.1 Hz), 72.02 (p, *J* = 12.6 Hz), 68.54, 53.91.

^{19}F NMR (471 MHz, $CDCl_3$) δ 84.93 – 83.11 (m, 1F), 67.92 (dt, *J* = 147.3, 8.3 Hz, 4F), -113.24 – -114.84 (m, 1F).

HRMS (ESI) (*m/z*): $[M+H]^+$ calcd. for $C_{10}H_{13}F_6O_2S$: 311.0535, found: 311.0551.

(2-(3-(4-chlorophenyl)propoxy)-2-methoxyethyl)pentafluoro-λ^6-sulfane

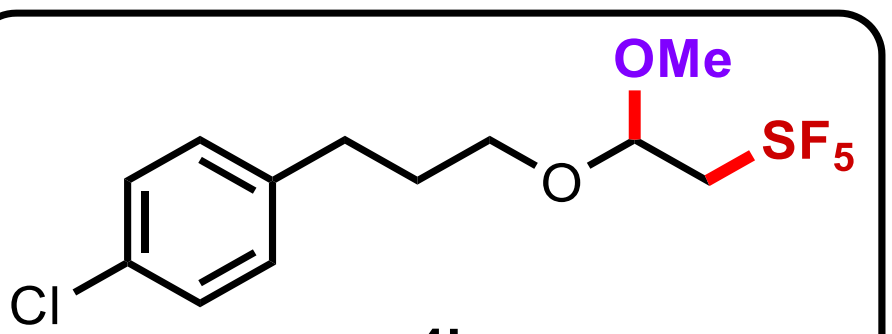

4j

The product **4j** was purified with silica gel chromatography (PE: DCM = 10:1) as a colorless oil (36% yield)

^{1}H NMR (500 MHz, $CDCl_3$) δ 7.30 – 7.27 (m, 2H), 7.17 – 7.13 (m, 2H), 4.93 (t, *J* = 5.0 Hz, 1H), 3.81 (qt, *J* = 8.4, 4.2 Hz, 2H), 3.68 (dt, *J* = 9.2, 6.1 Hz, 1H), 3.56 – 3.49 (m, 1H), 3.42 (s, 3H), 2.71 (td, *J* = 7.5, 2.9 Hz, 2H), 1.97 – 1.88 (m, 2H).

^{13}C NMR (126 MHz, $CDCl_3$) δ 140.10, 131.79, 129.91, 128.64, 99.23 (p, *J* = 5.7 Hz), 72.16 (p, *J* =12.6 Hz), 66.54, 54.08, 31.69, 31.23.

^{19}F NMR (471 MHz, $CDCl_3$) δ 84.90 – 83.39 (m, 1F), 67.82 (dt, *J* = 147.4, 8.6 Hz, 4F).

HRMS (ESI) (*m/z*): $[M+Na]^+$ calcd. for $C_{12}H_{16}ClF_5O_2SNa$: 377.0372, found: 377.0391.

(2-(3-(4-bromophenyl)propoxy)-2-methoxyethyl)pentafluoro-λ⁶-sulfane

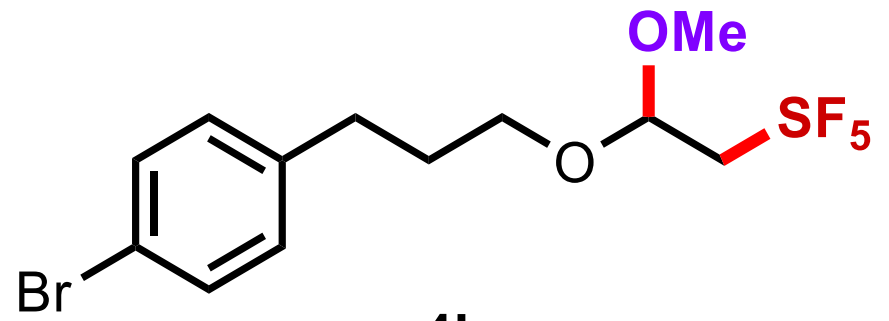

4k

The product **4k** was purified with silica gel chromatography (PE: DCM = 10:1) as a colorless oil (41% yield)

^{1}H NMR (400 MHz, $CDCl_3$) δ 7.41 (d, *J* = 8.1 Hz, 2H), 7.06 (d, *J* = 8.0 Hz, 2H), 4.90 (t, *J* = 5.0 Hz, 1H), 3.83 – 3.72 (m, 2H), 3.69 – 3.62 (m, 1H), 3.52 – 3.45 (m, 1H), 3.38 (s, 3H), 2.70 – 2.63 (m, 2H), 1.94 – 1.85 (m, 2H).

^{13}C NMR (101 MHz, $CDCl_3$) δ 140.64, 131.59, 130.33, 119.81, 99.23 (p, *J* =5.1 Hz), 72.17 (p, *J* = 12.1 Hz), 66.53, 54.07, 31.75, 31.17.

^{19}F NMR (376 MHz, $CDCl_3$) δ 85.12 – 83.16 (m, 1F), 67.84 (dt, *J* = 147.3, 8.3 Hz, 4F).

HRMS (ESI) (*m/z*): $[M+H]^+$ calcd. for $C_{12}H_{17}BrF_5O_2S$: 399.0047, found: 399.0030.

pentafluoro(-2-methoxy-2-(1-phenylpropoxy)ethyl)-λ⁶-sulfane

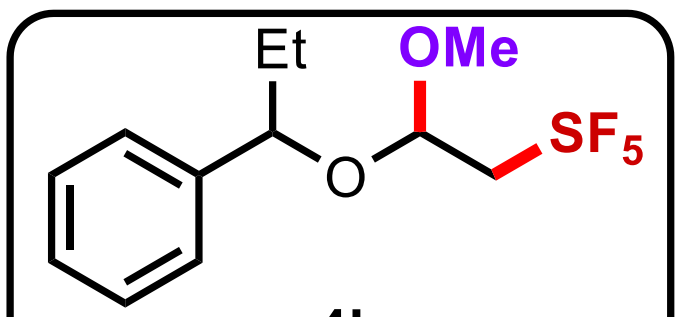

4l

The product **4l** was purified with silica gel chromatography (PE: DCM = 20:1) as a colorless oil (48% yield)

^{1}H NMR (400 MHz, $CDCl_3$) δ 7.39 – 7.30 (m, 3H), 7.30 – 7.25 (m, 2H), 4.78 – 4.72 (m, 1H), 4.47 (t, *J* = 6.9 Hz, 1H), 3.79 – 3.57 (m, 2H), 3.37 (s, 3H), 1.98 – 1.85 (m, 1H), 1.80 – 1.66 (m, 1H), 0.89 (t, *J* = 7.4 Hz, 3H).

^{13}C NMR (101 MHz, $CDCl_3$) δ 140.55, 128.68, 128.32, 127.31, 97.08 (p, *J* =5.5 Hz), 81.19, 72.36 (p, *J* = 12.1 Hz), 53.65, 30.77, 10.41.

^{19}F NMR (376 MHz, $CDCl_3$) δ 84.98 – 83.12 (m, 1F), 68.01 (dt, *J* = 147.2, 8.4 Hz, 4F).

HRMS (ESI) (*m/z*): $[M+Na]^+$ calcd. for $C_{12}H_{17}F_5O_2SNa$: 343.0762, found: 343.0749.

^{1}H NMR (400 MHz, $CDCl_3$) δ 7.39 – 7.28 (m, 5H), 4.87 (t, J = 4.9 Hz, 1H), 4.30 (t, J = 6.7 Hz, 1H), 3.81 – 3.68 (m, 2H), 1.97 – 1.84 (m, 1H), 1.80 – 1.67 (m, 1H), 0.86 (t, J = 7.4 Hz, 3H).

^{13}C NMR (101 MHz, $CDCl_3$) δ 141.68, 128.59, 128.16, 127.11, 98.77 (p, J = 5.3 Hz), 81.60, 72.83 (p, J = 11.1 Hz), 54.82, 30.32, 10.19.

^{19}F NMR (376 MHz, $CDCl_3$) δ 85.22 – 83.25 (m, 1F), 67.50 (dt, J = 147.2, 8.5 Hz, 4F).

HRMS (ESI) (*m/z*): $[M+H]^+$ calcd. for $C_{16}H_{18}F_5O_2S$: 321.0942, found: 321.0975.

(2-(benzhydryloxy)-2-methoxyethyl)pentafluoro-λ⁶-sulfane

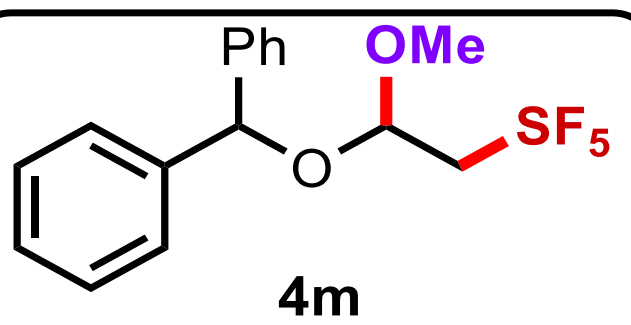

4m

The product **4m** was purified with silica gel chromatography (PE: DCM = 15:1) as a colorless oil (37% yield)

^{1}H NMR (500 MHz, $CDCl_3$) δ 7.41 – 7.25 (m, 10H), 5.70 (s, 1H), 4.98 (t, J = 5.0 Hz, 1H), 3.90 – 3.74 (m, 2H), 3.30 (s, 3H).

^{13}C NMR (126 MHz, $CDCl_3$) δ 141.76, 140.50, 128.76, 128.58, 128.25, 127.78, 127.62, 126.82, 97.41 (p, J = 5.3 Hz), 80.14, 72.32 (p, J = 12.5 Hz), 53.92.

^{19}F NMR (471 MHz, $CDCl_3$) δ 84.71 – 83.12 (m, 1F), 68.10 (dt, J = 147.3, 8.4 Hz, 4F).

HRMS (ESI) (*m/z*): $[M+Na]^+$ calcd. for $C_{16}H_{17}F_5NaO_2S$: 391.0762, found: 391.0758.

(2-([1,1'-biphenyl]-4-ylmethoxy)-2-propoxyethyl)pentafluoro-λ⁶-sulfane

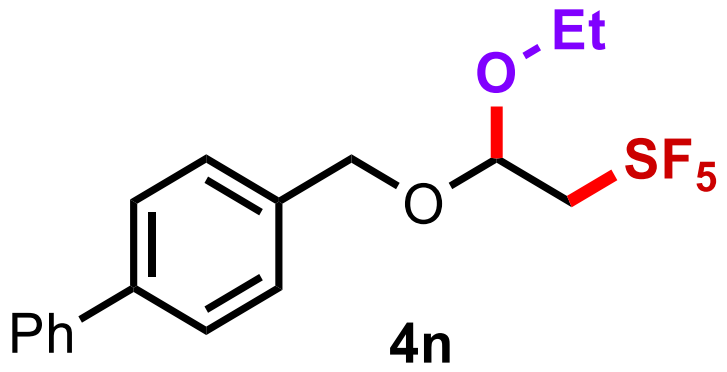

4n

The product **4n** was purified with silica gel chromatography (PE: DCM = 4:1) as a colorless oil (52% yield)

^{1}H NMR (600 MHz, $CDCl_3$) δ 7.63 – 7.58 (m, 4H), 7.48 – 7.35 (m, 5H), 5.14 (t, J = 5.1 Hz, 1H), 4.75 – 4.61 (m, 2H), 3.94 – 3.83 (m, 2H), 3.77 – 3.70 (m, 1H), 3.66 – 3.59 (m, 1H), 1.26 (t, J = 7.0 Hz, 3H).

^{13}C NMR (151 MHz, $CDCl_3$) δ 141.13, 140.86, 136.07, 128.94, 128.42, 127.54, 127.43, 127.25, 97.90 (p, J = 5.2 Hz), 72.66 (p, J = 12.3 Hz), 68.72, 62.95, 15.20.

^{19}F NMR (565 MHz, $CDCl_3$) δ 85.03 – 83.56 (m, 1F), 68.03 (dt, J = 149.2, 9.5 Hz, 4F).

HRMS (ESI) (*m/z*): $[M+H]^+$ calcd. for $C_{18}H_{22}F_5O_2S$: 397.1255, found: 397.1287.

(2-([1,1'-biphenyl]-4-ylmethoxy)-2-isopropoxyethyl)pentafluoro-λ^6-sulfane

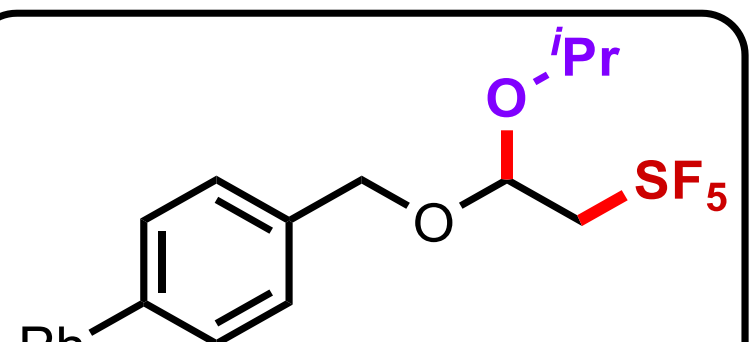

4o

The product **4o** was purified with silica gel chromatography (PE: Et_2O = 30:1) as a colorless oil (39% yield)

^{1}H NMR (400 MHz, $CDCl_3$) δ 7.63 – 7.58 (m, 4H), 7.48 – 7.40 (m, 4H), 7.41 – 7.33 (m, 1H), 5.19 (t, J = 5.0 Hz, 1H), 4.75 – 4.61 (m, 2H), 3.99 – 3.78 (m, 3H), 1.27 (d, J = 6.3 Hz, 3H), 1.20 (d, J = 6.1 Hz, 3H).

^{13}C NMR (101 MHz, $CDCl_3$) δ 141.08, 140.90, 136.24, 128.94, 128.32, 127.52, 127.43, 127.25, 96.74 (p, J = 5.1 Hz), 73.52 (p, J = 11.7 Hz), 70.75, 67.82, 23.21, 22.05.

^{19}F NMR (376 MHz, $CDCl_3$) δ 85.27 – 83.25 (m, 1F), 67.92 (dt, J = 147.3, 8.5 Hz, 4F).

HRMS (ESI) (*m/z*): $[M+Na]^+$ calcd. for $C_{18}H_{21}F_5O_2SNa$: 419.1075, found:419.1064.

(2-([1,1'-biphenyl]-4-ylmethoxy)-2-(cyclopropylmethoxy)ethyl)pentafluoro-λ^6-sulfane

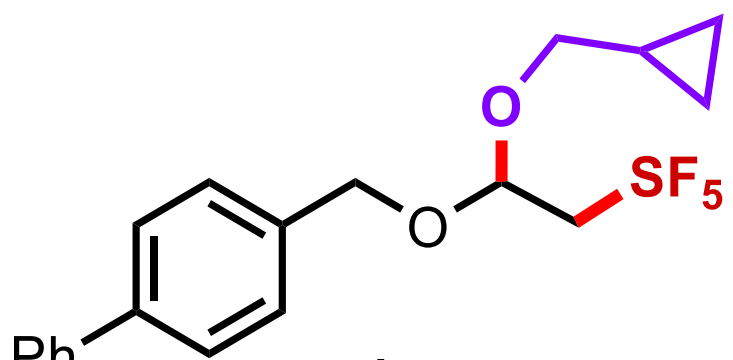

4p

The product **4p** was purified with silica gel chromatography (PE: Et_2O = 30:1) as a colorless oil (37% yield)

^1H NMR (600 MHz, $CDCl_3$) δ 7.59 (d, *J* = 7.6 Hz, 4H), 7.47 – 7.33 (m, 5H), 5.20 – 5.16 (m, 1H), 4.73 (d, *J* = 11.4 Hz, 1H), 4.64 (d, *J* = 11.5 Hz, 1H), 3.97 – 3.83 (m, 2H), 3.51 (t, *J* = 8.7 Hz, 1H), 3.40 (t, *J* = 8.9 Hz, 1H), 1.13 – 1.04 (m, 1H), 0.58 (d, *J* = 8.1 Hz, 2H), 0.24 (d, *J* = 4.9 Hz, 2H).

^{13}C NMR (151 MHz, $CDCl_3$) δ 141.15, 140.87, 136.07, 128.95, 128.40, 127.54, 127.44, 127.26, 97.72 (p, *J* = 4.5 Hz), 72.66 (p, *J* = 12.1 Hz), 72.14, 68.65, 10.59, 3.32, 3.11.

^{19}F NMR (565 MHz, $CDCl_3$) δ 84.90 – 83.56 (m, 1F), 68.06 (dt, *J* = 146.8, 9.0 Hz, 4F).

HRMS (ESI) (*m/z*): $[M+Na]^+$ calcd. for $C_{19}H_{21}F_5O_2SNa$: 431.1075, found: 431.1071.

(2-([1,1'-biphenyl]-4-ylmethoxy)-2-(cyclopentyloxy)ethyl)pentafluoro-λ⁶-sulfane

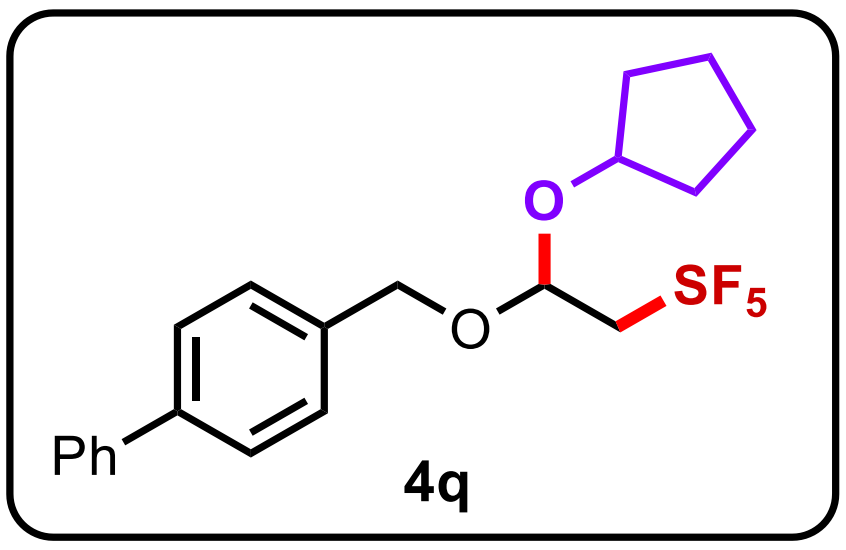

The product **4q** was purified with silica gel chromatography (PE: Et_2O = 30:1) as a colorless oil (27% yield)

^1H NMR (500 MHz, $CDCl_3$) δ 7.62 – 7.53 (m, 4H), 7.47 – 7.33 (m, 5H), 5.18 – 5.11 (m, 1H), 4.71 – 4.58 (m, 2H), 4.24 – 4.17 (m, 1H), 3.92 – 3.78 (m, 2H), 1.90 – 1.58 (m, 8H).

^{13}C NMR (126 MHz, $CDCl_3$) δ 141.09, 140.90, 136.22, 128.94, 128.38, 127.52, 127.43, 127.25, 97.09 (p, *J* = 5.0 Hz), 79.56, 73.36 (p, *J* = 12.6 Hz), 68.13, 33.27, 32.20, 23.46, 23.33.

^{19}F NMR (471 MHz, $CDCl_3$) δ 85.05 – 83.53 (m, 1F), 67.95 (dt, *J* = 147.3, 8.3 Hz, 4F).

HRMS (ESI) (*m/z*): $[M+Na]^+$ calcd. for $C_{20}H_{23}F_5O_2SNa$: 445.1231, found: 445.1226.

(2-([1,1'-biphenyl]-4-ylmethoxy)-2-(2-isopropoxyethoxy)ethyl)pentafluoro-λ⁶-sulfane

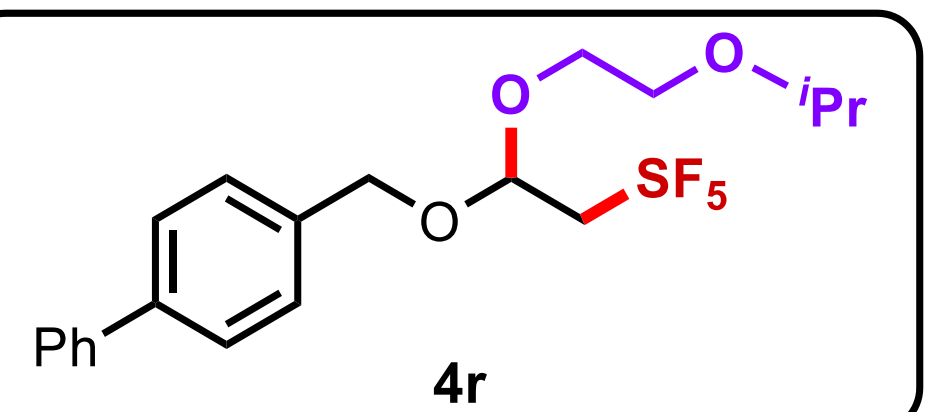

4r

The product **4r** was purified with silica gel chromatography (PE: Et_2O = 30:1) as a colorless oil (37% yield)

^{1}H NMR (500 MHz, $CDCl_3$) δ 7.61 – 7.58 (m, 4H), 7.47 – 7.42 (m, 4H), 7.38 – 7.34 (m, 1H), 5.22 (dd, *J* = 5.8, 4.3 Hz, 1H), 4.79 (d, *J* = 11.4 Hz, 1H), 4.66 (d, *J* = 11.4 Hz, 1H), 4.01 – 3.84 (m, 2H), 3.82 – 3.71 (m, 2H), 3.66 – 3.57 (m, 3H), 1.18 (dd, *J* = 6.1, 1.3 Hz, 6H)..

^{13}C NMR (126 MHz, $CDCl_3$) δ 141.10, 140.93, 136.12, 128.93, 128.51, 127.51, 127.40, 127.26, 98.41 (p, *J* = 5.3 Hz), 72.70 (p, *J* = 12.6 Hz), 72.24, 68.99, 67.42, 66.82, 22.16, 22.14.

^{19}F NMR (471 MHz, $CDCl_3$) δ 84.94 – 83.52 (m, 1F), 68.05 (dt, *J* = 147.3, 8.3 Hz, 4F).

HRMS (ESI) (*m/z*): $[M+Na]^+$ calcd. for $C_{20}H_{25}F_5O_3SNa$: 463.1337, found: 463.1337.

(2-(1-([1,1'-biphenyl]-4-ylmethoxy)-2-(pentafluoro-λ^6-sulfaneyl)ethoxy)ethyl)trimethylsilane

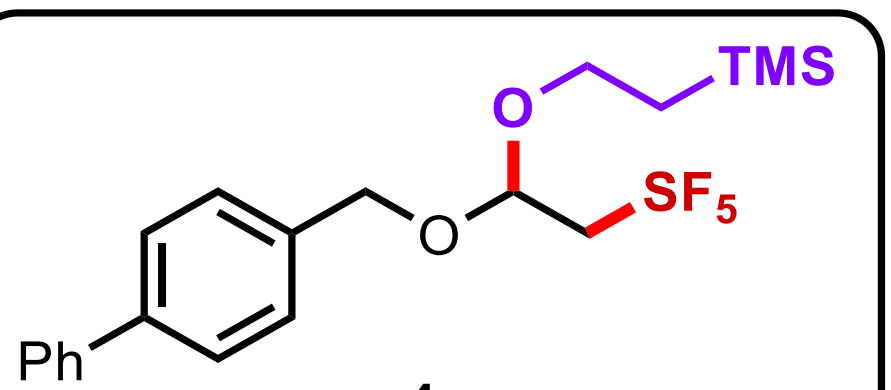

4s

The product **4s** was purified with silica gel chromatography (PE: Et_2O = 30:1) as a colorless oil (26% yield)

^{1}H NMR (500 MHz, $CDCl_3$) δ 7.65 – 7.54 (m, 4H), 7.51 – 7.30 (m, 5H), 5.12 (td, *J* = 5.1, 1.3 Hz, 1H), 4.73 – 4.58 (m, 2H), 3.95 – 3.52 (m, 4H), 1.00 – 0.90 (m, 2H), 0.03 (s, 9H).

^{13}C NMR (126 MHz, $CDCl_3$) δ 141.17, 140.87, 136.15, 128.94, 128.47, 127.54, 127.46, 127.26, 97.75 (p, *J* = 5.0 Hz), 72.80 (p, *J* = 12.6 Hz), 68.68, 65.13, 18.27, -1.29.

^{19}F NMR (471 MHz, $CDCl_3$) δ 85.05 – 83.41 (m, 1F), 67.93 (dt, *J* = 147.6, 8.3 Hz, 4F).

HRMS (ESI) (*m/z*): $[M+Na]^+$ calcd. for $C_{20}H_{27}F_5O_2SSiNa$: 477.1313, found: 477.1314.

(2-([1,1'-biphenyl]-4-ylmethoxy)-2-(2,2-difluoropropoxy)ethyl)pentafluoro-λ⁶-sulfane

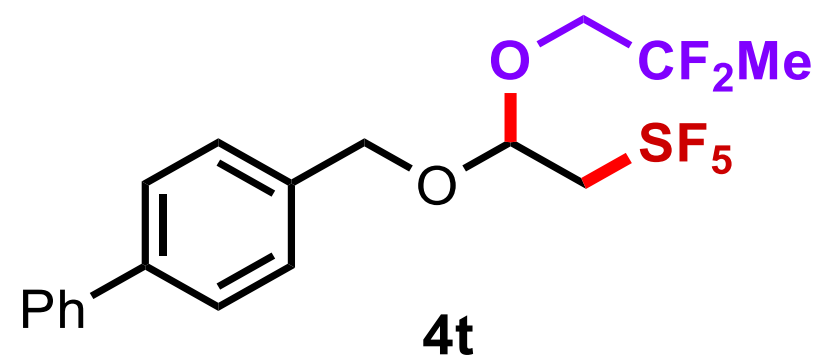

4t

The product **4t** was purified with silica gel chromatography (PE: Et_2O = 30:1) as a colorless oil (45% yield)

^{1}H NMR (500 MHz, $CDCl_3$) δ 7.65 – 7.56 (m, 4H), 7.50 – 7.35 (m, 5H), 5.25 (t, *J* = 5.1 Hz, 1H), 4.75 (d, *J* = 11.4 Hz, 1H), 4.66 (d, *J* = 11.3 Hz, 1H), 3.89 (pd, *J* = 8.1, 5.0 Hz, 2H), 3.81 – 3.67 (m, 2H), 1.68 (t, *J* = 18.8 Hz, 3H).

^{13}C NMR (126 MHz, $CDCl_3$) δ 141.43, 140.76, 135.40, 128.97, 128.57, 127.62, 127.54, 127.27, 121.72 (t, *J* = 239.3 Hz)., 98.11 (p, *J* = 5.5 Hz), 72.01 (p, *J* = 13.1 Hz), 69.30, 67.84 (t, *J* = 33.6 Hz), 20.88 (t, *J* = 26.0 Hz).

^{19}F NMR (471 MHz, $CDCl_3$) δ 84.38 – 82.27 (m, 1F), 68.15 (dt, *J* = 147.4, 8.3 Hz, 4F), -98.14 – -98.44 (m, 2F).

HRMS (ESI) (*m/z*): $[M+Na]^+$ calcd. for $C_{18}H_{19}F_7O_2SNa$: 455.0886, found: 455.0870.

(2-([1,1'-biphenyl]-4-ylmethoxy)-2-(3,3,3-trifluoropropoxy)ethyl)pentafluoro-λ⁶-sulfane

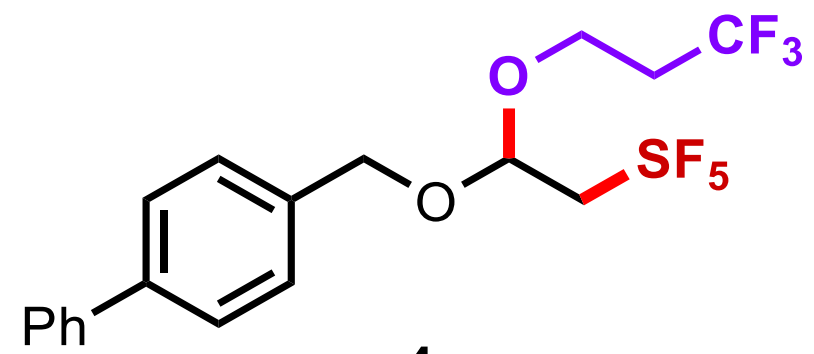

4u

The product **4u** was purified with silica gel chromatography (PE: Et_2O = 30:1) as a colorless oil (44% yield)

^{1}H NMR (500 MHz, $CDCl_3$) δ 7.65 – 7.57 (m, 4H), 7.49 – 7.34 (m, 5H), 5.17 (t, *J* = 5.1 Hz, 1H), 4.75 – 4.62 (m, 2H), 3.97 – 3.83 (m, 3H), 3.75 (dt, *J* = 9.9, 6.5 Hz, 1H), 2.43 (qt, *J* = 10.6, 6.5 Hz, 2H).

^{13}C NMR (126 MHz, $CDCl_3$) δ 141.39, 140.76, 135.60, 128.97, 128.51, 127.62, 127.54, 127.26, 126.01 (q, J = 276.7 Hz), 98.01 (p, J = 5.2 Hz), 72.09 (p, J = 12.8 Hz), 69.01, 59.82 (q, J = 3.7 Hz), 34.35 (q, J = 28.8 Hz).

^{19}F NMR (471 MHz, $CDCl_3$) δ 84.39 – 82.93 (m, 1F), 67.99 (dt, J = 147.4, 8.3 Hz, 4F), -64.76 (t, J = 10.4 Hz, 3F).

HRMS (ESI) (*m/z*): $[M+H]^+$ calcd. for $C_{18}H_{19}F_8O_2S$: 451.0973, found: 451.1008.

(2-(2,2-dimethyl-3-(m-tolyl)propoxy)-2-methoxyethyl)pentafluoro-λ^6-sulfane

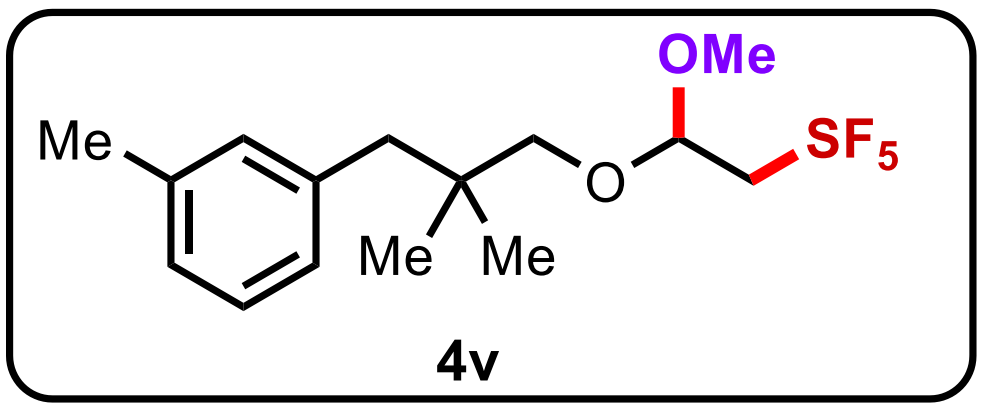

The product **4v** was purified with silica gel chromatography (PE: Et_2O = 30:1) as a colorless oil (46% yield)

^{1}H NMR (500 MHz, $CDCl_3$) δ 7.17 (t, J = 7.5 Hz, 1H), 7.04 (d, J = 7.6 Hz, 1H), 7.00 – 6.91 (m, 2H), 4.92 (t, J = 5.0 Hz, 1H), 3.89 – 3.75 (m, 2H), 3.42 (s, 3H), 3.33 (d, J = 8.5 Hz, 1H), 3.11 (d, J = 8.5 Hz, 1H), 2.62 – 2.51 (m, 2H), 2.34 (s, 3H), 0.92 (d, J = 5.8 Hz, 6H).

^{13}C NMR (126 MHz, $CDCl_3$) δ 138.58, 137.40, 131.51, 127.80, 127.74, 126.86, 99.43 (p, J = 5.1 Hz), 75.57, 72.16 (p, J = 12.4 Hz), 54.21, 44.92, 35.63, 24.69, 24.53, 21.52.

^{19}F NMR (471 MHz, $CDCl_3$) δ 85.26 – 83.25 (m, 1F), 67.86 (dt, J = 147.3, 8.6 Hz, 4F).

HRMS (ESI) (*m/z*): $[M+Na]^+$ calcd. for $C_{15}H_{23}F_5O_2SNa$: 385.1231, found: 385.1209.

pentafluoro(-2-methoxy-2-(((1R,2S,4R)-1,7,7-trimethylbicyclo[2.2.1]heptan-2-yl)oxy)ethyl)-λ^6-sulfane

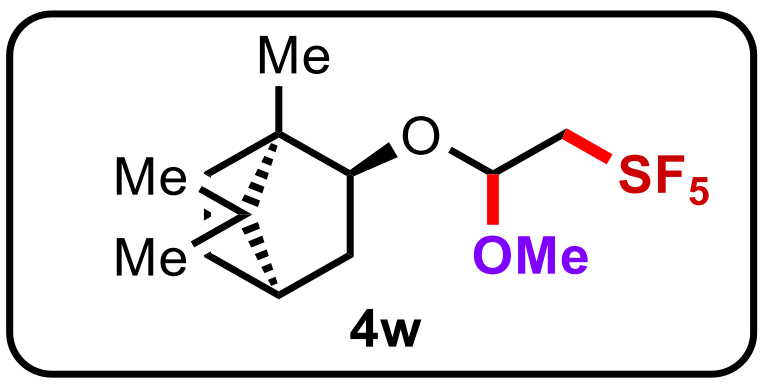

The product **4w** was purified with silica gel chromatography as a colorless oil (45% yield)

^{1}H NMR (500 MHz, $CDCl_3$) δ 4.93 (dt, *J* = 43.1, 5.1 Hz, 1H), 3.97 – 3.72 (m, 3H), 3.36 (d, *J* = 21.6 Hz, 3H), 2.24 – 2.06 (m, 1H), 2.02 – 1.91 (m, 1H), 1.77 – 1.63 (m, 2H), 1.28 – 1.18 (m, 2H), 1.17 – 1.01 (m, 1H), 0.95 – 0.79 (m, 9H).

^{13}C NMR (126 MHz, $CDCl_3$) δ 100.16 (p, *J* = 10.6, 5.4 Hz), 97.28 (p, *J* = 10.1, 5.1 Hz), 84.14, 81.54, 72.77 (p, J = 12.6 Hz), 72.68 (p, J = 12.6 Hz), 53.96, 52.48, 49.61, 49.31, 47.78, 47.67, 45.13, 45.08, 37.27, 34.92, 26.63, 26.61, 19.82, 19.00, 18.93, 13.69.

^{19}F NMR (471 MHz, $CDCl_3$) δ 85.08 – 83.59 (m, 1F), 68.12 (dt, *J* = 147.3, 8.4 Hz, 1.84F), 67.66 (dt, *J* = 147.2, 8.4 Hz, 2.16F)

HRMS (ESI) (*m/z*): $[M+Na]^+$ calcd. for $C_{13}H_{23}F_5O_2SNa$: 361.1231, found: 361.1232.

pentafluoro(-2-(((1S,2R,5S)-2-isopropyl-5-methylcyclohexyl)oxy)-2-methoxyethyl)-λ^6-sulfane

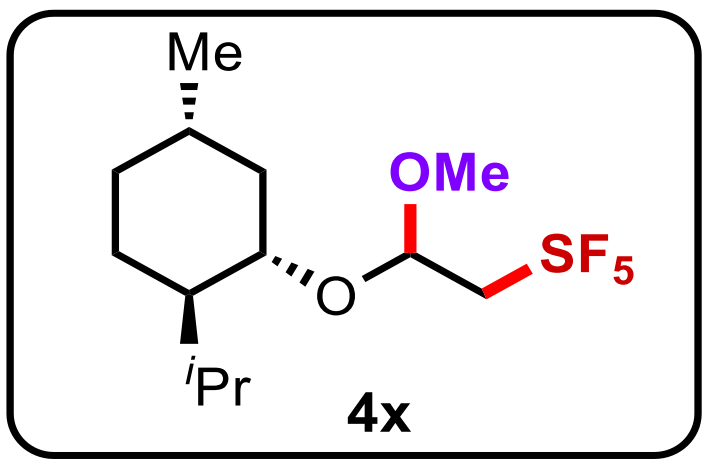

The product **4x** was purified with silica gel chromatography as a colorless oil (47% yield)

^{1}H NMR (600 MHz, $CDCl_3$) δ 5.04 (t, *J* = 4.8 Hz, 1H), 3.87 – 3.67 (m, 2H), 3.43 (td, *J* = 10.9, 4.0 Hz, 1H), 3.39 (d, *J* = 2.0 Hz, 3H), 2.26 (p, *J* = 7.2 Hz, 1H), 2.02 (d, *J* = 12.4 Hz, 1H), 1.70 – 1.62 (m, 2H), 1.40 – 1.31 (m, 1H), 1.27 – 1.20 (m, 1H), 1.03 – 0.82 (m, 10H), 0.77 (d, *J* = 6.9 Hz, 3H).

^{13}C NMR (151 MHz, $CDCl_3$) δ 96.62 (p, *J* = 5.0 Hz), 73.17 (p, *J* = 11.8 Hz), 53.90, 48.15, 40.18, 34.36, 31.53, 25.19, 23.07, 22.41, 21.24, 15.88.

^{19}F NMR (565 MHz, $CDCl_3$) δ 85.09 – 83.84 (m, 1F), 67.94 (dt, *J* = 147.3, 8.6 Hz, 4F).

^{1}H NMR (600 MHz, $CDCl_3$) δ 5.04 – 4.81 (m, 1H), 3.83 – 3.67 (m, 2H), 3.39 (s, 3H), 3.29 – 3.20 (m, 1H), 2.18 – 2.10 (m, 2H), 1.68 – 1.64 (m, 2H), 1.40 – 1.35 (m, 1H), 1.26 – 1.21 (m, 1H), 1.01 – 0.88 (m, 10H), 0.75 (d, *J* = 6.9 Hz, 3H).

^{13}C NMR (151 MHz, $CDCl_3$) δ 99.16 (p, *J* = 6.0 Hz), 79.05, 72.58 (p, *J* = 11.9 Hz), 53.65, 48.36, 42.00, 34.30, 31.71, 25.04, 22.94, 22.40, 21.33, 15.76.

^{19}F NMR (565 MHz, $CDCl_3$) δ 85.04 – 83.63 (m, 1F), 67.47 (dt, *J* = 147.3, 8.6 Hz, 4F).

HRMS (ESI) (*m/z*): $[M+H]^+$ calcd. for $C_{13}H_{26}F_5O_2S$: 341.1568, found: 341.1588.

(3a*S*,5a*R*,8a*R*,8b*S*)-3a-((-1-methoxy-2-(pentafluoro-λ^6-sulfaneyl)ethoxy)methyl)-2,2,7,7-tetramethyltetrahydro-5H-bis([1,3]dioxolo)[4,5-b:4',5'-*d*]pyran

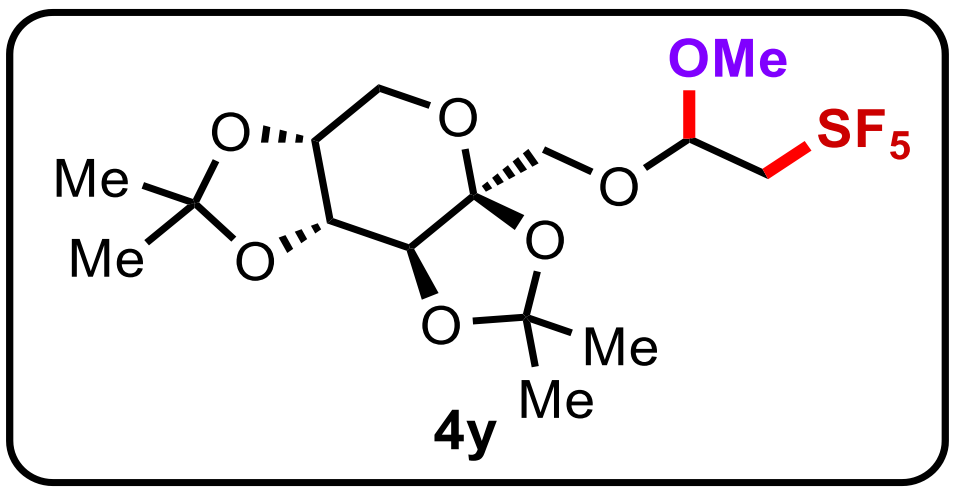

4y

The product **4y** was purified with silica gel chromatography as a colorless oil (39% yield)

^{1}H NMR (400 MHz, $CDCl_3$) δ 5.04 – 4.96 (m, 1H), 4.60 (dd, *J* = 7.9, 2.7 Hz, 1H), 4.34 (dd, *J* = 6.6, 2.6 Hz, 1H), 4.24 (d, *J* = 7.9 Hz, 1H), 3.95 – 3.63 (m, 6H), 3.42 (d, *J* = 6.6 Hz, 3H), 1.54 (s, 3H), 1.46 (s, 3H), 1.39 (d, *J* = 6.2 Hz, 3H), 1.34 (s, 3H).

^{13}C NMR (101 MHz, $CDCl_3$) δ 109.10 (d, *J* = 1.9 Hz), 108.86 (d, *J* = 3.1 Hz), 102.07 (d, *J* = 4.8 Hz), 99.32 – 98.66 (m), 72.13 – 71.43 (m), 71.01, 70.10 (d, *J* = 5.5 Hz), 67.91, 67.56, 61.18 (d, *J* = 2.2 Hz), 54.53 (d, *J* = 57.2 Hz), 26.67, 25.94 (d, *J* = 2.7 Hz), 25.33 (d, *J* = 8.5 Hz), 24.08 (d, *J* = 2.3 Hz).

^{19}F NMR (376 MHz, $CDCl_3$) δ 84.83 – 82.95 (m, 2F), 67.92 (dq, *J* = 147.2, 8.2 Hz, 8F).

HRMS (ESI) (*m/z*): $[M+Na]^+$ calcd. for $C_{15}H_{25}F_5O_7SNa$: 467.1133, found: 467.1146.

(2-(((3R,8R,9R,10S,13S,14R,17S)-10,13-dimethyl-17-((S)-6-methylheptan-2-yl)-2,3,4,7,8,9,10,11,12,13,14,15,16,17-tetradecahydro-1H-cyclopenta[a]phenanthren-3-yl)oxy)-2-methoxyethyl)pentafluoro-λ^6-sulfane

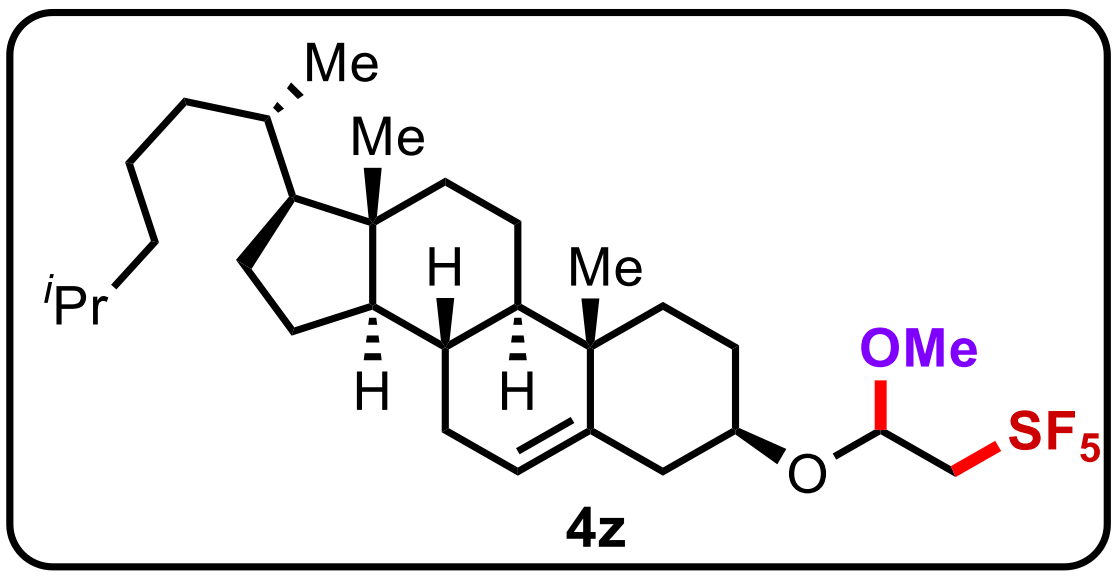

4z

The product **4z** was purified with silica gel chromatography (PE) as a white solid (30% yield)

^{1}H NMR (500 MHz, $CDCl_3$) δ 5.42 – 5.32 (m, 1H), 5.00 (t, *J* = 5.0 Hz, 1H), 3.83 – 3.68 (m, 2H), 3.49 – 3.40 (m, 1H), 3.37 (d, *J* = 2.4 Hz, 3H), 2.37 – 2.21 (m, 2H), 2.04 – 1.94 (m, 2H), 1.90 – 1.79 (m, 3H), 1.63 – 1.43 (m, 8H), 1.38 – 1.23 (m, 5H), 1.19 – 1.03 (m, 8H), 1.01 (s, 3H), 0.91 (d, *J* = 6.5 Hz, 3H), 0.86 (dd, *J* = 6.7, 2.3 Hz, 6H), 0.67 (s, 3H).

^{13}C NMR (126 MHz, $CDCl_3$) δ 140.37 (d, *J* = 27.8 Hz), 122.38 (d, *J* = 13.8 Hz), 98.82 – 96.75 (m), 78.02 (d, *J* = 7.4 Hz), 73.03 (p, *J* = 12.6 Hz), 56.87, 56.28, 53.34 (d, *J* = 2.9 Hz), 50.27 (d, *J* = 4.2 Hz), 42.45, 39.87 (d, *J* = 4.0 Hz), 39.65, 39.08, 37.26 (d, *J* = 20.2 Hz), 36.82, 36.32, 35.93, 32.02 (d, *J* = 9.2 Hz), 29.30, 28.42 (d, *J* = 10.4 Hz), 28.16, 24.42, 23.96, 22.97, 22.71, 21.19, 19.48, 18.85, 12.00.

^{19}F NMR (471 MHz, $CDCl_3$) δ 85.06 – 83.53 (m, 2F), 67.76 (dt, *J* = 147.5, 8.7 Hz, 8F).

HRMS (ESI) (*m/z*): $[M+Na]^+$ calcd. for $C_{30}H_{51}F_5O_2SNa$: 539.3422, found: 539.3408.

(3-(2-([1,1'-biphenyl]-4-yl)-3,3-difluoroallyl)bicyclo[1.1.1]pentan-1-yl)pentafluoro-λ^6-sulfane

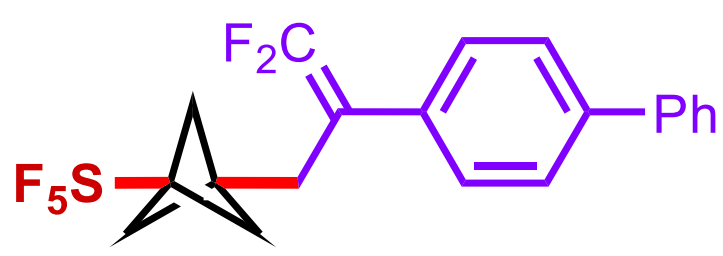

6a

The product **6a** was purified with HPLC Thermo Scientific UltiMate 3000 (Shimadzu Shim-pack PRC-ODS) (MeCN) as a colorless oil (51% yield)

^{1}H NMR (400 MHz, DMSO-d_6) δ 7.68 – 7.63 (m, 4H), 7.47 – 7.40 (m, 4H), 7.35 – 7.30 (m, 1H), 2.76 (t, *J* = 2.4 Hz, 2H), 2.07 (s, 6H)

^{13}C NMR (126 MHz, DMSO-d_6) δ 153.78 (dd, *J* = 294.8, 287.0 Hz), 139.41, 139.36, 131.90 (t, *J* = 4.1 Hz), 129.09, 128.73 (t, *J* = 3.5 Hz), 127.77, 126.83, 126.70, 89.79 (dd, *J* = 21.6, 13.3 Hz), 66.91 (p, *J* = 12.6 Hz), 55.03, 33.99, 27.31.

^{19}F NMR (471 MHz, DMSO-d_6) δ 83.81 – 80.83 (m, 1F), 49.41 (d, *J* = 145.7 Hz, 4F), -90.05 (d, *J* = 41.1 Hz, 1F), -90.97 (d, *J* = 41.3 Hz, 1F).

HRMS (ESI) (*m/z*): $[M+H]^+$ calcd. for $C_{20}H_{18}F_7S$: 423.1012, found: 423.1020.

(3-(3,3-difluoro-2-phenylallyl)bicyclo[1.1.1]pentan-1-yl)pentafluoro-λ^6-sulfane

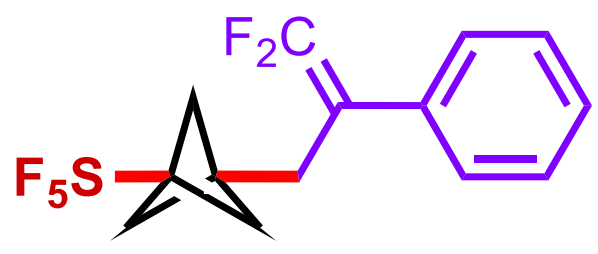

6b

The product **6b** was purified with HPLC Thermo Scientific UltiMate 3000 (Shimadzu Shim-pack PRC-ODS) (MeCN) as a colorless oil (35% yield)

^{1}H NMR (500 MHz, DMSO-d_6) δ 7.35 (d, J = 4.4 Hz, 4H), 7.30 – 7.23 (m, 1H), 2.72 (t, J = 2.3 Hz, 2H), 2.02 (s, 6H).

^{13}C NMR (126 MHz, DMSO-d_6) δ 153.65 (dd, J = 290.4, 286.7 Hz), 132.92 – 132.71 (m), 128.69, 128.22 (d, J = 3.8 Hz), 127.83, 90.13 (dd, J = 21.3, 13.9 Hz), 66.89 (p, J = 12.6 Hz), 55.01, 33.98, 27.43.

^{19}F NMR (376 MHz, DMSO-d_6) δ 83.60 – 81.04 (m, 1F), 49.41 (d, J = 146.3 Hz, 4F), -90.80 (d, J = 40.9 Hz, 1F), -91.59 (d, J = 41.4 Hz, 1F).

HRMS (ESI) (m/z): $[M+H]^+$ calcd. for $C_{14}H_{14}F_7S$: 347.0699, found: 347.0685.

(3-(3,3-difluoro-2-(naphthalen-2-yl)allyl)bicyclo[1.1.1]pentan-1-yl)pentafluoro-λ^6-sulfane

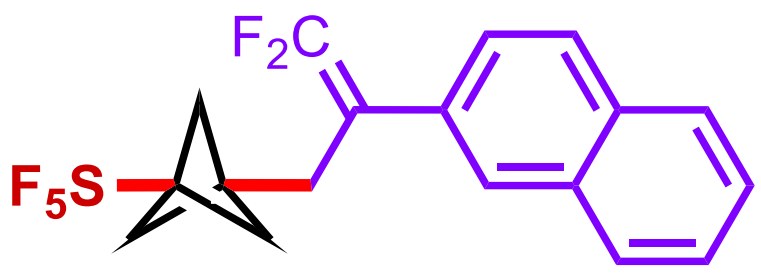

6c

The product **6c** was purified with HPLC Thermo Scientific UltiMate 3000 (Shimadzu Shim-pack PRC-ODS) (MeCN) as a colorless oil (45% yield)

^{1}H NMR (400 MHz, DMSO-d_6) δ 7.98 – 7.77 (m, 4H), 7.55 – 7.39 (m, 3H), 2.81 (s, 2H), 2.01 (s, 6H).

^{13}C NMR (101 MHz, DMSO-d_6) δ 153.93 (dd, *J* = 291.2, 286.7 Hz), 132.90, 132.23, 130.30, 128.20, 128.03, 127.64, 127.24, 126.63, 126.59, 126.00, 90.23 (dd, *J* = 21.1, 13.1 Hz), 66.90 (p, *J* = 12.6 Hz), 55.01, 34.02, 27.50.

^{19}F NMR (376 MHz, DMSO-d_6) δ 84.38 – 80.24 (m, 1F), 49.46 (d, *J* = 146.6 Hz, 4F), -90.03 (d, *J* = 40.8 Hz, 1F), -90.97 (d, *J* = 40.8 Hz, 1F).

HRMS (ESI) (*m/z*): $[M+H]^+$ calcd. for $C_{18}H_{16}F_7S$: 397.0855, found: 397.0854.

(3-(3,3-difluoro-2-(phenanthren-9-yl)allyl)bicyclo[1.1.1]pentan-1-yl)pentafluoro-λ^6-sulfane

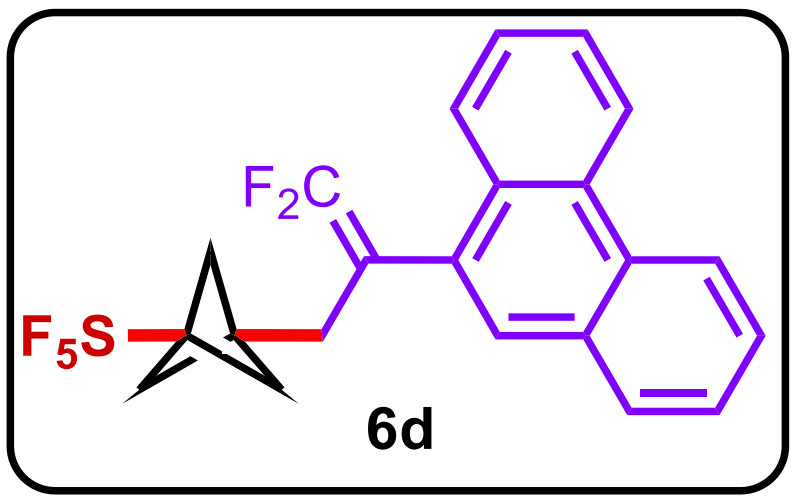

The product **6d** was purified with HPLC Thermo Scientific UltiMate 3000 (Shimadzu Shim-pack PRC-ODS) (MeCN) as a colorless oil (50% yield)

^1H NMR (400 MHz, DMSO-d_6) δ 8.91 – 8.85 (m, 1H), 8.82 (d, *J* = 8.2 Hz, 1H), 8.05 – 7.98 (m, 1H), 7.97 – 7.88 (m, 2H), 7.77 – 7.61 (m, 4H), 3.08 – 2.90 (m, 1H), 2.75 – 2.62 (m, 1H), 2.12 (d, *J* = 10.6 Hz, 6H).

^{13}C NMR (101 MHz, DMSO-d_6) δ 153.75 (dd, *J* = 289.9, 287.9 Hz), 130.82, 130.28, 129.73, 129.35, 128.86 (d, *J* = 5.0 Hz), 128.71, 128.63, 127.56, 127.29, 127.24, 127.14, 125.43, 123.66, 122.88, 88.14 (dd, *J* = 21.9, 18.1 Hz), 79.22, 67.08 (p, *J* = 12.6 Hz), 55.07, 33.93, 29.19.

^{19}F NMR (376 MHz, DMSO-d_6) δ 82.42 (p, *J* = 146.3, 145.7 Hz, 1F), 49.51 (d, *J* = 145.9 Hz, 4F), -87.73 (d, *J* = 40.7 Hz, 1F), -90.93 (d, *J* = 40.5 Hz, 1F).

HRMS (ESI) (*m/z*): $[M+Na]^+$ calcd. for $C_{22}H_{17}F_7SNa$: 469.0831, found: 469.0822.

3-(1,1-difluoro-3-(3-(pentafluoro-λ^6-sulfaneyl)bicyclo[1.1.1]pentan-1-yl)prop-1-en-2-yl)quinoline

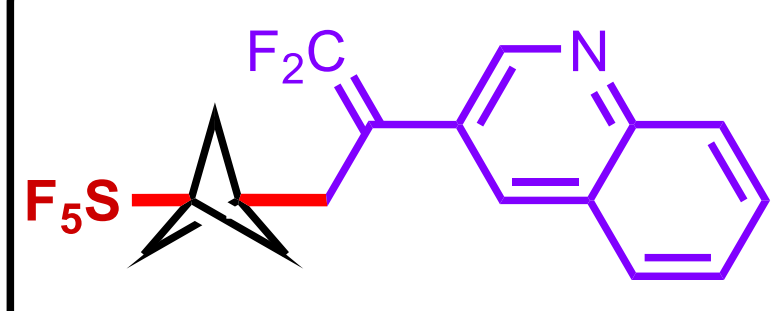

6e

The product **6e** was purified with HPLC Thermo Scientific UltiMate 3000 (Shimadzu Shim-pack PRC-ODS) (MeCN) as a colorless oil (28% yield)

^{1}H NMR (400 MHz, DMSO-d_6) δ 8.92 (t, J = 2.2 Hz, 1H), 8.38 (d, J = 2.3 Hz, 1H), 7.96 (ddd, J = 12.4, 8.4, 1.2 Hz, 2H), 7.73 (ddd, J = 8.4, 6.8, 1.5 Hz, 1H), 7.59 (ddd, J = 8.1, 6.8, 1.2 Hz, 1H), 2.87 (t, J = 2.4 Hz, 2H), 2.07 (s, 6H).

^{13}C NMR (101 MHz, DMSO-d_6) δ 155.72 (dd, J = 292.9, 284.8 Hz), 150.16, 146.63, 134.97, 130.12, 128.79, 128.31, 127.33, 127.26, 126.34, 87.86 (dd, J = 23.5, 14.9 Hz), 66.93 (p, J = 12.6 Hz), 54.97, 33.93, 27.23.

^{19}F NMR (376 MHz, DMSO-d_6) δ 82.42 (p, J = 146.2, 145.5 Hz, 1F), 49.53 (d, J = 146.1 Hz, 4F), -88.56 (d, J = 38.1 Hz, 1F), -89.71 (d, J = 38.2 Hz, 1F).

HRMS (ESI) (m/z): $[M+H]^+$ calcd. for $C_{17}H_{15}F_7NS$: 398.0808, found: 398.0802

3-(1,1-difluoro-3-(3-(pentafluoro-λ⁶-sulfaneyl)bicyclo[1.1.1]pentan-1-yl)prop-1-en-2-yl)pyridine

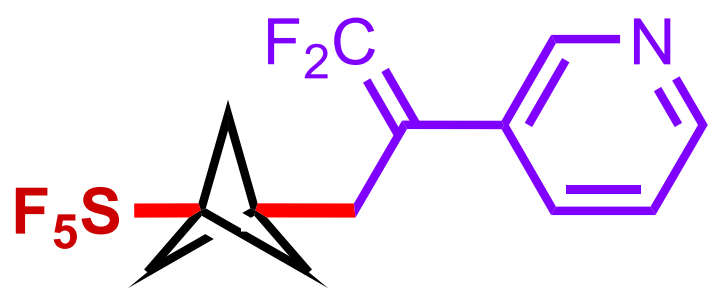

6f

The product **6f** was purified with HPLC Thermo Scientific UltiMate 3000 (Shimadzu Shim-pack PRC-ODS) (MeCN) as a colorless oil (30% yield)

^{1}H NMR (500 MHz, DMSO-d_6) δ 8.64 (t, J = 2.0 Hz, 1H), 8.51 (dd, J = 4.8, 1.6 Hz, 1H), 7.84 (d, J = 8.0 Hz, 1H), 7.43 (dd, J = 8.0, 4.8 Hz, 1H), 2.80 (t, J = 2.4 Hz, 2H), 2.10 (s, 6H).

^{13}C NMR (126 MHz, DMSO-d_6) δ 153.11 (dd, J =293.6, 289.8 Hz,), 149.05 (d, J = 3.6 Hz), 148.73, 135.68, 128.98, 123.62, 87.63 (dd, J = 23.1, 13.8 Hz), 66.88 (p, J = 12.6 Hz), 54.92, 33.82, 27.01.

^{19}F NMR (471 MHz, DMSO-d_6) δ 83.14 – 81.62 (m, 1F), 49.53 (d, *J* = 145.9 Hz, 4F), -89.07 (d, *J* = 38.3 Hz, 1F), -90.22 (d, *J* = 39.1 Hz, 1F).

HRMS (ESI) (*m/z*): $[M+H]^+$ calcd. for $C_{13}H_{13}F_7NS$: 348.0651, found: 348.0665.

(3-(2-(4-bromophenyl)-3,3-difluoroallyl)bicyclo[1.1.1]pentan-1-yl)pentafluoro-λ⁶-sulfane

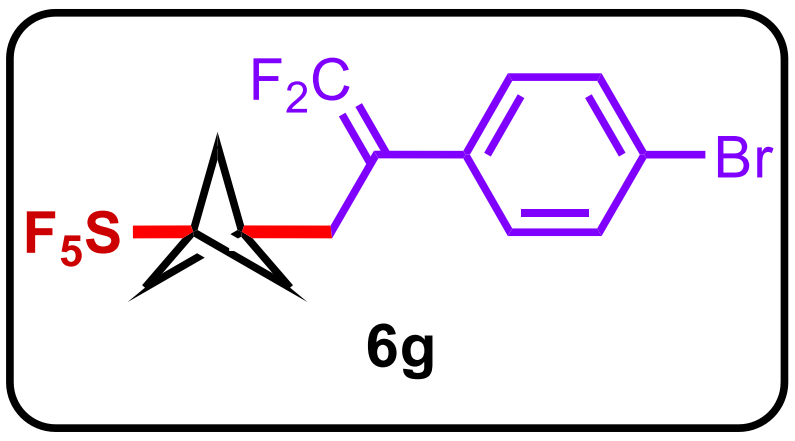

The product **6g** was purified with HPLC Thermo Scientific UltiMate 3000 (Shimadzu Shim-pack PRC-ODS) (MeCN) as a colorless oil (40% yield)

^{1}H NMR (400 MHz, DMSO-d_6) δ 7.62 – 7.55 (m, 2H), 7.40 – 7.32 (m, 2H), 2.74 (t, *J* = 2.4 Hz, 2H), 2.07 (s, 6H).

^{13}C NMR (101 MHz, DMSO-d_6) δ 153.61 (dd, *J* = 291.5, 287.3 Hz), 132.12 (t, *J* = 4.1 Hz), 131.58, 130.30 (t, *J* = 3.3 Hz), 120.92, 89.34 (dd, *J* = 22.4, 13.5 Hz), 66.82 (p, *J* = 12.6 Hz), 54.94, 33.84, 27.21.

^{19}F NMR (376 MHz, DMSO-d_6) δ 82.27 (m, 1F), 49.48 (d, *J* = 146.3 Hz, 4F), -89.72 (d, *J* = 39.6 Hz, 1F), -90.39 (d, *J* = 39.8 Hz, 1F).

HRMS (ESI) (*m/z*): $[M+Na]^+$ calcd. for $C_{14}H_{12}BrF_7SNa$: 446.9624, found: 446.9625.

(3-(2-(4-chlorophenyl)-3,3-difluoroallyl)bicyclo[1.1.1]pentan-1-yl)pentafluoro-λ⁶-sulfane

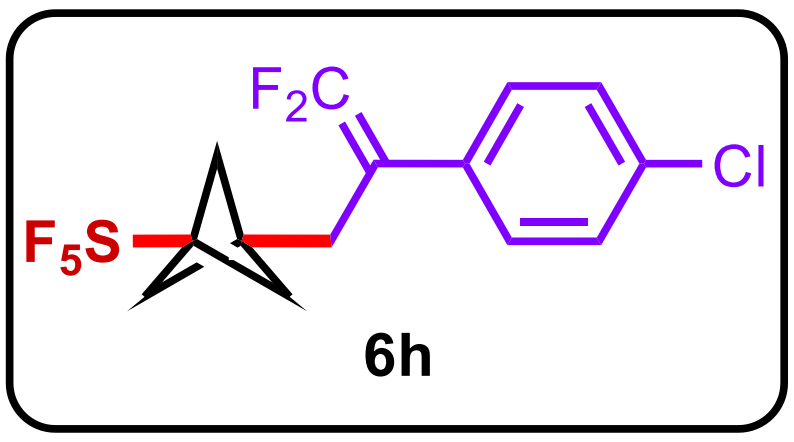

The product **6h** was purified with HPLC Thermo Scientific UltiMate 3000 (Shimadzu Shim-pack PRC-ODS) (MeCN) as a colorless oil (33% yield)

^{1}H NMR (400 MHz, DMSO-d_6) δ 7.48 – 7.37 (m, 4H), 2.75 (t, J = 2.4 Hz, 2H), 2.08 (s, 6H).

^{13}C NMR (101 MHz, DMSO-d_6) δ 153.67 (dd, J = 291.3, 287.1 Hz), 132.38, 131.72 (t, J = 4.0 Hz), 130.01 (t, J = 3.5 Hz), 128.65, 89.29 (dd, J = 22.3, 13.5 Hz), 66.83 (p, J = 12.6 Hz), 54.95, 33.84, 27.26.

^{19}F NMR (376 MHz, DMSO-d_6) δ 82.29 (m, 1F), 49.47 (d, J = 146.0 Hz, 4F), -89.87 (d, J = 40.2 Hz, 1F), -90.51 (d, J = 39.8 Hz, 1F).

HRMS (ESI) (*m/z*): $[M+Na]^+$ calcd. for $C_{14}H_{12}ClF_7S$: 403.0129, found:403.0127.

(3-(2-(3-chloro-4-methylphenyl)-3,3-difluoroallyl)bicyclo[1.1.1]pentan-1-yl)pentafluoro-λ^6-sulfane

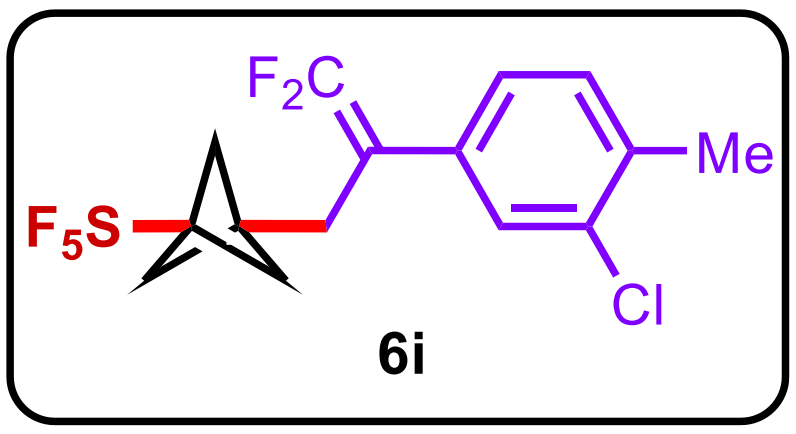

The product **6i** was purified with HPLC Thermo Scientific UltiMate 3000 (Shimadzu Shim-pack PRC-ODS) (MeCN) as a colorless oil (40% yield)

^{1}H NMR (400 MHz, DMSO-d_6) δ 7.46 (s, 1H), 7.35 (d, J = 8.0 Hz, 1H), 7.30 – 7.23 (m, 1H), 2.74 (t, J = 2.4 Hz, 2H), 2.31 (s, 3H), 2.08 (s, 6H).

^{13}C NMR (101 MHz, DMSO-d_6) δ 153.75 (dd, J = 291.2, 286.9 Hz), 135.00, 133.48, 132.82 – 131.76 (m), 131.33, 128.23 (t, J = 3.6 Hz), 126.86 (t, J = 3.3 Hz), 89.08 (dd, J = 22.5, 13.4 Hz), 66.84 (p, J = 12.6 Hz), 54.93, 33.84, 27.18, 19.25.

^{19}F NMR (376 MHz, DMSO-d_6) δ 82.25 (p, J = 145.9, 145.3 Hz, 1F), 49.45 (d, J = 145.9 Hz, 4F), -89.97 (d, J = 39.8 Hz, 1F), -90.42 (d, J = 40.6 Hz, 1F).

HRMS (ESI) (*m/z*): $[M+Na]^+$ calcd. for $C_{15}H_{14}ClF_7SNa$: 417.0285, found: 417.0298.

(3-(2-(4-(tert-butyl)phenyl)-3,3-difluoroallyl)bicyclo[1.1.1]pentan-1-yl)pentafluoro-λ⁶-sulfane

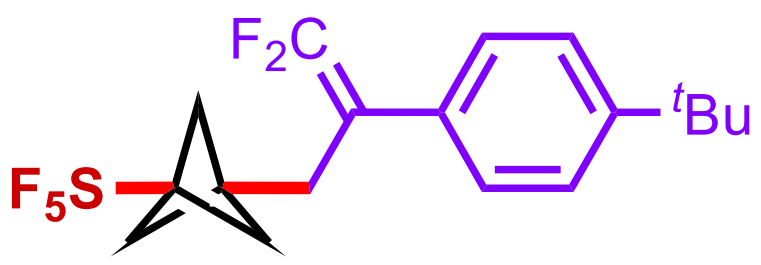

6j

The product **6j** was purified with HPLC Thermo Scientific UltiMate 3000 (Shimadzu Shim-pack PRC-ODS) (MeCN) as a colorless oil (47% yield)

^{1}H NMR (500 MHz, DMSO-d_6) δ 7.40 – 7.35 (m, 2H), 7.29 – 7.25 (m, 2H), 2.71 (t, J = 2.4 Hz, 2H), 2.05 (s, 6H), 1.23 (s, 9H).

^{13}C NMR (126 MHz, DMSO-d_6) δ 153.70 (dd, J = 290.5, 286.7 Hz), 150.21, 129.80 (t, J = 4.1 Hz), 127.75 (t, J = 3.2 Hz), 125.37, 89.74 (dd, J = 21.2, 13.7 Hz), 66.86 (p, J = 12.6 Hz), 54.98, 34.33, 33.97 (t, J = 3.2 Hz), 31.05, 27.42.

^{19}F NMR (471 MHz, DMSO-d_6) δ 83.09 – 81.20 (m, 1F), 49.42 (d, J = 145.8 Hz, 4F), -90.73 (d, J = 43.2 Hz, 1F), -91.57 (d, J = 42.2 Hz, 1F).

HRMS (ESI) (m/z): [M+Na]$^+$ calcd. for $C_{18}H_{21}F_7SNa$: 425.1144, found:425.1140.

(4-(1,1-difluoro-3-(3-(pentafluoro-λ⁶-sulfaneyl)bicyclo[1.1.1]pentan-1-yl)prop-1-en-2-yl)phenyl)trimethylsilane

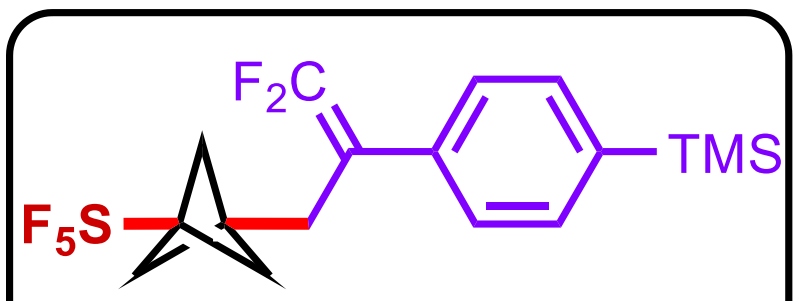

6k

The product **6k** was purified with HPLC Thermo Scientific UltiMate 3000 (Shimadzu Shim-pack PRC-ODS) (MeCN) as a colorless oil (48% yield)

^{1}H NMR (500 MHz, DMSO-d_6) δ 7.51 – 7.47 (m, 2H), 7.34 (dd, J = 8.1, 1.5 Hz, 2H), 2.72 (t, J = 2.4 Hz, 2H), 2.05 (s, 6H), 0.20 (s, 9H).

^{13}C NMR (126 MHz, DMSO-d_6) δ 153.78 (dd, J = 291.3, 287.0 Hz), 139.40, 133.43, 133.31 (t, J = 4.3 Hz), 127.36 (t, J = 3.2 Hz), 89.99 (dd, J = 21.2, 13.0 Hz), 66.83 (p, J = 12.6 Hz),

54.97, 33.95 (t, J = 3.2 Hz), 27.35, -1.21.

^{19}F NMR (471 MHz, DMSO-d_6) δ 83.28 – 81.18 (m, 1F), 49.41 (d, J = 145.7 Hz, 4F), -90.05 (d, J = 41.1 Hz, 1F), -90.97 (d, J = 41.3 Hz, 1F).

HRMS (ESI) (m/z): $[M+H]^+$ calcd. for $C_{17}H_{21}F_7SSiNa$: 441.0914, found: 441.0953.

(3-(3,3-difluoro-2-(4-methoxyphenyl)allyl)bicyclo[1.1.1]pentan-1-yl)pentafluoro-λ^6-sulfane

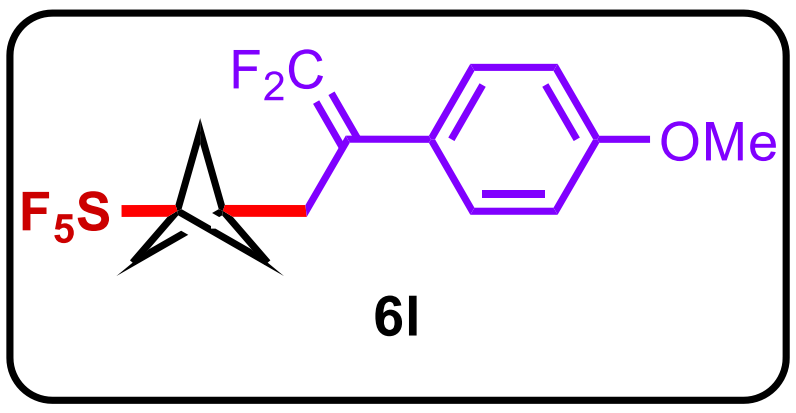

The product **6l** was purified with HPLC Thermo Scientific UltiMate 3000 (Shimadzu Shim-pack PRC-ODS) (MeCN) as a colorless oil (41% yield)

^{1}H NMR (400 MHz, DMSO-d_6) δ 7.34 – 7.26 (m, 2H), 6.97 – 6.90 (m, 2H), 3.74 (s, 3H), 2.70 (t, J = 2.4 Hz, 2H), 2.05 (s, 6H).

^{13}C NMR (101 MHz, DMSO-d_6) δ 158.69, 153.44 (dd, J = 289.1, 285.4 Hz), 129.40 (t, J = 3.3 Hz), 124.63 (t, J = 3.9 Hz), 114.08, 89.59 (dd, J = 21.5, 14.2 Hz), 66.89 (p, J = 12.6 Hz), 55.07 (d, J = 16.4 Hz), 33.91, 27.44.

^{19}F NMR (376 MHz, DMSO-d_6) δ 82.40 (p, J = 146.3, 145.5 Hz, 1F), 49.46 (d, J = 146.4 Hz, 4F), -91.95 (d, J = 43.4 Hz, 1F), -92.55 (d, J = 44.9 Hz, 1F).

HRMS (ESI) (m/z): $[M+H]^+$ calcd. for $C_{15}H_{16}F_7OS$: 377.0805, found: 377.0781.

(3-(3,3-difluoro-2-(4-phenoxyphenyl)allyl)bicyclo[1.1.1]pentan-1-yl)pentafluoro-λ^6-sulfane

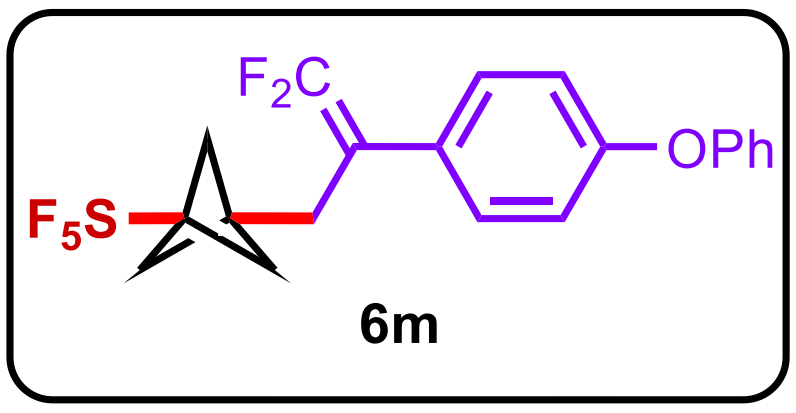

The product **6m** was purified with HPLC Thermo Scientific UltiMate 3000 (Shimadzu Shim-pack PRC-ODS) (MeCN) as a colorless oil (36% yield)

^{1}H NMR (500 MHz, DMSO-d_6) δ 7.42 – 7.35 (m, 4H), 7.18 – 7.12 (m, 1H), 7.04 – 6.98 (m, 4H), 2.74 (t, *J* = 2.3 Hz, 2H), 2.09 (s, 6H).

^{13}C NMR (126 MHz, DMSO-d_6) δ 156.27, 156.25, 153.59 (dd, *J* = 290.1, 286.3 Hz), 130.15, 129.87 (t, *J* = 3.6 Hz), 127.60 (t, *J* = 4.1 Hz), 123.84, 118.97, 118.42, 89.45 (dd, *J* = 21.5, 13.8 Hz), 66.86 (p, *J* = 12.6 Hz), 54.98, 33.91, 27.45.

^{19}F NMR (471 MHz, DMSO-d_6) δ 83.22 – 81.28 (m, 1F), 49.49 (d, *J* = 145.9 Hz, 4F), -90.98 (d, *J* = 42.5 Hz, 1F), -91.61 (d, *J* = 43.1 Hz, 1F).

HRMS (ESI) (*m/z*): [M+Na]$^+$ calcd. for $C_{20}H_{17}F_7OS$:461.0781, found: 461.0796.

3-(1,1-difluoro-3-(3-(pentafluoro-λ^6-sulfaneyl)bicyclo[1.1.1]pentan-1-yl)prop-1-en-2-yl)dibenzo[b,d]thiophene

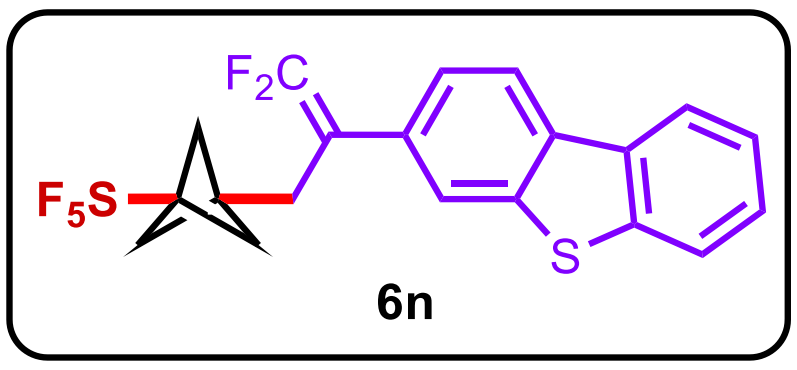

The product **6n** was purified with HPLC Thermo Scientific UltiMate 3000 (Shimadzu Shim-pack PRC-ODS) (MeCN) as a colorless oil (39% yield)

^{1}H NMR (400 MHz, DMSO-d_6) δ 8.41 – 8.29 (m, 2H), 8.08 – 7.94 (m, 1H), 7.59 – 7.43 (m, 4H), 2.85 (t, *J* = 2.2 Hz, 2H), 2.00 (s, 6H).

^{13}C NMR (101 MHz, DMSO-d_6) δ 154.45 (dd, *J* = 293.9, 289.9 Hz,), 138.32, 138.01, 135.72, 135.27, 127.98, 127.64, 127.59, 125.40, 125.14, 123.16, 122.50, 122.17, 89.26 (dd, *J* = 22.7, 16.2 Hz), 66.76 (p, *J* = 12.6 Hz), 54.98, 33.90, 27.96.

^{19}F NMR (376 MHz, DMSO-d_6) δ 84.41 – 80.15 (m, 1F), 49.43 (d, *J* = 146.1 Hz, 4F), -85.35 (d, *J* = 36.5 Hz, 1F), -90.20 (d, *J* = 35.6 Hz, 1F).

HRMS (ESI) (*m/z*): [M+Na]$^+$ calcd. for $C_{20}H_{15}F_7S_2$: 475.0396, found: 475.0389.

Supplementary Figures

NMR Spectra of Compounds

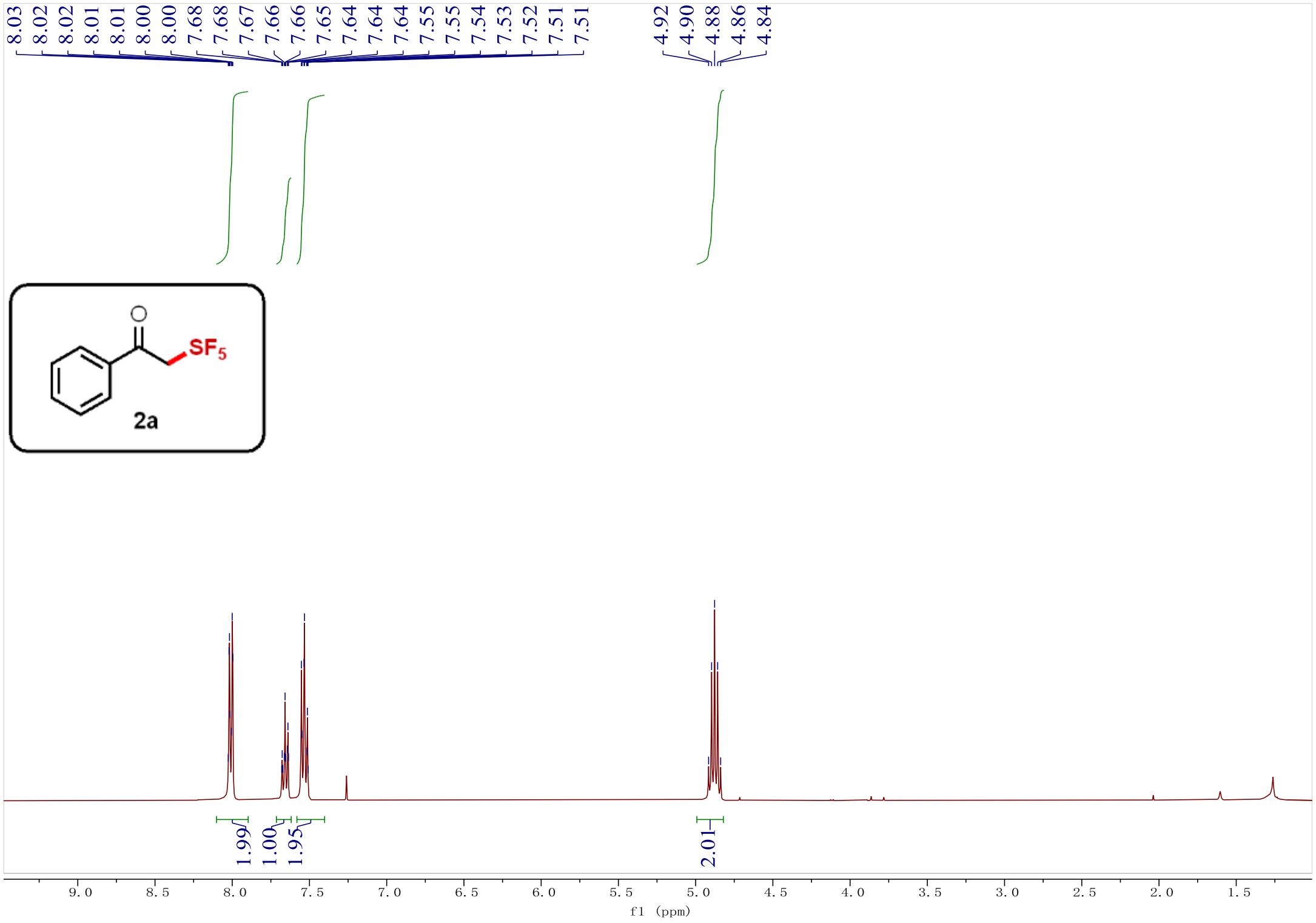

Supplementary Figure 1. ^{1}H NMR Spectrum of Compound 2a (400 MHz, $CDCl_3$, 25 °C)

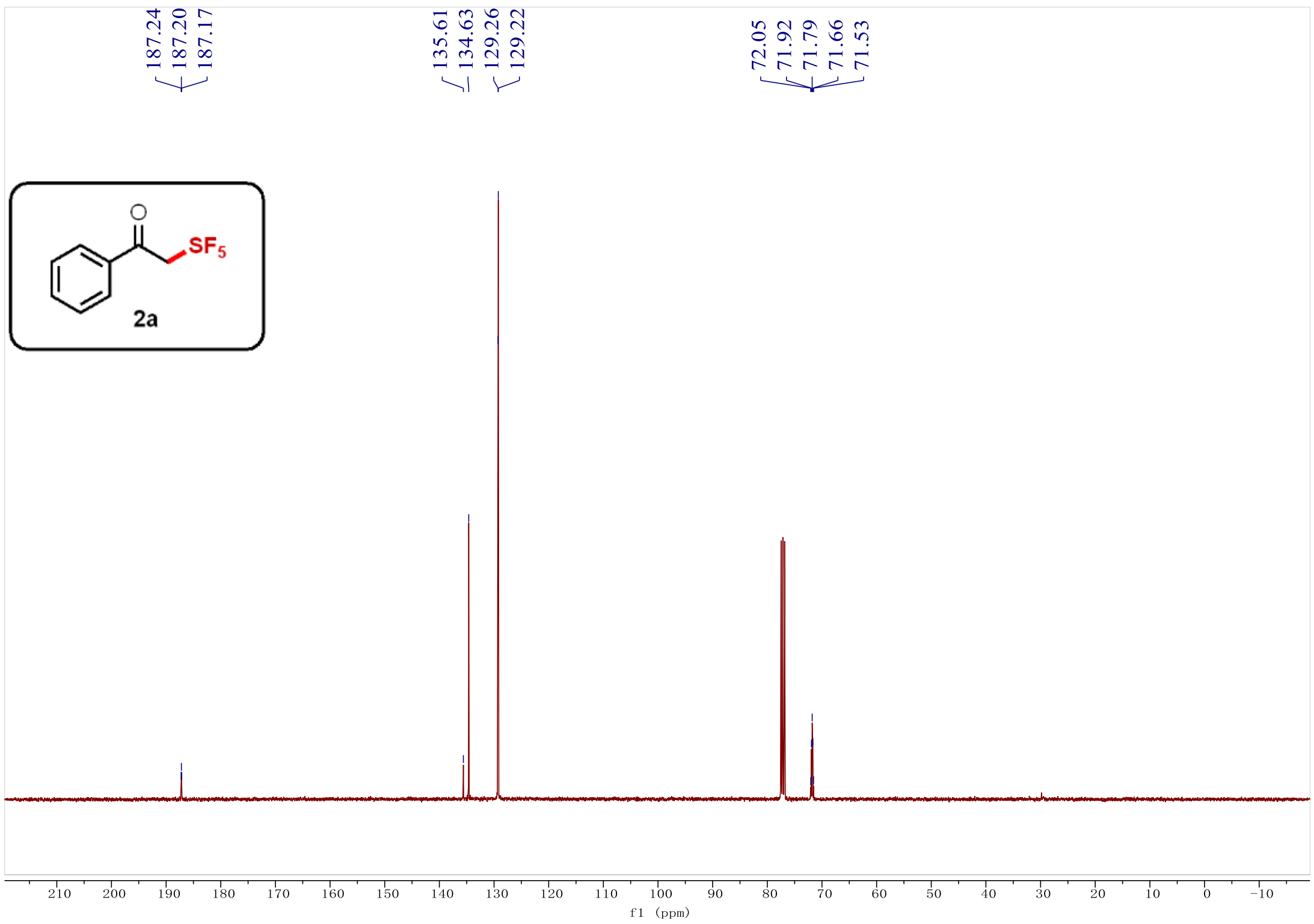

Supplementary Figure 2. ^{13}C NMR Spectrum of Compound 2a (101 MHz, $CDCl_3$, 25 °C)

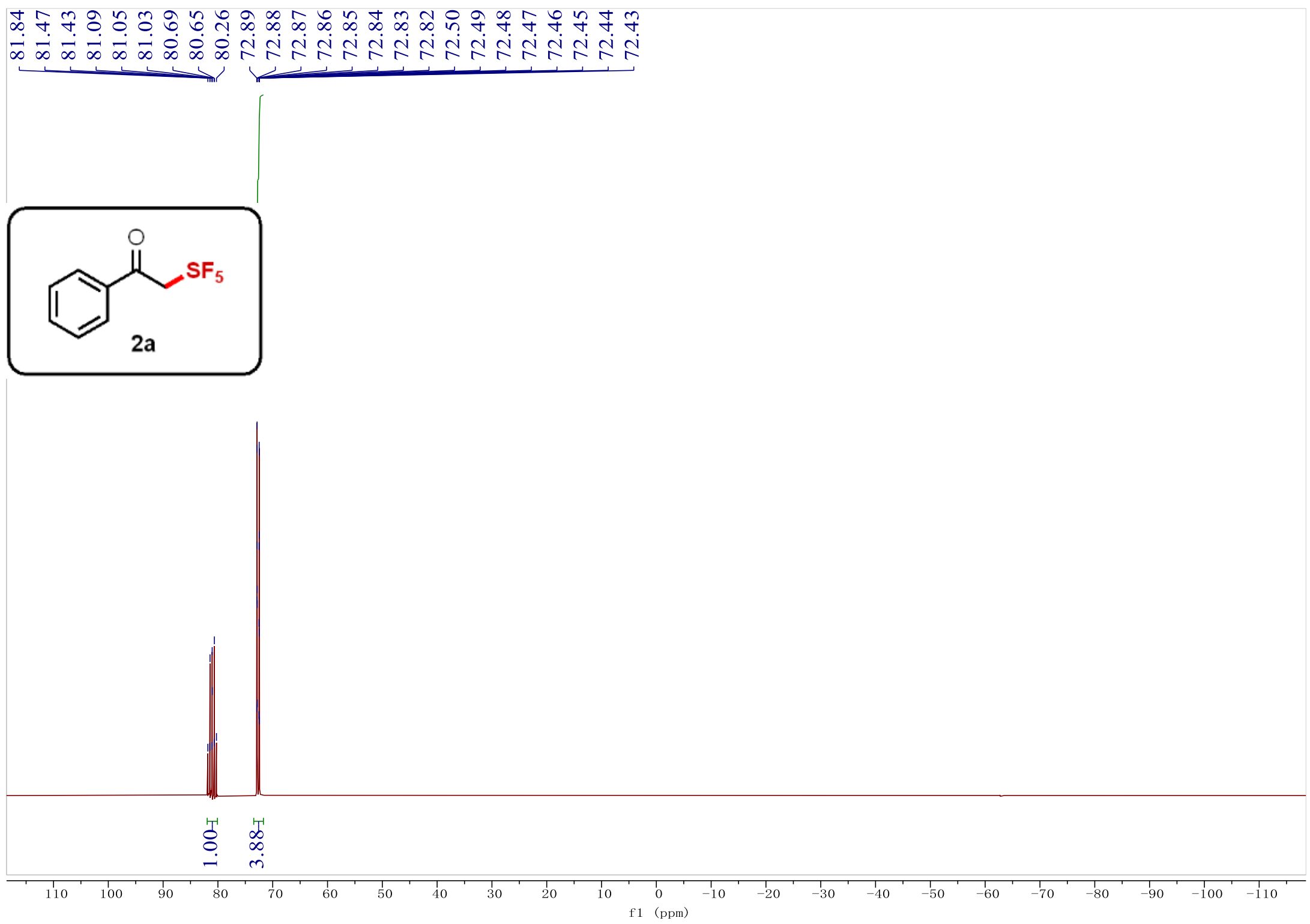

Supplementary Figure 3. ^{19}F NMR Spectrum of Compound 2a (376 MHz, $CDCl_3$, 25 °C)

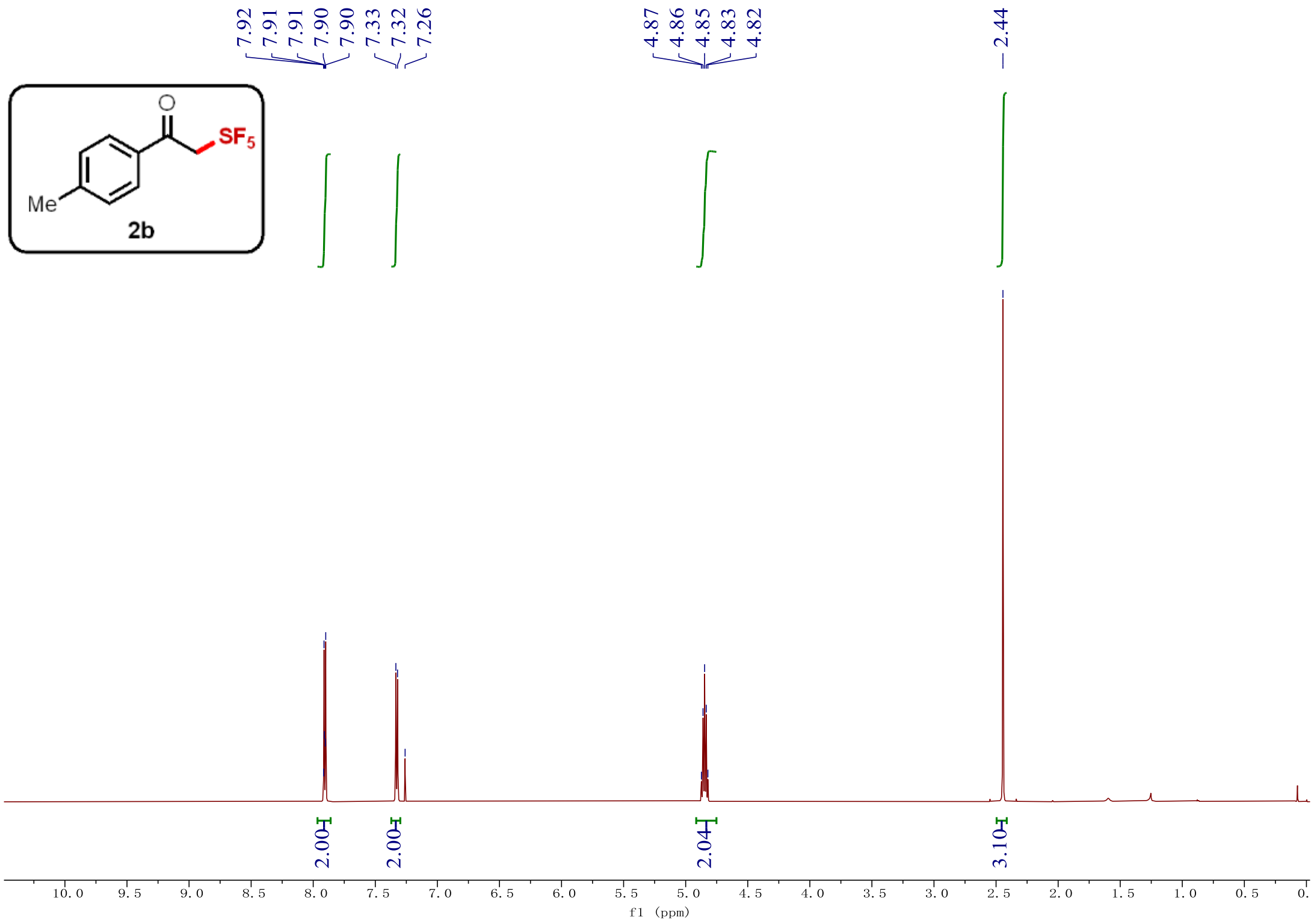

Supplementary Figure 4. ^{1}H NMR Spectrum of Compound 2b (600 MHz, $CDCl_3$, 25 °C)

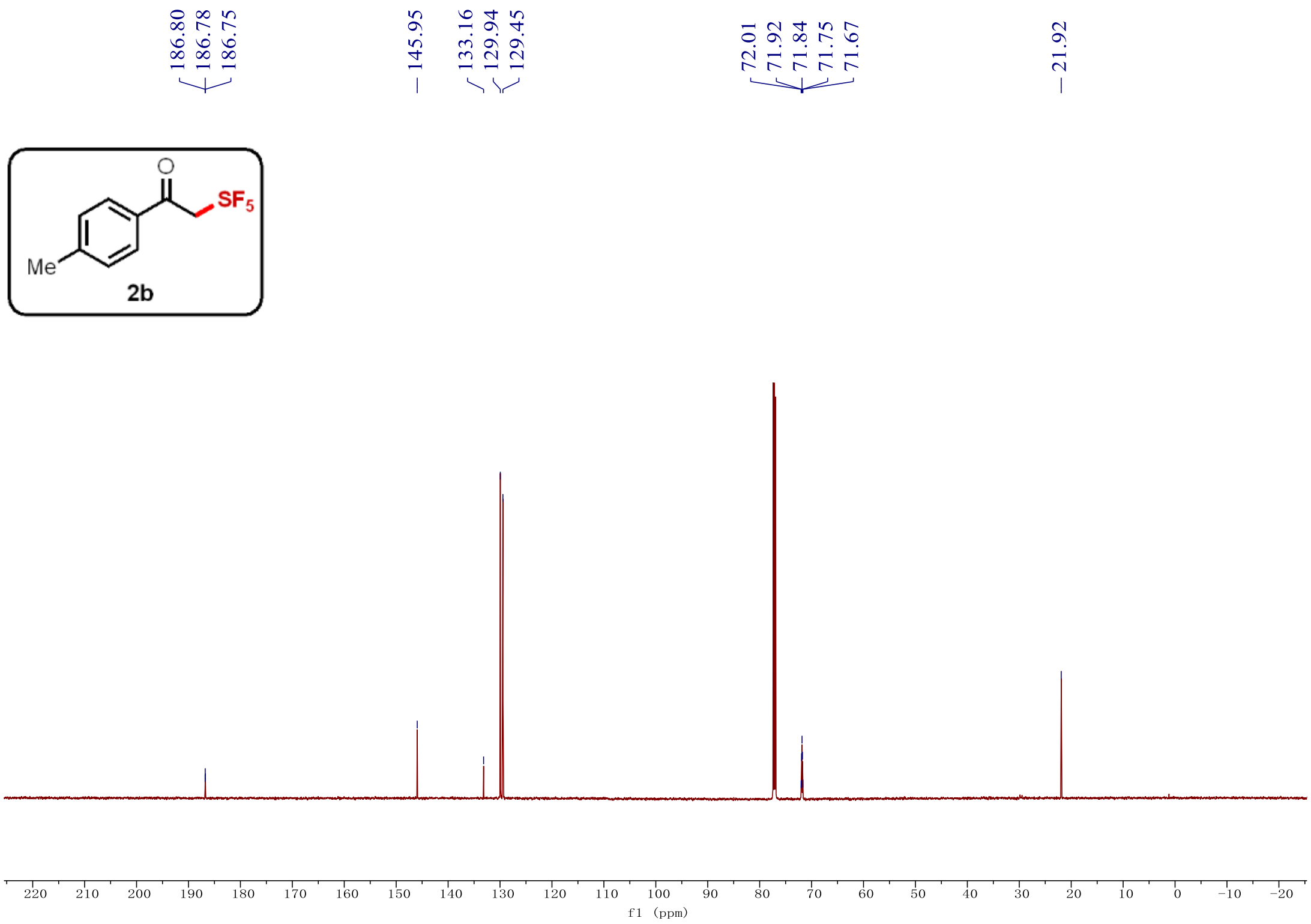

Supplementary Figure 5. ^{13}C NMR Spectrum of Compound 2b (151 MHz, $CDCl_3$, 25 °C)

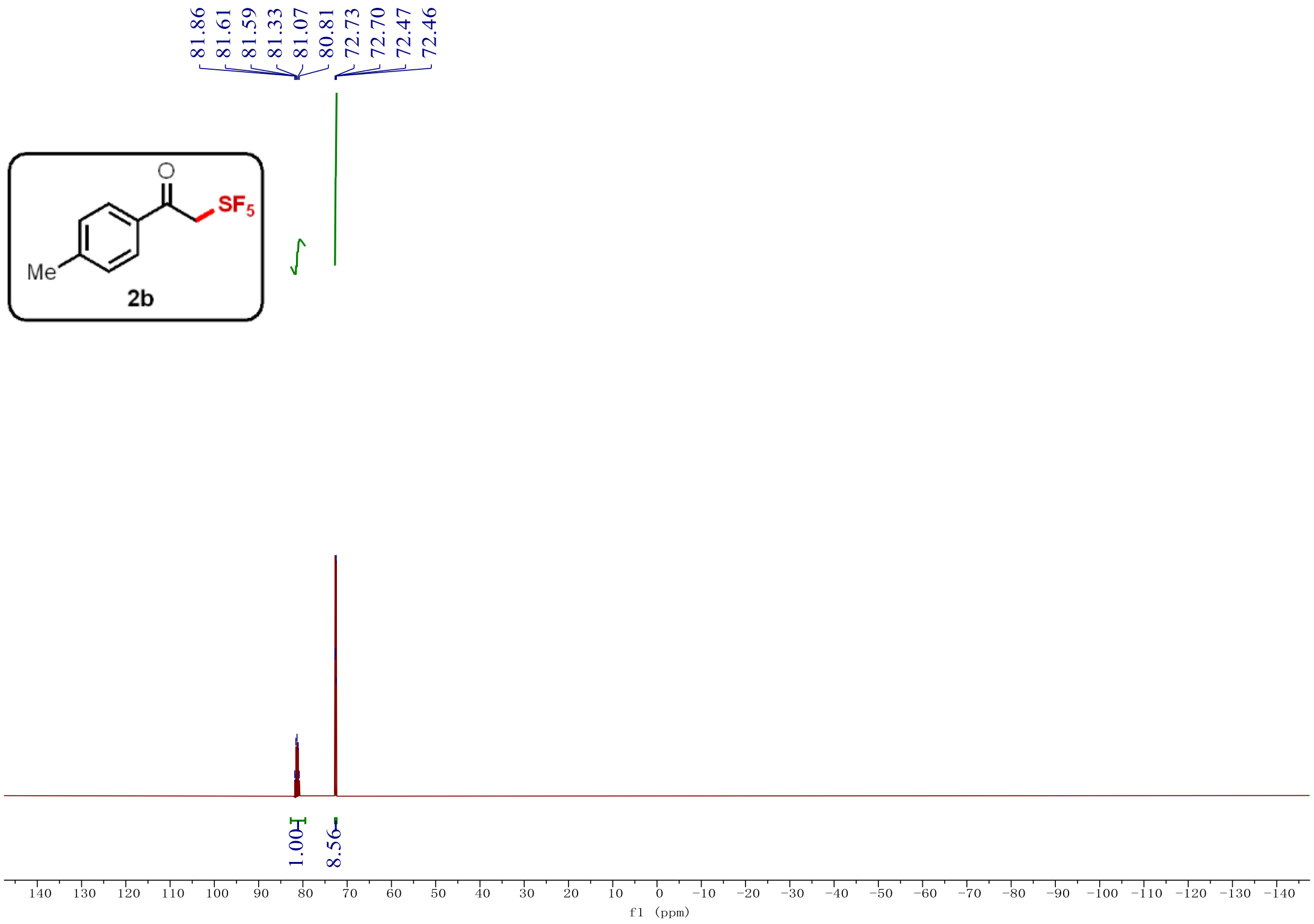

Supplementary Figure 6. ^{19}F NMR Spectrum of Compound 2b (565 MHz, $CDCl_3$, 25 °C)

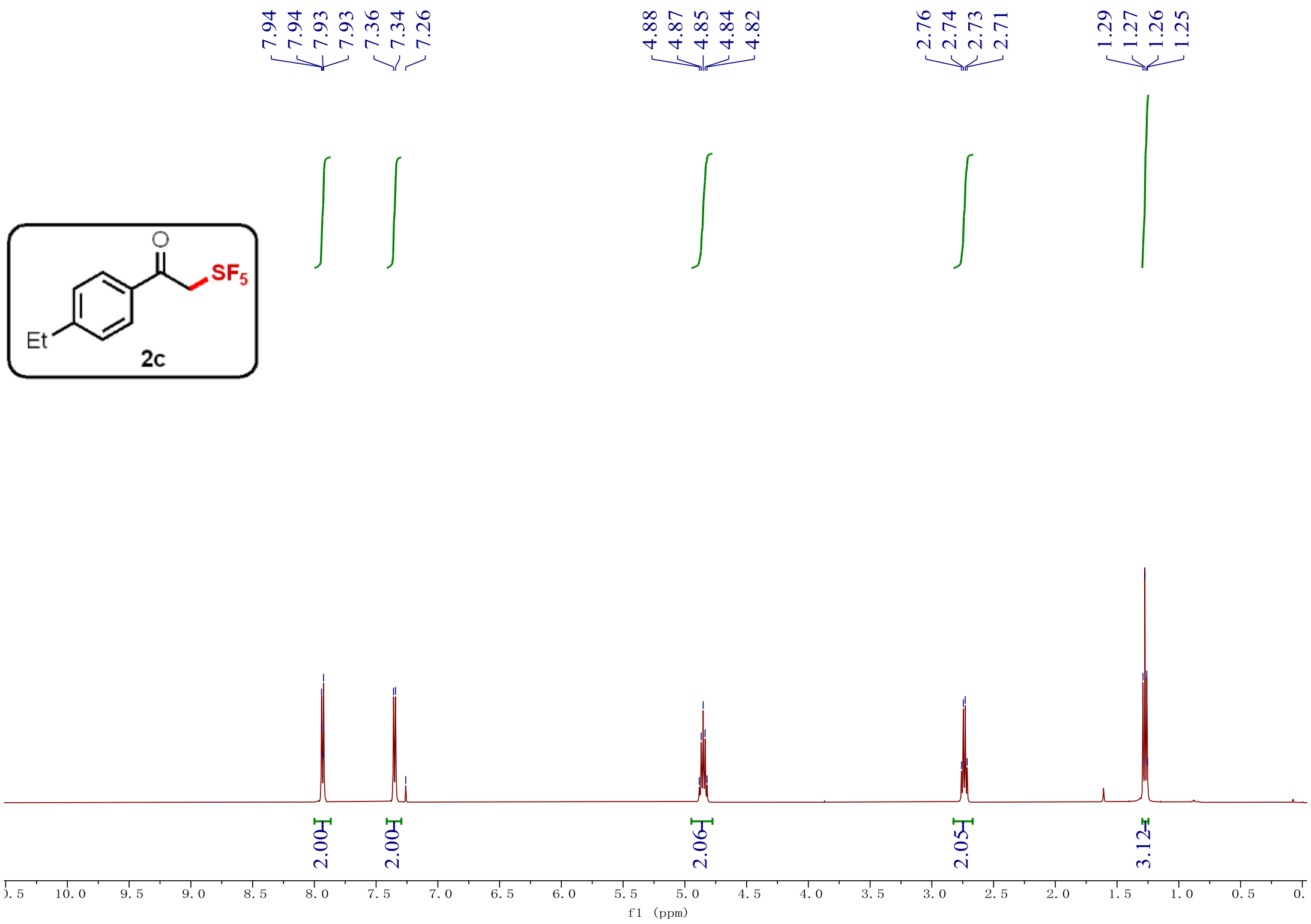

Supplementary Figure 7. ^{1}**H NMR Spectrum of Compound 2c (500 MHz, CDCl$_3$, 25 °C)**

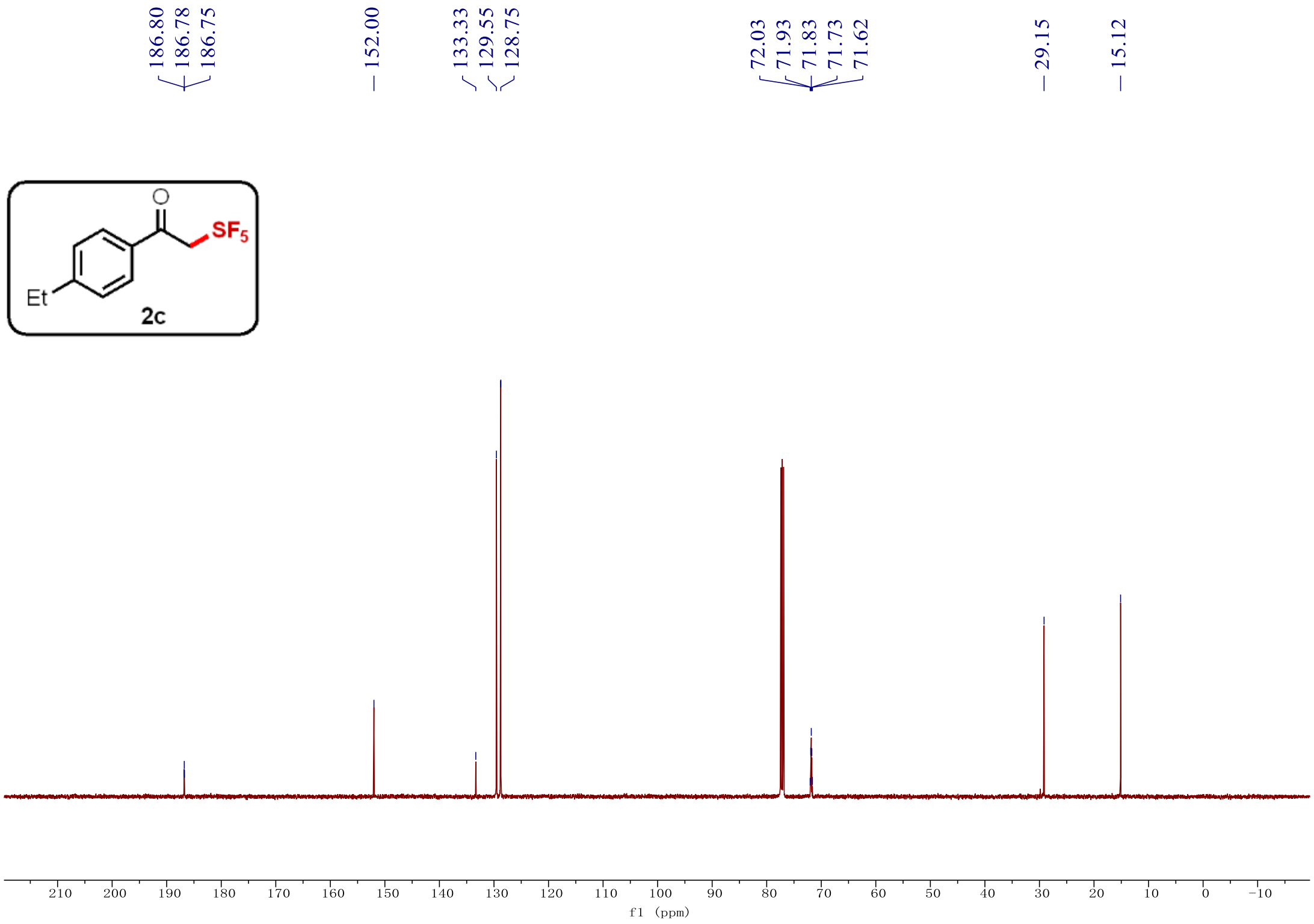

Supplementary Figure 8. ^{13}**C NMR Spectrum of Compound 2c (126 MHz, CDCl$_3$, 25 °C)**

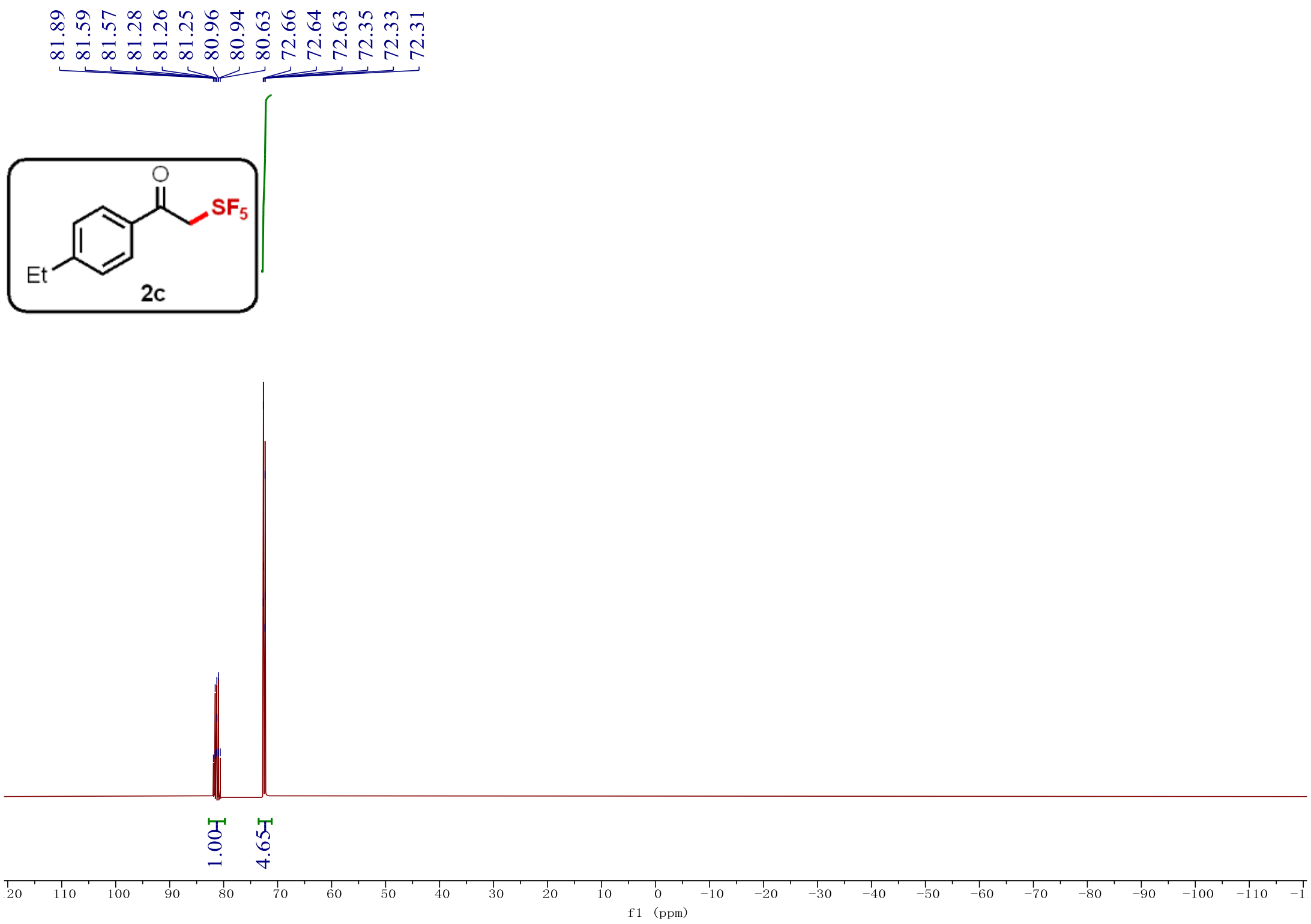

Supplementary Figure 9. ^{19}F NMR Spectrum of Compound 2c (471 MHz, $CDCl_3$, 25 °C)

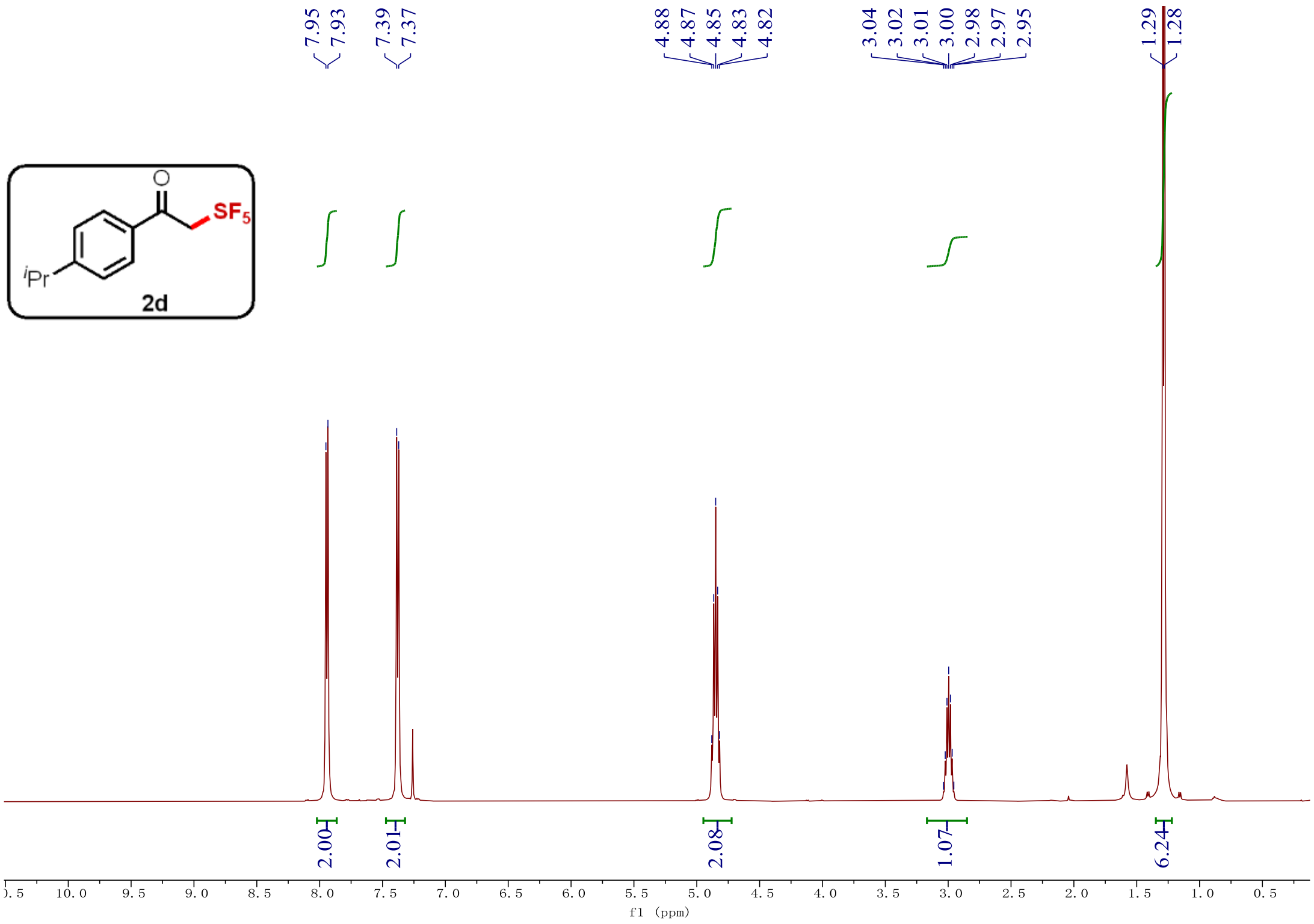

Supplementary Figure 10. ^{1}H NMR Spectrum of Compound 2d (500 MHz, $CDCl_3$, 25 °C)

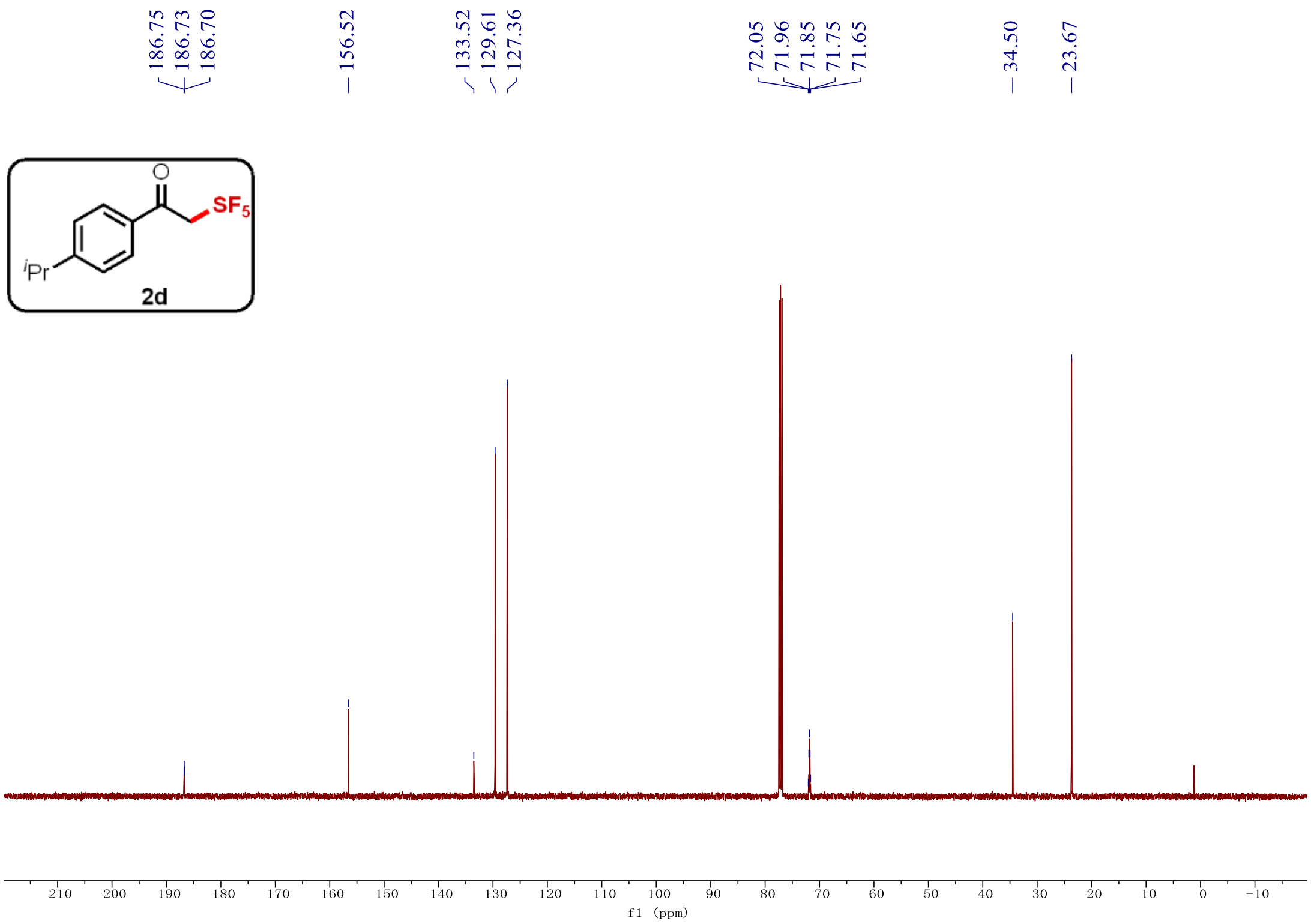

Supplementary Figure 11. ^{13}C NMR Spectrum of Compound 2d (126 MHz, $CDCl_3$, 25 ˚C)

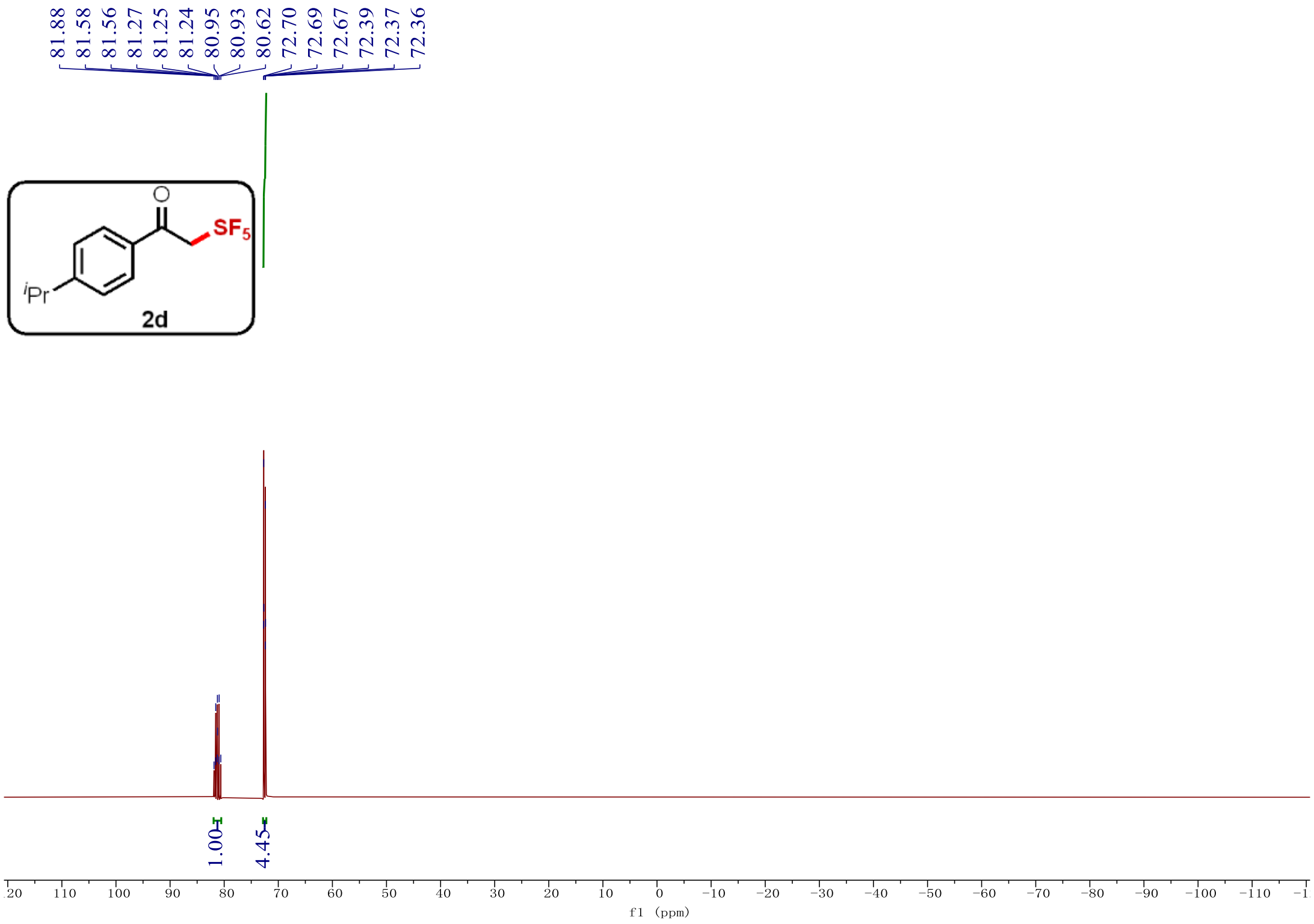

Supplementary Figure 12. ^{19}F NMR Spectrum of Compound 2d (471 MHz, $CDCl_3$, 25 ˚C)

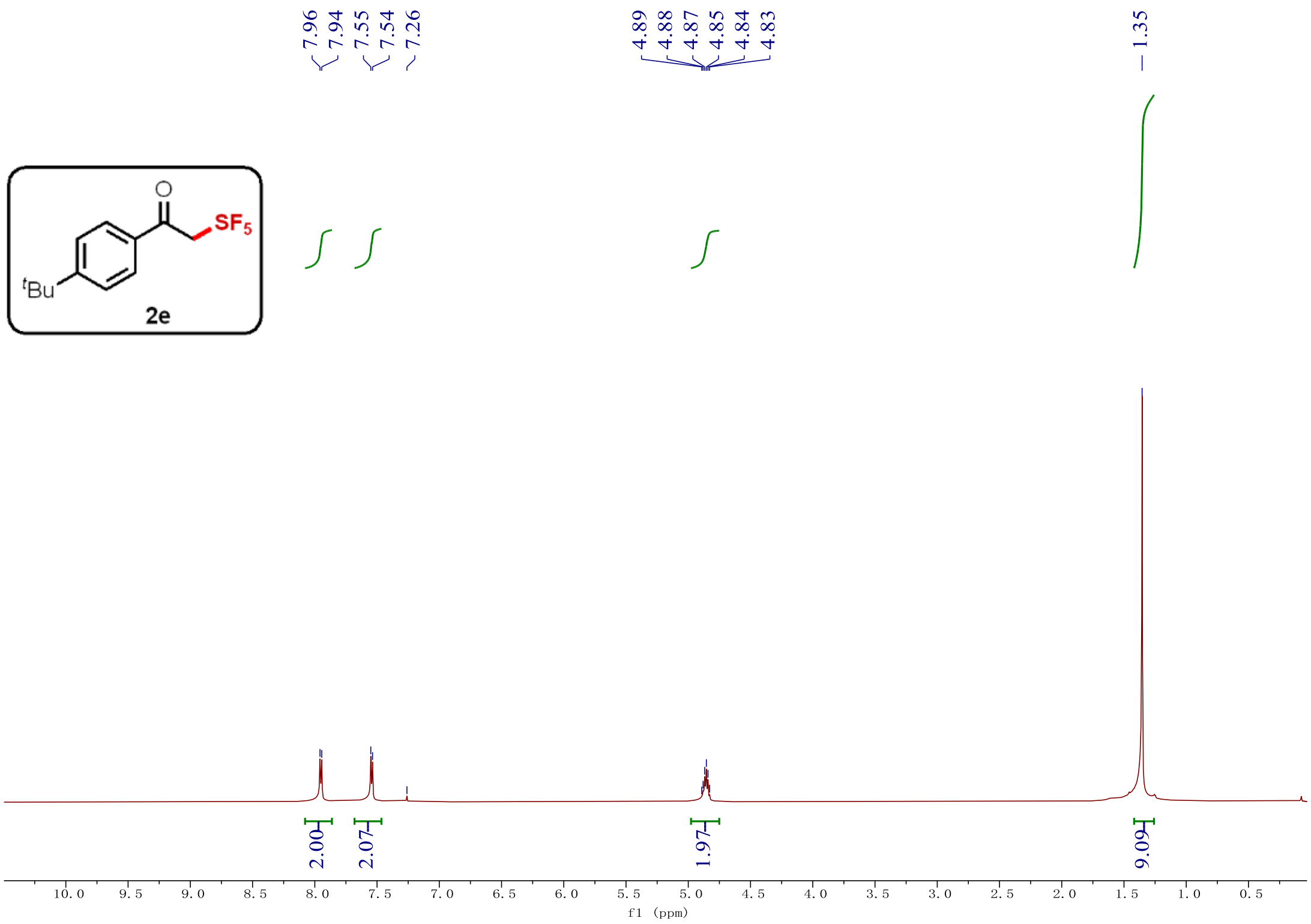

Supplementary Figure 13. ^{1}H NMR Spectrum of Compound 2e (600 MHz, $CDCl_3$, 25 ˚C)

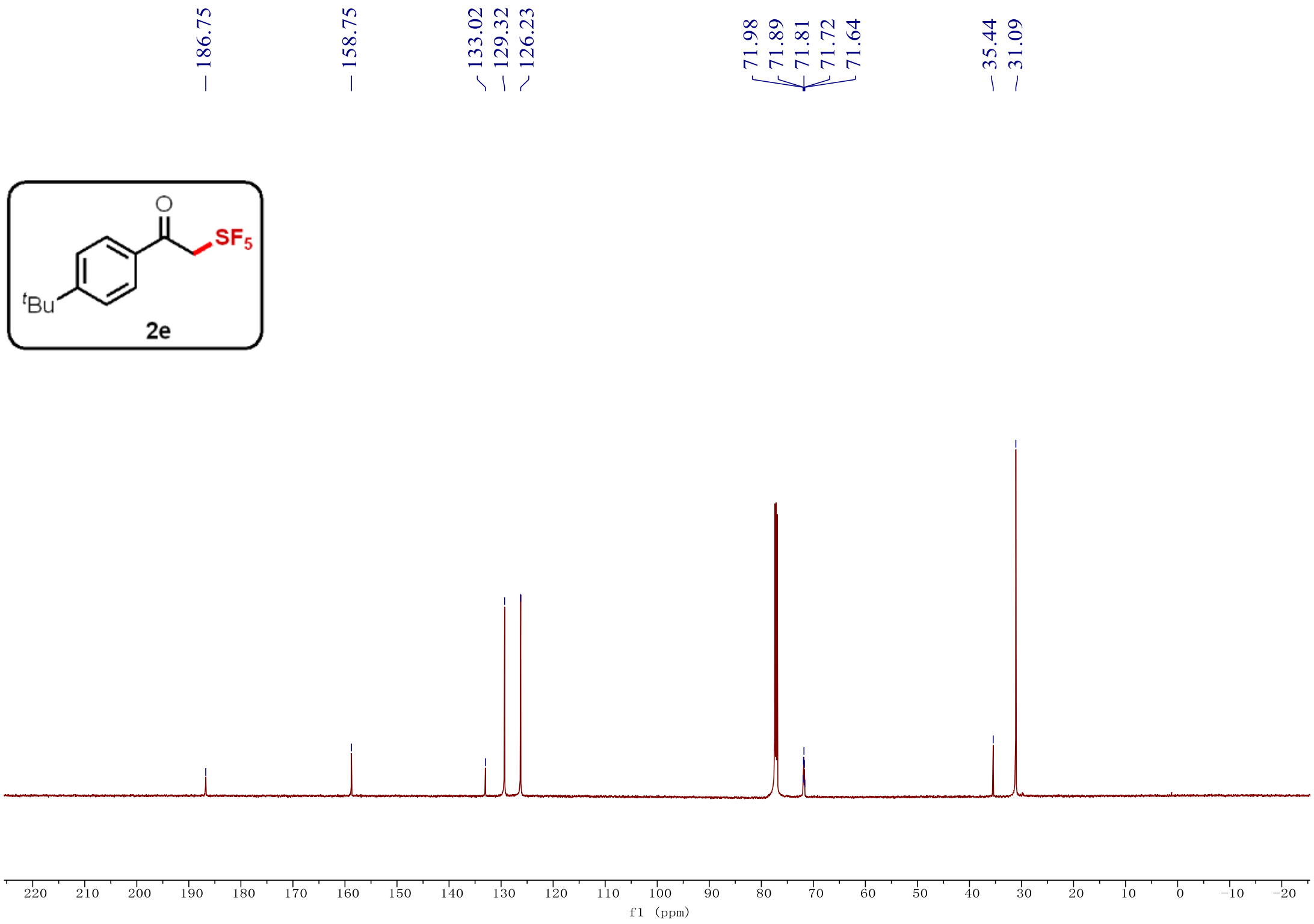

Supplementary Figure 14. ^{13}C NMR Spectrum of Compound 2e (151 MHz, $CDCl_3$, 25 ˚C)

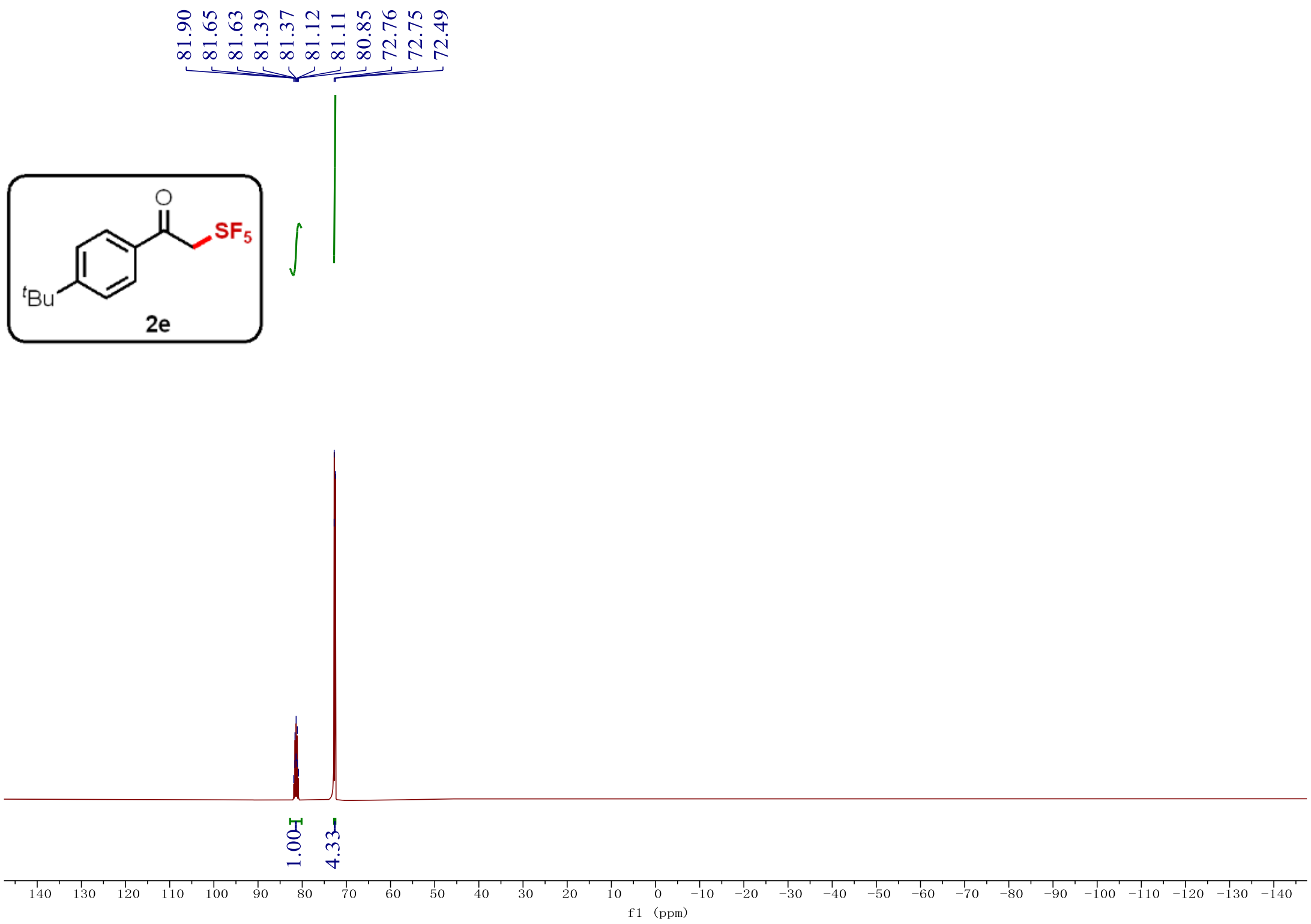

Supplementary Figure15. ^{19}F NMR Spectrum of Compound 2e (565 MHz, $CDCl_3$, 25 °C)

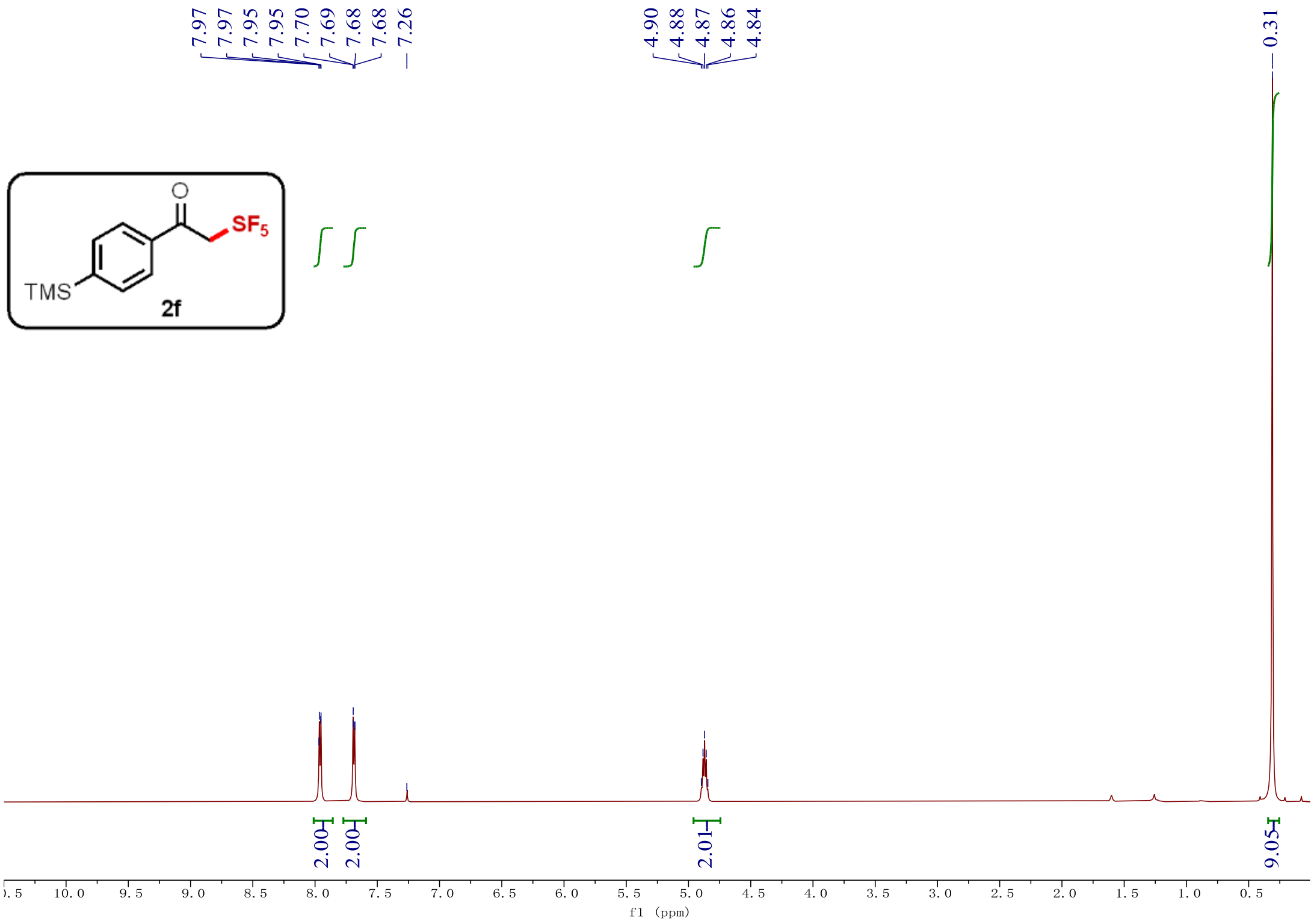

Supplementary Figure 16. ^{1}H NMR Spectrum of Compound 2f (600 MHz, $CDCl_3$, 25 °C)

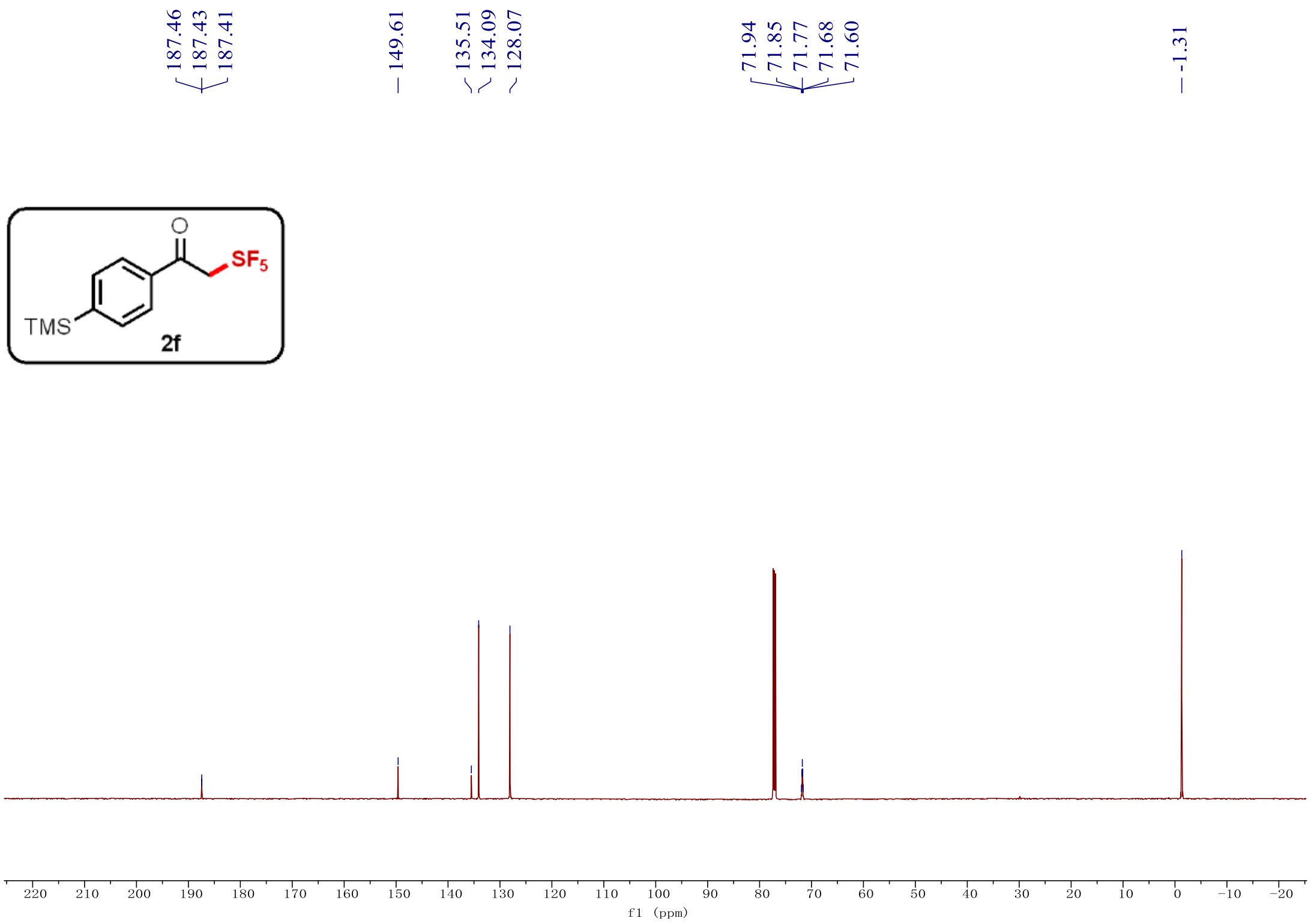

Supplementary Figure 17. ^{13}C NMR Spectrum of Compound 2f (151 MHz, $CDCl_3$, 25 °C)

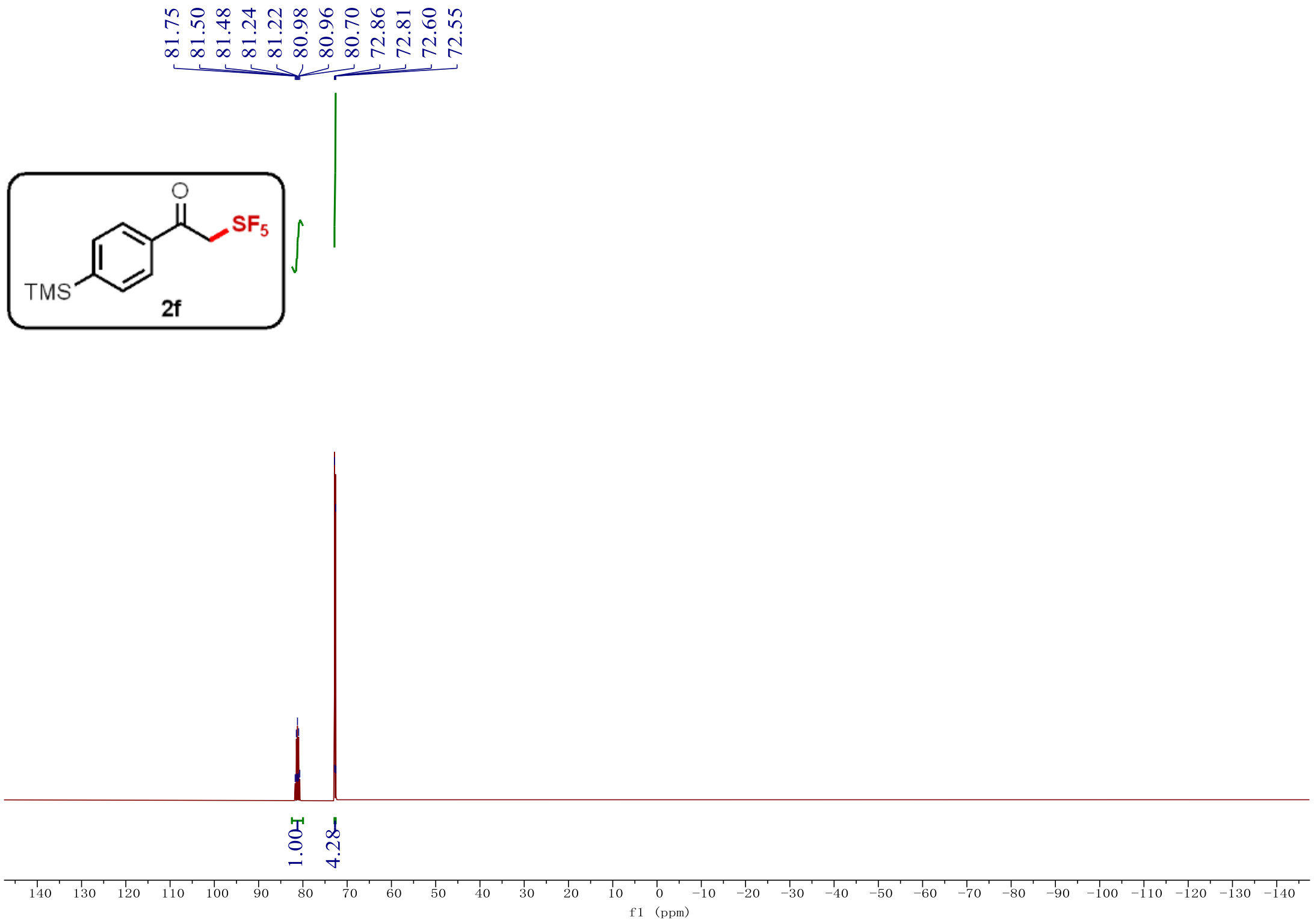

Supplementary Figure 18. ^{19}F NMR Spectrum of Compound 2f (565 MHz, $CDCl_3$, 25 °C)

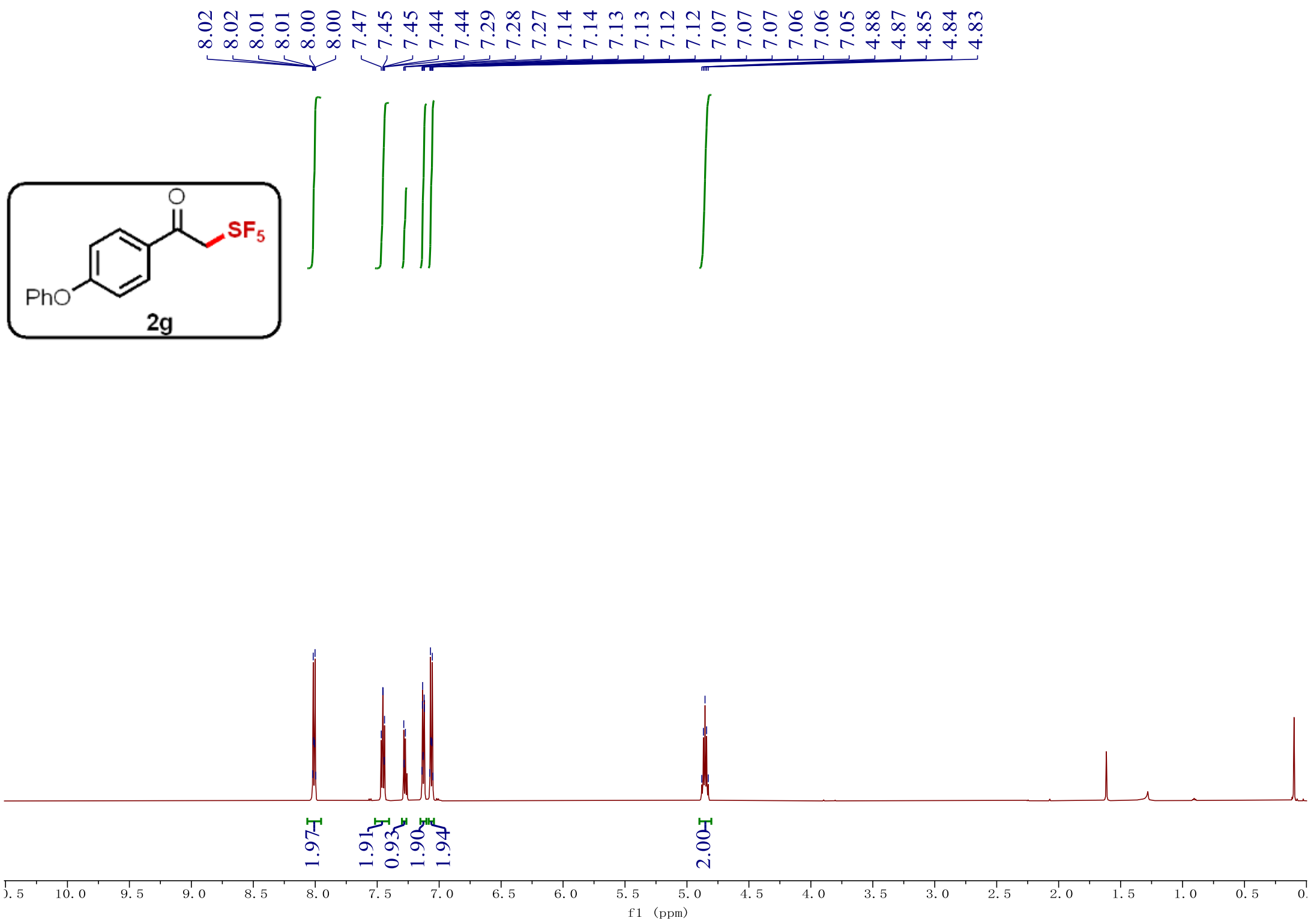

Supplementary Figure 19. ^{1}H NMR Spectrum of Compound 2g (600 MHz, $CDCl_3$, 25 ˚C)

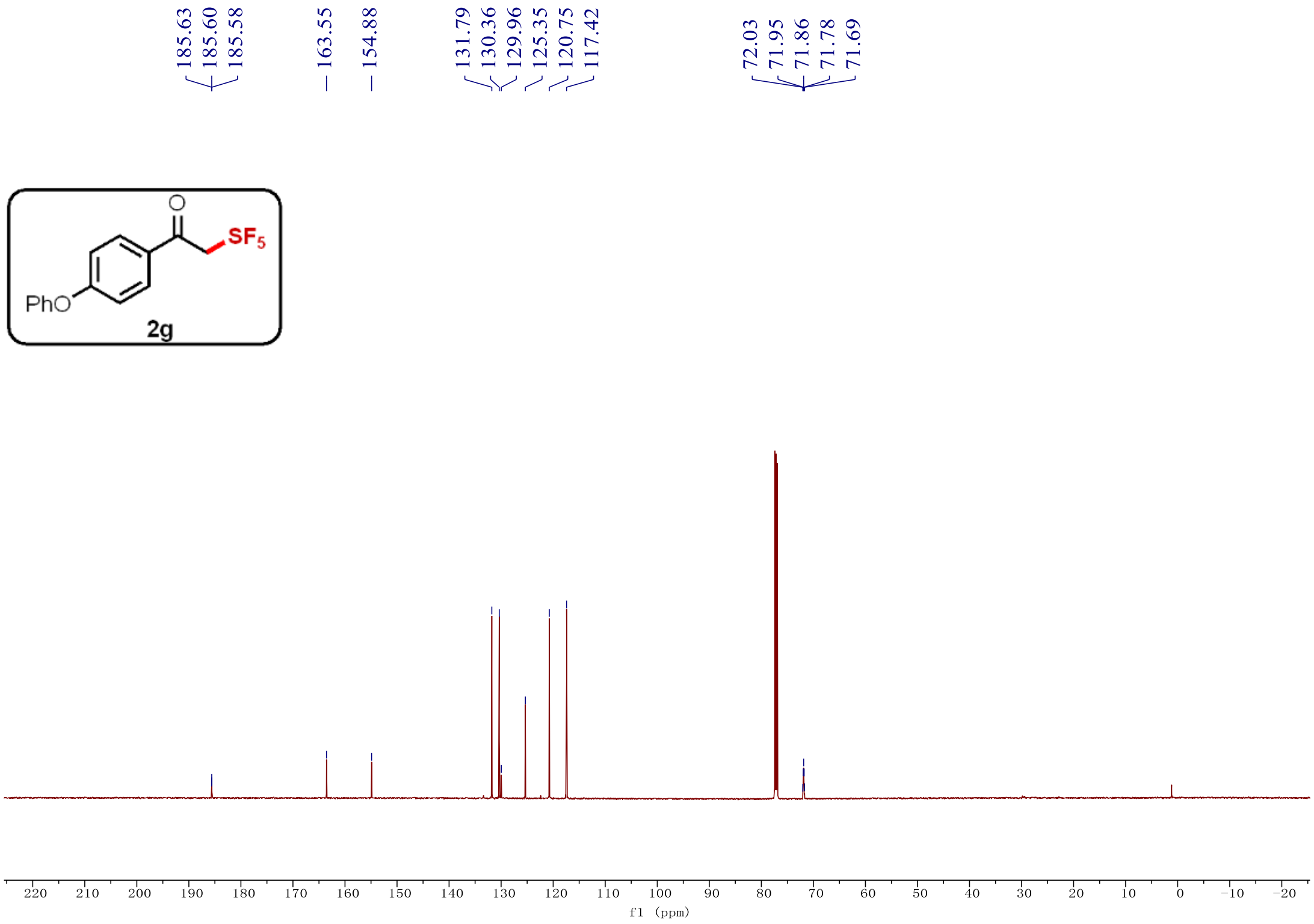

Supplementary Figure 20. ^{13}C NMR Spectrum of Compound 2g (151 MHz, $CDCl_3$, 25 ˚C)

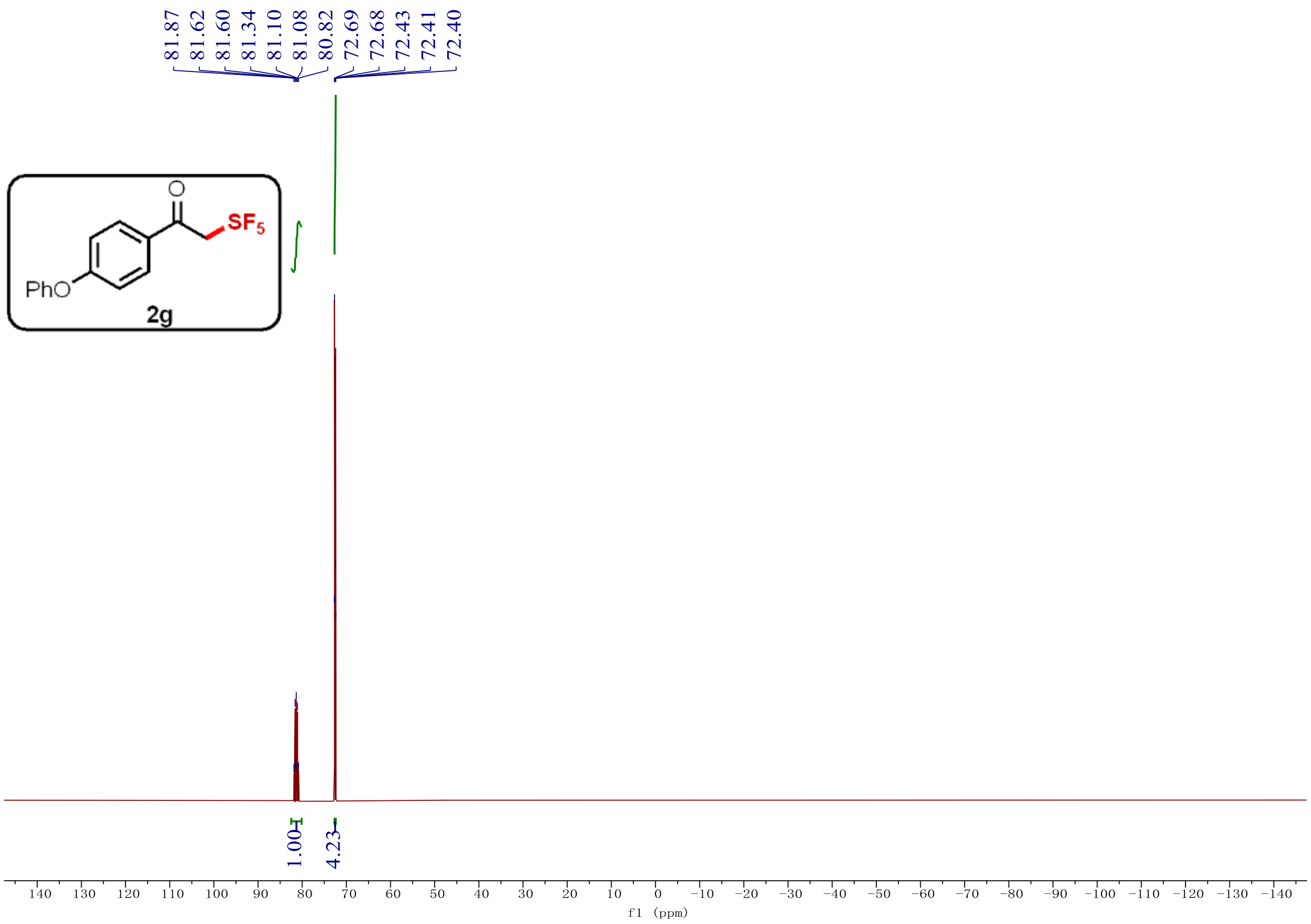

Supplementary Figure 21. ^{19}F NMR Spectrum of Compound 2g (565 MHz, $CDCl_3$, 25 °C)

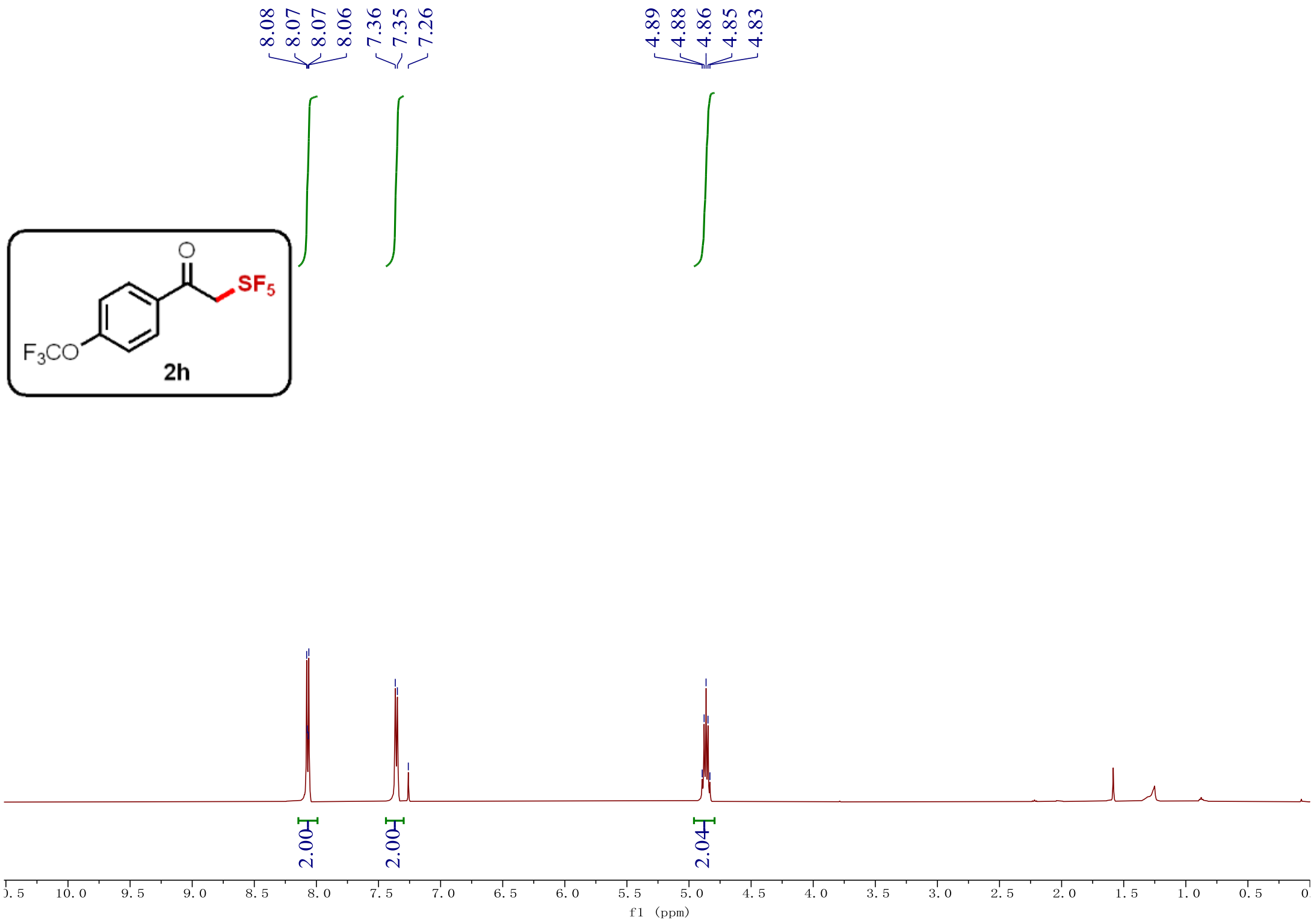

Supplementary Figure 22. ^{1}H NMR Spectrum of Compound 2h (500 MHz, $CDCl_3$, 25 °C)

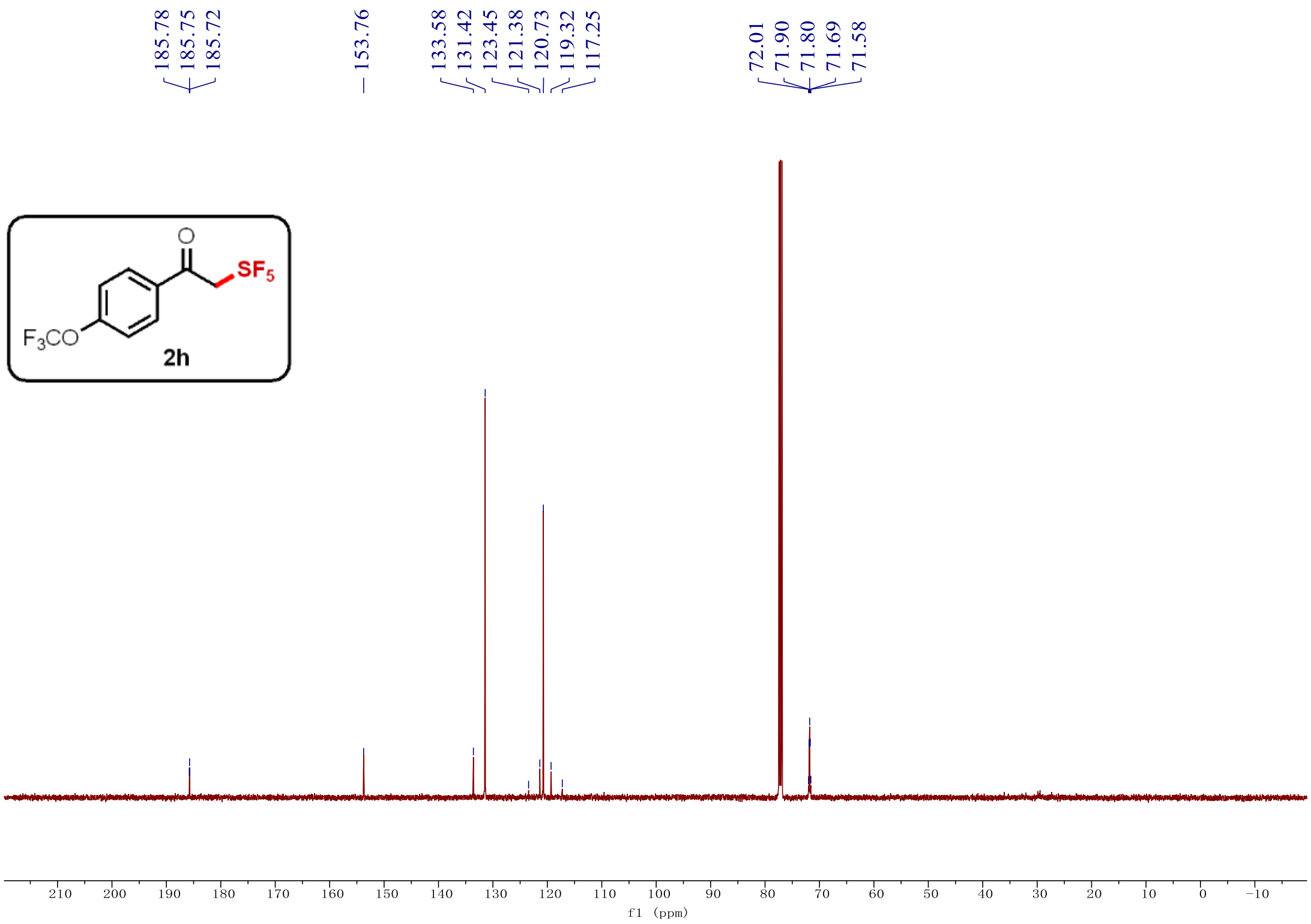

Supplementary Figure 23. ^{13}C NMR Spectrum of Compound 2h (126 MHz, $CDCl_3$, 25 ˚C)

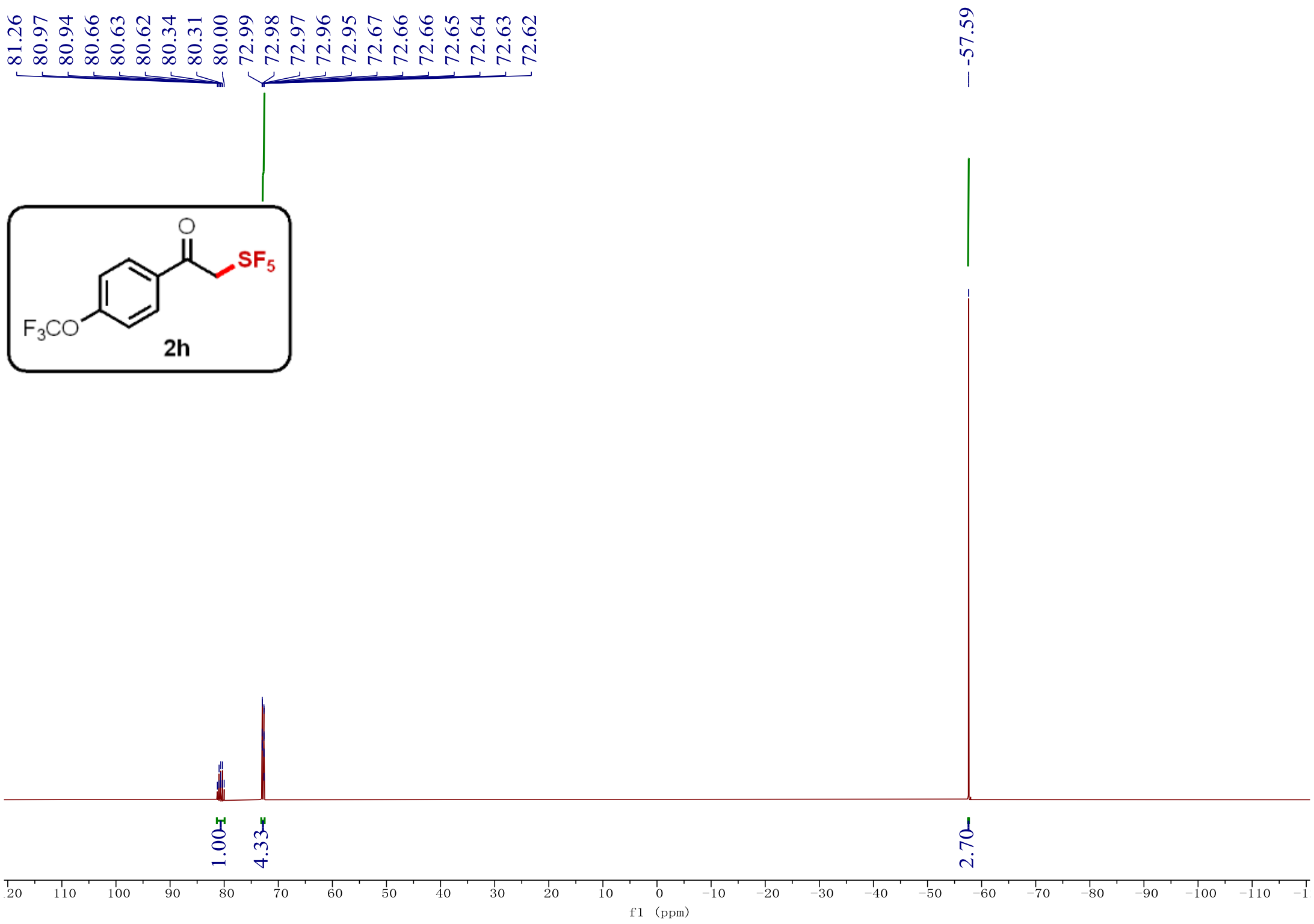

Supplementary Figure 24. ^{19}F NMR Spectrum of Compound 2h (471 MHz, $CDCl_3$, 25 ˚C)

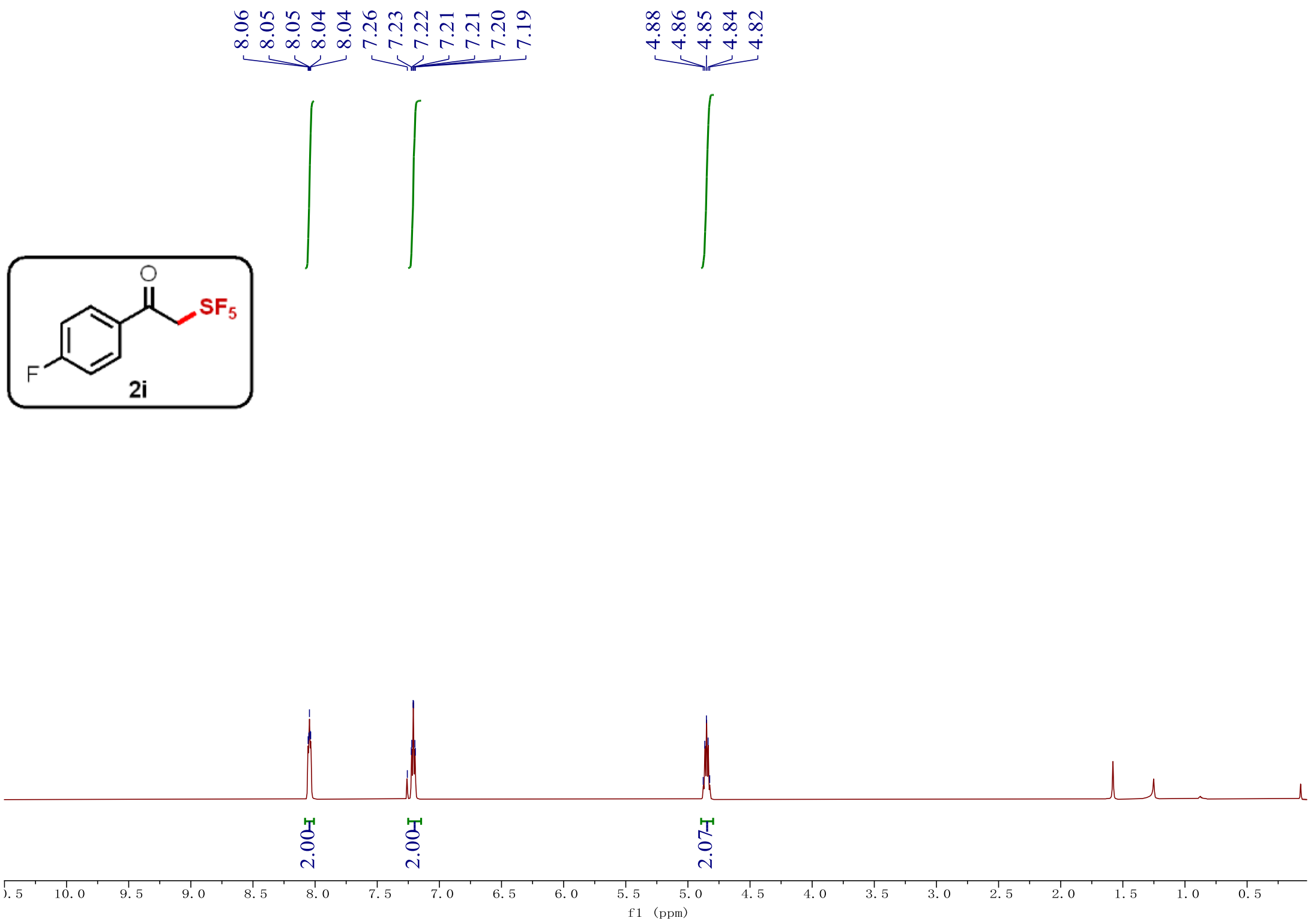

Supplementary Figure 25. ^{1}H NMR Spectrum of Compound 2i (600 MHz, $CDCl_3$, 25 °C)

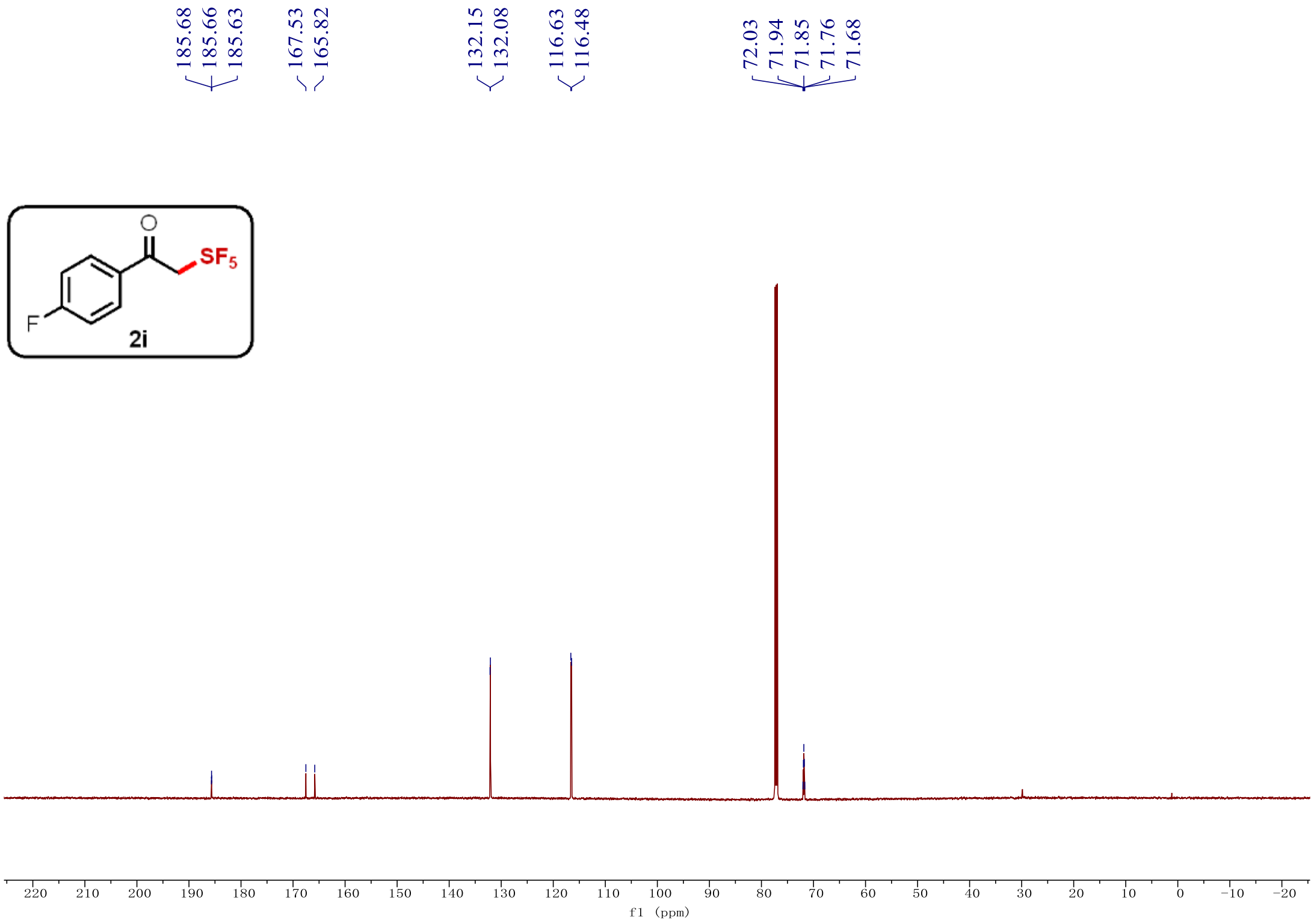

Supplementary Figure 26. ^{13}C NMR Spectrum of Compound 2i (151 MHz, $CDCl_3$, 25 °C)

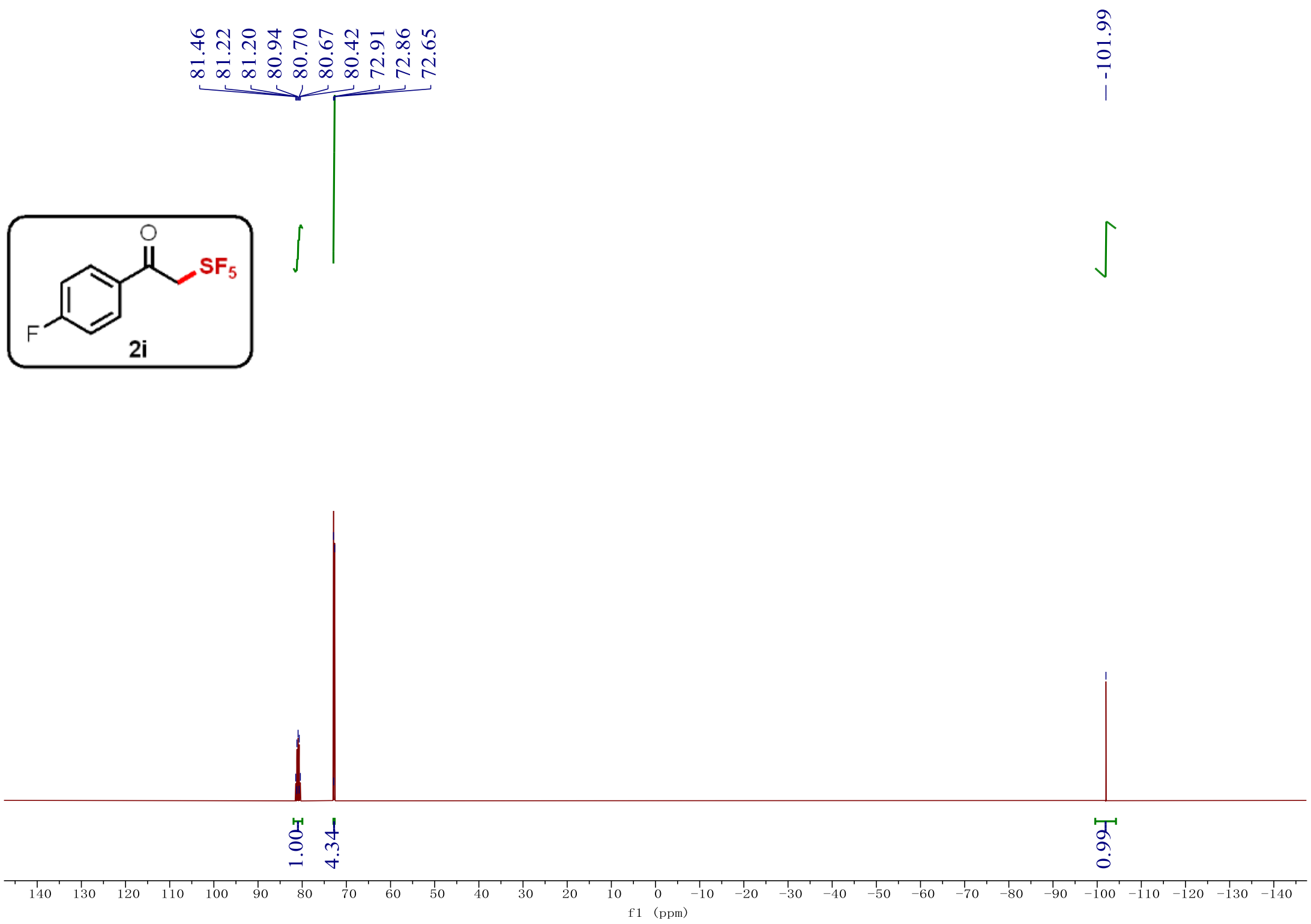

Supplementary Figure 27. ^{19}F NMR Spectrum of Compound 2i (565 MHz, $CDCl_3$, 25 °C)

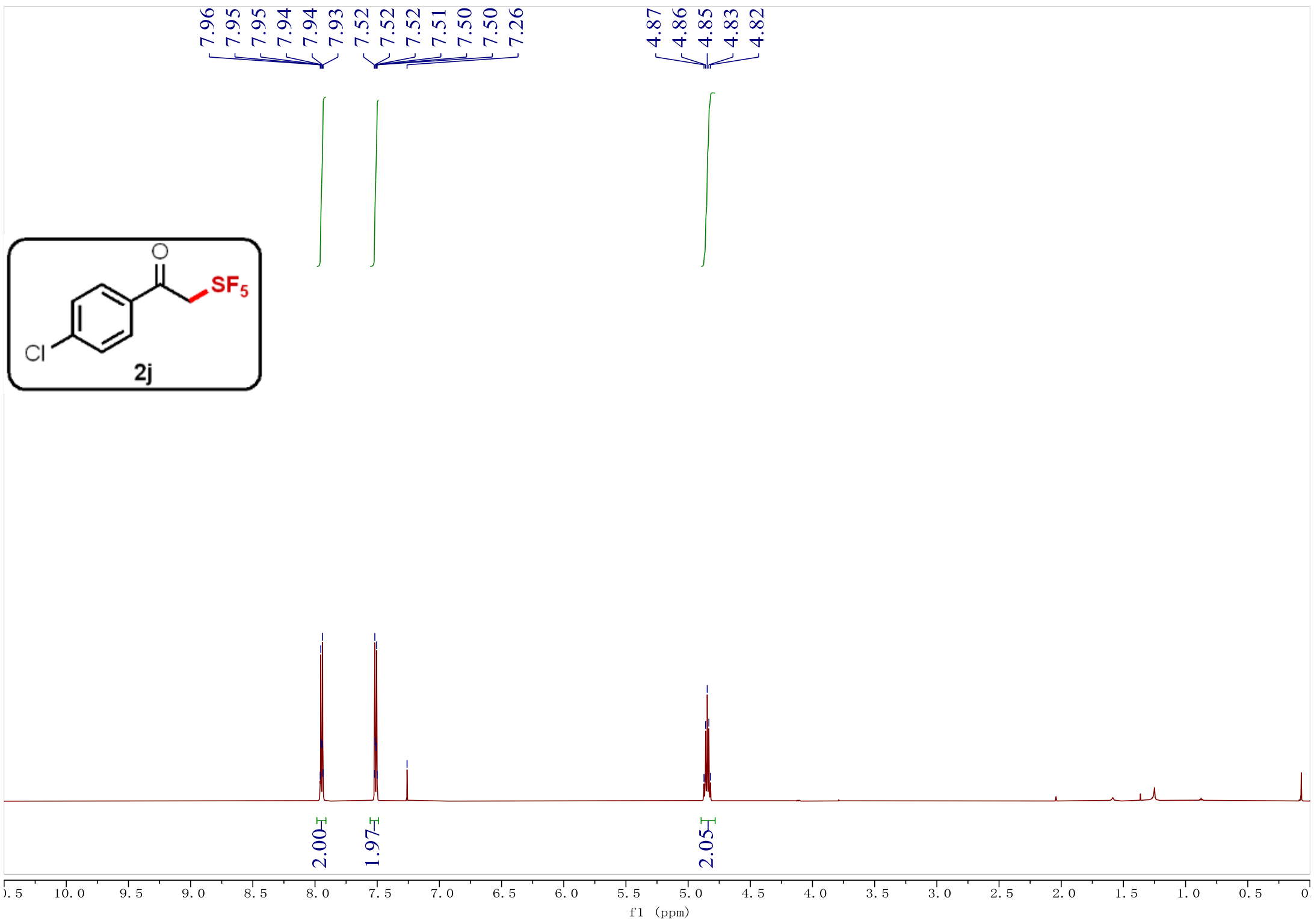

Supplementary Figure 28. ^{1}H NMR Spectrum of Compound 2j (600 MHz, $CDCl_3$, 25 °C)

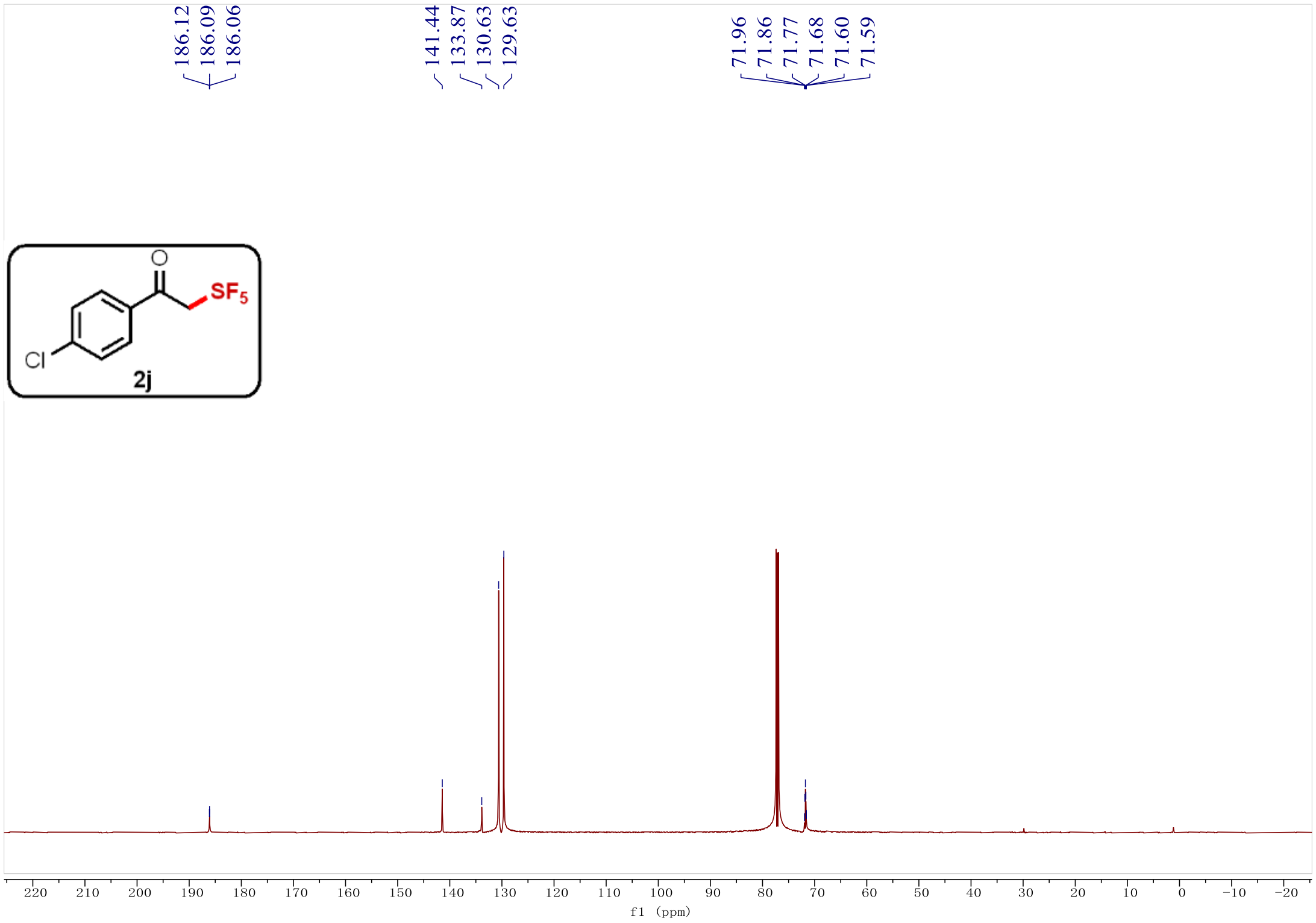

Supplementary Figure 29. ^{13}C NMR Spectrum of Compound 2j (151 MHz, $CDCl_3$, 25 °C)

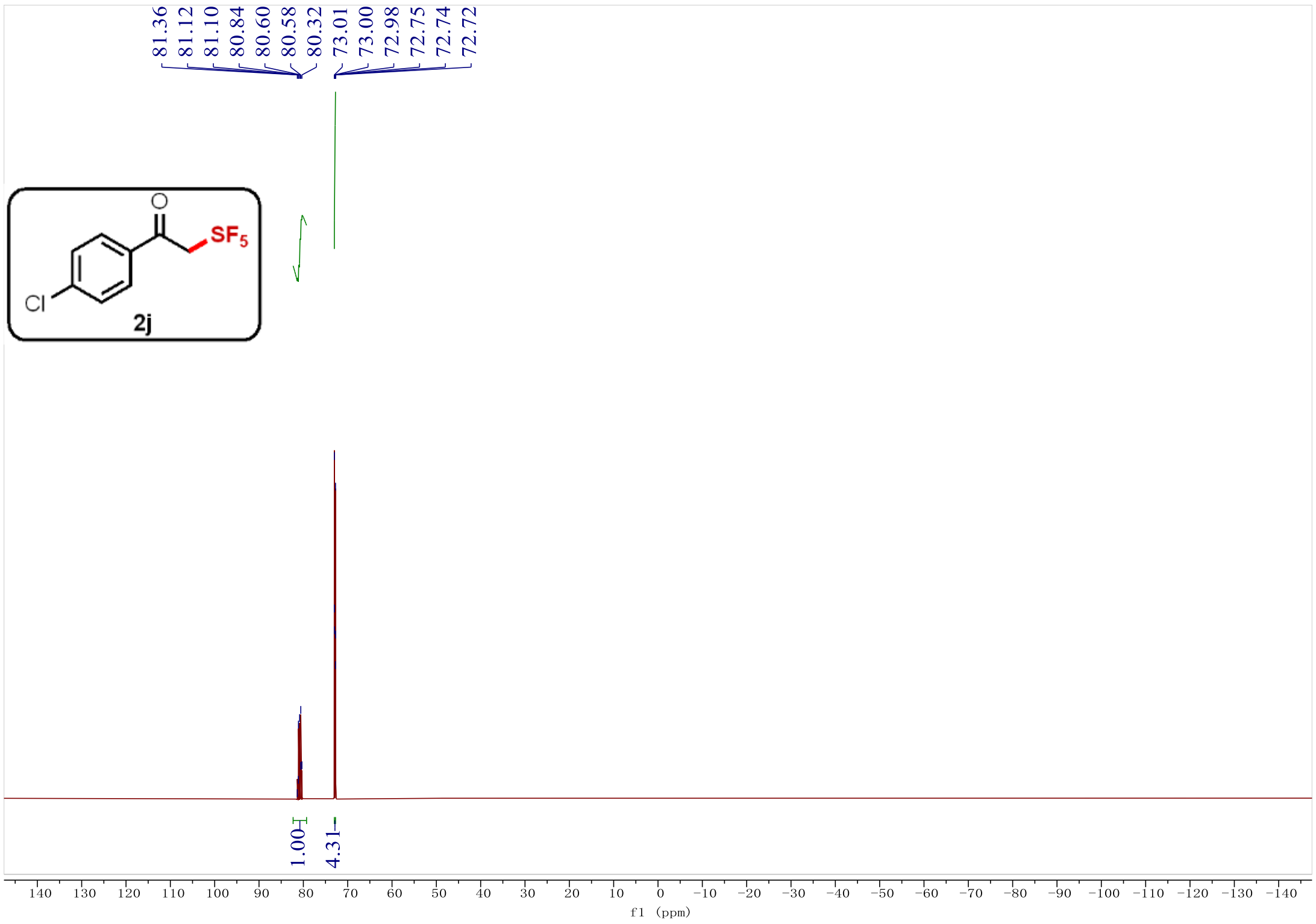

Supplementary Figure 30. ^{19}F NMR Spectrum of Compound 2j (565 MHz, $CDCl_3$, 25 °C)

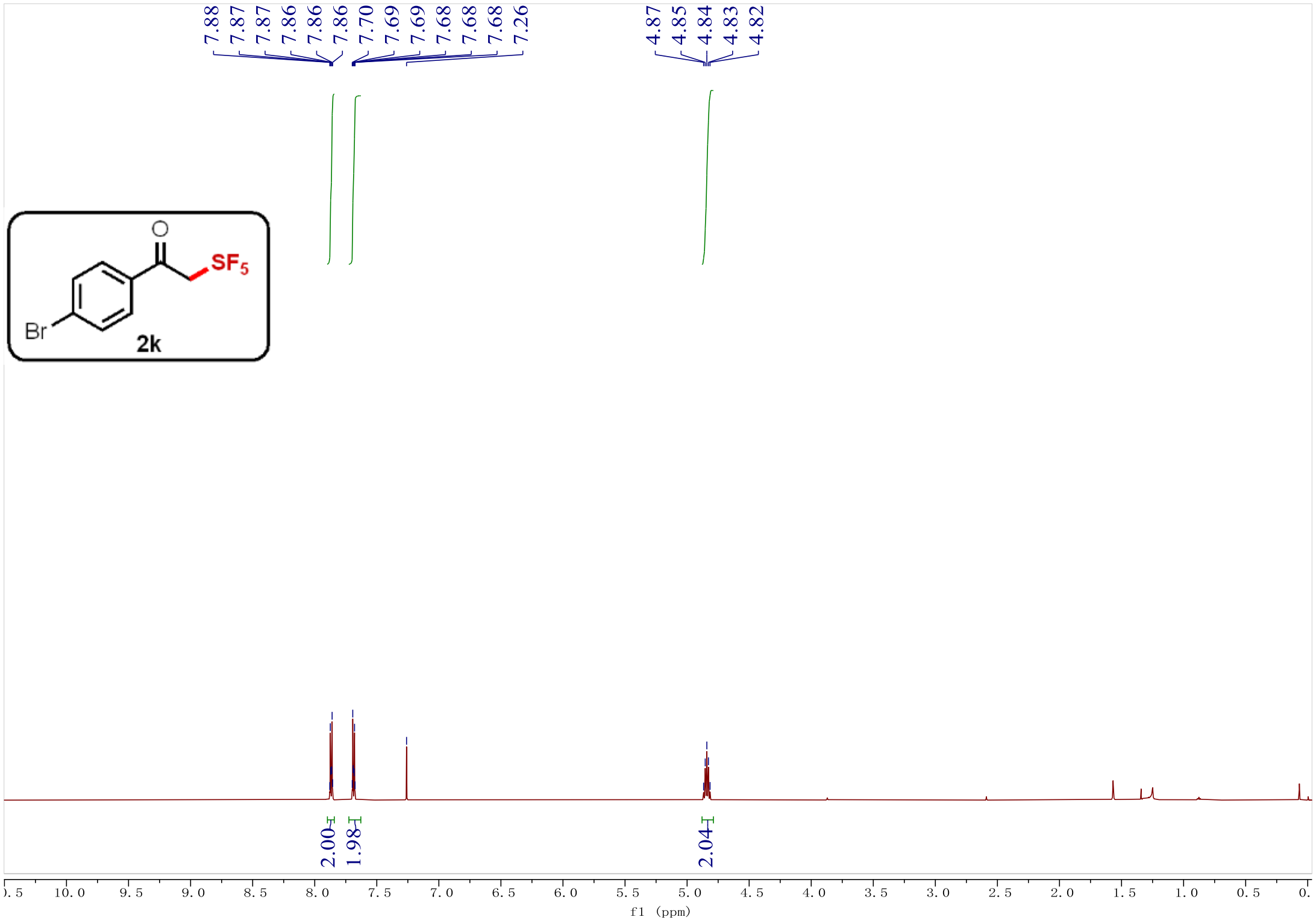

Supplementary Figure 31. ^{1}H NMR Spectrum of Compound 2k (600 MHz, $CDCl_3$, 25 ˚C)

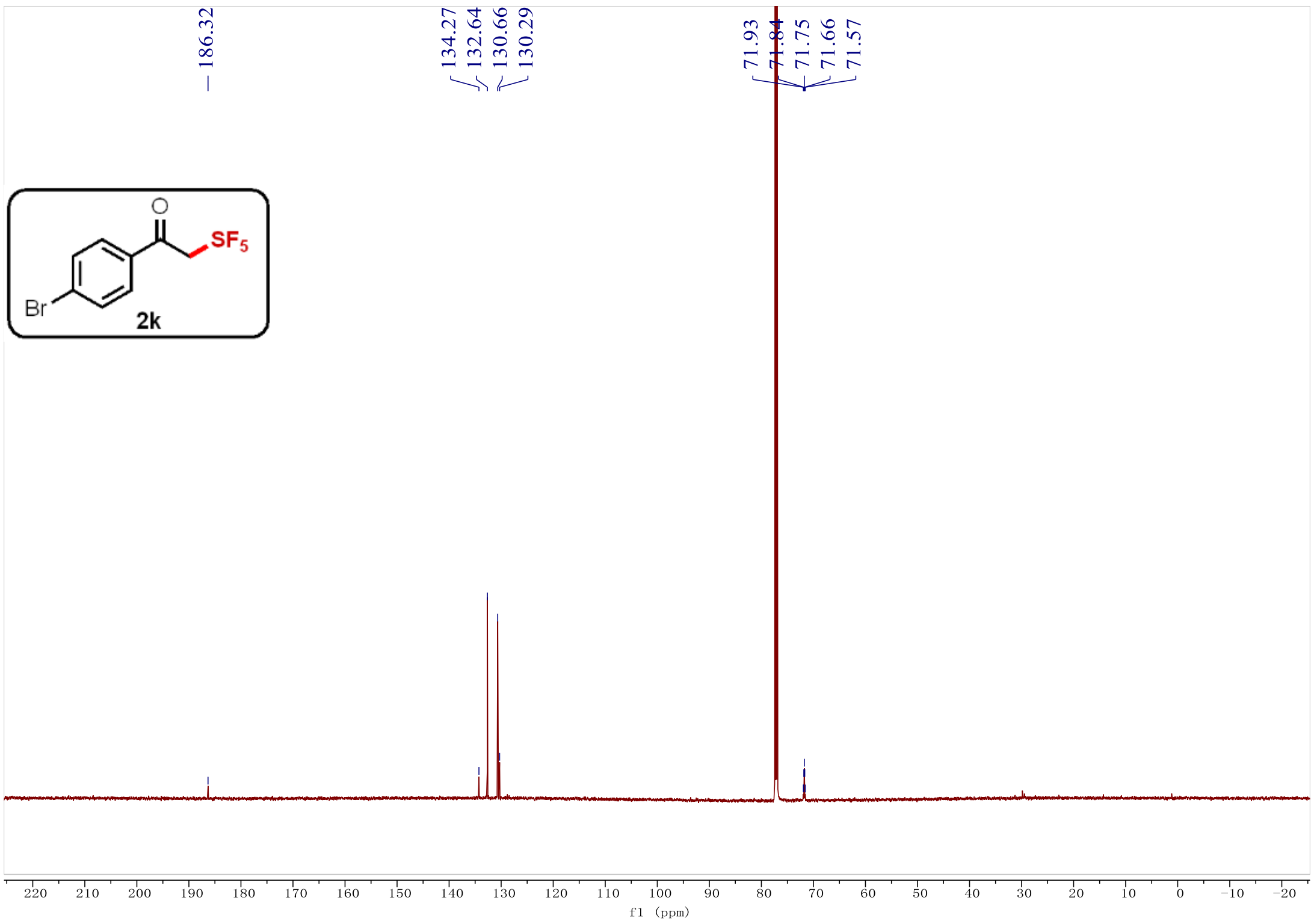

Supplementary Figure 32. ^{13}C NMR Spectrum of Compound 2k (151 MHz, $CDCl_3$, 25 ˚C)

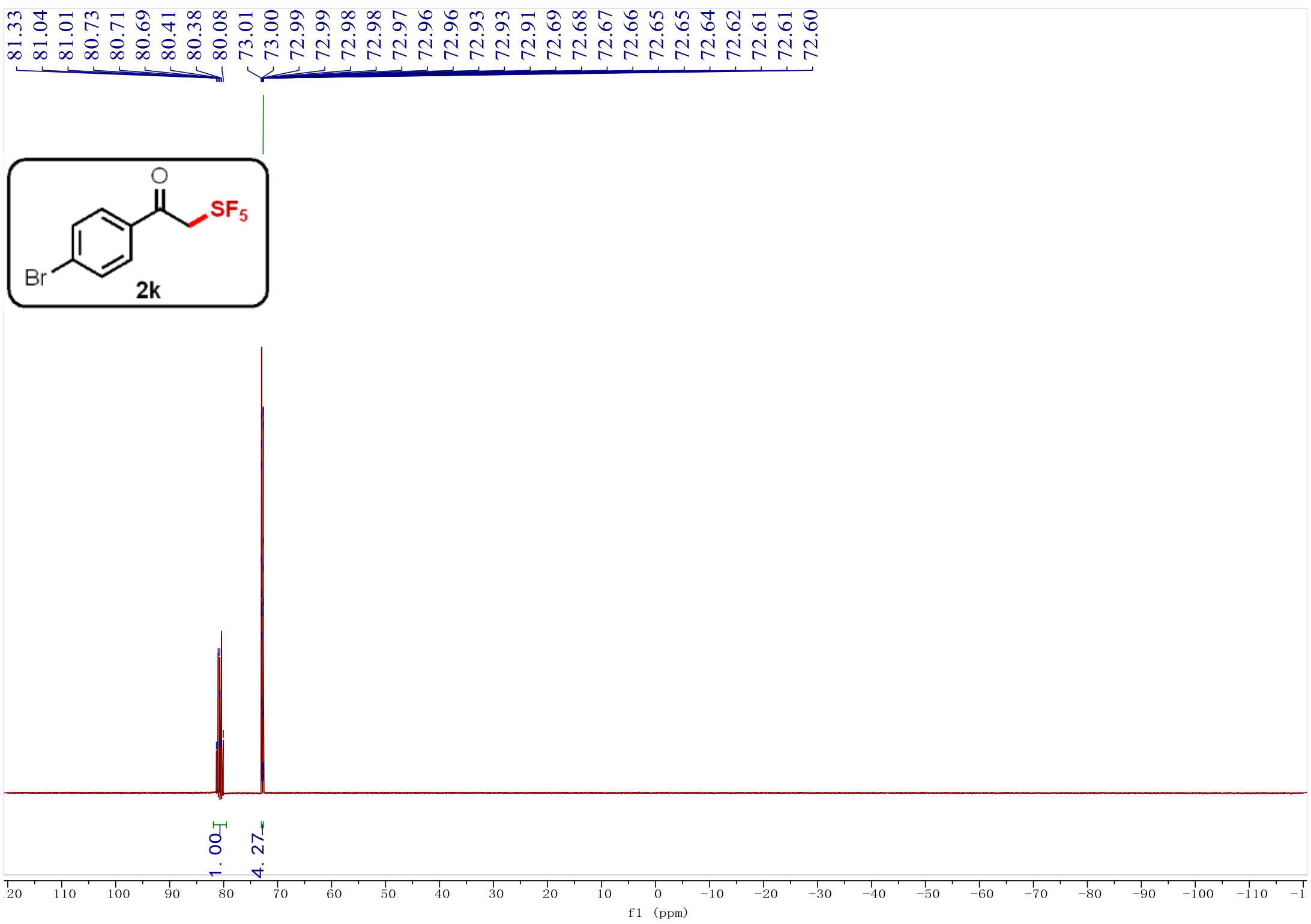

Supplementary Figure 33. ^{19}F NMR Spectrum of Compound 2k (565 MHz, $CDCl_3$, 25 °C)

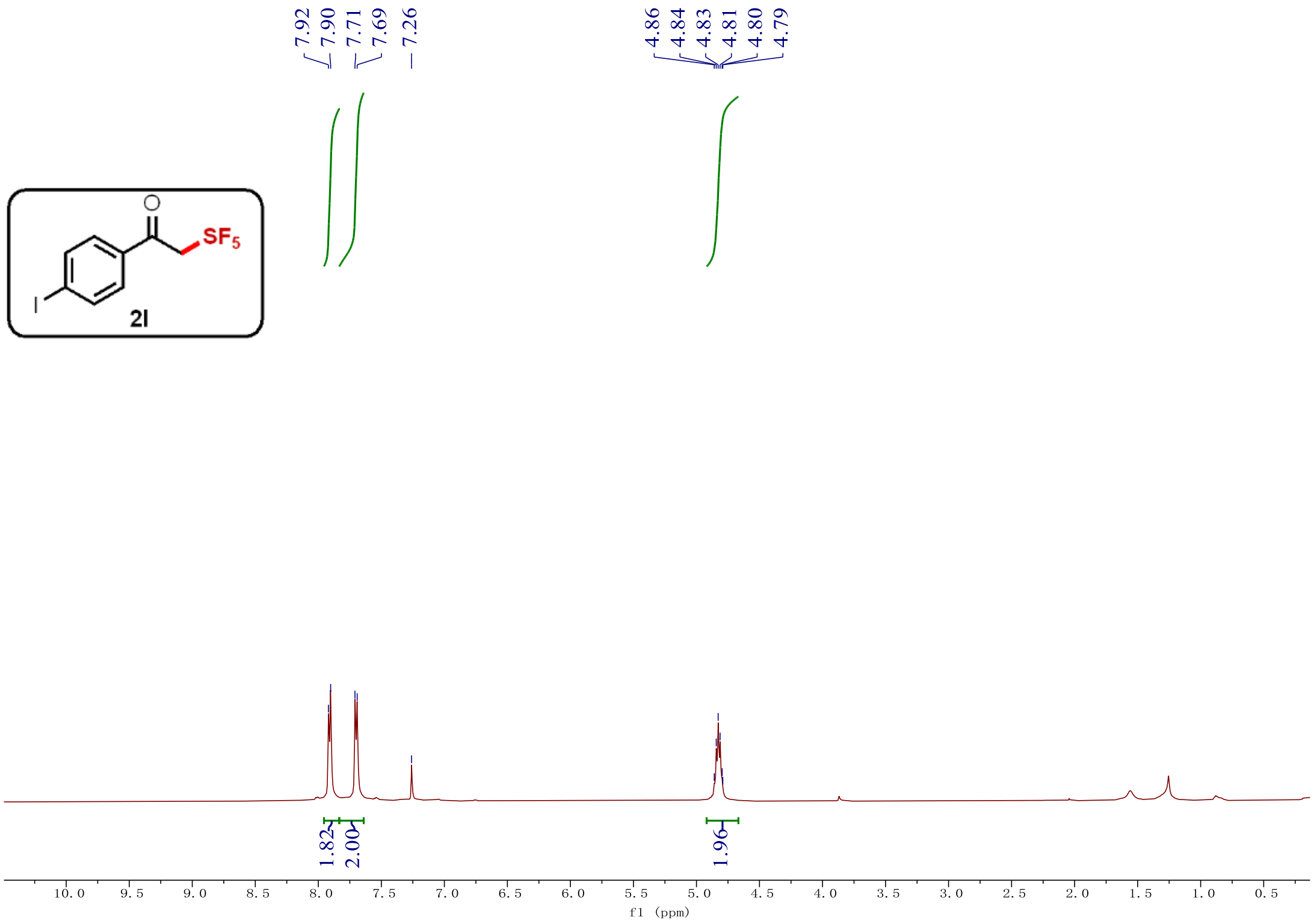

Supplementary Figure 34. ^{1}H NMR Spectrum of Compound 2l (500 MHz, $CDCl_3$, 25 °C)

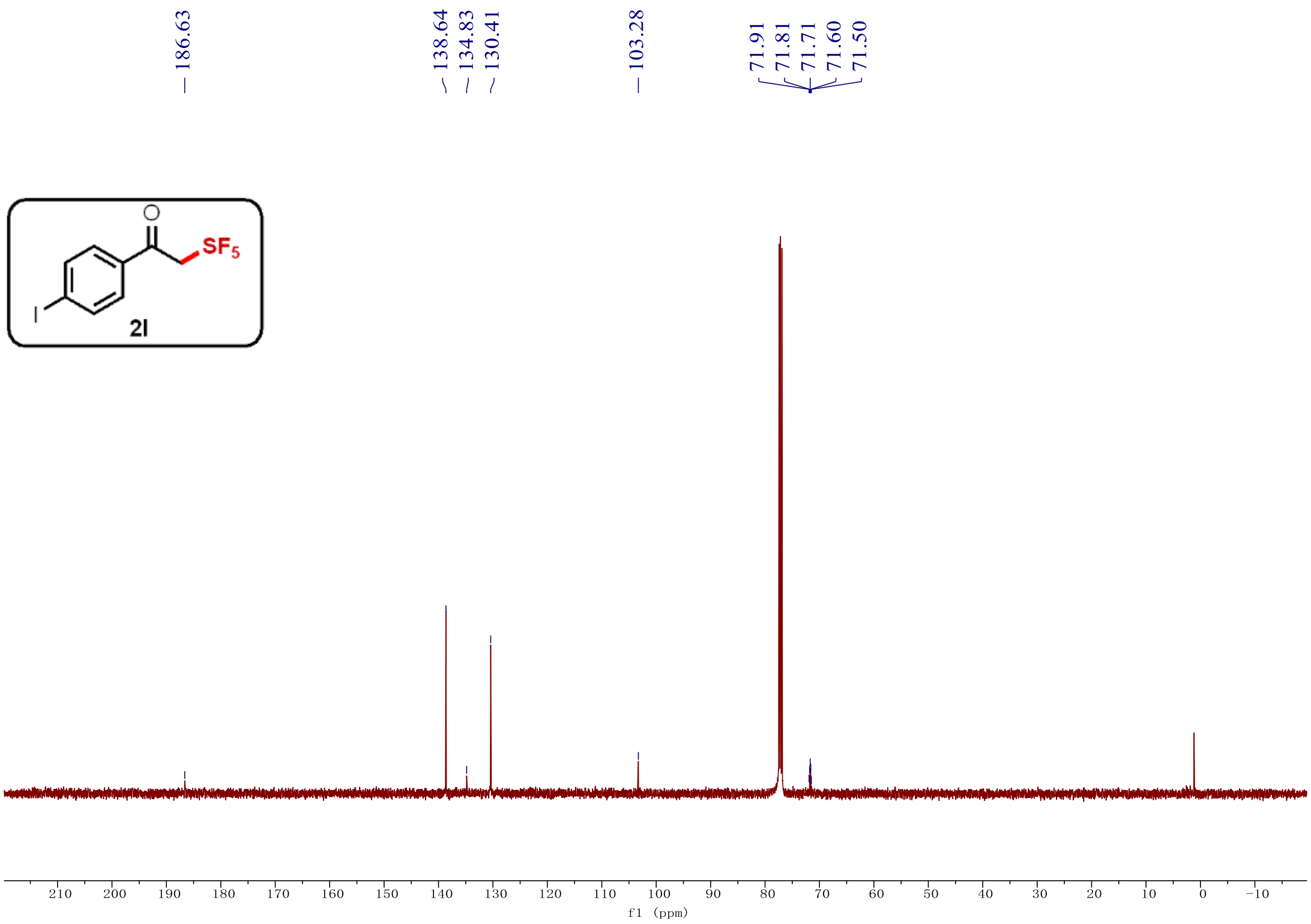

Supplementary Figure 35. ^{13}C NMR Spectrum of Compound 2l (126 MHz, $CDCl_3$, 25 °C)

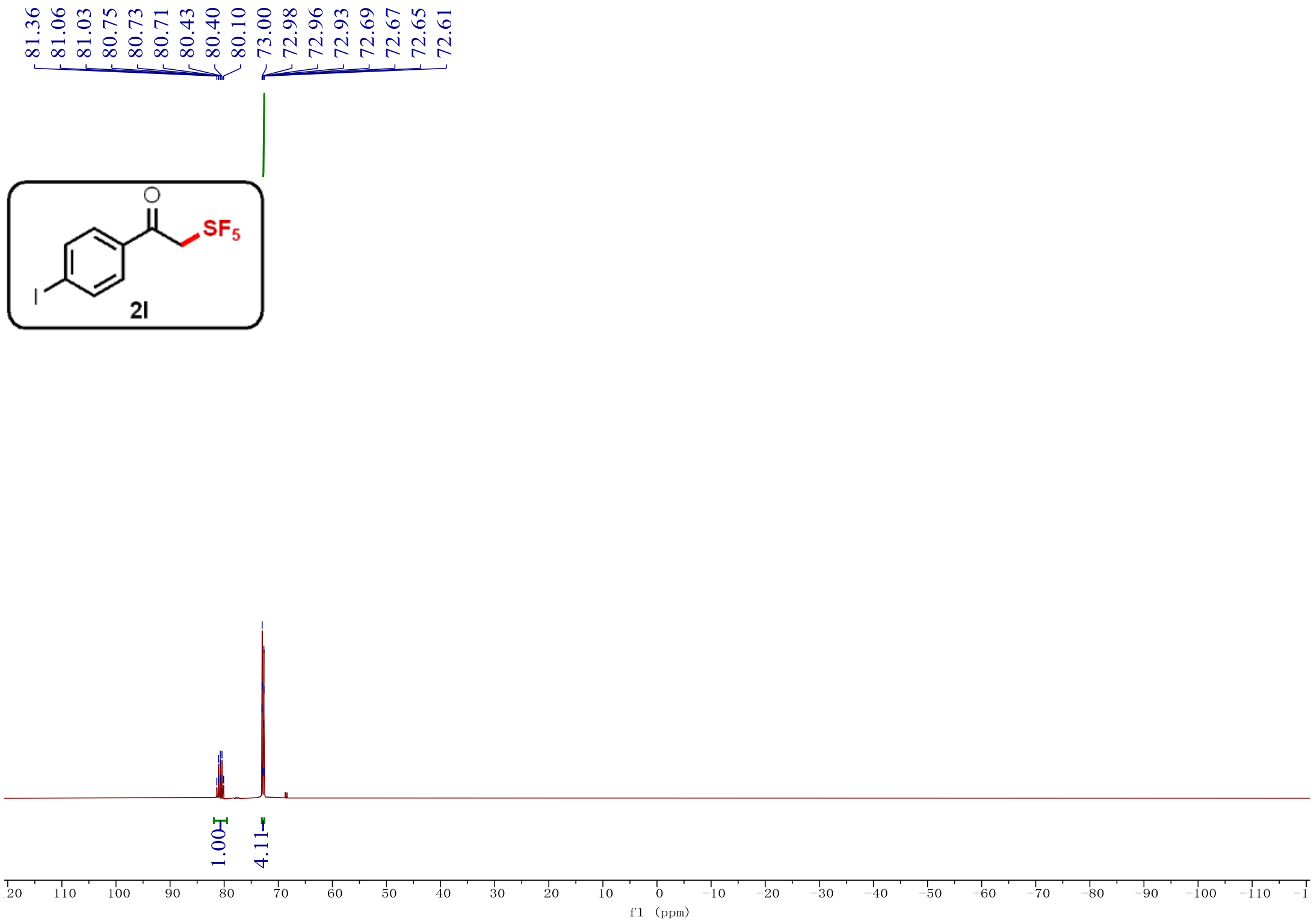

Supplementary Figure 36. ^{19}F NMR Spectrum of Compound 2l (471 MHz, $CDCl_3$, 25 °C)

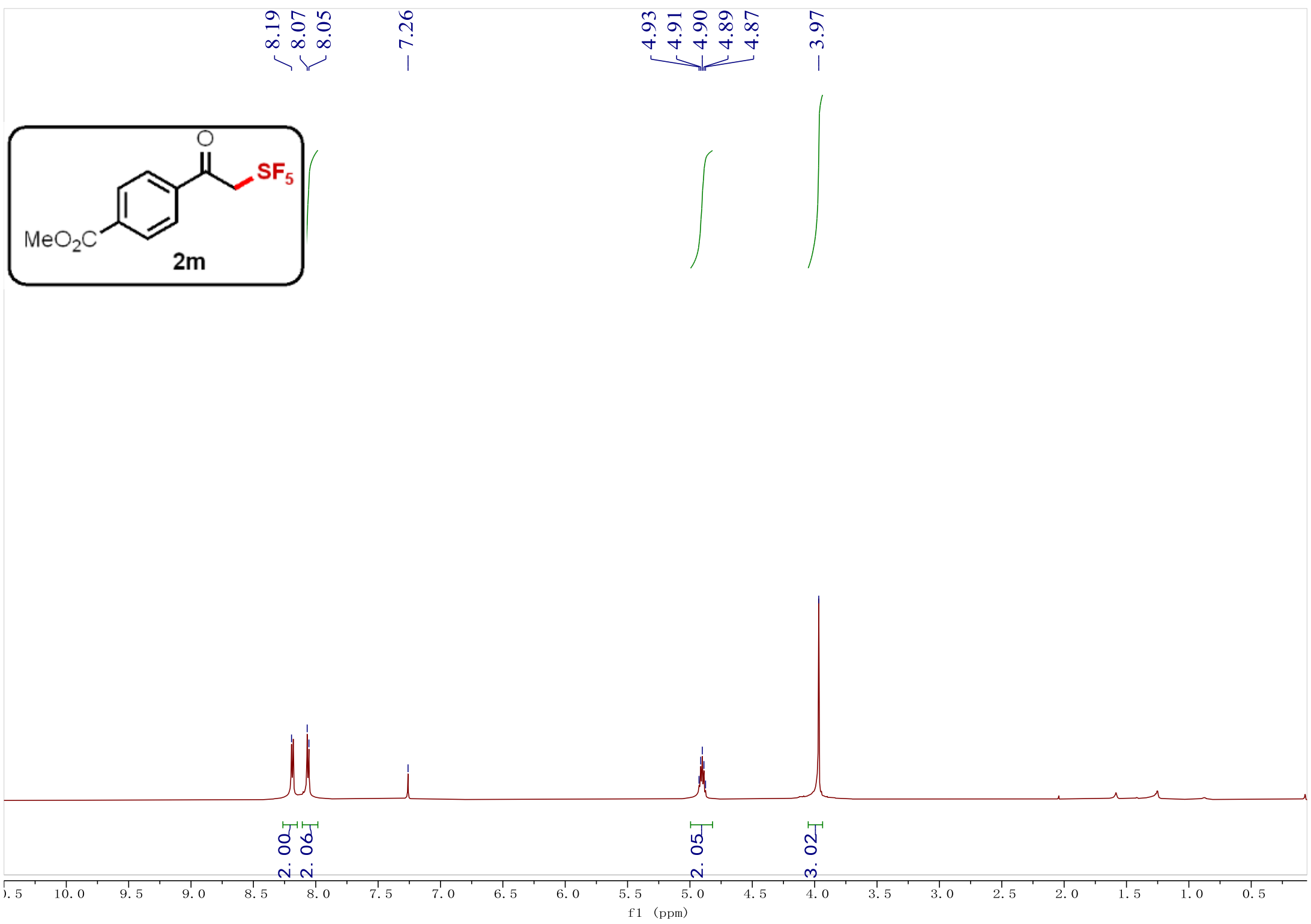

Supplementary Figure 37. ^{1}H NMR Spectrum of Compound 2m (500 MHz, $CDCl_3$, 25 ˚C)

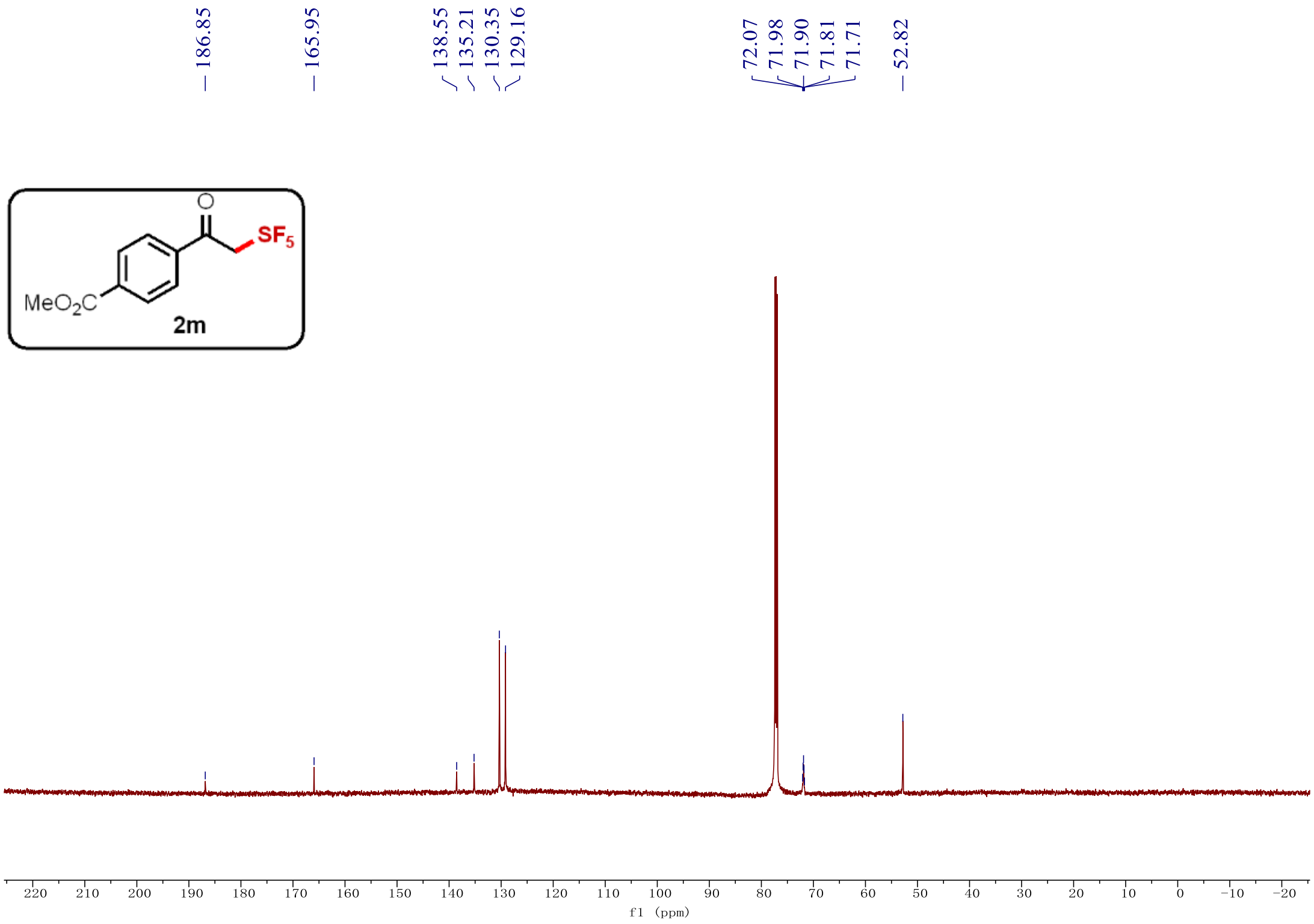

Supplementary Figure 38. ^{13}C NMR Spectrum of Compound 2m (126 MHz, $CDCl_3$, 25 ˚C)

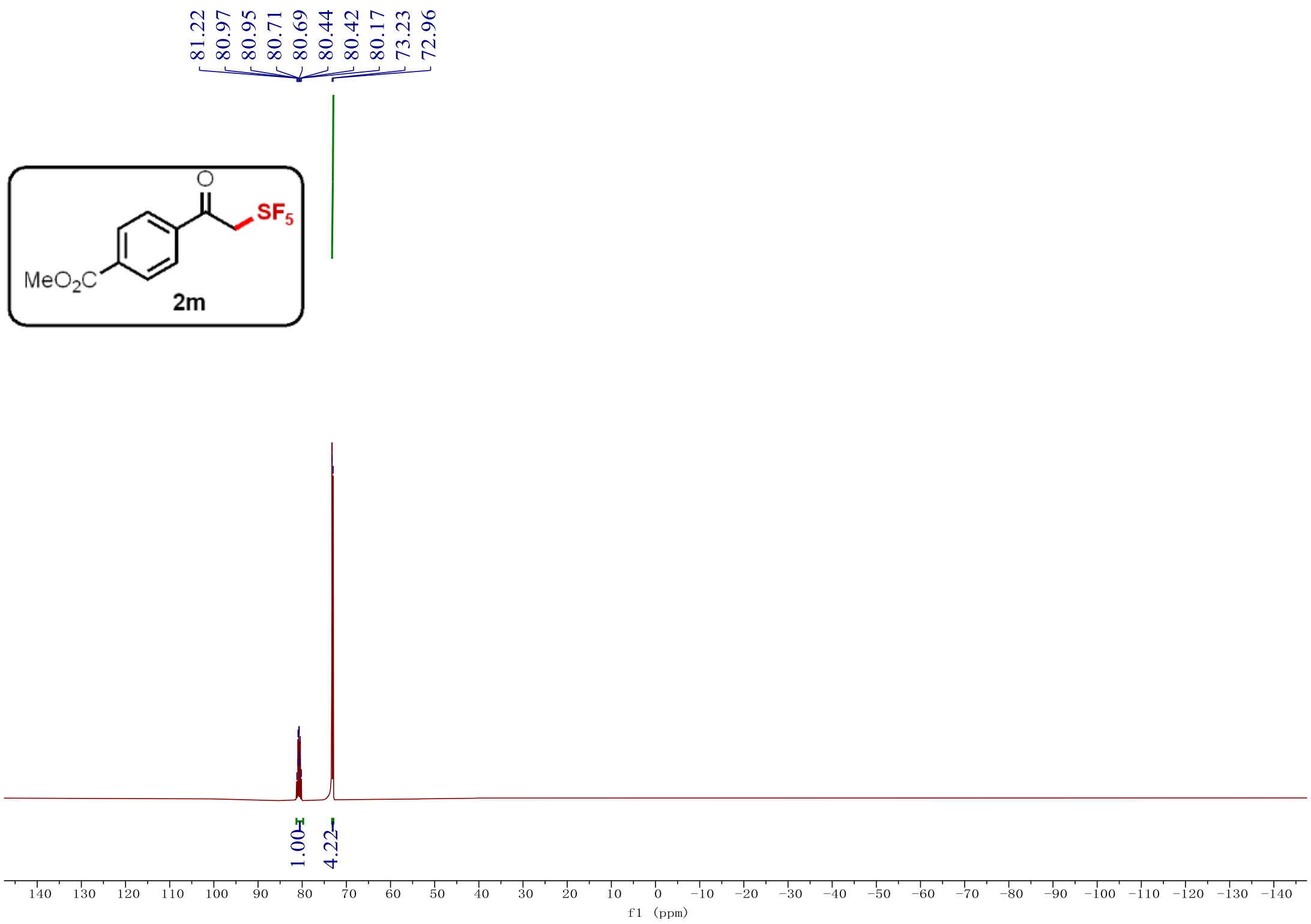

Supplementary Figure 39. ^{19}F NMR Spectrum of Compound 2m (471 MHz, $CDCl_3$, 25 °C)

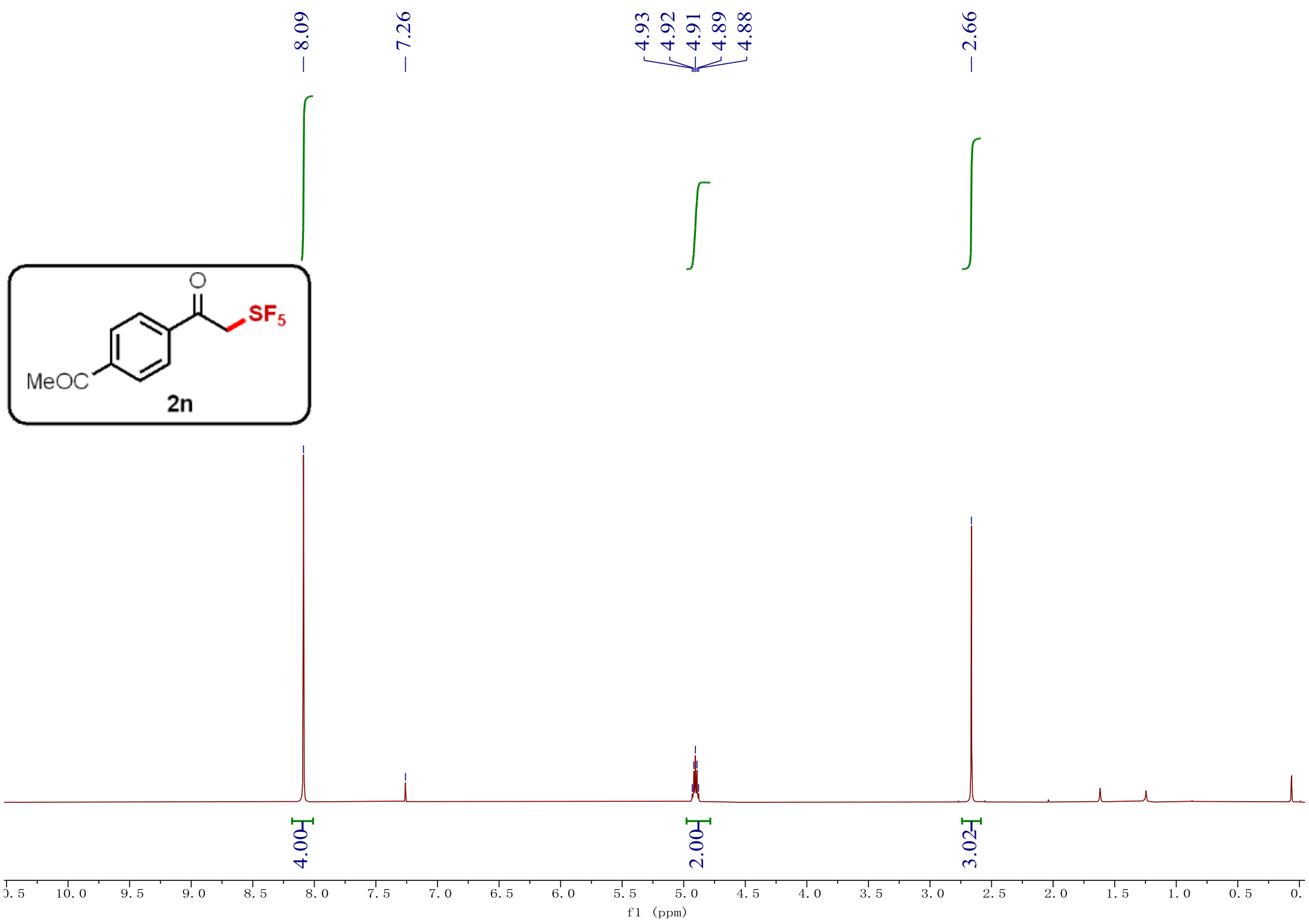

Supplementary Figure 40. ^{1}H NMR Spectrum of Compound 2n (600 MHz, $CDCl_3$, 25 °C)

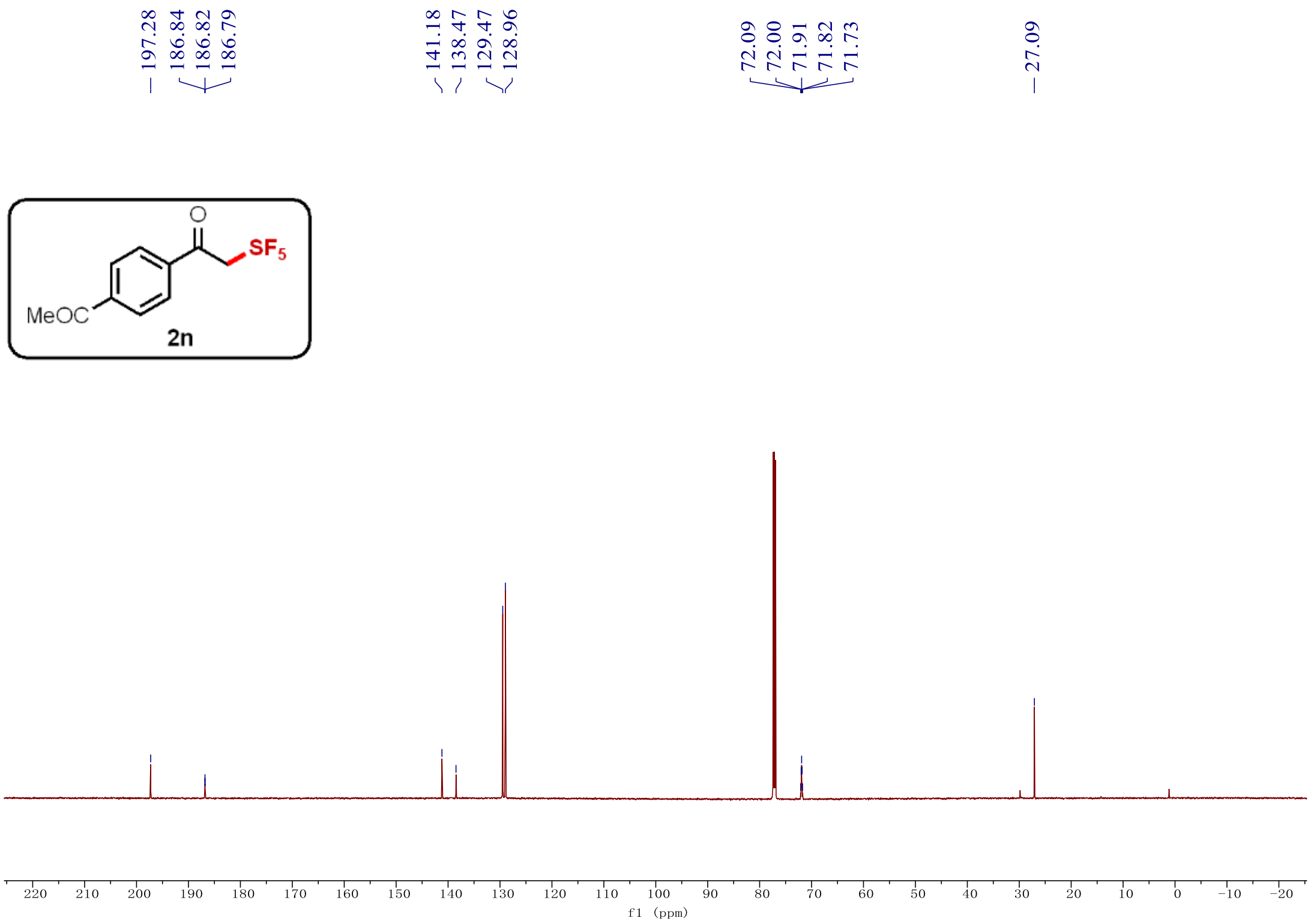

Supplementary Figure 41. ^{13}C NMR Spectrum of Compound 2n (151 MHz, $CDCl_3$, 25 ˚C)

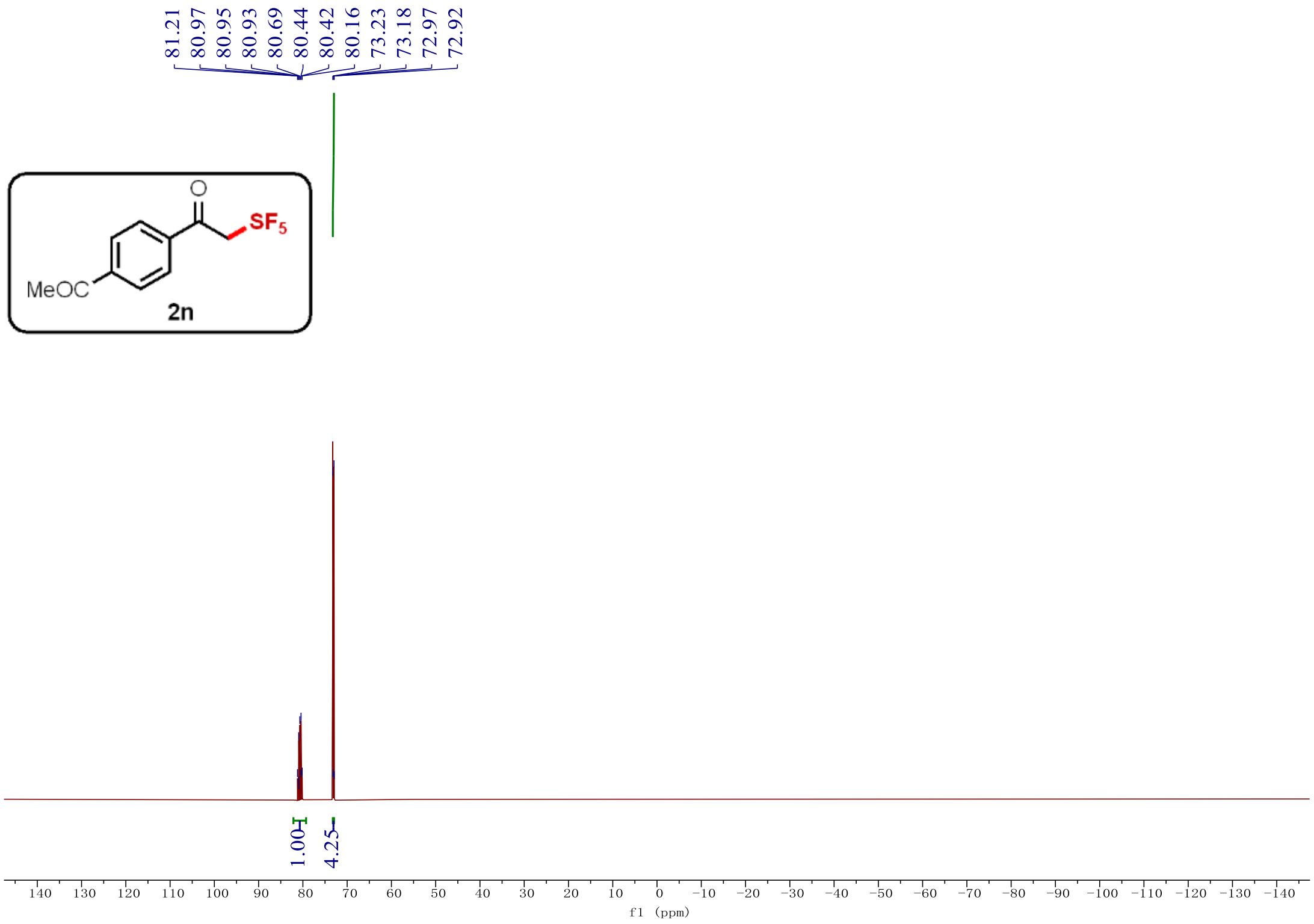

Supplementary Figure 42. ^{19}F NMR Spectrum of Compound 2n (565 MHz, $CDCl_3$, 25 ˚C)

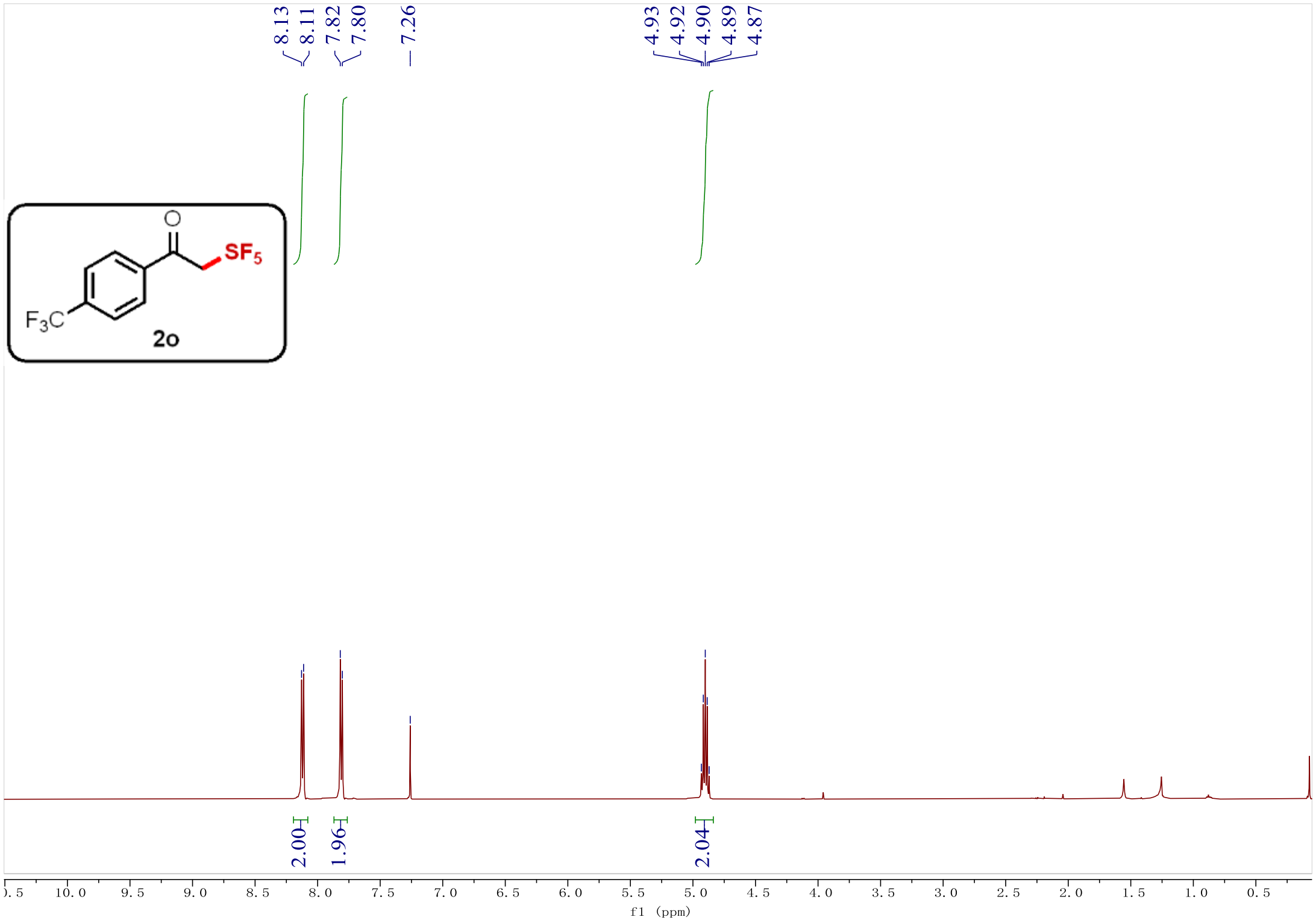

Supplementary Figure 43. ^{1}H NMR Spectrum of Compound 2o (500 MHz, $CDCl_3$, 25 ˚C)

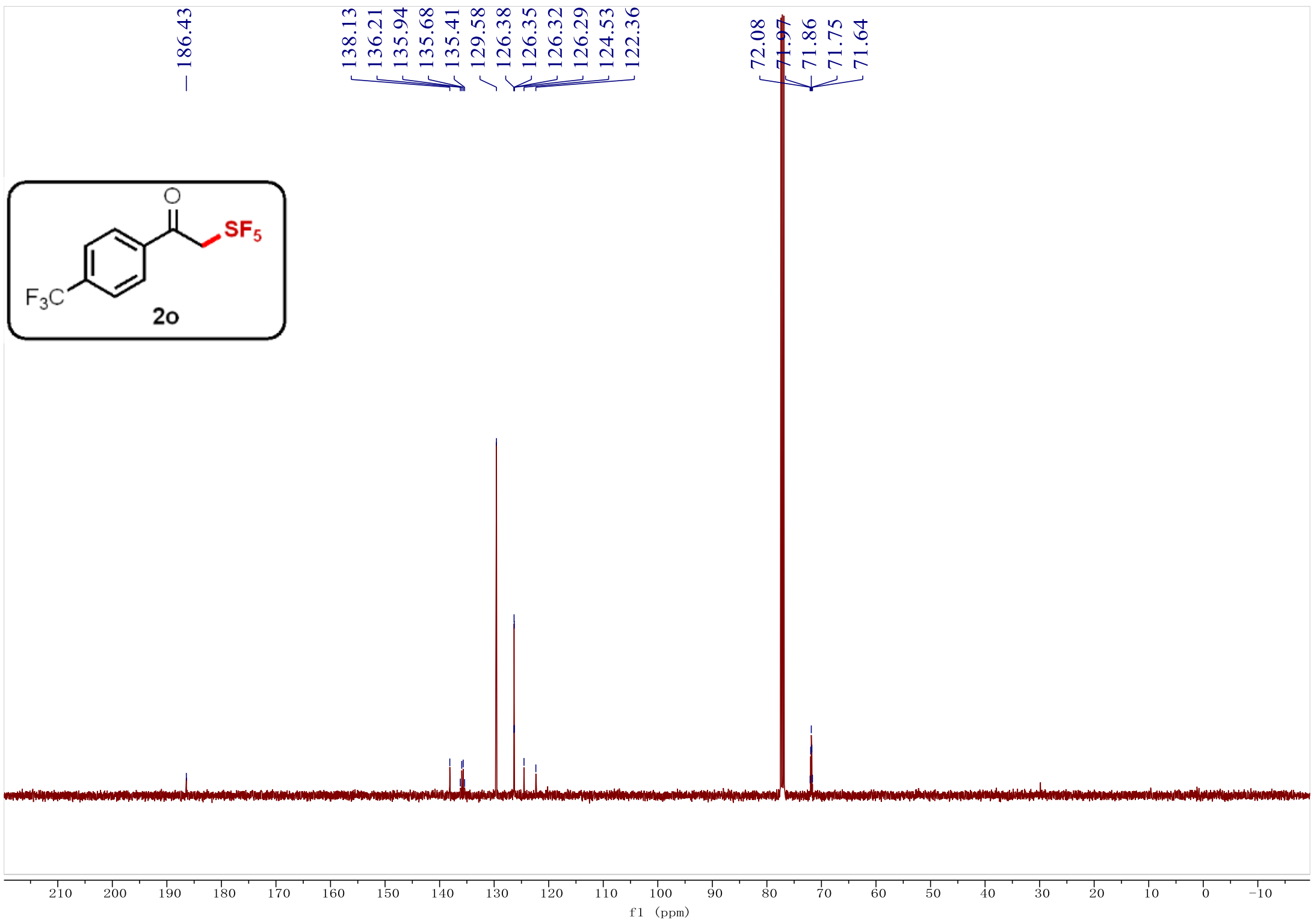

Supplementary Figure 44. ^{13}C NMR Spectrum of Compound 2o (126 MHz, $CDCl_3$, 25 ˚C)

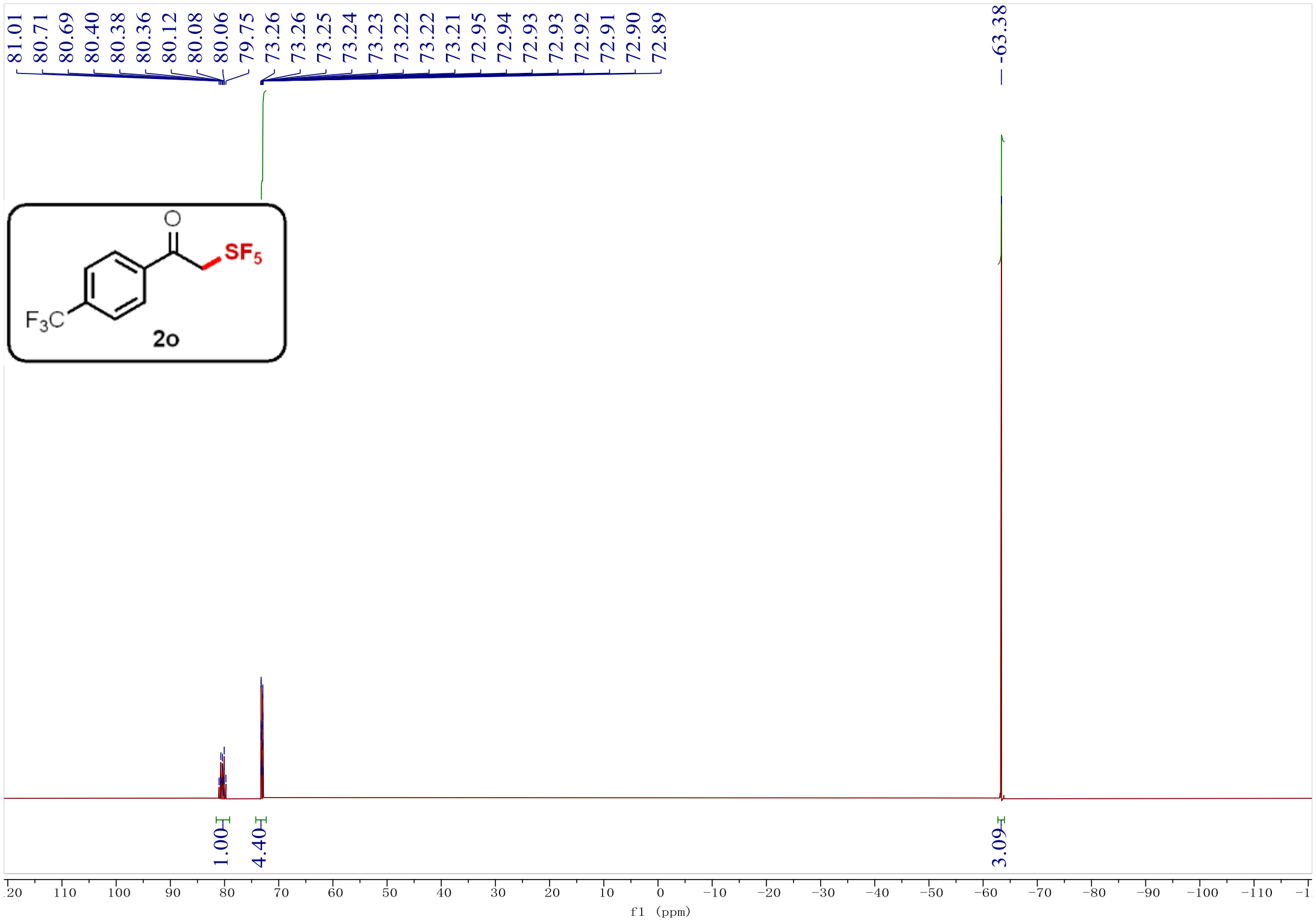

Supplementary Figure 45. **^{19}F NMR Spectrum of Compound 2o (471 MHz, $CDCl_3$, 25 °C)**

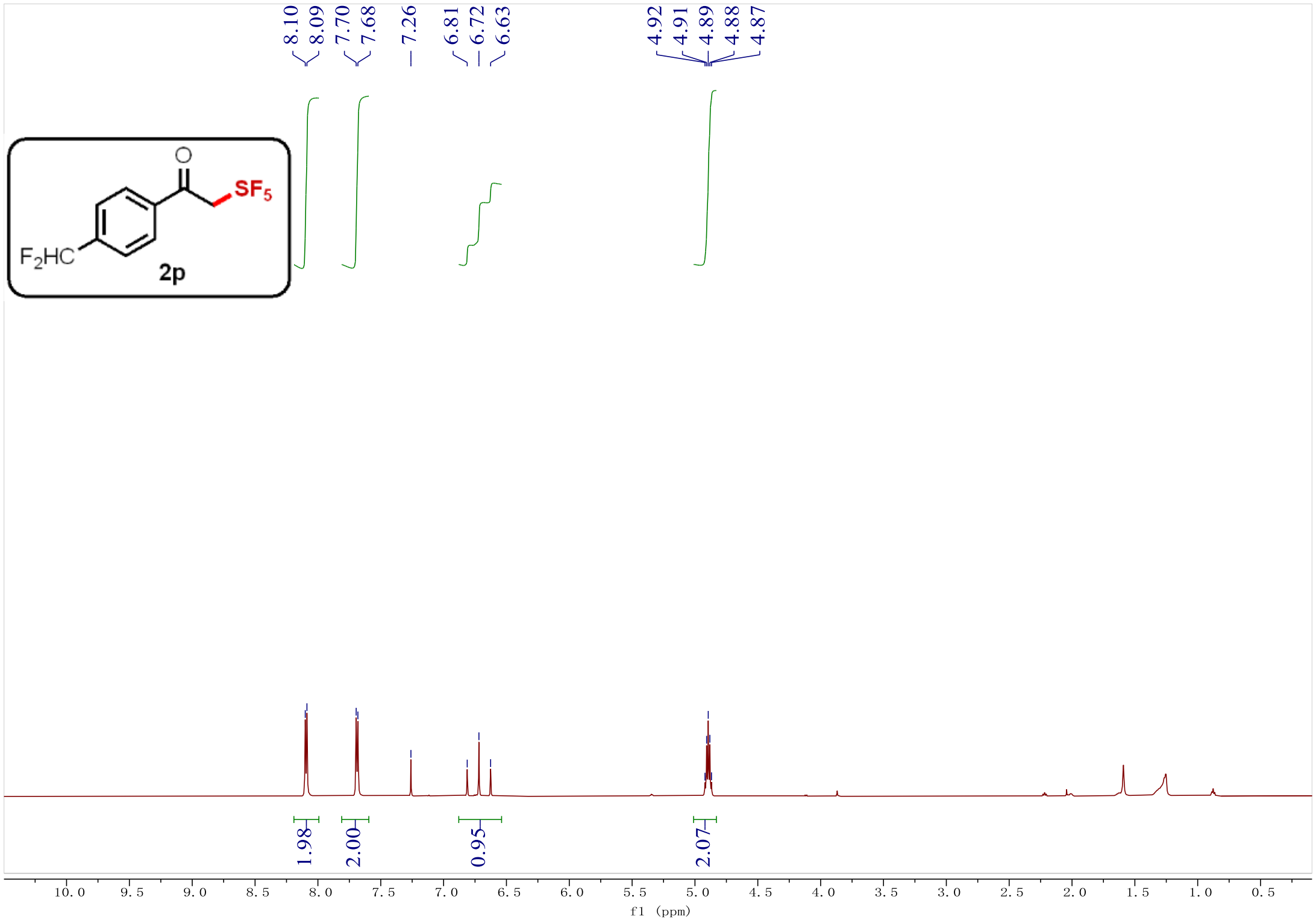

Supplementary Figure 46. **^{1}H NMR Spectrum of Compound 2p (600 MHz, $CDCl_3$, 25 °C)**

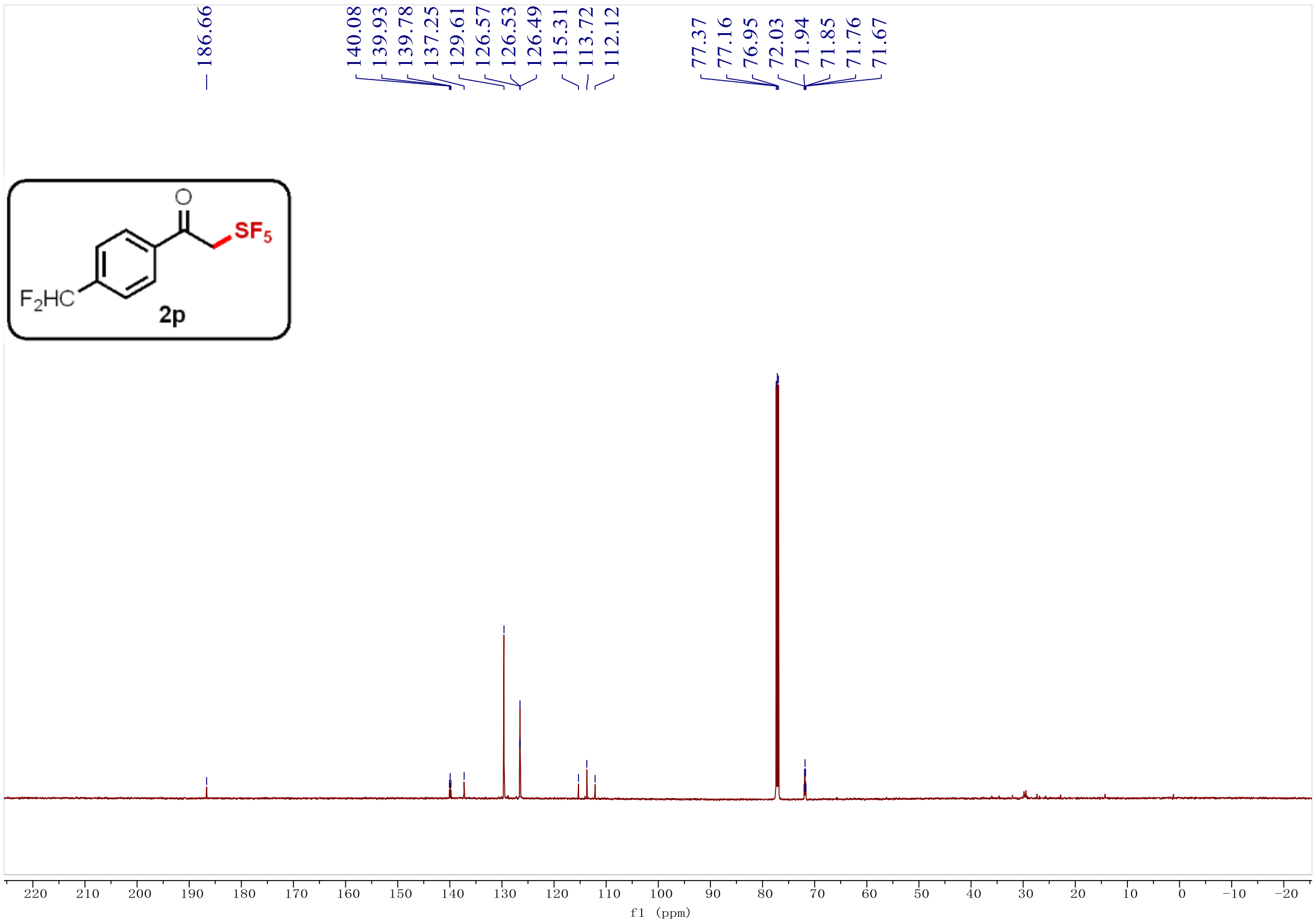

Supplementary Figure 47. ^{13}C NMR Spectrum of Compound 2p (151 MHz, $CDCl_3$, 25 ˚C)

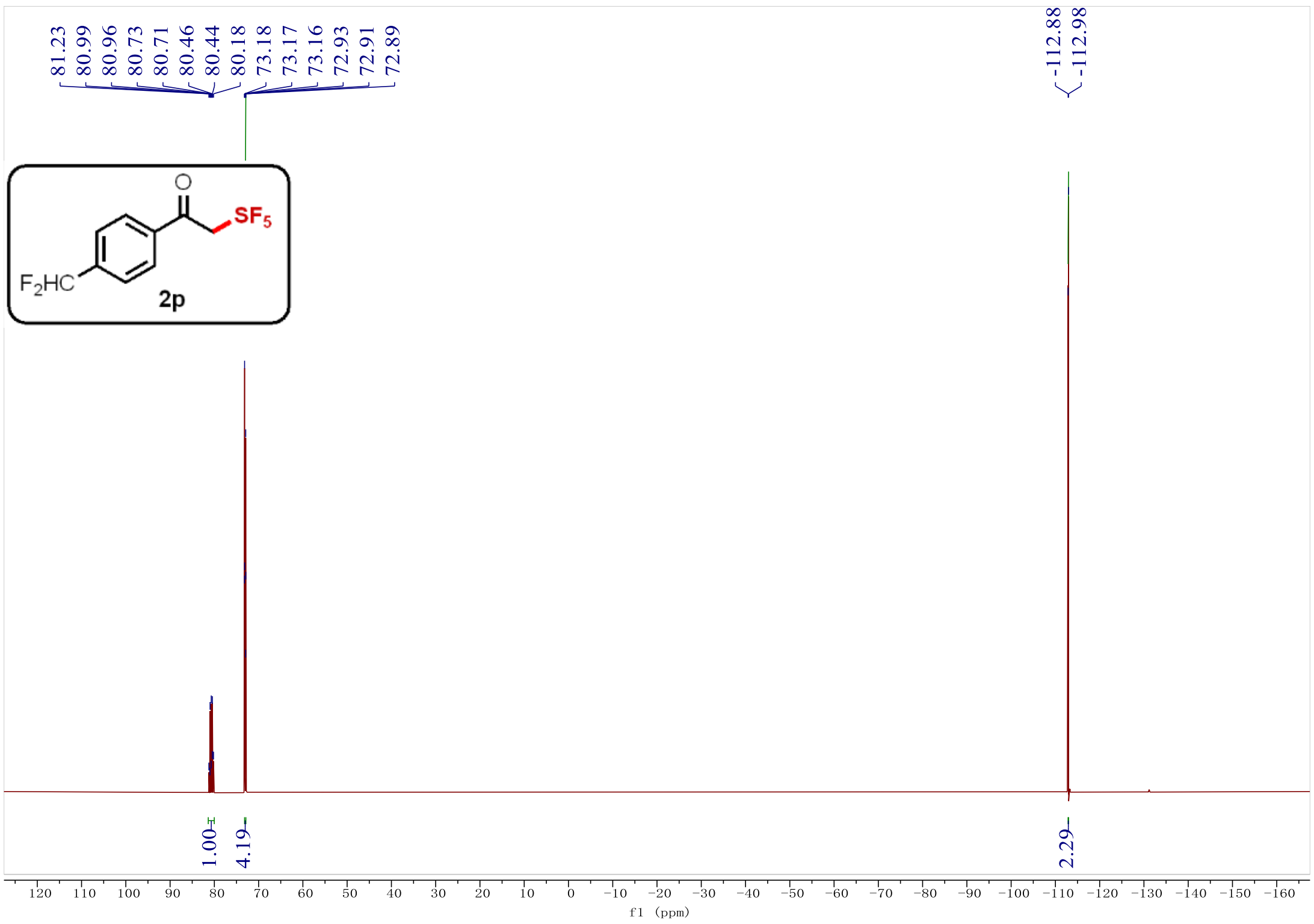

Supplementary Figure 48. ^{19}F NMR Spectrum of Compound 2p (565 MHz, $CDCl_3$, 25 ˚C)

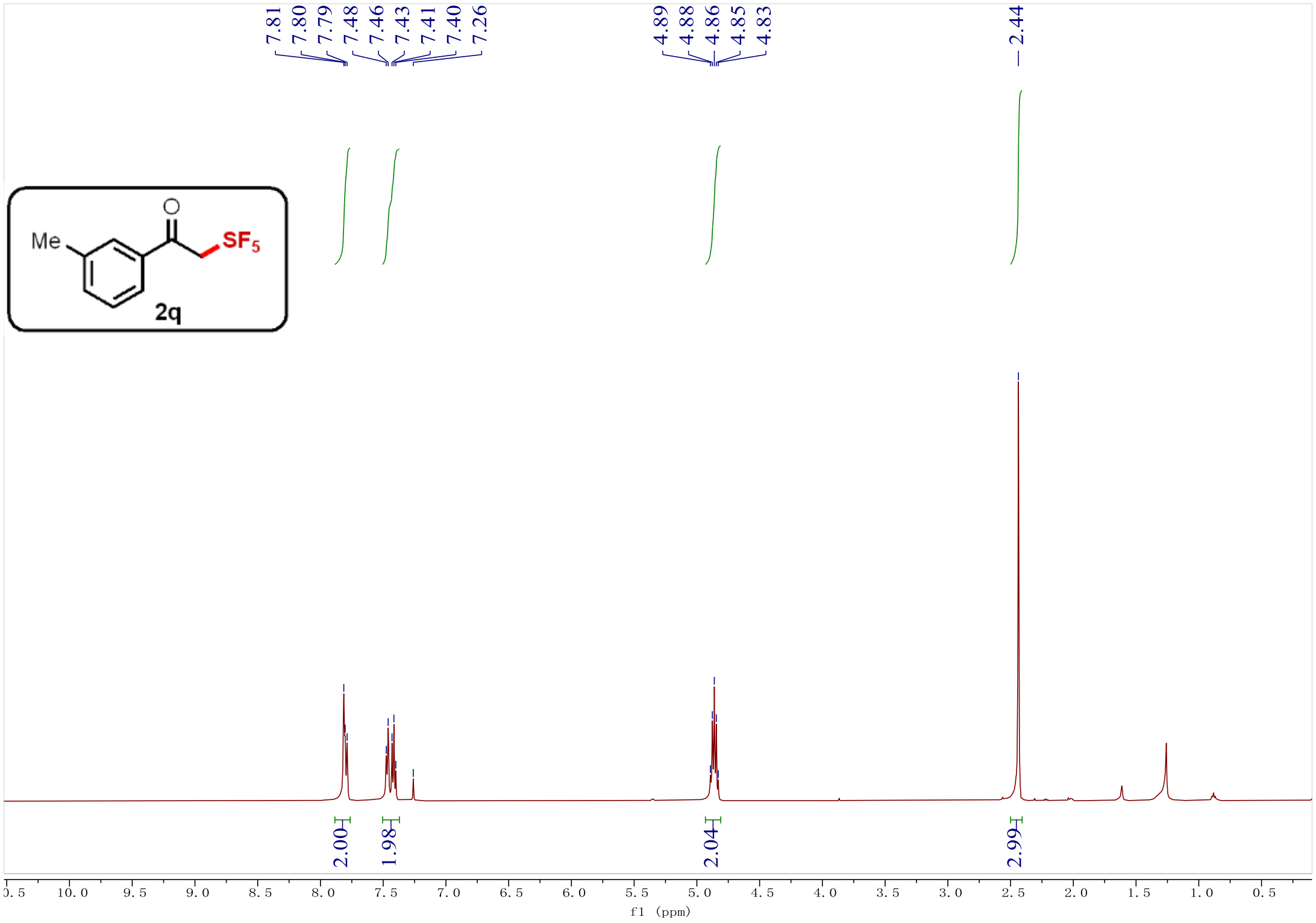

Supplementary Figure 49. ^{1}H NMR Spectrum of Compound 2q (500 MHz, $CDCl_3$, 25 ˚C)

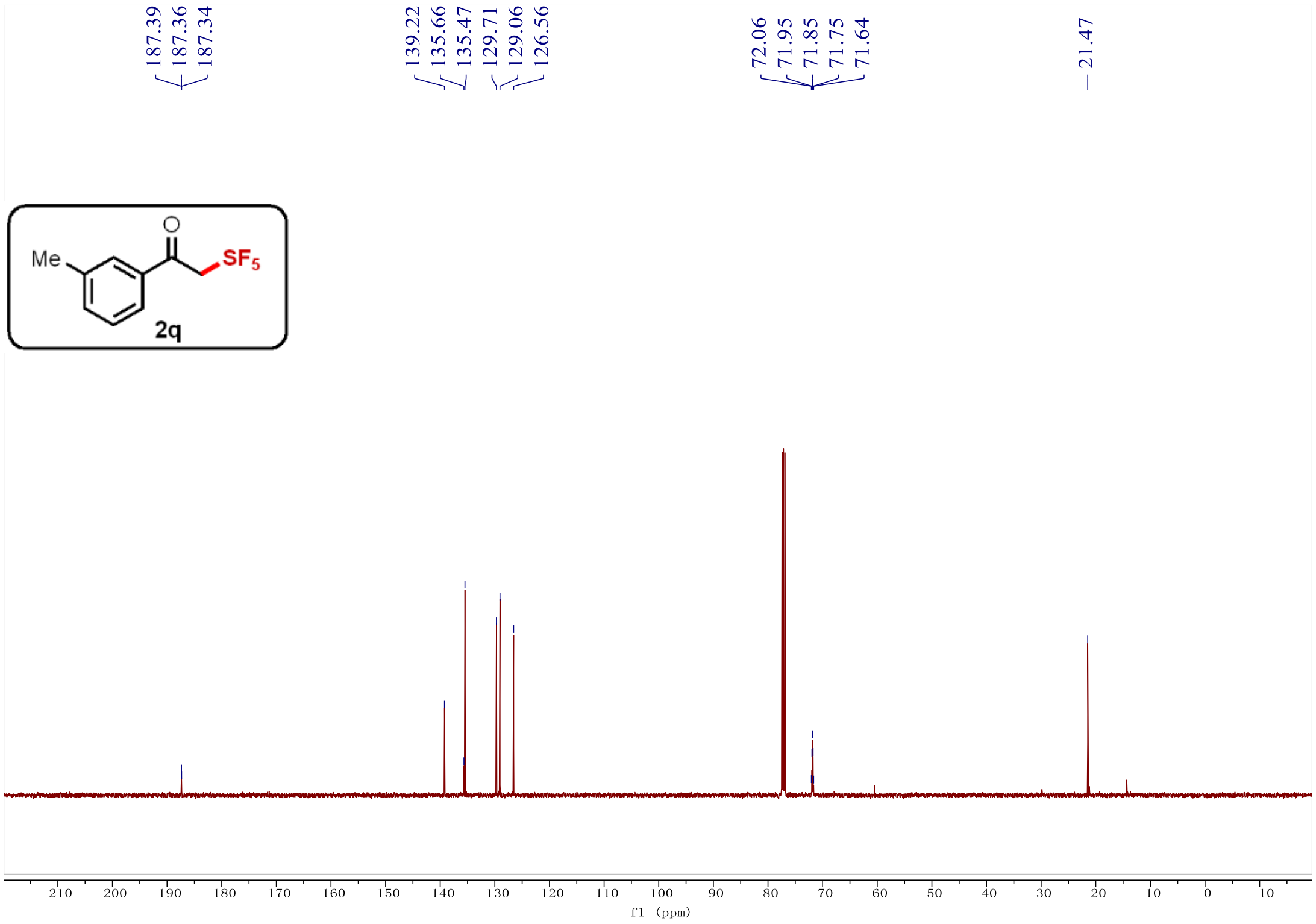

Supplementary Figure 50. ^{13}C NMR Spectrum of Compound 2q (126 MHz, $CDCl_3$, 25 ˚C)

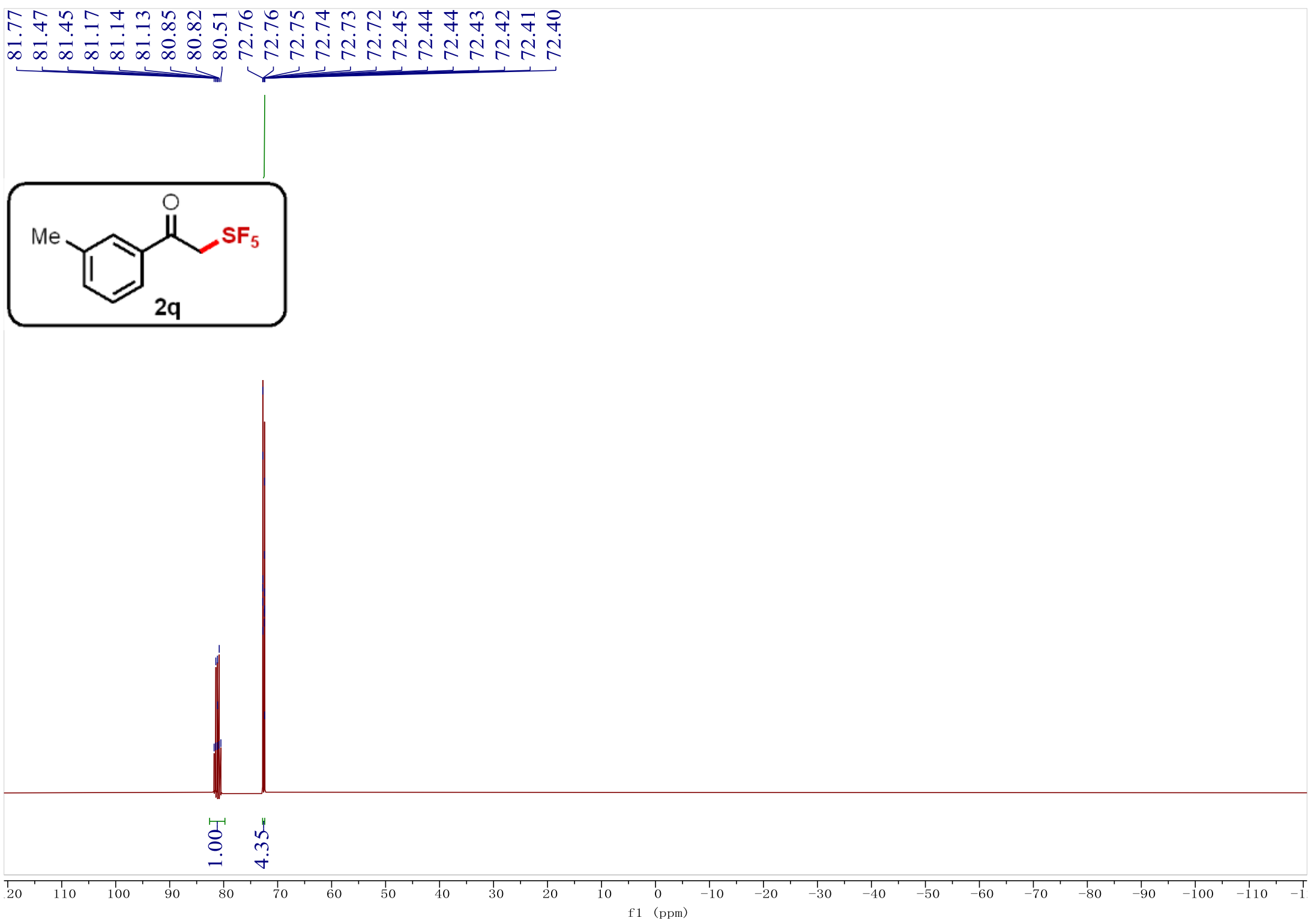

Supplementary Figure 51. ^{19}F **NMR Spectrum of Compound 2q (471 MHz,** $CDCl_3$**, 25 °C)**

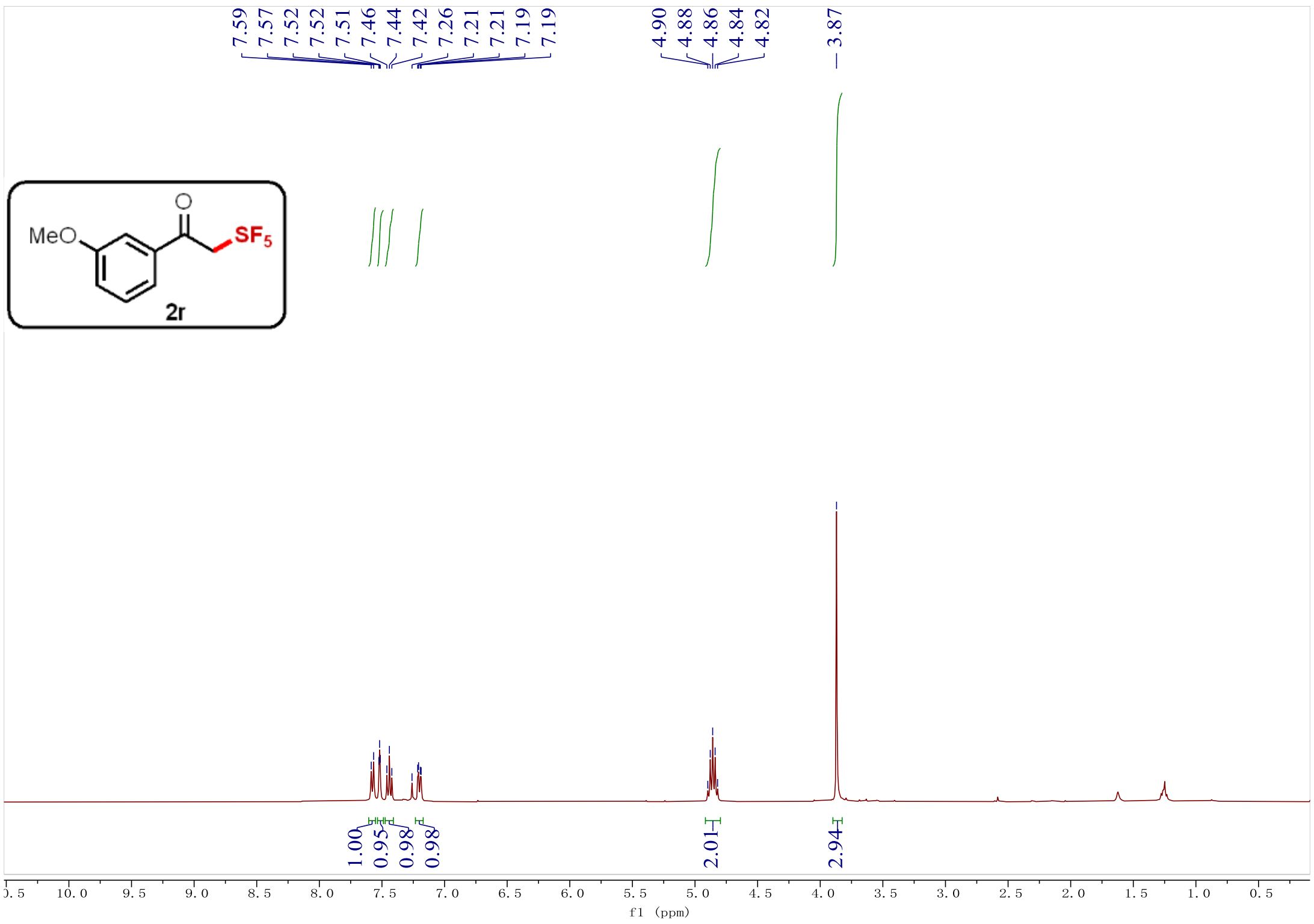

Supplementary Figure 52. ^{1}H **NMR Spectrum of Compound 2r (400 MHz,** $CDCl_3$**, 25 °C)**

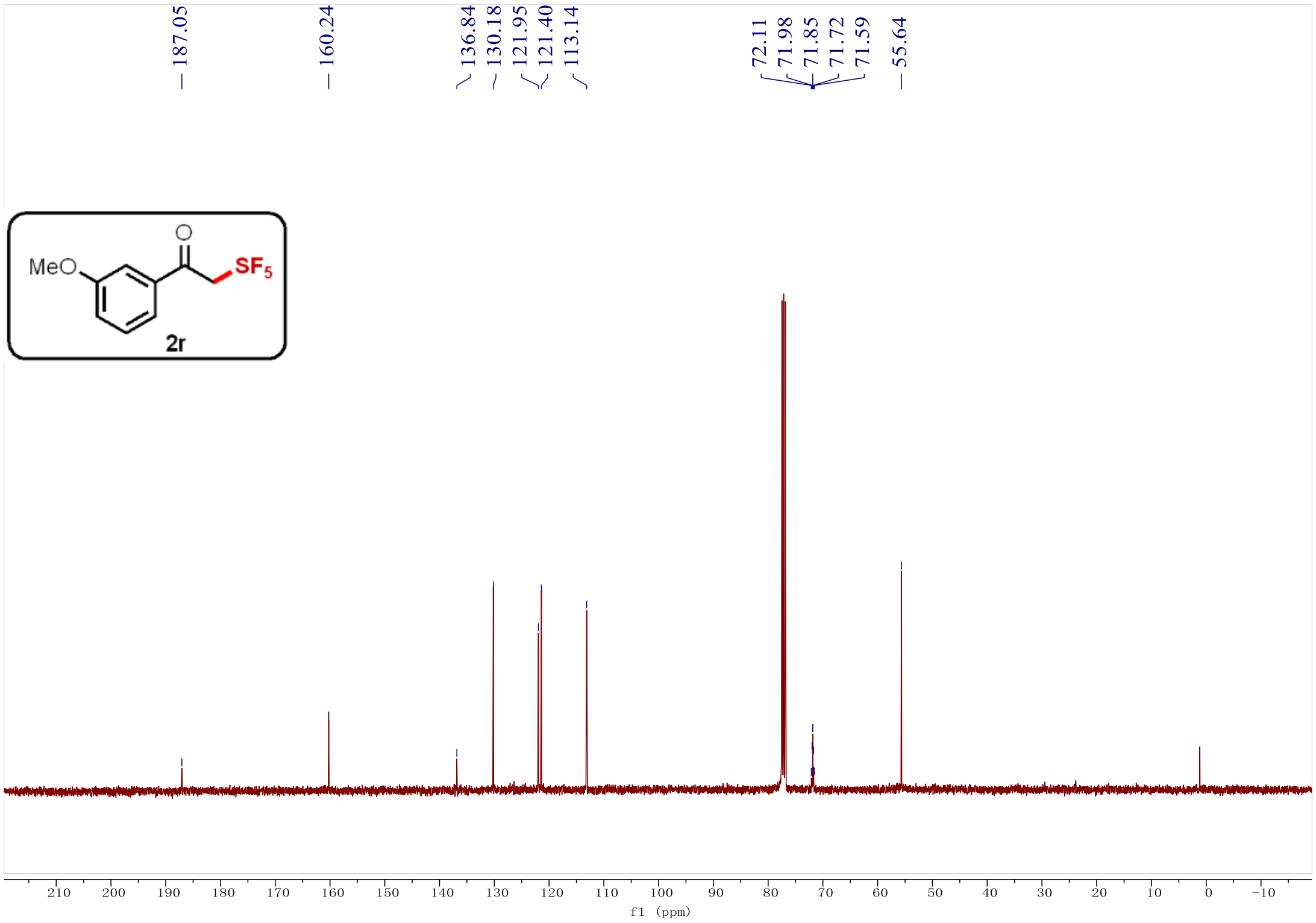

Supplementary Figure 53. ^{13}C NMR Spectrum of Compound 2r (101 MHz, $CDCl_3$, 25 °C)

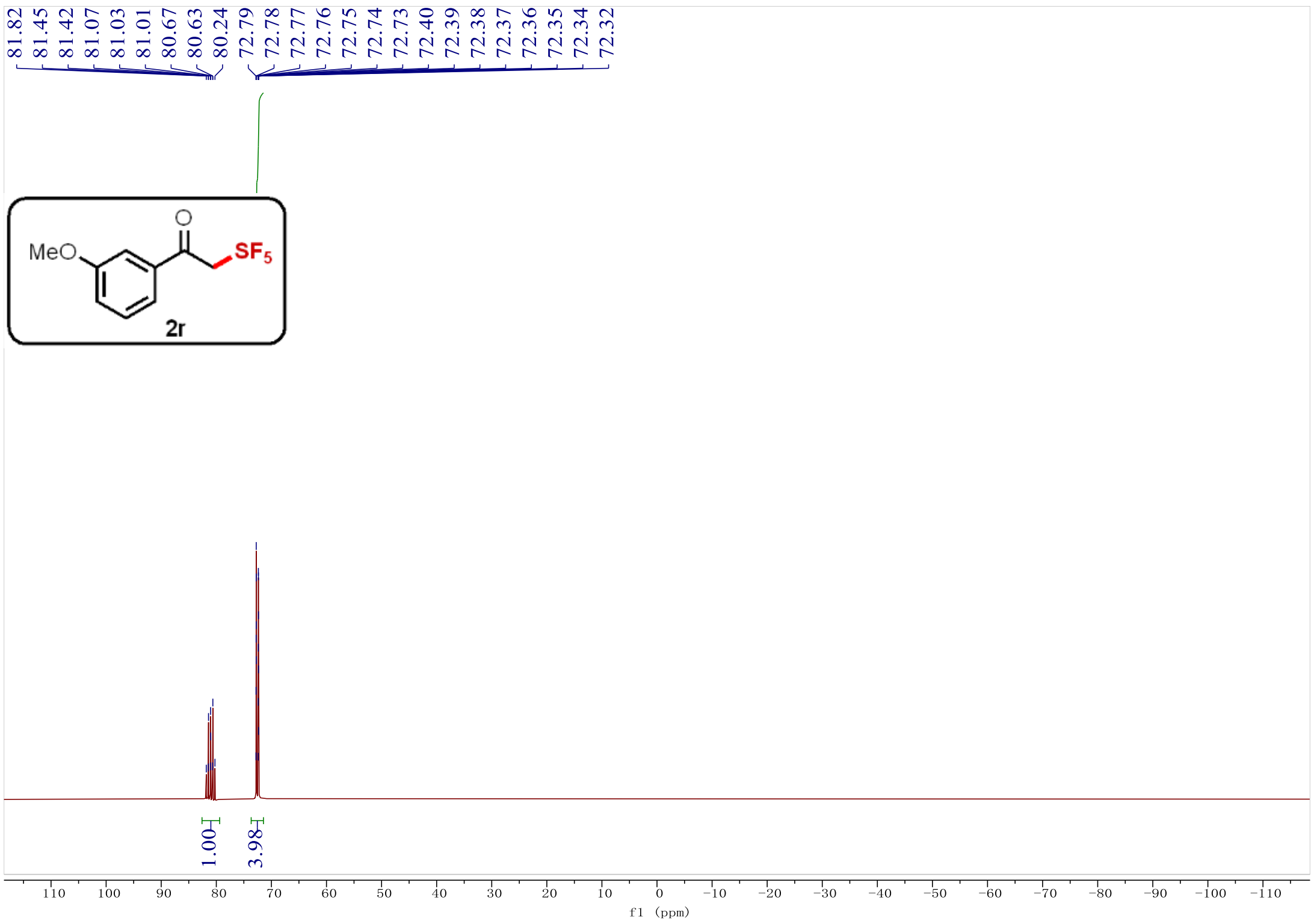

Supplementary Figure 54. ^{19}F NMR Spectrum of Compound 2r (376 MHz, $CDCl_3$, 25 °C)

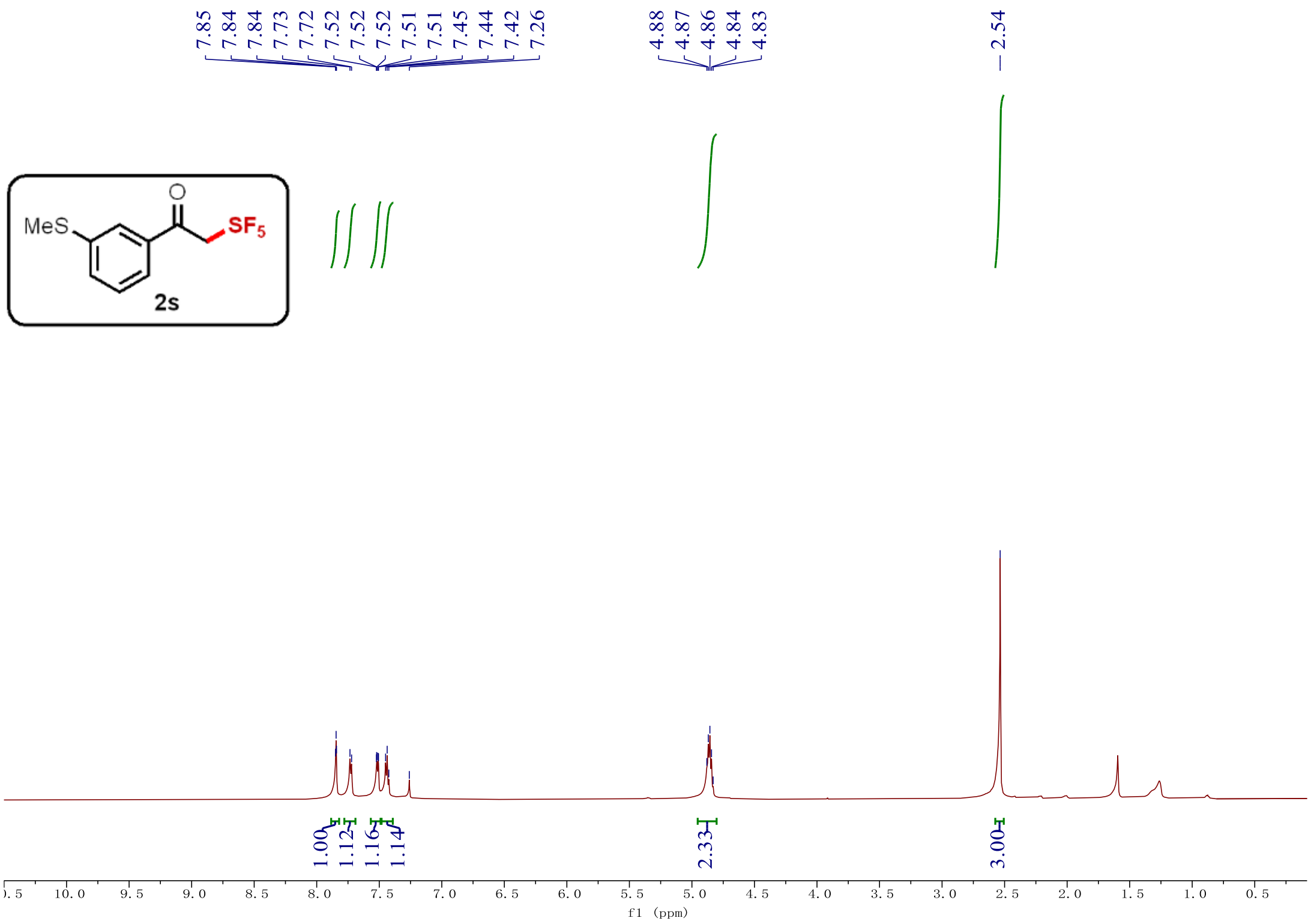

Supplementary Figure 55. ^{1}H NMR Spectrum of Compound 2s (600 MHz, $CDCl_3$, 25 ˚C)

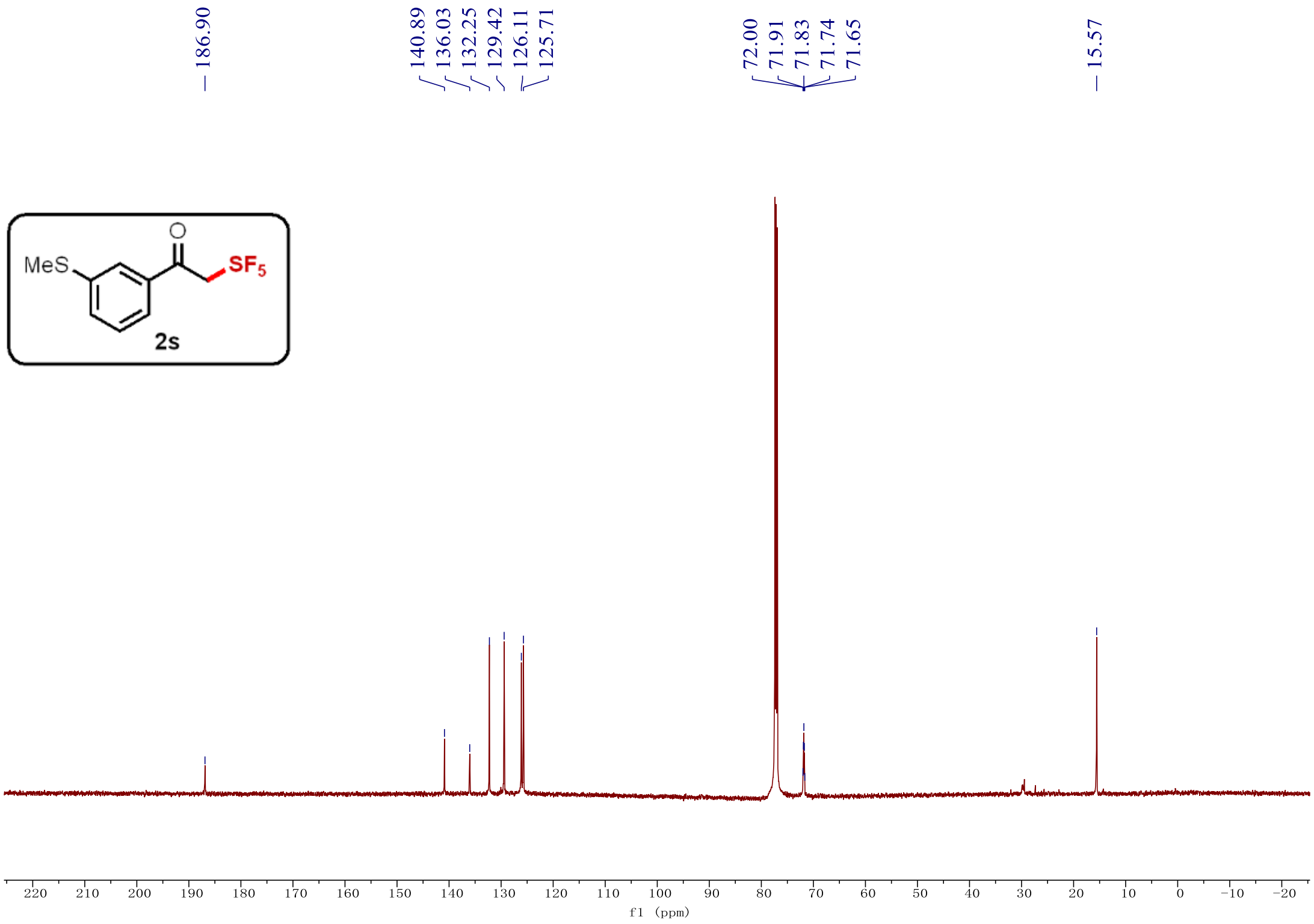

Supplementary Figure 56. ^{13}C NMR Spectrum of Compound 2s (151 MHz, $CDCl_3$, 25 ˚C)

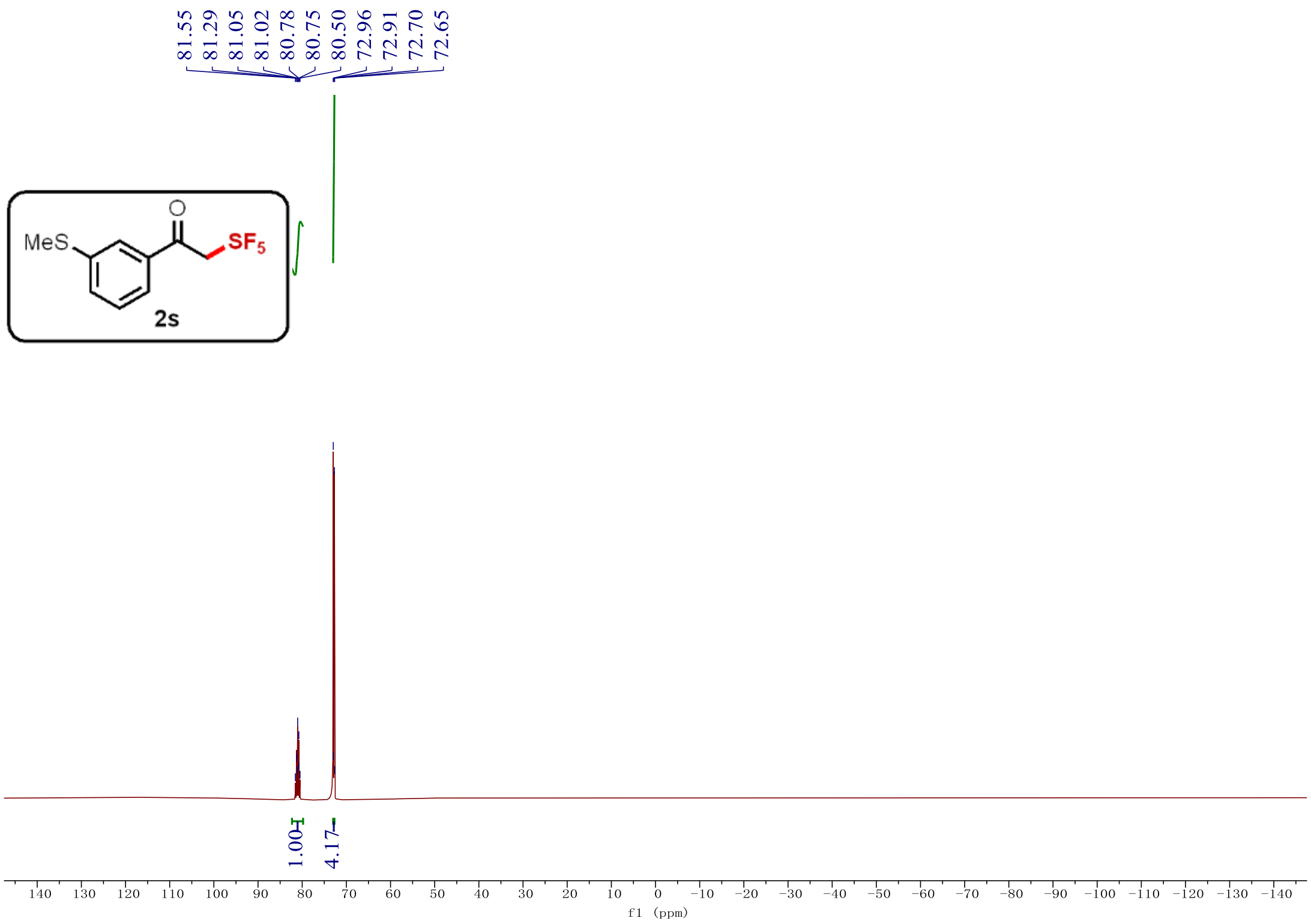

Supplementary Figure 57. ^{19}F NMR Spectrum of Compound 2s (565 MHz, $CDCl_3$, 25 °C)

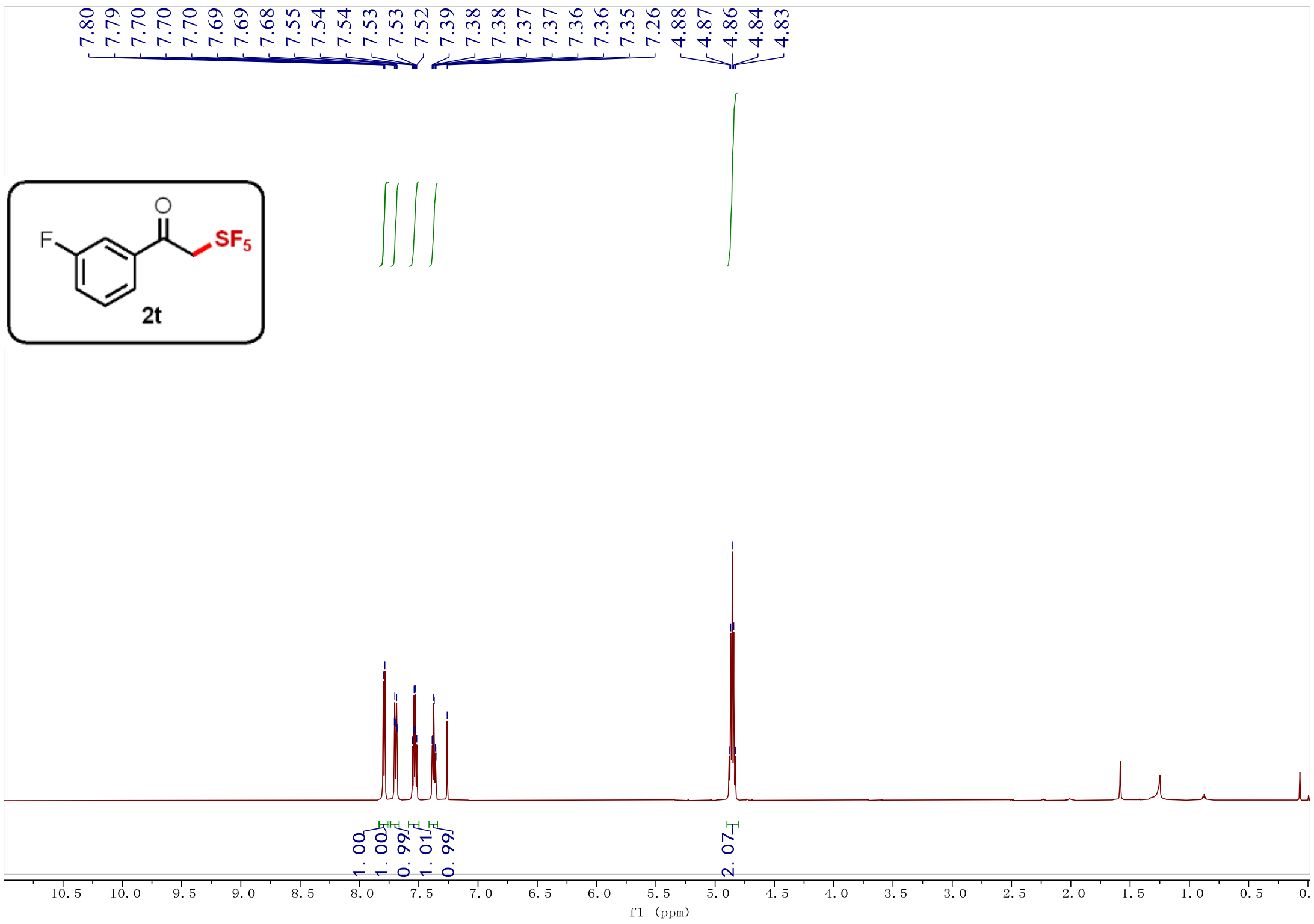

Supplementary Figure 58. ^{1}H NMR Spectrum of Compound 2t (600 MHz, $CDCl_3$, 25 °C)

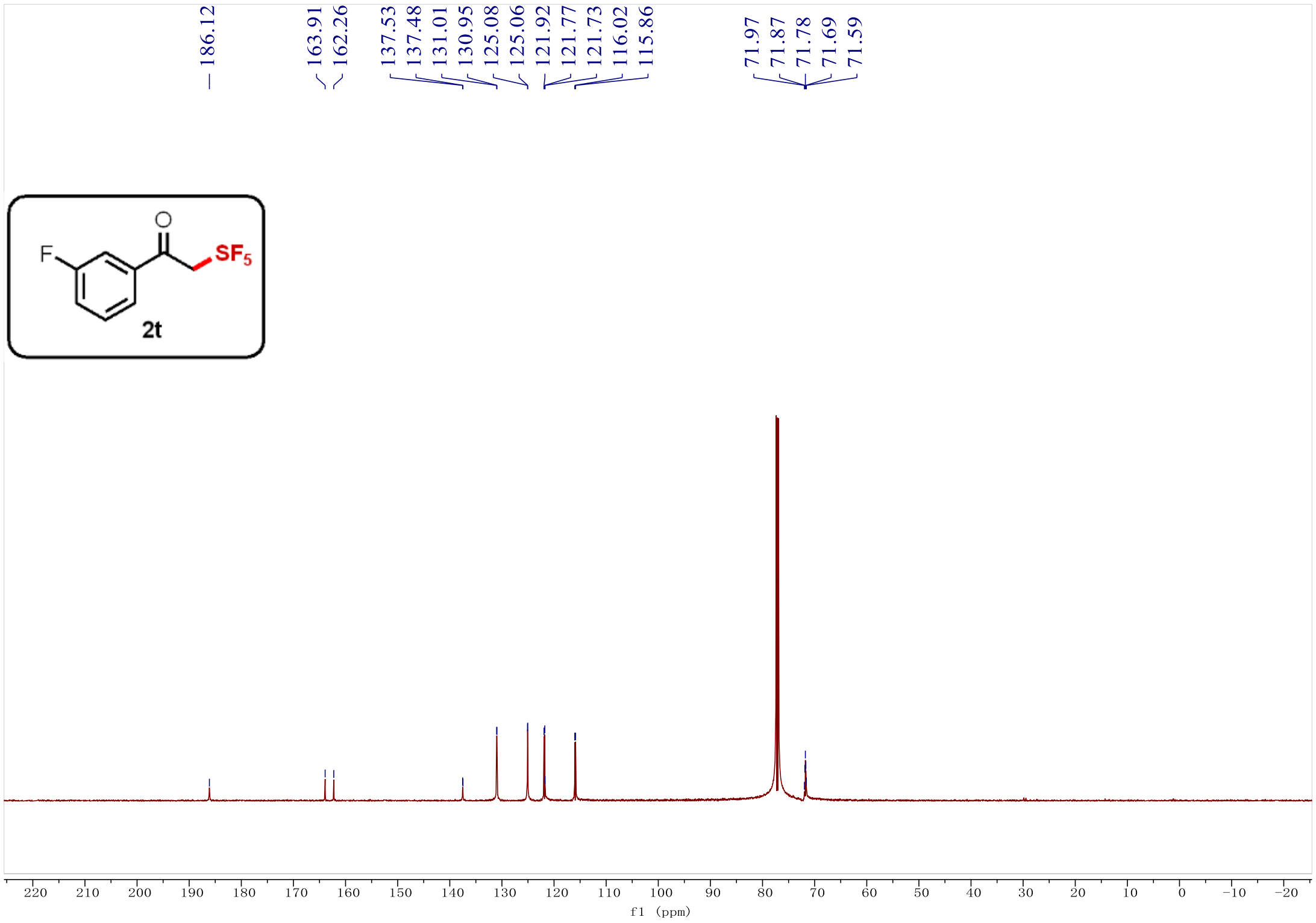

Supplementary Figure 59. ^{13}**C NMR Spectrum of Compound 2t (151 MHz, $CDCl_3$, 25 °C)**

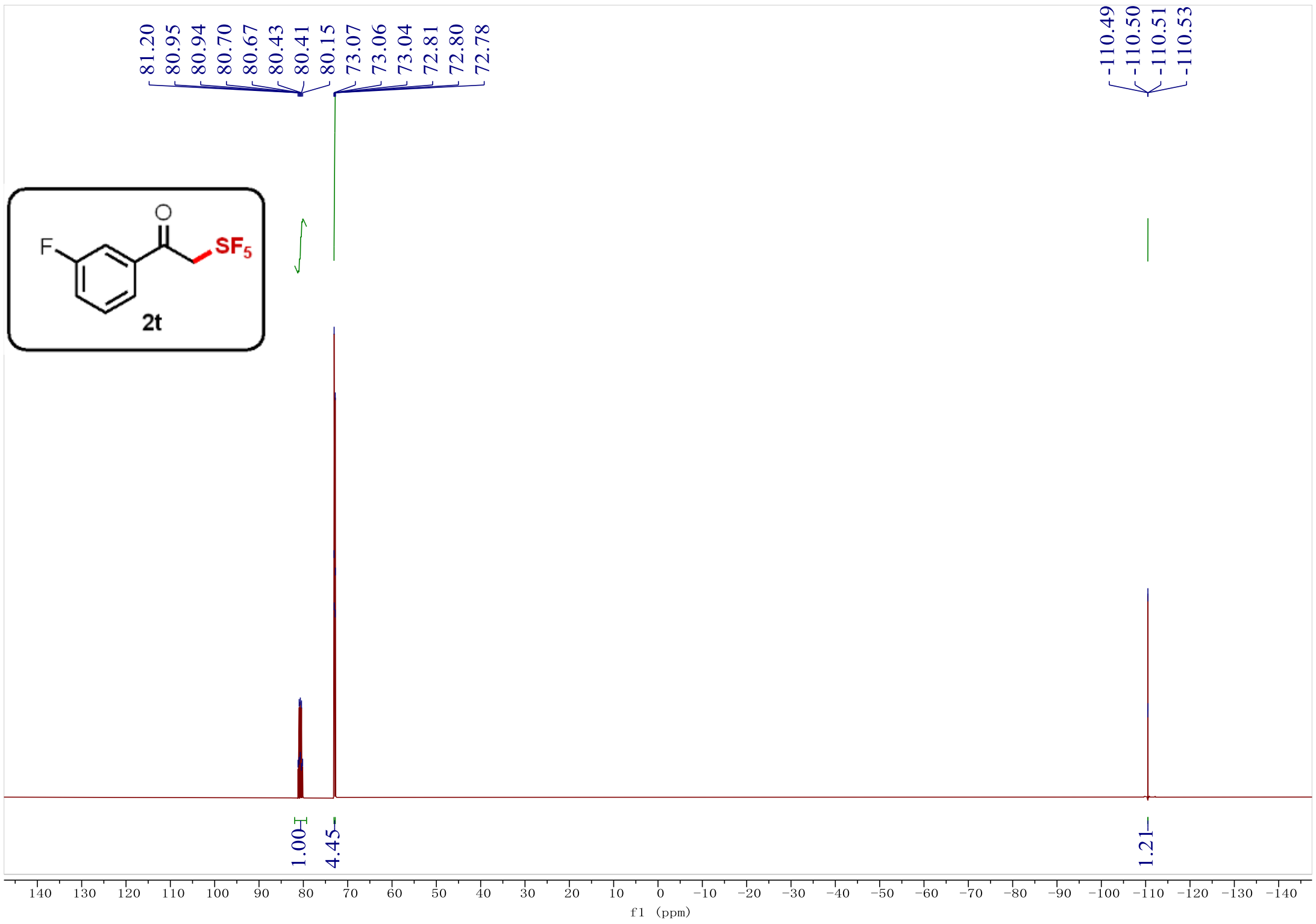

Supplementary Figure 60. ^{19}**F NMR Spectrum of Compound 2t (565 MHz, $CDCl_3$, 25 °C)**

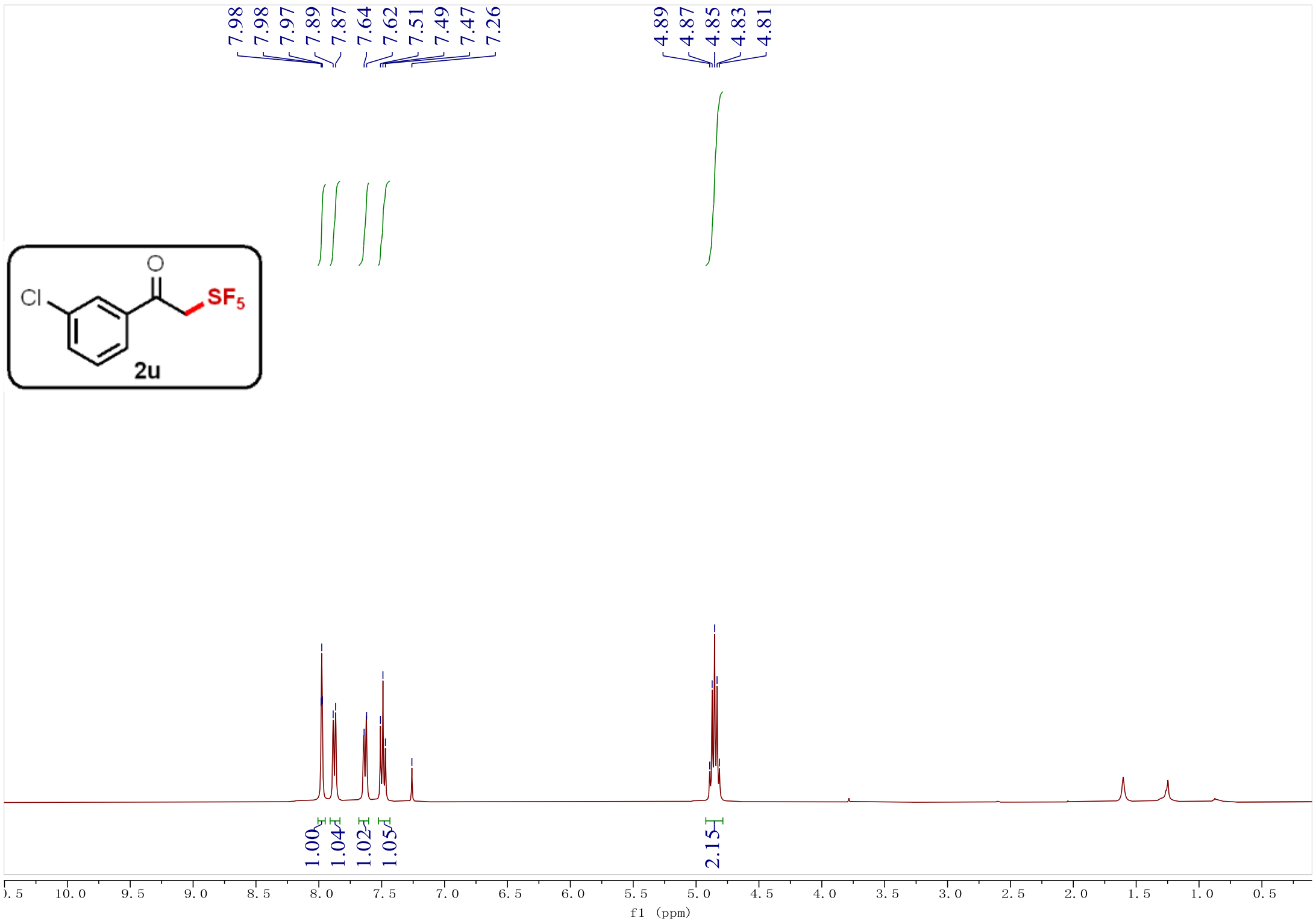

Supplementary Figure 61. ^{1}H NMR Spectrum of Compound 2u (400 MHz, $CDCl_3$, 25 ˚C)

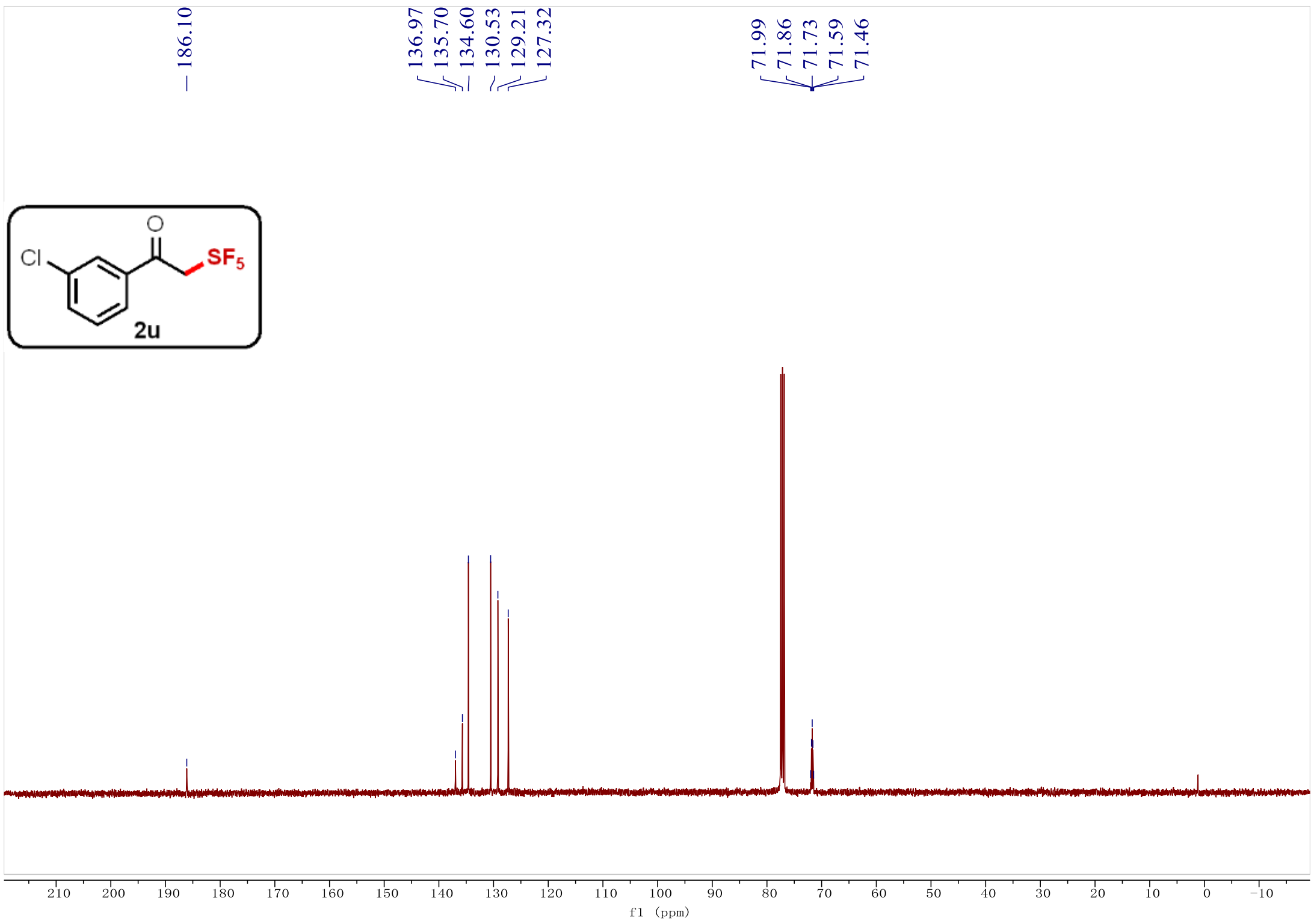

Supplementary Figure 62. ^{13}C NMR Spectrum of Compound 2u (101 MHz, $CDCl_3$, 25 ˚C)

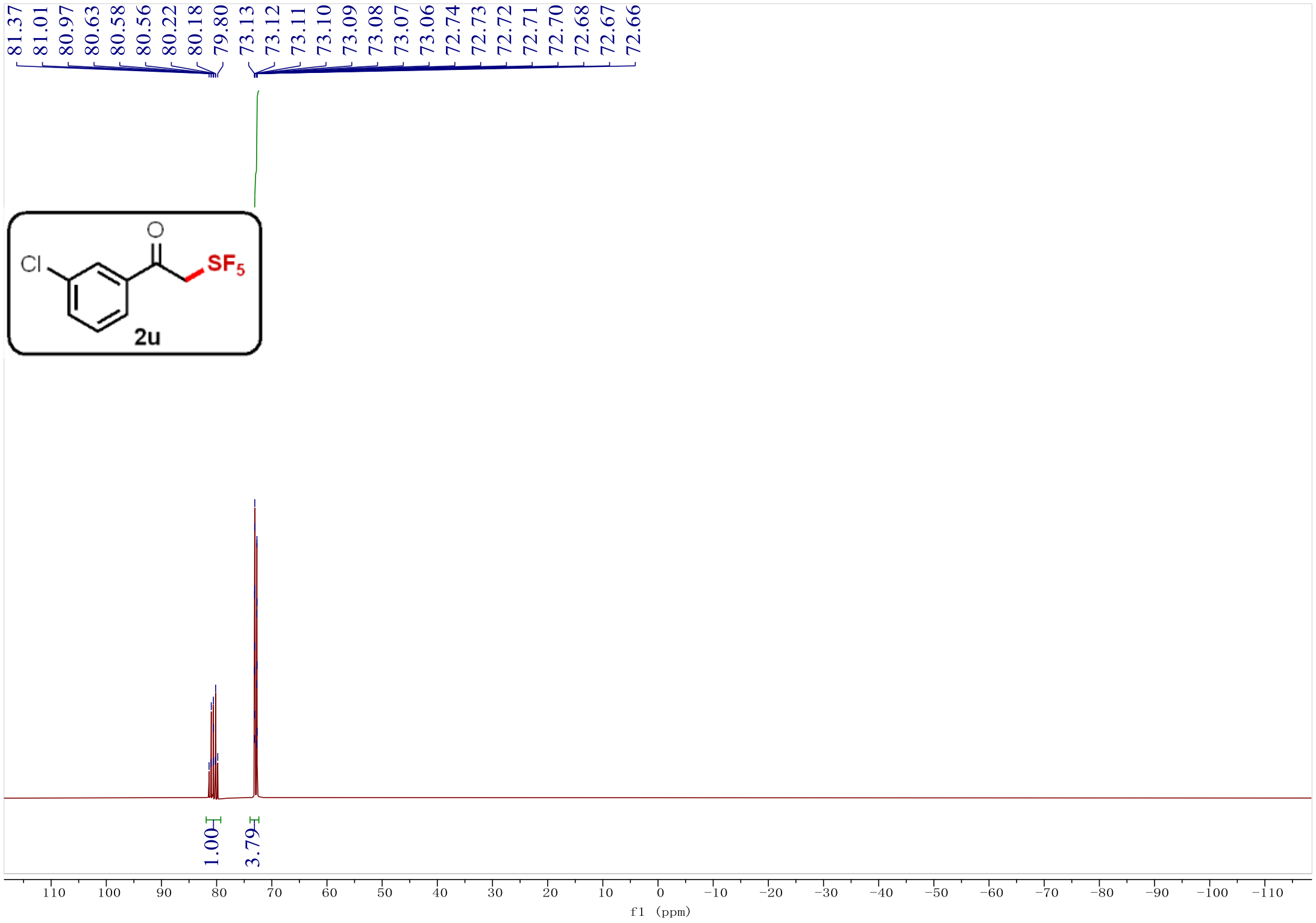

Supplementary Figure 63. ^{19}F NMR Spectrum of Compound 2u (376 MHz, $CDCl_3$, 25 ˚C)

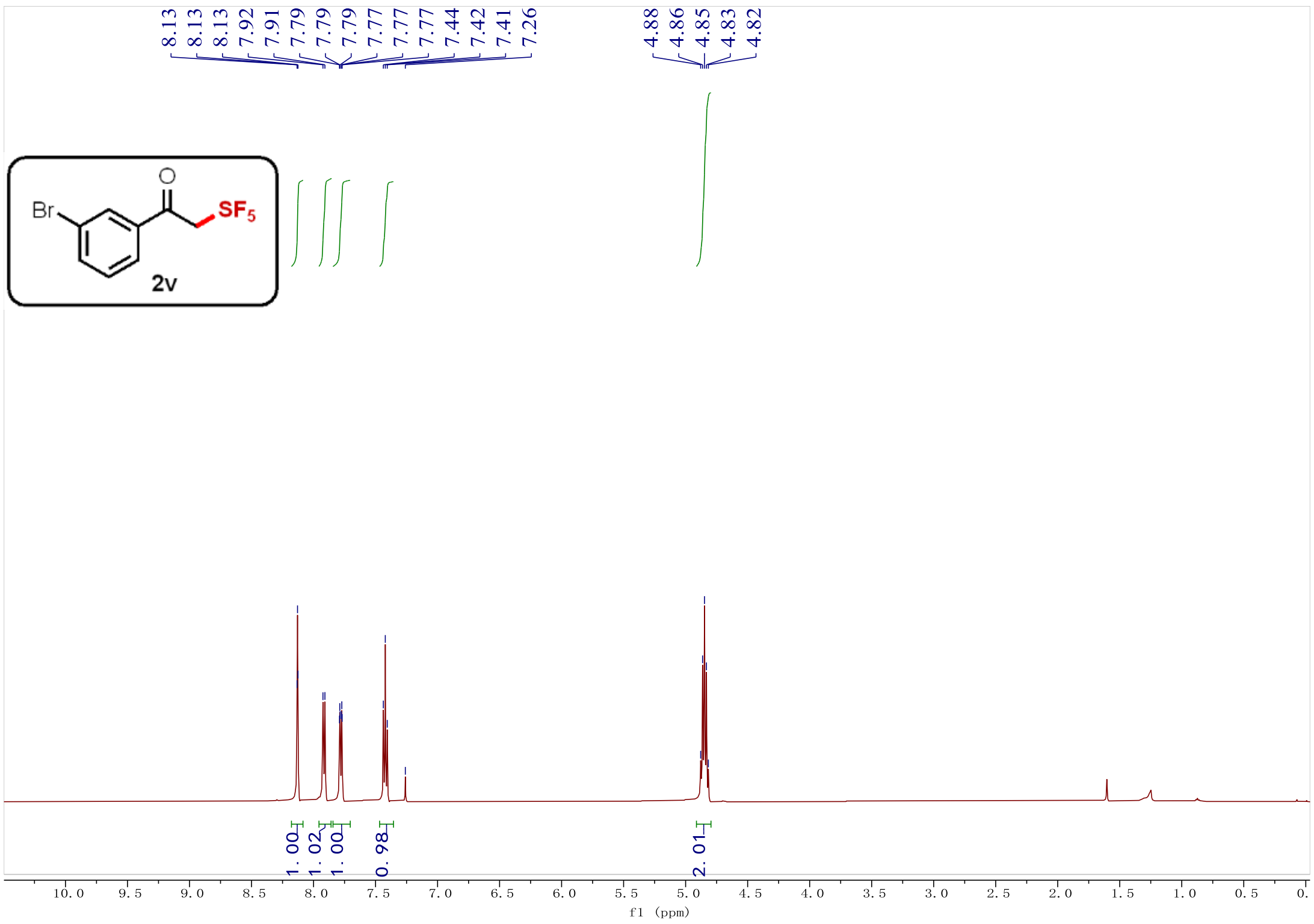

Supplementary Figure 64. ^{1}H NMR Spectrum of Compound 2v (500 MHz, $CDCl_3$, 25 ˚C)

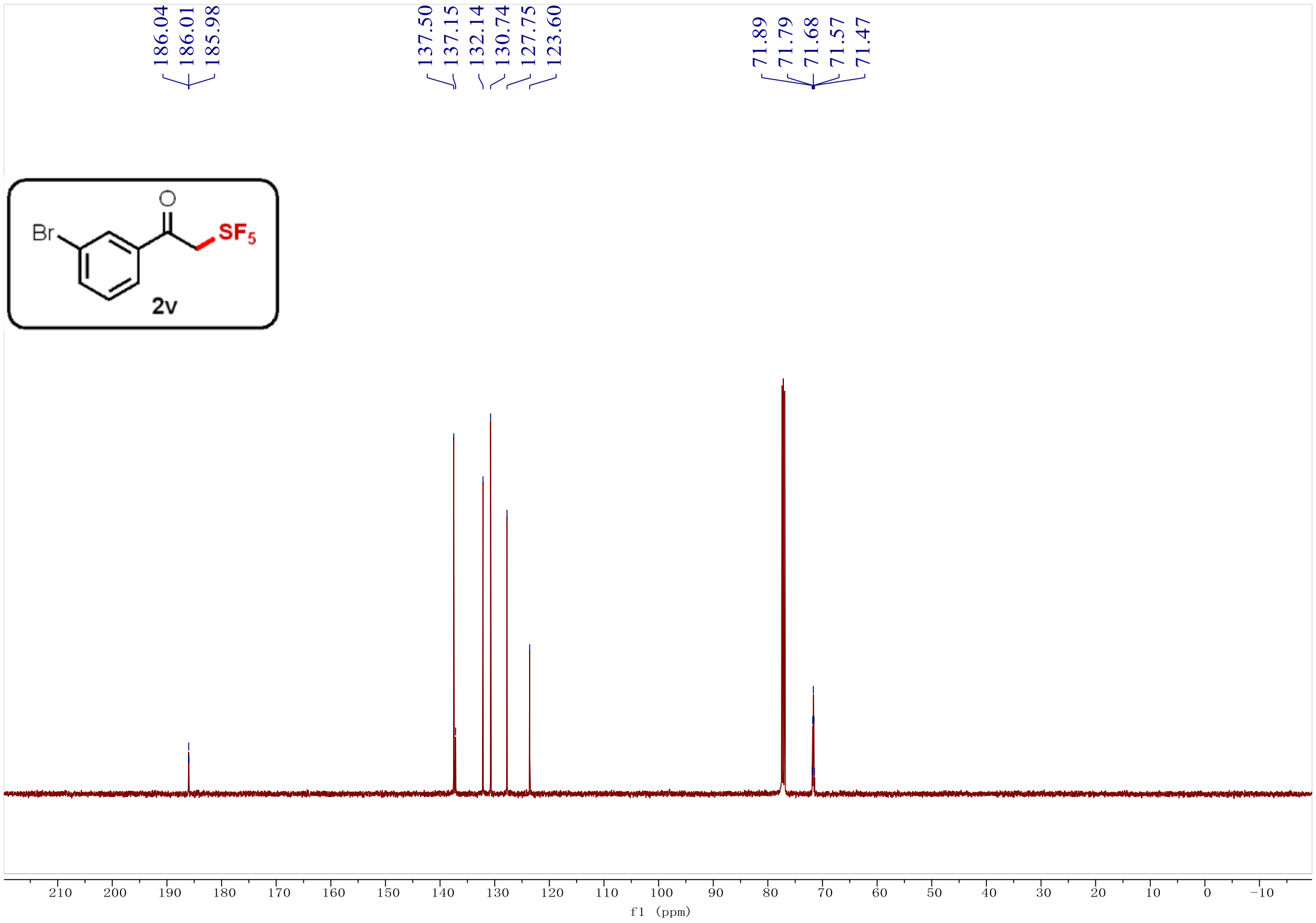

Supplementary Figure 65. ^{13}C NMR Spectrum of Compound 2v (126 MHz, $CDCl_3$, 25 °C)

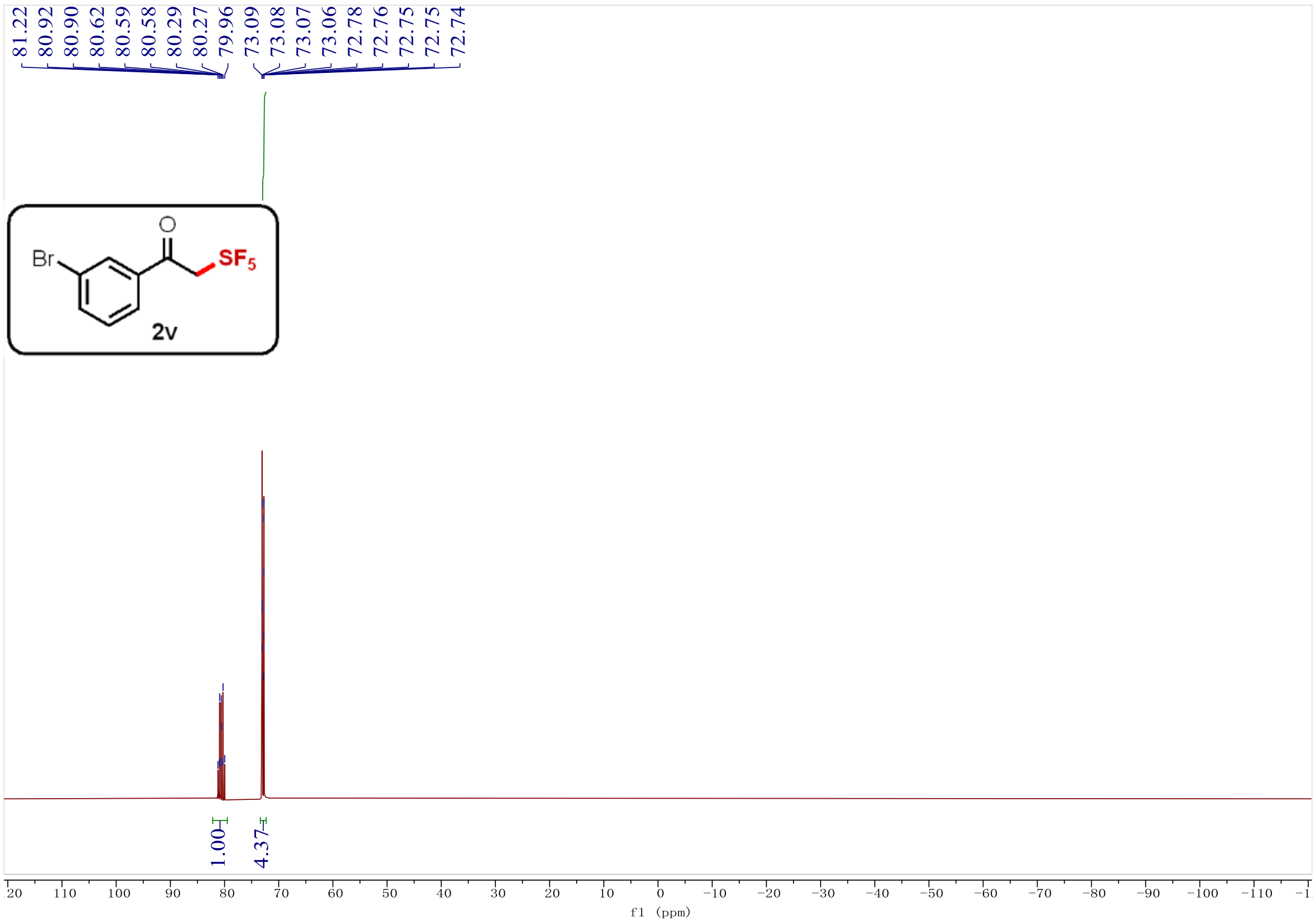

Supplementary Figure 66. ^{19}F NMR Spectrum of Compound 2v (471 MHz, $CDCl_3$, 25 °C)

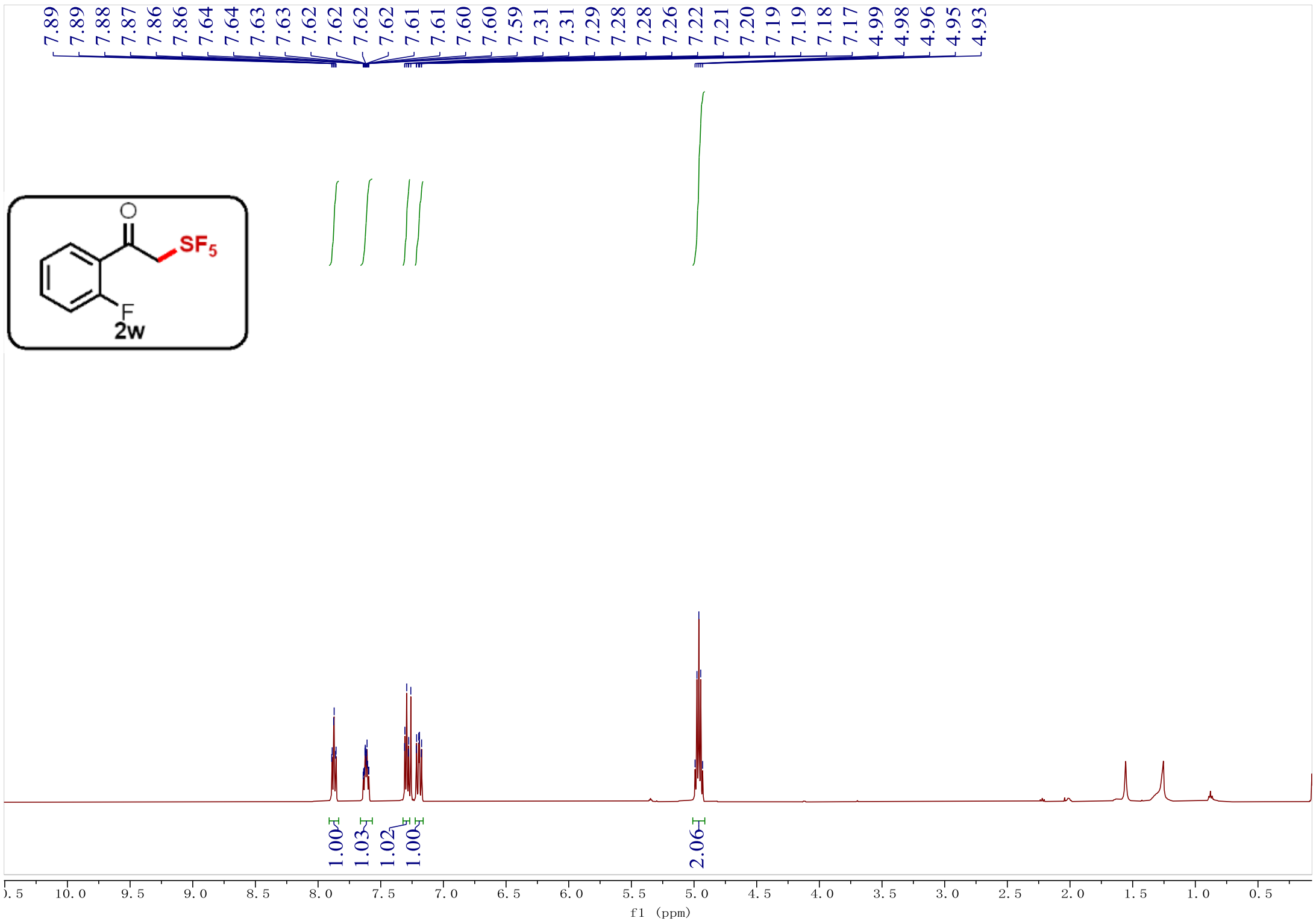

Supplementary Figure 67. **^{1}H NMR Spectrum of Compound 2v (500 MHz, $CDCl_3$, 25 ˚C)**

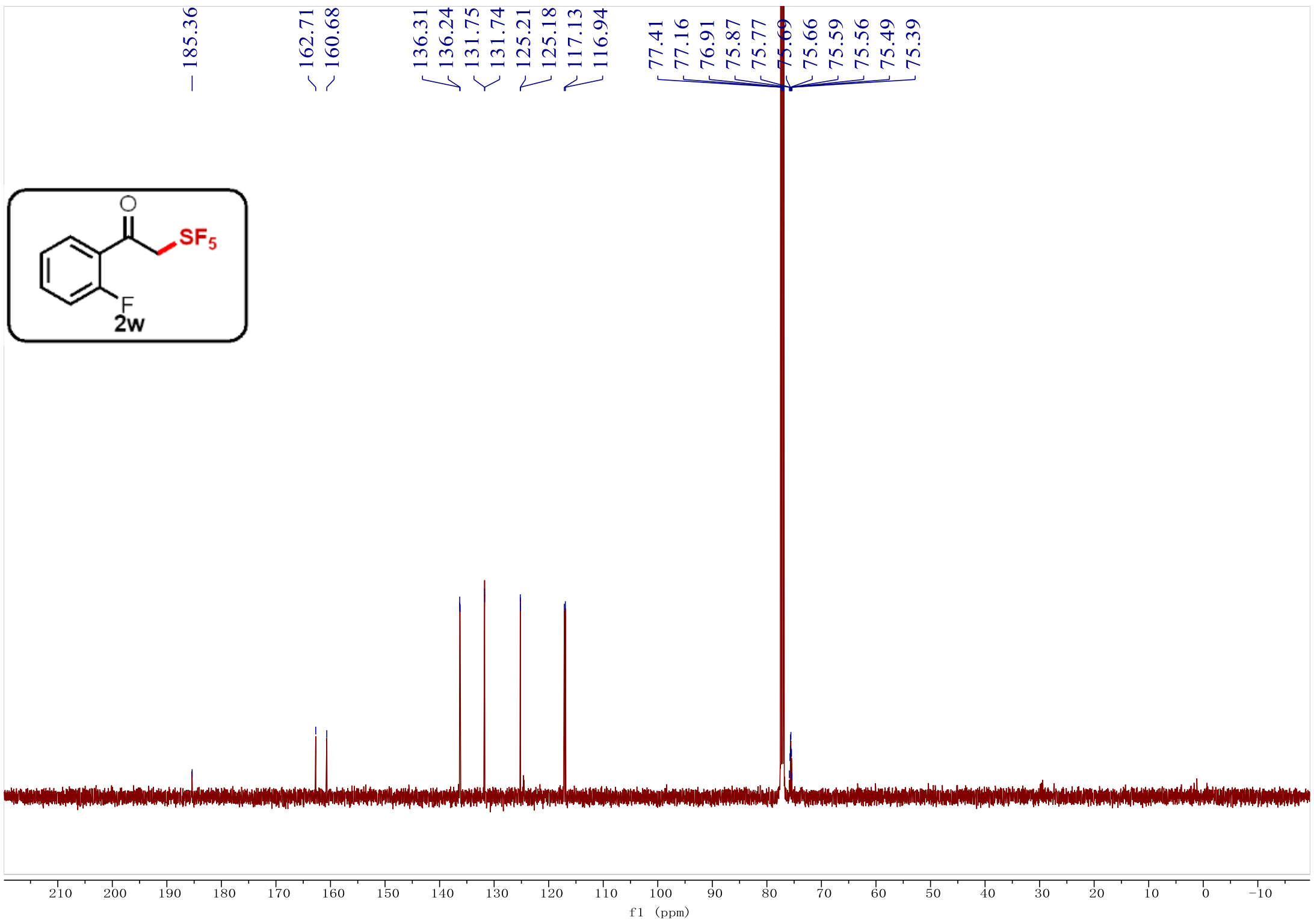

Supplementary Figure 68. **^{13}C NMR Spectrum of Compound 2v (126 MHz, $CDCl_3$, 25 ˚C)**

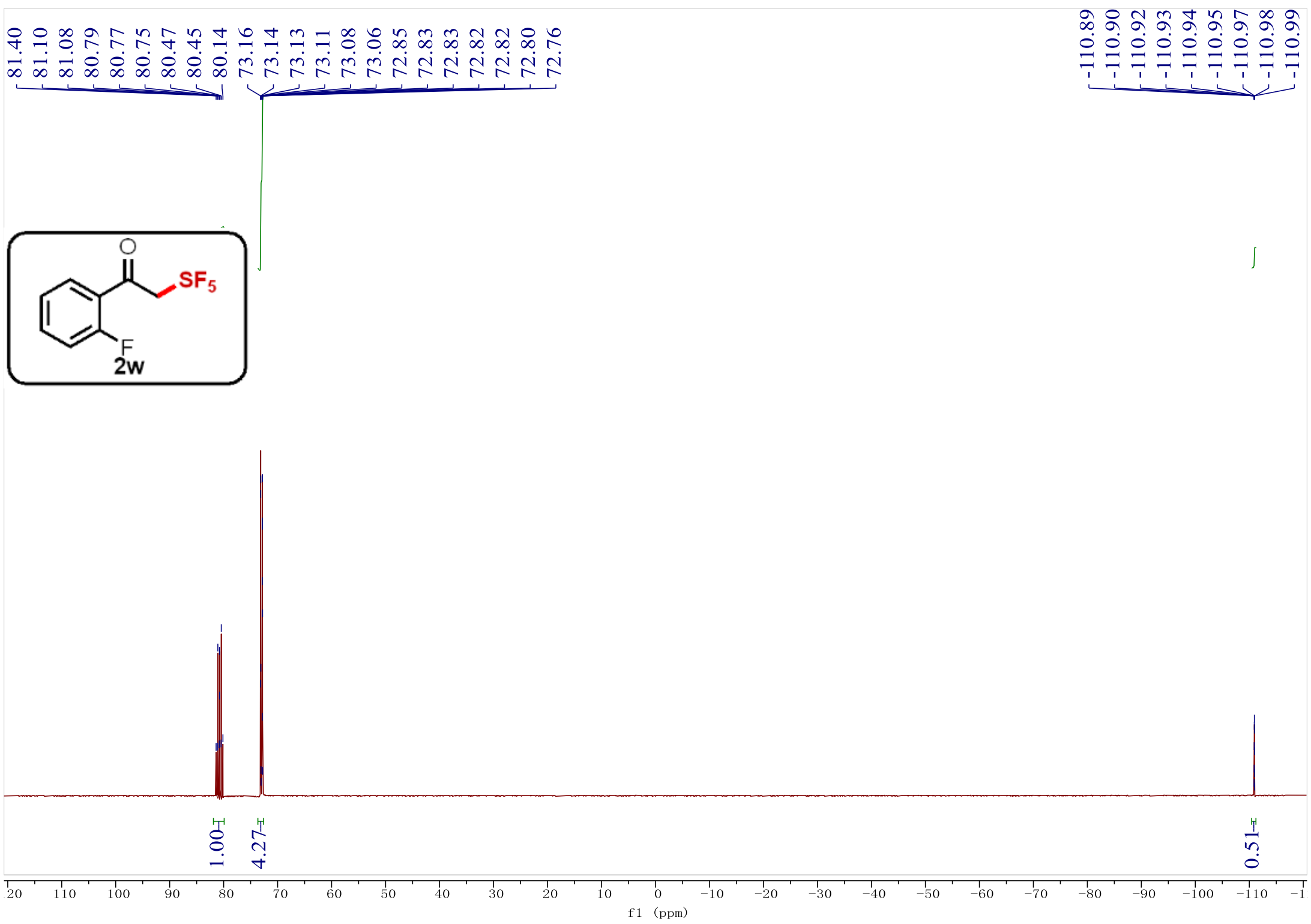

Supplementary Figure 69. ^{19}F NMR Spectrum of Compound 2v (471 MHz, $CDCl_3$, 25 ˚C)

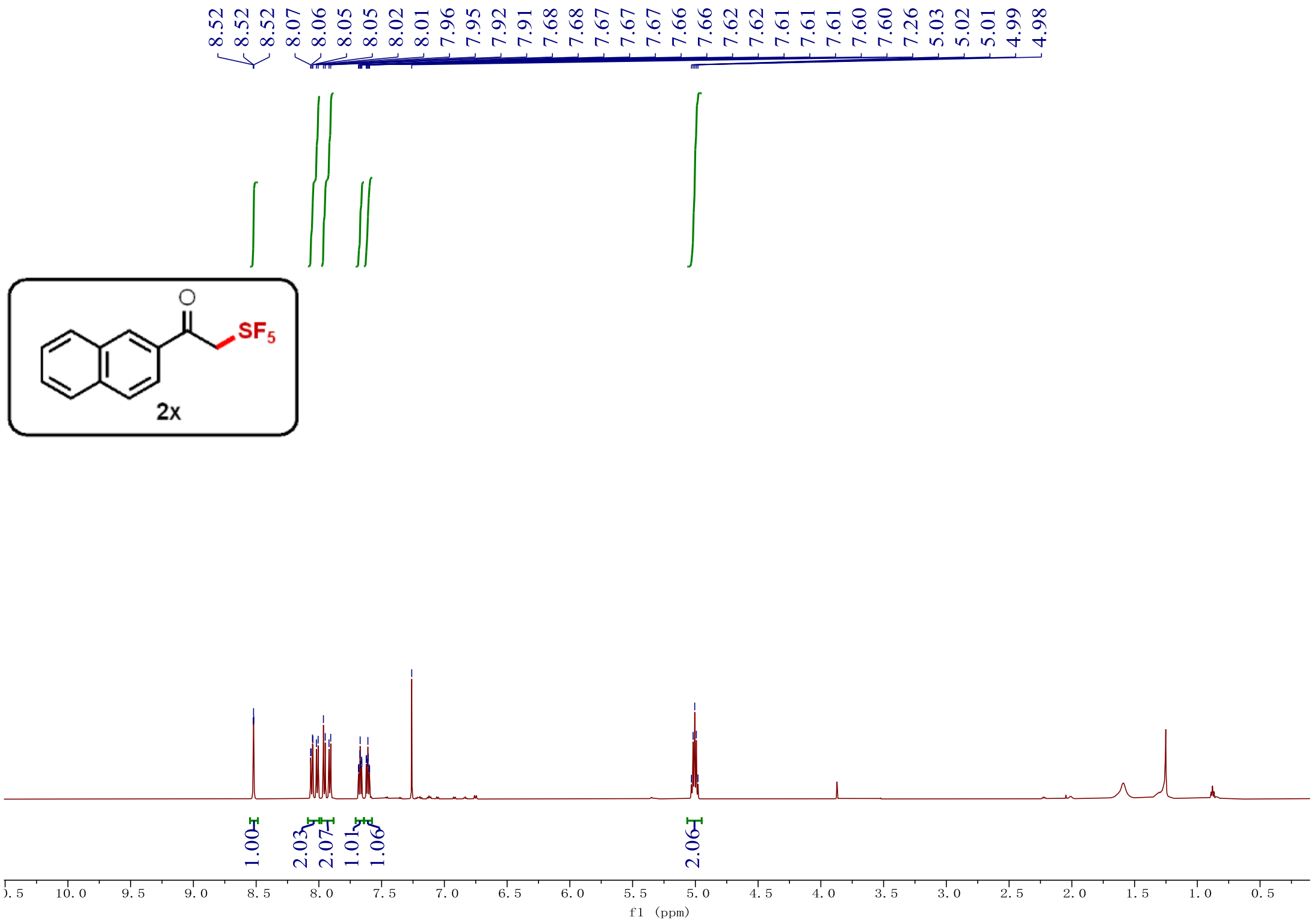

Supplementary Figure 70. ^{1}H NMR Spectrum of Compound 2x (600 MHz, $CDCl_3$, 25 ˚C)

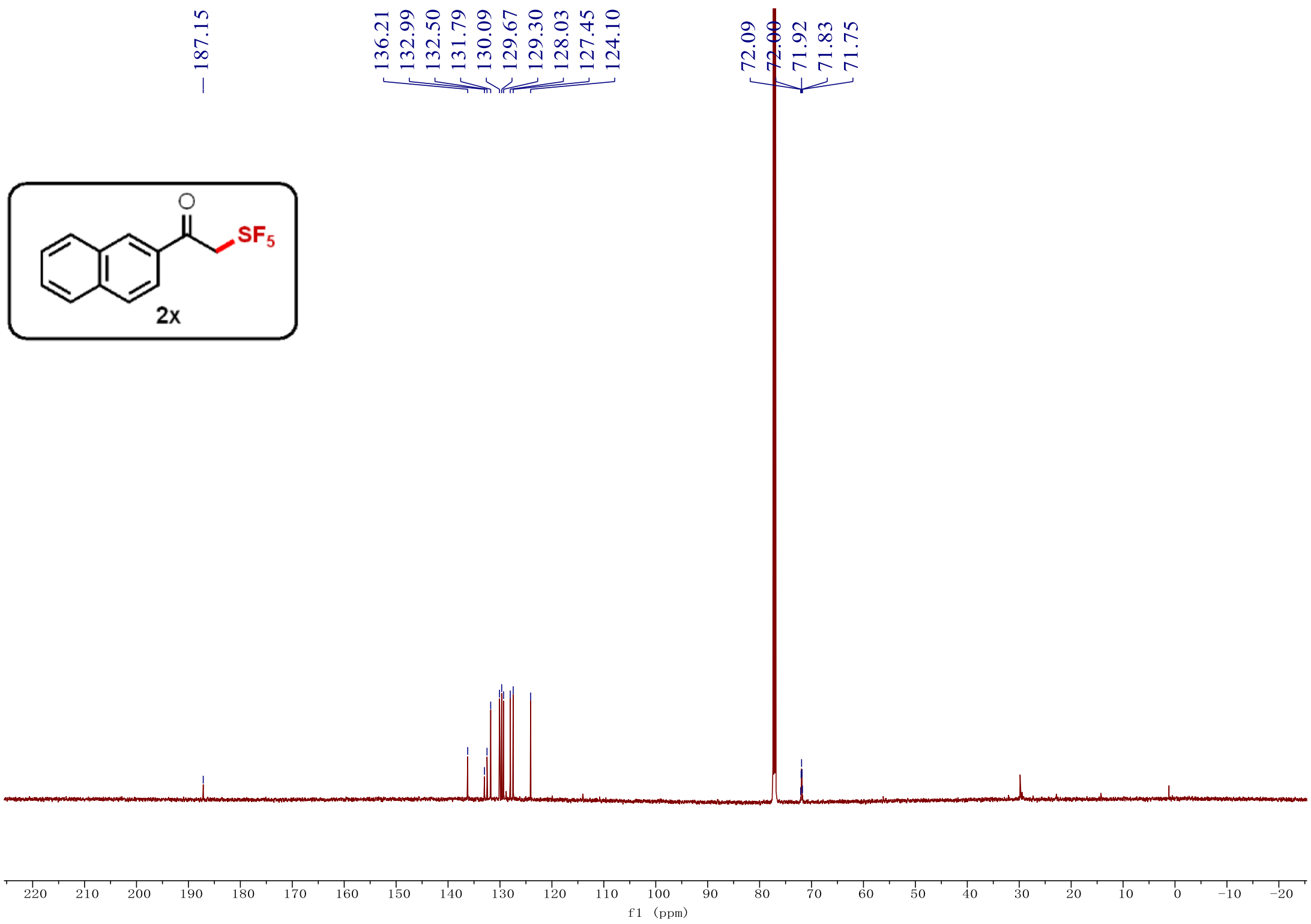

Supplementary Figure 71. ^{13}C NMR Spectrum of Compound 2x (151 MHz, $CDCl_3$, 25 ˚C)

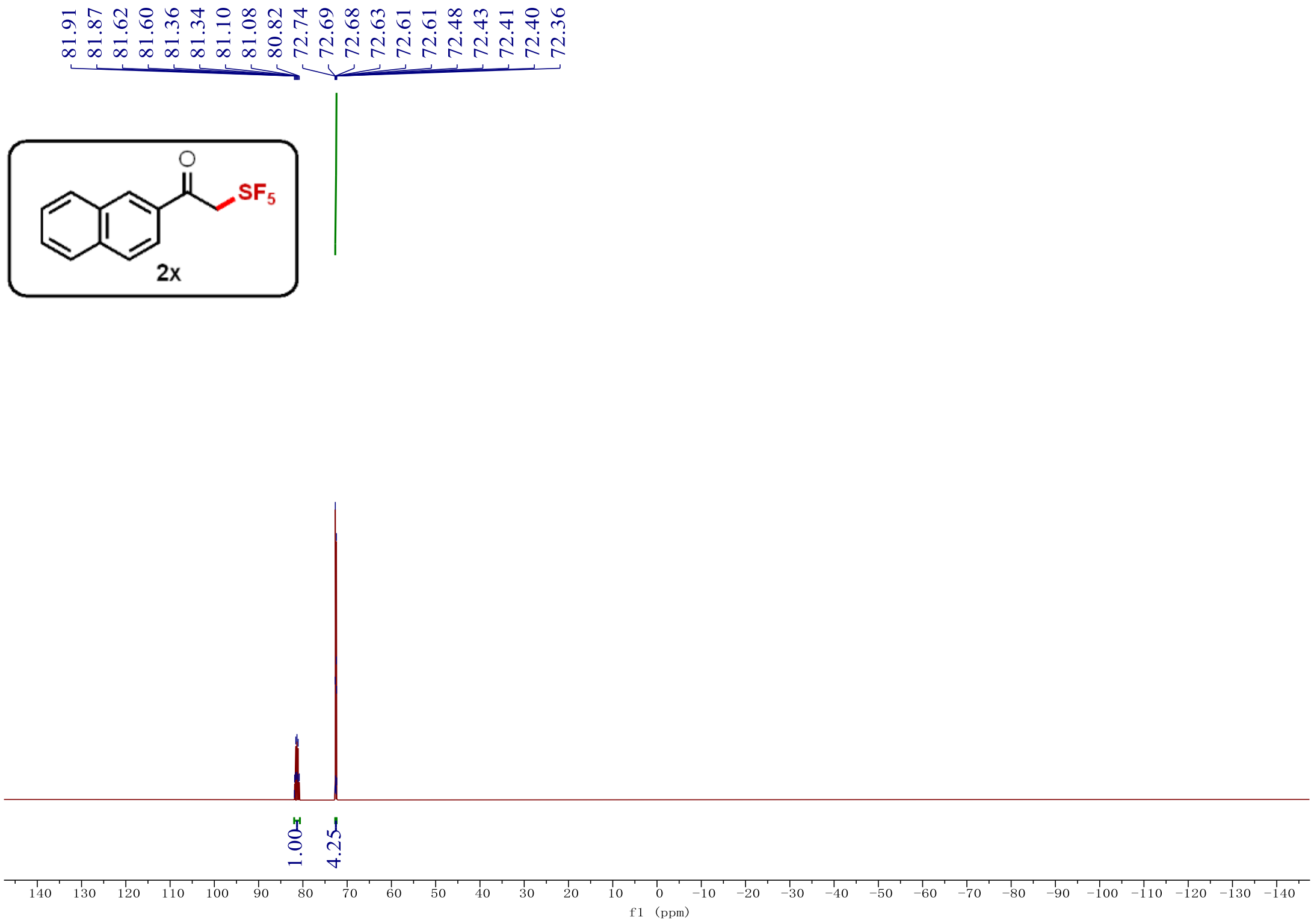

Supplementary Figure 72. ^{19}F NMR Spectrum of Compound 2x (565 MHz, $CDCl_3$, 25 ˚C)

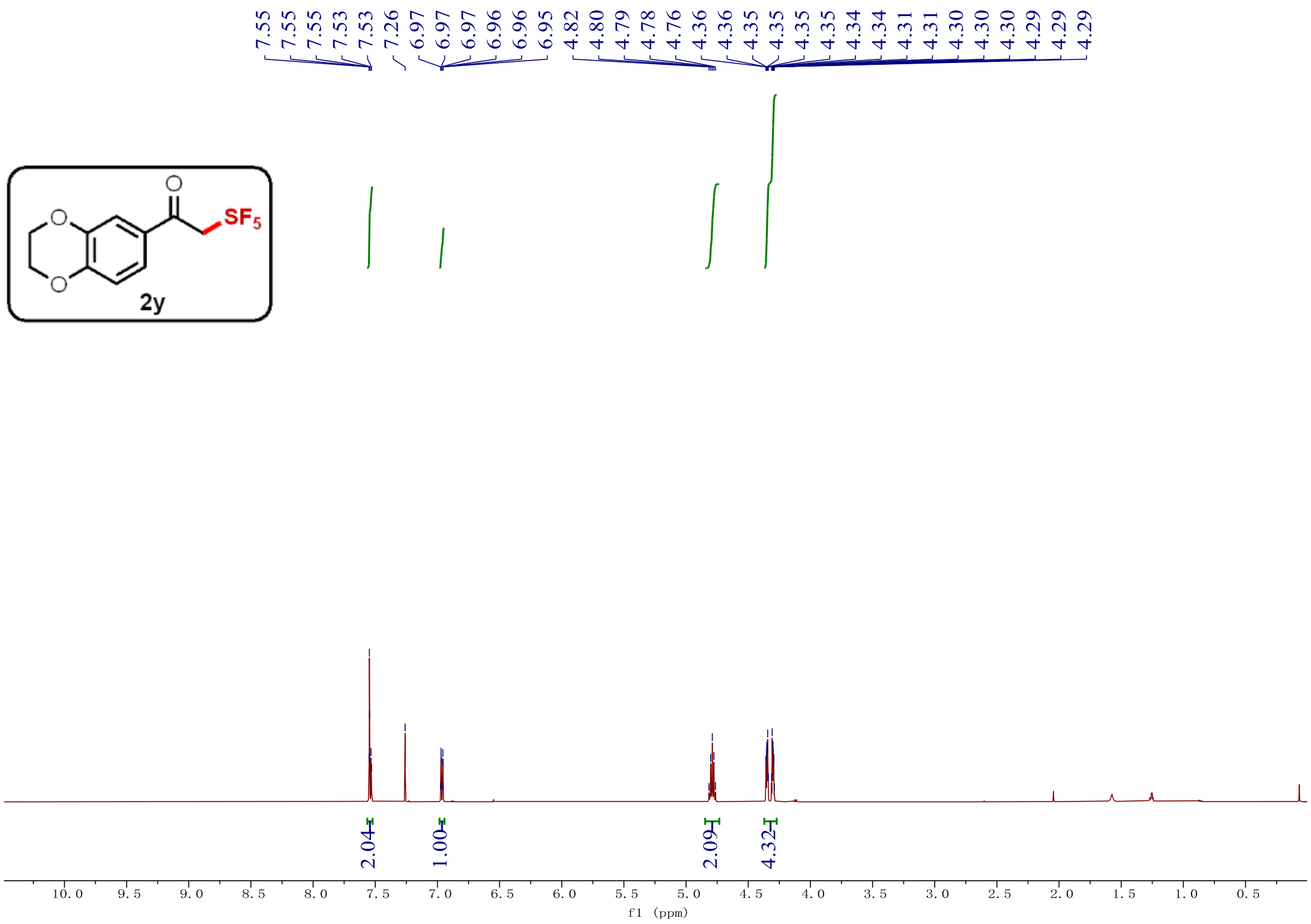

Supplementary Figure 73. ^{1}H NMR Spectrum of Compound 2y (600 MHz, $CDCl_3$, 25 °C)

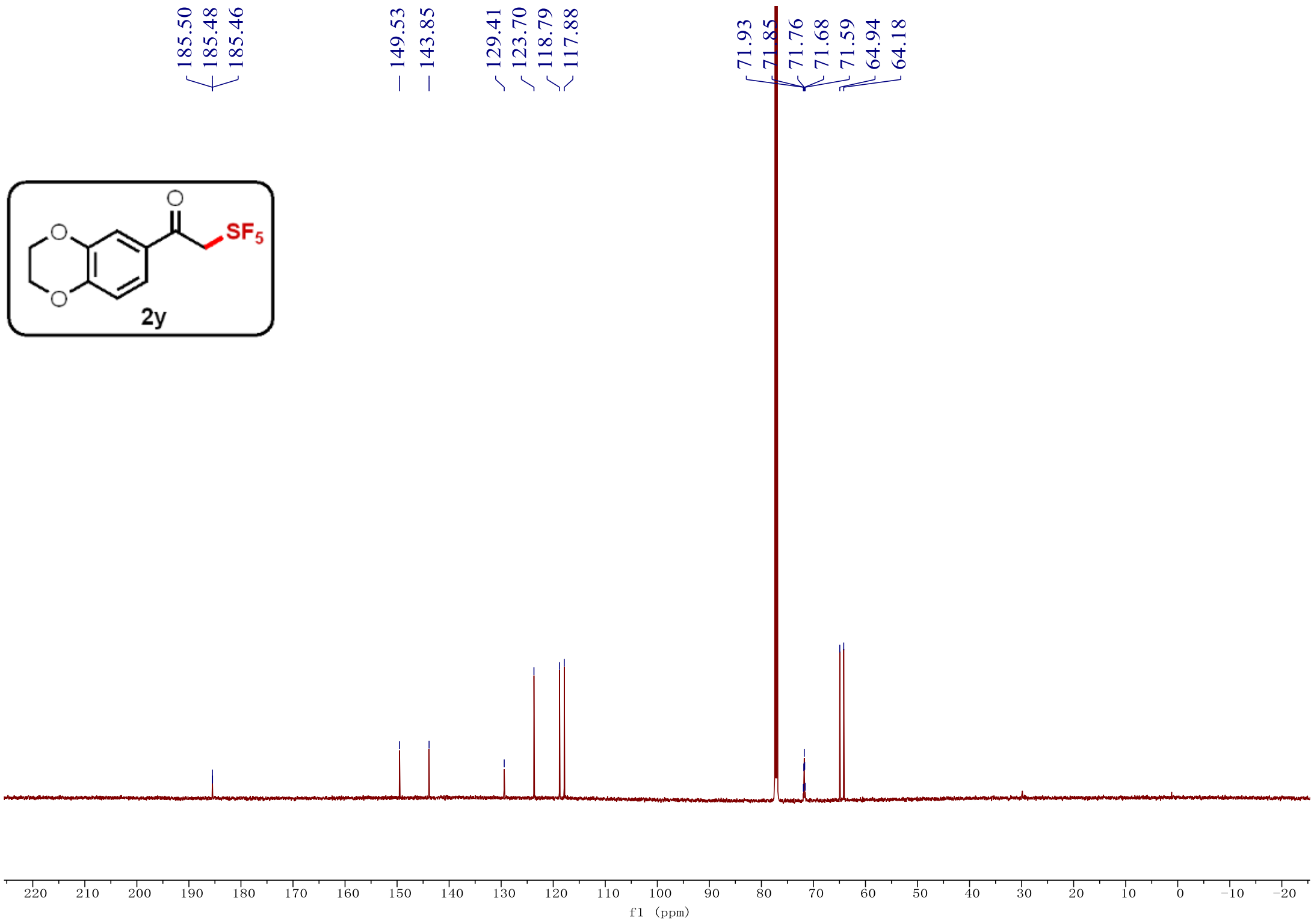

Supplementary Figure 74. ^{13}C NMR Spectrum of Compound 2y (151 MHz, $CDCl_3$, 25 °C)

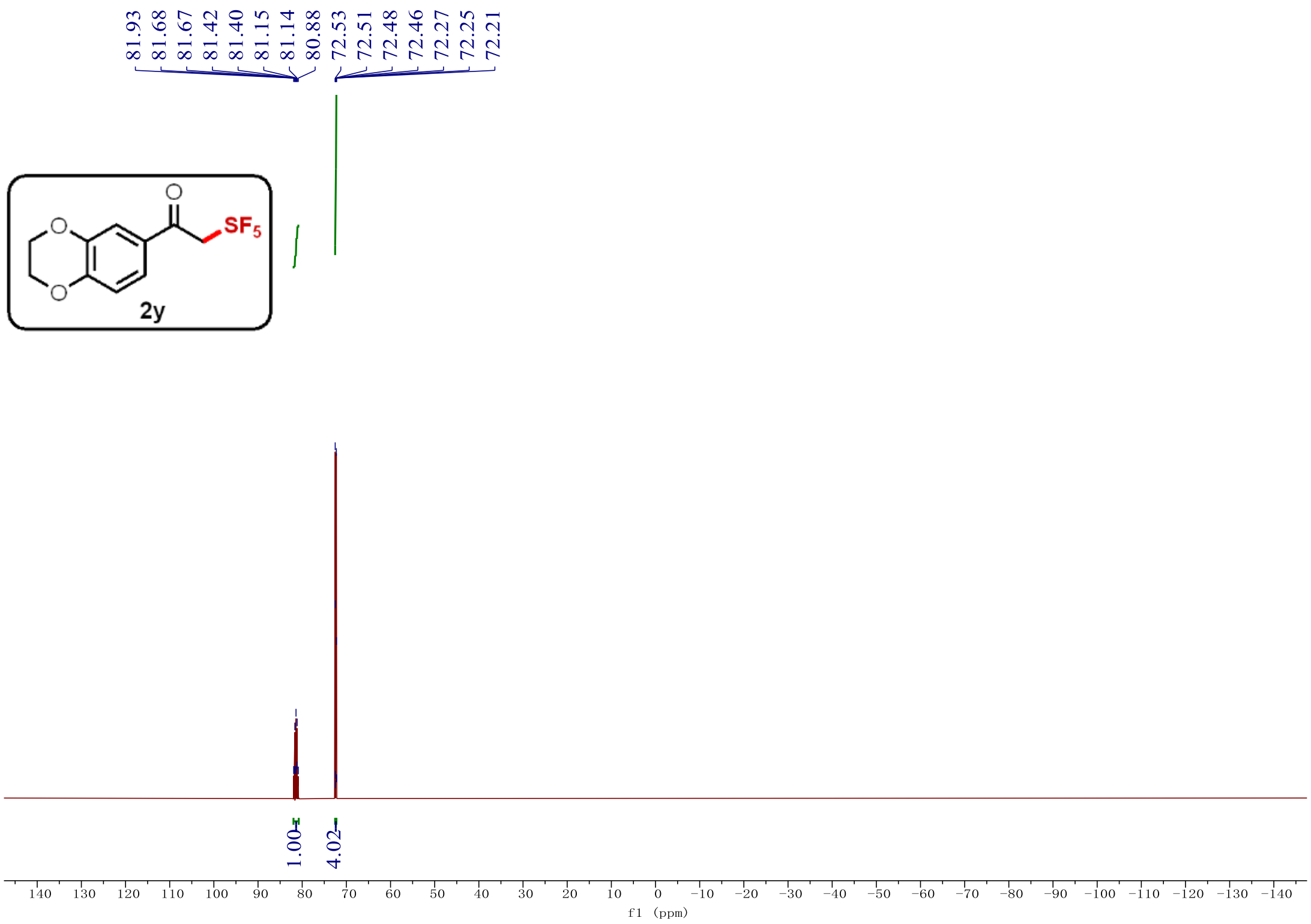

Supplementary Figure 75. [19]**F NMR Spectrum of Compound 2y (565 MHz, $CDCl_3$, 25 °C)**

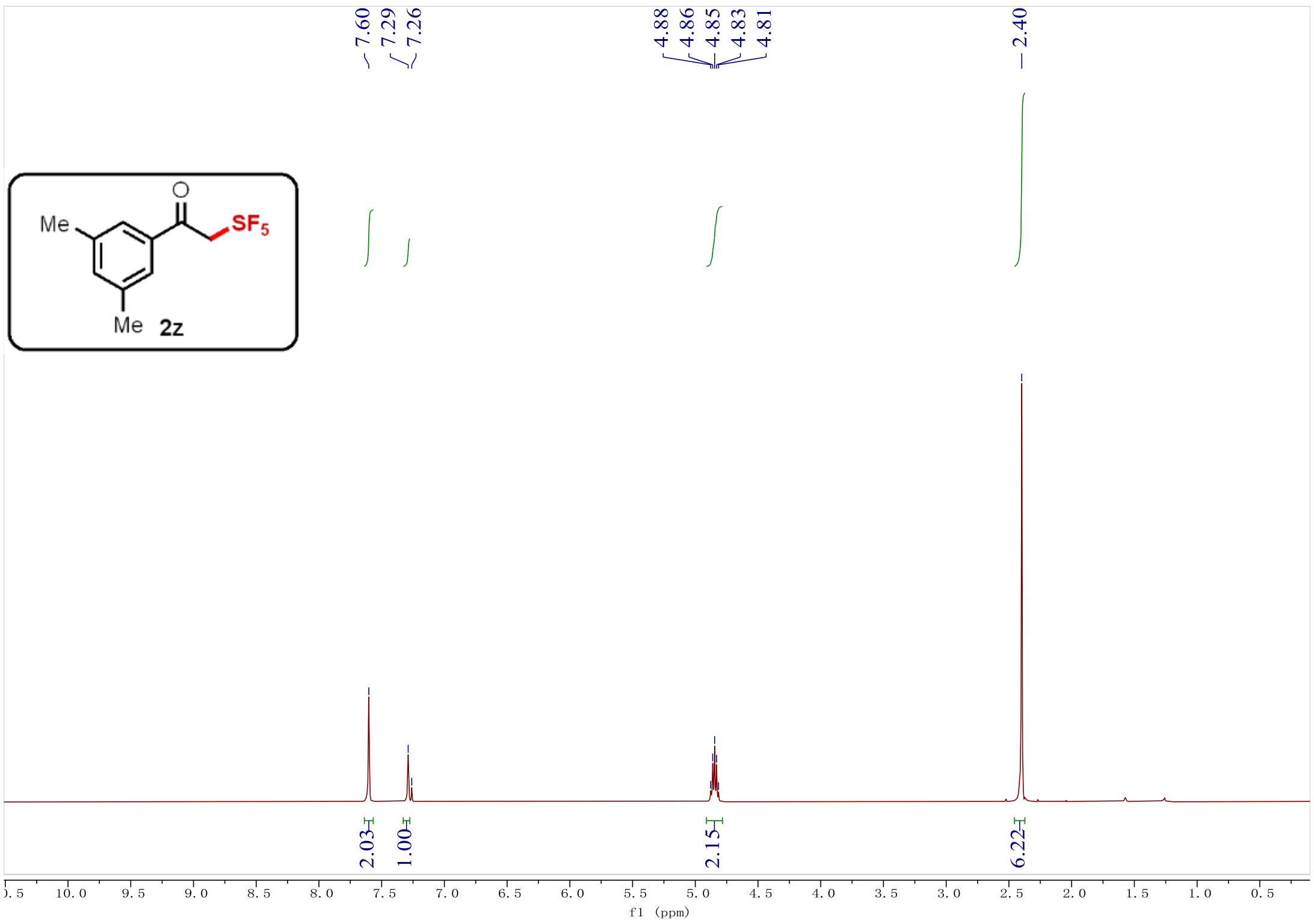

Supplementary Figure 76. [1]**H NMR Spectrum of Compound 2z (500 MHz, $CDCl_3$, 25 °C)**

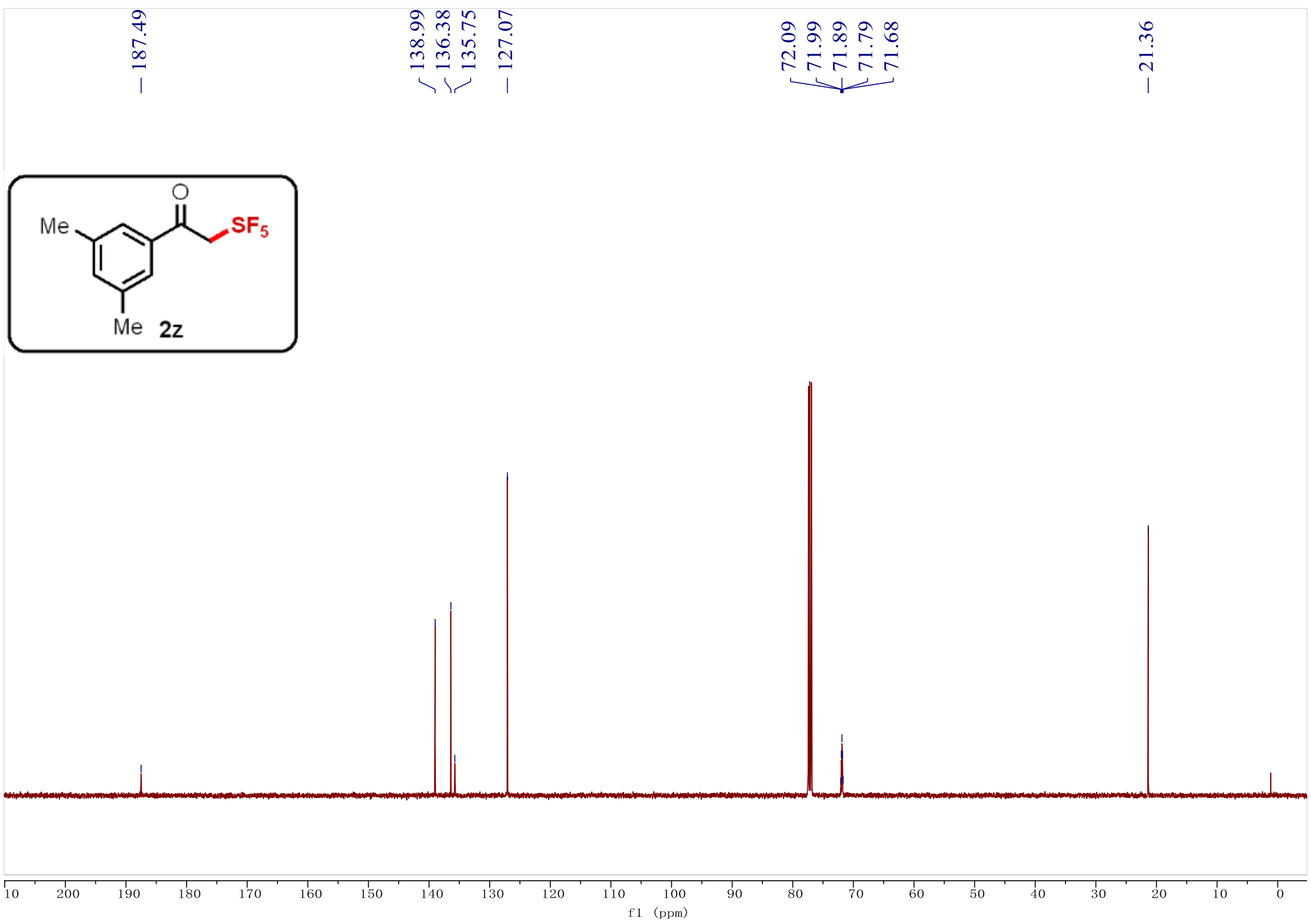

Supplementary Figure 77. ^{13}C NMR Spectrum of Compound 2z (126 MHz, $CDCl_3$, 25 ˚C)

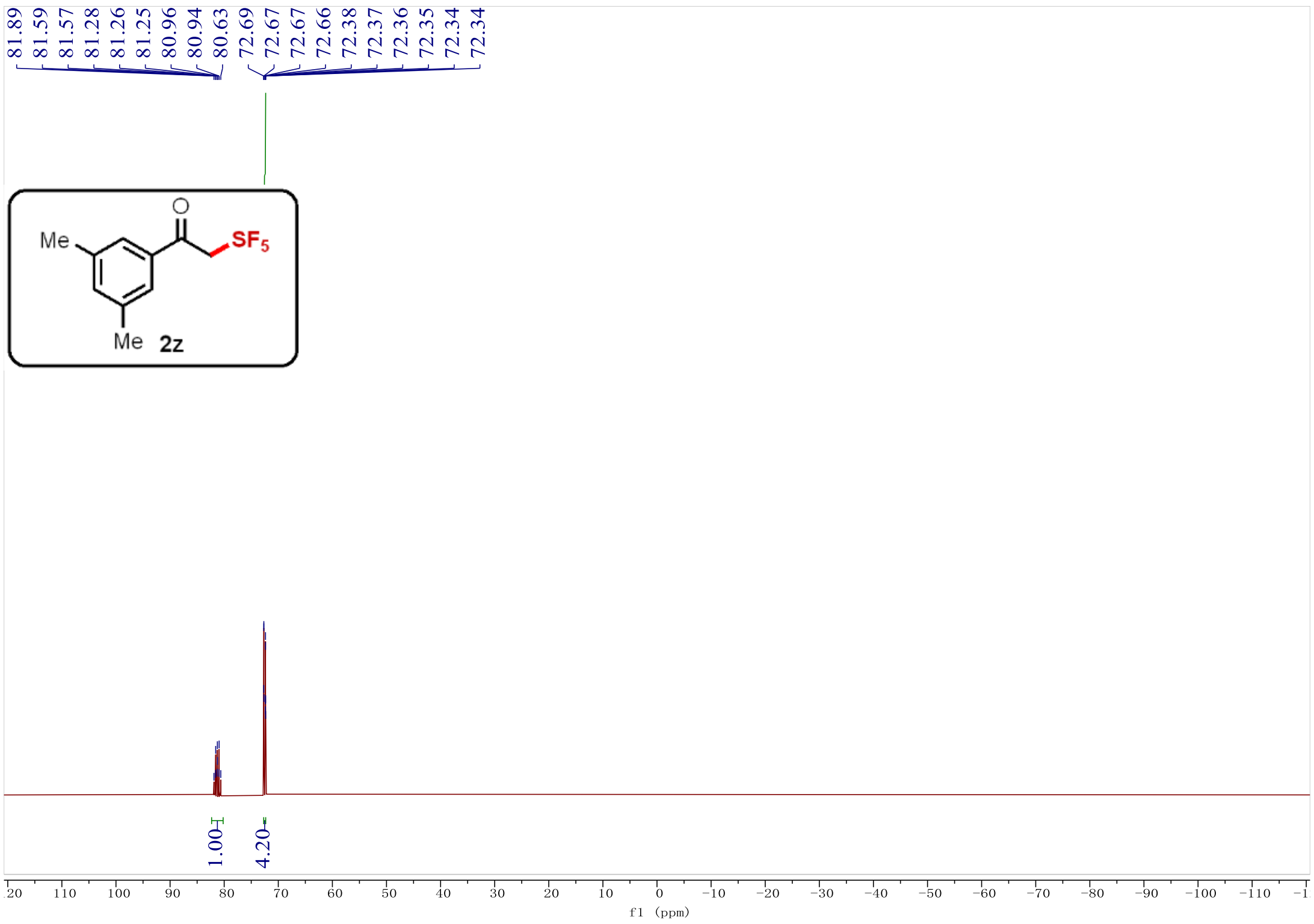

Supplementary Figure 78. ^{19}F NMR Spectrum of Compound 2z (471 MHz, $CDCl_3$, 25 ˚C)

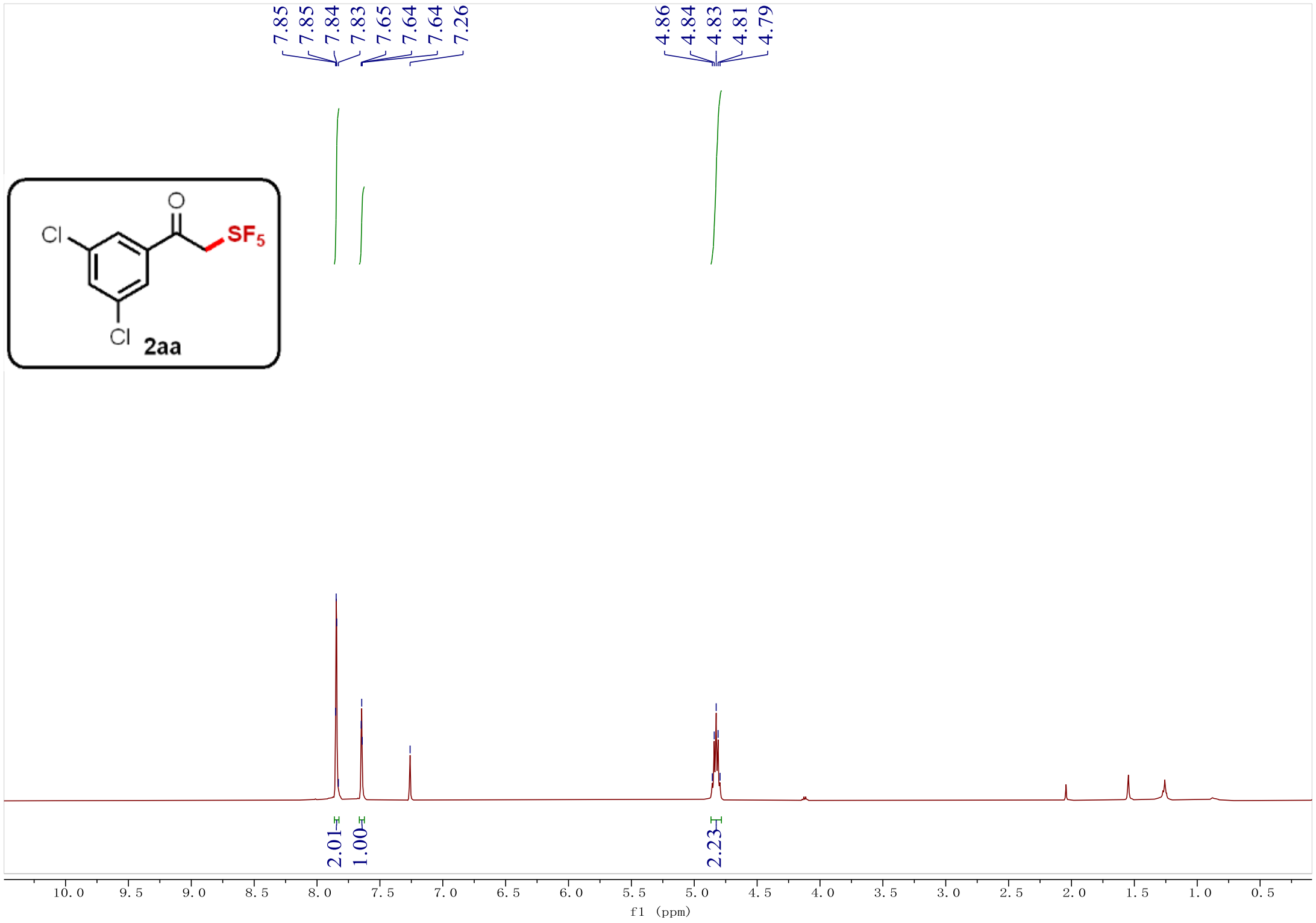

Supplementary Figure 79. ^{1}H NMR Spectrum of Compound 2aa (500 MHz, $CDCl_3$, 25 °C)

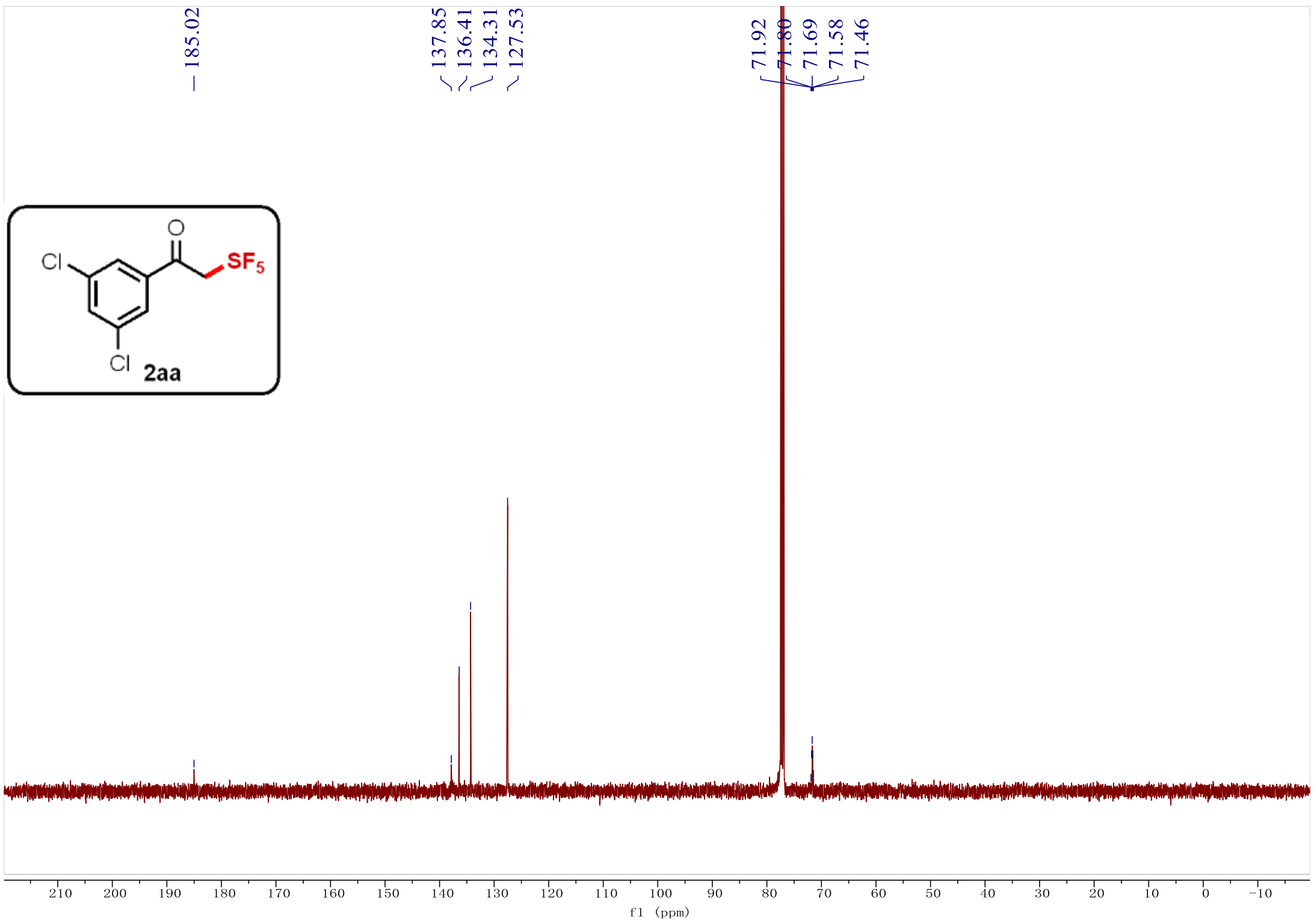

Supplementary Figure 80. ^{13}C NMR Spectrum of Compound 2aa (126 MHz, $CDCl_3$, 25 °C)

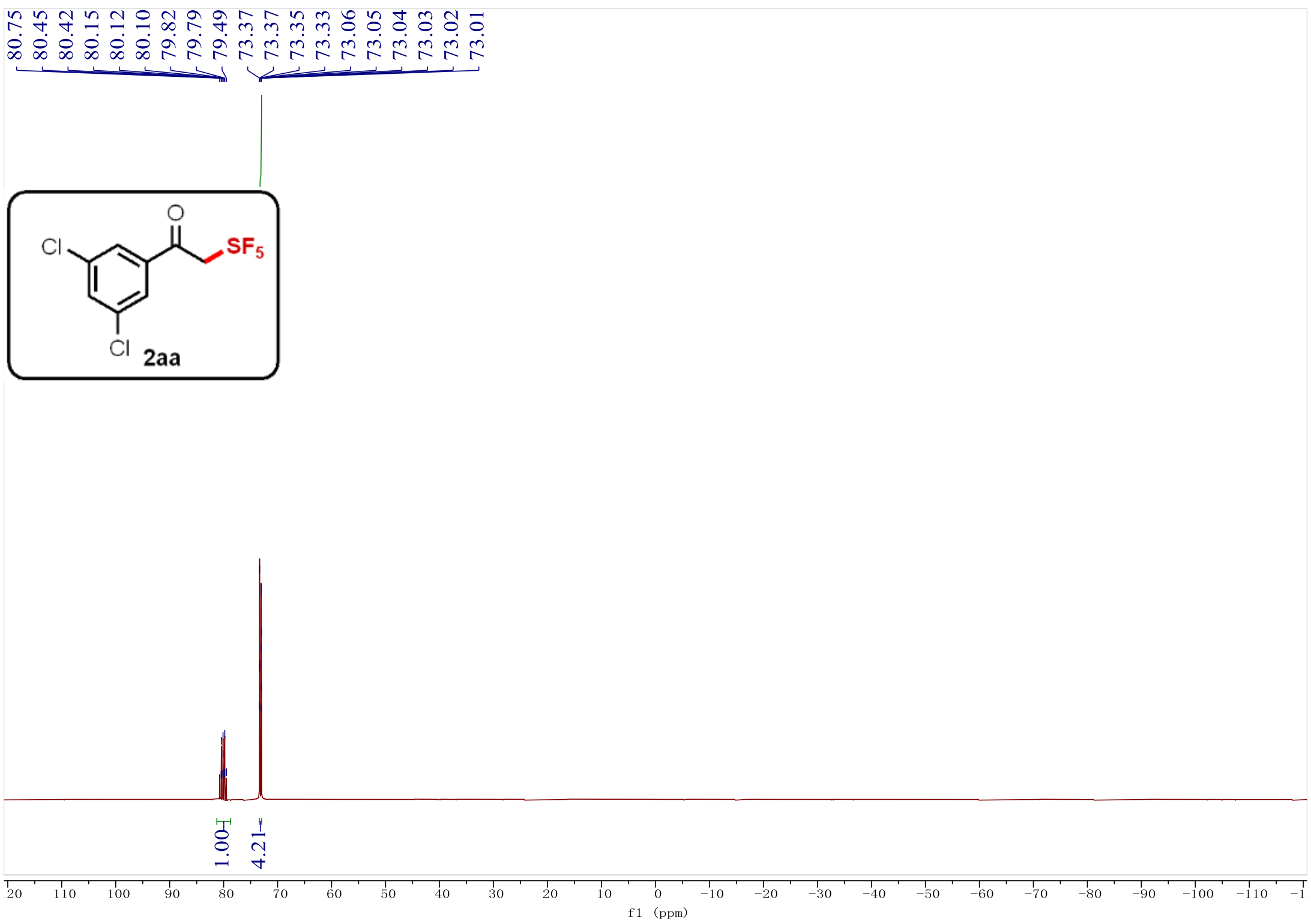

Supplementary Figure 81. ^{19}**F NMR Spectrum of Compound 2aa (471 MHz,** $CDCl_3$**, 25 °C)**

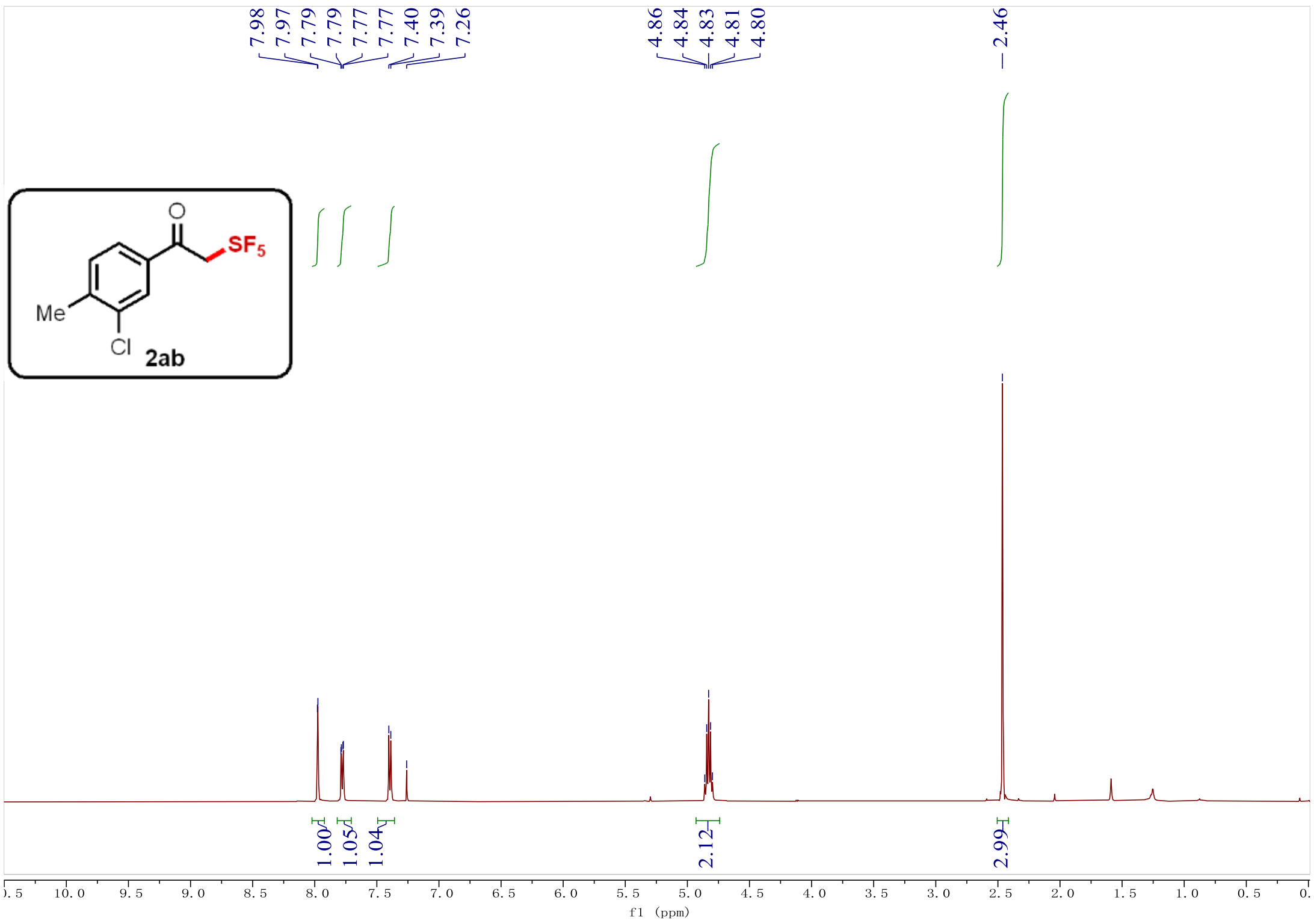

Supplementary Figure 82. ^{1}**H NMR Spectrum of Compound 2ab (500 MHz,** $CDCl_3$**, 25 °C)**

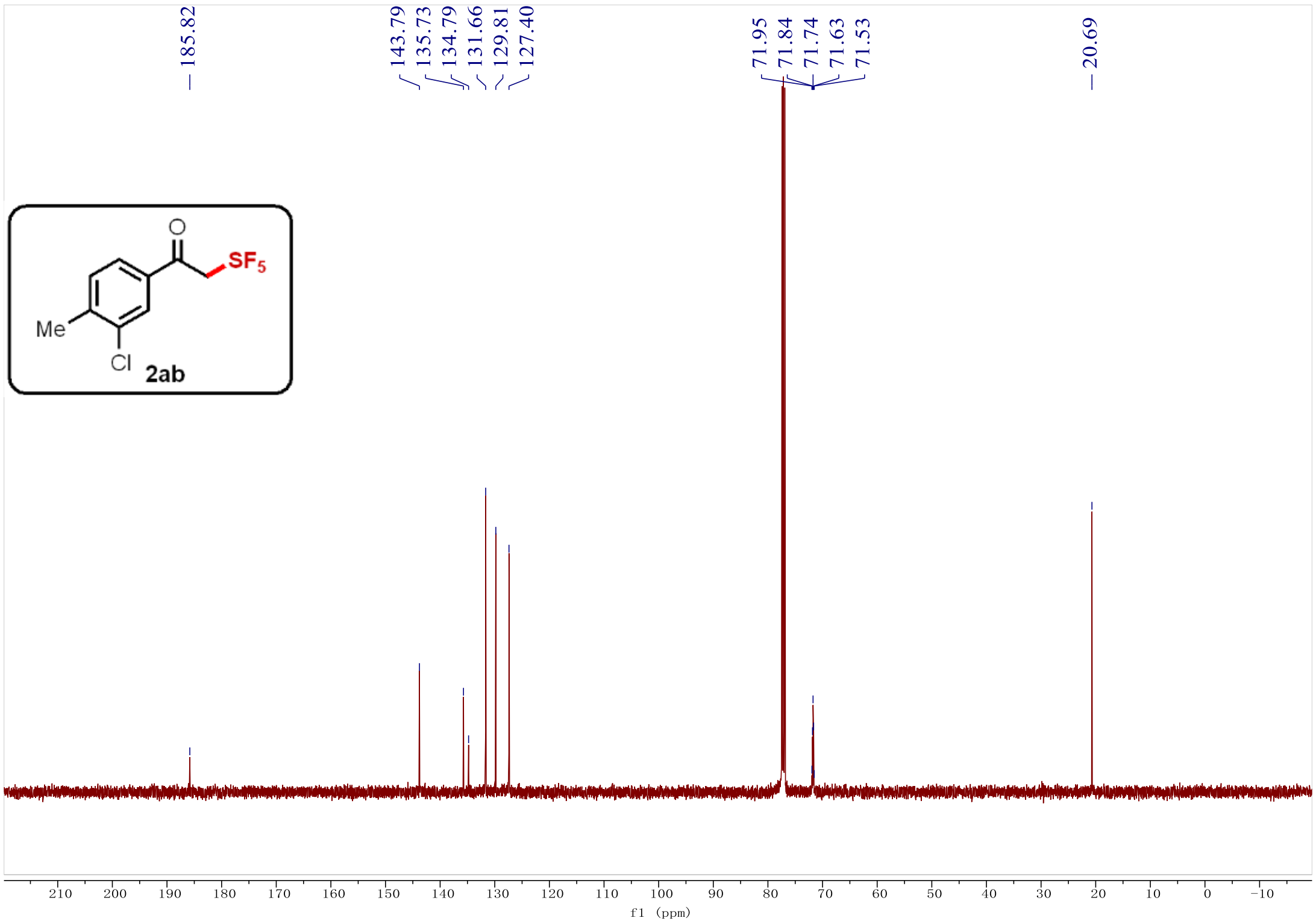

Supplementary Figure 83. ^{13}C NMR Spectrum of Compound 2ab (126 MHz, $CDCl_3$, 25 °C)

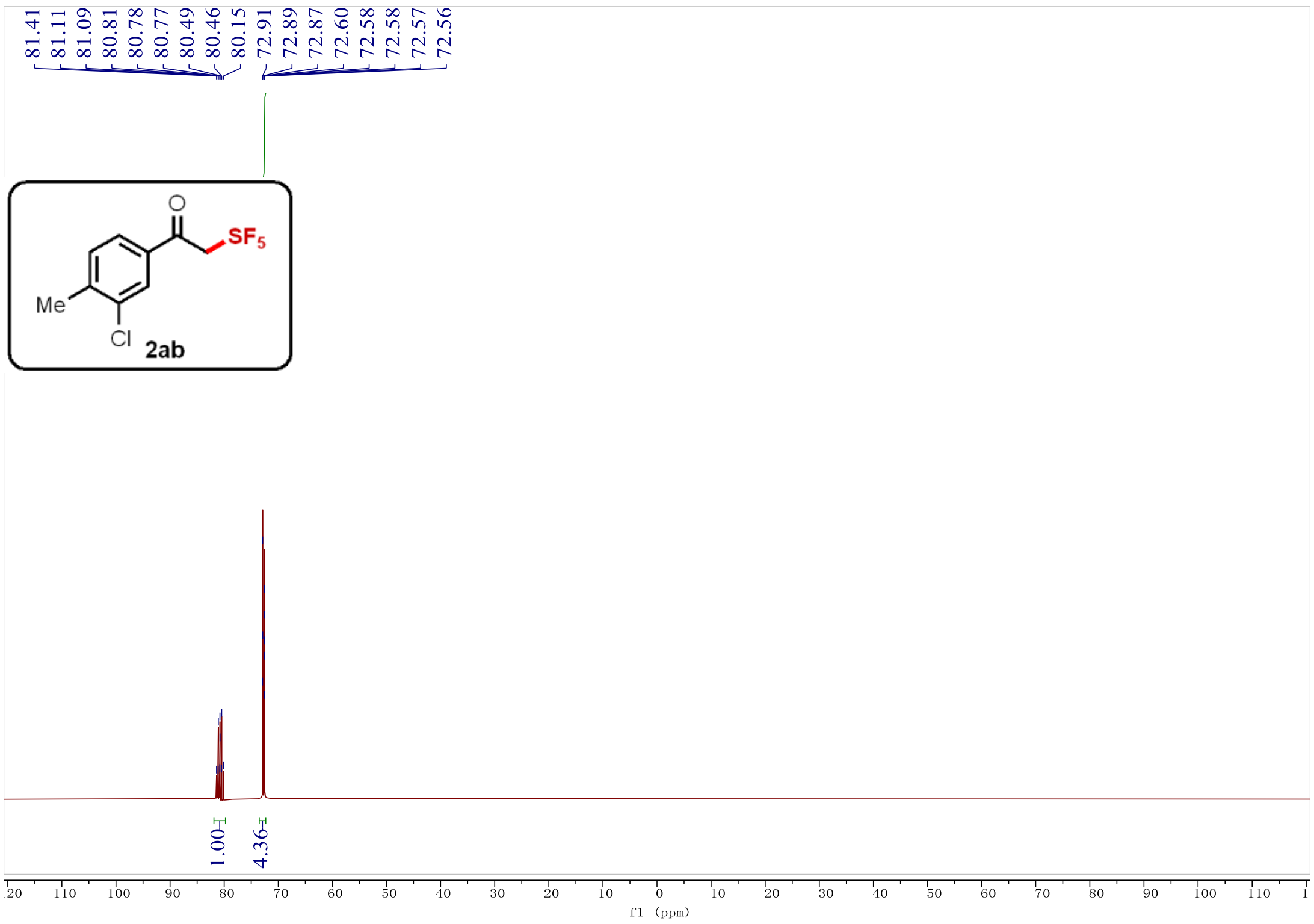

Supplementary Figure 84. ^{19}F NMR Spectrum of Compound 2ab (471 MHz, $CDCl_3$, 25 °C)

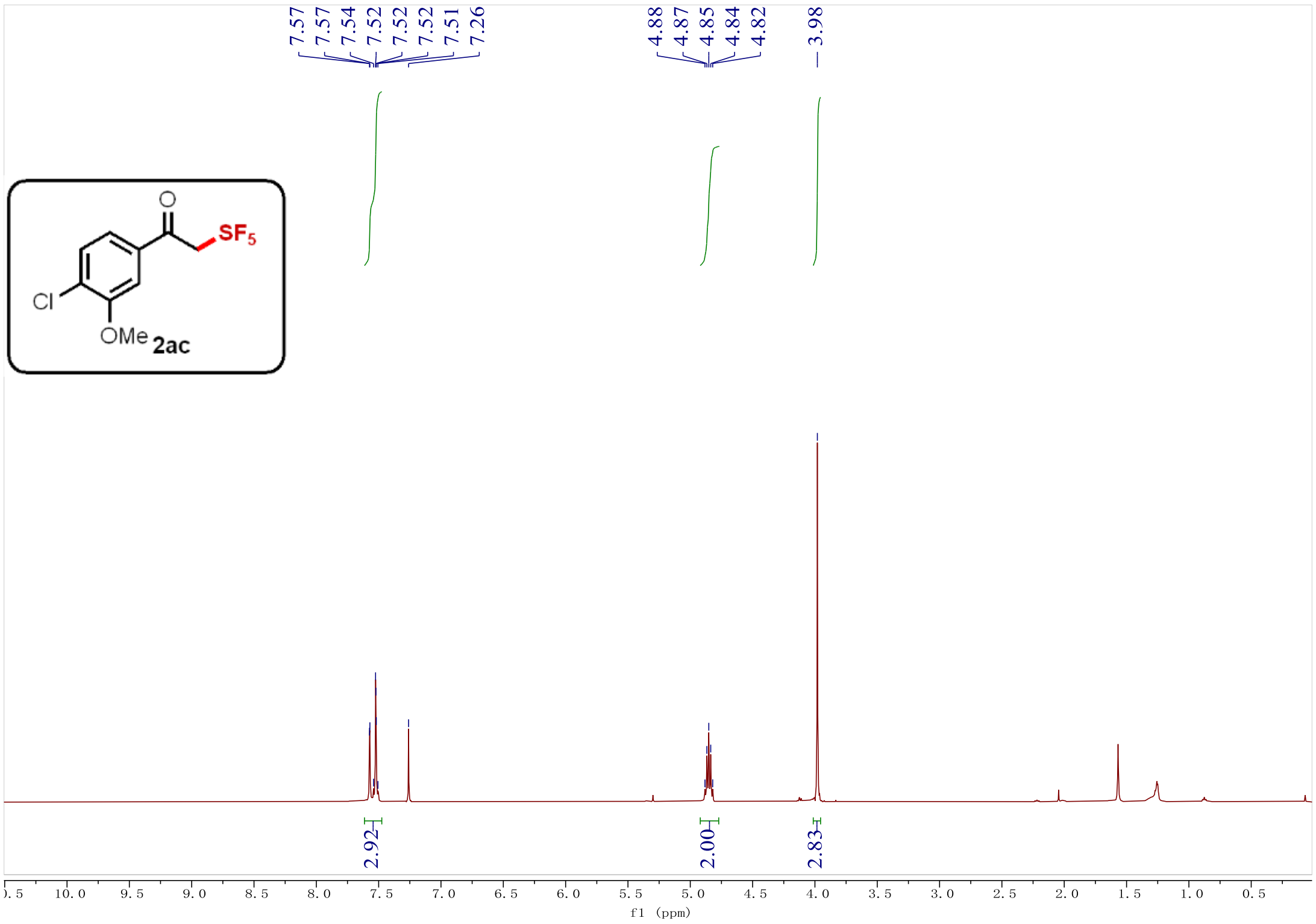

Supplementary Figure 85. ^{1}H NMR Spectrum of Compound 2ac (500 MHz, $CDCl_3$, 25 ˚C)

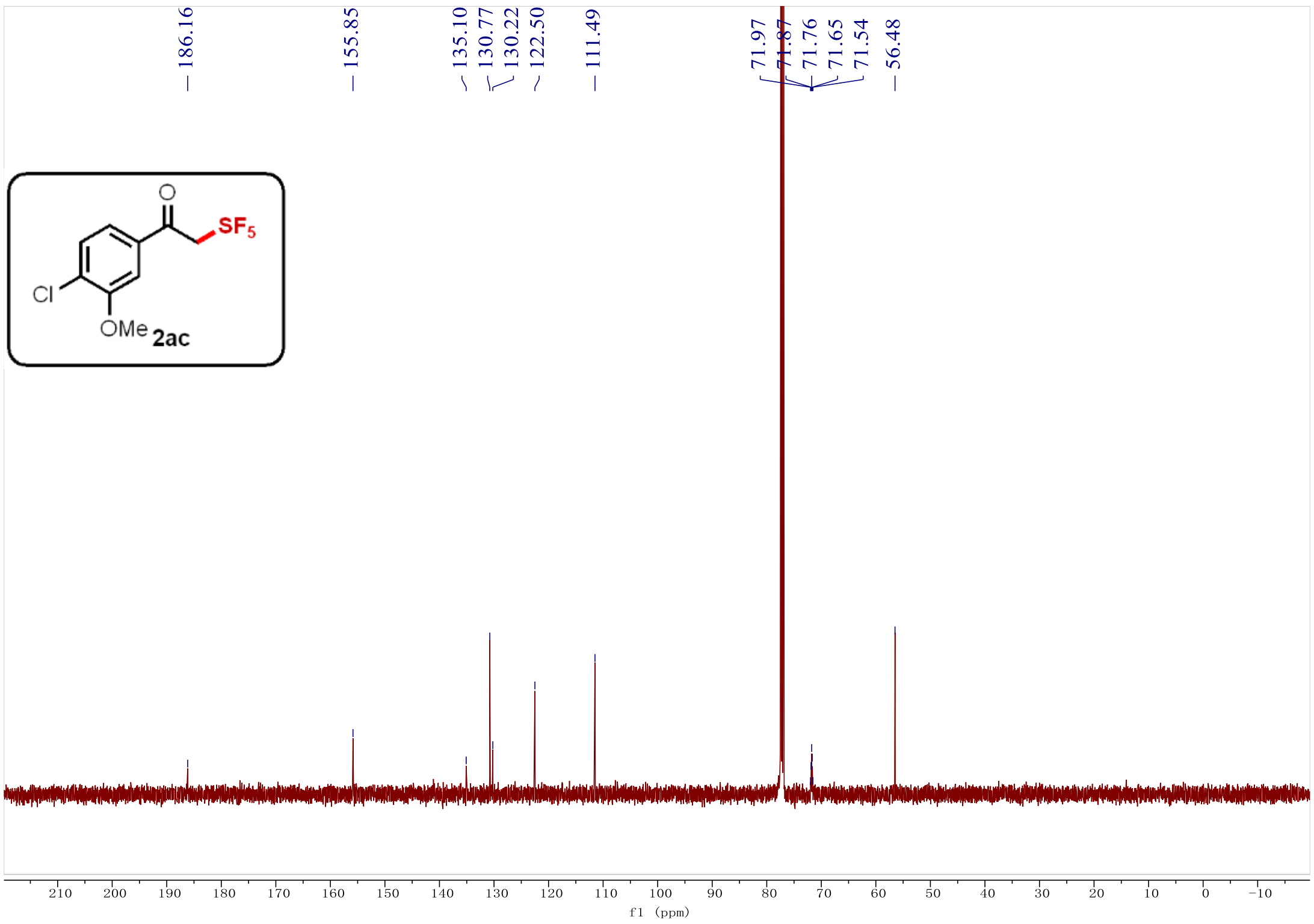

Supplementary Figure 86. ^{13}C NMR Spectrum of Compound 2ac (126 MHz, $CDCl_3$, 25 ˚C)

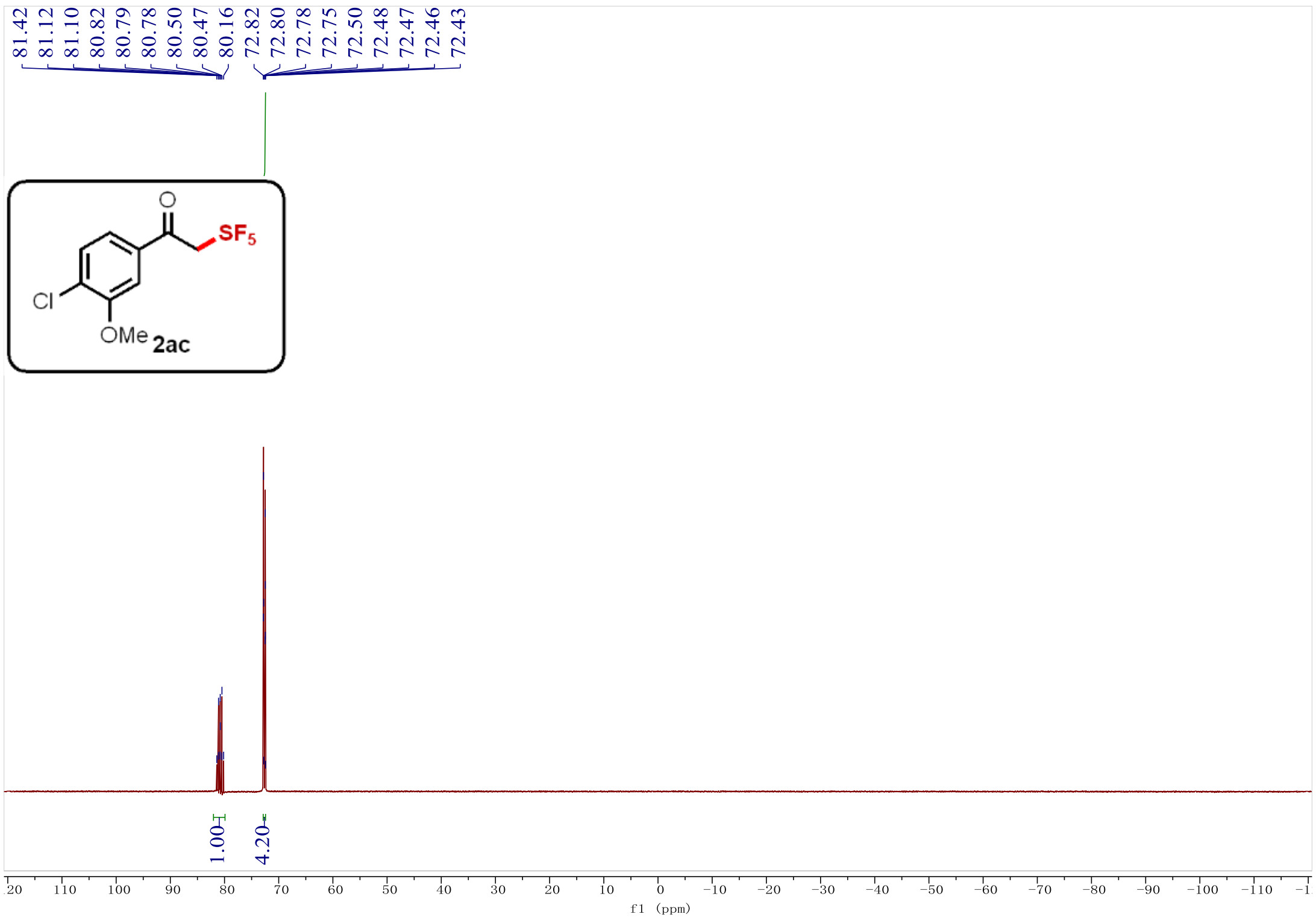

Supplementary Figure 87. ^{19}F NMR Spectrum of Compound 2ac (471 MHz, $CDCl_3$, 25 ˚C)

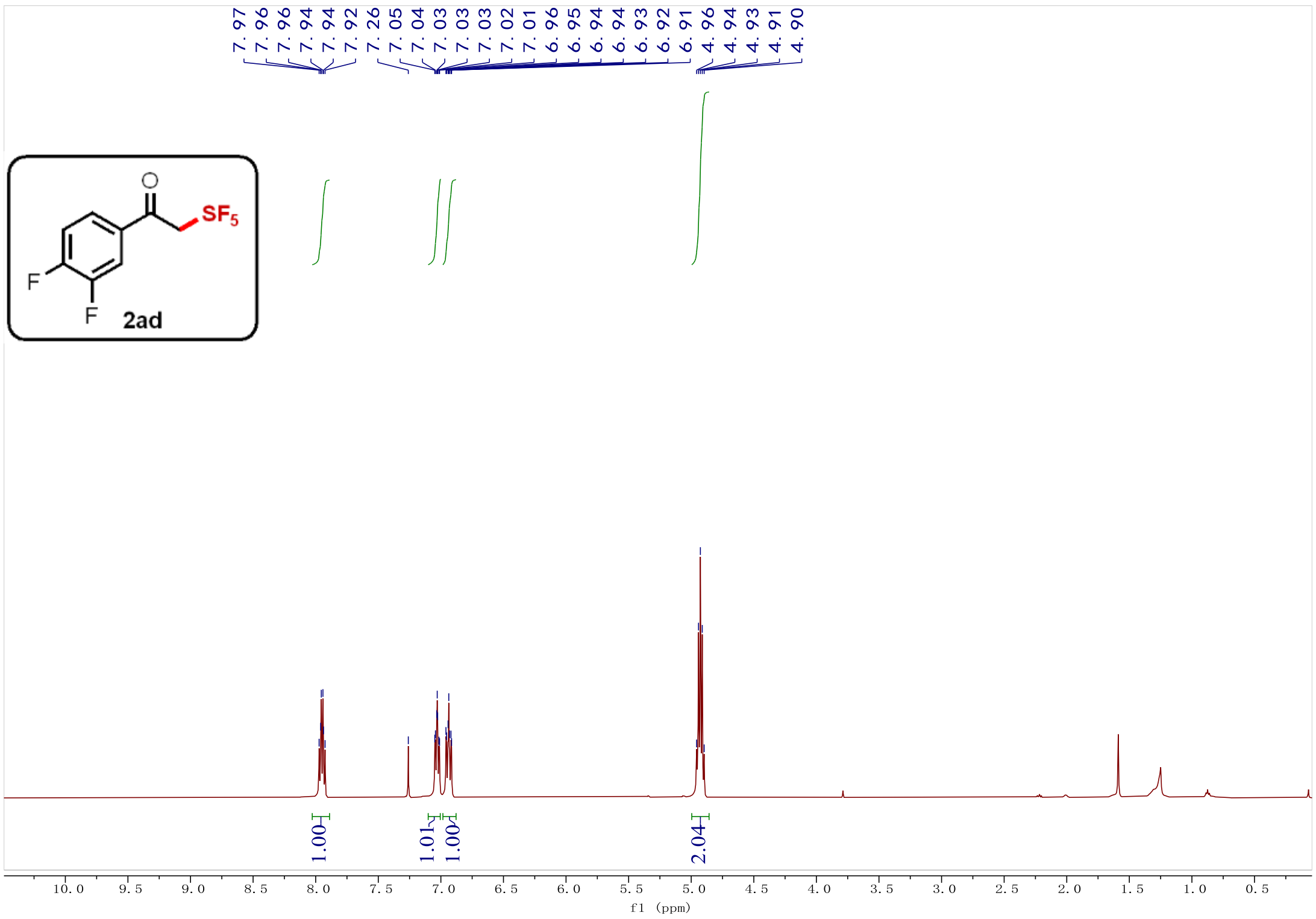

Supplementary Figure 88. ^{1}H NMR Spectrum of Compound 2ad (500 MHz, $CDCl_3$, 25 ˚C)

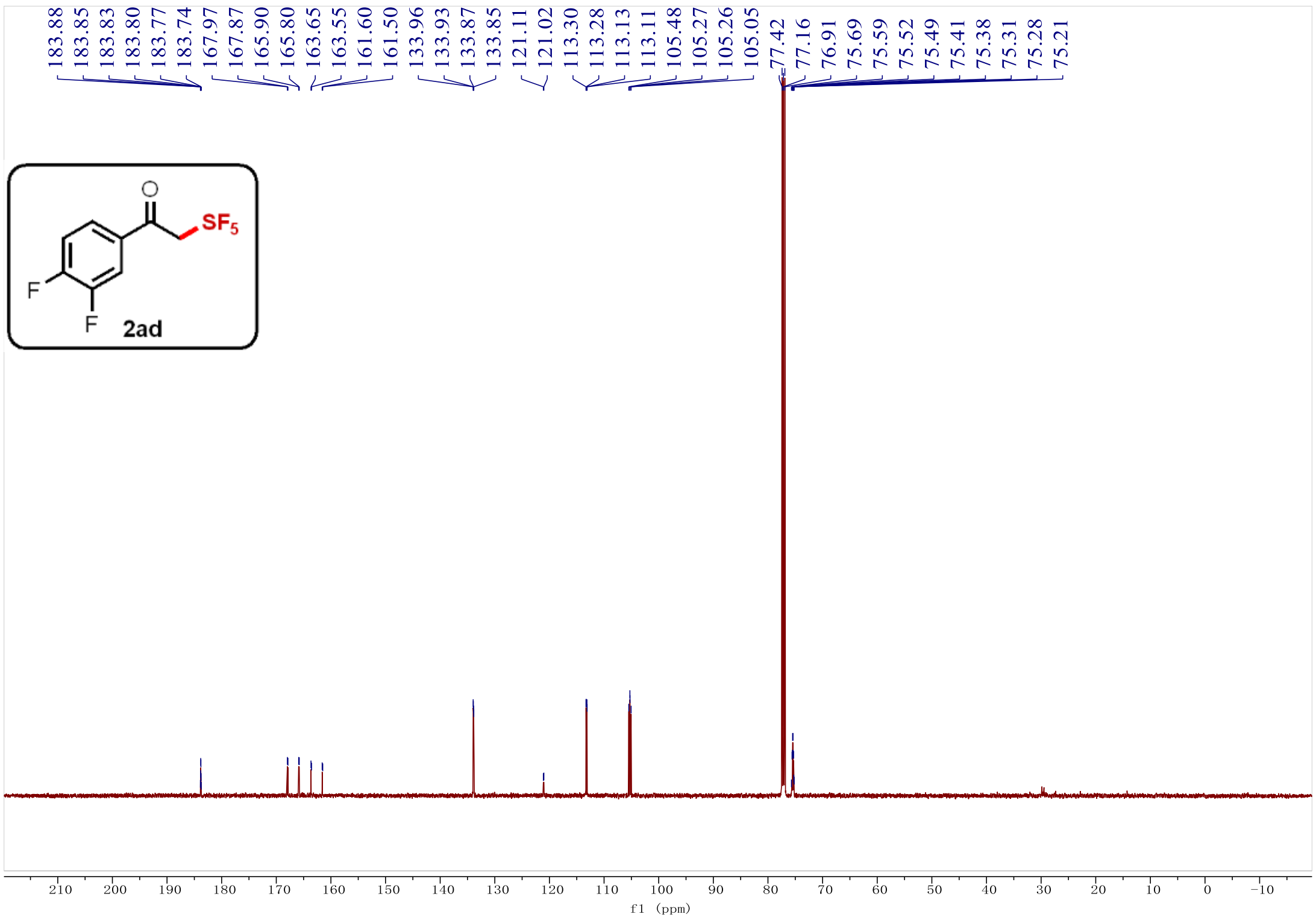

Supplementary Figure 89. ^{13}C NMR Spectrum of Compound 2ad (126 MHz, $CDCl_3$, 25 °C)

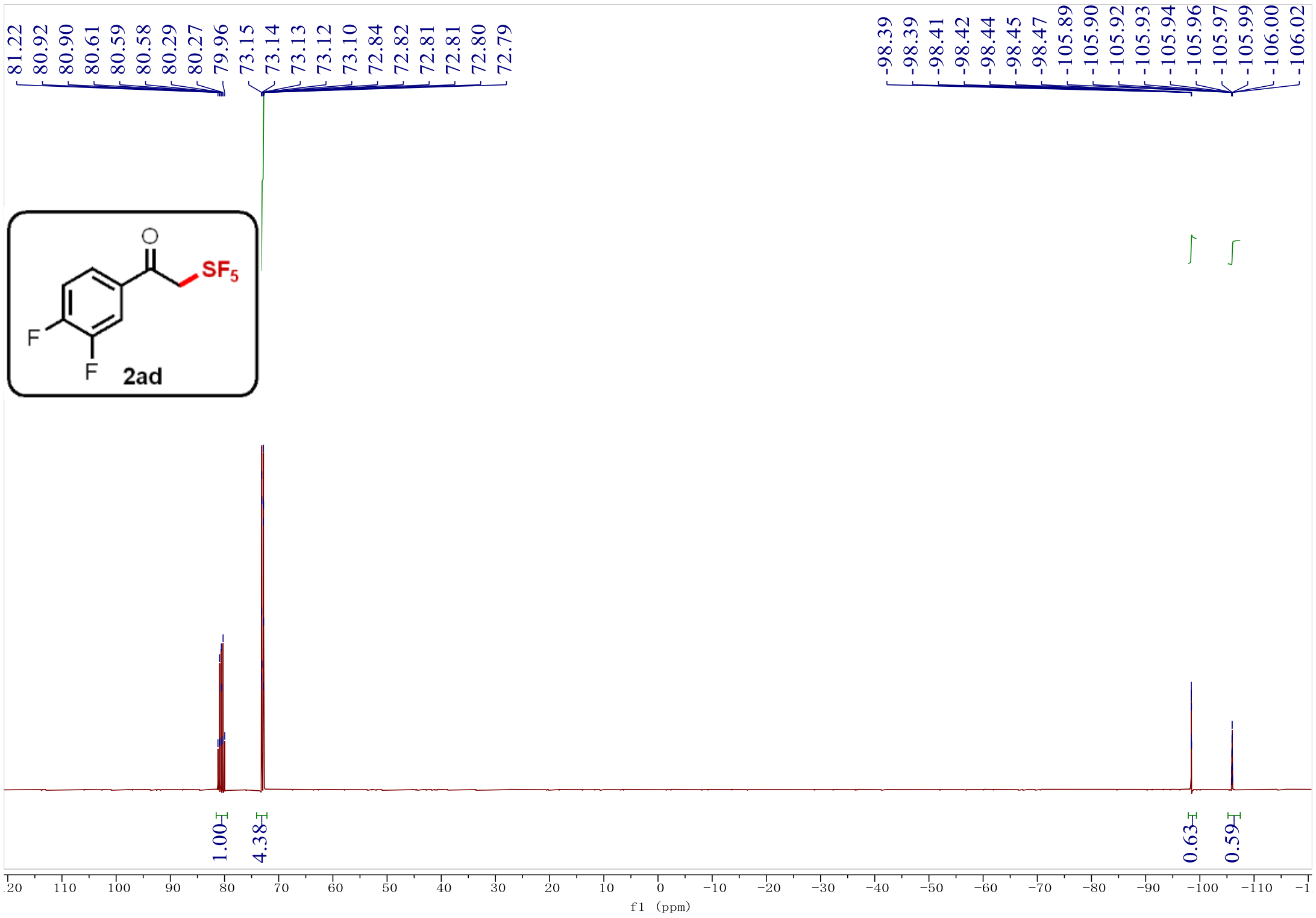

Supplementary Figure 90. ^{19}F NMR Spectrum of Compound 2ad (471 MHz, $CDCl_3$, 25 °C)

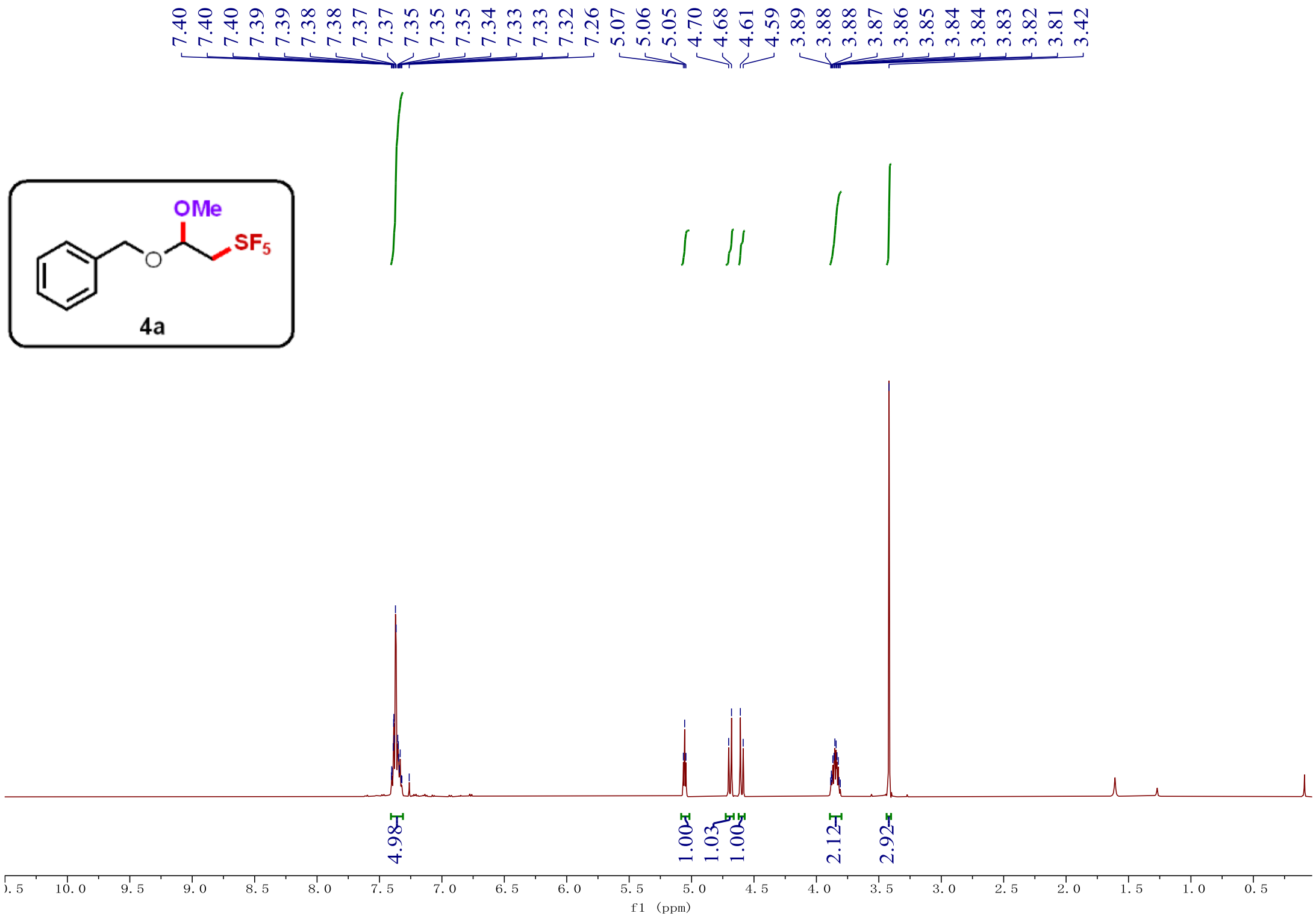

Supplementary Figure 91. ^{1}H NMR Spectrum of Compound 4a (500 MHz, $CDCl_3$, 25 ˚C)

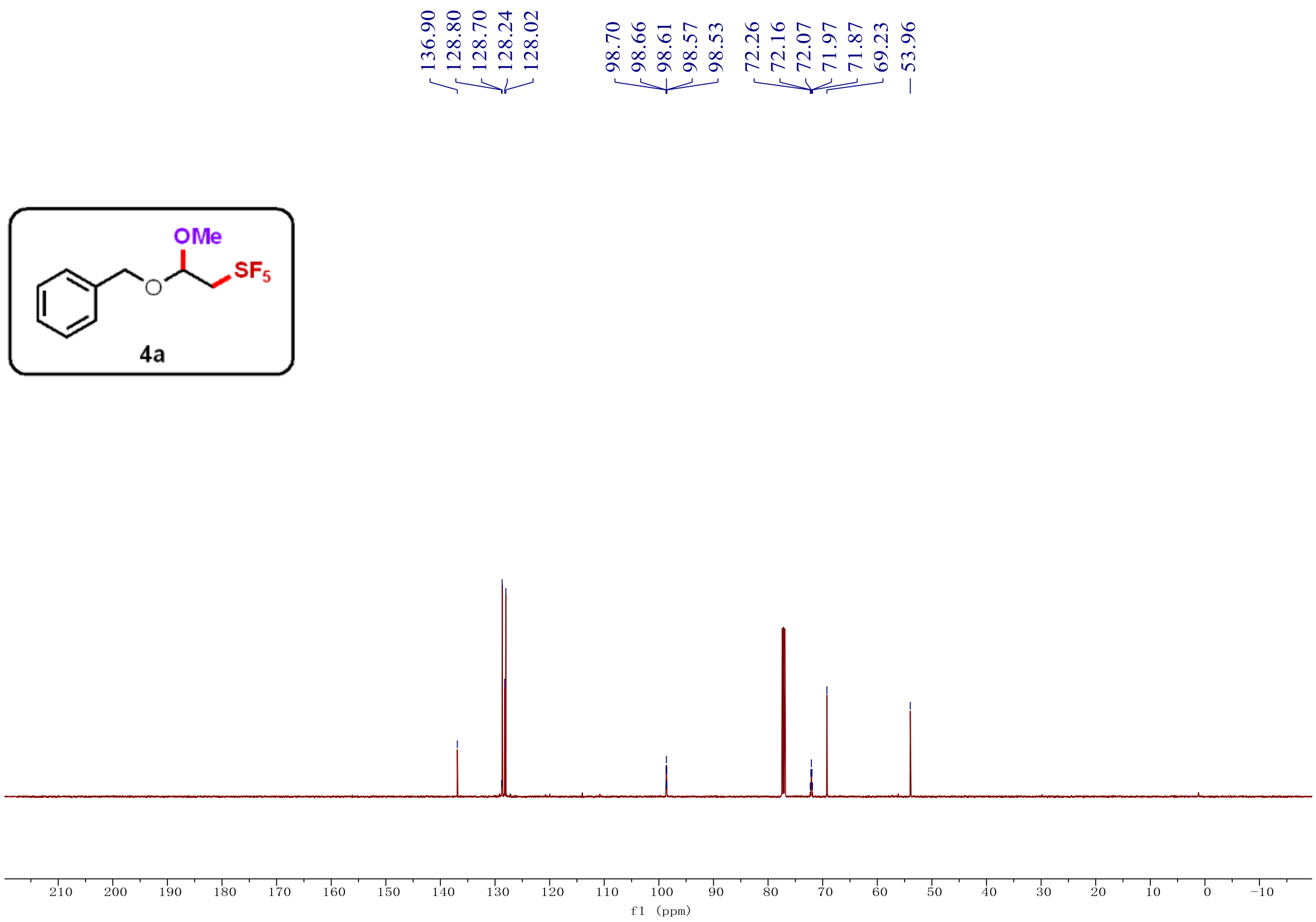

Supplementary Figure 92. ^{13}C NMR Spectrum of Compound 4a (126 MHz, $CDCl_3$, 25 ˚C)

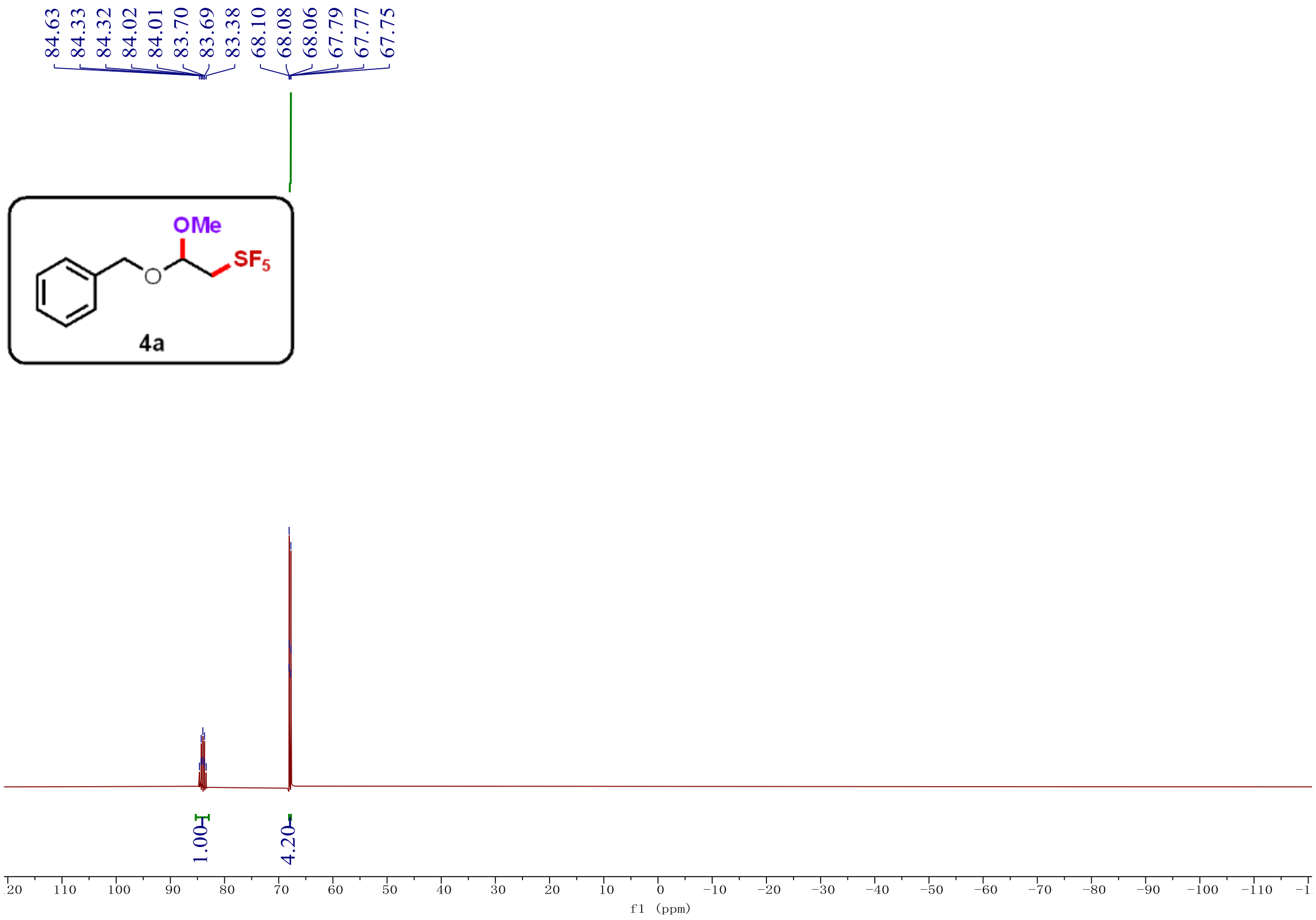

Supplementary Figure 93. ^{19}F NMR Spectrum of Compound 4a (471 MHz, $CDCl_3$, 25 °C)

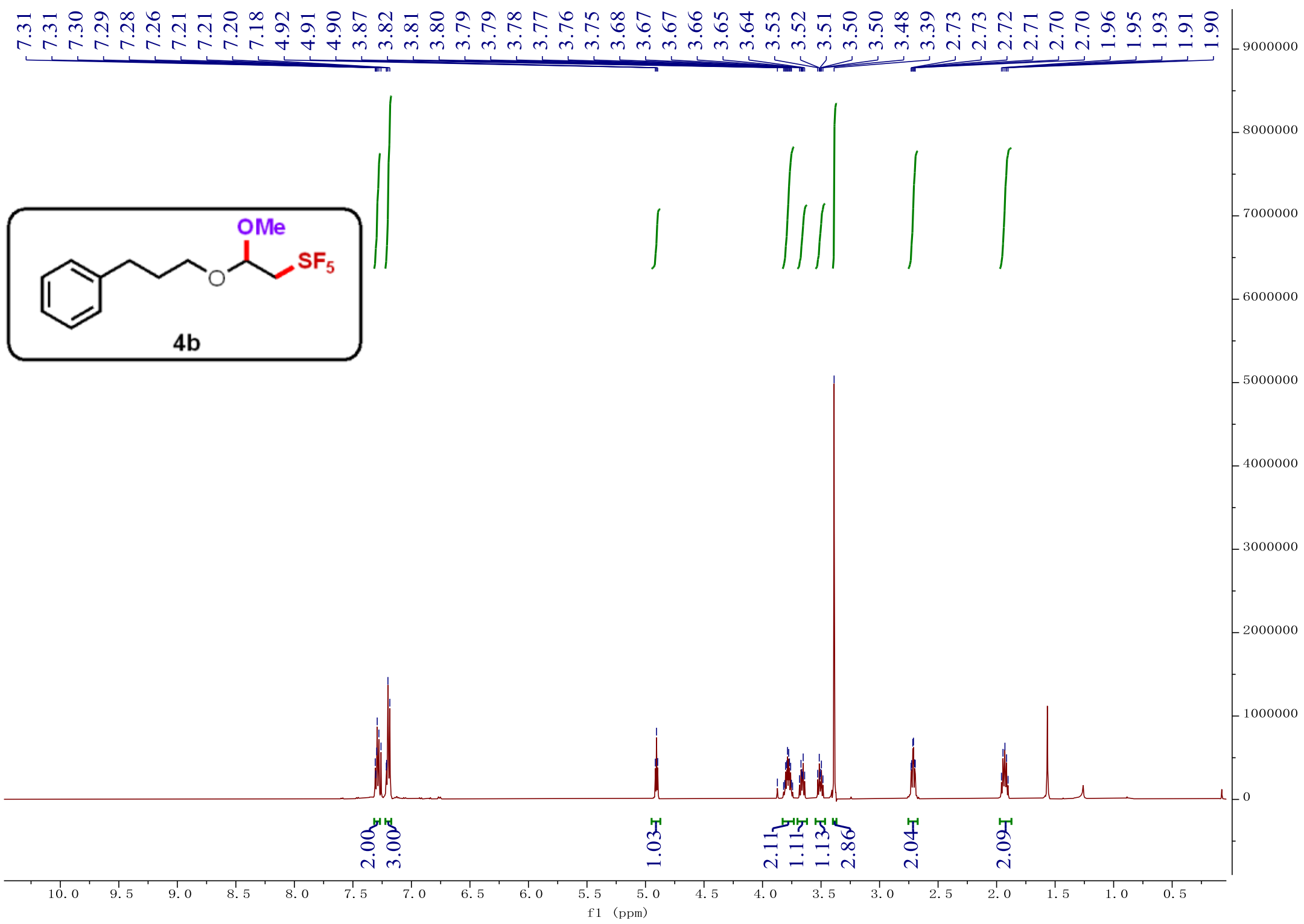

Supplementary Figure 94. ^{1}H NMR Spectrum of Compound 4b (500 MHz, $CDCl_3$, 25 °C)

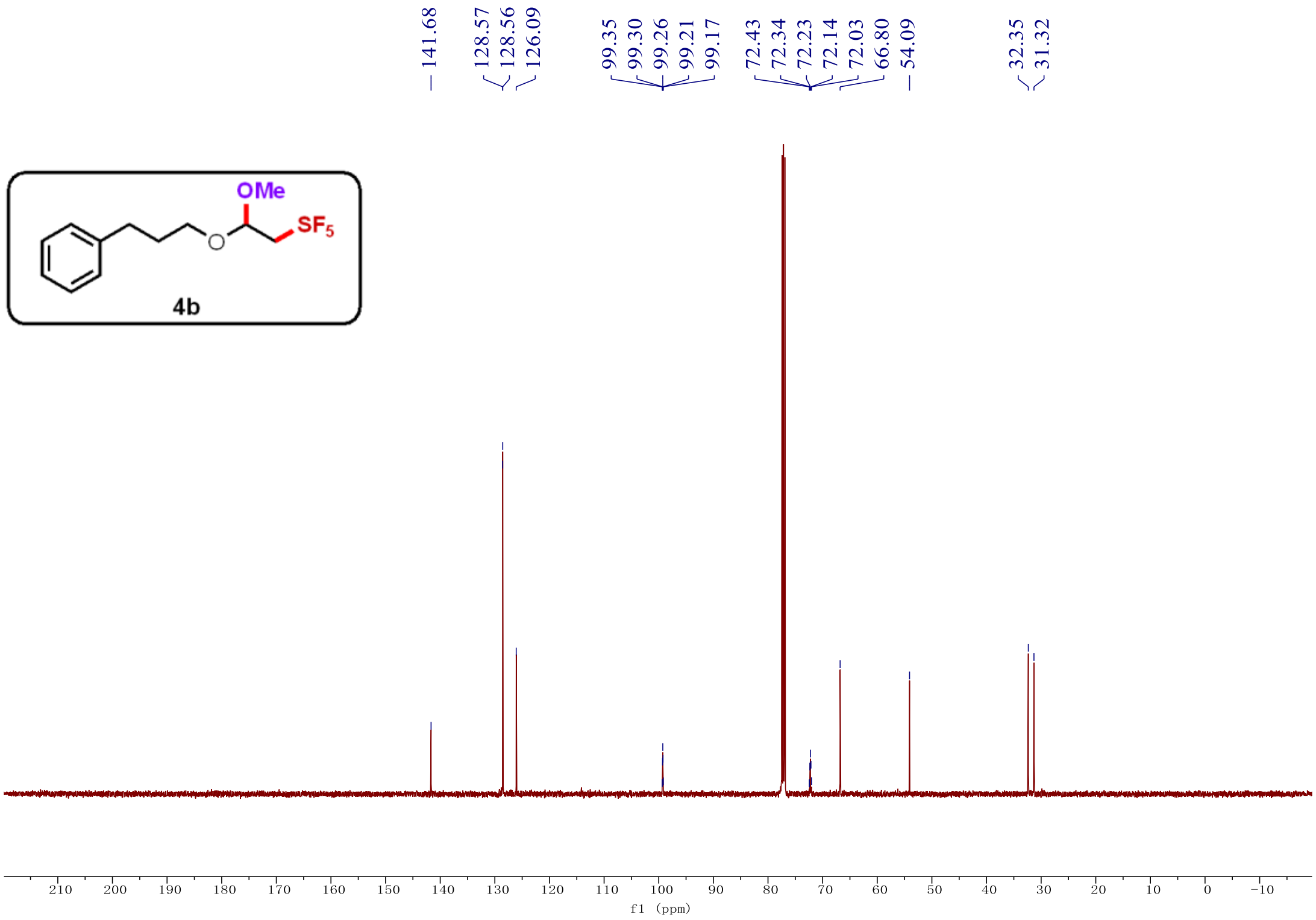

Supplementary Figure 95. ^{13}C NMR Spectrum of Compound 4b (126 MHz, $CDCl_3$, 25 ˚C)

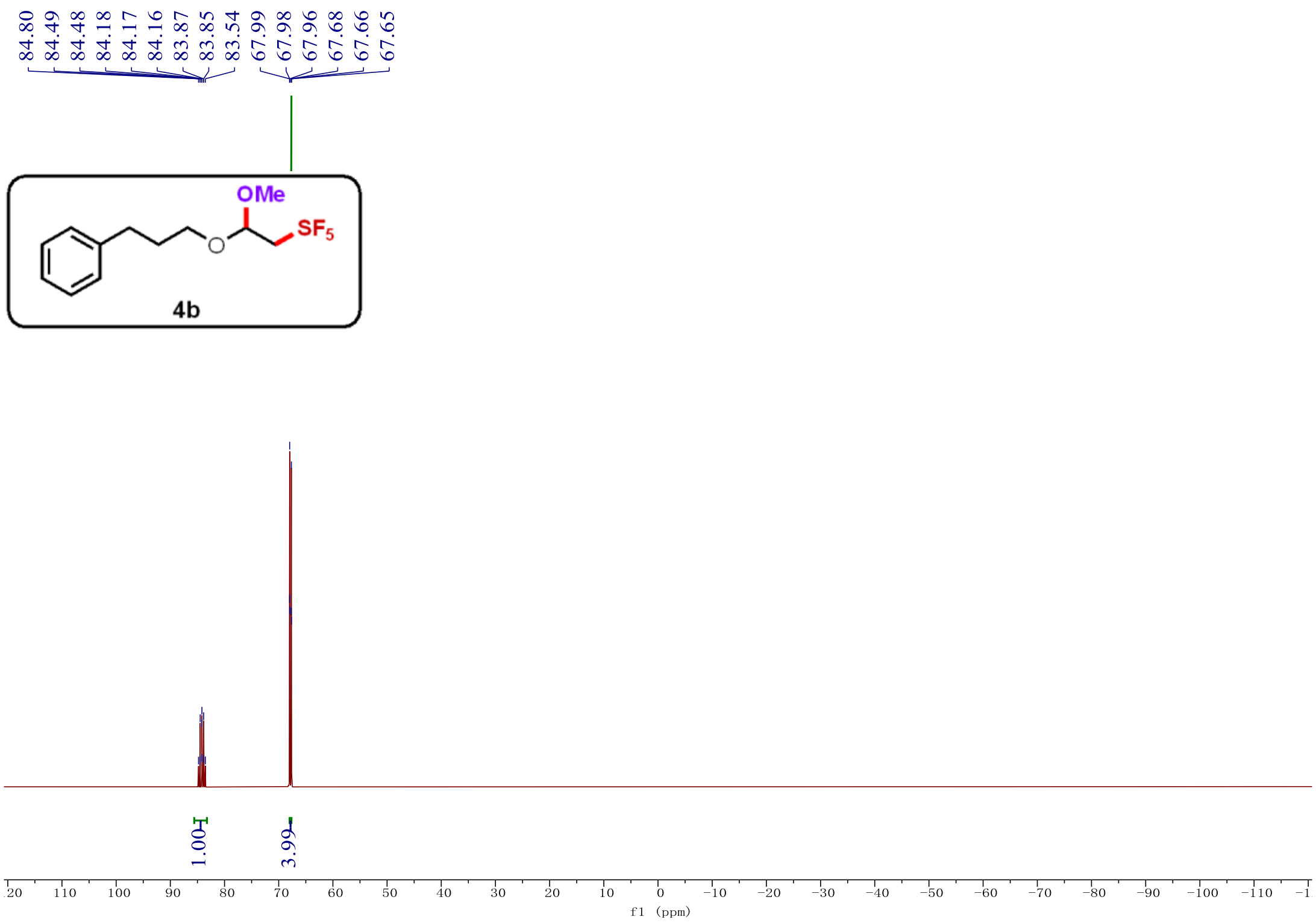

Supplementary Figure 96. ^{19}F NMR Spectrum of Compound 4b (471 MHz, $CDCl_3$, 25 ˚C)

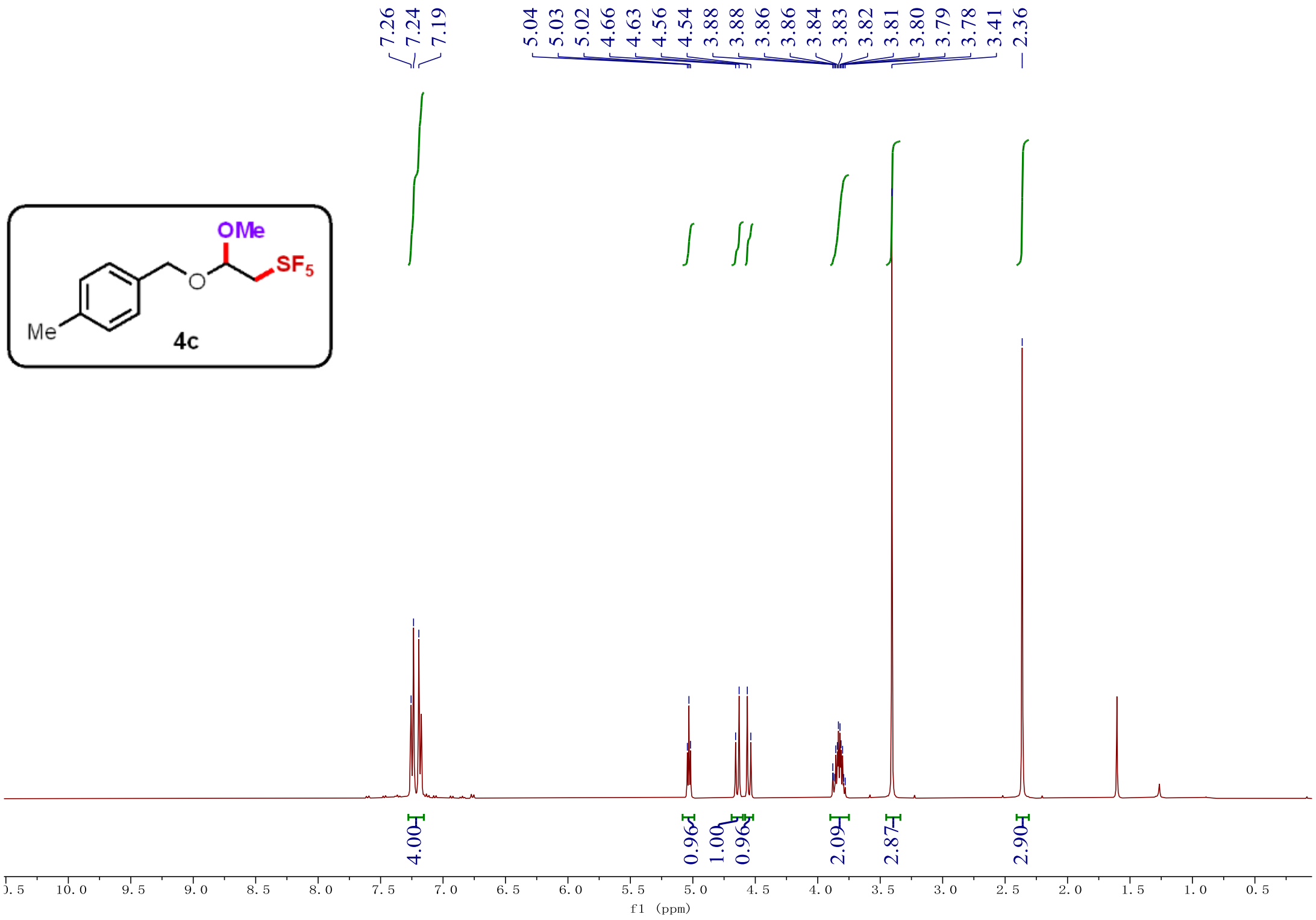

Supplementary Figure 97. ^{1}**H NMR Spectrum of Compound 4c (400 MHz, CDCl$_3$, 25 ˚C)**

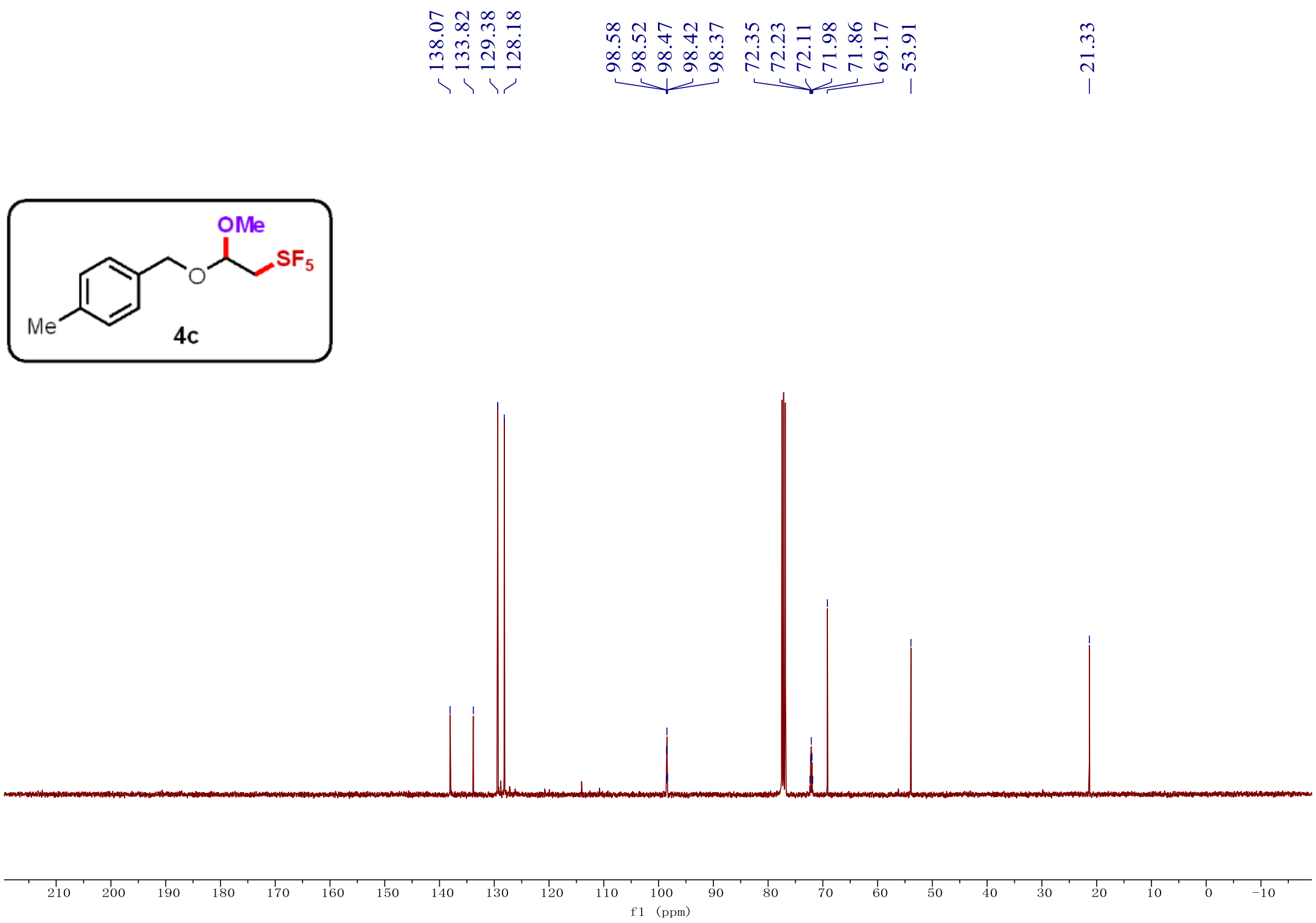

Supplementary Figure 98. ^{13}**C NMR Spectrum of Compound 4c (106 MHz, CDCl$_3$, 25 ˚C)**

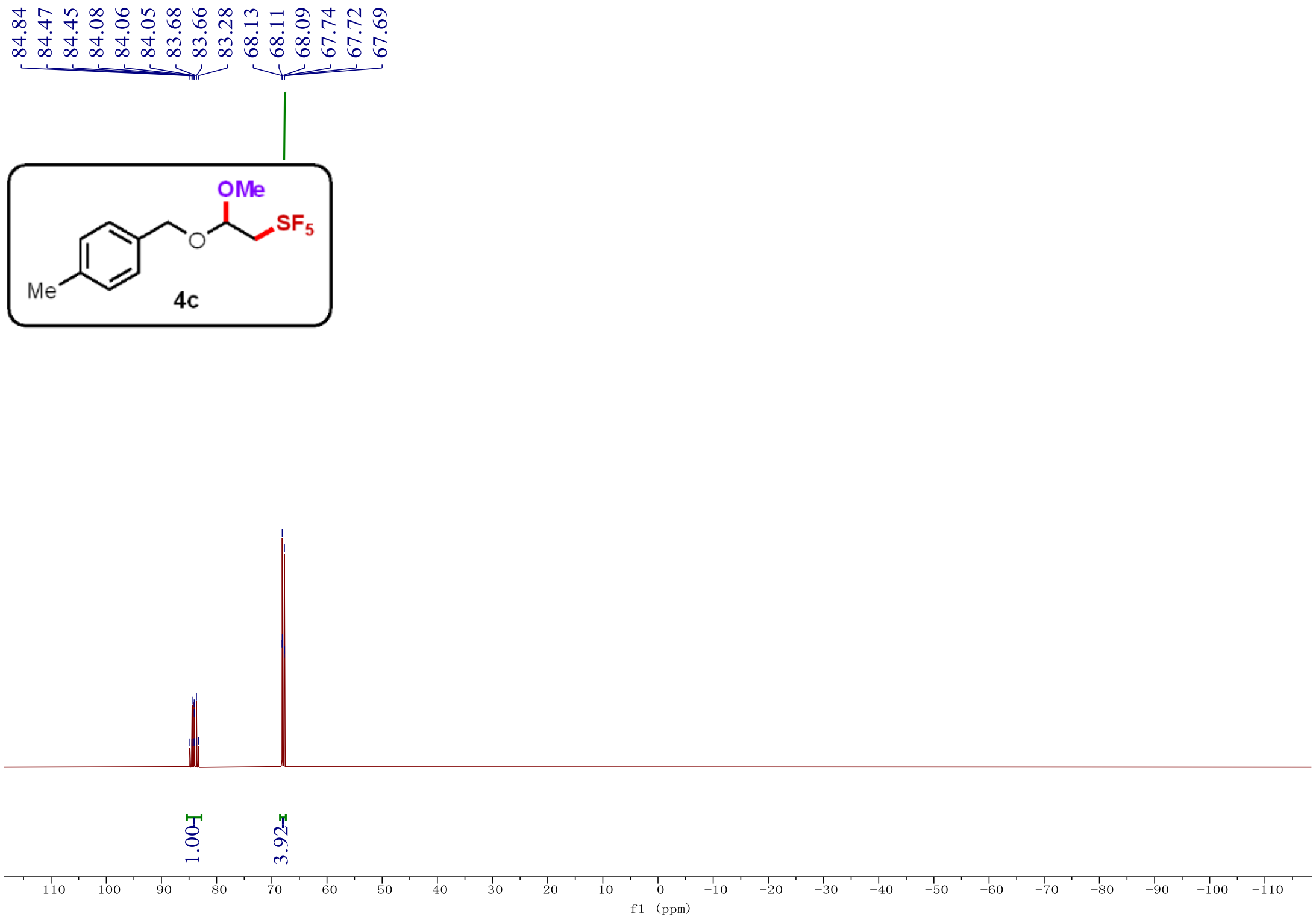

Supplementary Figure 99. ^{19}F NMR Spectrum of Compound 4c (376 MHz, $CDCl_3$, 25 ˚C)

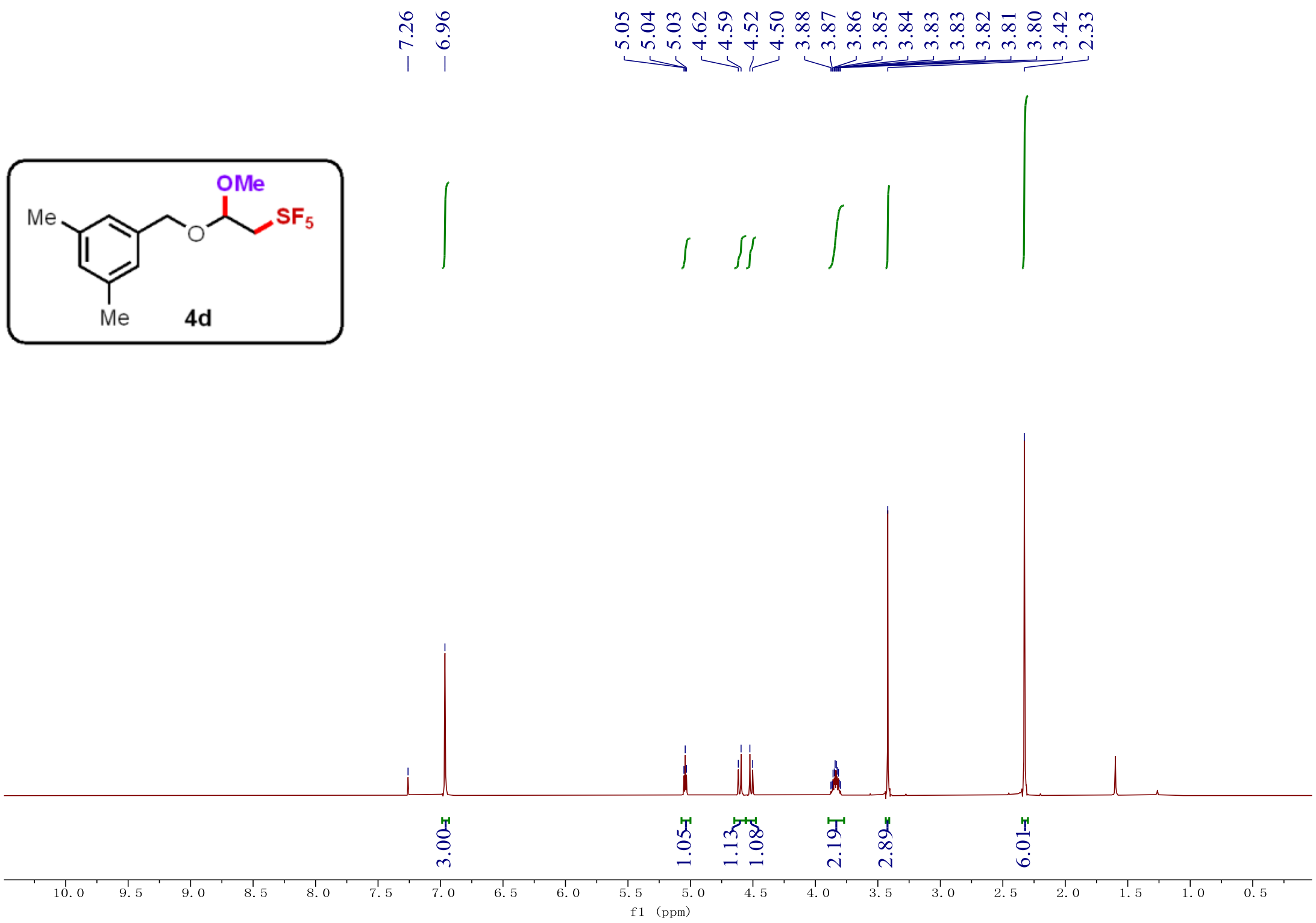

Supplementary Figure 100. ^{1}H NMR Spectrum of Compound 4d (500 MHz, $CDCl_3$, 25 ˚C)

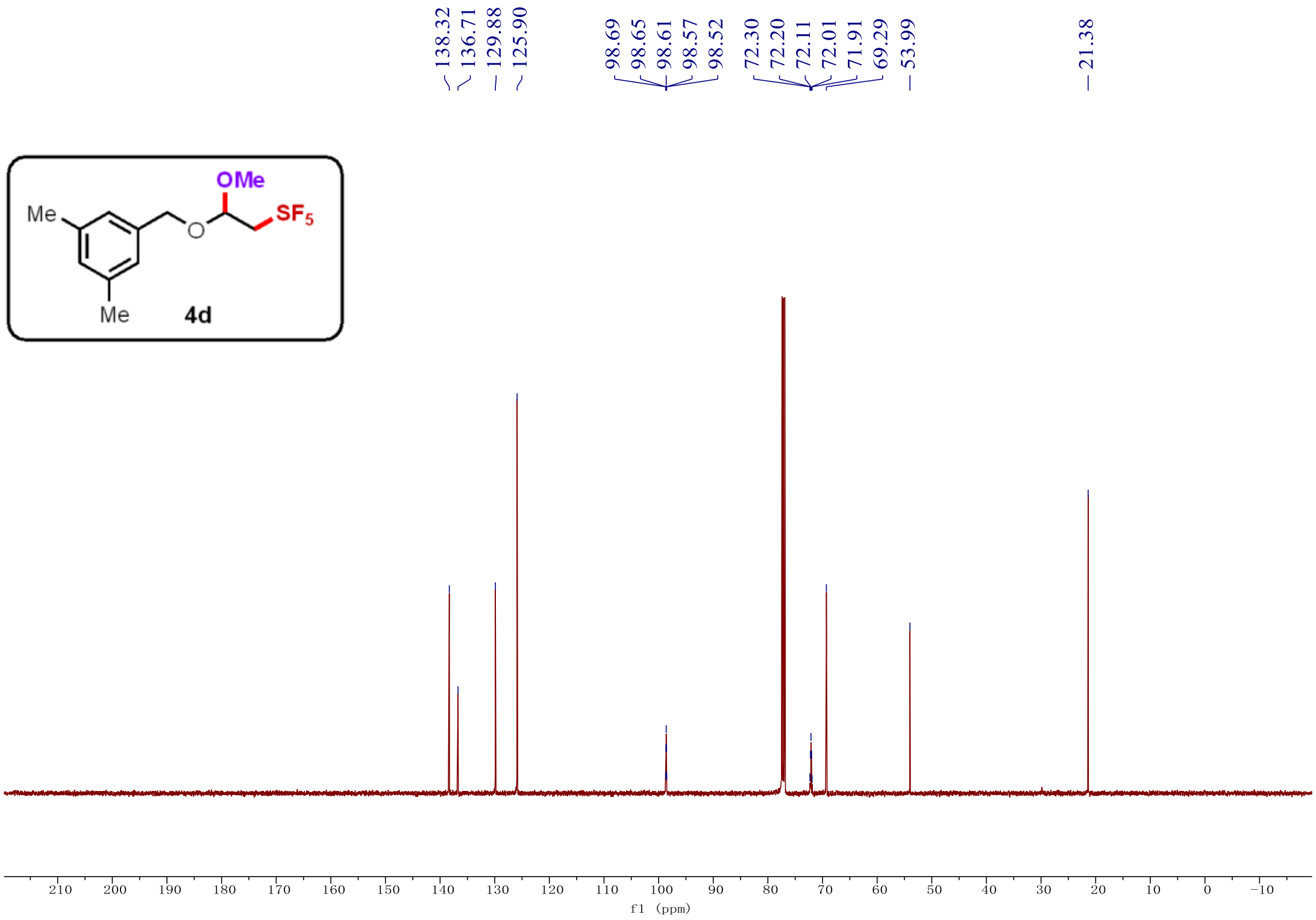

Supplementary Figure 101. ^{13}C **NMR Spectrum of Compound 4d (126 MHz,** $CDCl_3$**, 25 ˚C)**

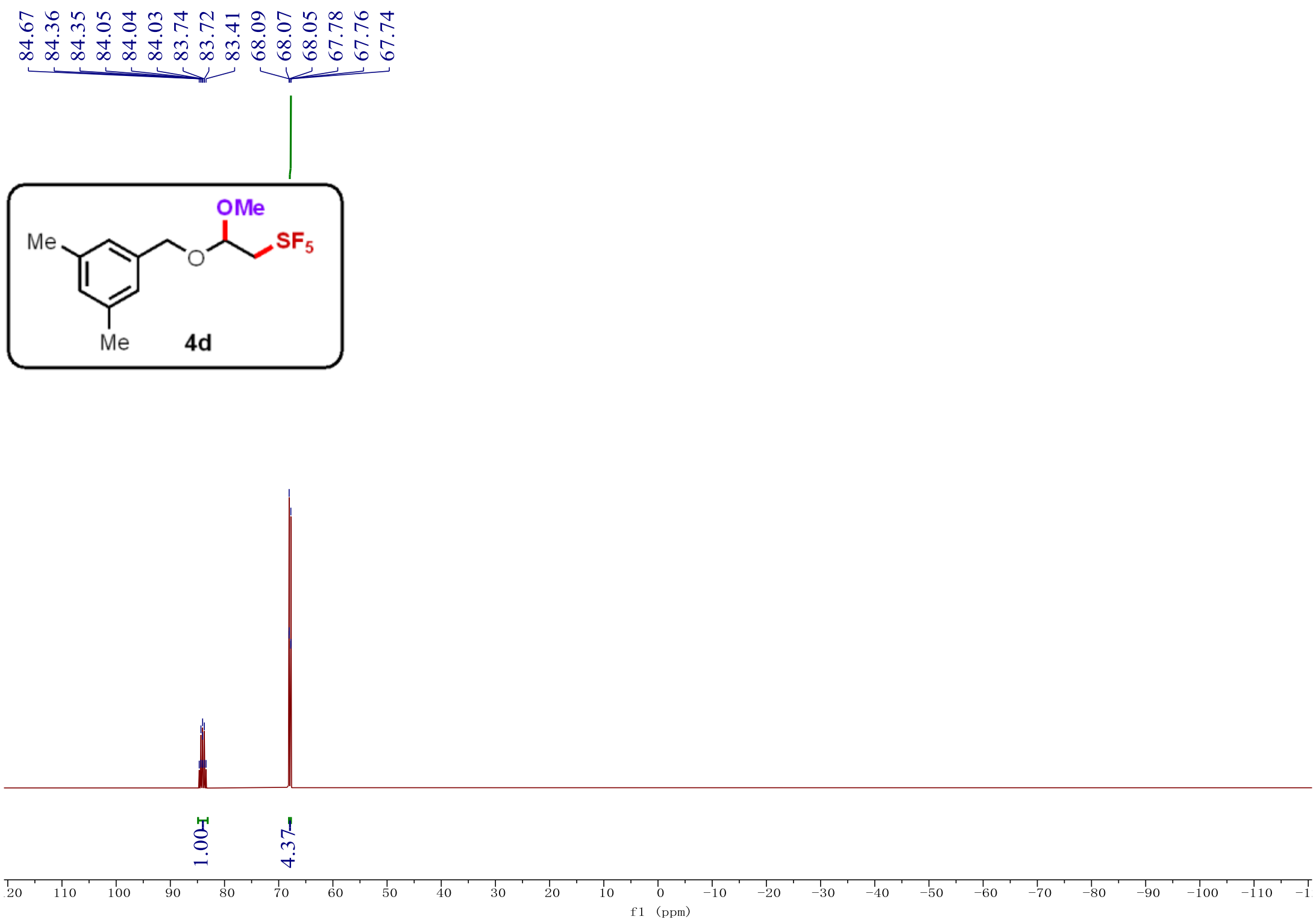

Supplementary Figure 102. ^{19}F **NMR Spectrum of Compound 4d (471 MHz,** $CDCl_3$**, 25 ˚C)**

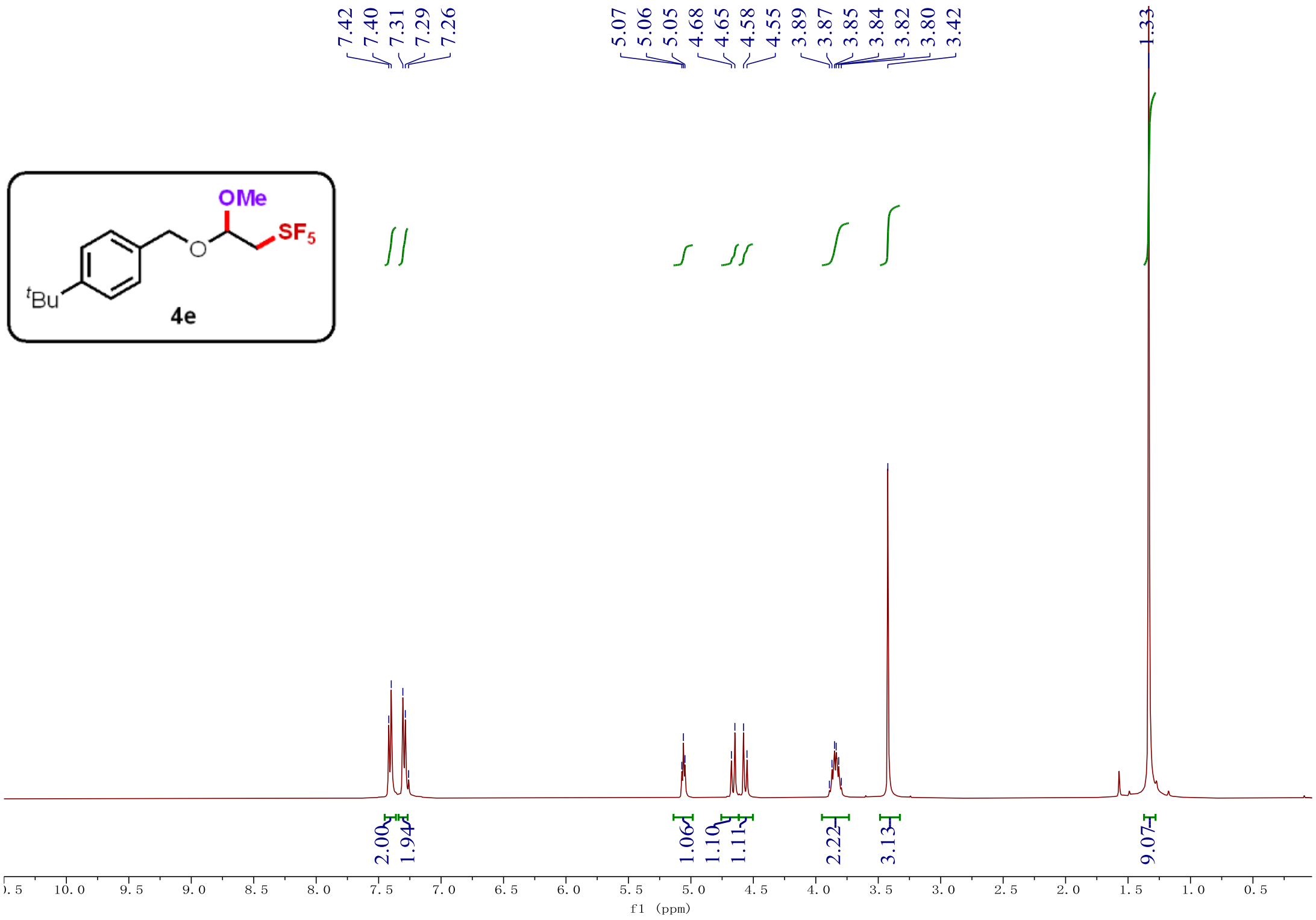

Supplementary Figure 103. ^{1}H NMR Spectrum of Compound 4e (400 MHz, $CDCl_3$, 25 ˚C)

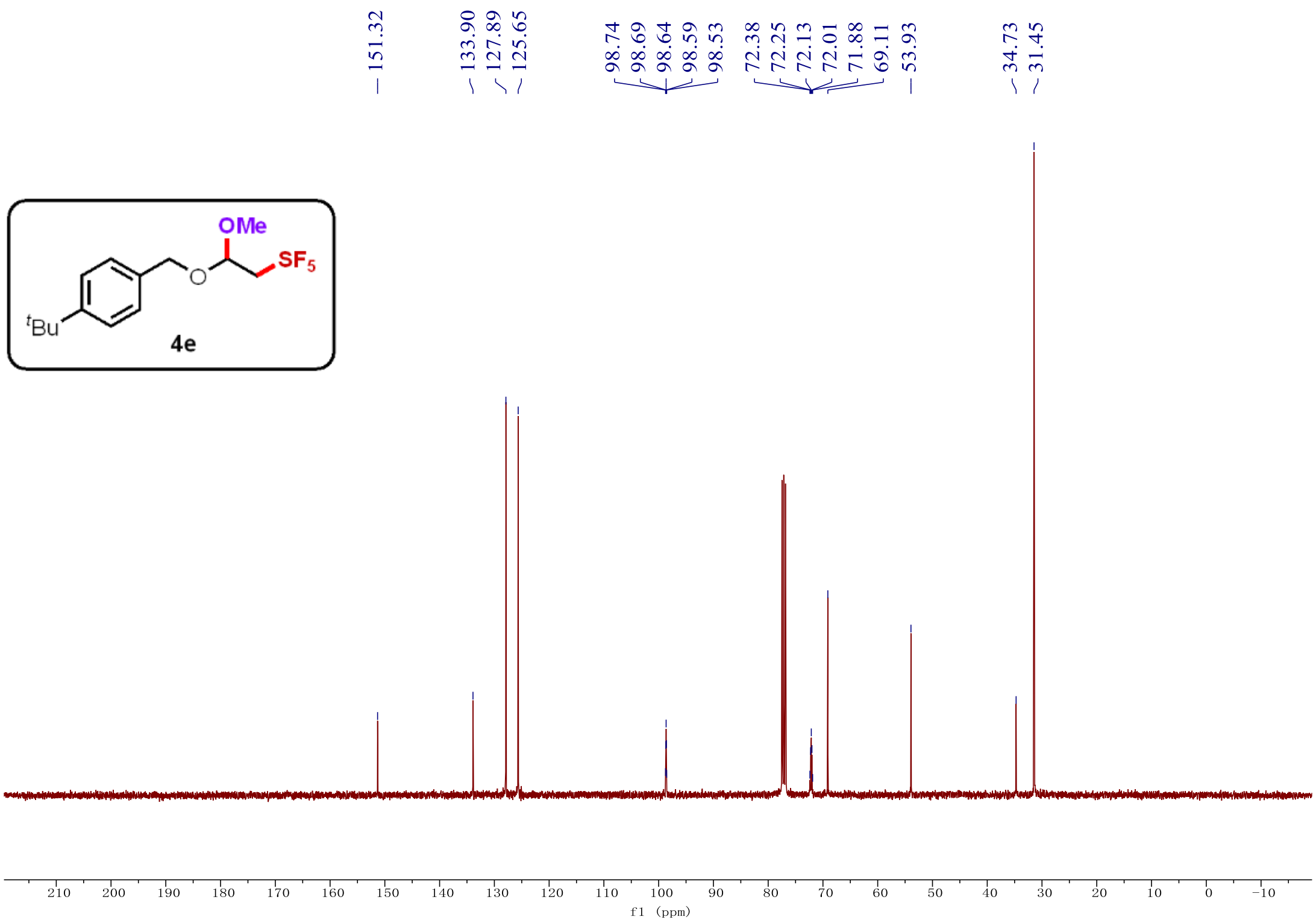

Supplementary Figure 104. ^{13}C NMR Spectrum of Compound 4e (101 MHz, $CDCl_3$, 25 ˚C)

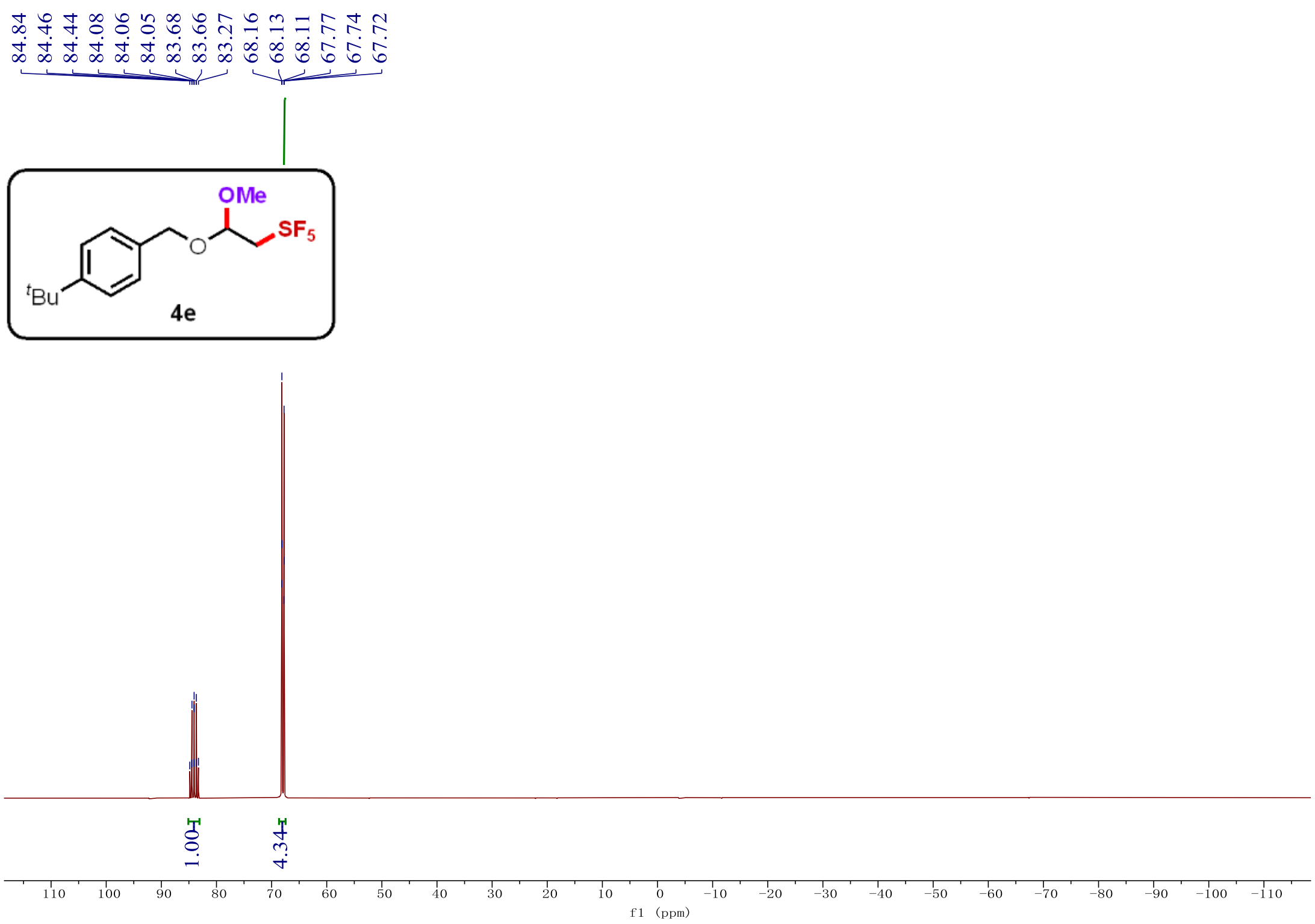

Supplementary Figure 105. ^{19}F NMR Spectrum of Compound 4e (376 MHz, $CDCl_3$, 25 ˚C)

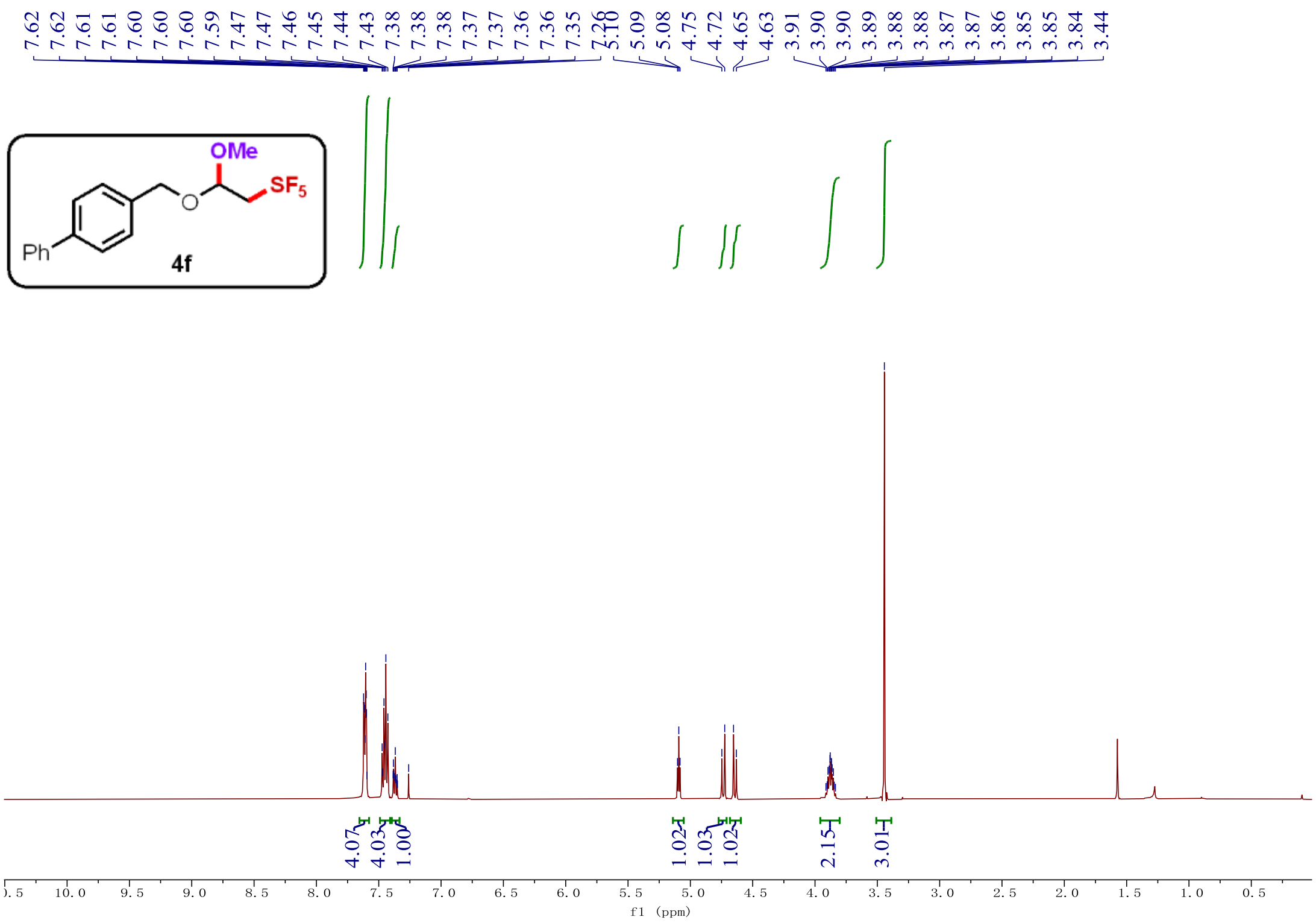

Supplementary Figure 106. ^{1}H NMR Spectrum of Compound 4f (500 MHz, $CDCl_3$, 25 ˚C)

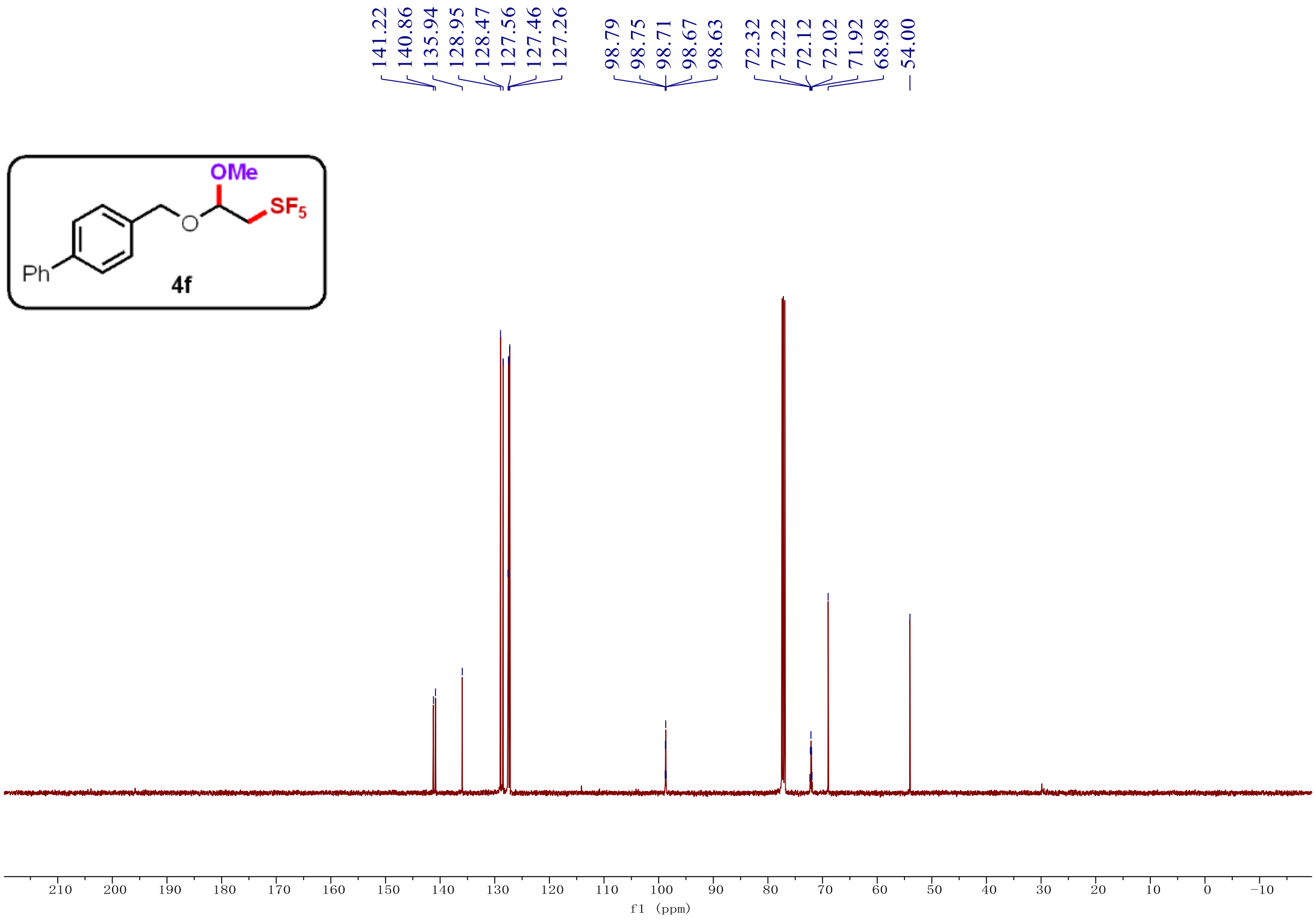

Supplementary Figure 107. ^{13}C **NMR Spectrum of Compound 4f (126 MHz, CDCl$_3$, 25 ˚C)**

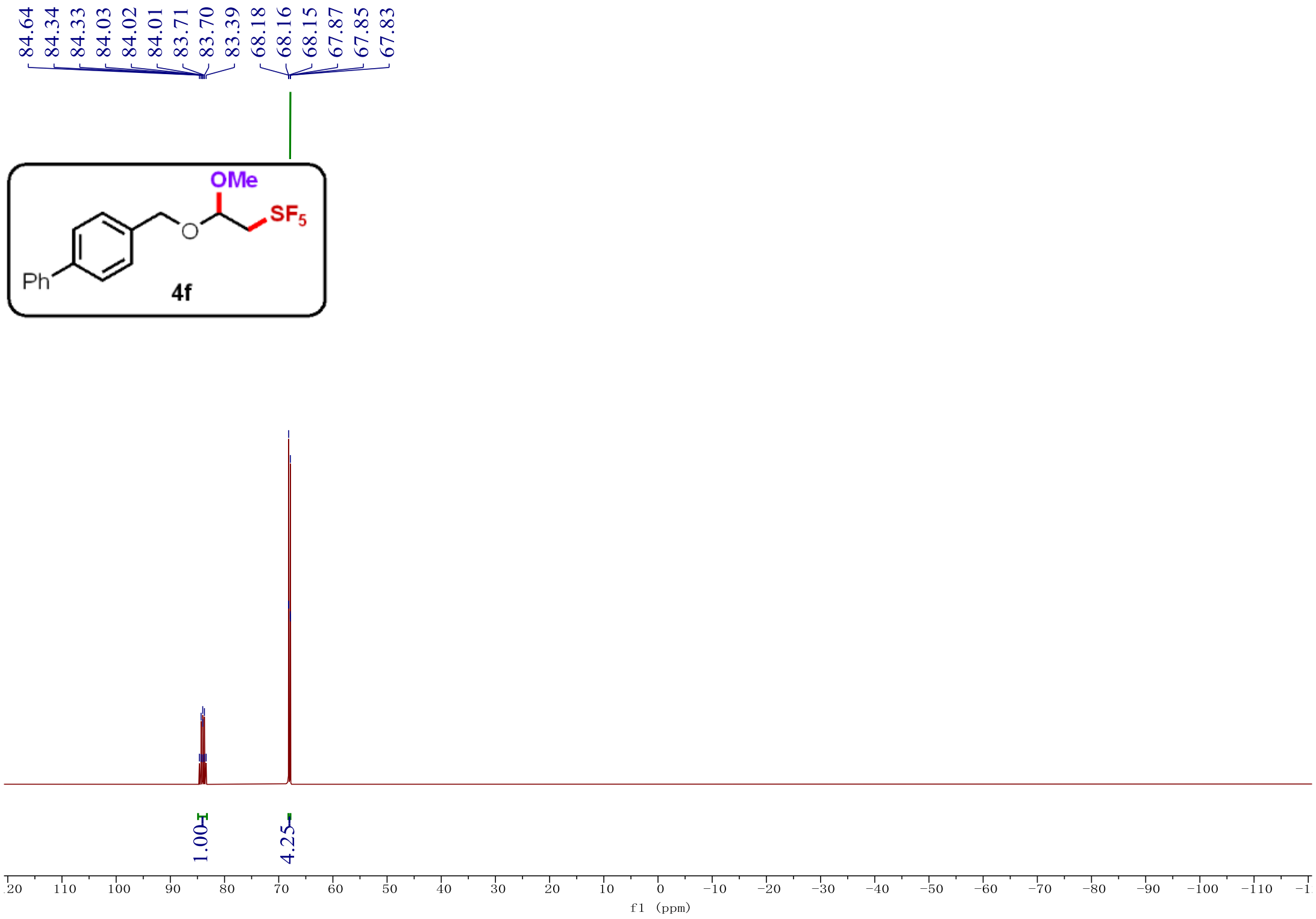

Supplementary Figure 108. ^{19}F **NMR Spectrum of Compound 4f (471 MHz, CDCl$_3$, 25 ˚C)**

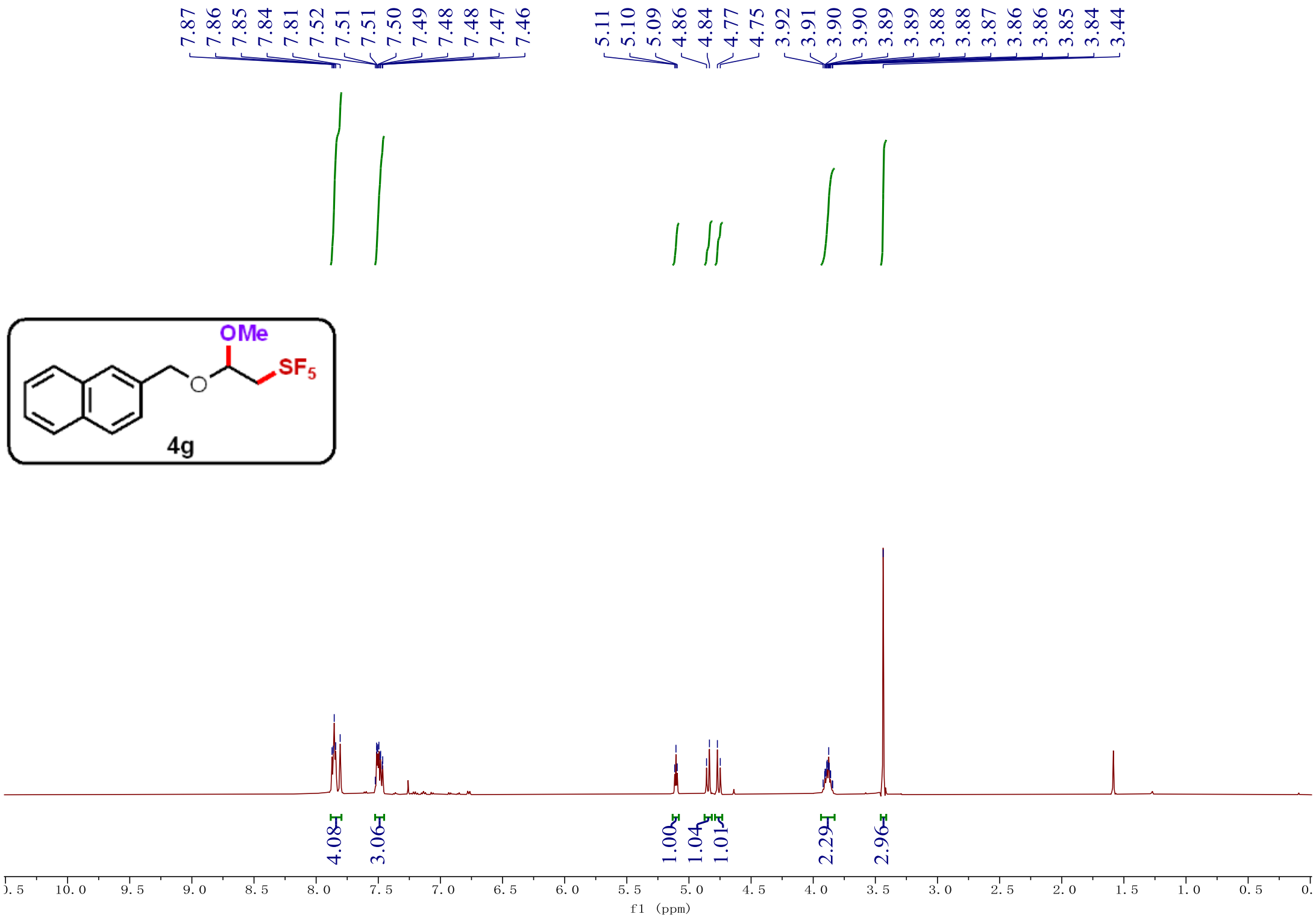

Supplementary Figure 109. ^{1}H NMR Spectrum of Compound 4g (500 MHz, $CDCl_3$, 25 °C)

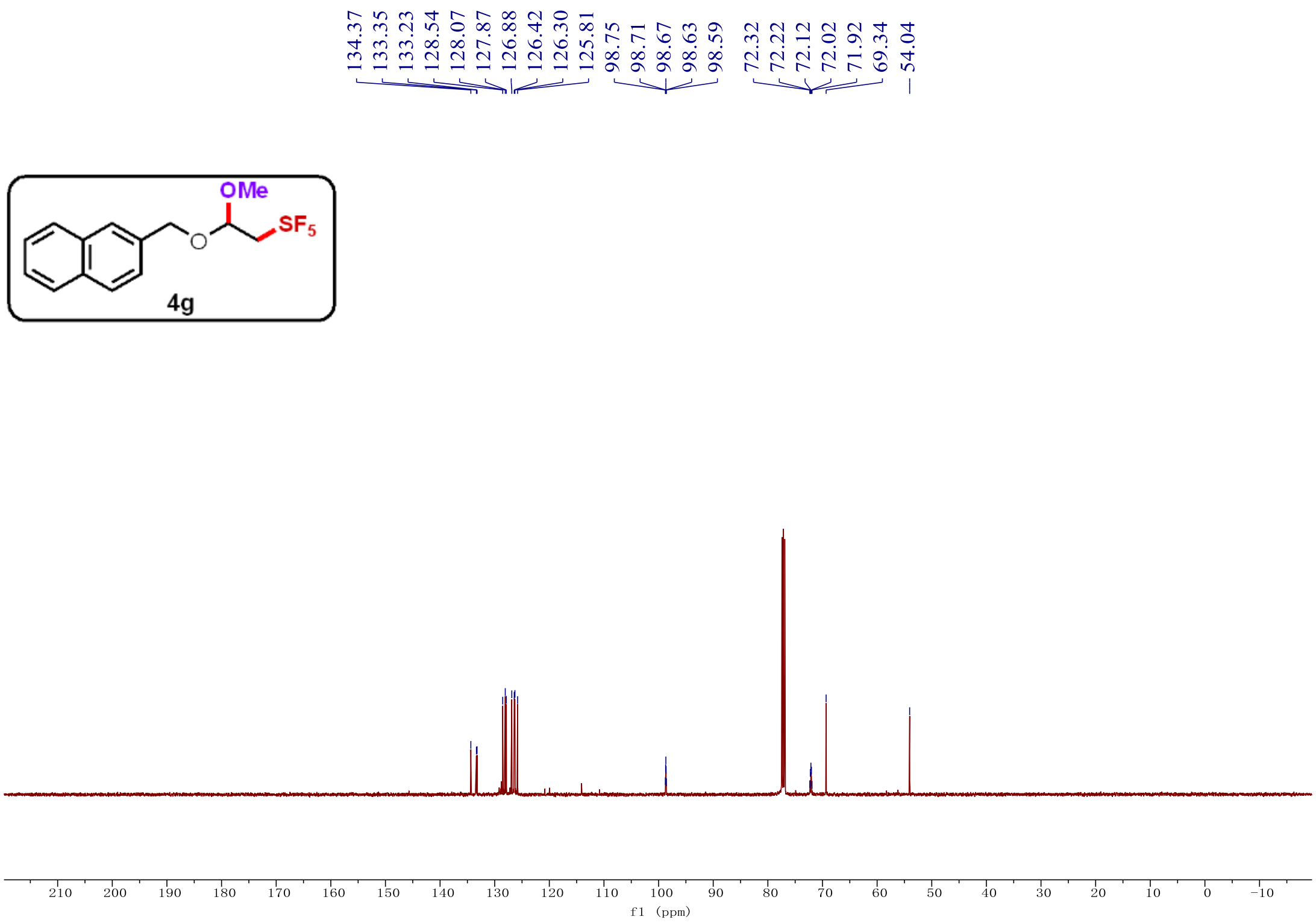

Supplementary Figure 110. ^{13}C NMR Spectrum of Compound 4g (126 MHz, $CDCl_3$, 25 °C)

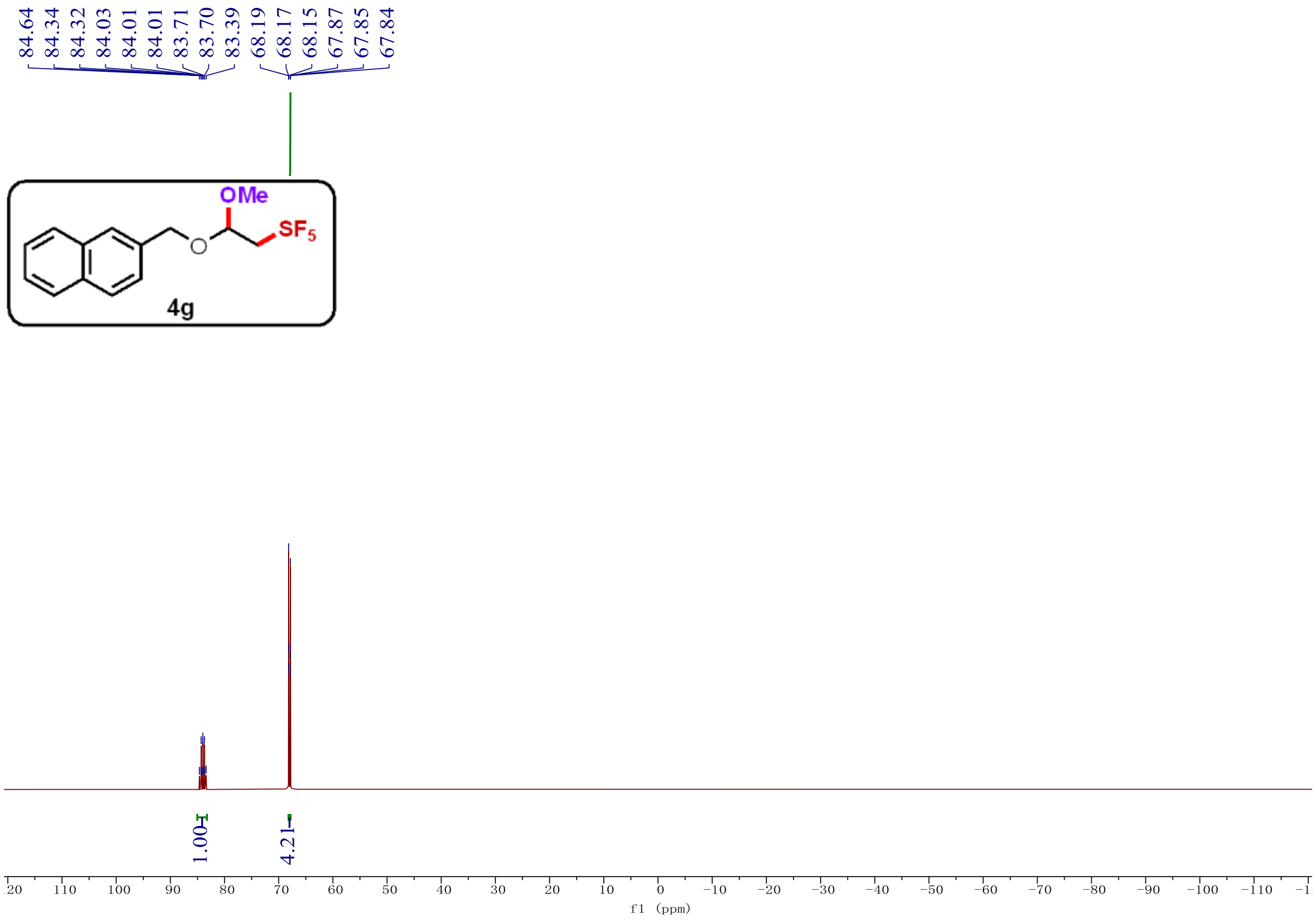

Supplementary Figure 111. ^{19}F NMR Spectrum of Compound 4g (471 MHz, $CDCl_3$, 25 ˚C)

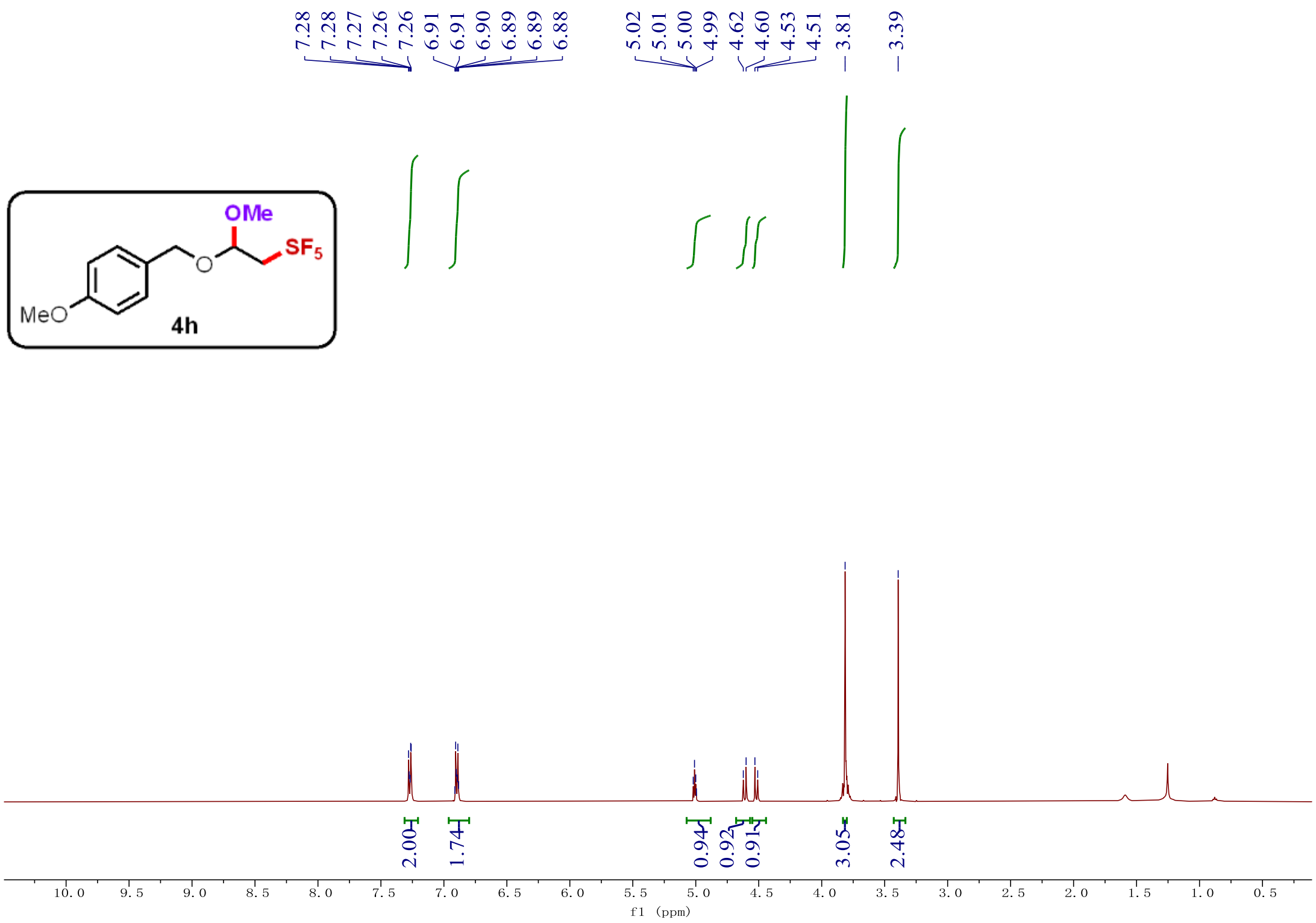

Supplementary Figure 112. ^{1}H NMR Spectrum of Compound 4h (500 MHz, $CDCl_3$, 25 ˚C)

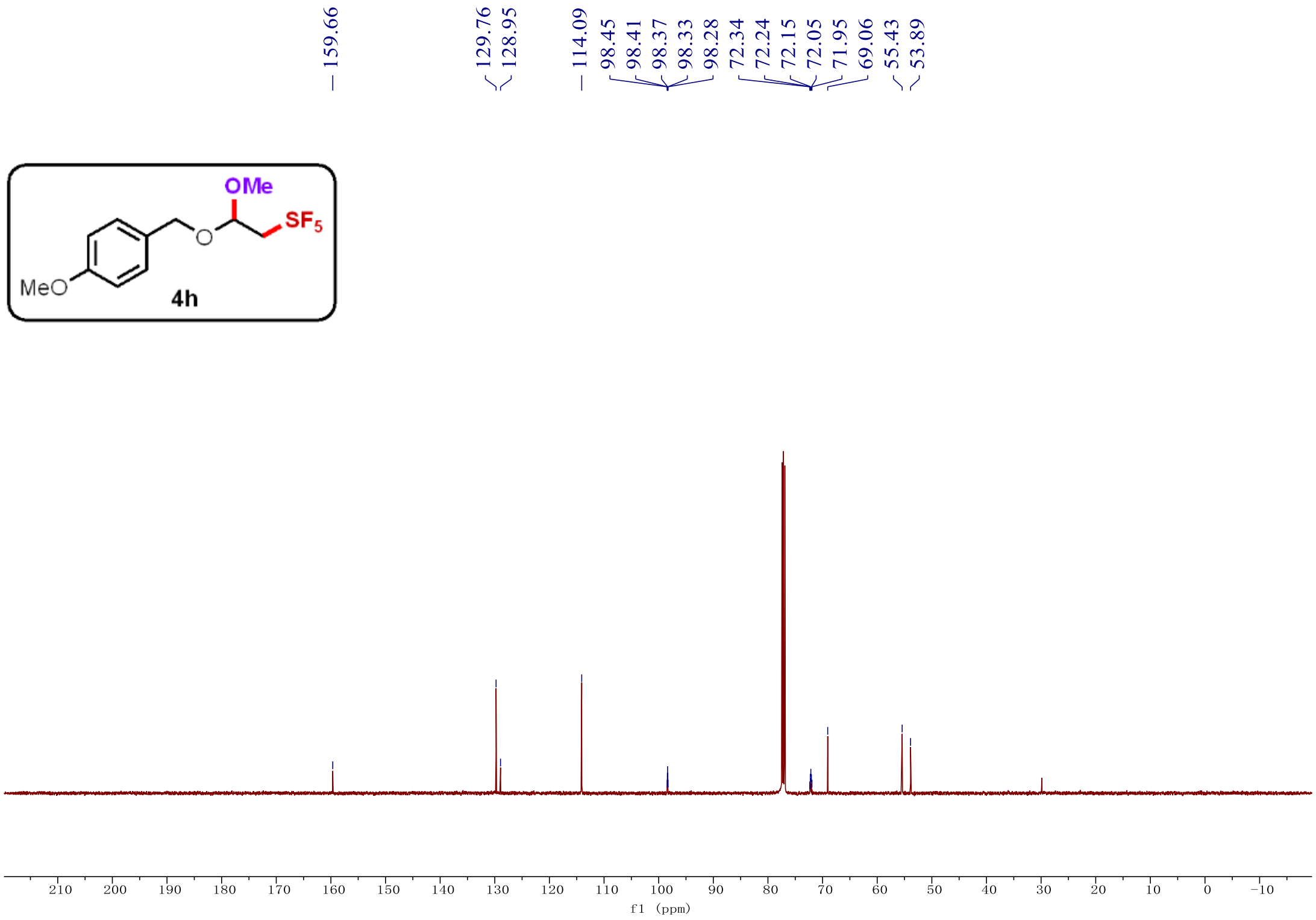

Supplementary Figure 113. ^{13}C NMR Spectrum of Compound 4h (126 MHz, $CDCl_3$, 25 ˚C)

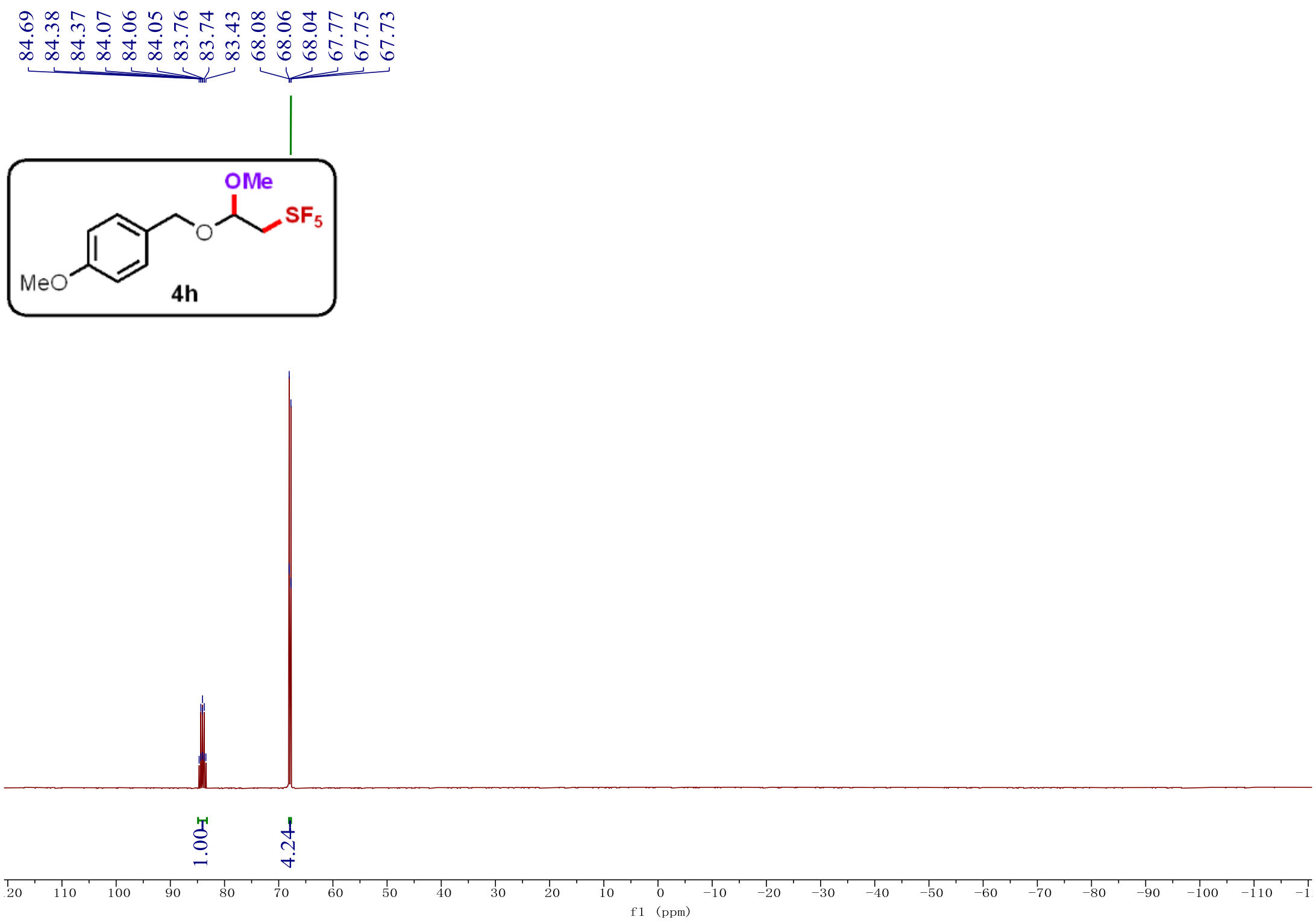

Supplementary Figure 114. ^{19}F NMR Spectrum of Compound 4h (471 MHz, $CDCl_3$, 25 ˚C)

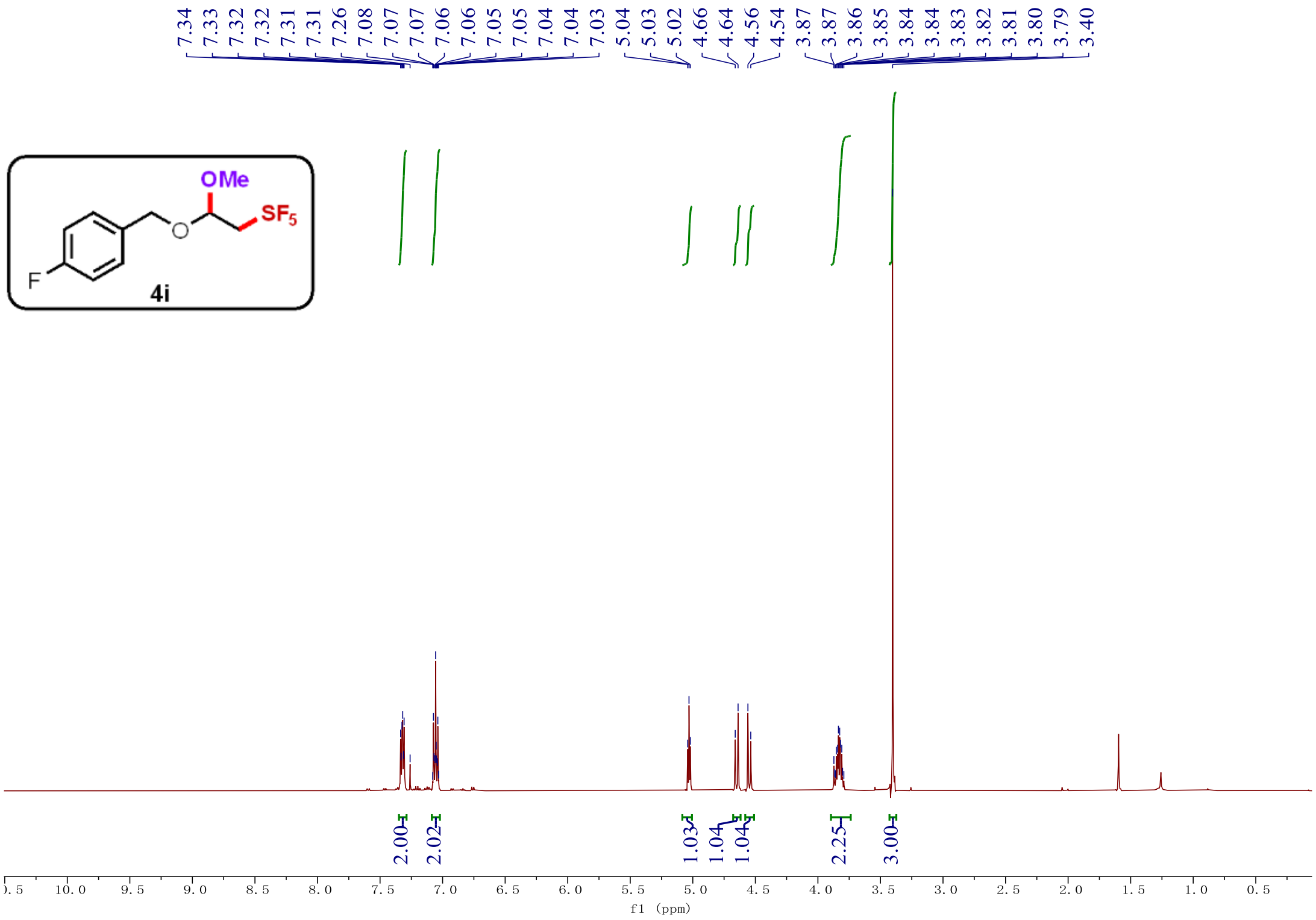

Supplementary Figure 115. ^{1}**H NMR Spectrum of Compound 4i (500 MHz, CDCl$_3$, 25 ˚C)**

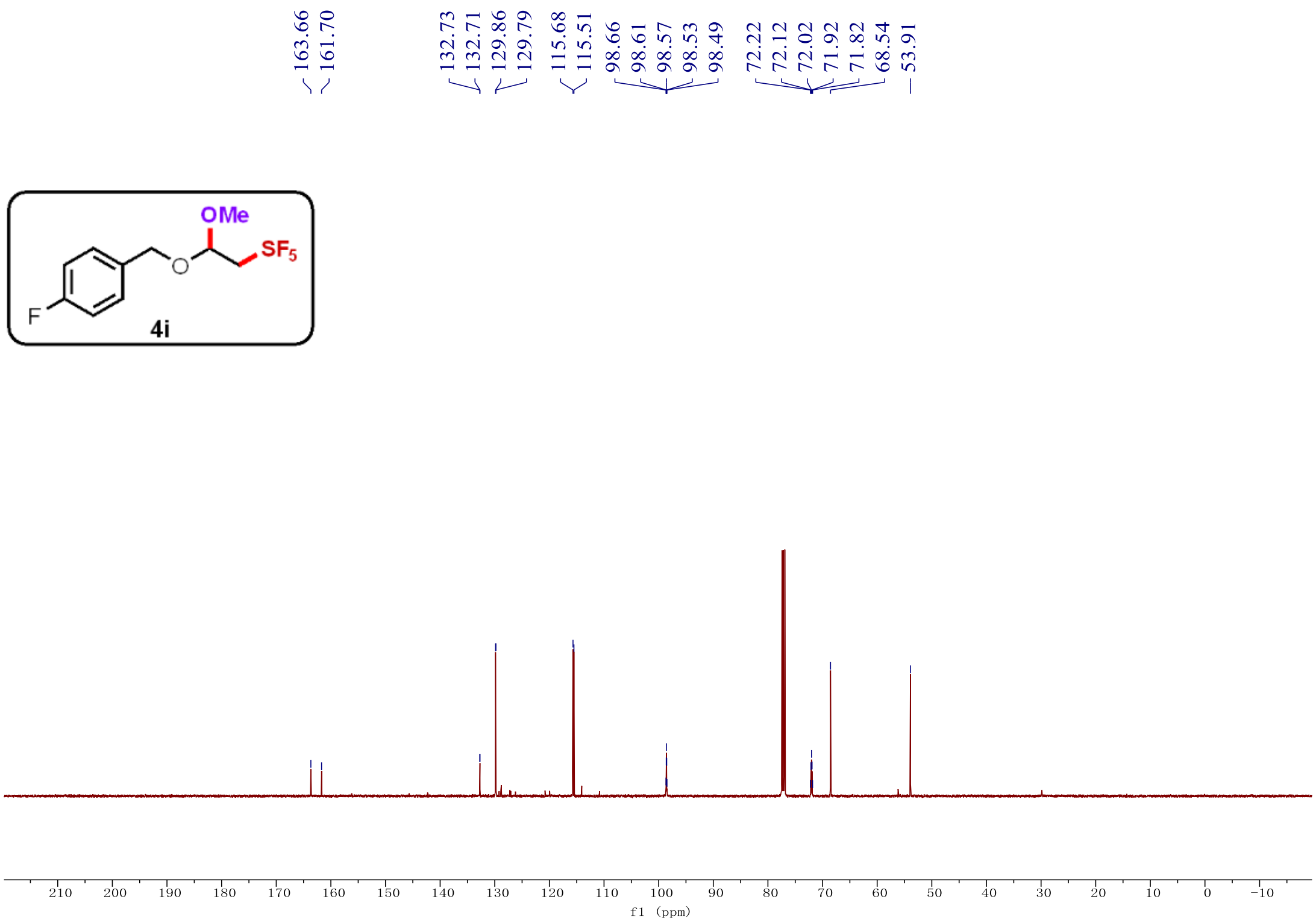

Supplementary Figure 116. ^{13}**C NMR Spectrum of Compound 4i (126 MHz, CDCl$_3$, 25 ˚C)**

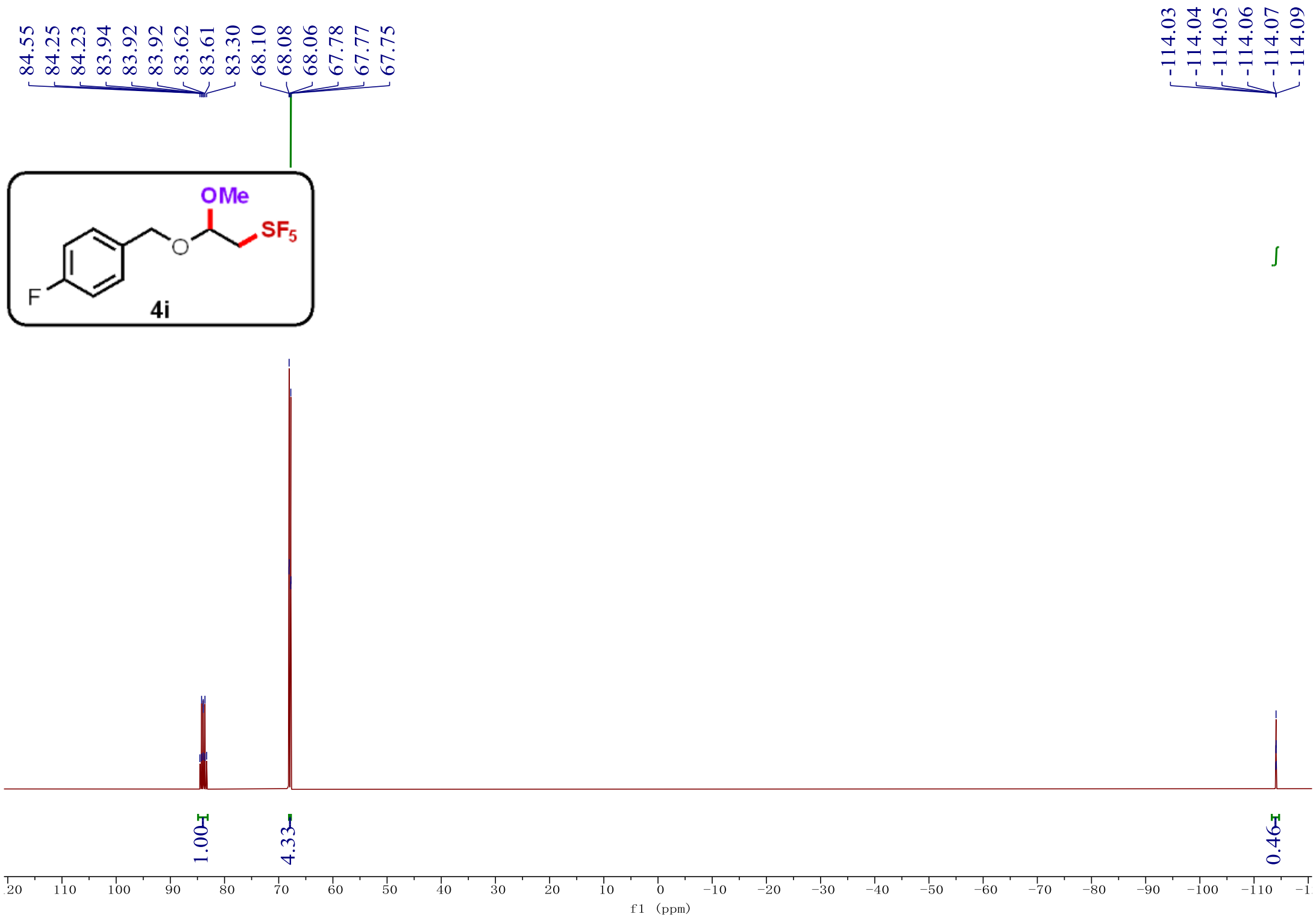

Supplementary Figure 117. ^{19}F NMR Spectrum of Compound 4i (471 MHz, $CDCl_3$, 25 ˚C)

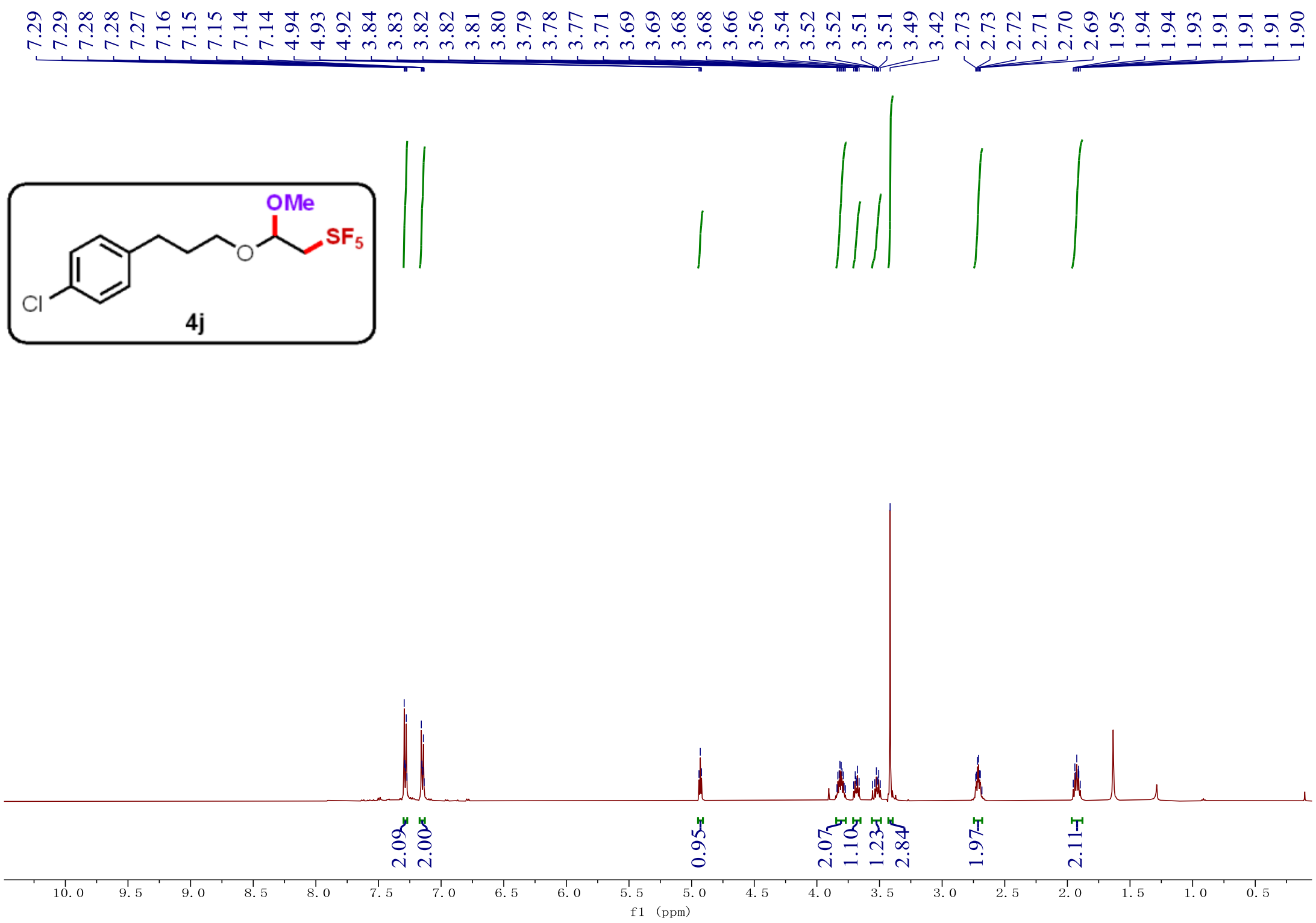

Supplementary Figure 118. ^{1}H NMR Spectrum of Compound 4j (500 MHz, $CDCl_3$, 25 ˚C)

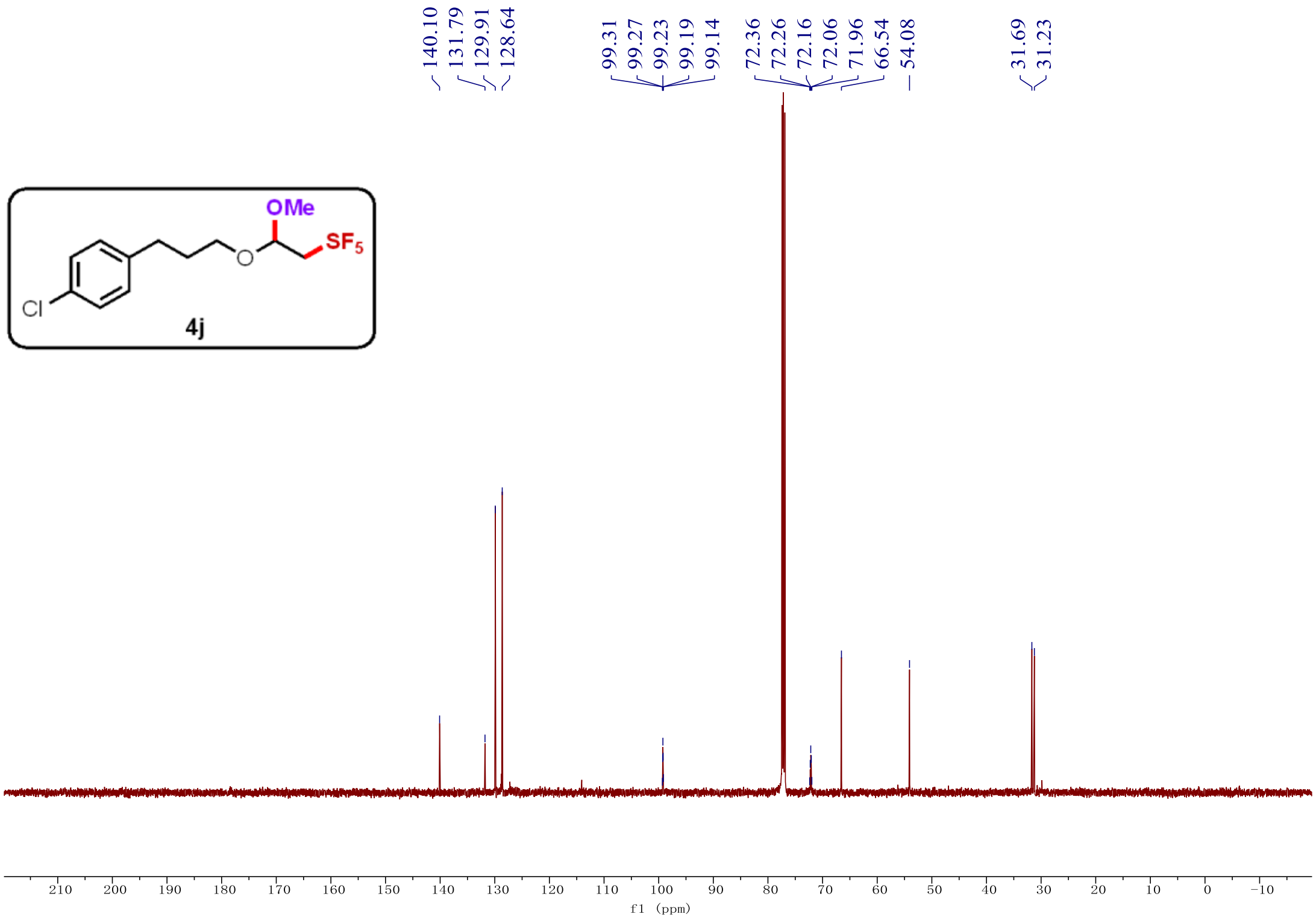

Supplementary Figure 119. **^{13}C NMR Spectrum of Compound 4j (126 MHz, $CDCl_3$, 25 ˚C)**

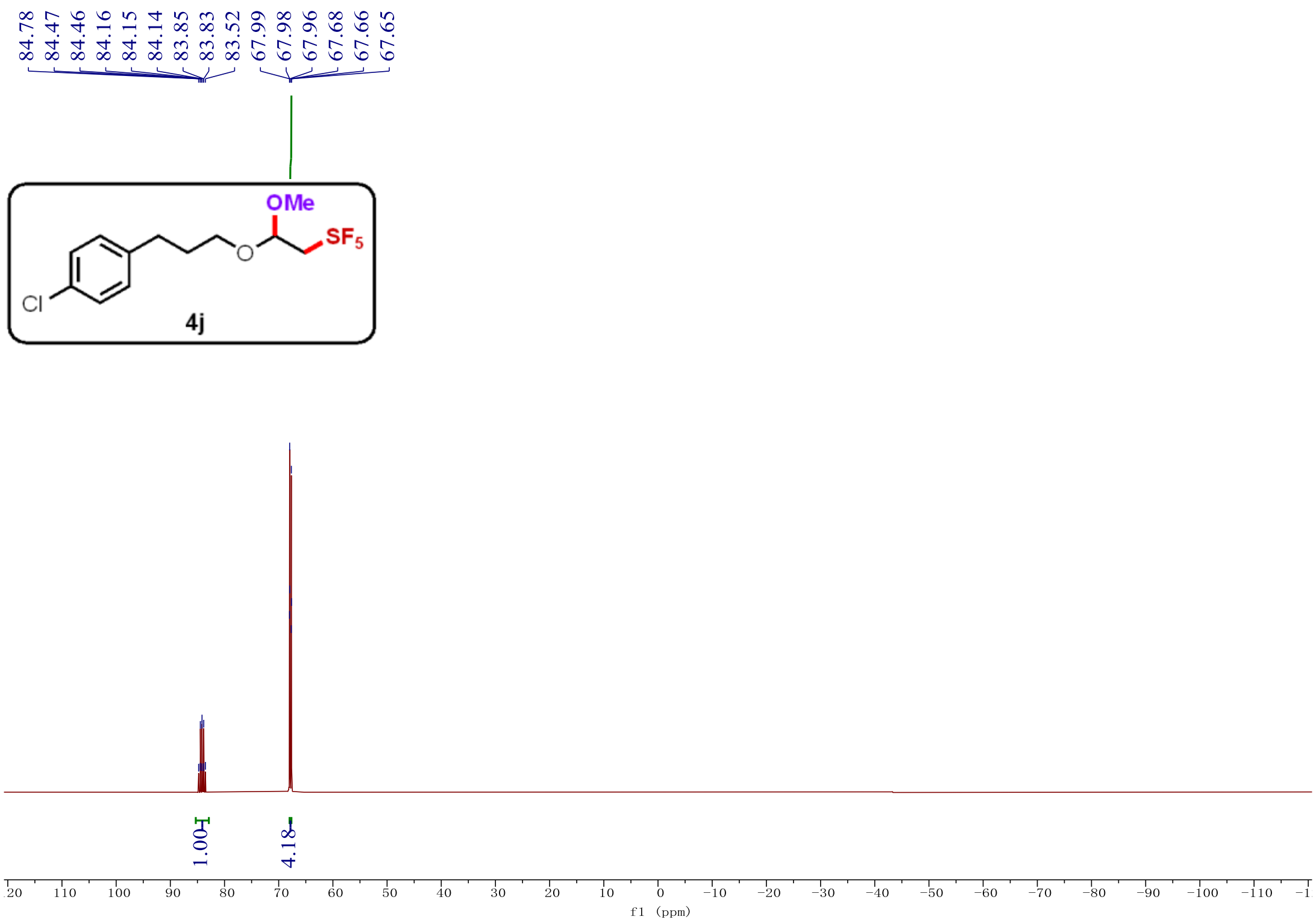

Supplementary Figure 120. **^{19}F NMR Spectrum of Compound 4j (471 MHz, $CDCl_3$, 25 ˚C)**

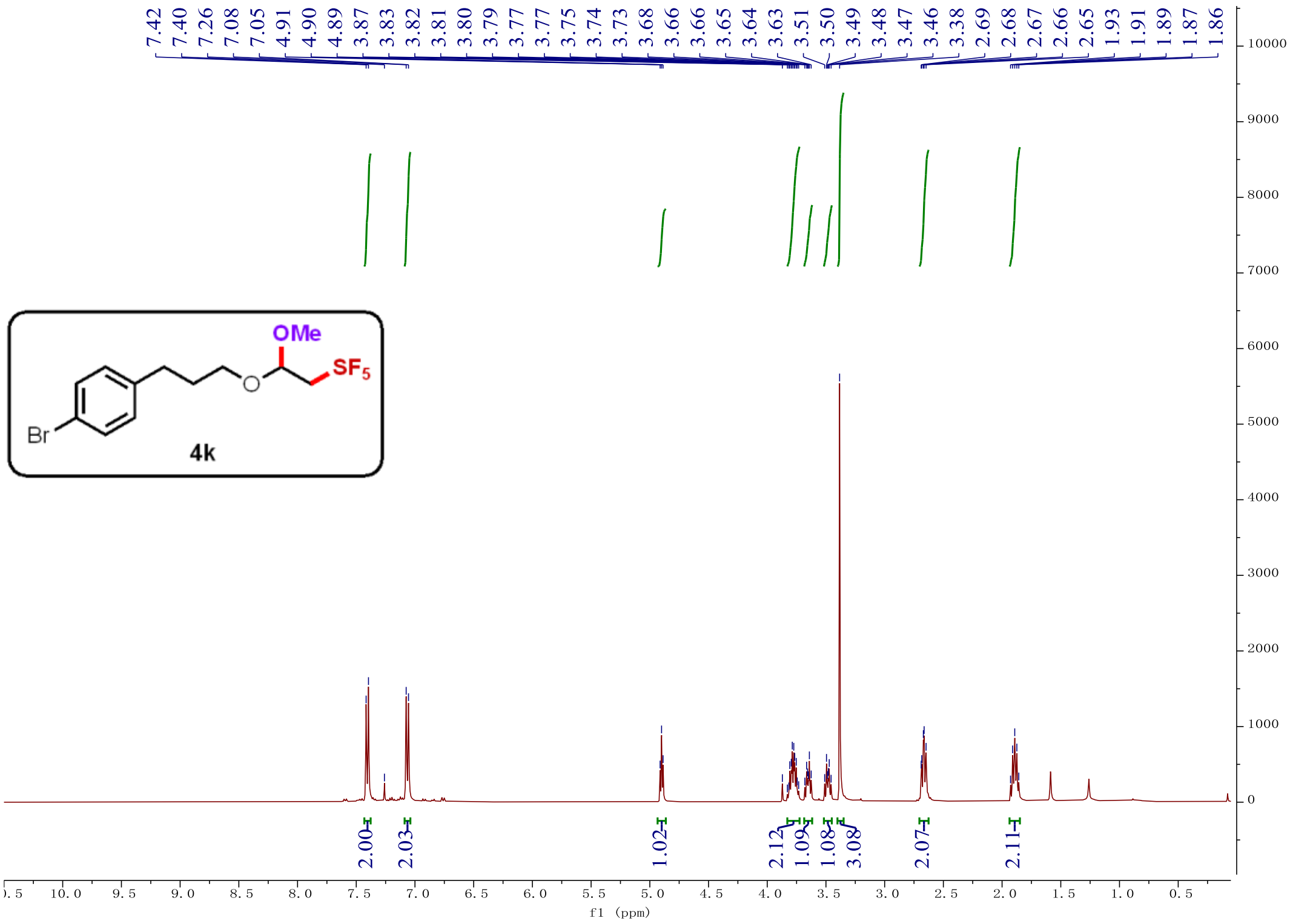

Supplementary Figure 121. ^{1}H NMR Spectrum of Compound 4k (400 MHz, $CDCl_3$, 25 ˚C)

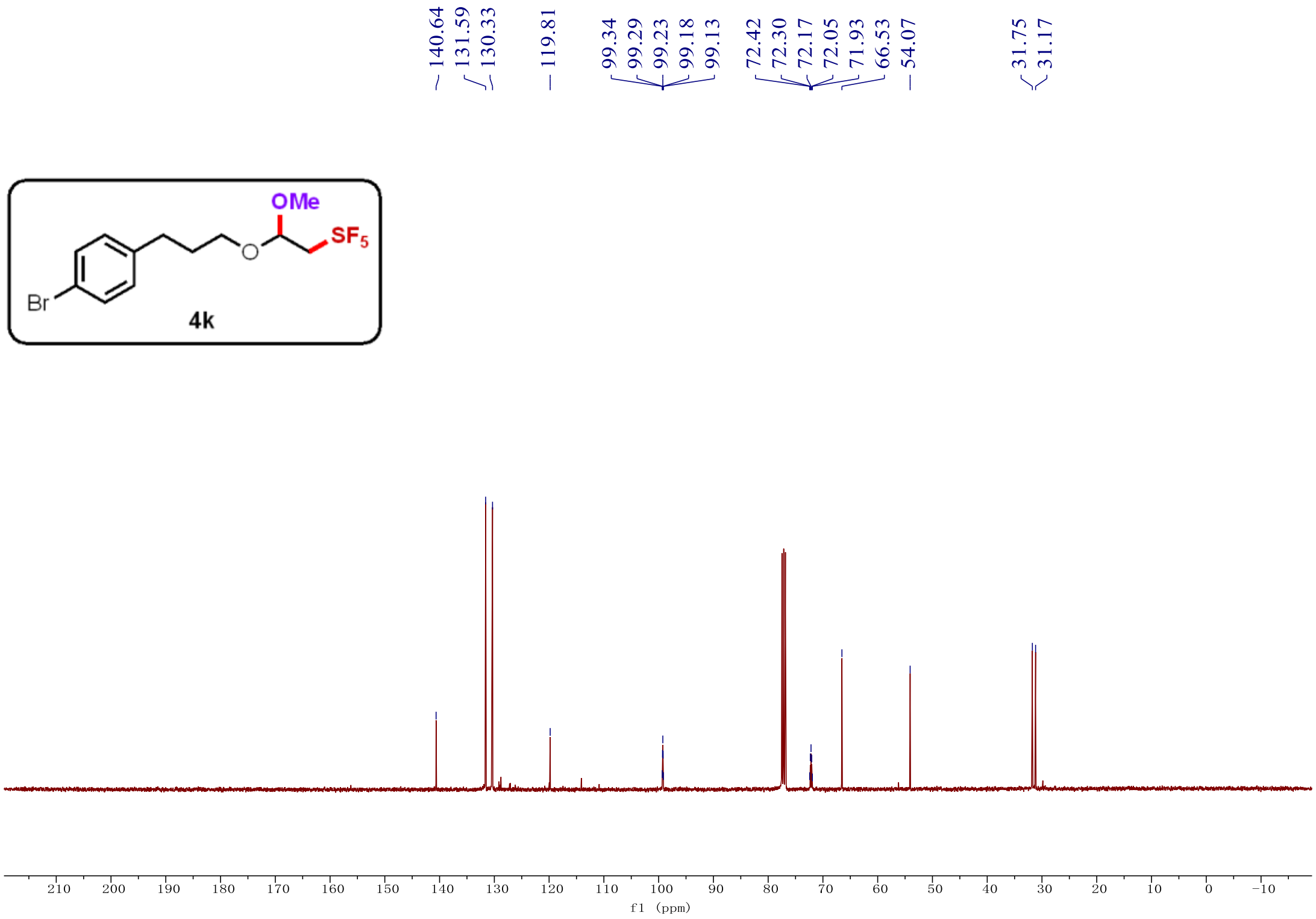

Supplementary Figure 122. ^{13}C NMR Spectrum of Compound 4k (101 MHz, $CDCl_3$, 25 ˚C)

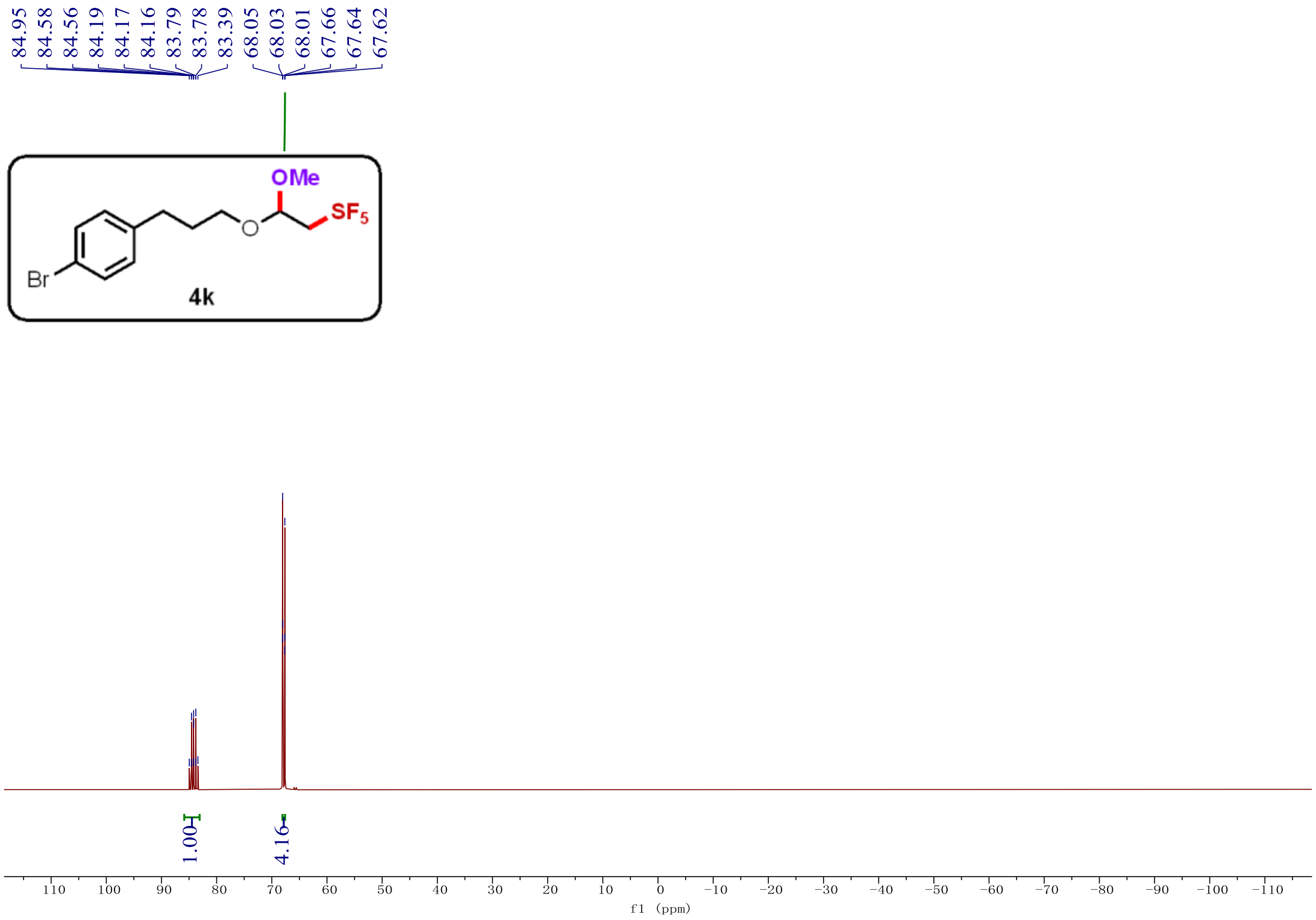

Supplementary Figure 123. ^{19}F NMR Spectrum of Compound 4k (376 MHz, $CDCl_3$, 25 ˚C)

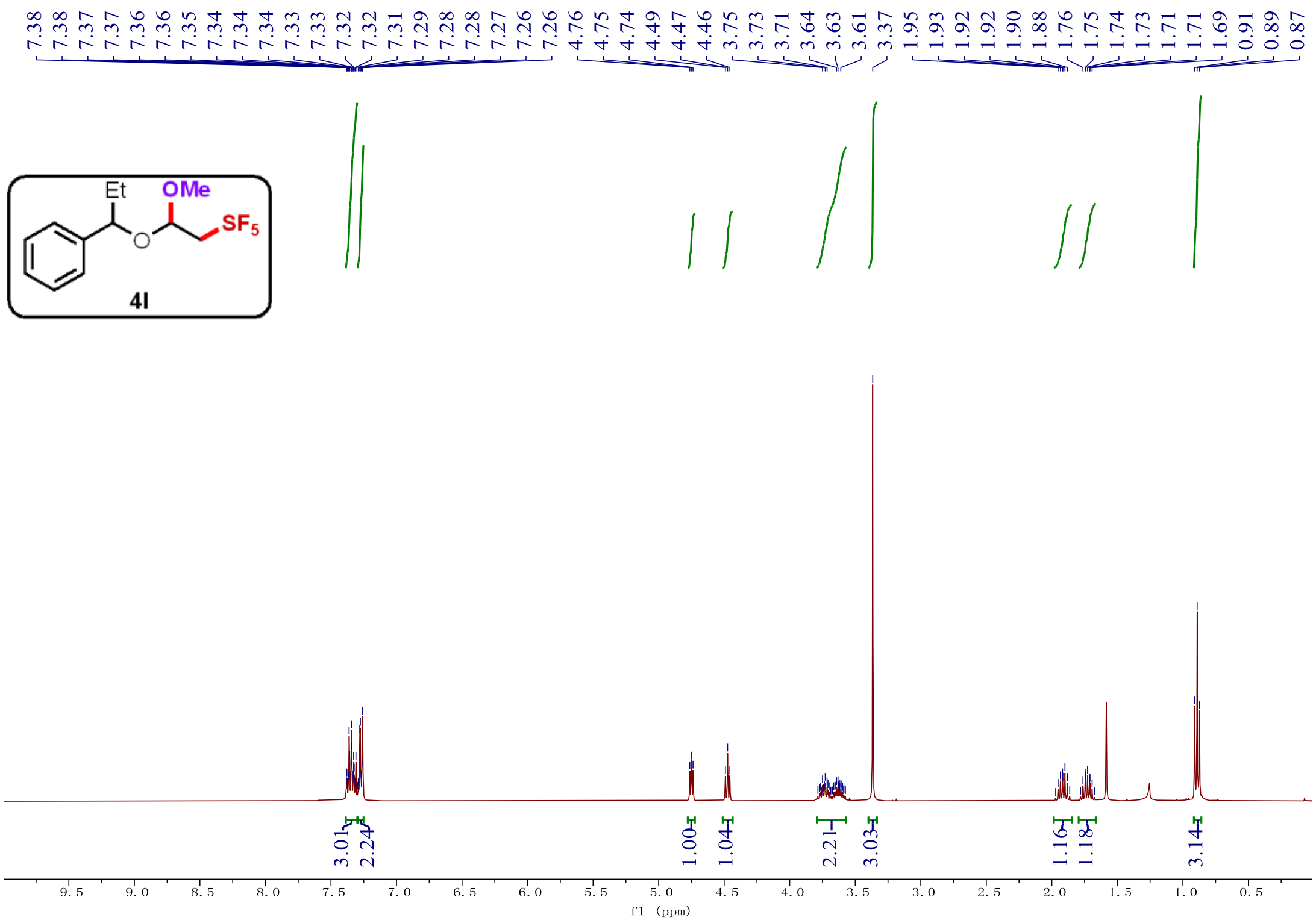

Supplementary Figure 124. ^{1}H NMR Spectrum of Compound 4l (400 MHz, $CDCl_3$, 25 ˚C)

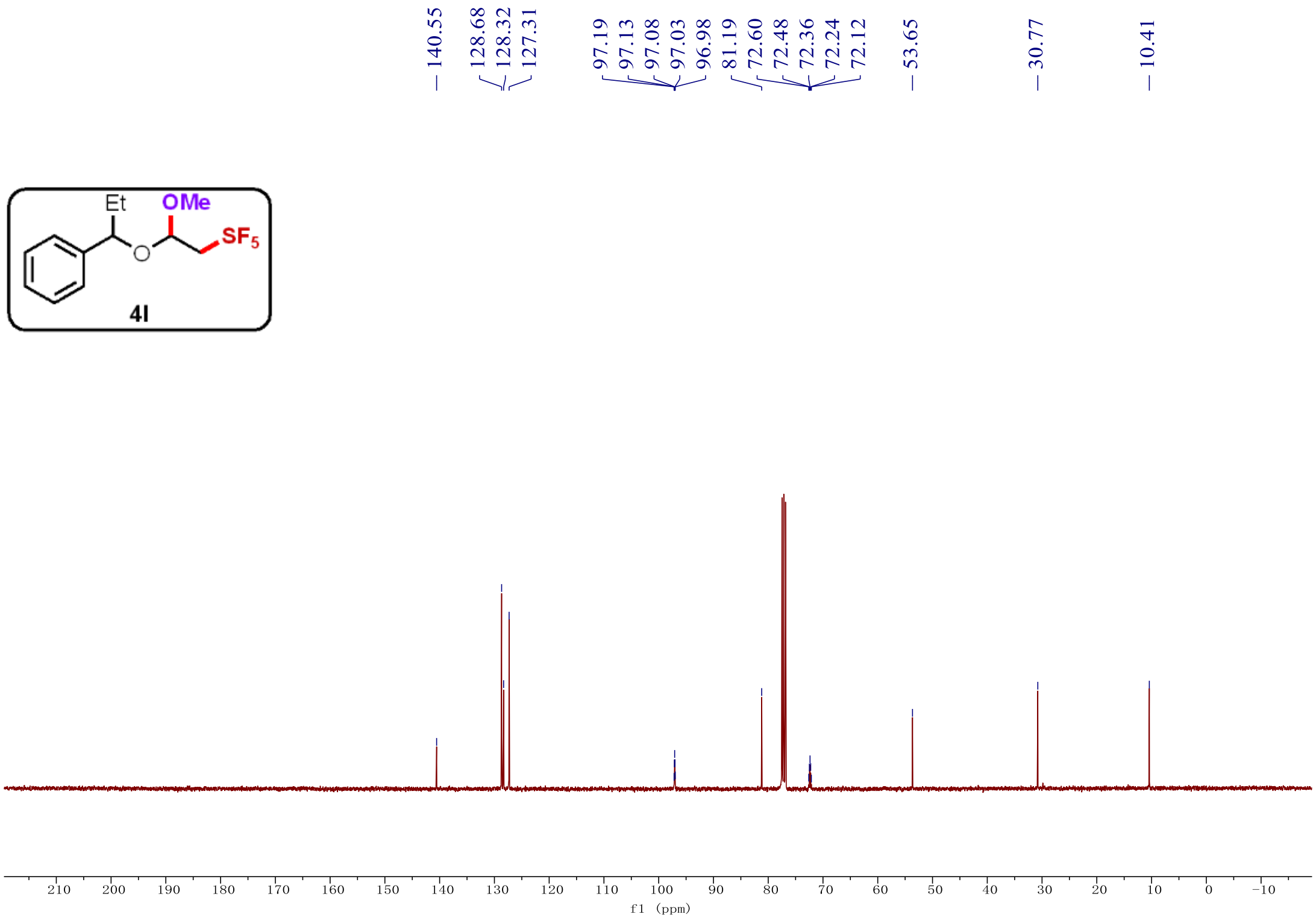

Supplementary Figure 125. ^{13}C **NMR Spectrum of Compound 4l (101 MHz, $CDCl_3$, 25 ˚C)**

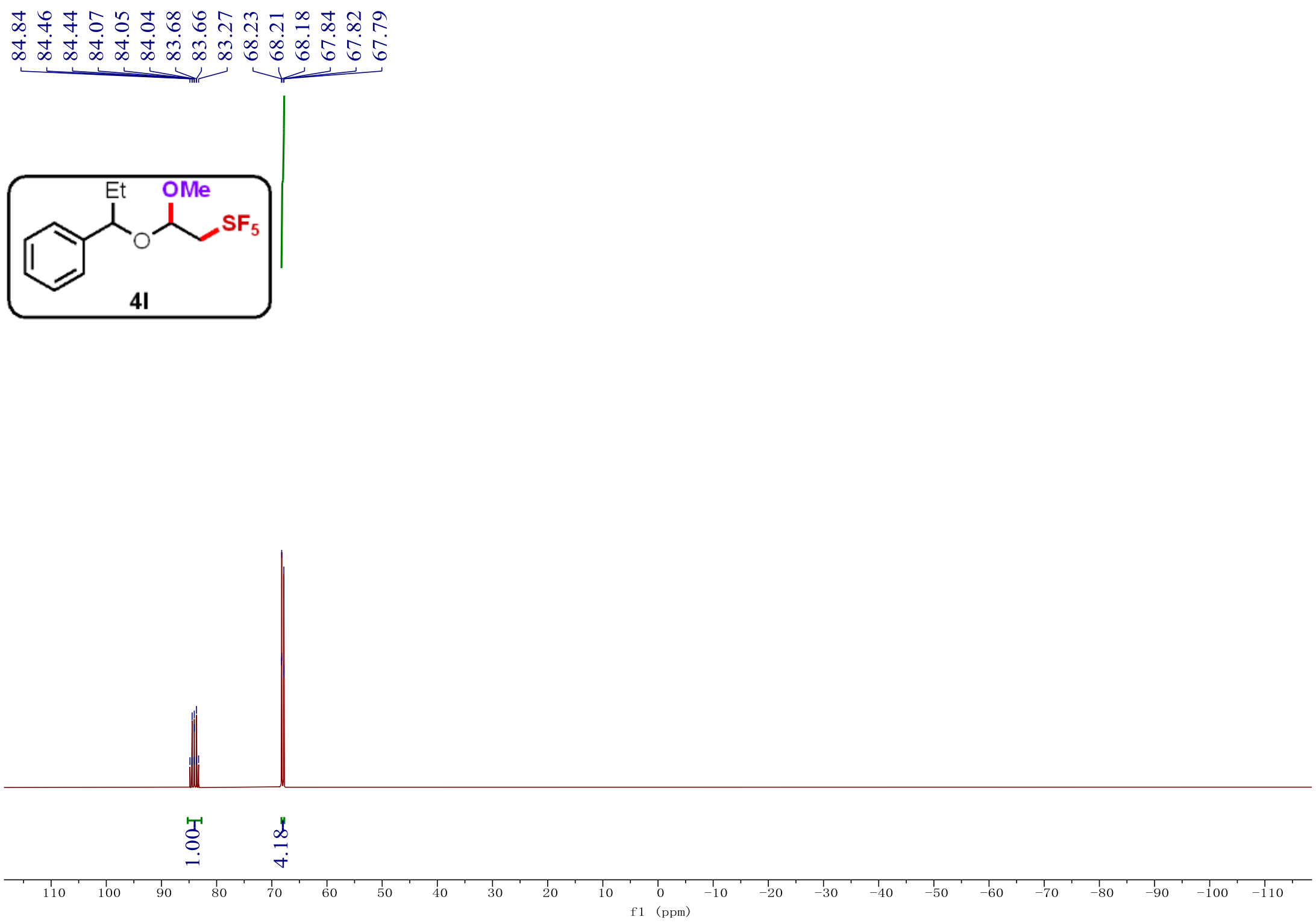

Supplementary Figure 126. ^{19}F **NMR Spectrum of Compound 4l (376 MHz, $CDCl_3$, 25 ˚C)**

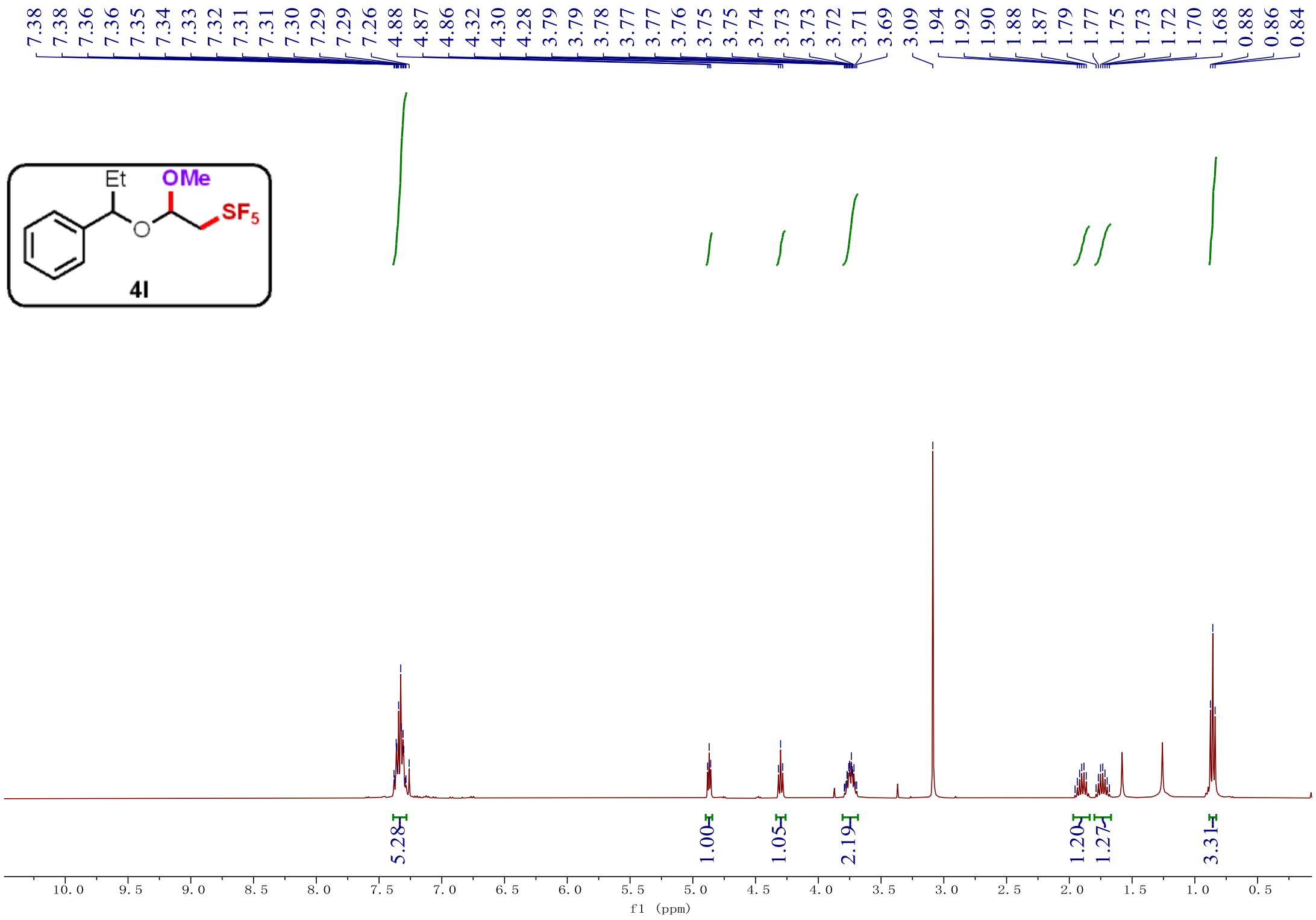

Supplementary Figure 127. ^{1}H NMR Spectrum of Compound 4l (400 MHz, $CDCl_3$, 25 ˚C)

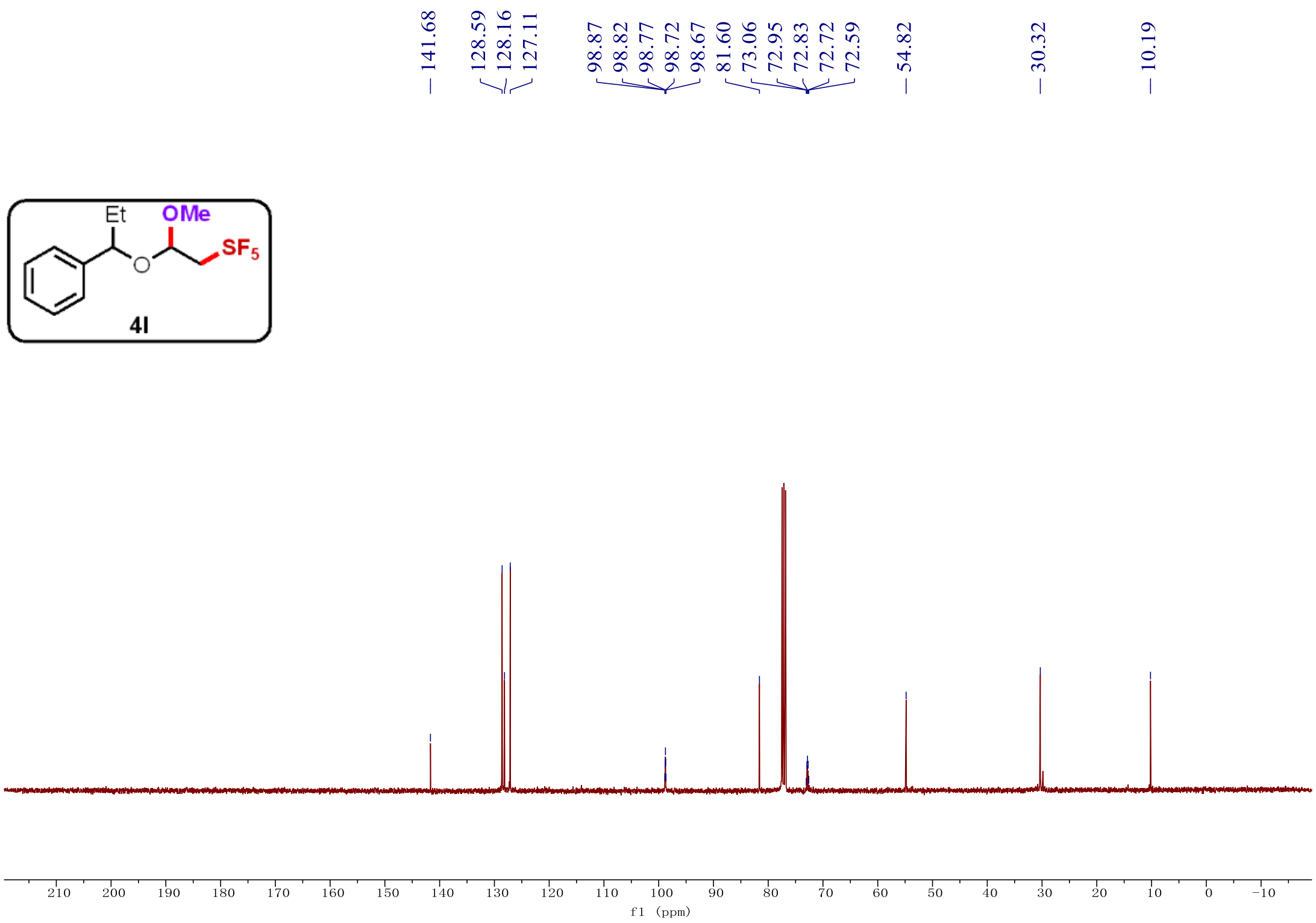

Supplementary Figure 128. ^{13}C NMR Spectrum of Compound 4l (101 MHz, $CDCl_3$, 25 ˚C)

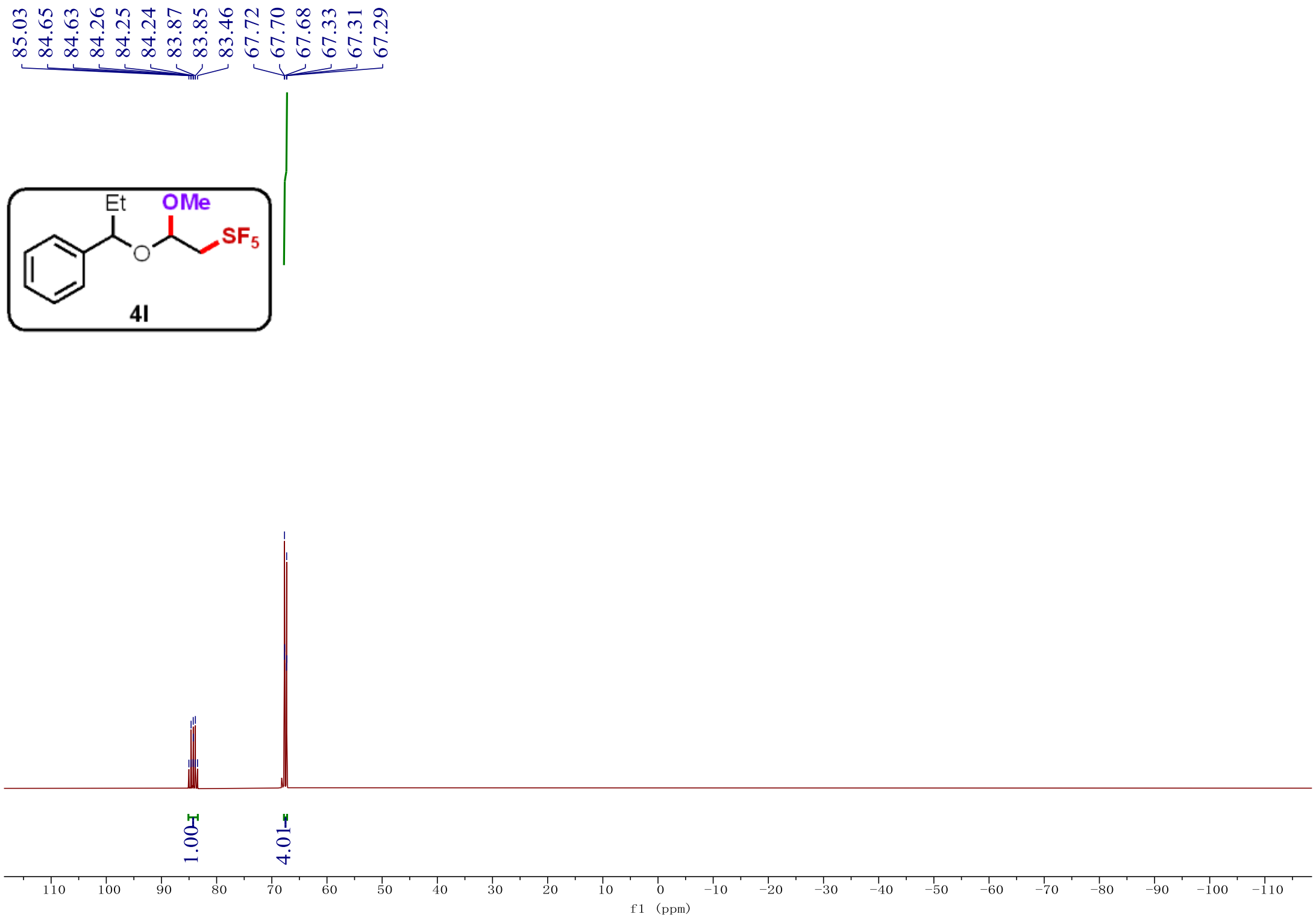

Supplementary Figure 129. ^{19}F NMR Spectrum of Compound 4l (376 MHz, $CDCl_3$, 25 ˚C)

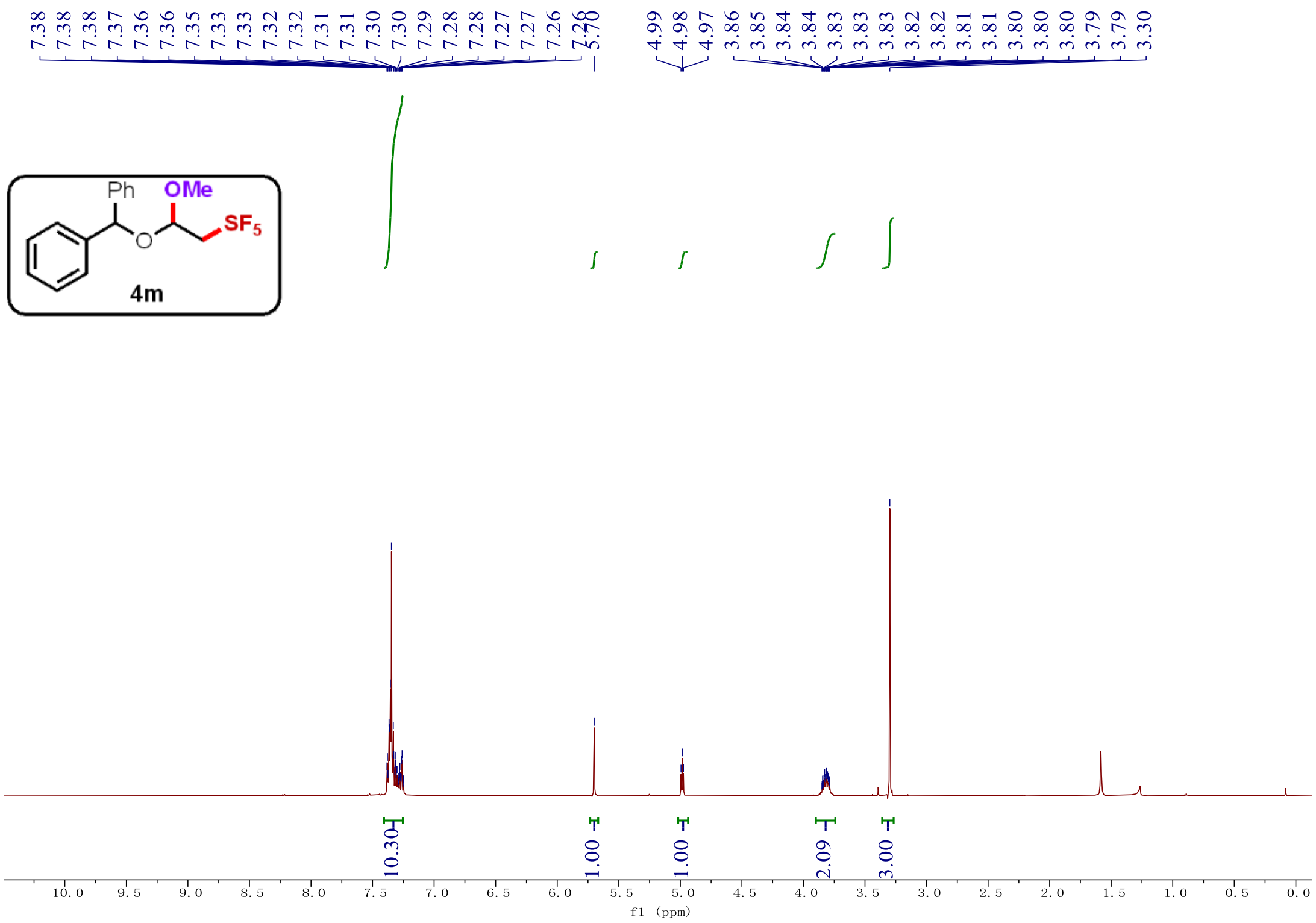

Supplementary Figure 130. ^{1}H NMR Spectrum of Compound 4m (500 MHz, $CDCl_3$, 25 ˚C)

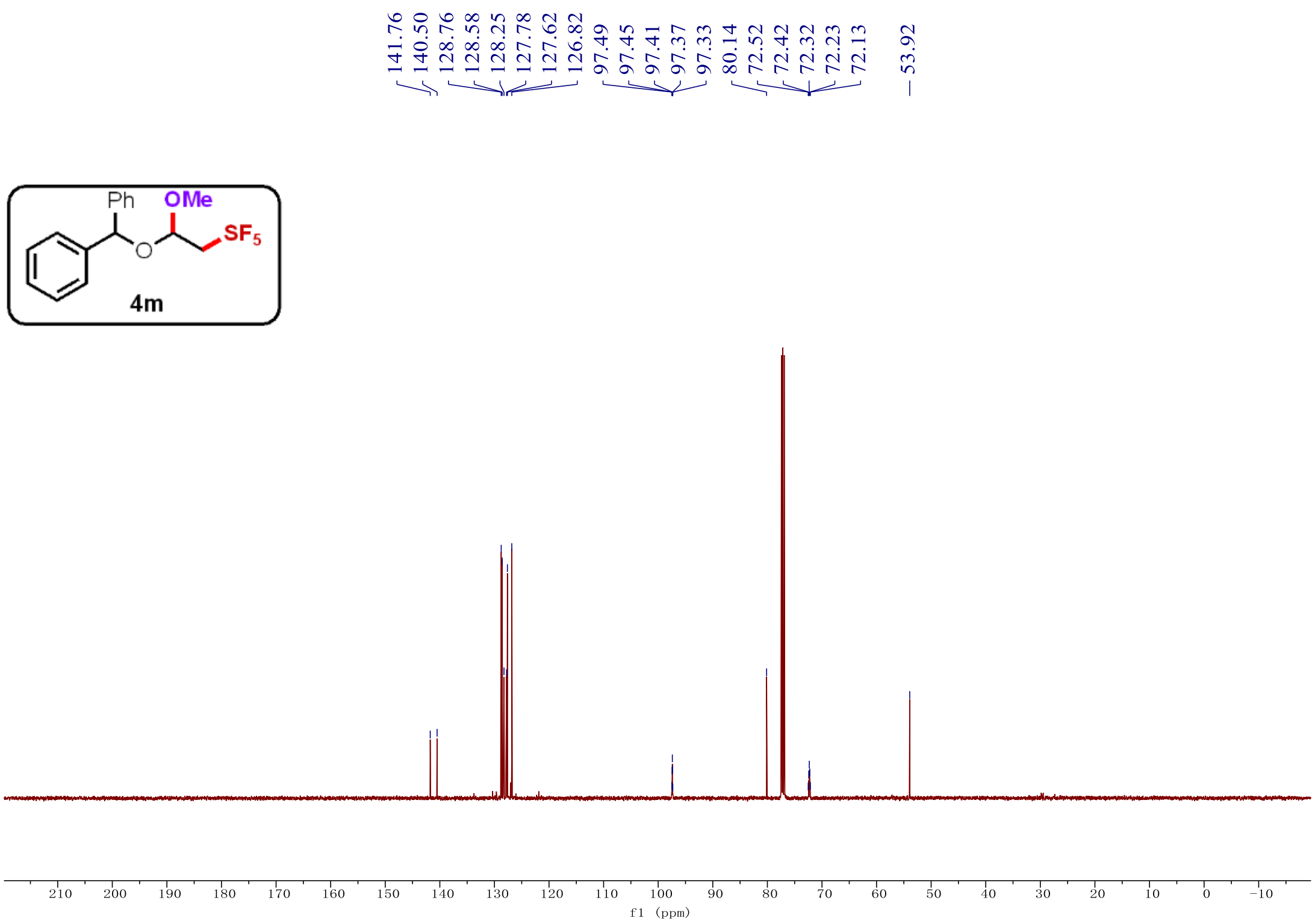

Supplementary Figure 131. ^{13}C NMR Spectrum of Compound 4m (126 MHz, $CDCl_3$, 25 °C)

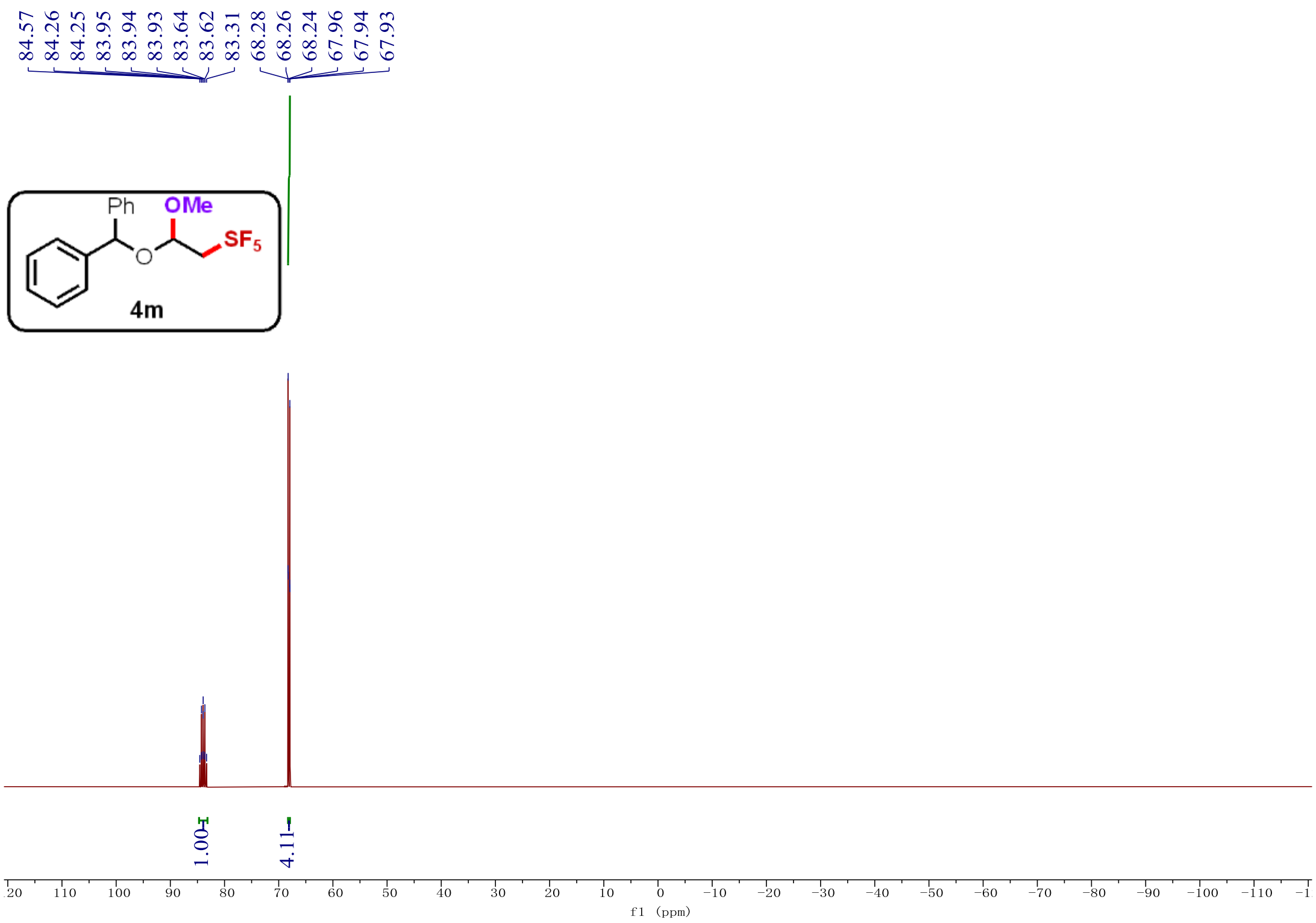

Supplementary Figure 132. ^{19}F NMR Spectrum of Compound 4m (471 MHz, $CDCl_3$, 25 °C)

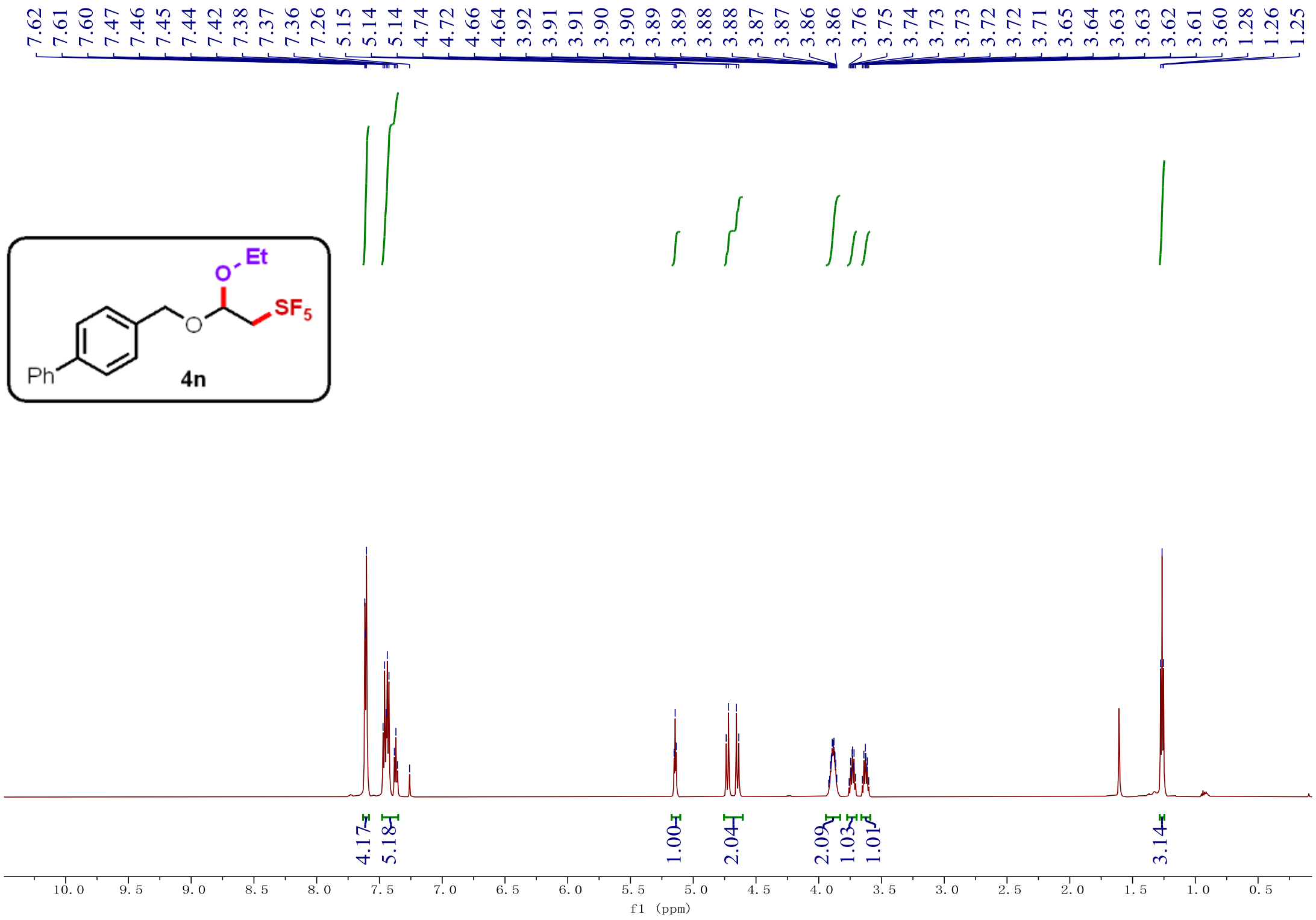

Supplementary Figure 133. ^{1}H **NMR Spectrum of Compound 4n (600 MHz, $CDCl_3$, 25 °C)**

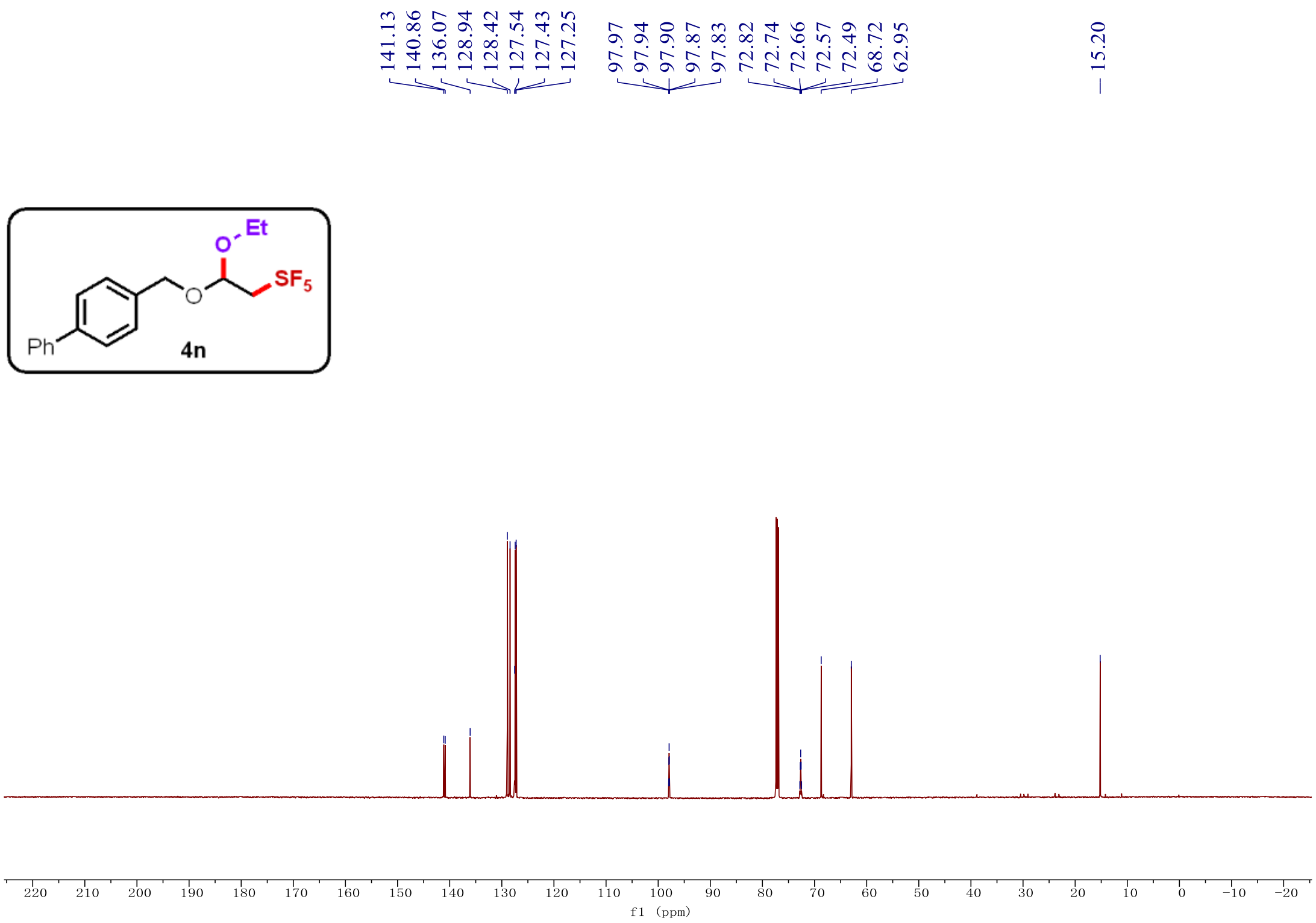

Supplementary Figure 134. ^{13}C **NMR Spectrum of Compound 4n (151 MHz, $CDCl_3$, 25 °C)**

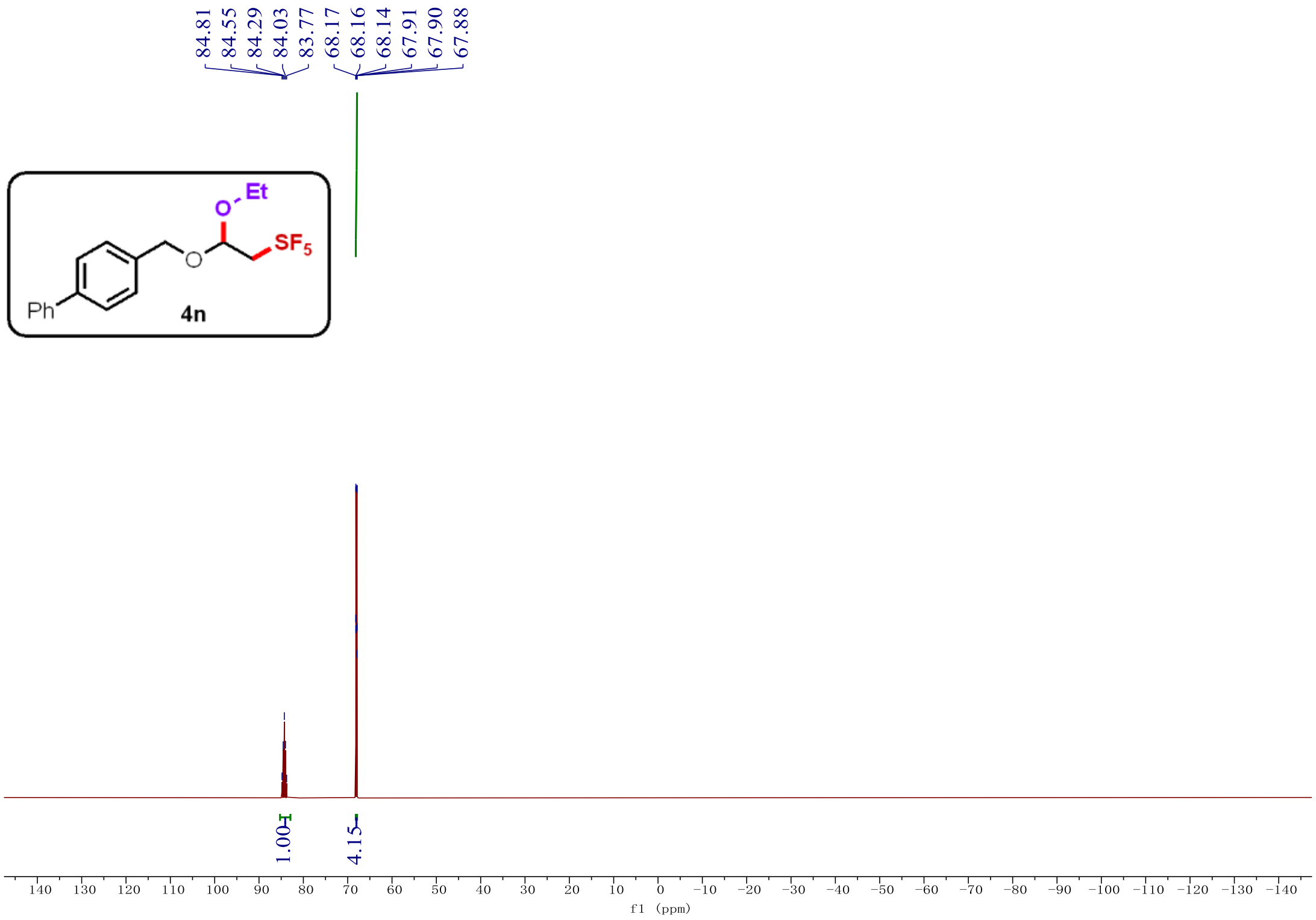

Supplementary Figure 135. ^{19}**F NMR Spectrum of Compound 4n (565 MHz,** $CDCl_3$**, 25 ˚C)**

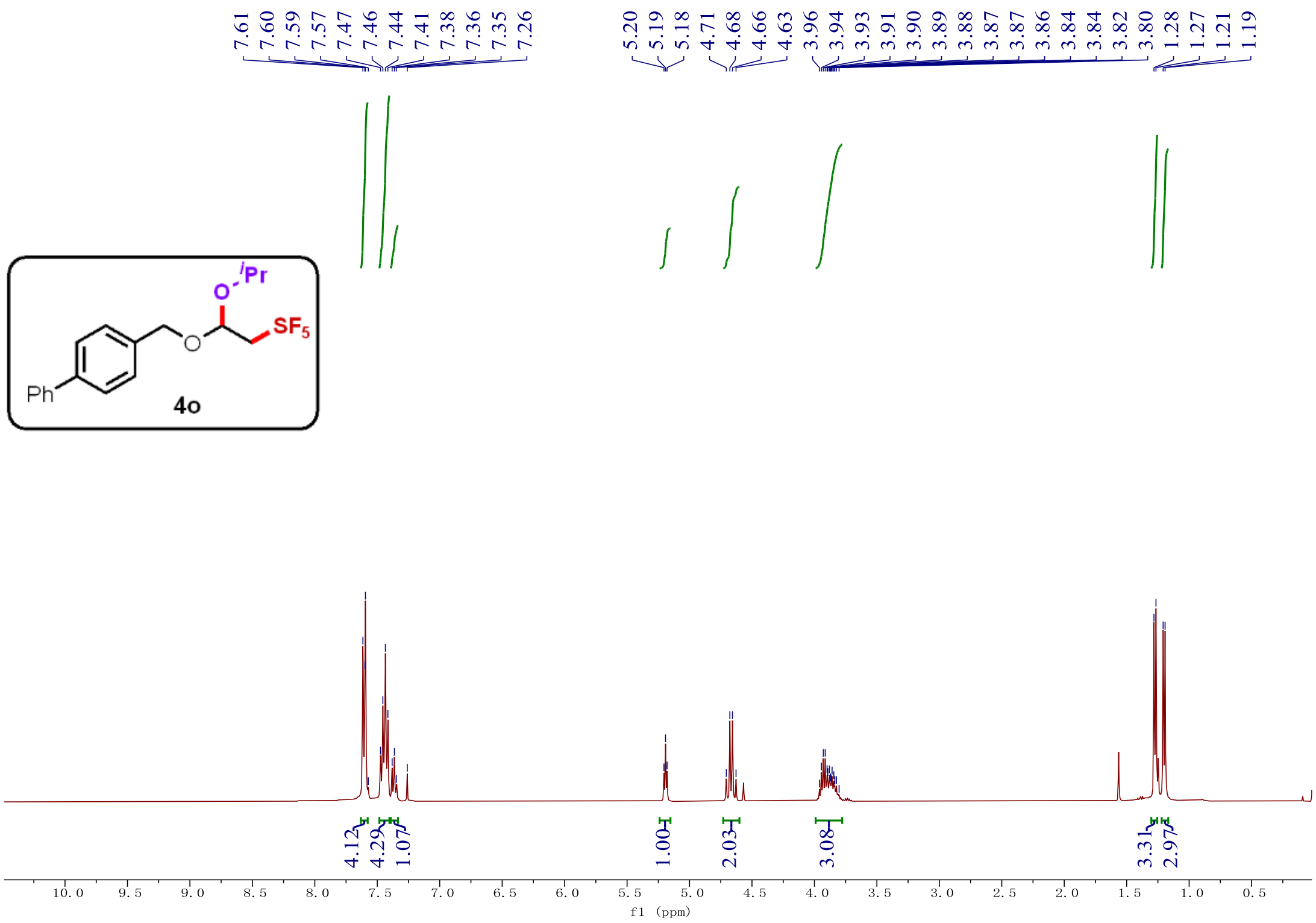

Supplementary Figure 136. ^{1}**H NMR Spectrum of Compound 4o (400 MHz,** $CDCl_3$**, 25 ˚C)**

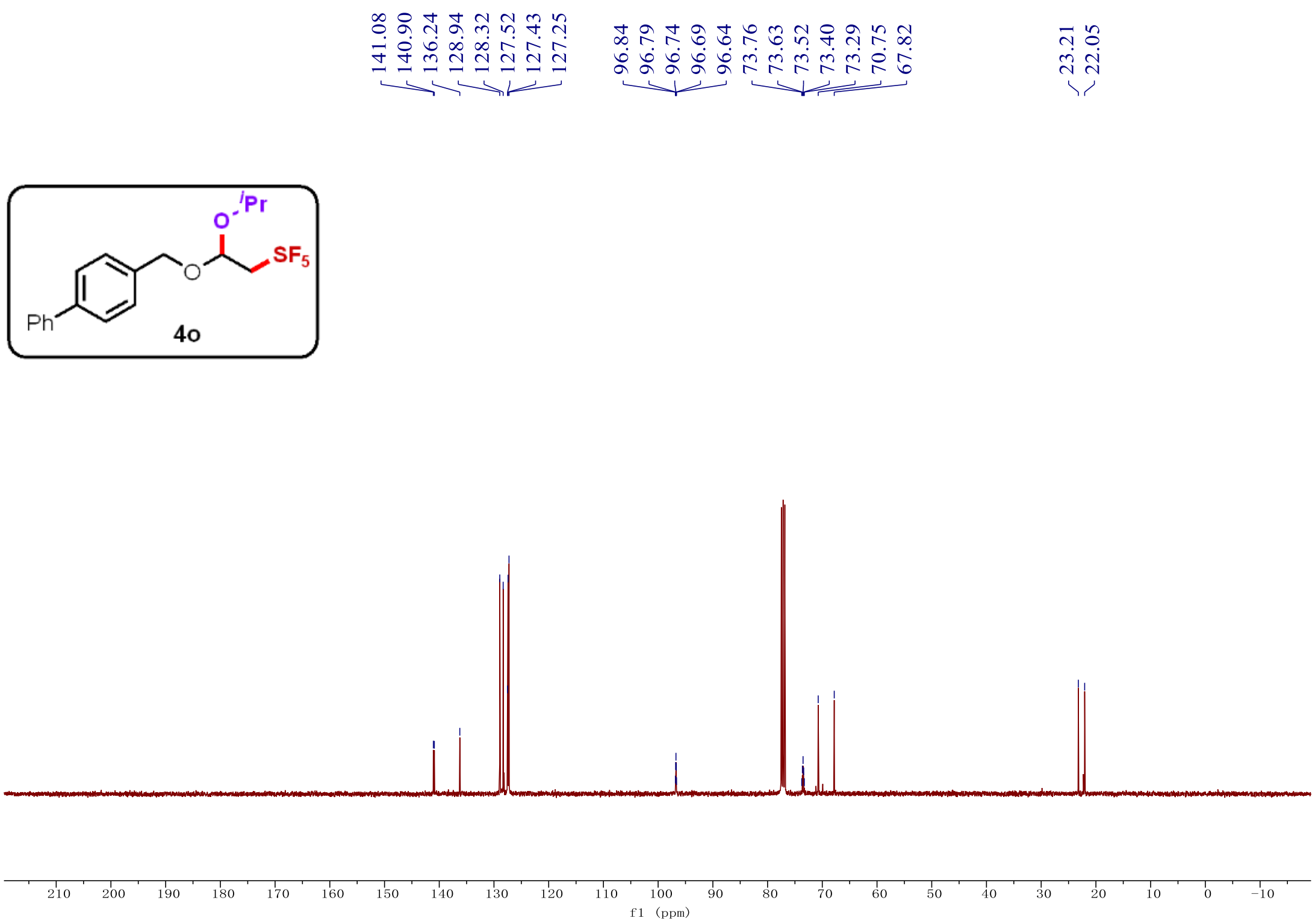

Supplementary Figure 137. ^{13}C NMR Spectrum of Compound 4o (101 MHz, $CDCl_3$, 25 ˚C)

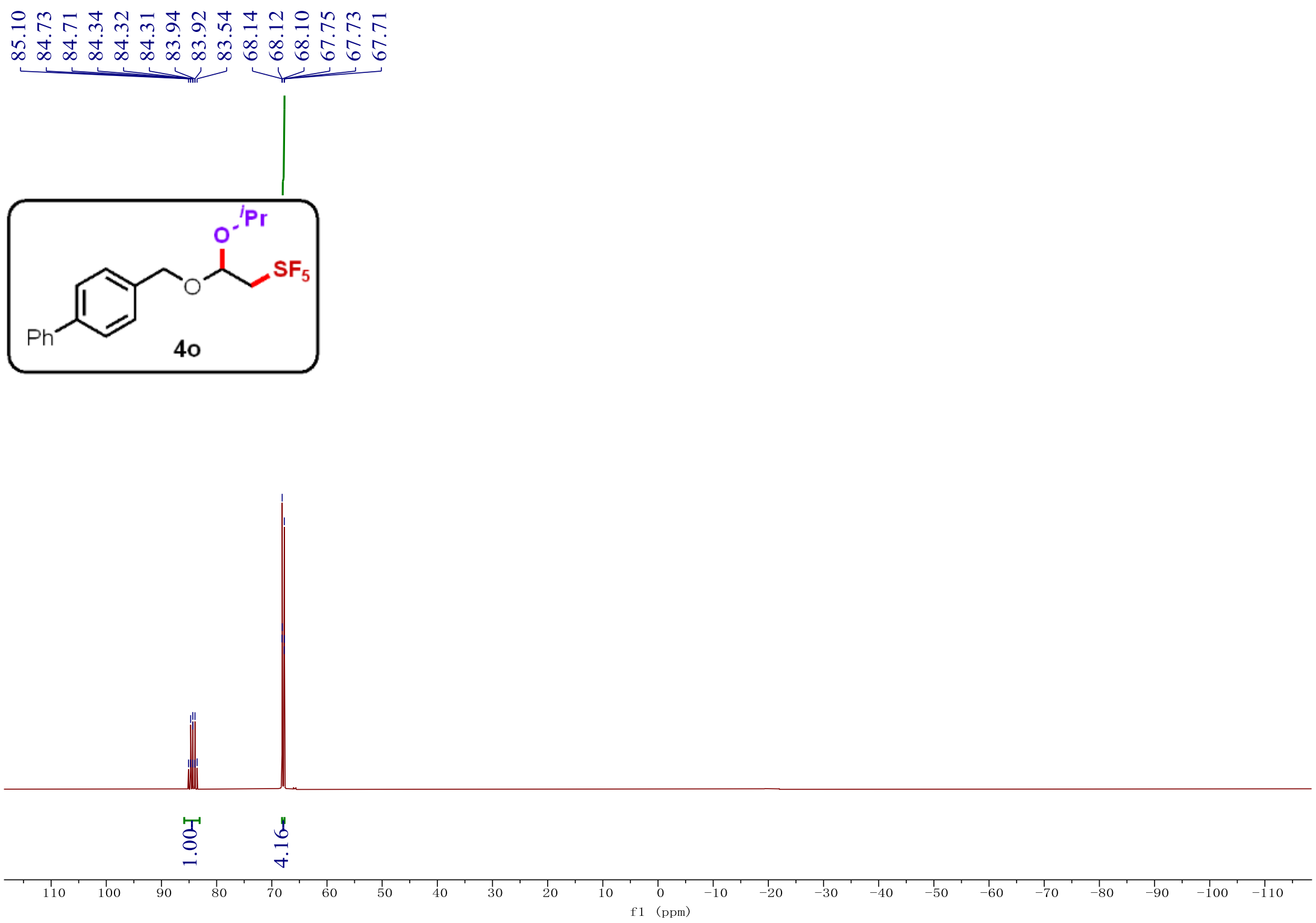

Supplementary Figure 138. ^{19}F NMR Spectrum of Compound 4o (376 MHz, $CDCl_3$, 25 ˚C)

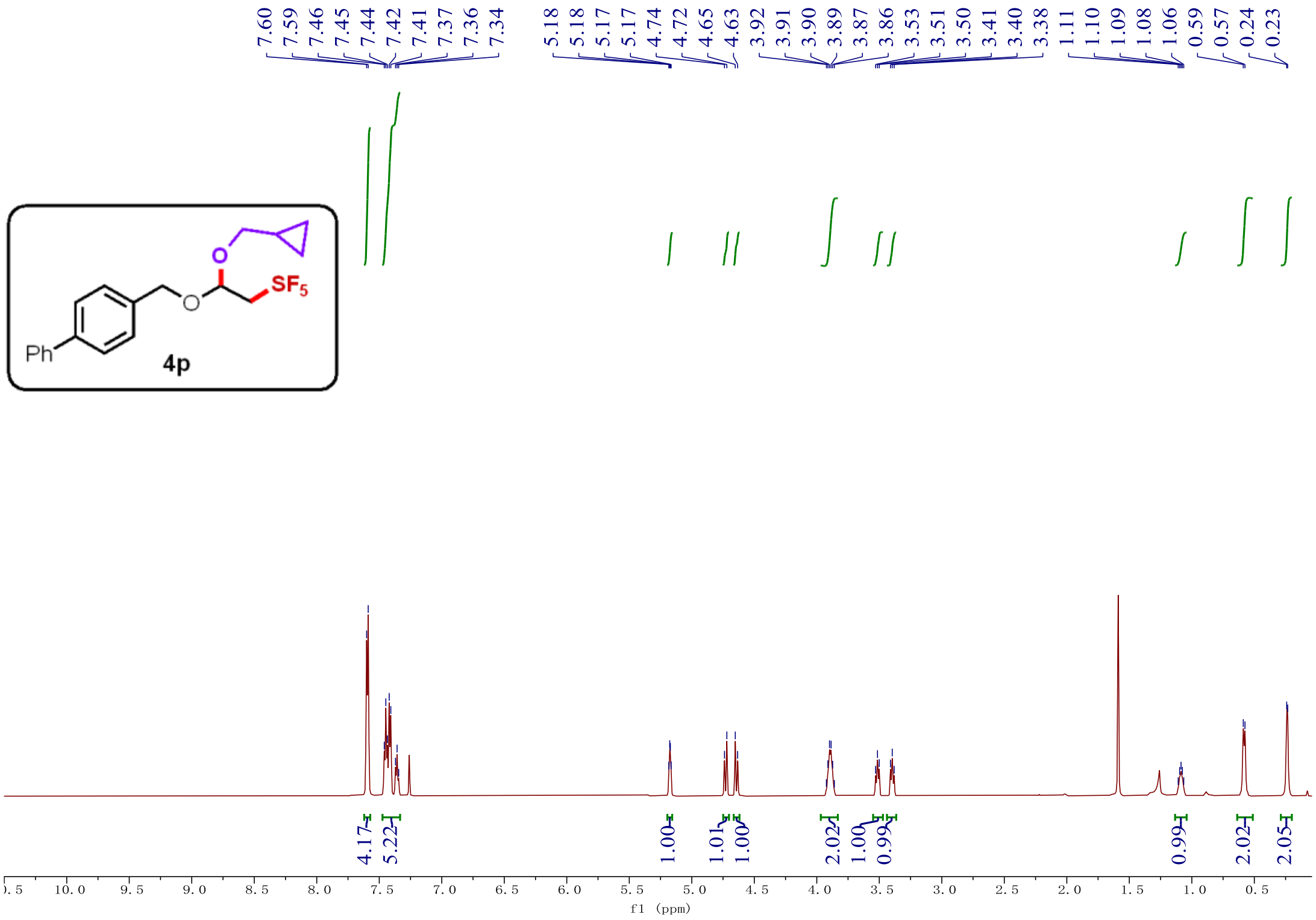

Supplementary Figure 139. ^{1}H NMR Spectrum of Compound 4p (500 MHz, $CDCl_3$, 25 °C)

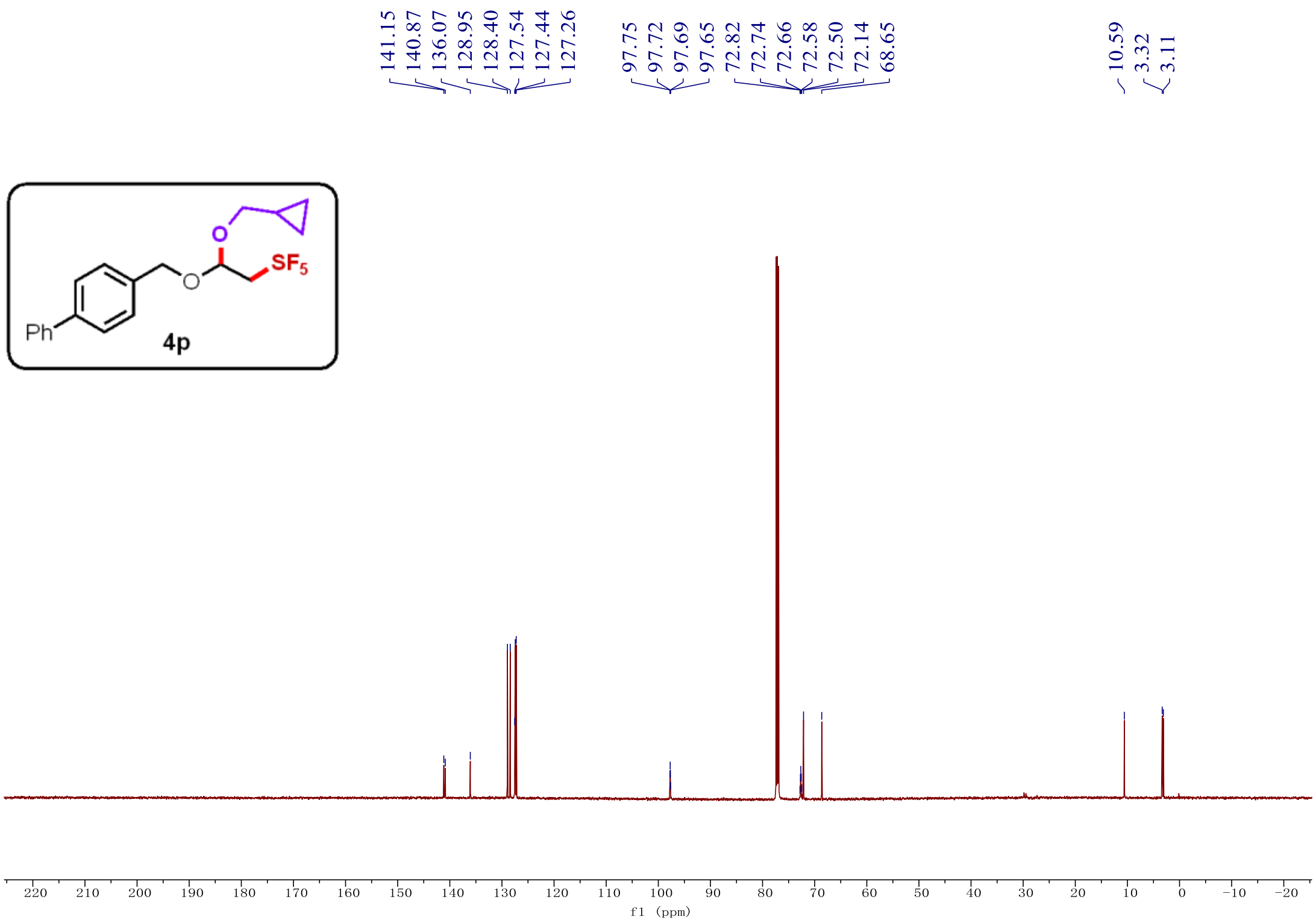

Supplementary Figure 140. ^{13}C NMR Spectrum of Compound 4p (126 MHz, $CDCl_3$, 25 °C)

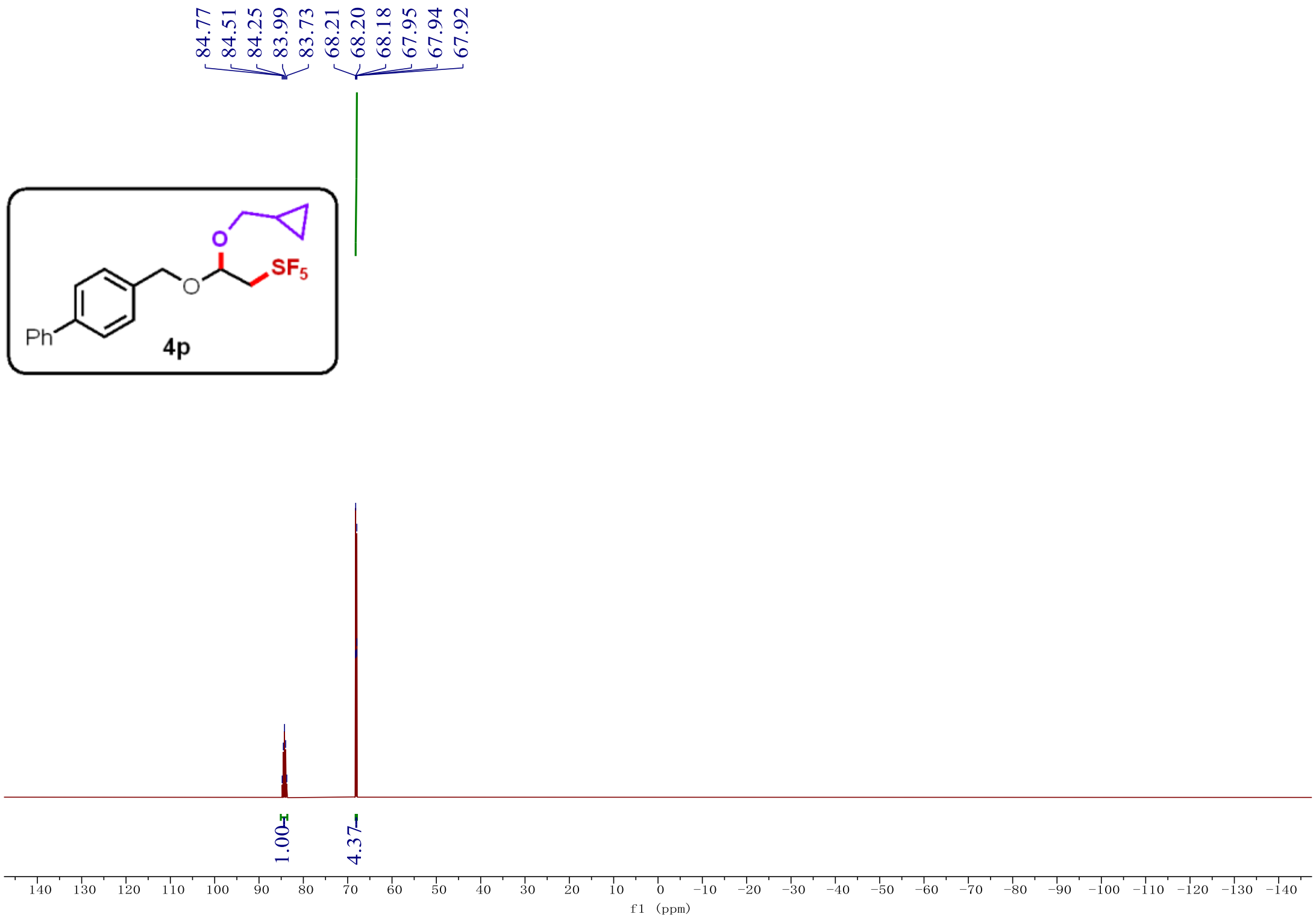

Supplementary Figure 141. ^{19}**F NMR Spectrum of Compound 4p (565 MHz, CDCl**$_3$**, 25 °C)**

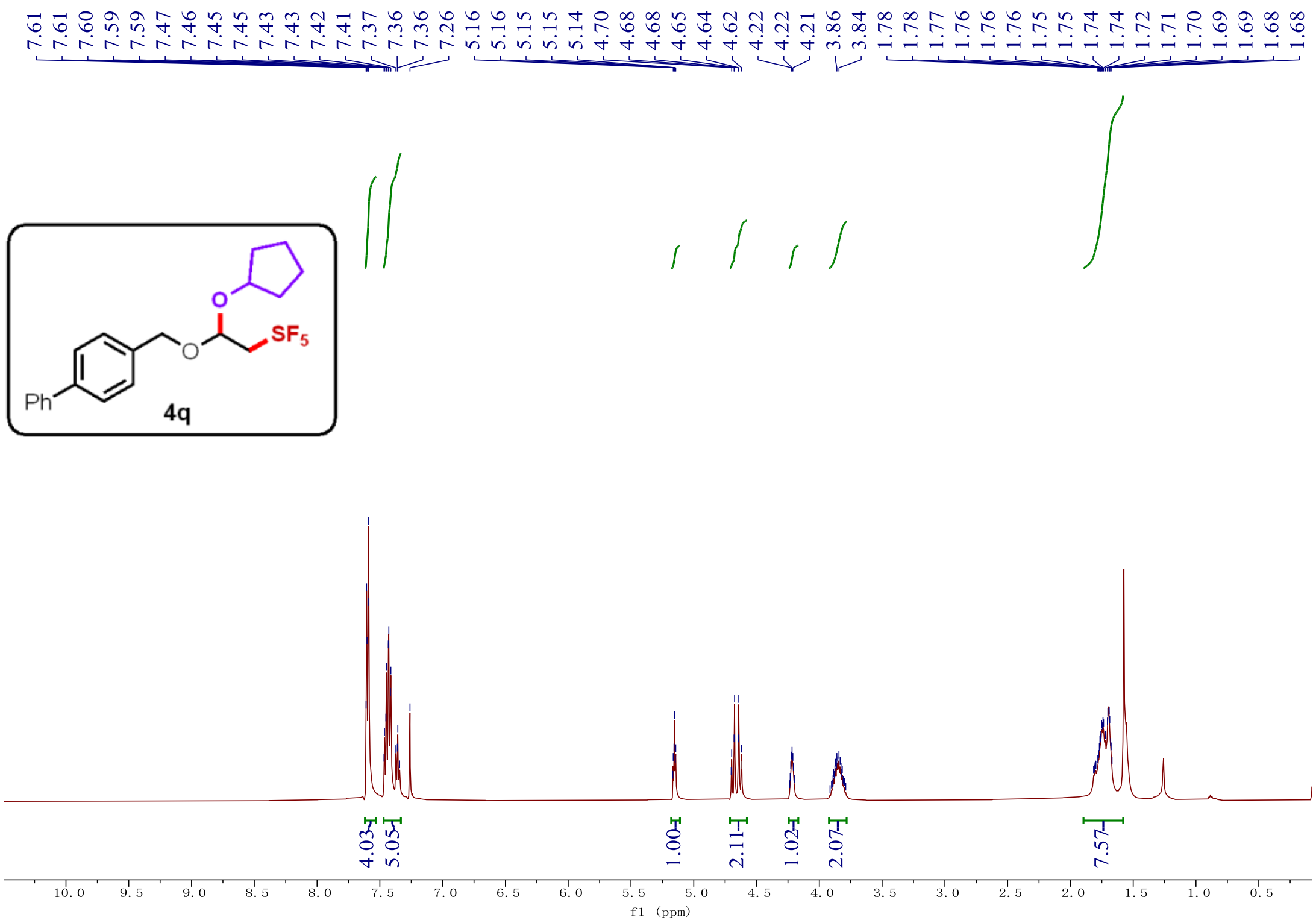

Supplementary Figure 142. ^{1}**H NMR Spectrum of Compound 4q (500 MHz, CDCl**$_3$**, 25 °C)**

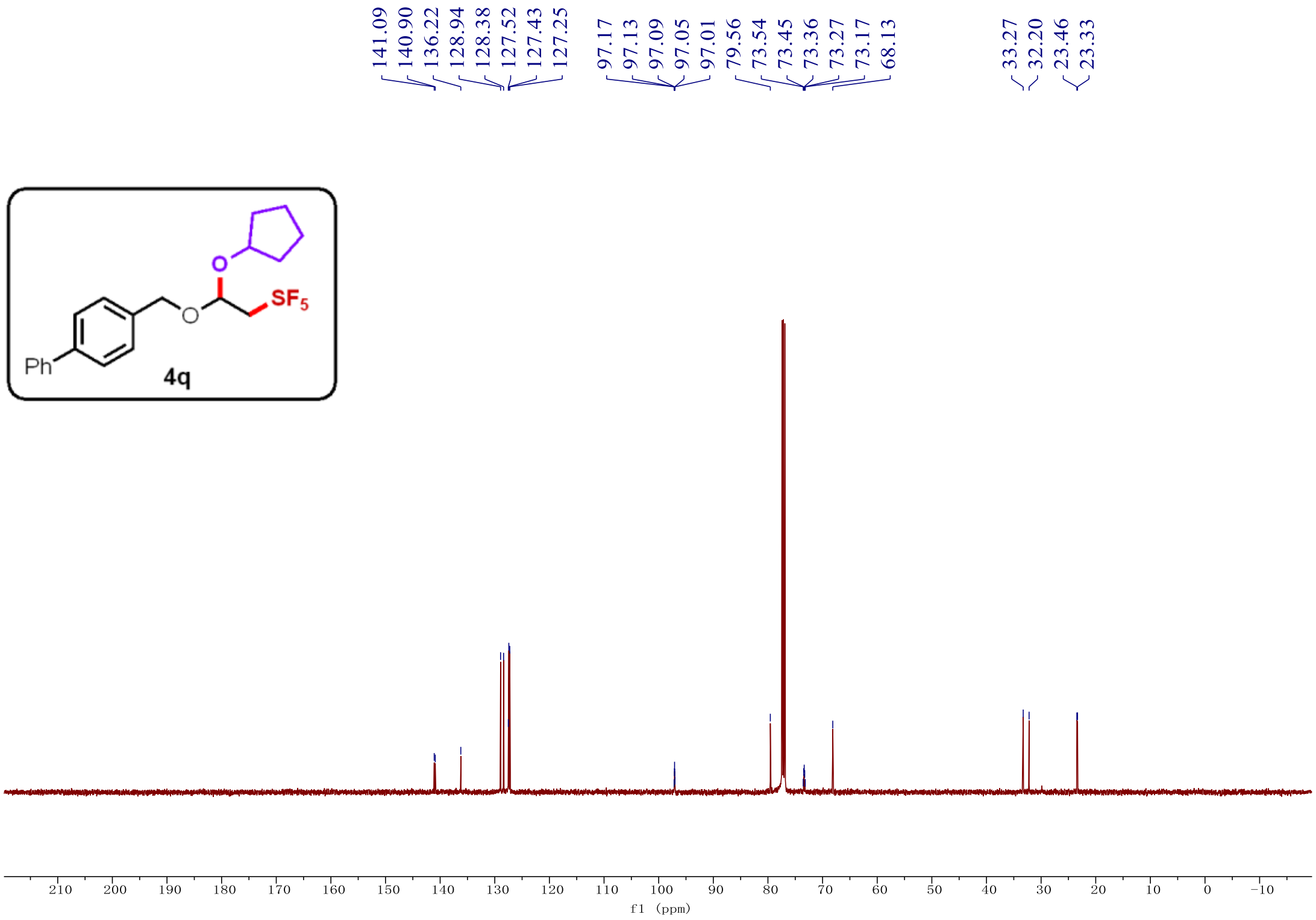

Supplementary Figure 143. ^{13}C NMR Spectrum of Compound 4q (126 MHz, $CDCl_3$, 25 ˚C)

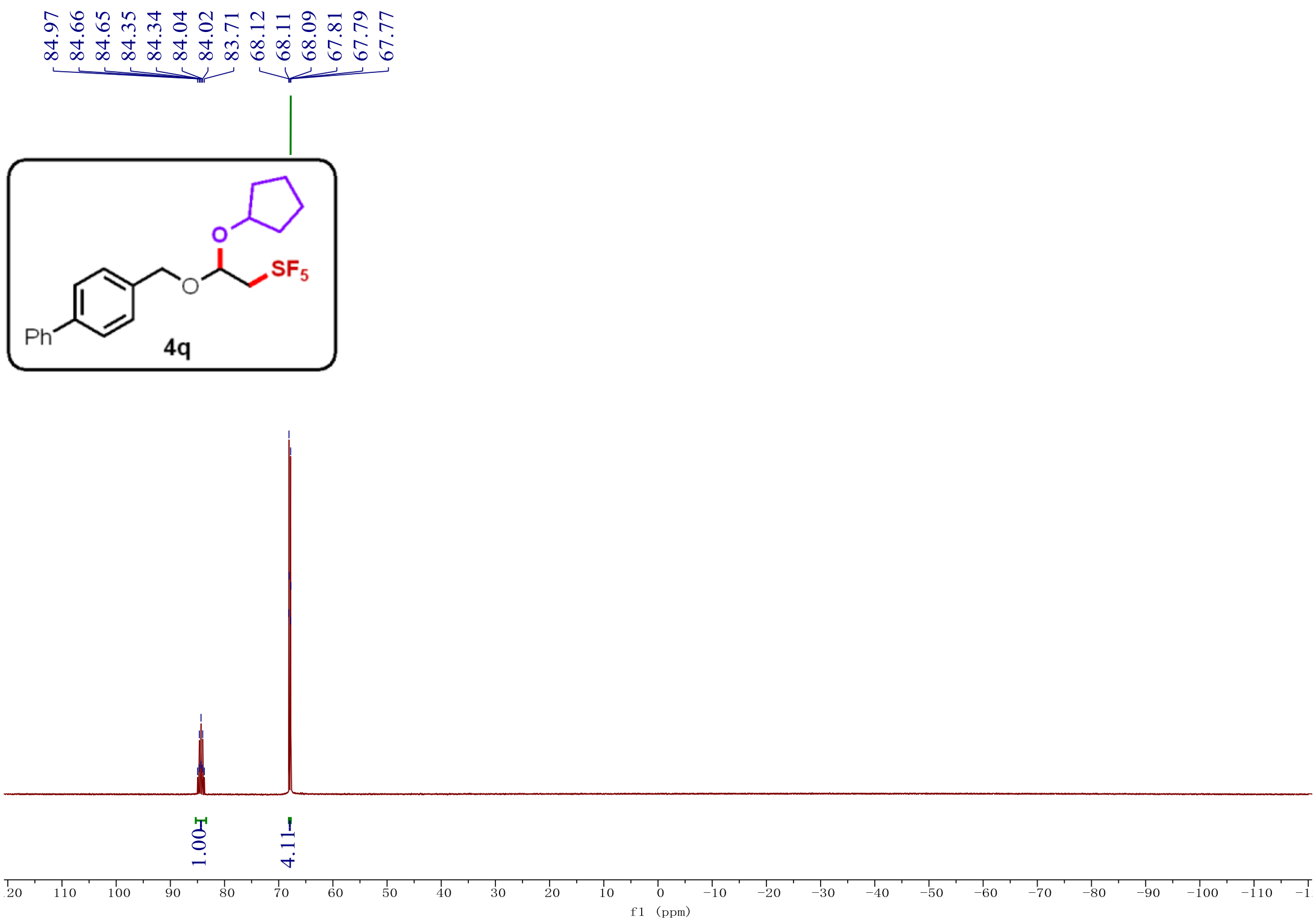

Supplementary Figure 144. ^{19}F NMR Spectrum of Compound 4q (471 MHz, $CDCl_3$, 25 ˚C)

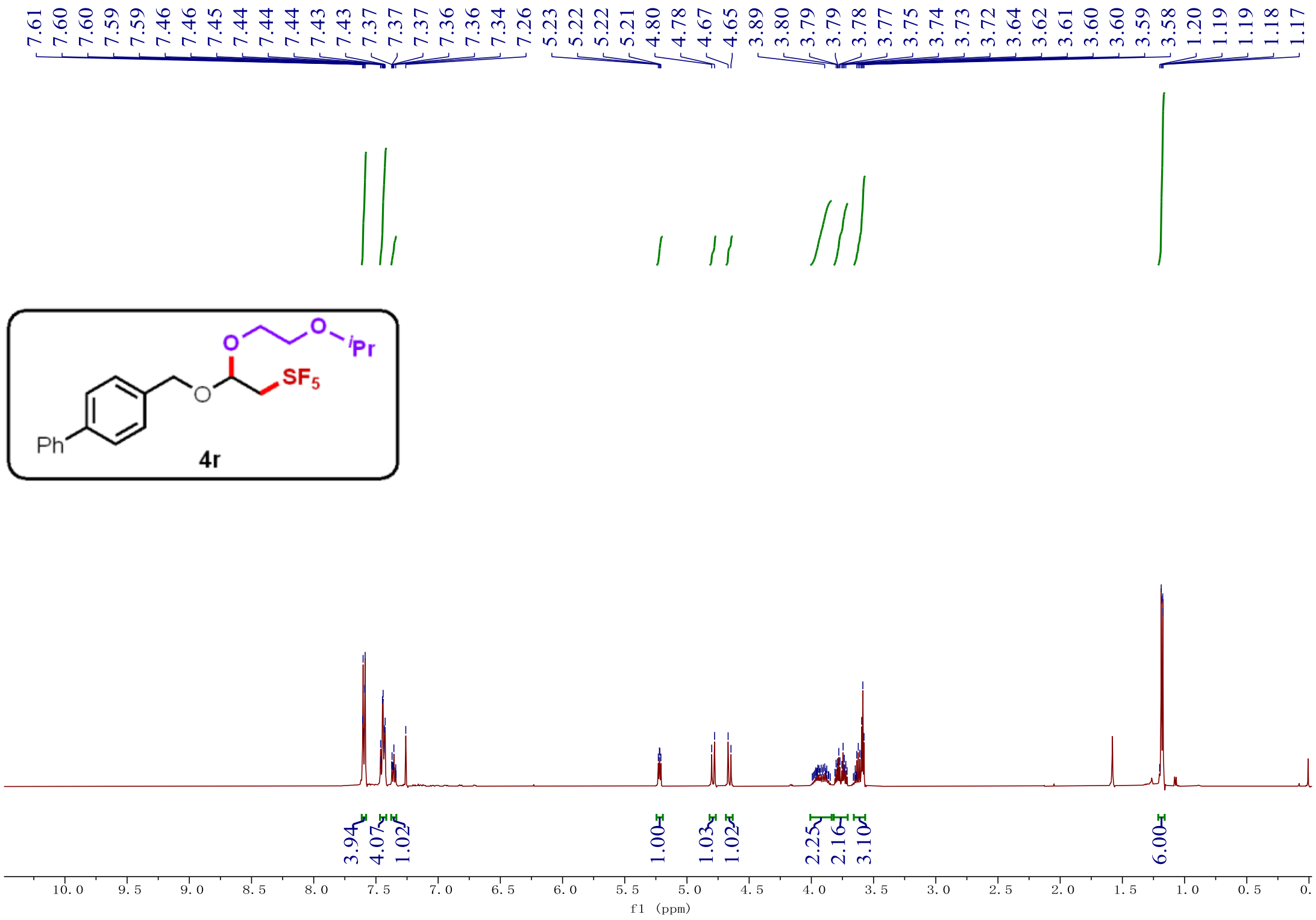

Supplementary Figure 145. **^{1}H NMR Spectrum of Compound 4r (500 MHz, $CDCl_3$, 25 ˚C)**

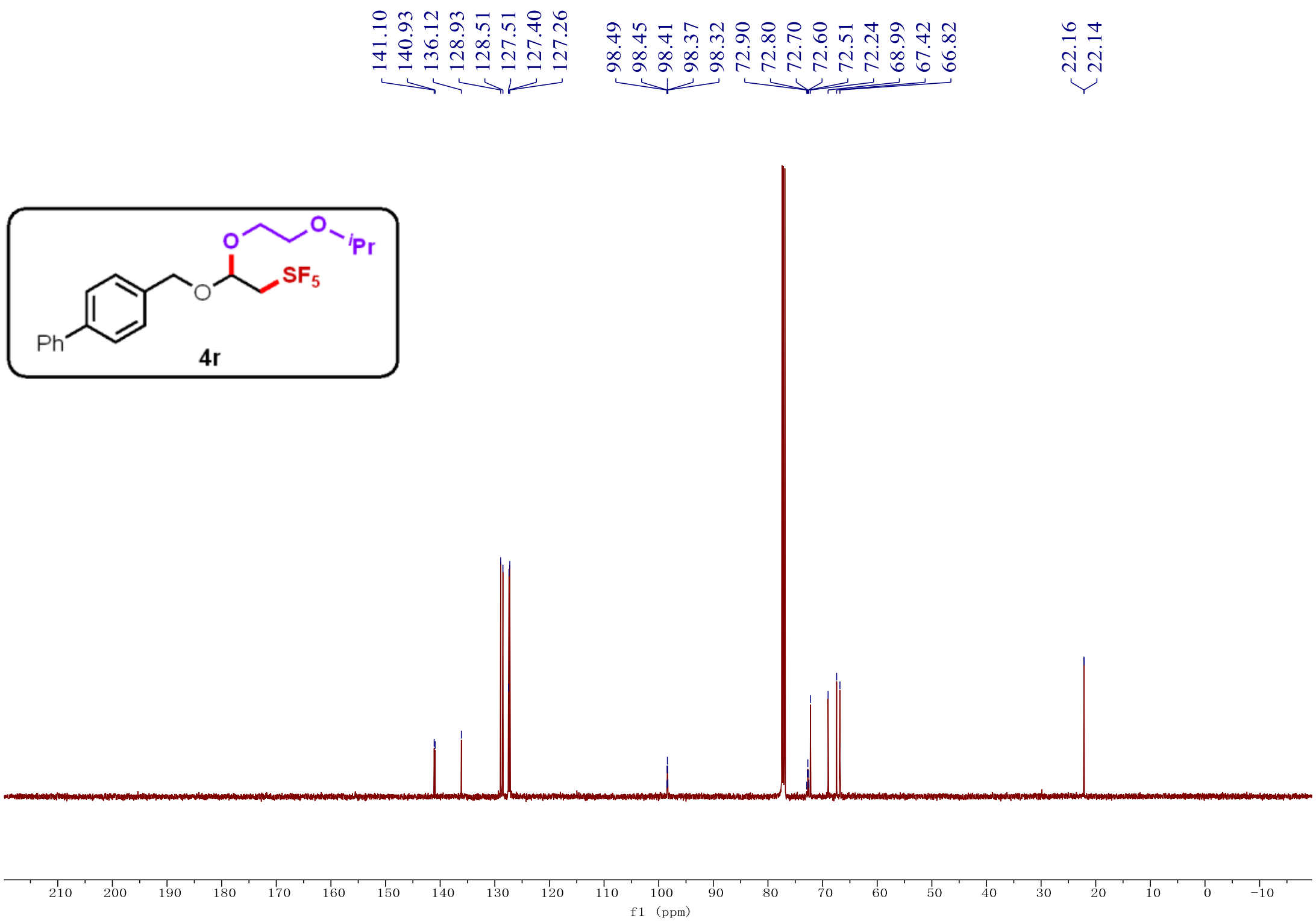

Supplementary Figure 146. **^{13}C NMR Spectrum of Compound 4r (126 MHz, $CDCl_3$, 25 ˚C)**

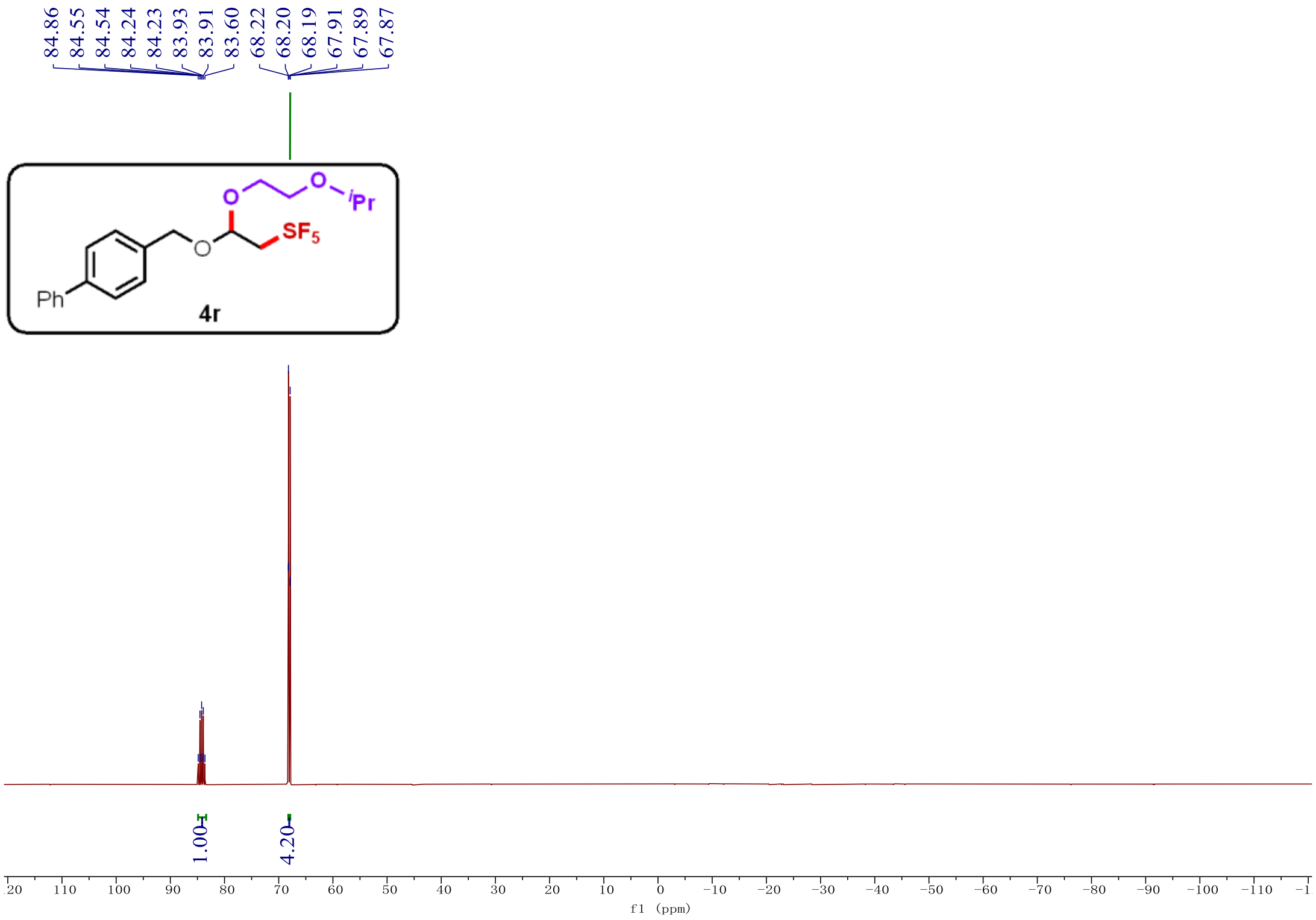

Supplementary Figure 147. ^{19}F NMR Spectrum of Compound 4r (471 MHz, $CDCl_3$, 25 ˚C)

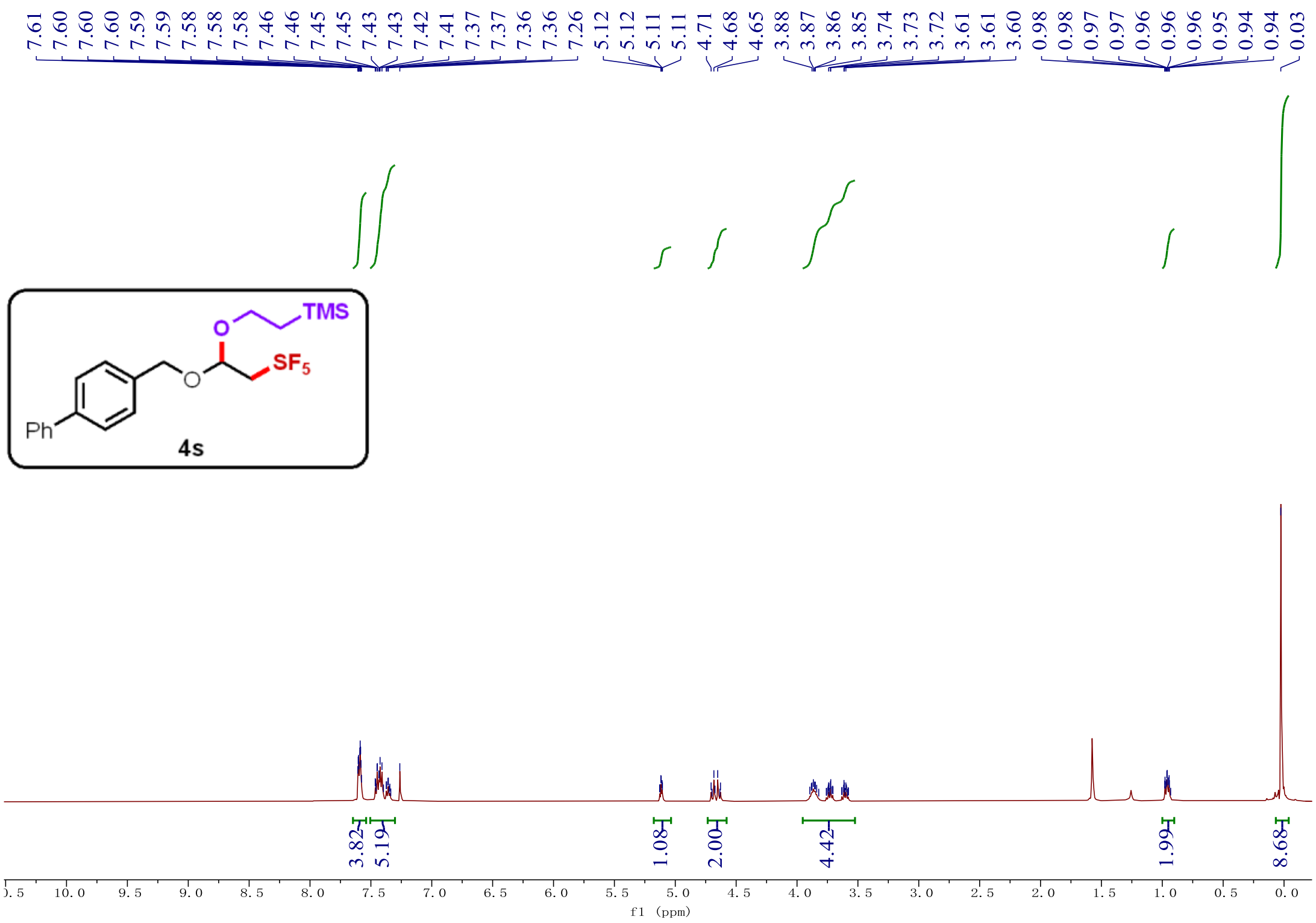

Supplementary Figure 148. ^{1}H NMR Spectrum of Compound 4s (500 MHz, $CDCl_3$, 25 ˚C)

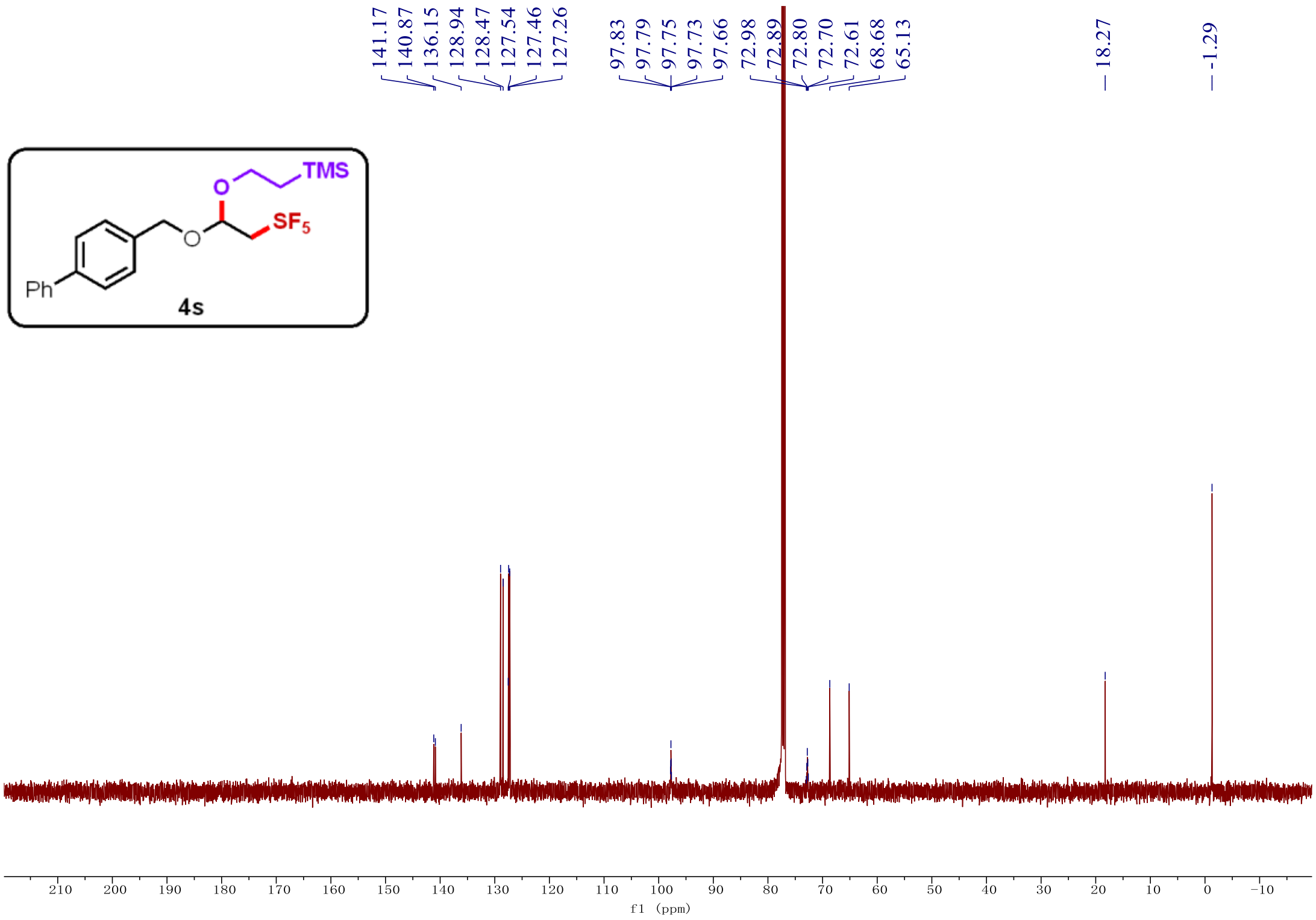

Supplementary Figure 149. ^{13}C **NMR Spectrum of Compound 4s (126 MHz, $CDCl_3$, 25 ˚C)**

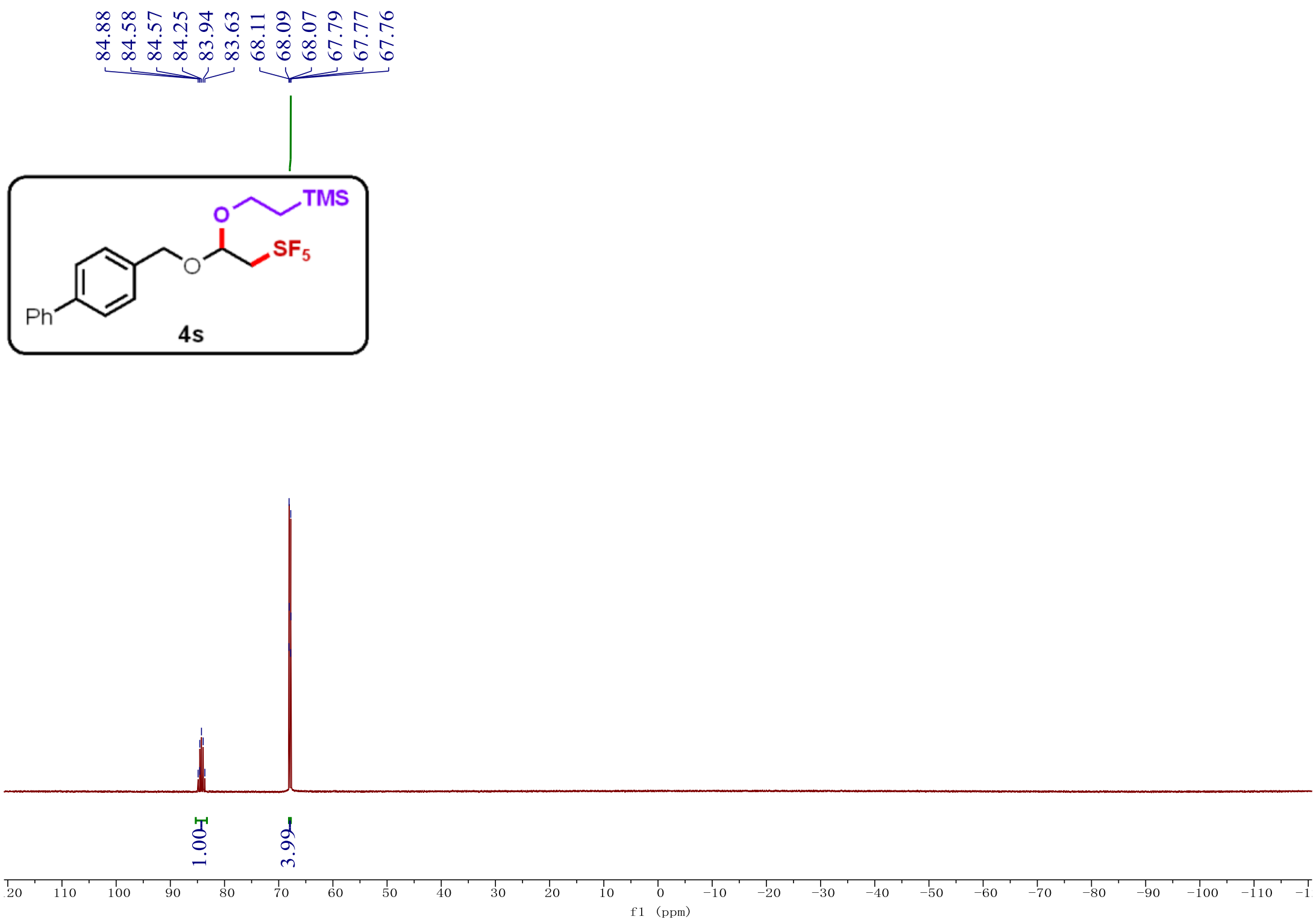

Supplementary Figure 150. ^{19}F **NMR Spectrum of Compound 4s (471 MHz, $CDCl_3$, 25 ˚C)**

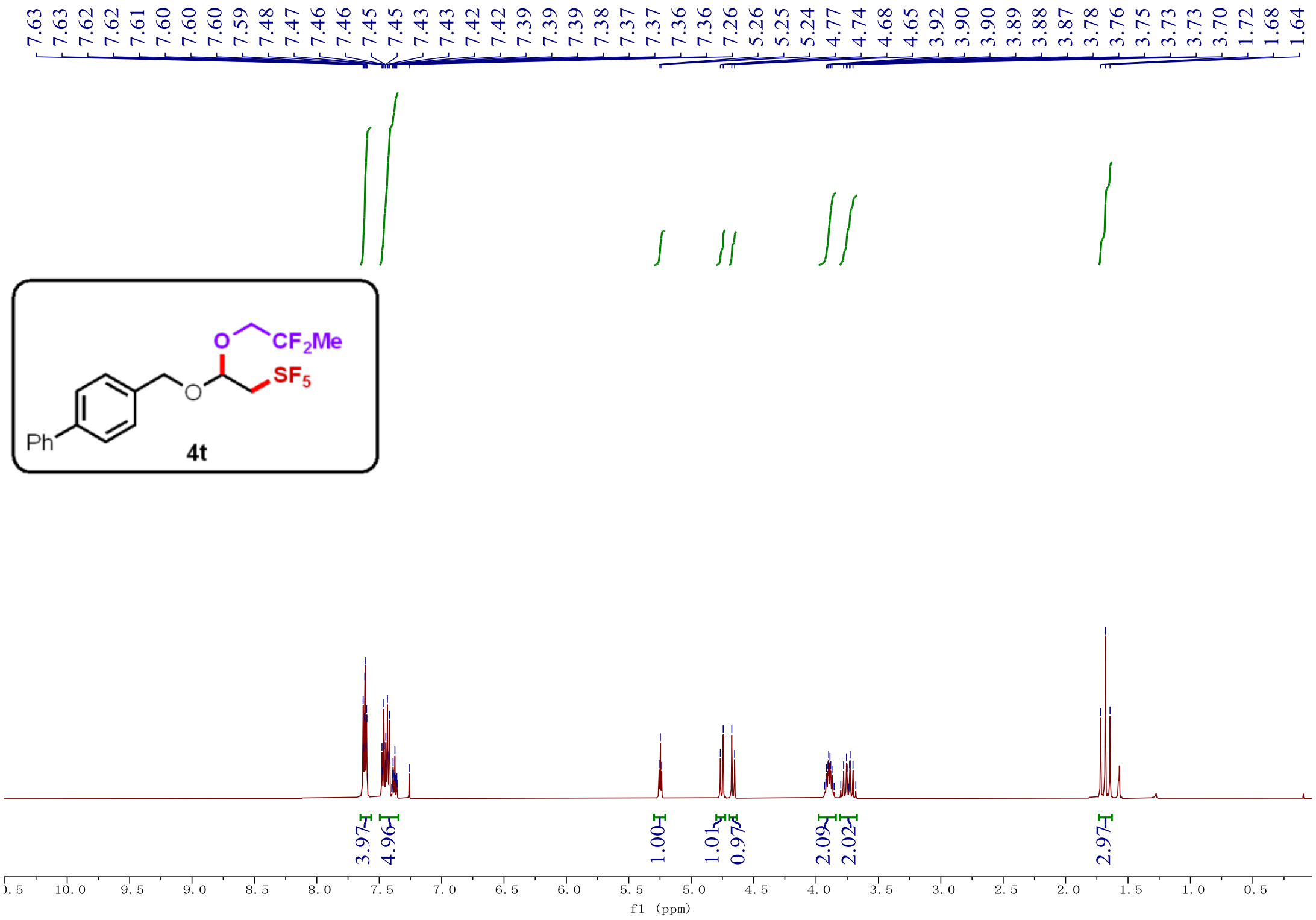

Supplementary Figure 151. ^{1}H NMR Spectrum of Compound 4t (500 MHz, $CDCl_3$, 25 ˚C)

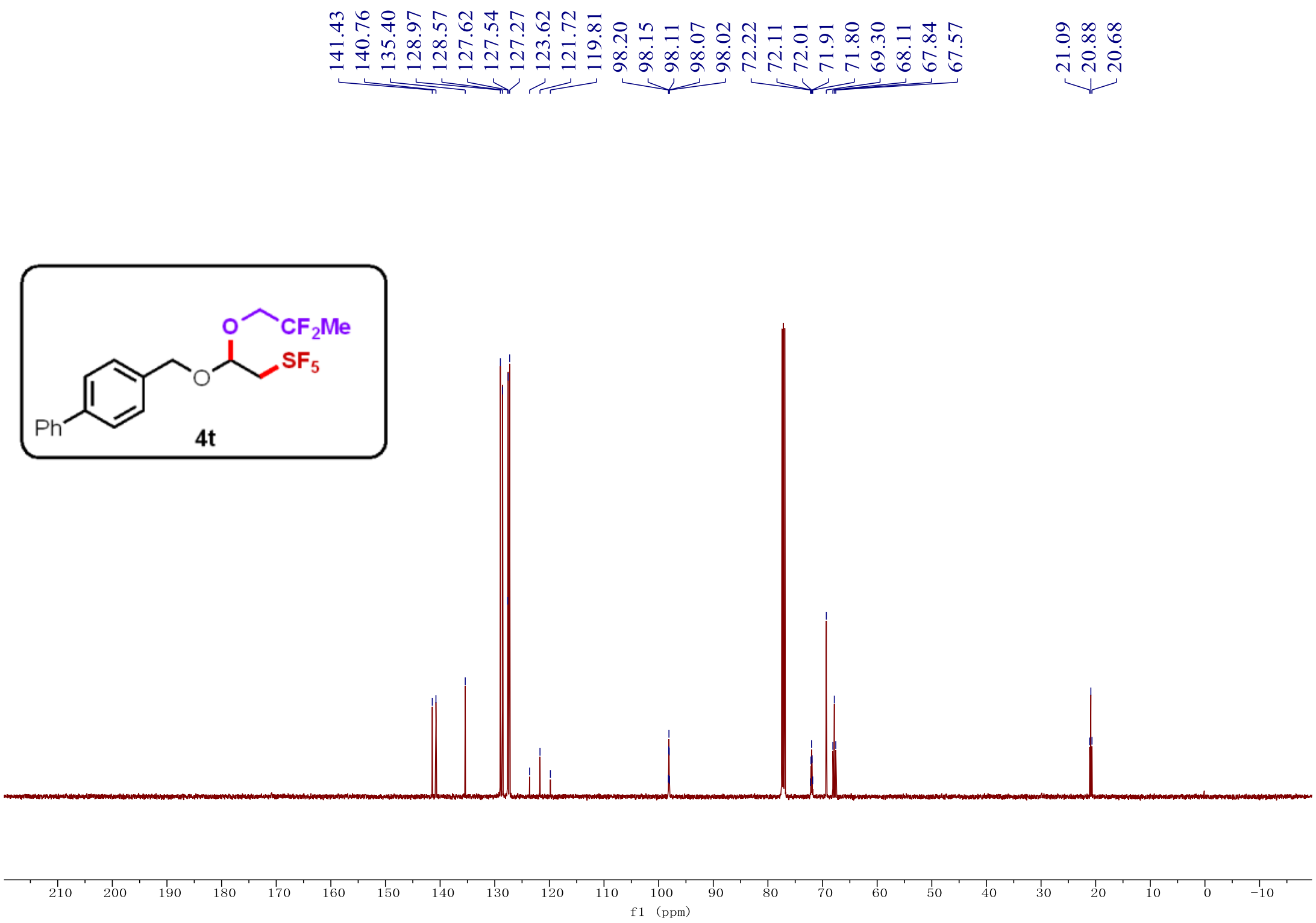

Supplementary Figure 152. ^{13}C NMR Spectrum of Compound 4t (126 MHz, $CDCl_3$, 25 ˚C)

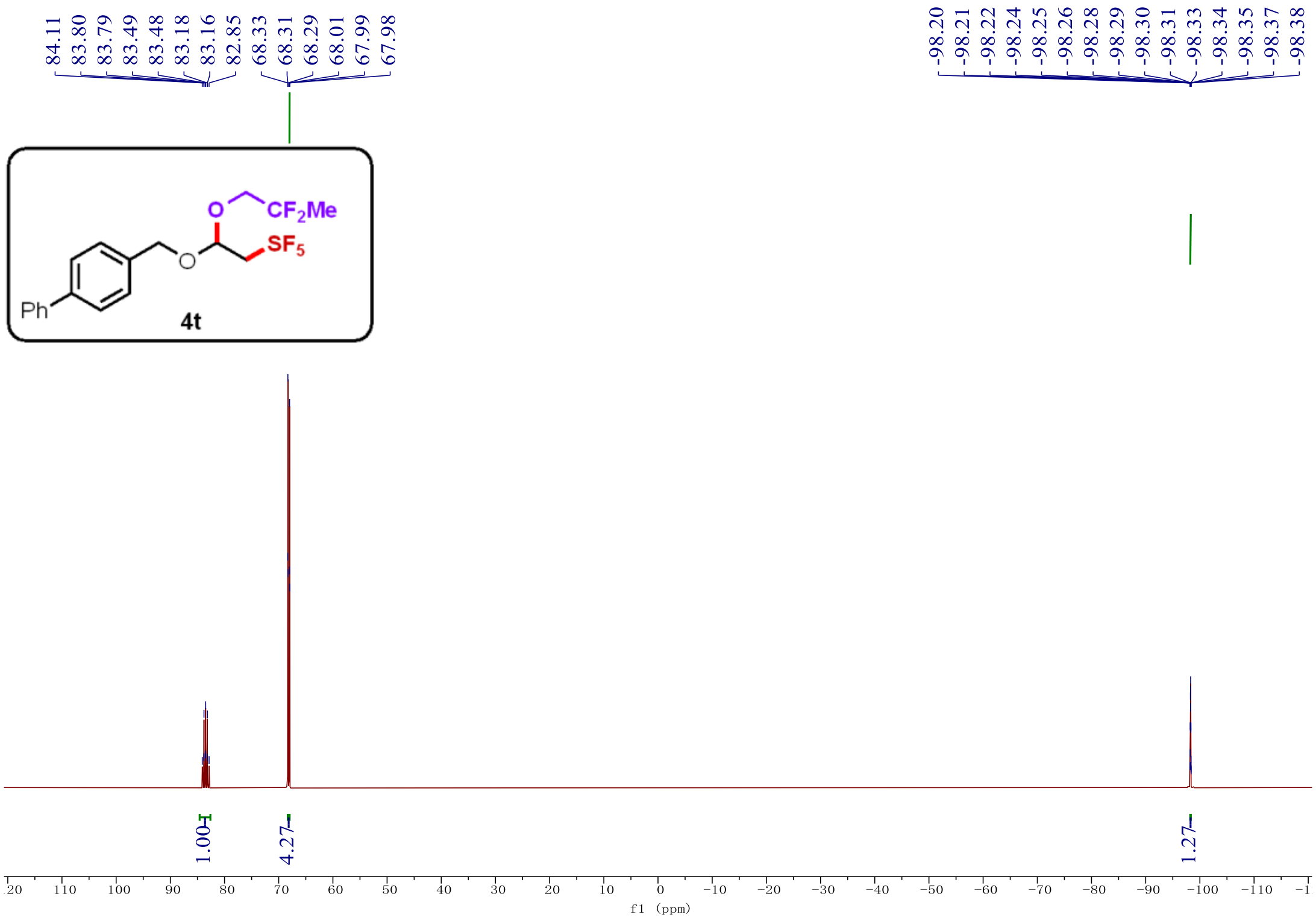

Supplementary Figure 153. ^{19}F NMR Spectrum of Compound 4t (471 MHz, $CDCl_3$, 25 ˚C)

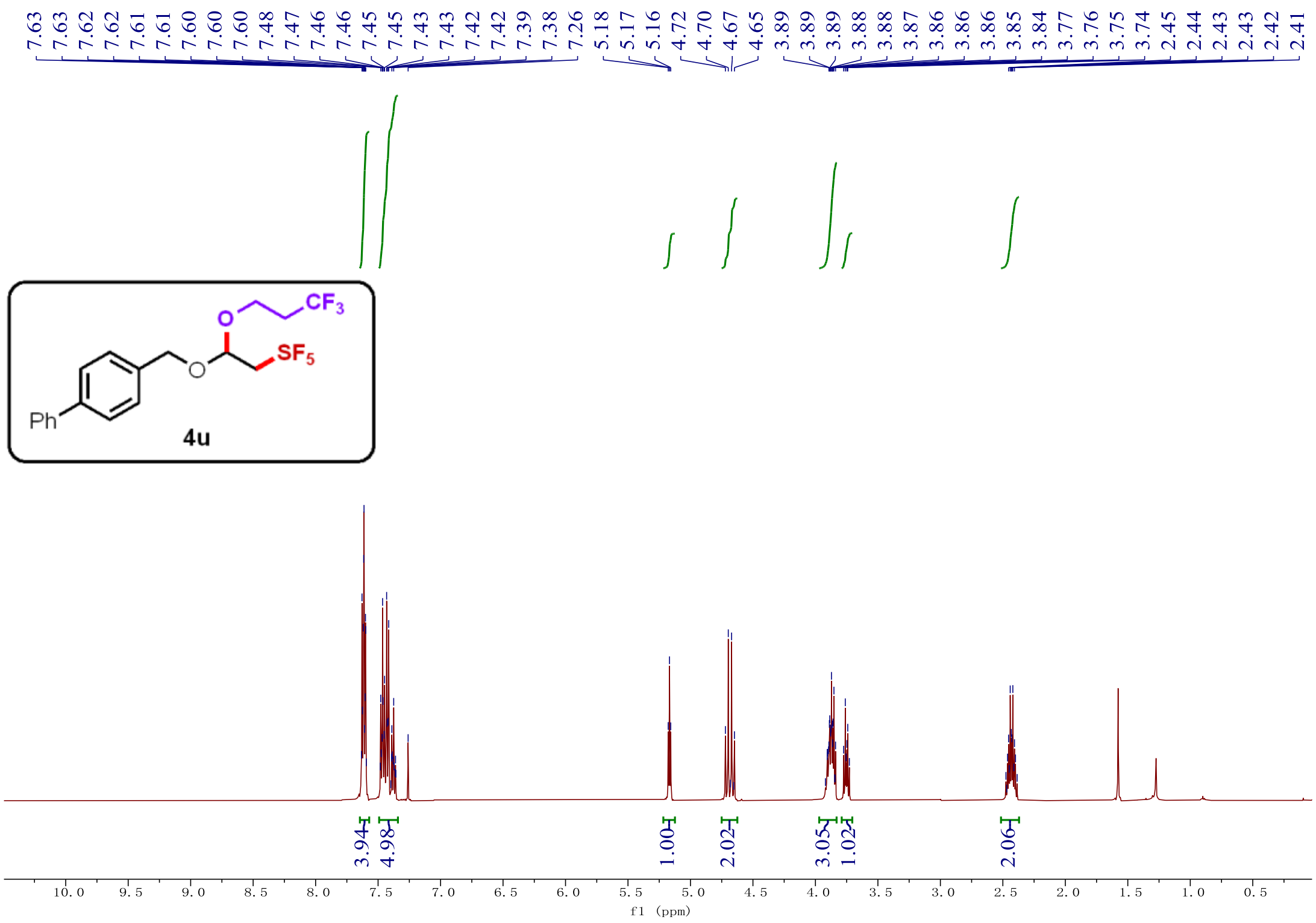

Supplementary Figure 154. ^{1}H NMR Spectrum of Compound 4u (500 MHz, $CDCl_3$, 25 ˚C)

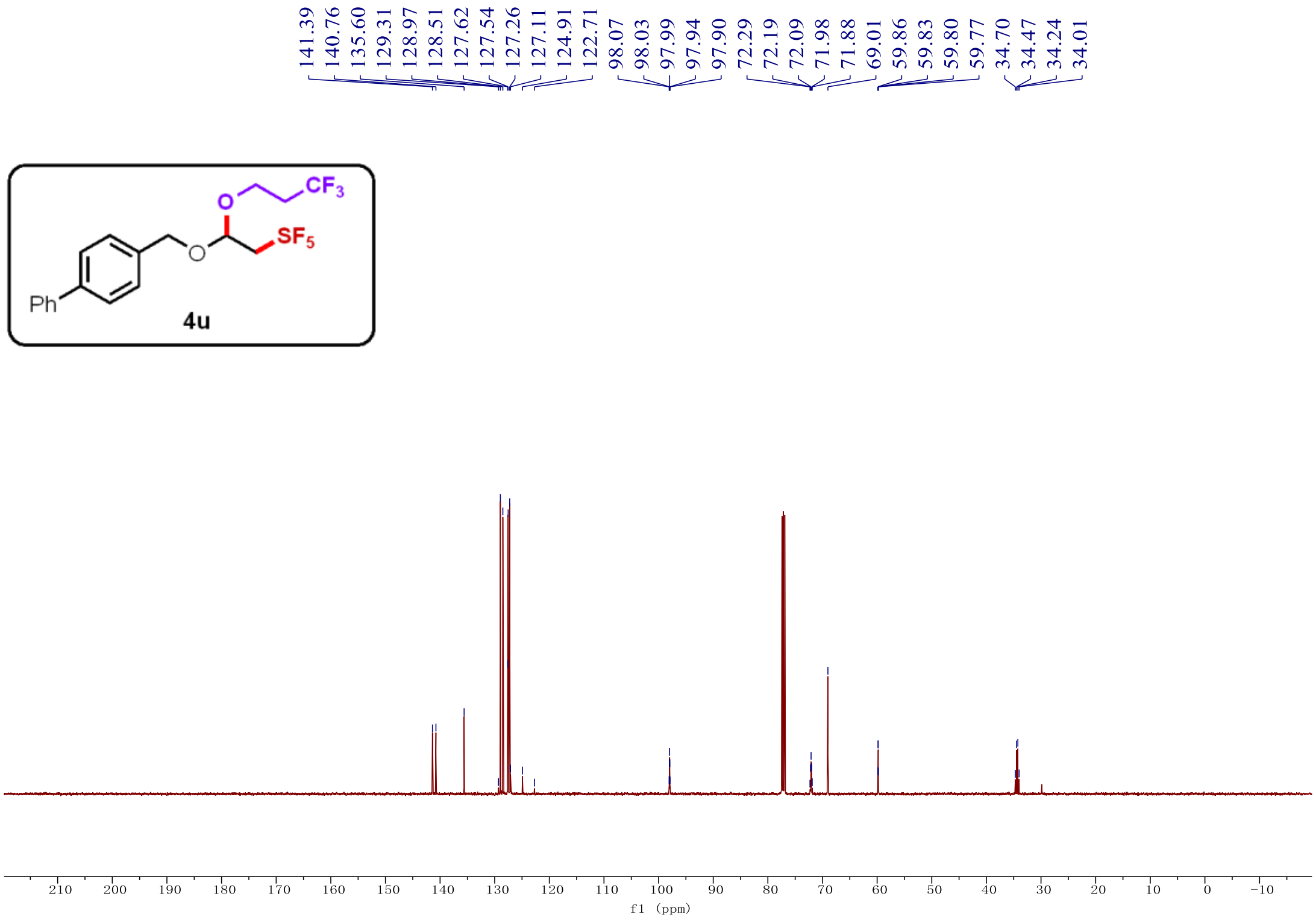

Supplementary Figure 155. ^{13}**C NMR Spectrum of Compound 4u (126 MHz, CDCl$_3$, 25 °C)**

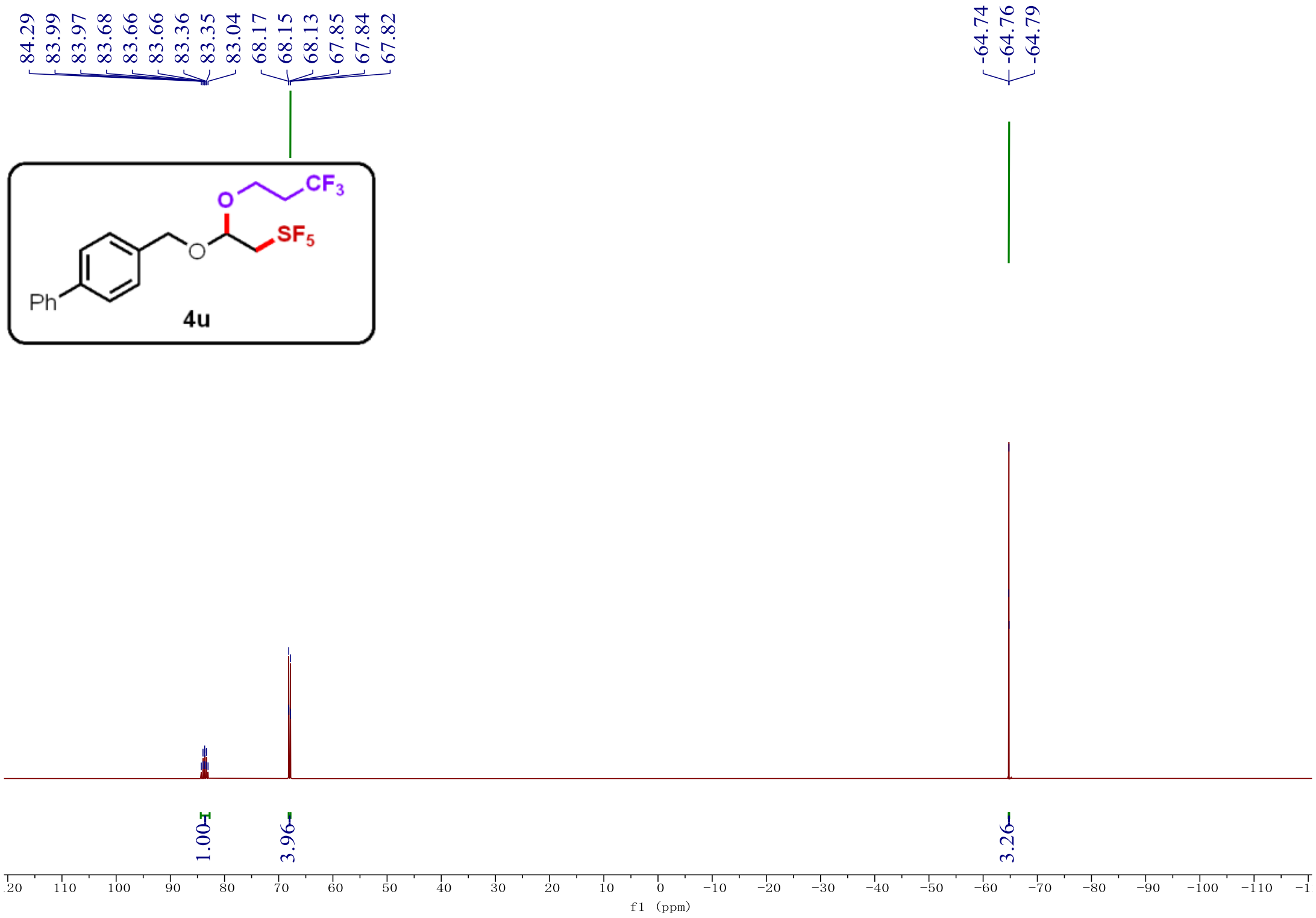

Supplementary Figure 156. ^{19}**F NMR Spectrum of Compound 4u (471 MHz, CDCl$_3$, 25 °C)**

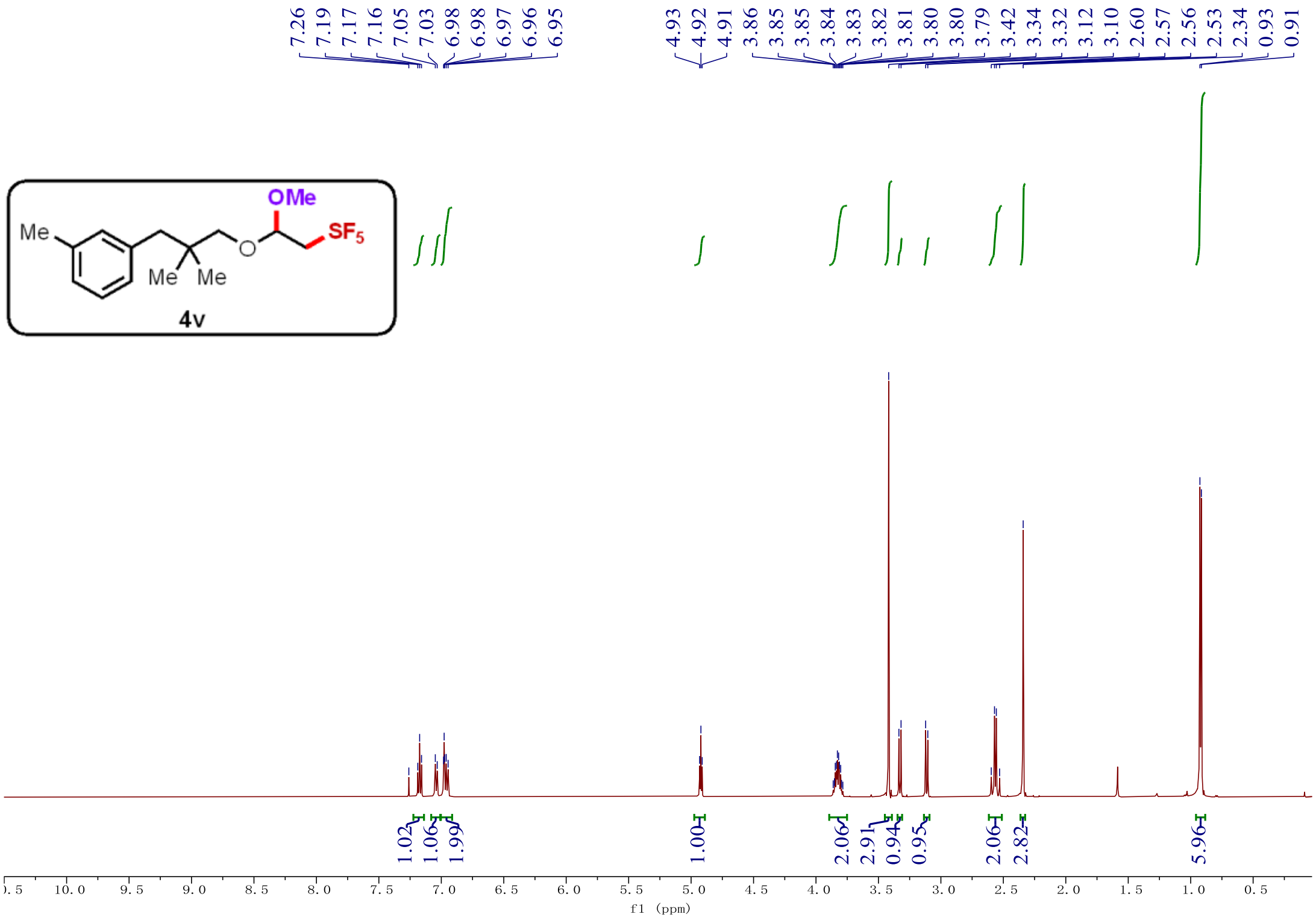

Supplementary Figure 157. **^{1}H NMR Spectrum of Compound 4v (500 MHz, $CDCl_3$, 25 °C)**

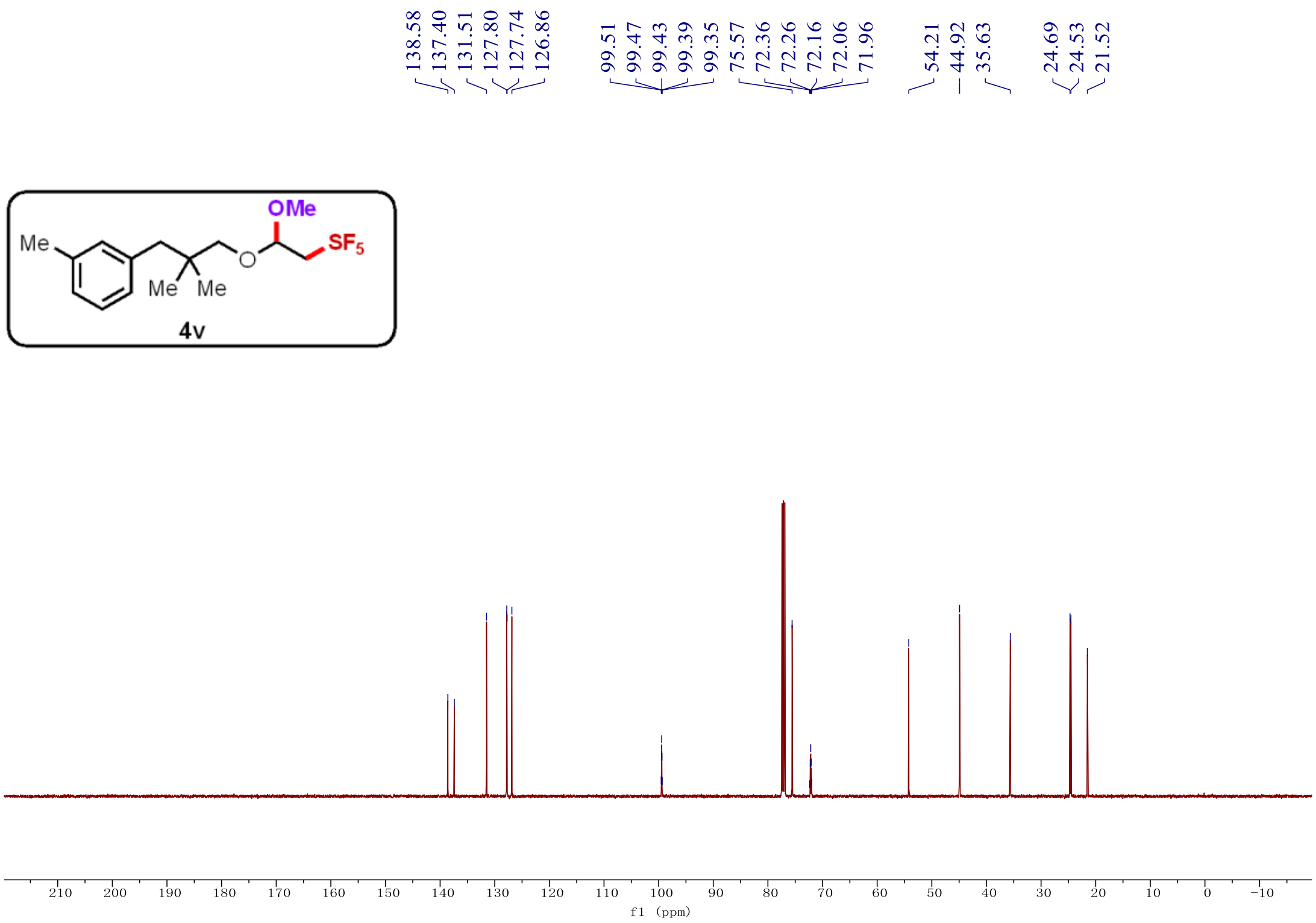

Supplementary Figure 158. **^{13}C NMR Spectrum of Compound 4v (126 MHz, $CDCl_3$, 25 °C)**

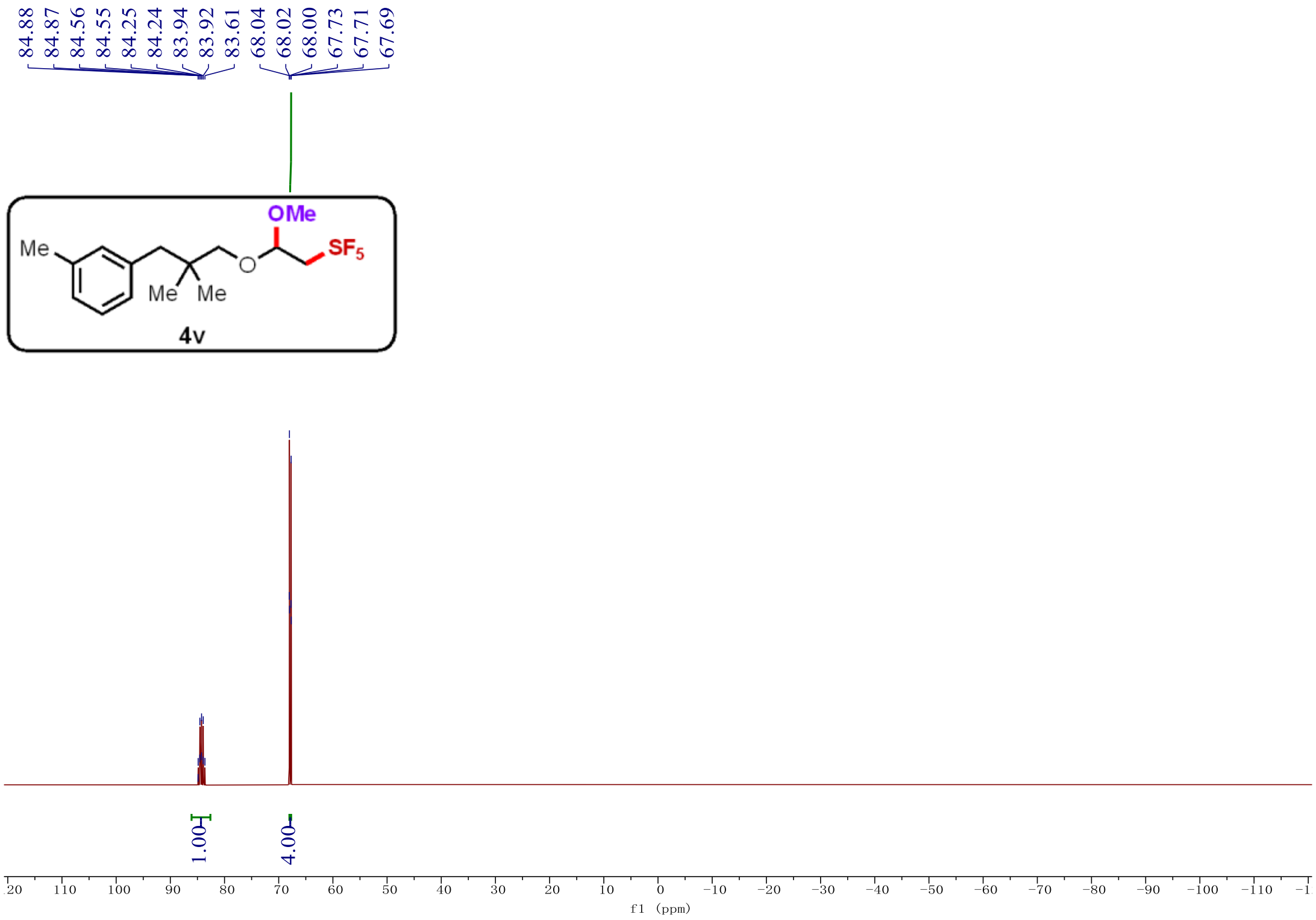

Supplementary Figure 159. ^{19}F NMR Spectrum of Compound 4v (471 MHz, $CDCl_3$, 25 ˚C)

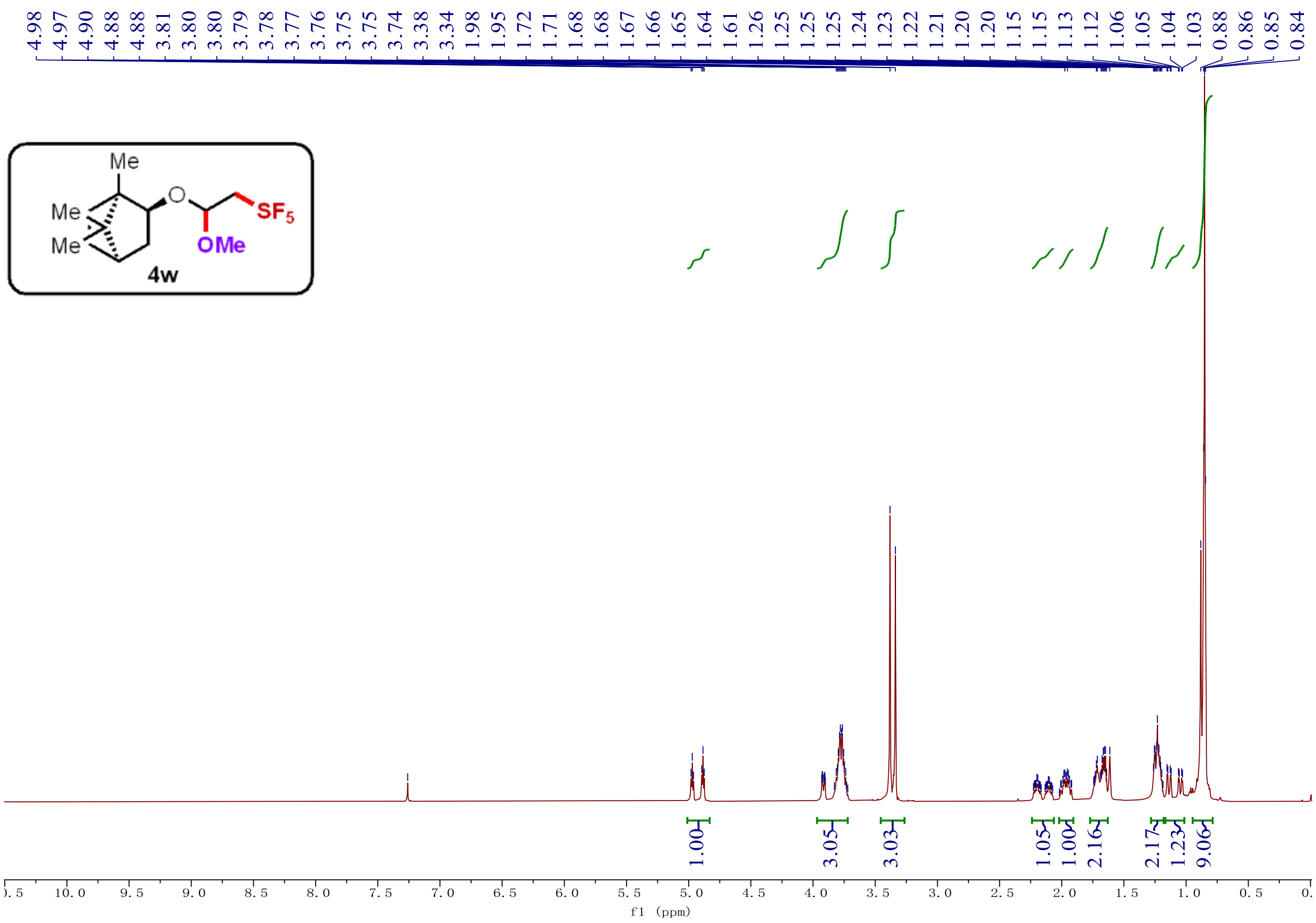

Supplementary Figure 160. ^{1}H NMR Spectrum of Compound 4 (500 MHz, $CDCl_3$, 25 ˚C)

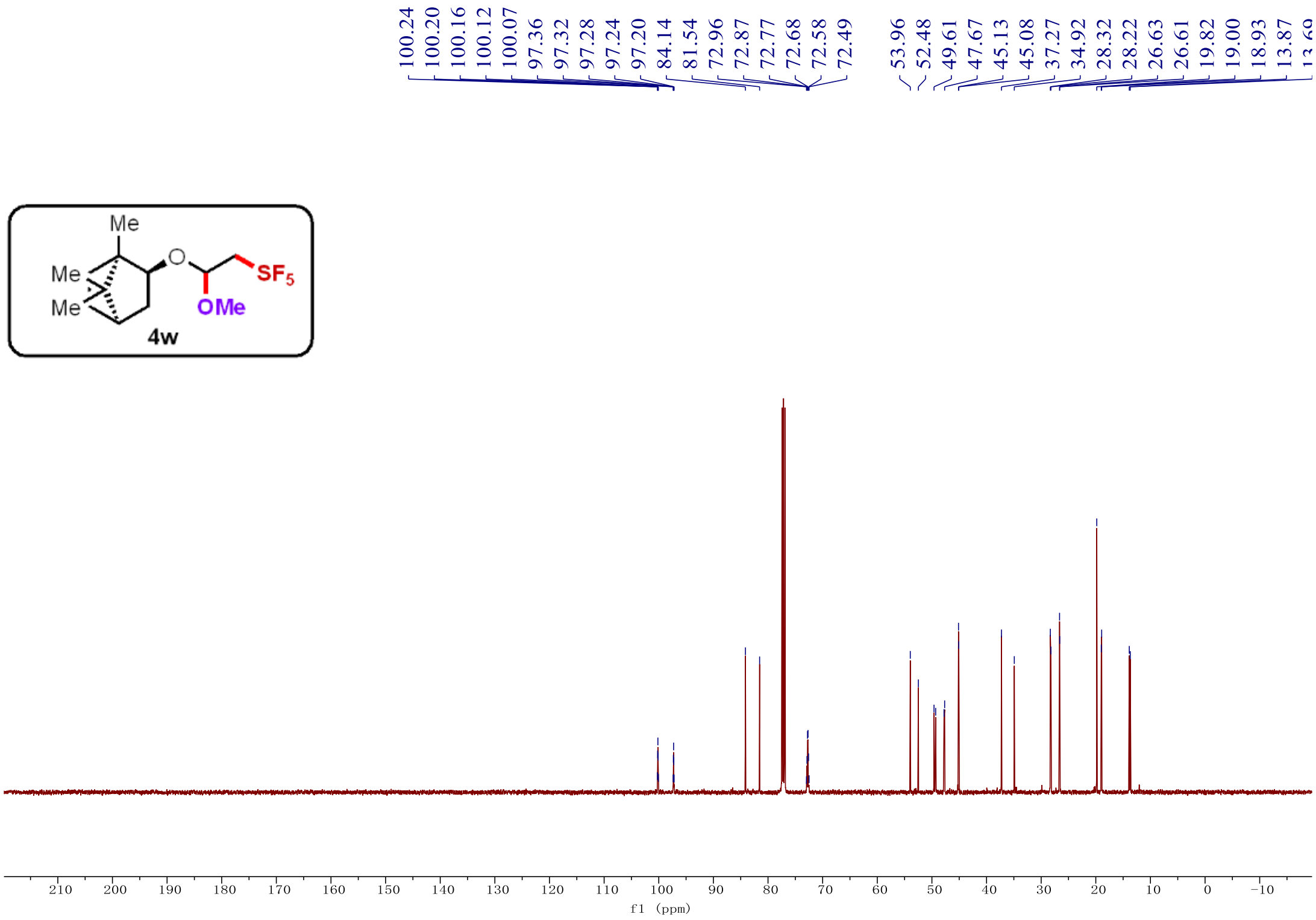

Supplementary Figure 161. ^{13}C NMR Spectrum of Compound 4 (126 MHz, $CDCl_3$, 25 ˚C)

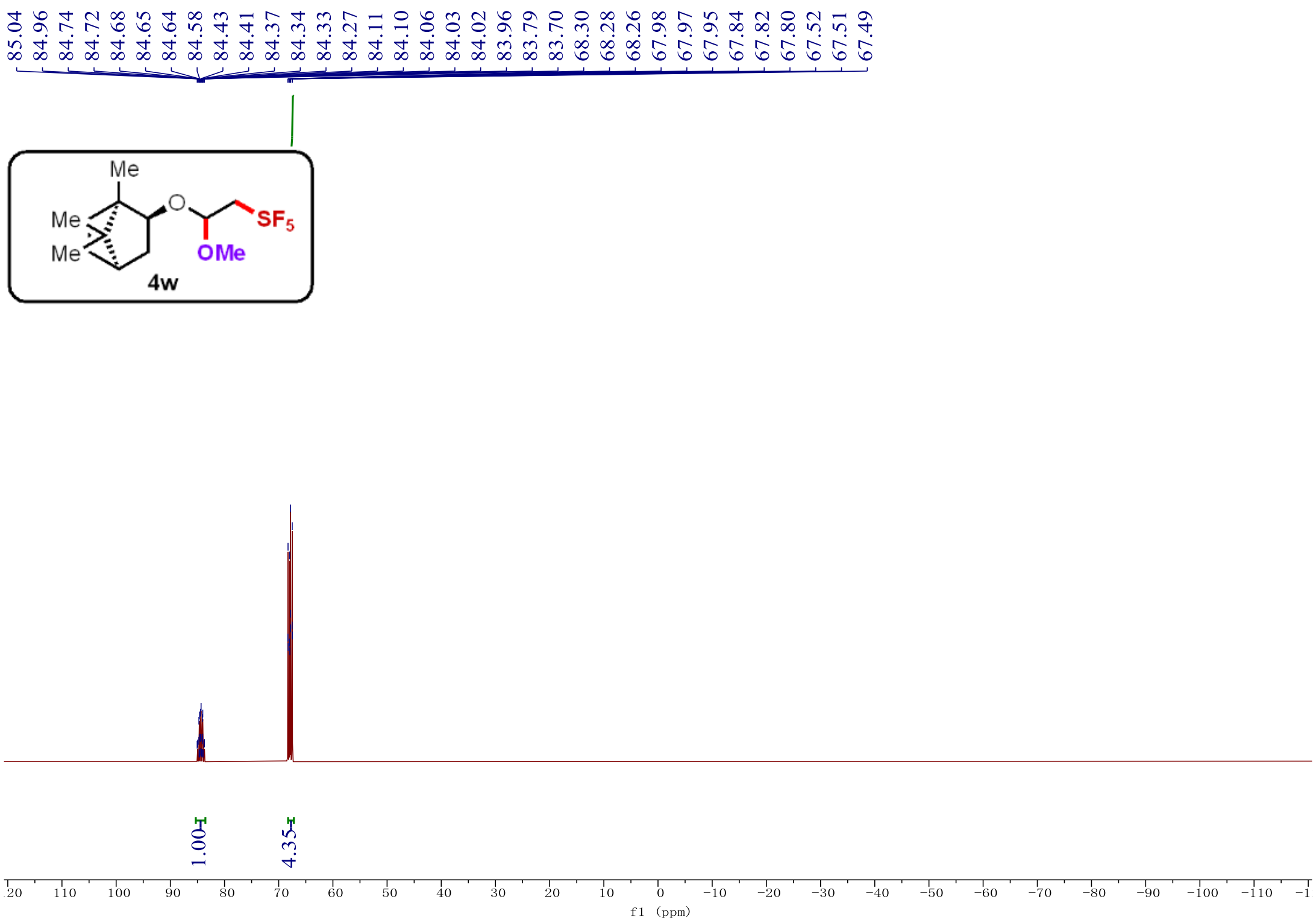

Supplementary Figure 162. ^{19}F NMR Spectrum of Compound 4 (471 MHz, $CDCl_3$, 25 ˚C)

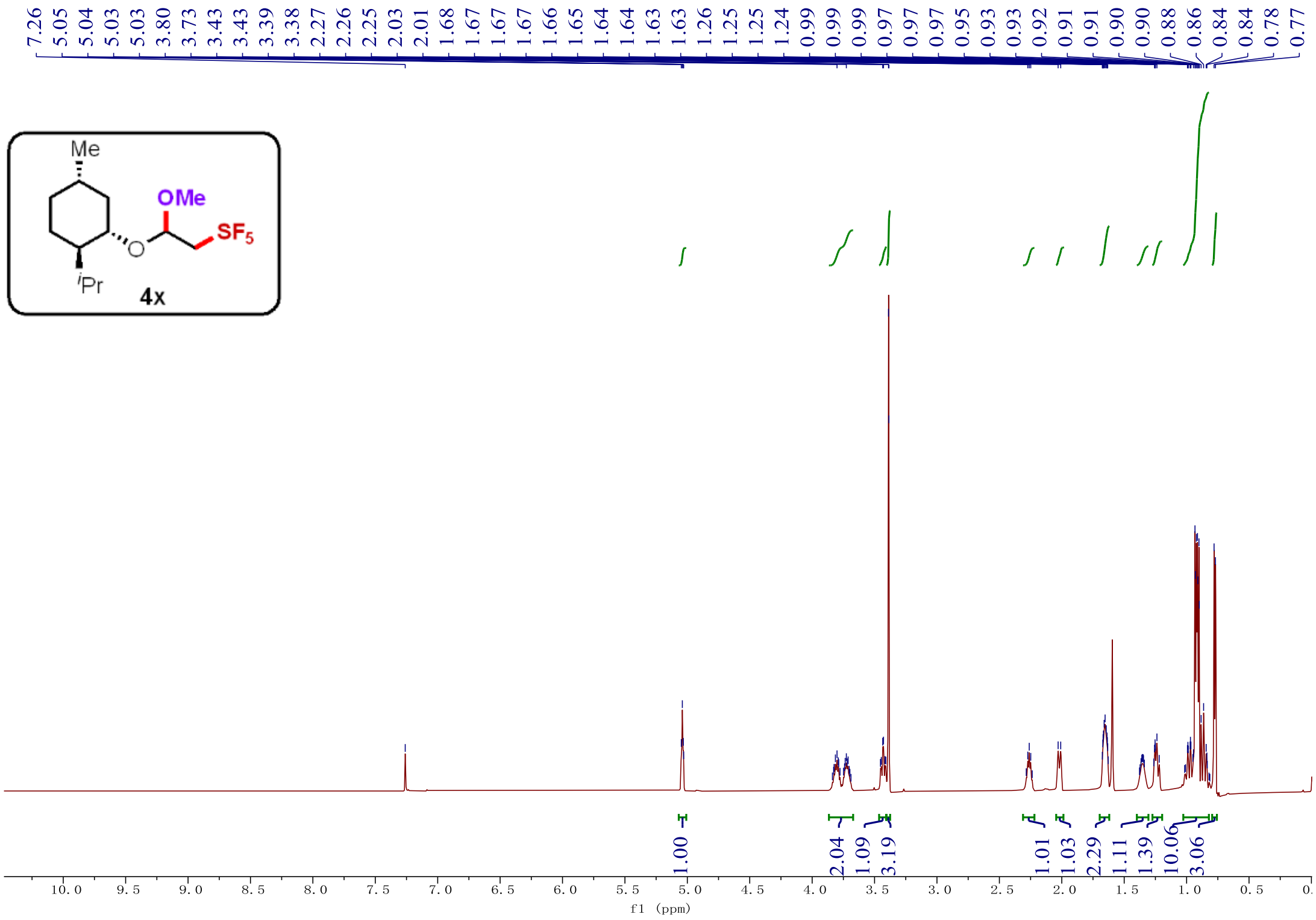

Supplementary Figure 163. ^{1}H NMR Spectrum of Compound 4x (600 MHz, $CDCl_3$, 25 ˚C)

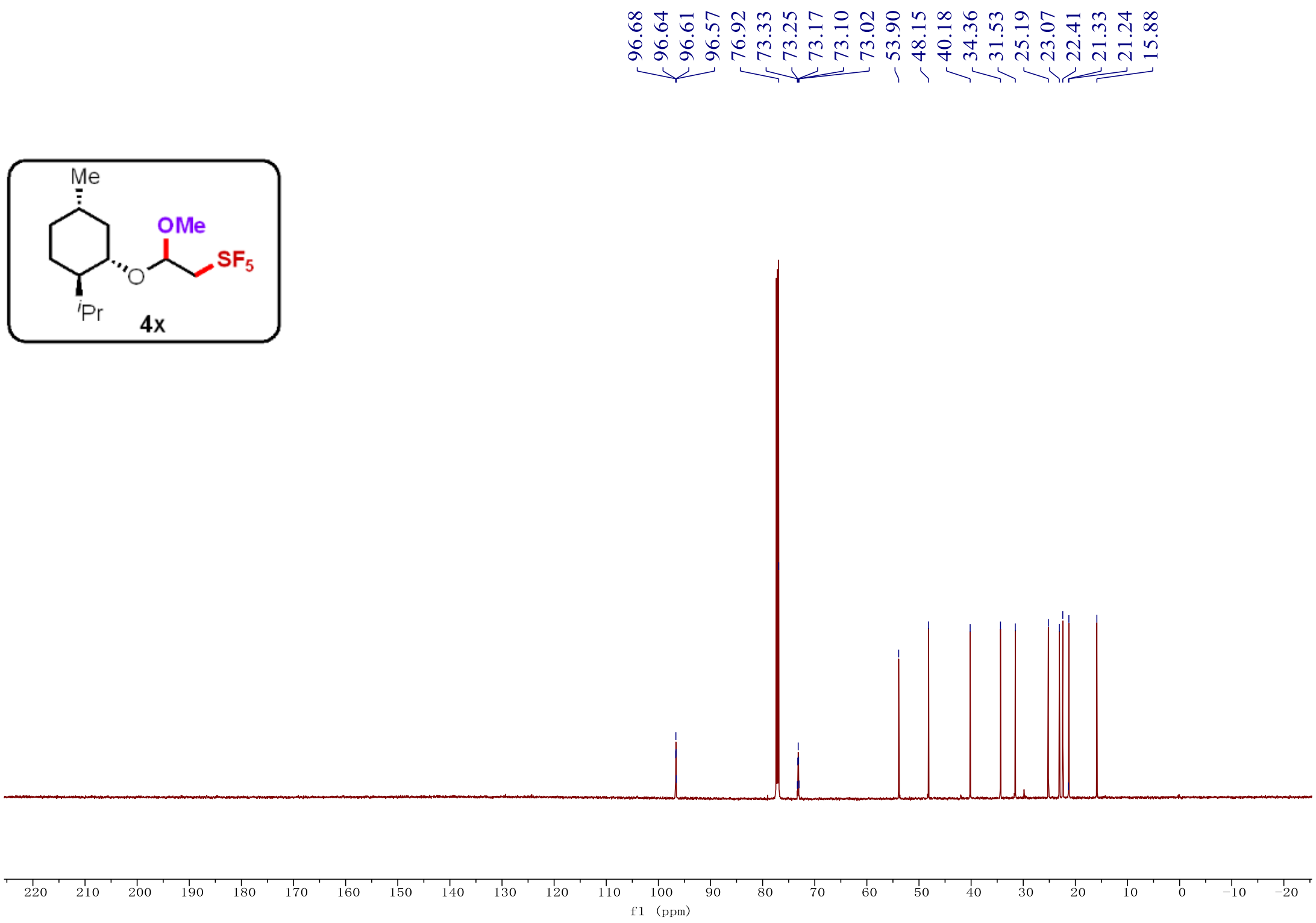

Supplementary Figure 164. ^{13}C NMR Spectrum of Compound 4x (151 MHz, $CDCl_3$, 25 ˚C)

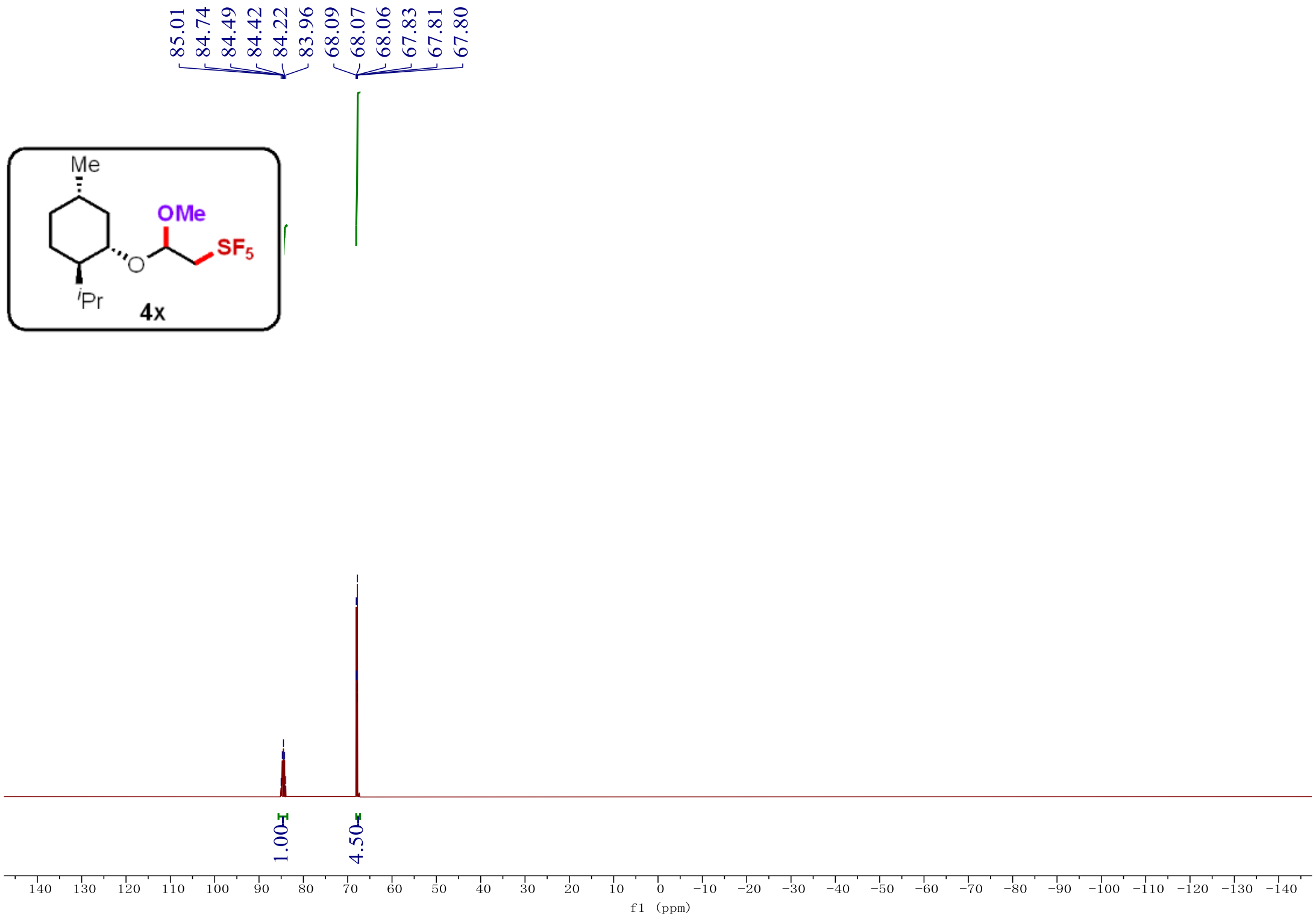

Supplementary Figure 165. ^{19}F NMR Spectrum of Compound 4x (565 MHz, $CDCl_3$, 25 ˚C)

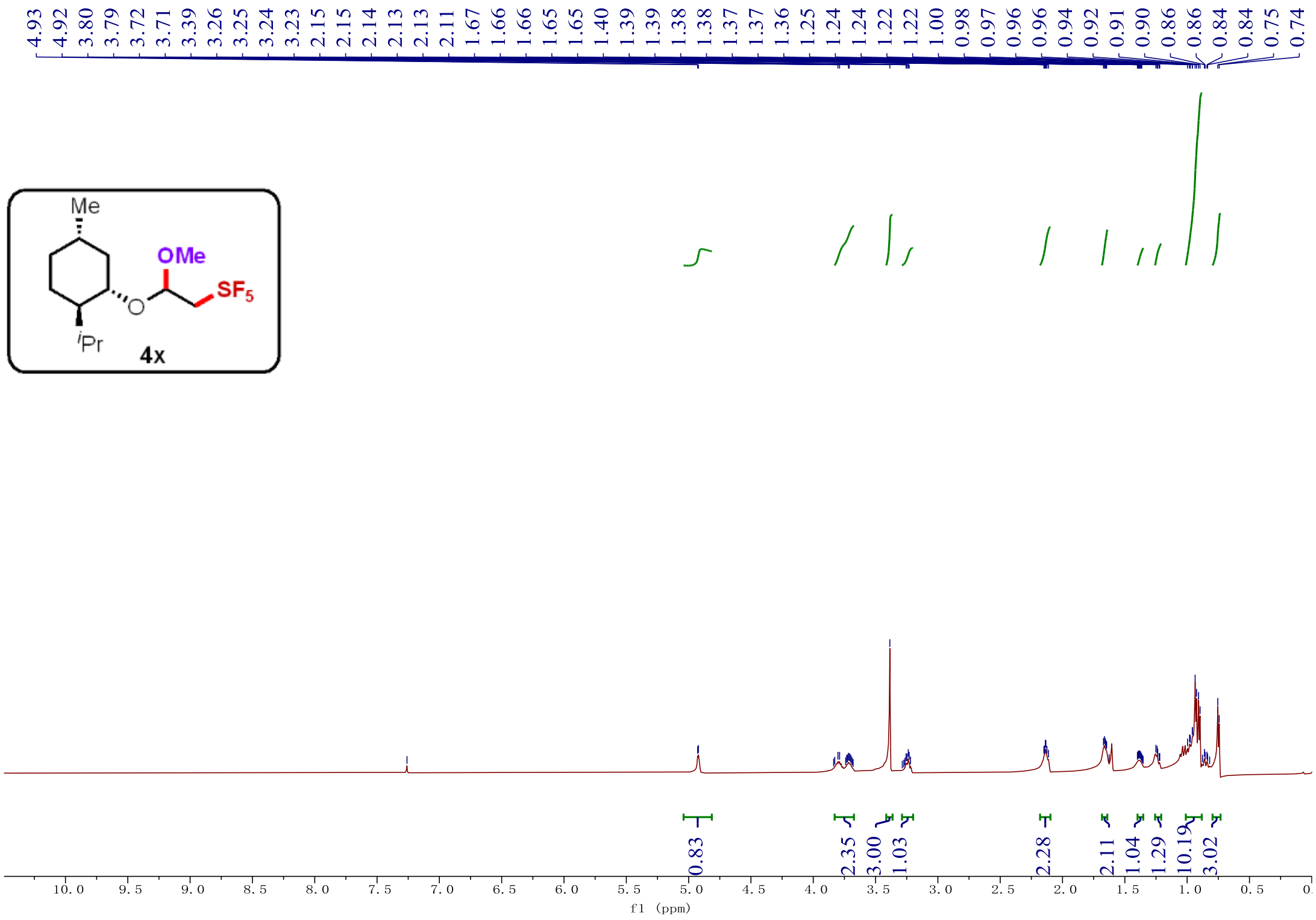

Supplementary Figure 166. ^{1}H NMR Spectrum of Compound 4x (600 MHz, $CDCl_3$, 25 ˚C)

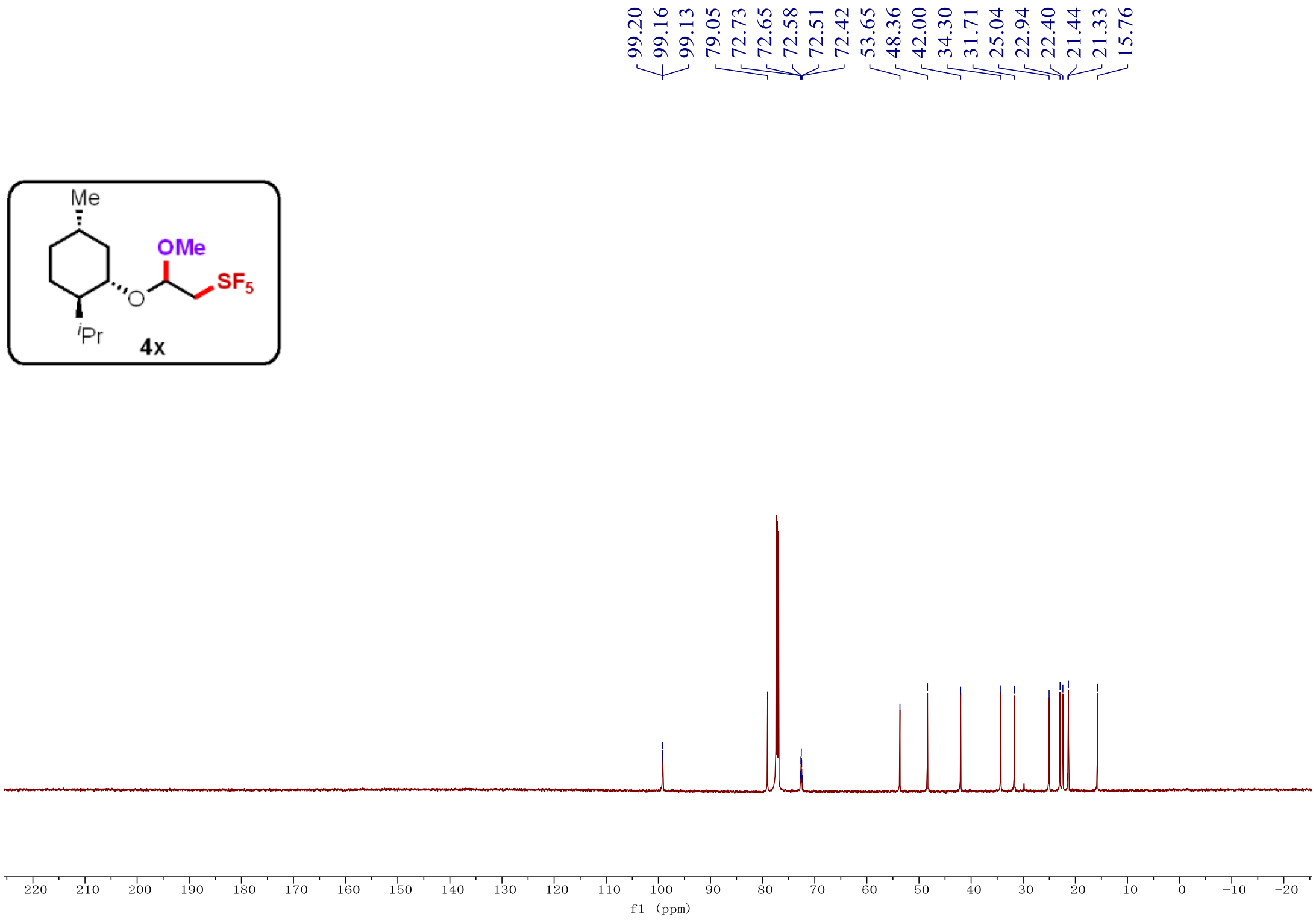

Supplementary Figure 167. ^{13}**C NMR Spectrum of Compound 4x (151 MHz, CDCl$_3$, 25 ˚C)**

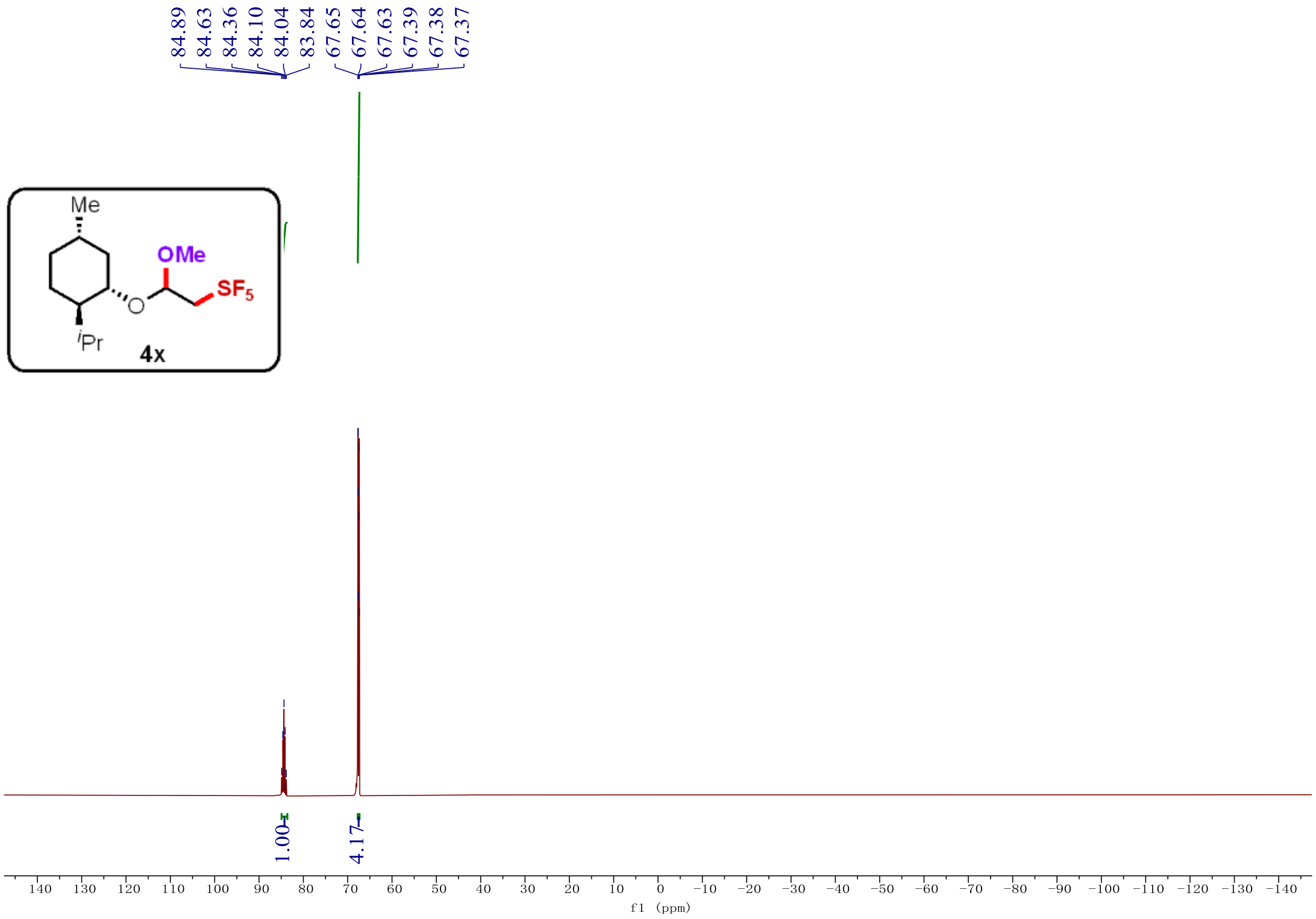

Supplementary Figure 168. ^{19}**F NMR Spectrum of Compound 4x (565 MHz, CDCl$_3$, 25 ˚C)**

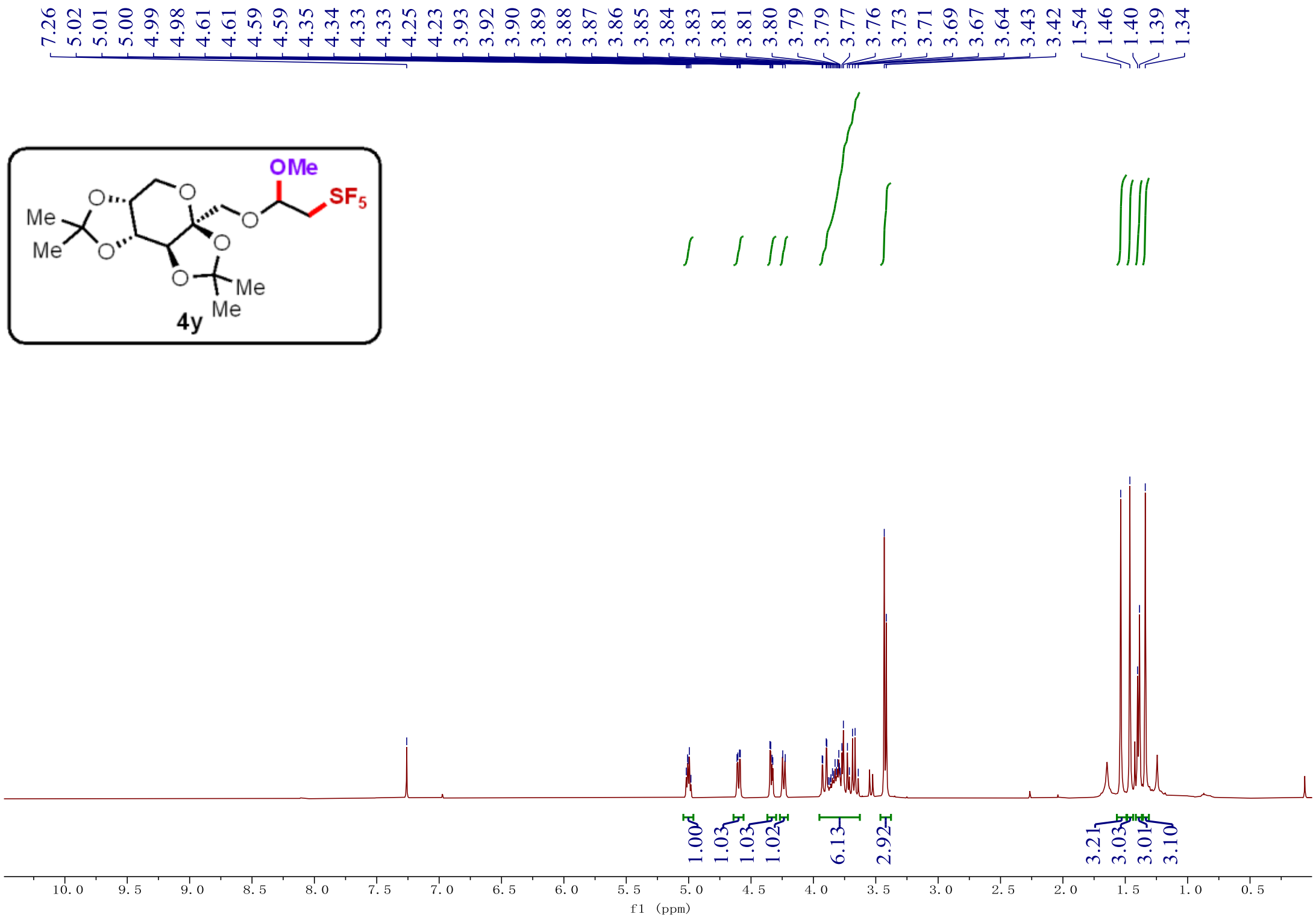

Supplementary Figure 169. ^{1}**H NMR Spectrum of Compound 4y (400 MHz, CDCl$_3$, 25 °C)**

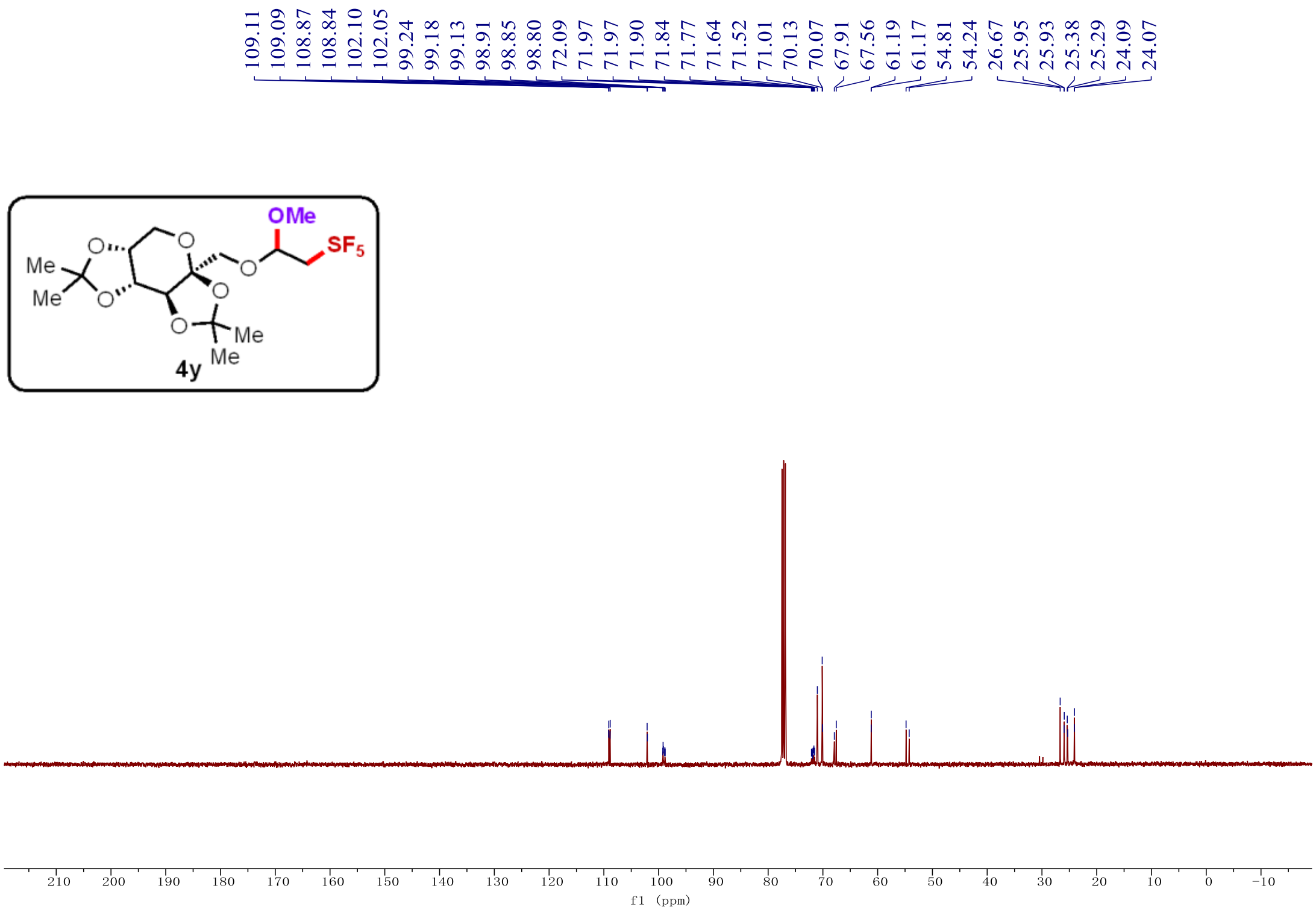

Supplementary Figure 170. ^{13}**C NMR Spectrum of Compound 4y (101 MHz, CDCl$_3$, 25 °C)**

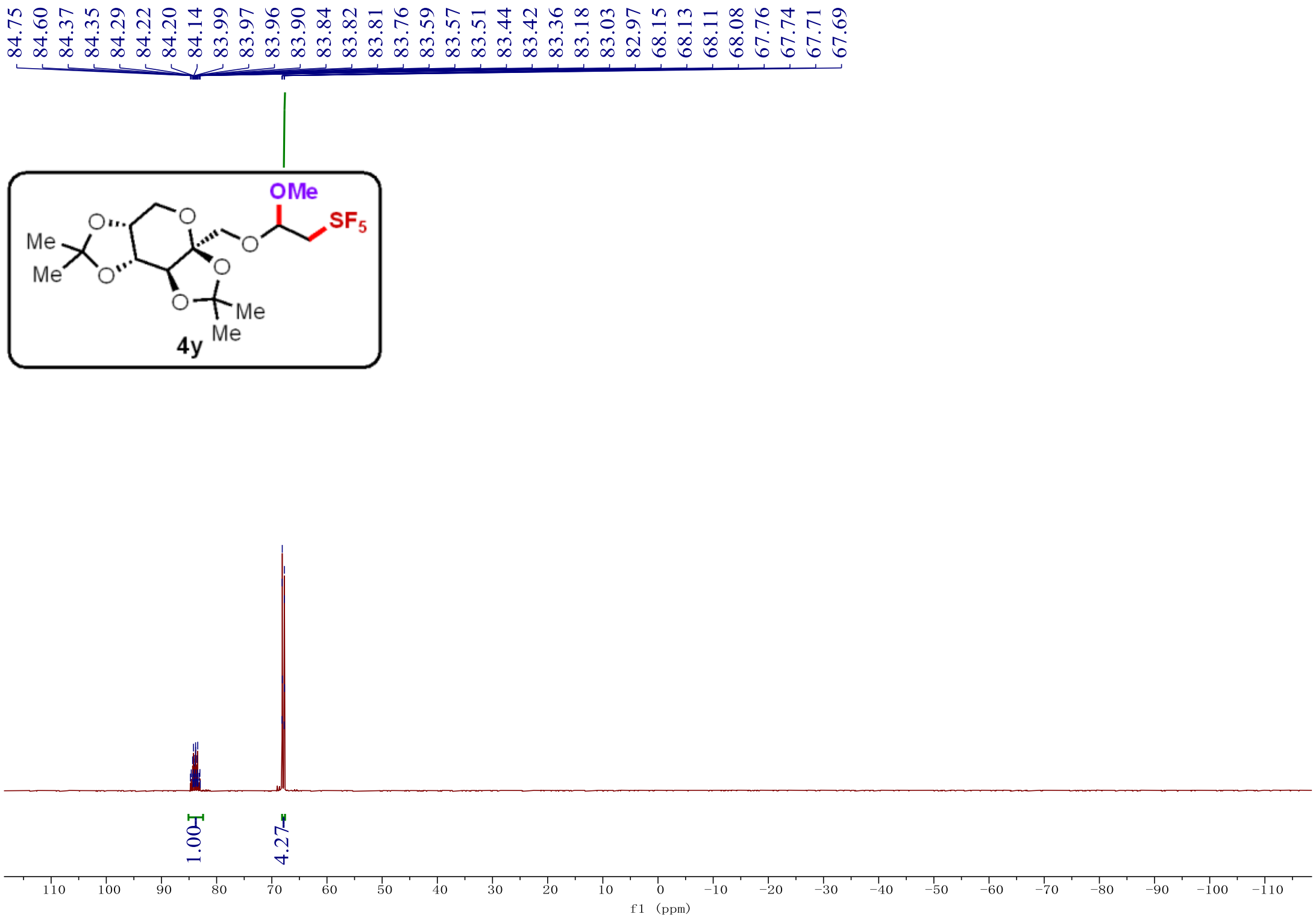

Supplementary Figure 171. ^{19}F NMR Spectrum of Compound 4y (376 MHz, $CDCl_3$, 25 ˚C)

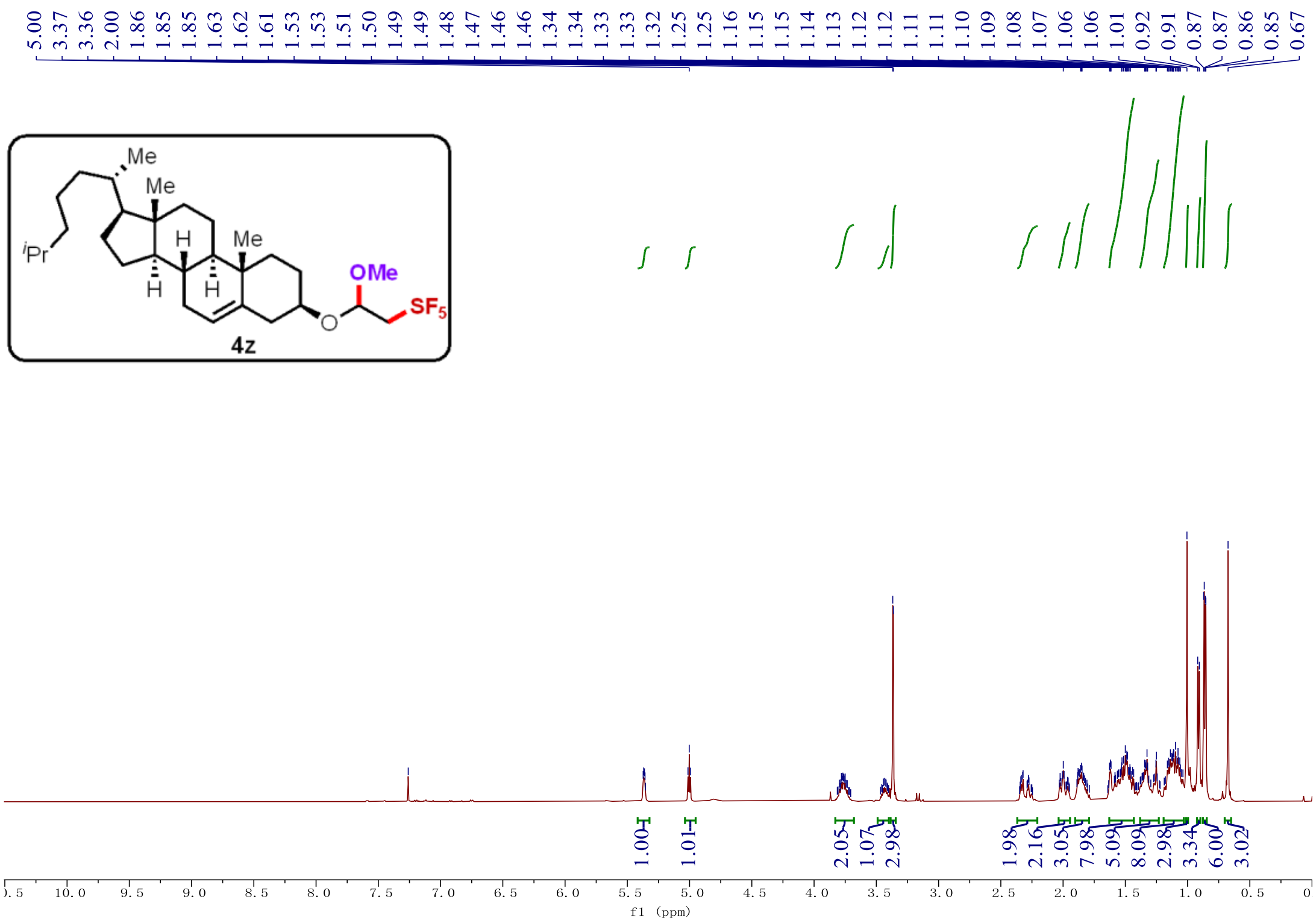

Supplementary Figure 172. ^{1}H NMR Spectrum of Compound 4z (500 MHz, $CDCl_3$, 25 ˚C)

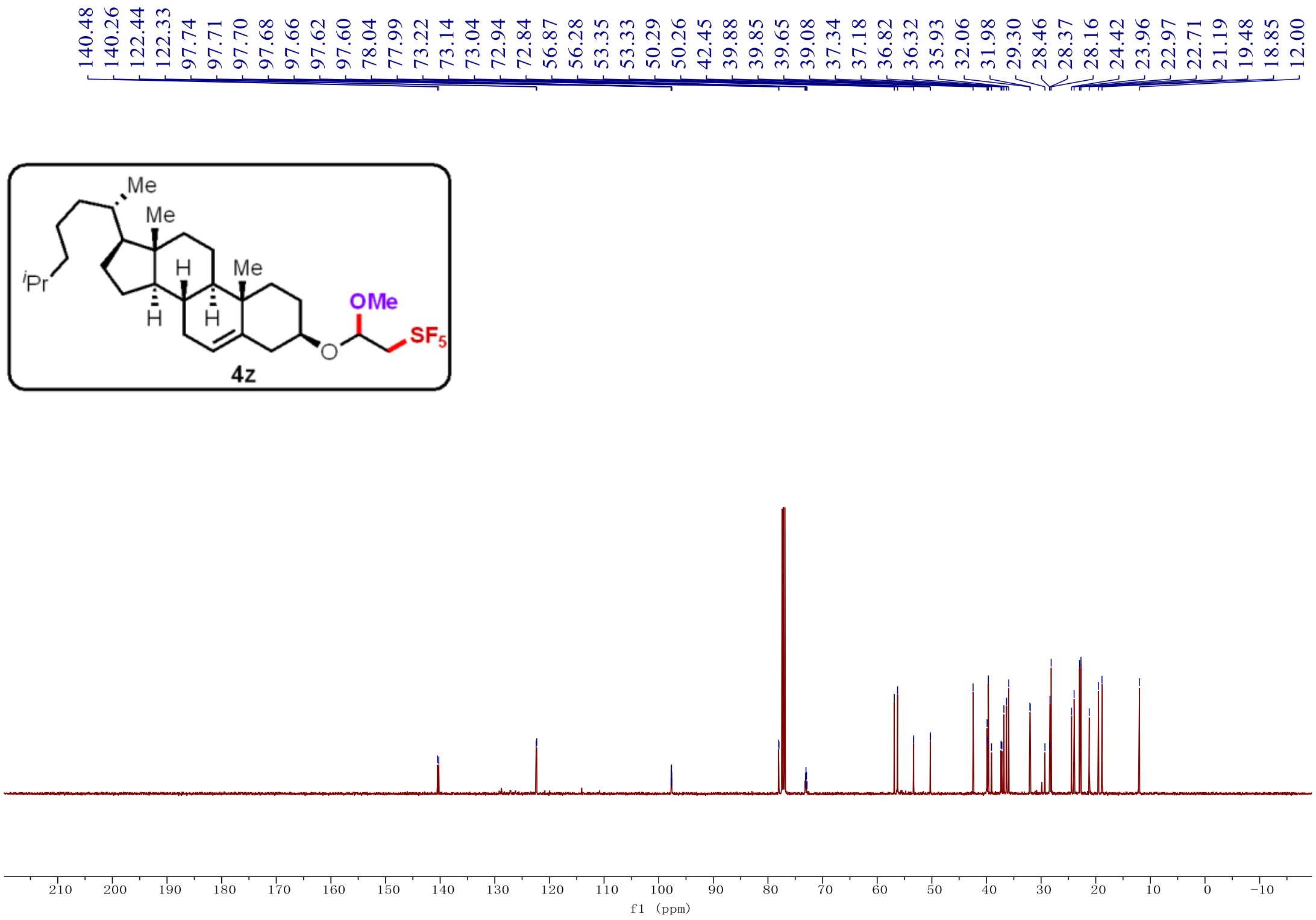

Supplementary Figure 173. ^{13}C NMR Spectrum of Compound 4z (126 MHz, $CDCl_3$, 25 °C)

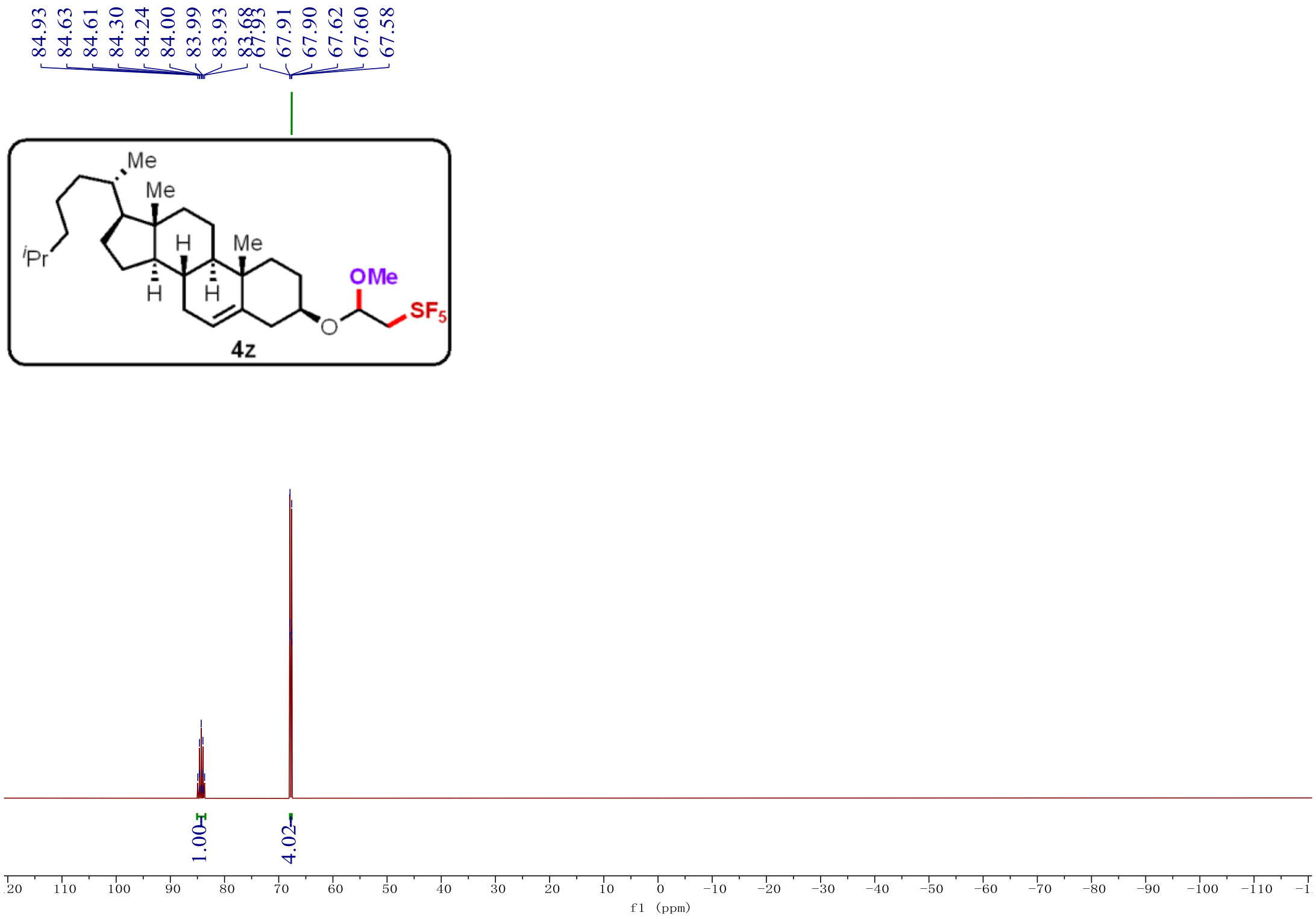

Supplementary Figure 174. ^{19}F NMR Spectrum of Compound 4z (471 MHz, $CDCl_3$, 25 °C)

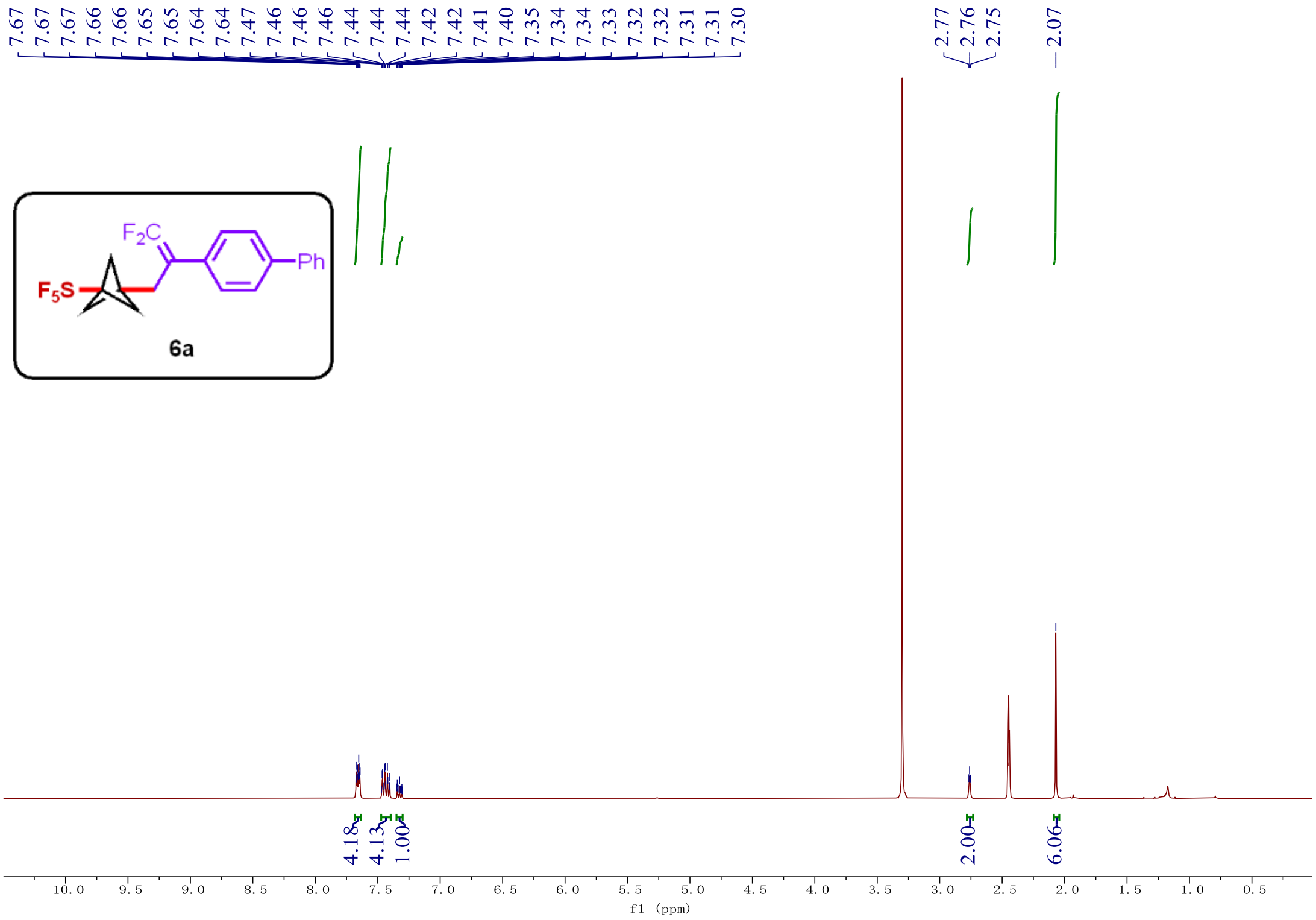

Supplementary Figure 175. ^{1}**H NMR Spectrum of Compound 6a (400 MHz, DMSO, 25 ˚C)**

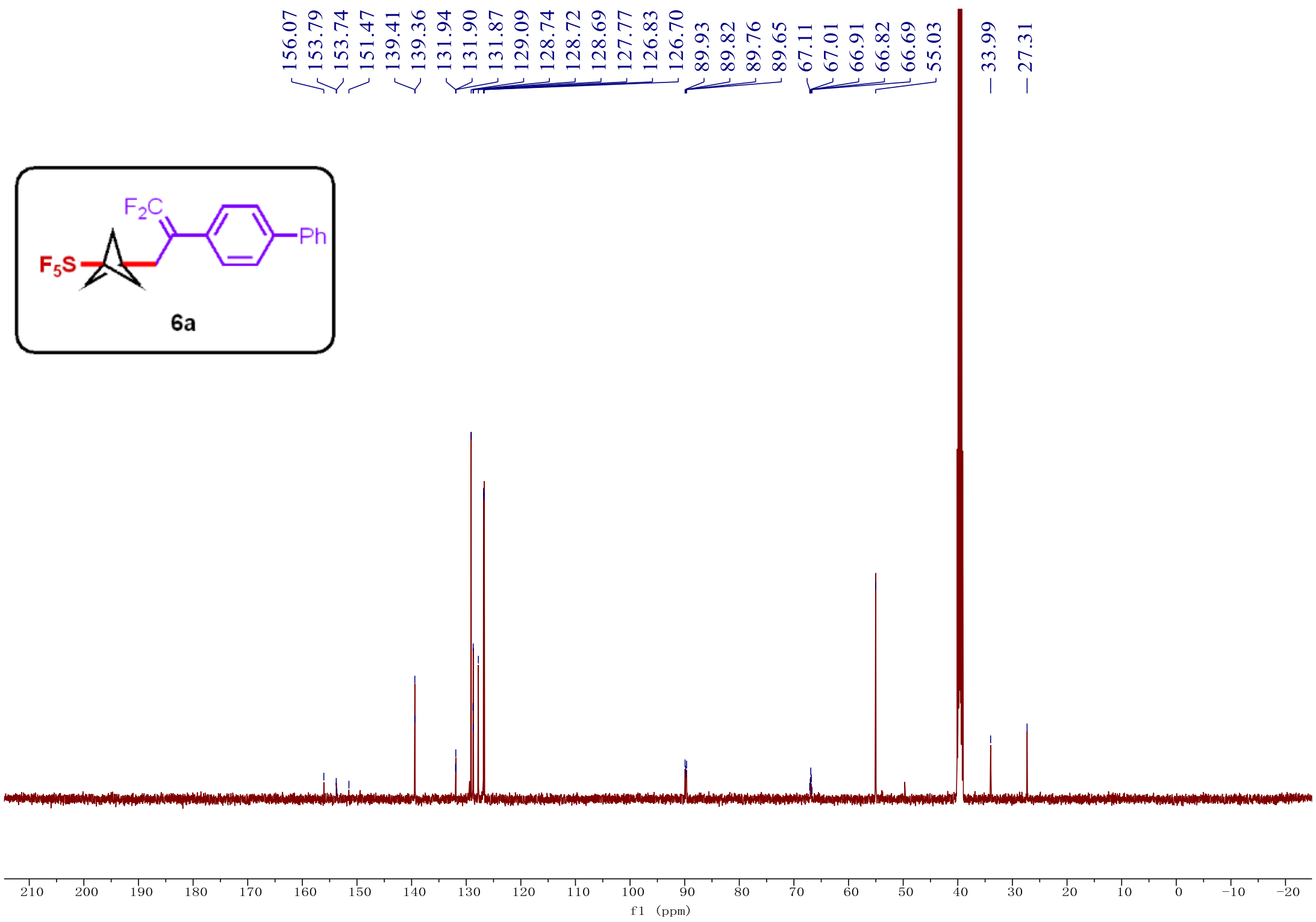

Supplementary Figure 176. ^{13}**C NMR Spectrum of Compound 6a (126 MHz, DMSO, 25 ˚C)**

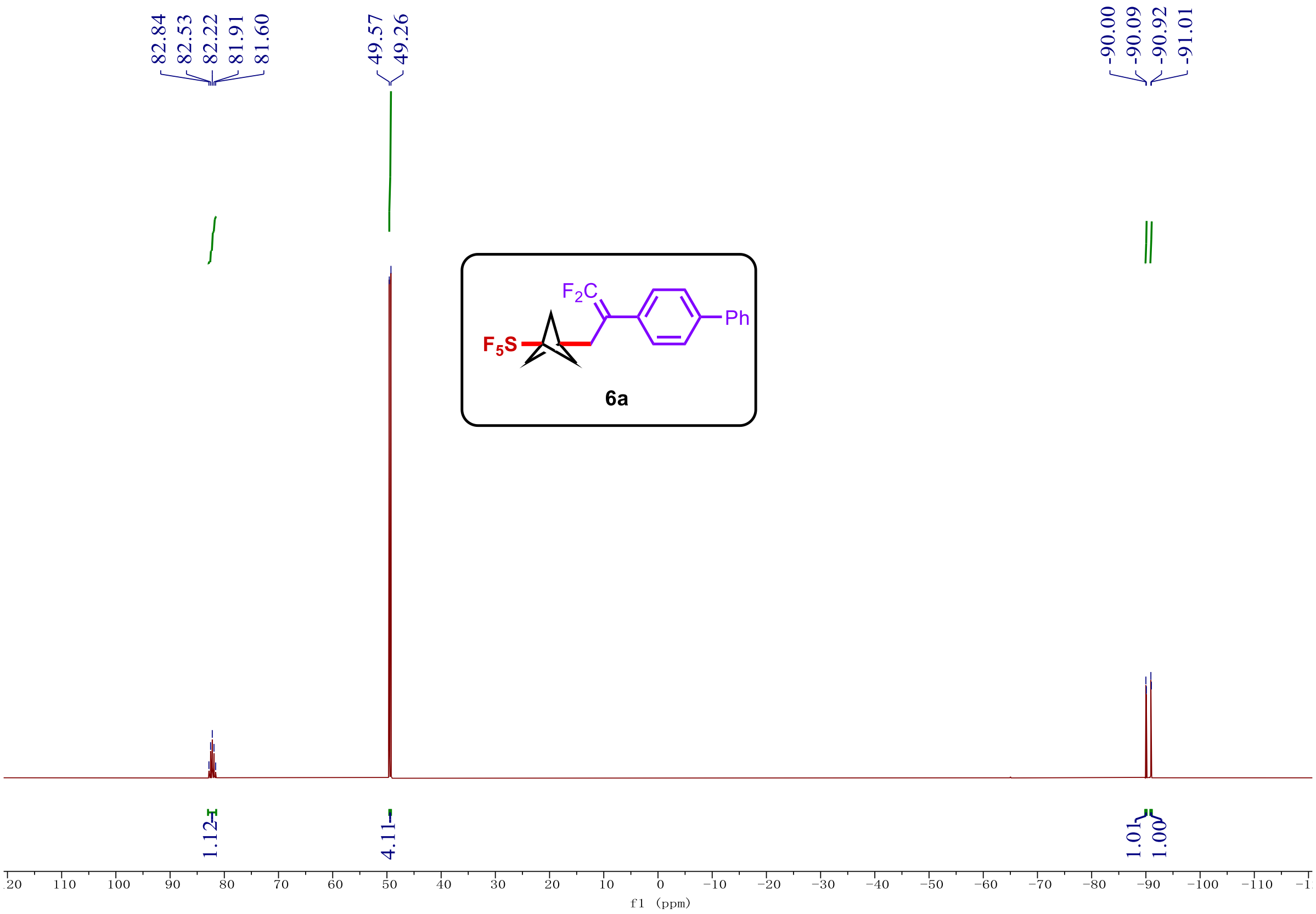

Supplementary Figure 177. ^{19}F NMR Spectrum of Compound 6a (471 MHz, DMSO, 25 °C)

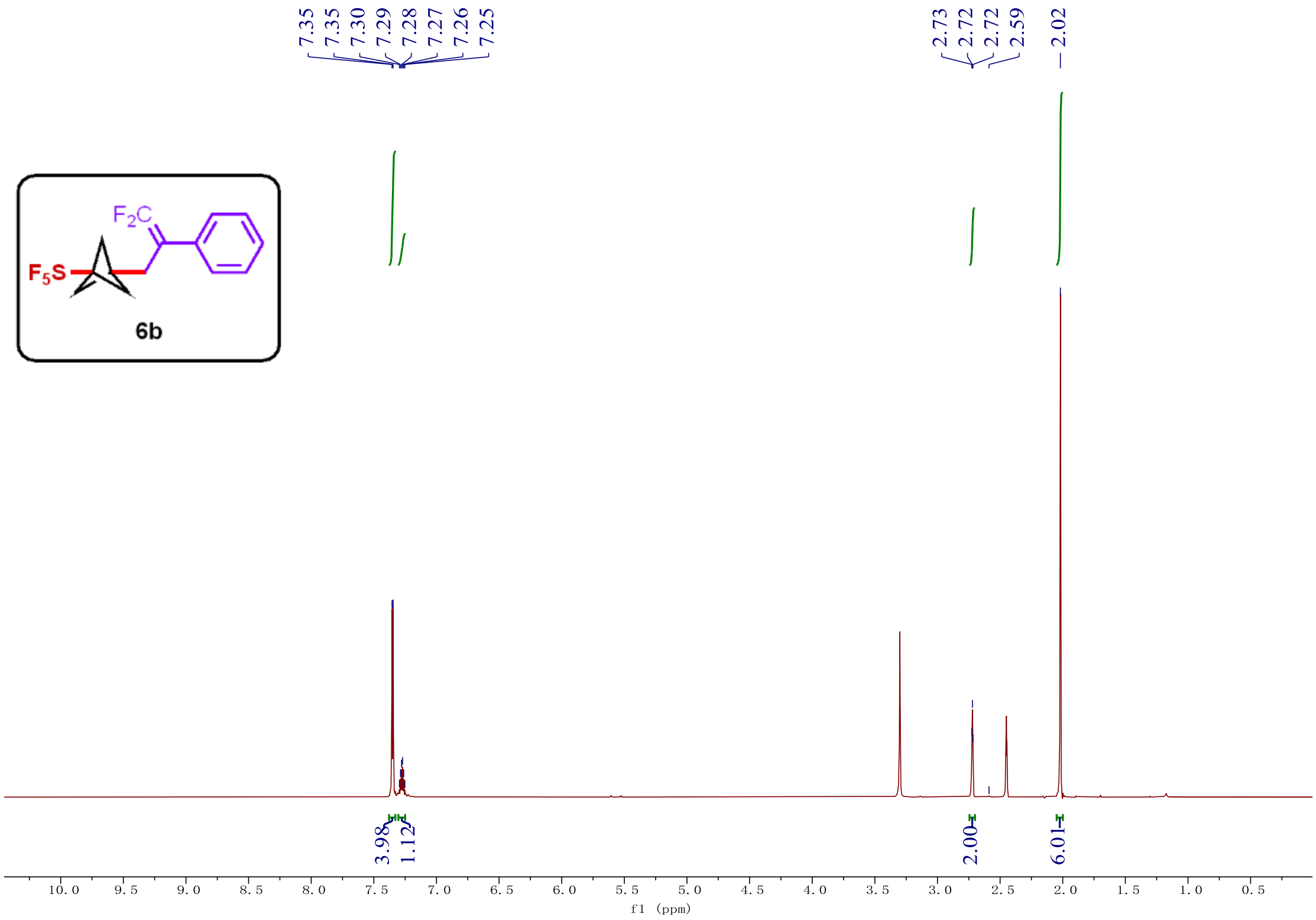

Supplementary Figure 178. ^{1}H NMR Spectrum of Compound 6b (500 MHz, DMSO, 25 °C)

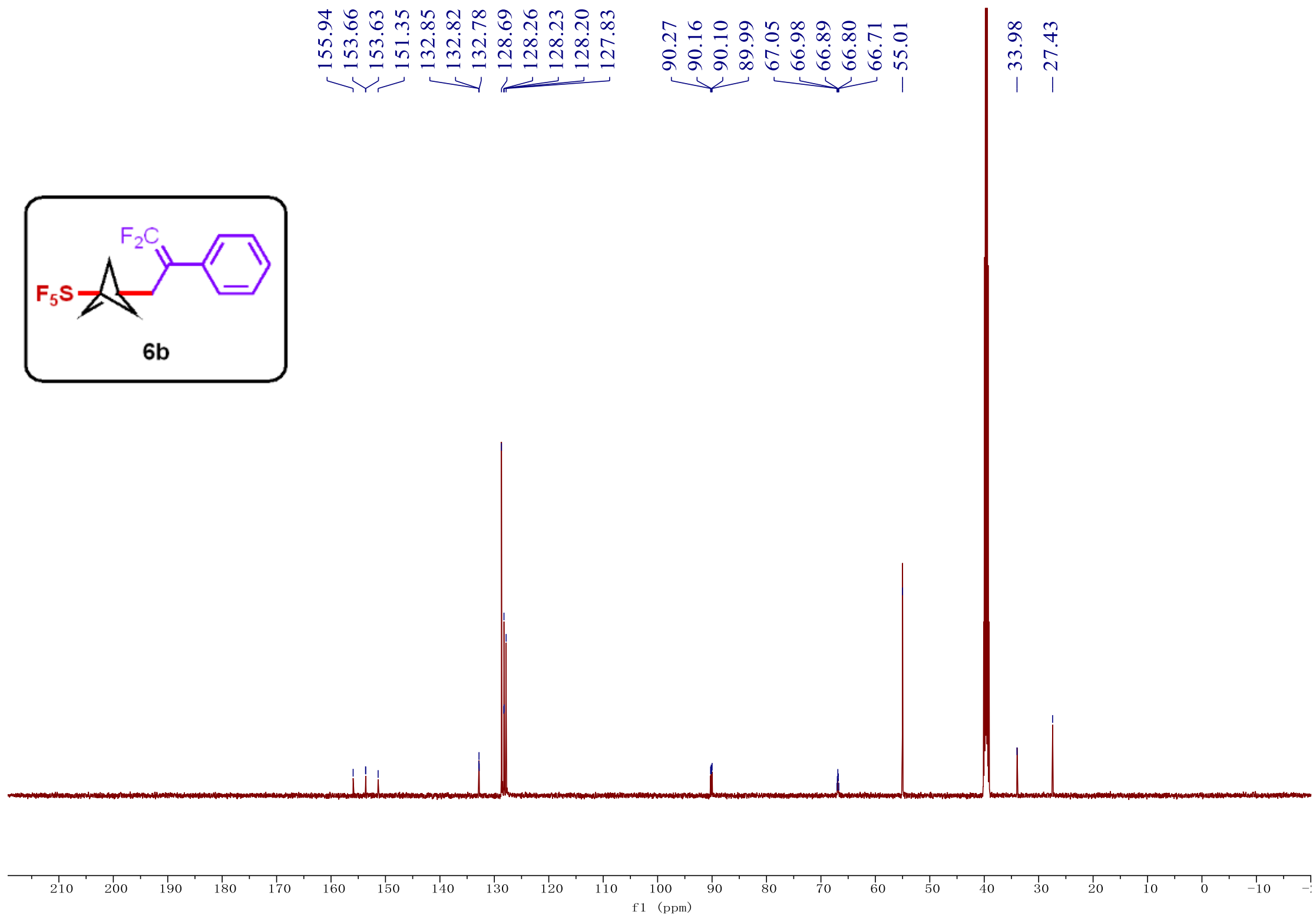

Supplementary Figure 179. ^{13}C NMR Spectrum of Compound 6b (126 MHz, DMSO, 25 ˚C)

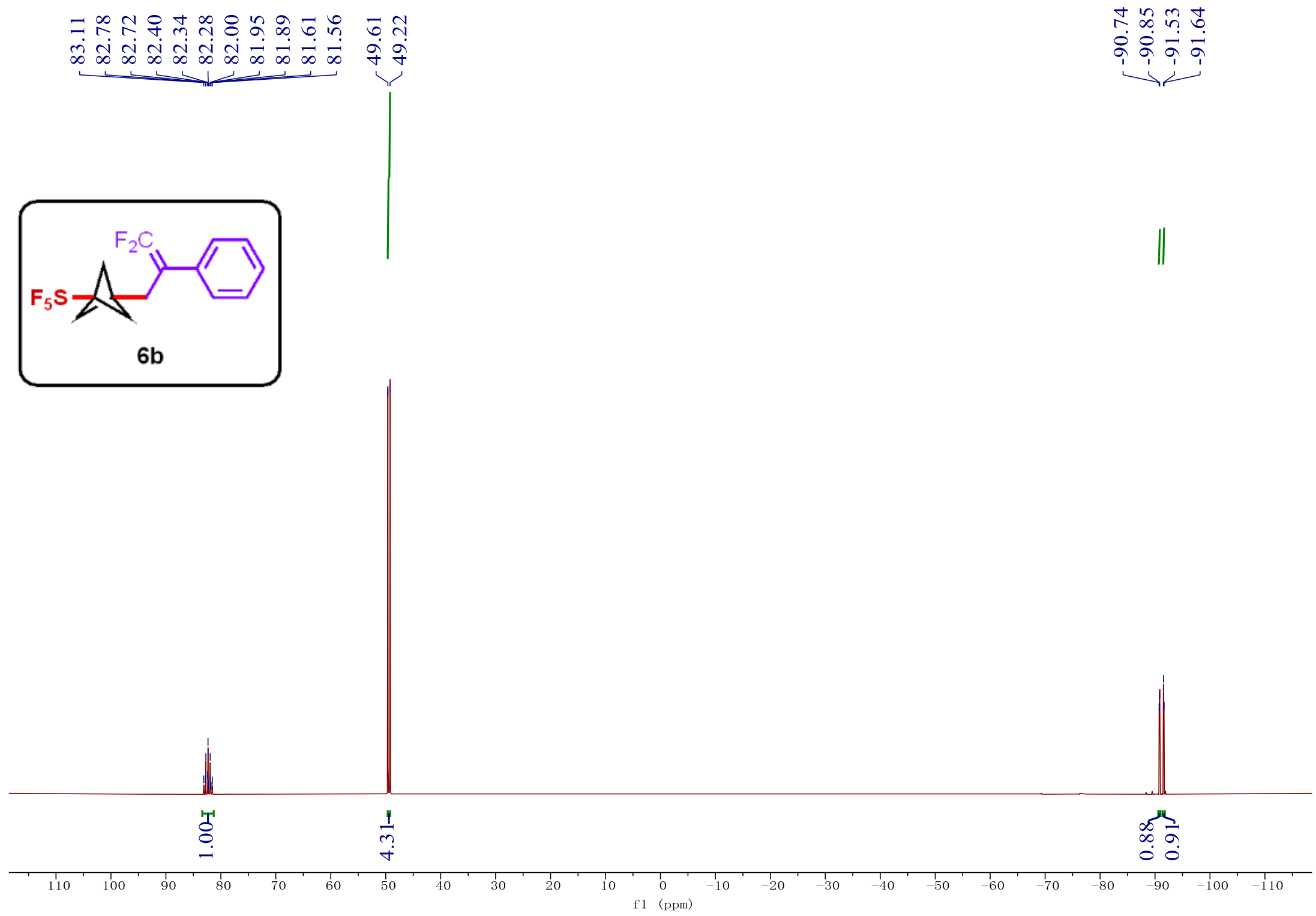

Supplementary Figure 180. ^{19}F NMR Spectrum of Compound 6b (376 MHz, DMSO, 25 ˚C)

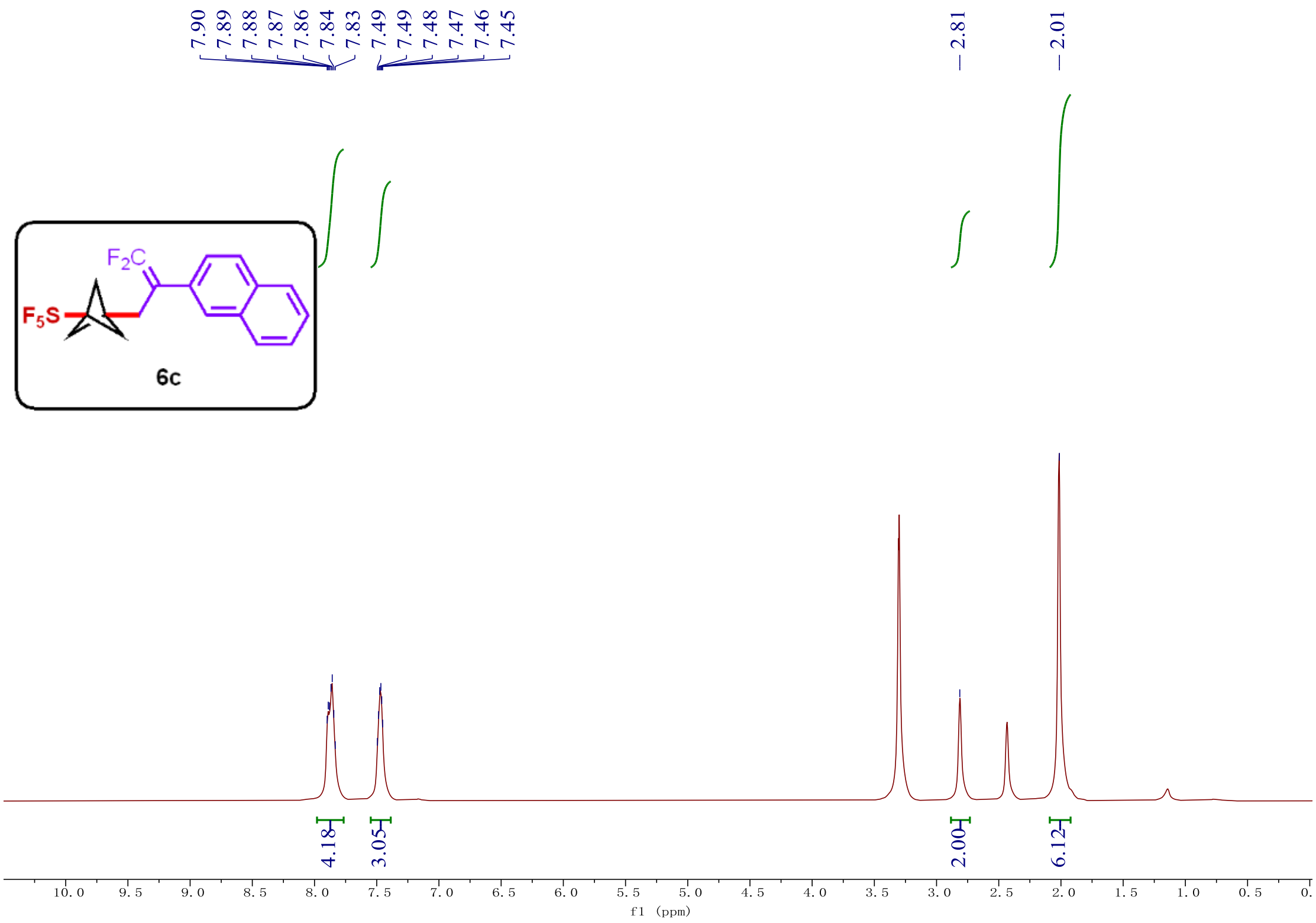

Supplementary Figure 181. ¹H NMR Spectrum of Compound 6c (400 MHz, DMSO, 25 ˚C)

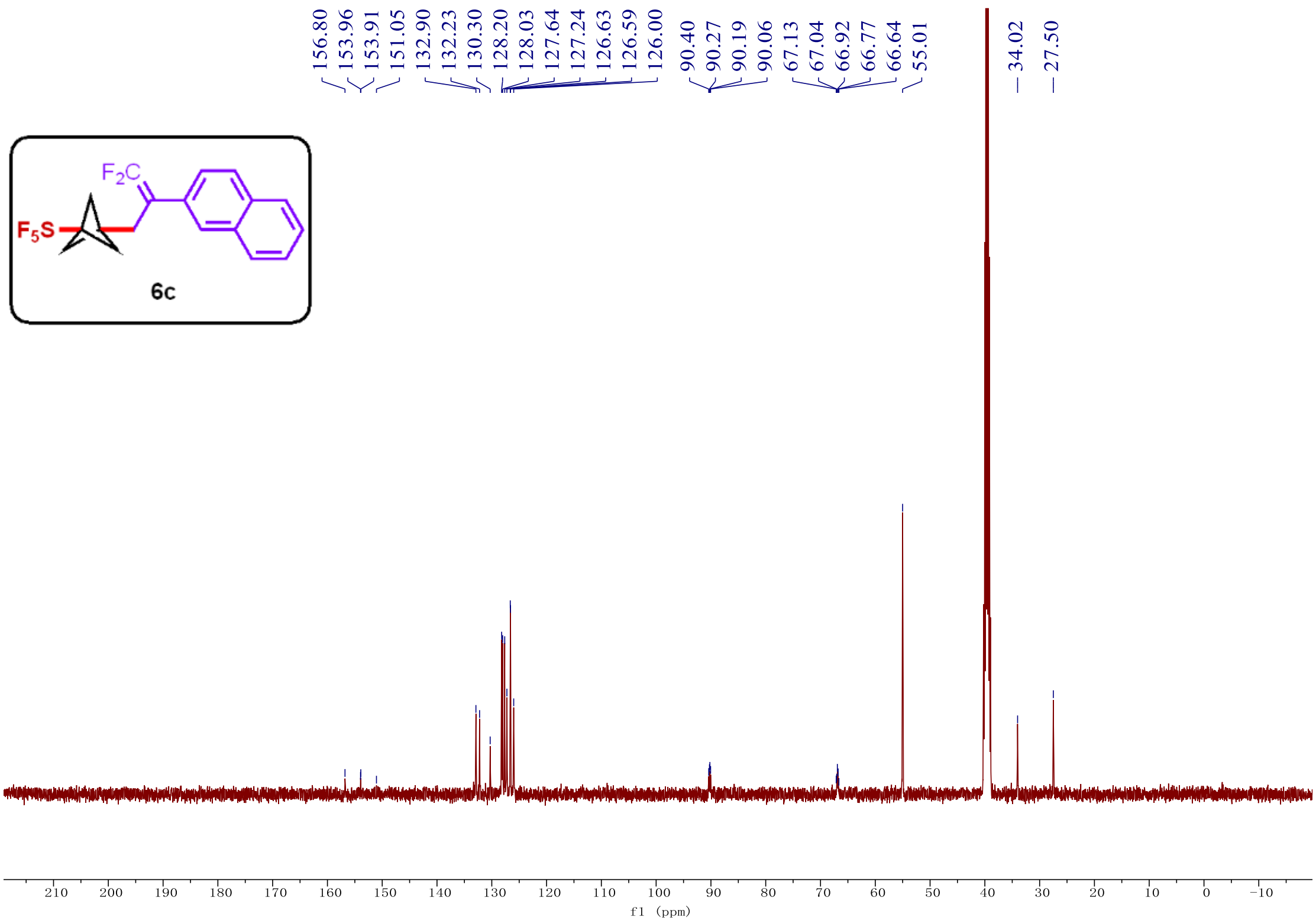

Supplementary Figure 182. ¹³C NMR Spectrum of Compound 6c (101 MHz, DMSO, 25 ˚C)

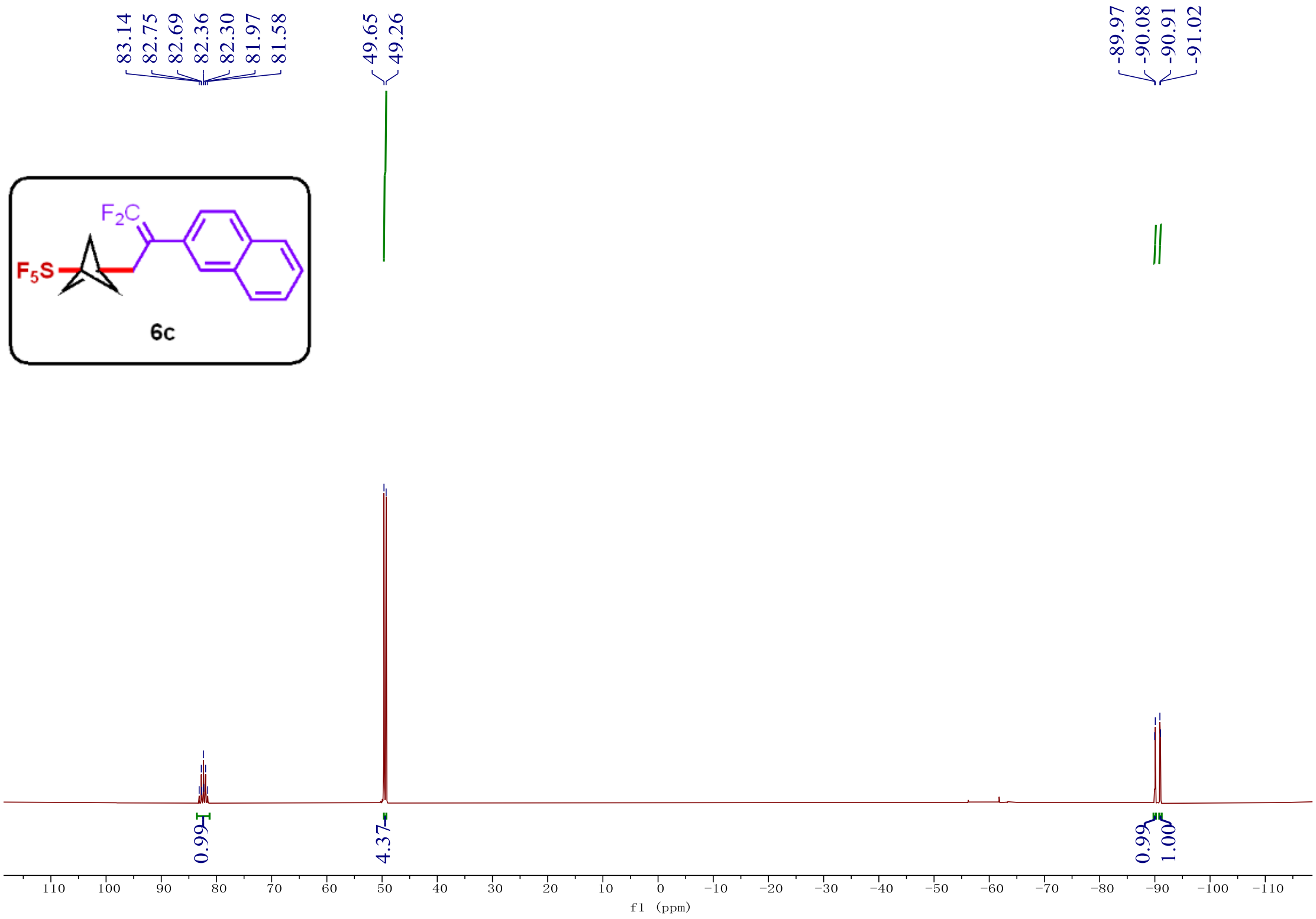

Supplementary Figure 183. ^{19}F NMR Spectrum of Compound 6c (376 MHz, DMSO, 25 °C)

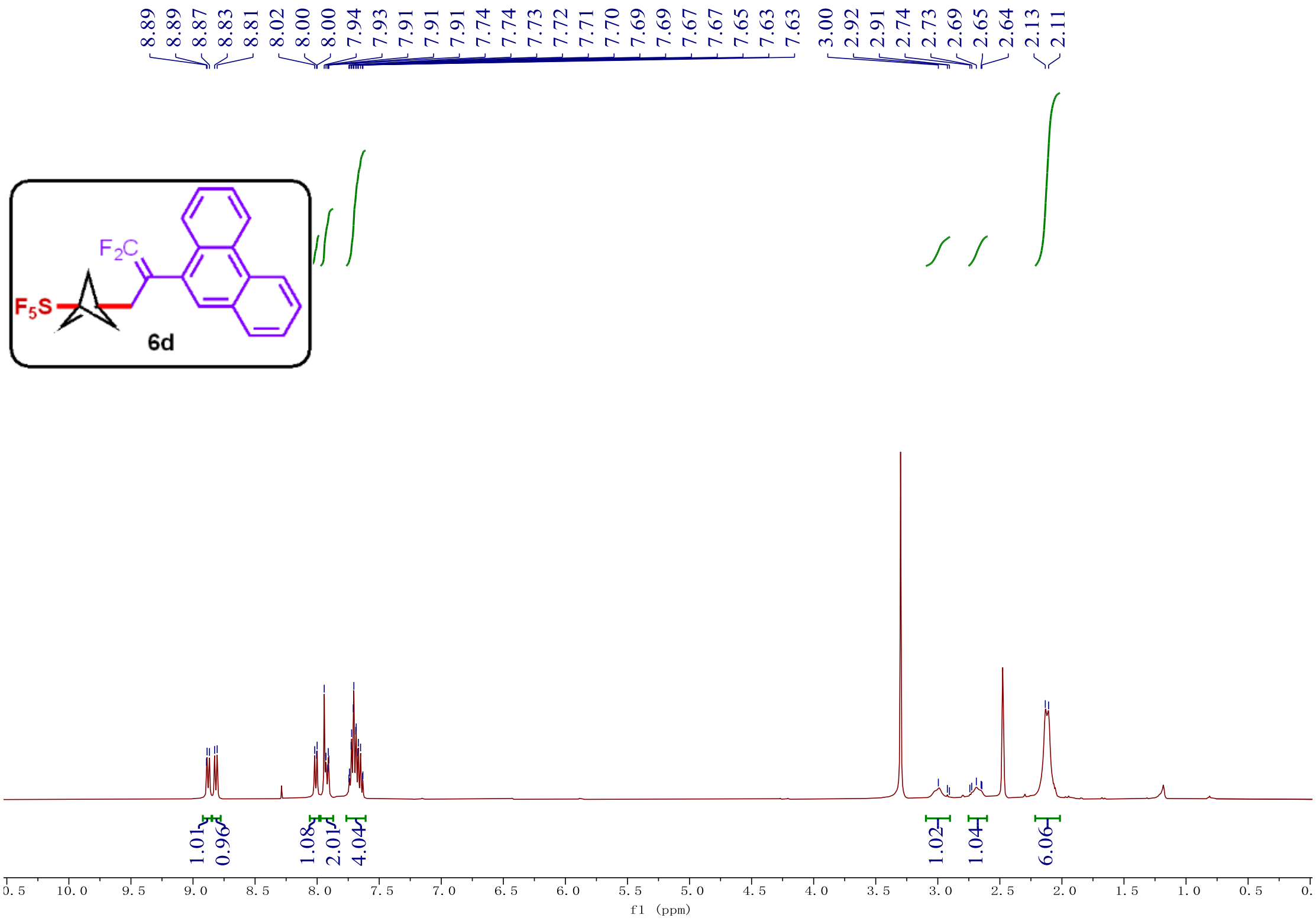

Supplementary Figure 184. ^{1}H NMR Spectrum of Compound 6d (400 MHz, DMSO, 25 °C)

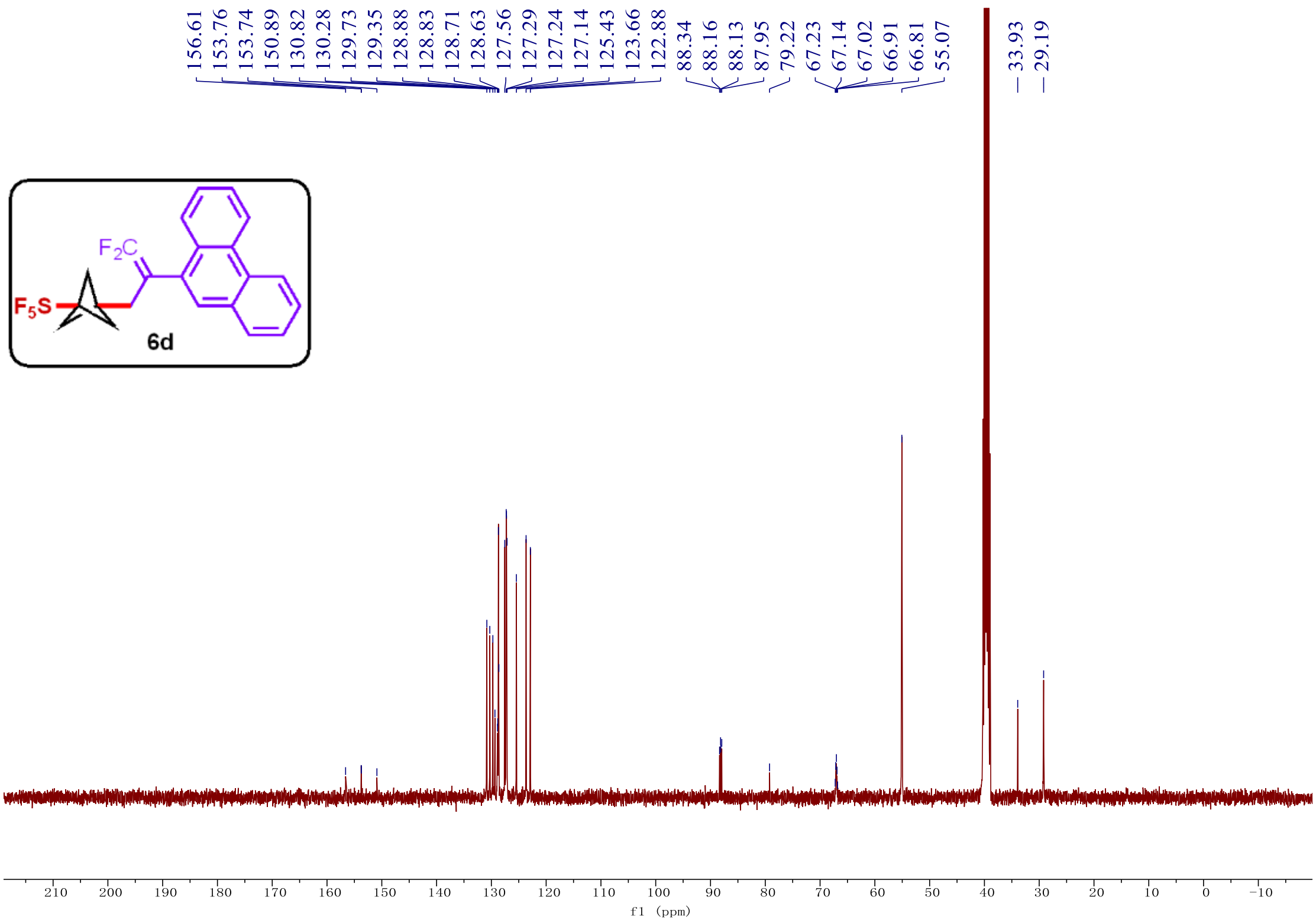

Supplementary Figure 185. ^{13}C NMR Spectrum of Compound 6d (101 MHz, DMSO, 25 °C)

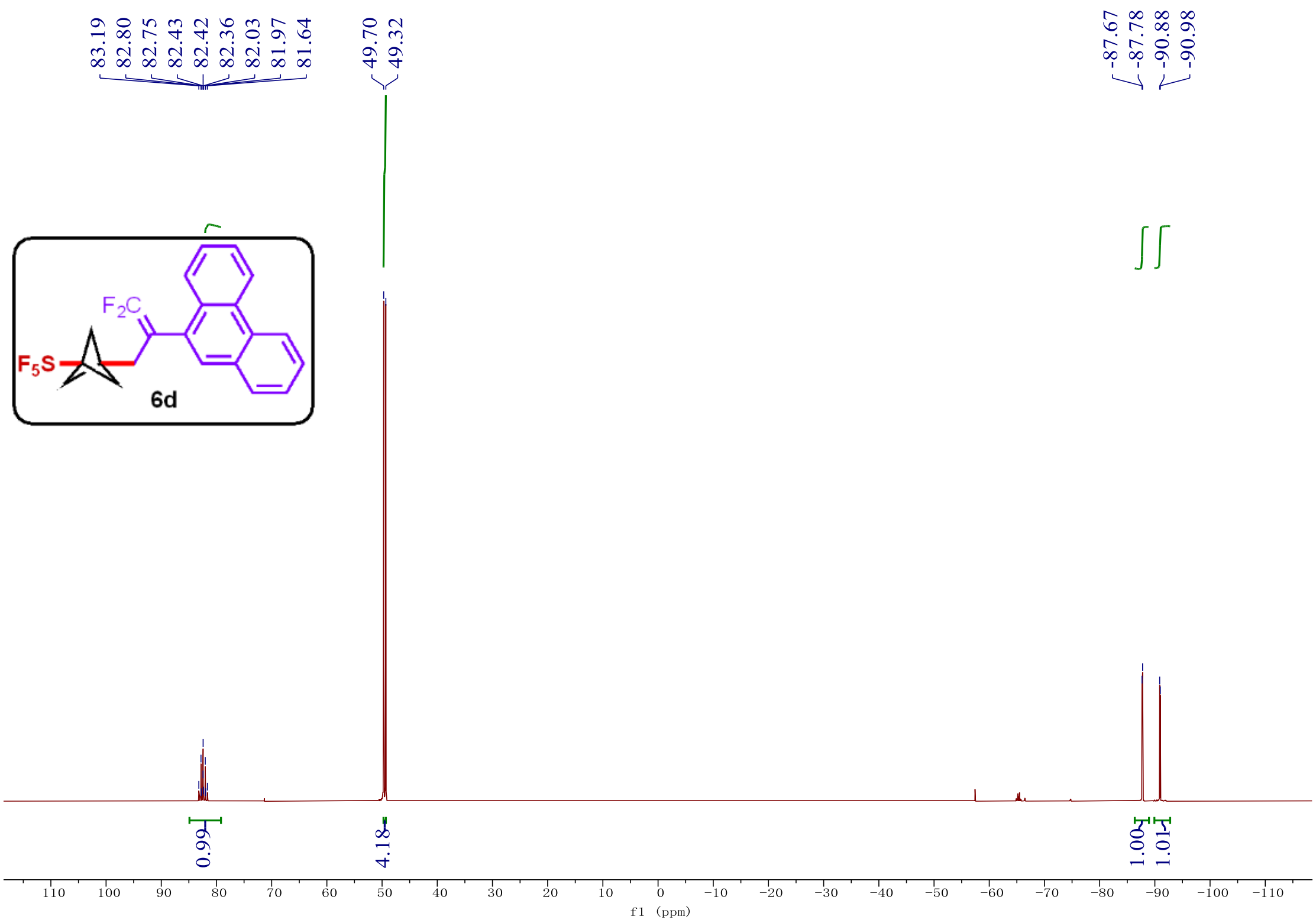

Supplementary Figure 186. ^{19}F NMR Spectrum of Compound 6d (376 MHz, DMSO, 25 °C)

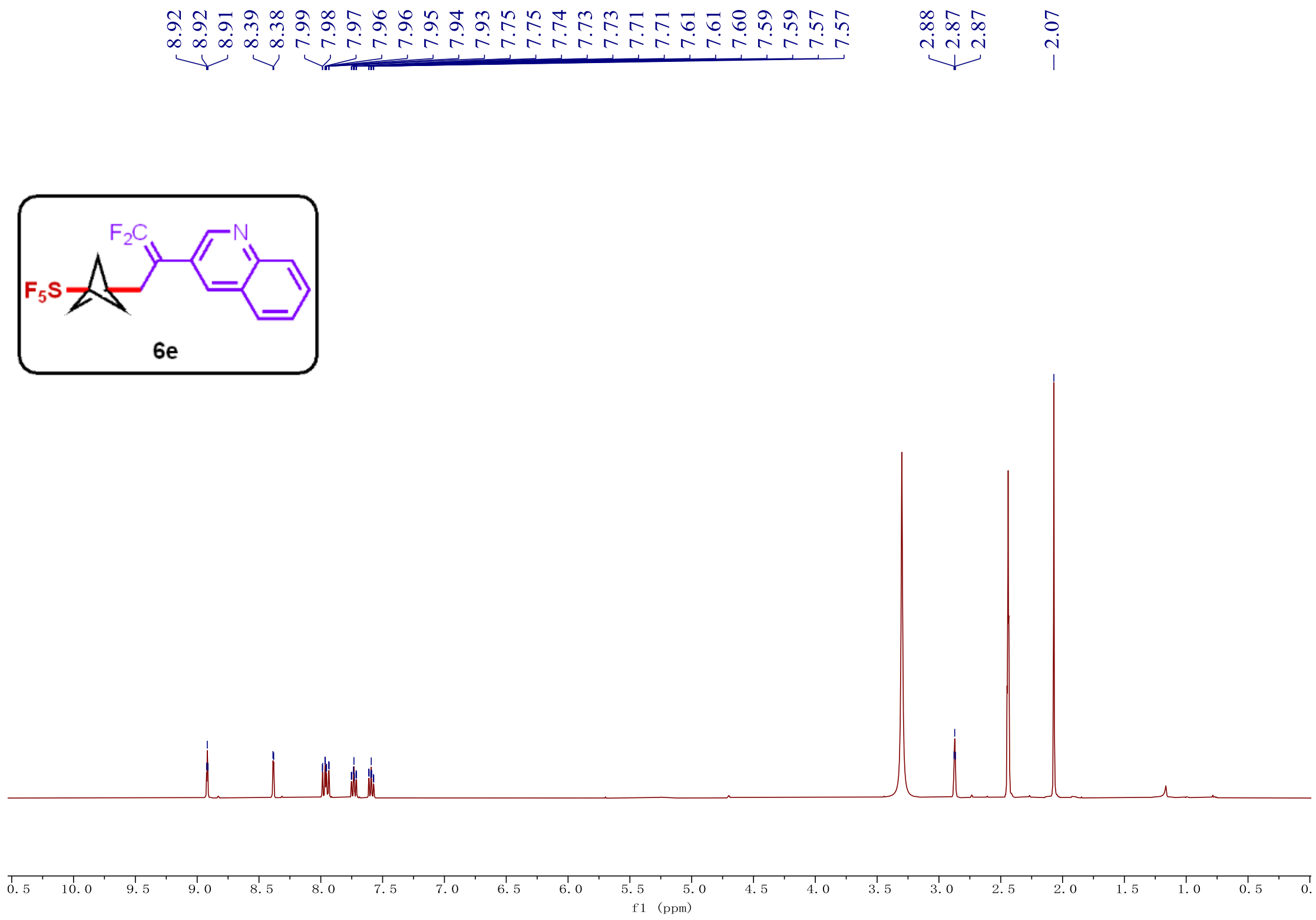

Supplementary Figure 187. ^{1}H **NMR Spectrum of Compound 6e (400 MHz, DMSO, 25 °C)**

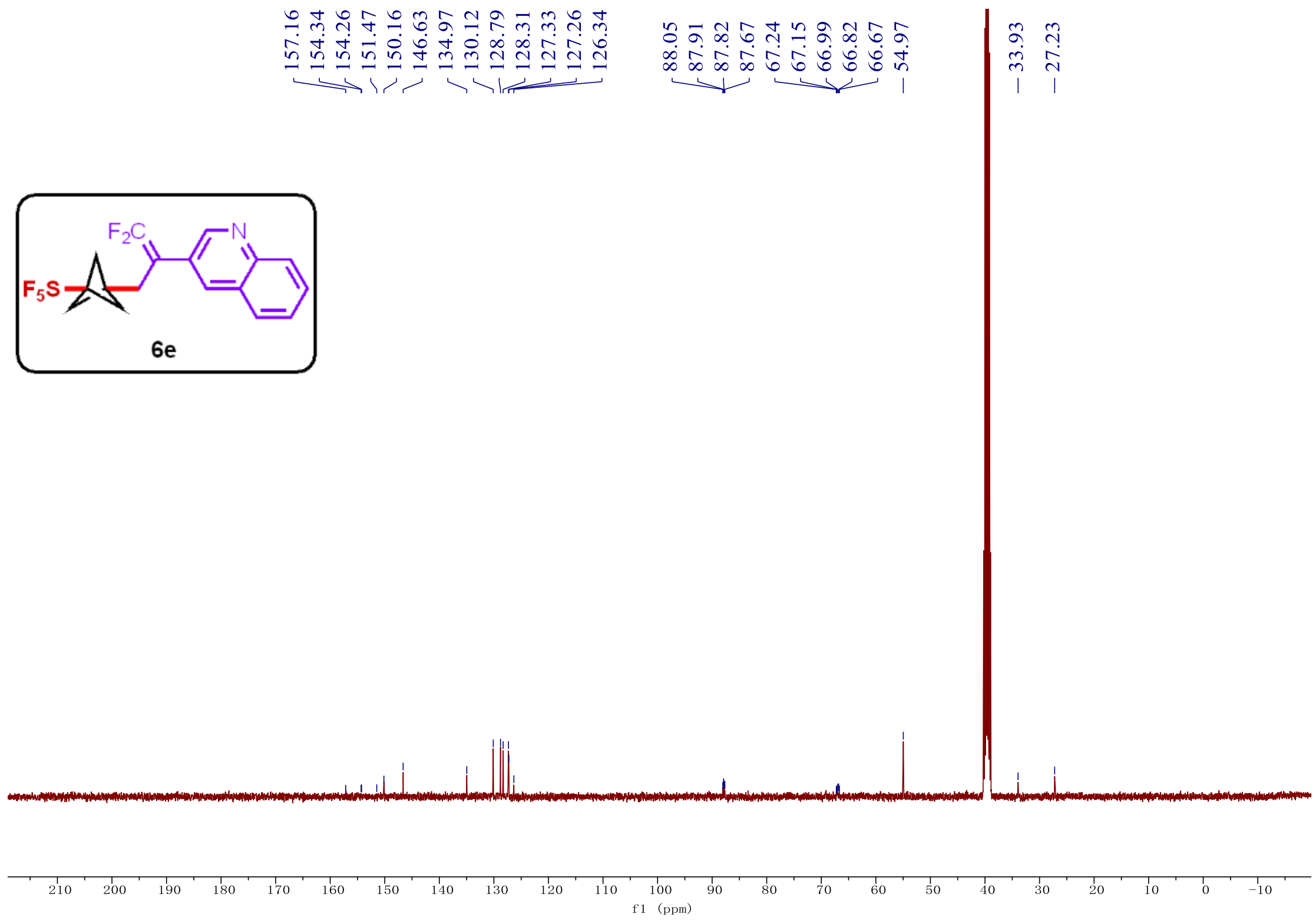

Supplementary Figure 188. ^{13}C **NMR Spectrum of Compound 6e (101 MHz, DMSO, 25 °C)**

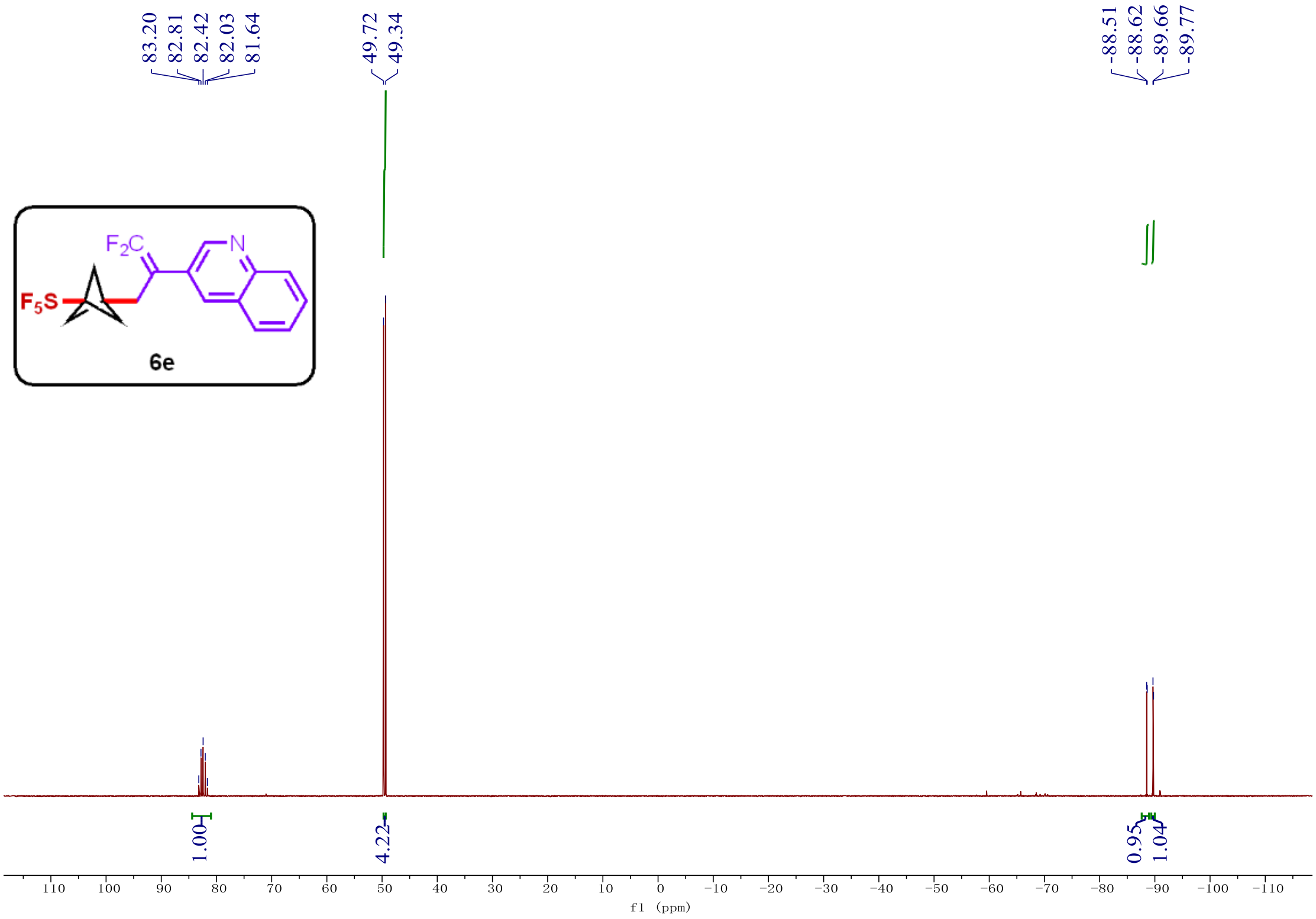

Supplementary Figure 189. ^{19}F NMR Spectrum of Compound 6e (376 MHz, DMSO, 25 °C)

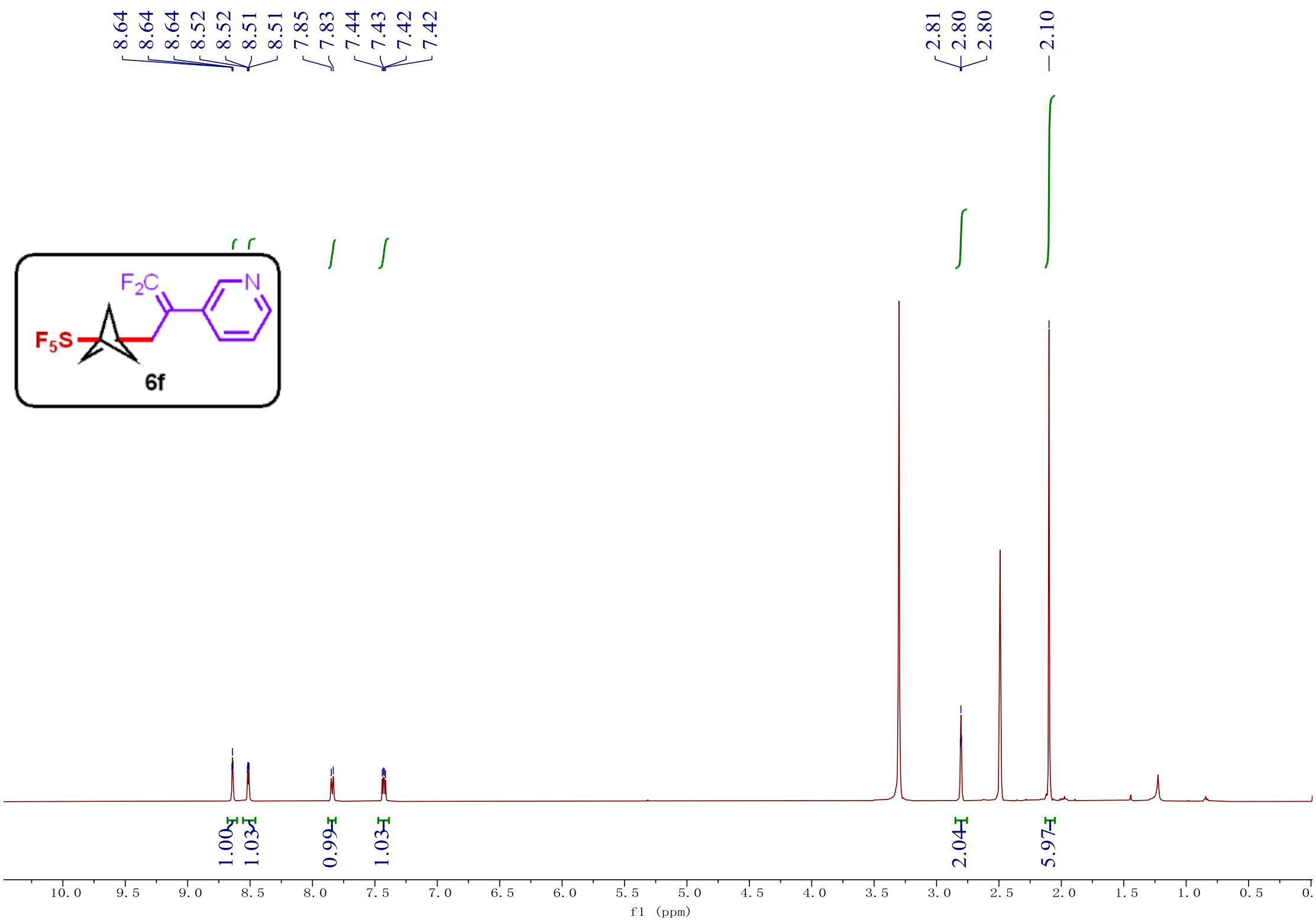

Supplementary Figure 190. ^{1}H NMR Spectrum of Compound 6f (500 MHz, DMSO, 25 °C)

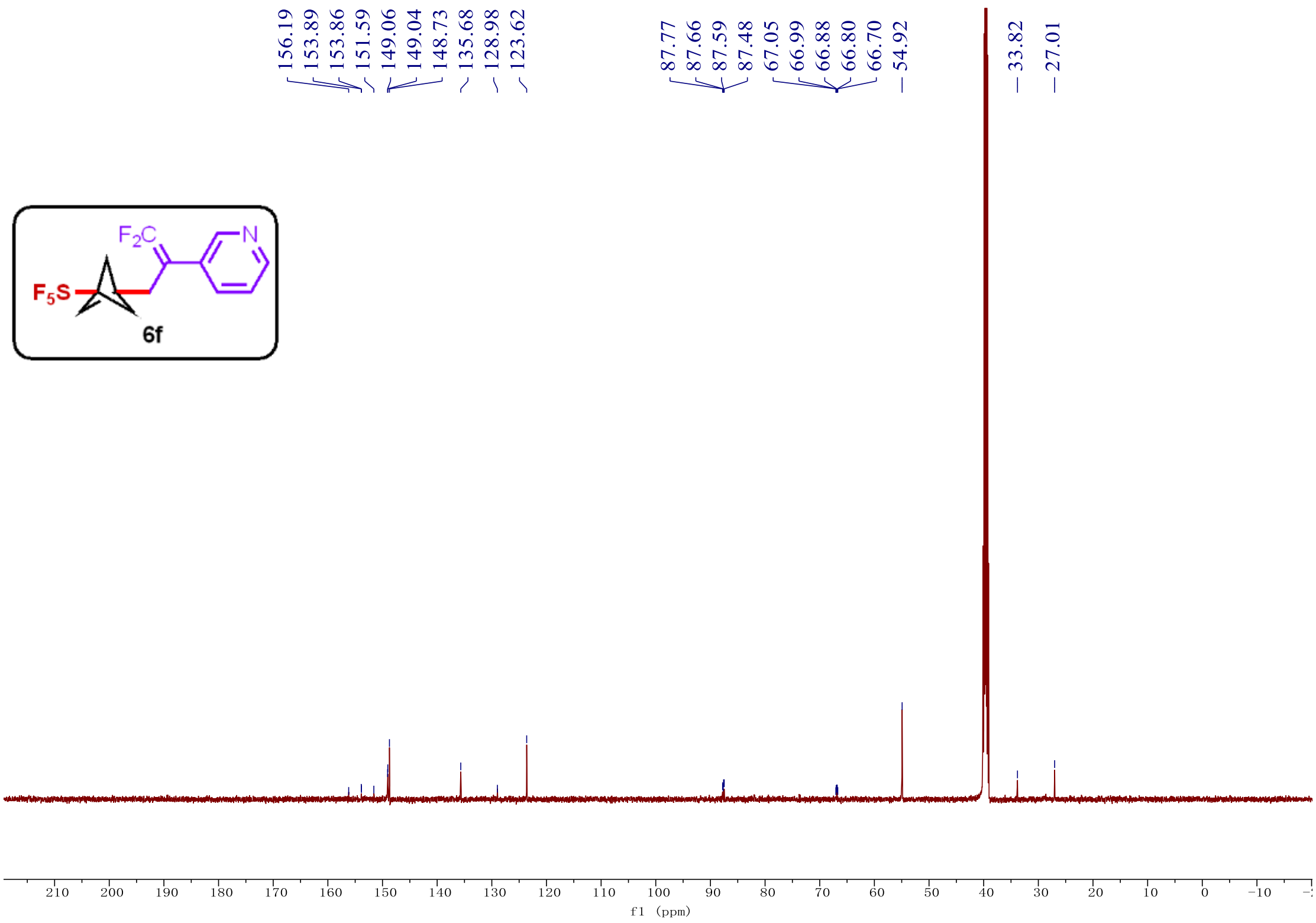

Supplementary Figure 191. ^{13}C NMR Spectrum of Compound 6f (126 MHz, DMSO, 25 °C)

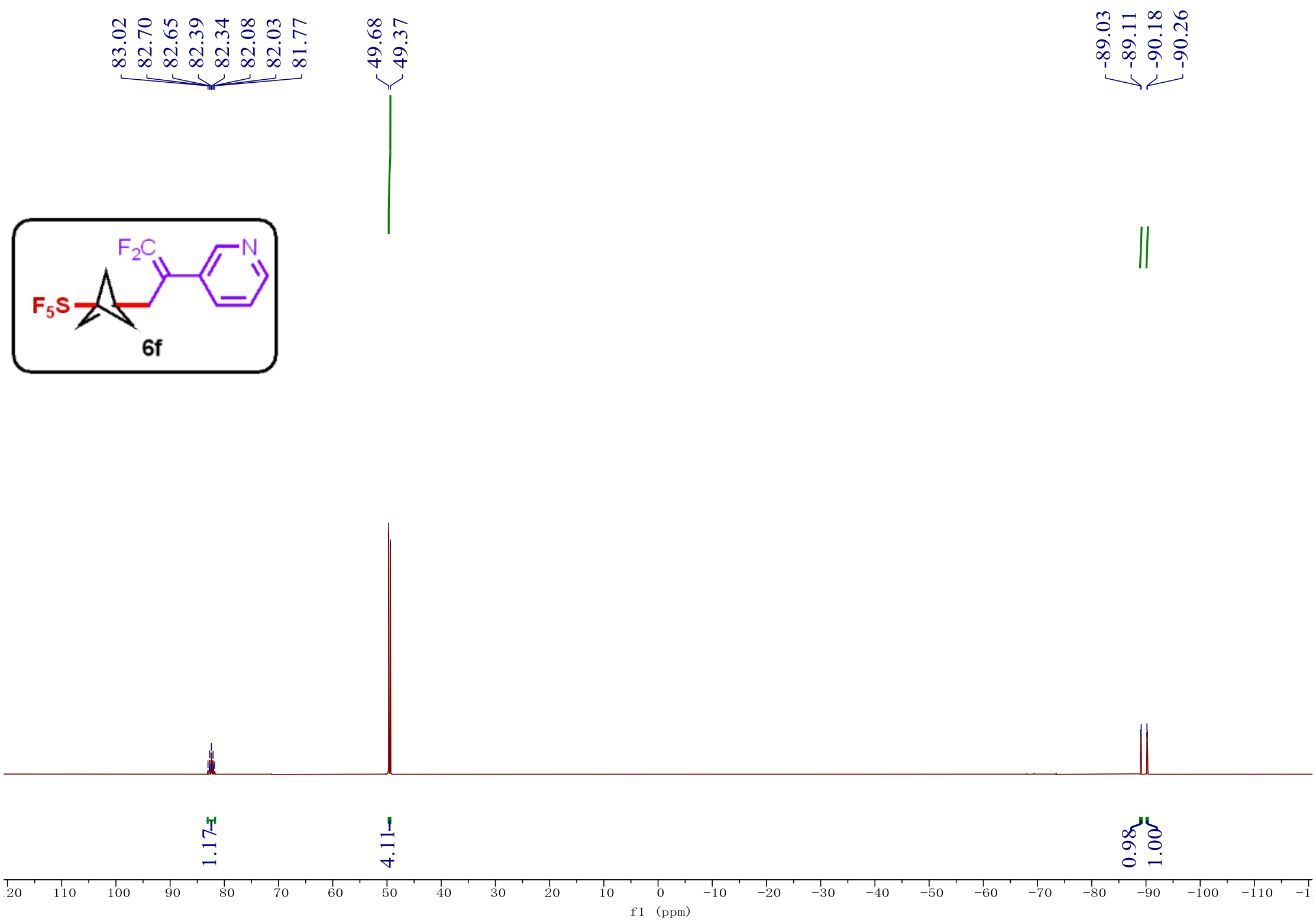

Supplementary Figure 192. ^{19}F NMR Spectrum of Compound 6f (471 MHz, DMSO, 25 °C)

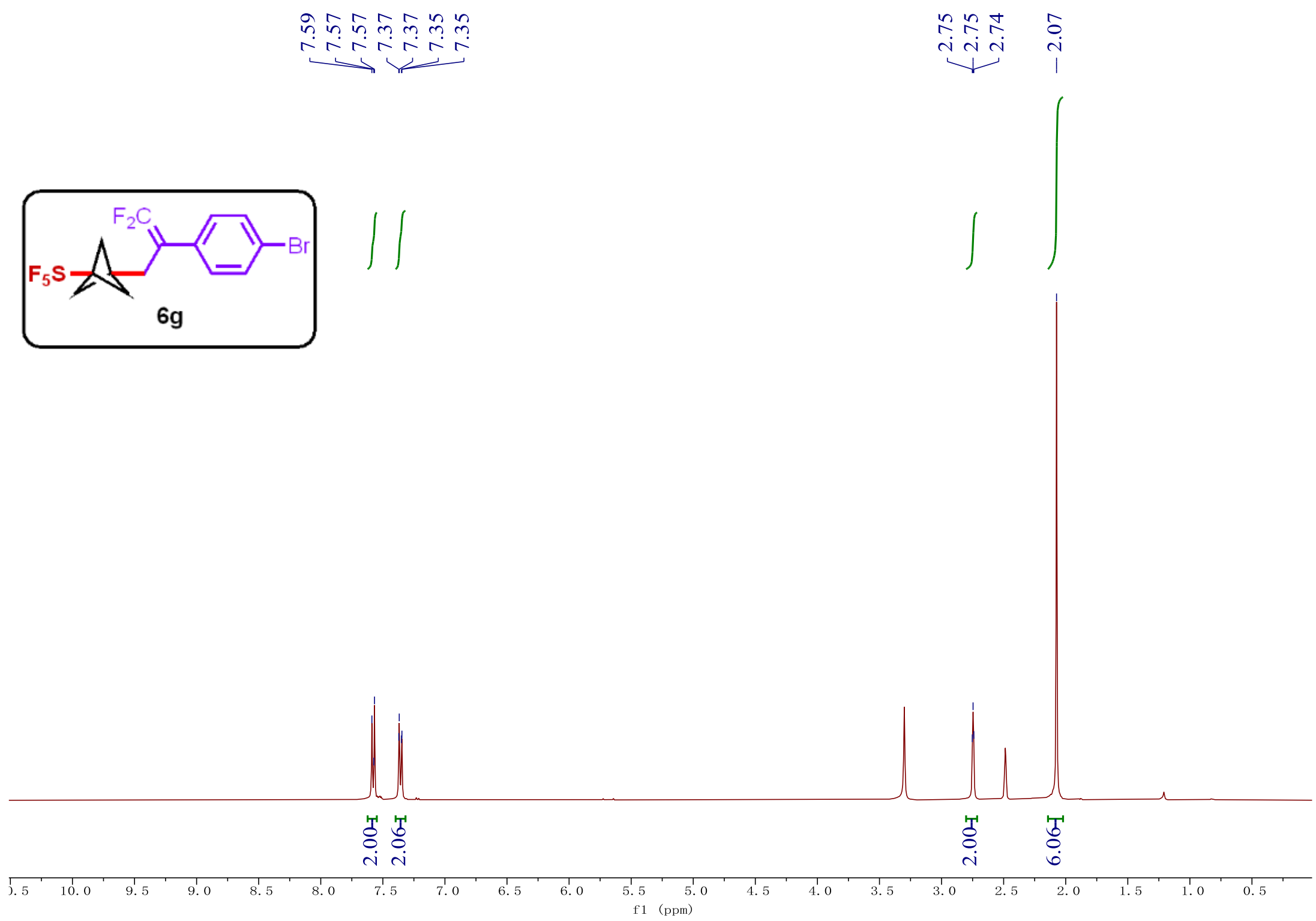

Supplementary Figure 193. ^{1}H NMR Spectrum of Compound 6g (400 MHz, DMSO, 25 °C)

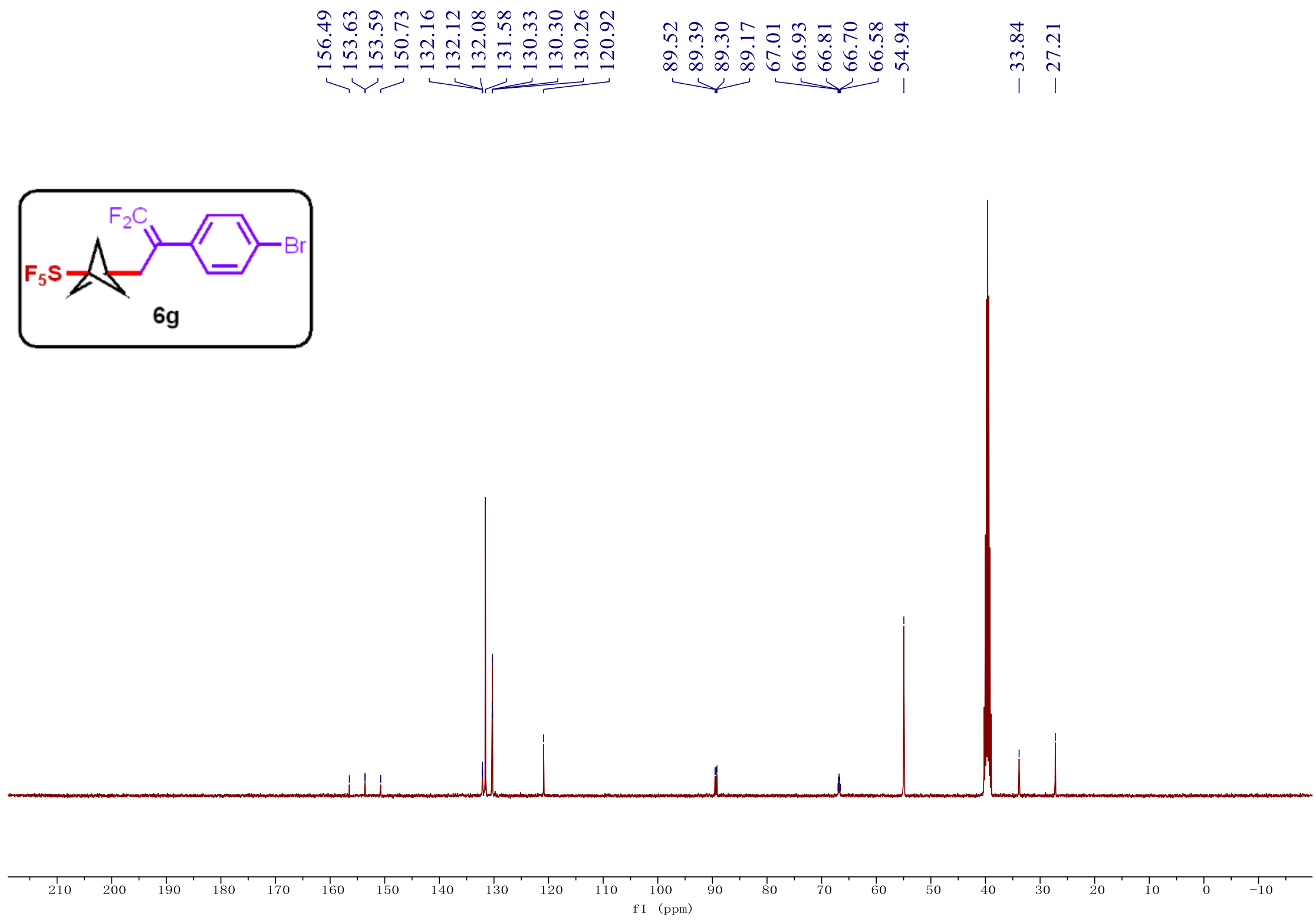

Supplementary Figure 194. ^{13}C NMR Spectrum of Compound 6g (101 MHz, DMSO, 25 °C)

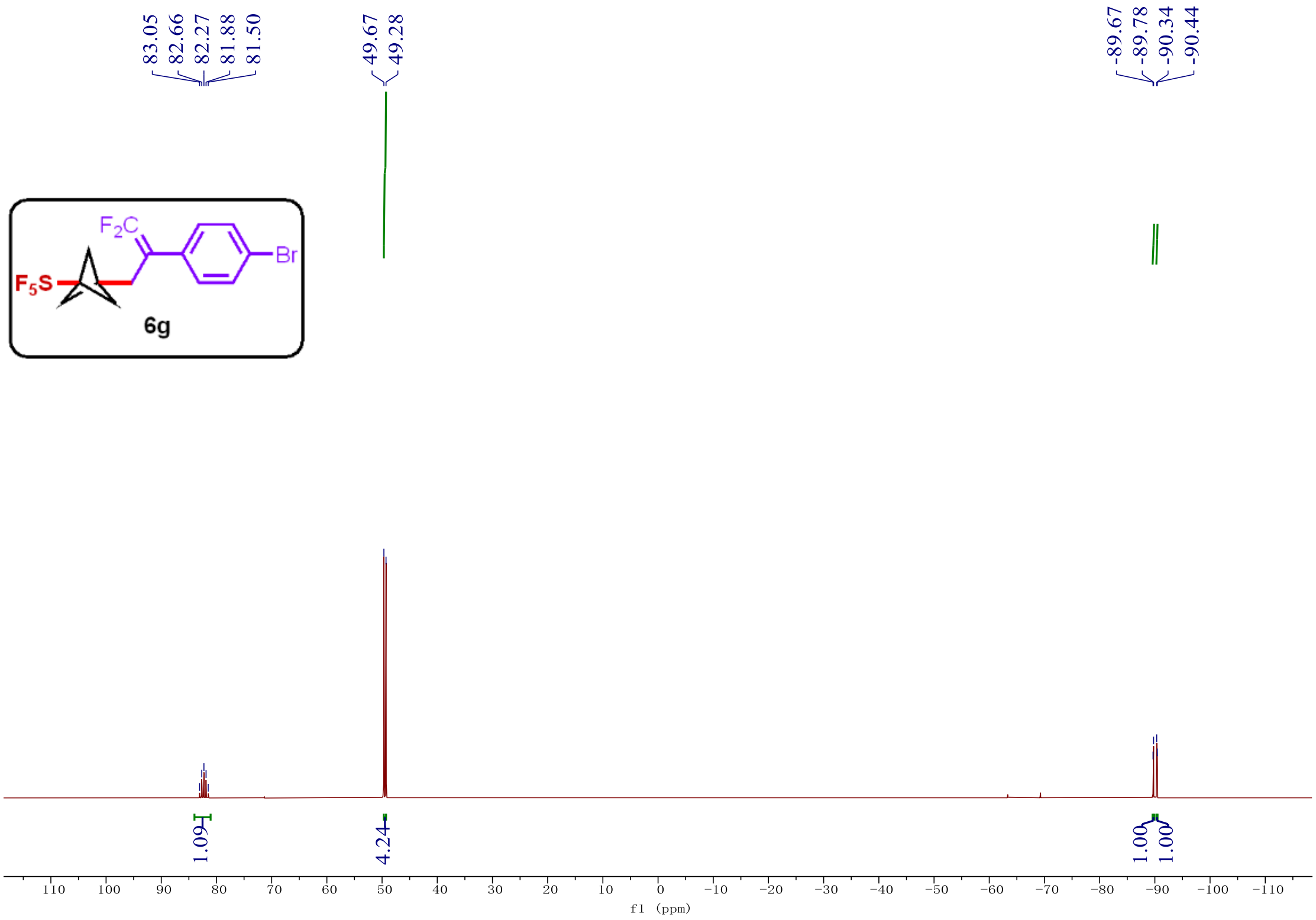

Supplementary Figure 195. ^{19}F NMR Spectrum of Compound 6g (376 MHz, DMSO, 25 °C)

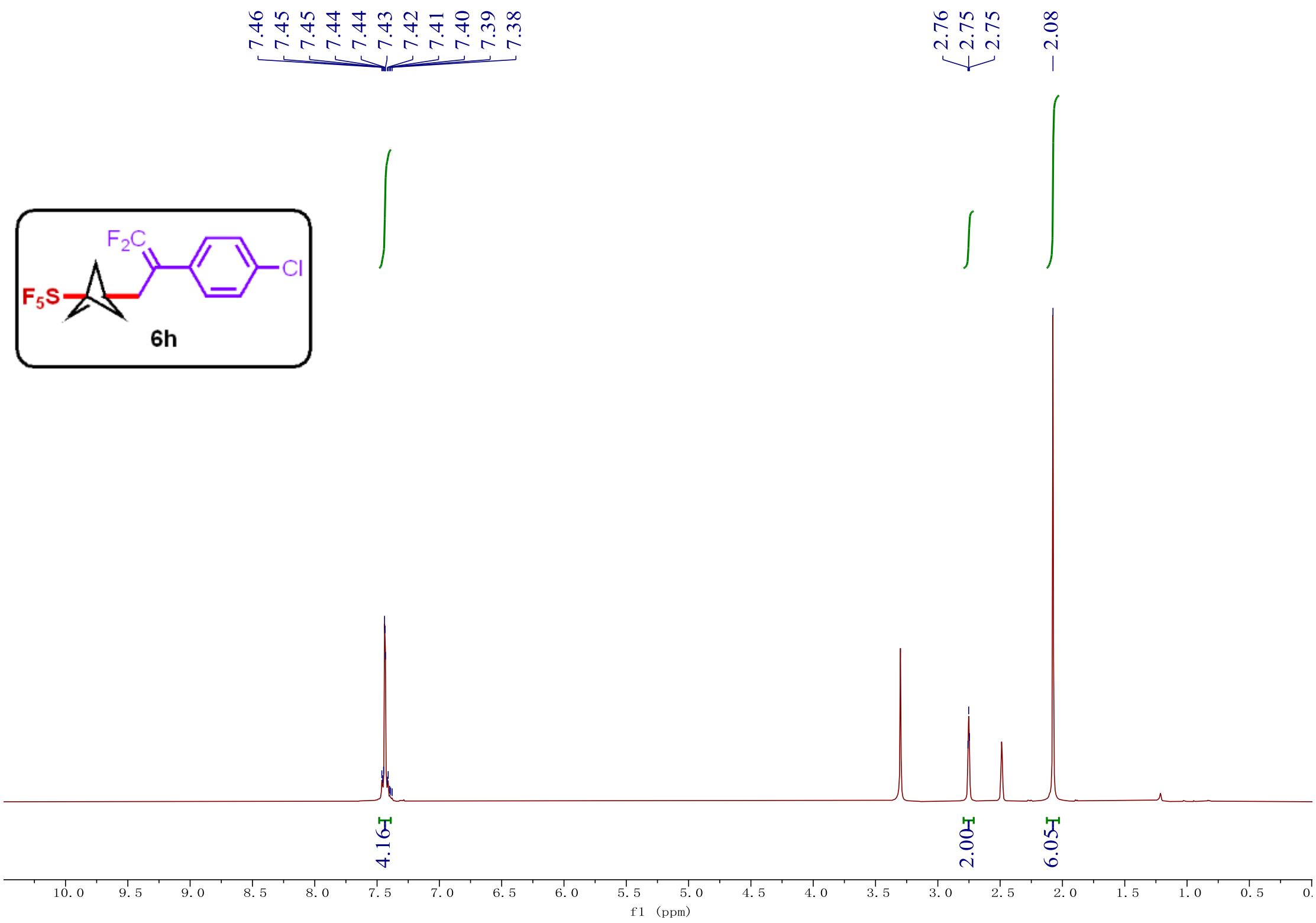

Supplementary Figure 196. ^{1}H NMR Spectrum of Compound 6h (400 MHz, DMSO, 25 °C)

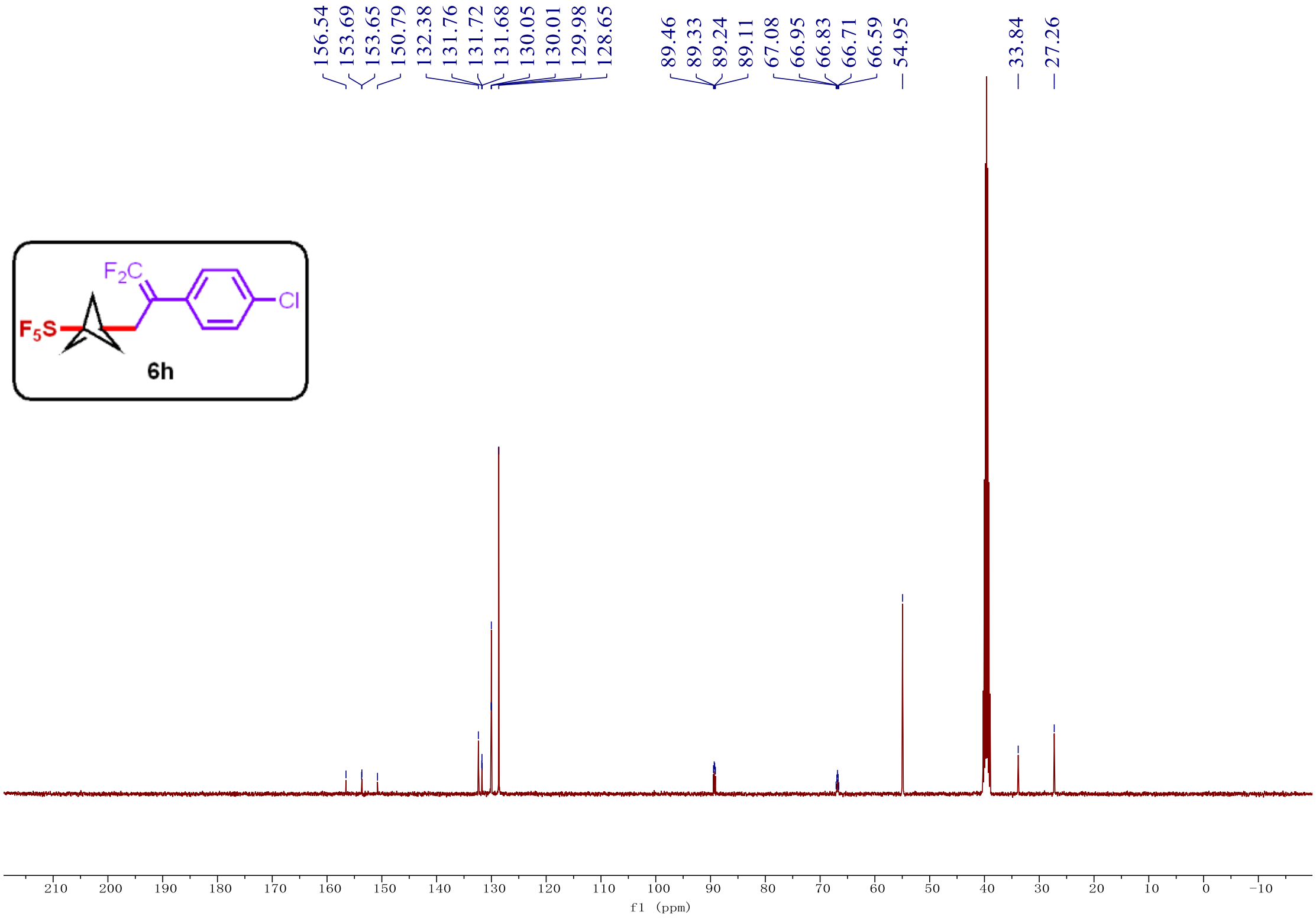

Supplementary Figure 197. ^{13}C NMR Spectrum of Compound 6h (101 MHz, DMSO, 25 °C)

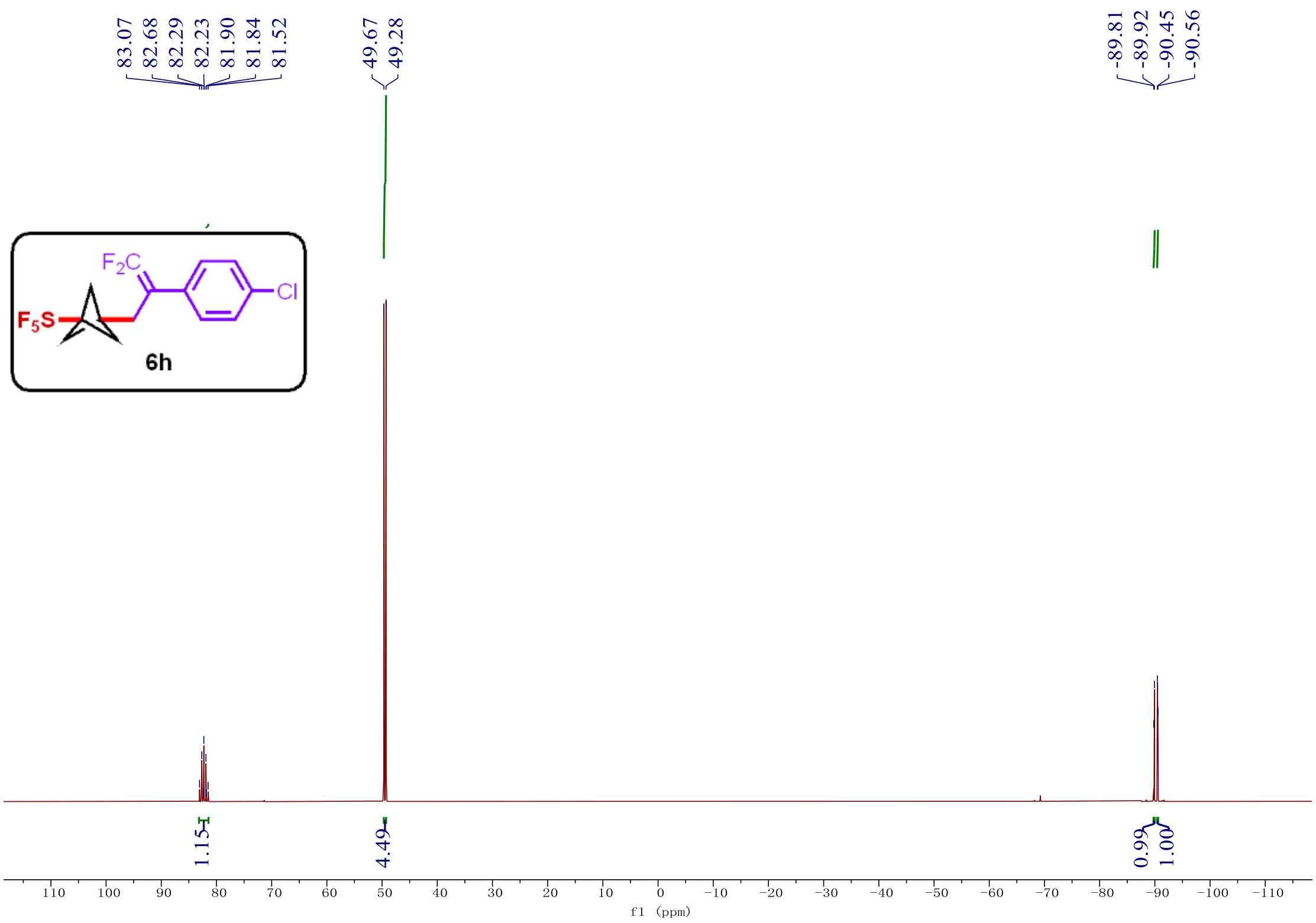

Supplementary Figure 198. ^{19}F NMR Spectrum of Compound 6h (376 MHz, DMSO, 25 °C)

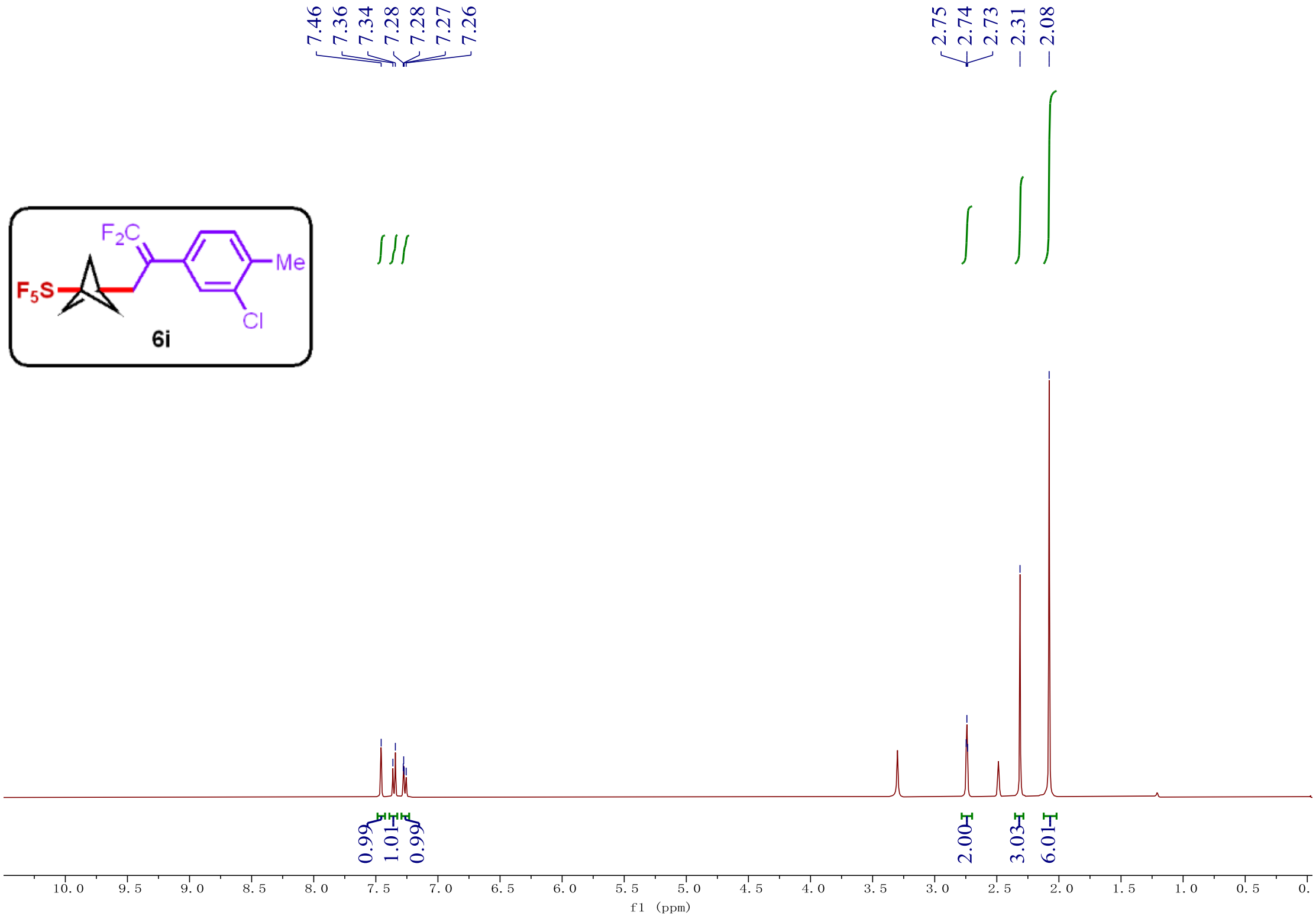

Supplementary Figure 199. ^{1}H NMR Spectrum of Compound 6i (400 MHz, DMSO, 25 °C)

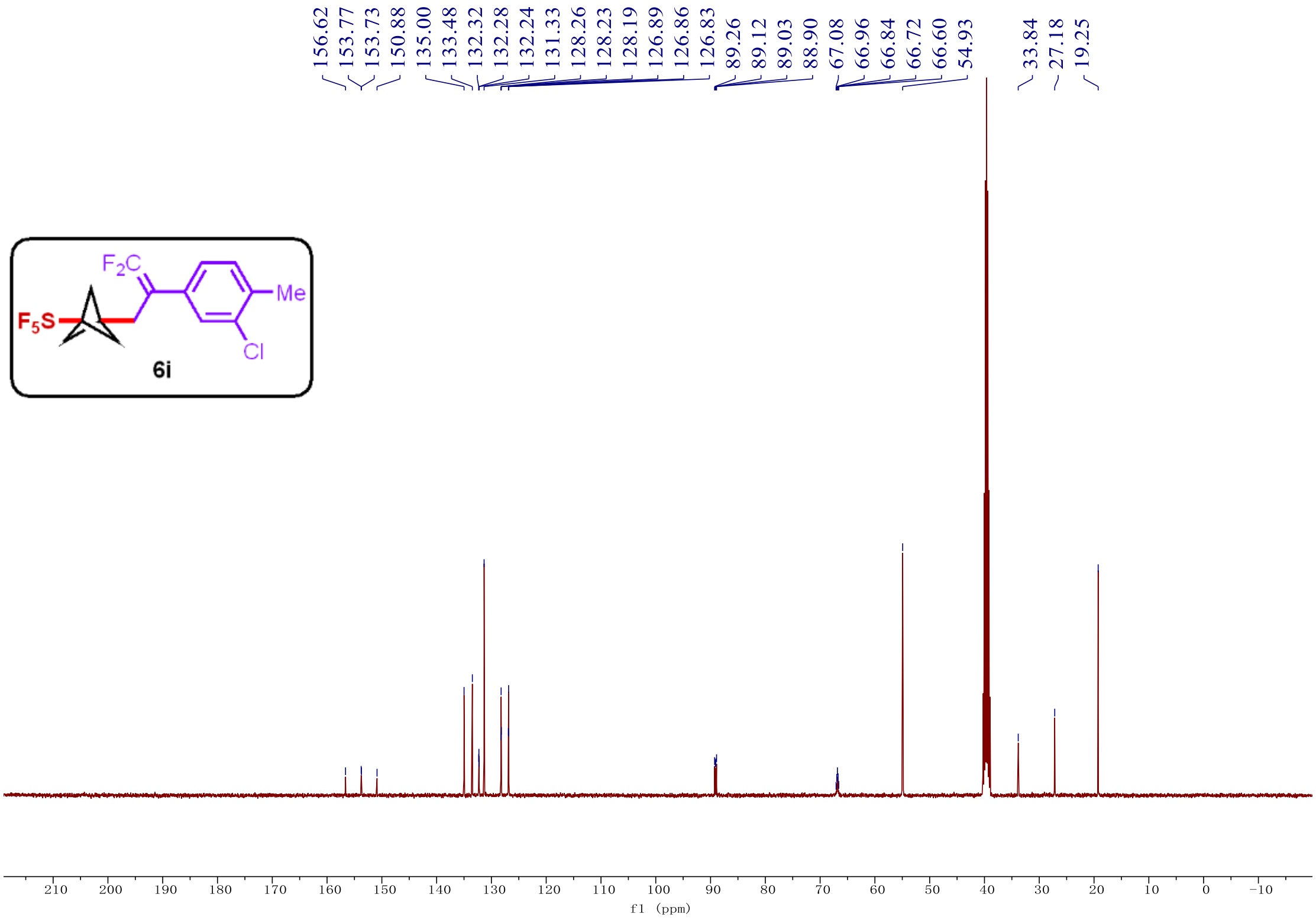

Supplementary Figure 200. ^{13}C NMR Spectrum of Compound 6i (101 MHz, DMSO, 25 °C)

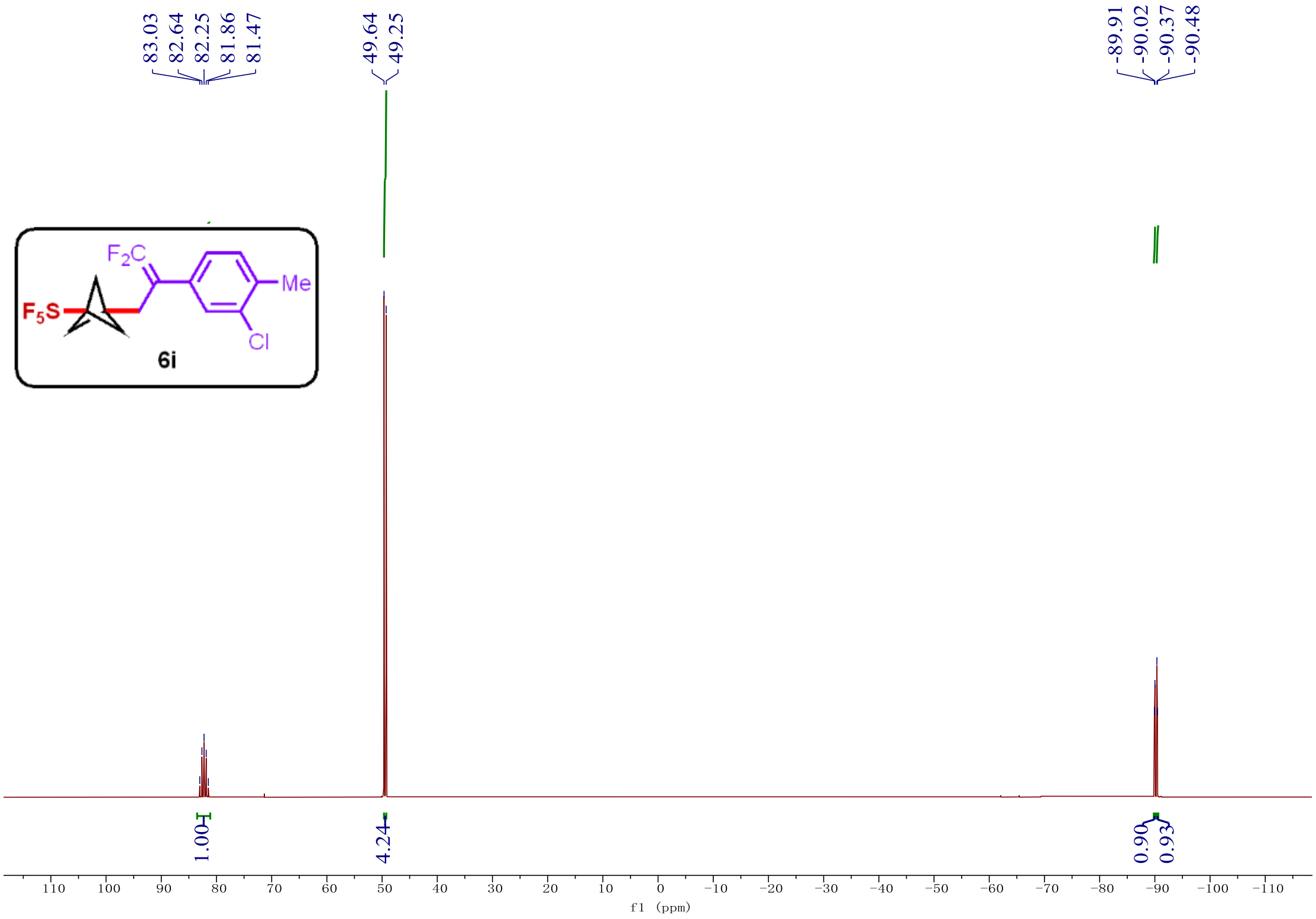

Supplementary Figure 201. ^{19}F NMR Spectrum of Compound 6i (376 MHz, DMSO, 25 ˚C)

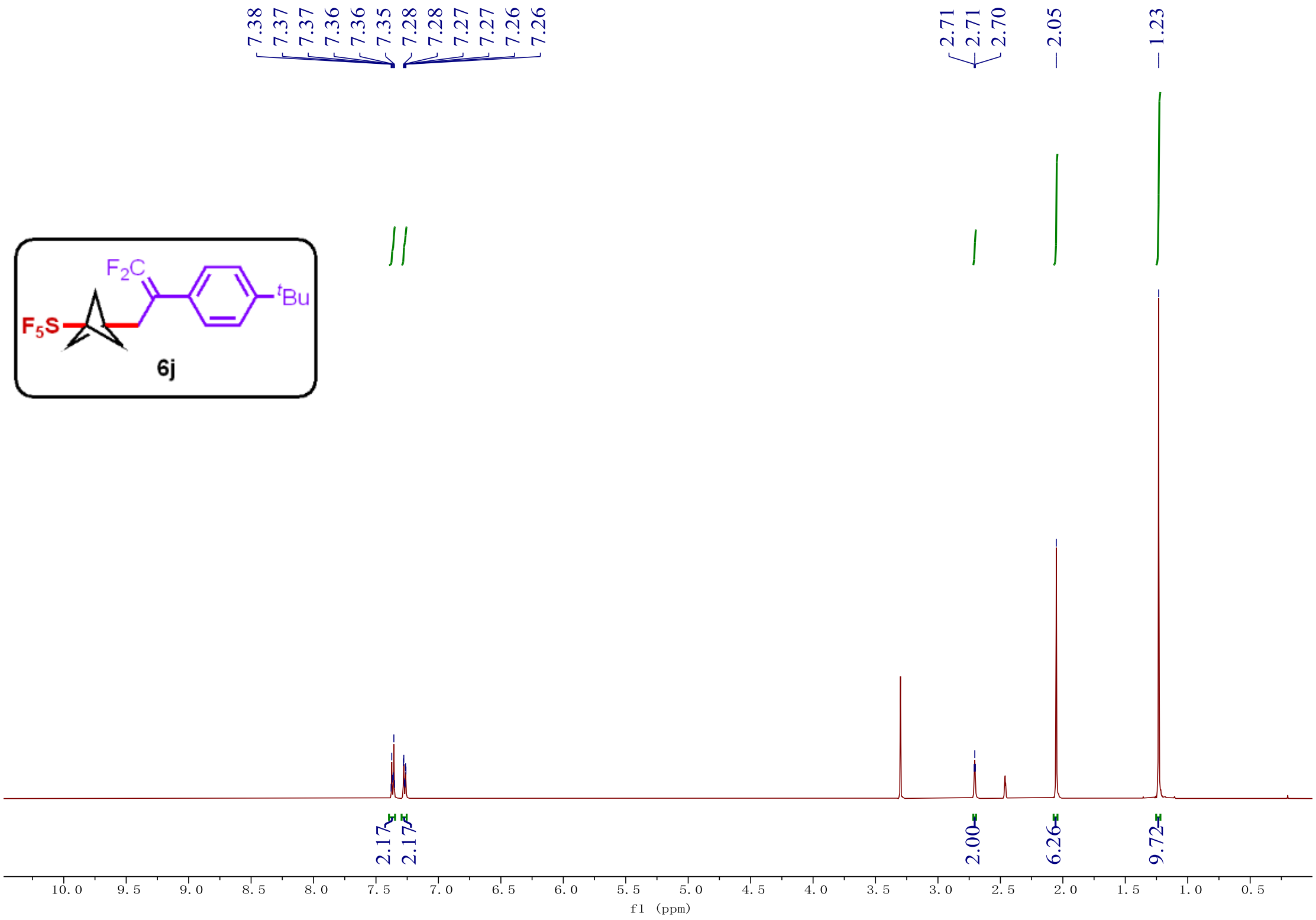

Supplementary Figure 202. ^{1}H NMR Spectrum of Compound 6j (500 MHz, DMSO, 25 ˚C)

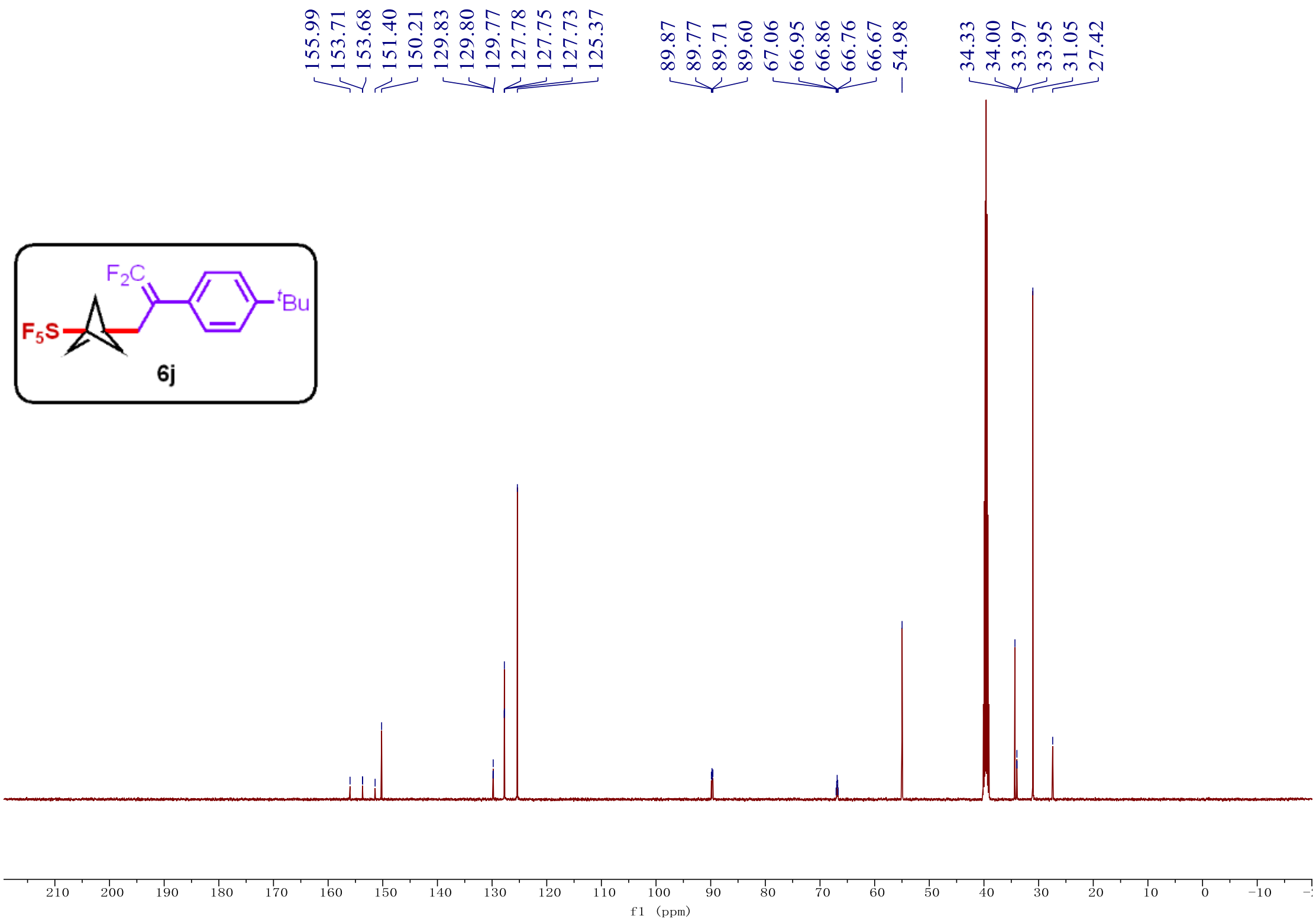

Supplementary Figure 203. ^{13}C NMR Spectrum of Compound 6j (126 MHz, DMSO, 25 °C)

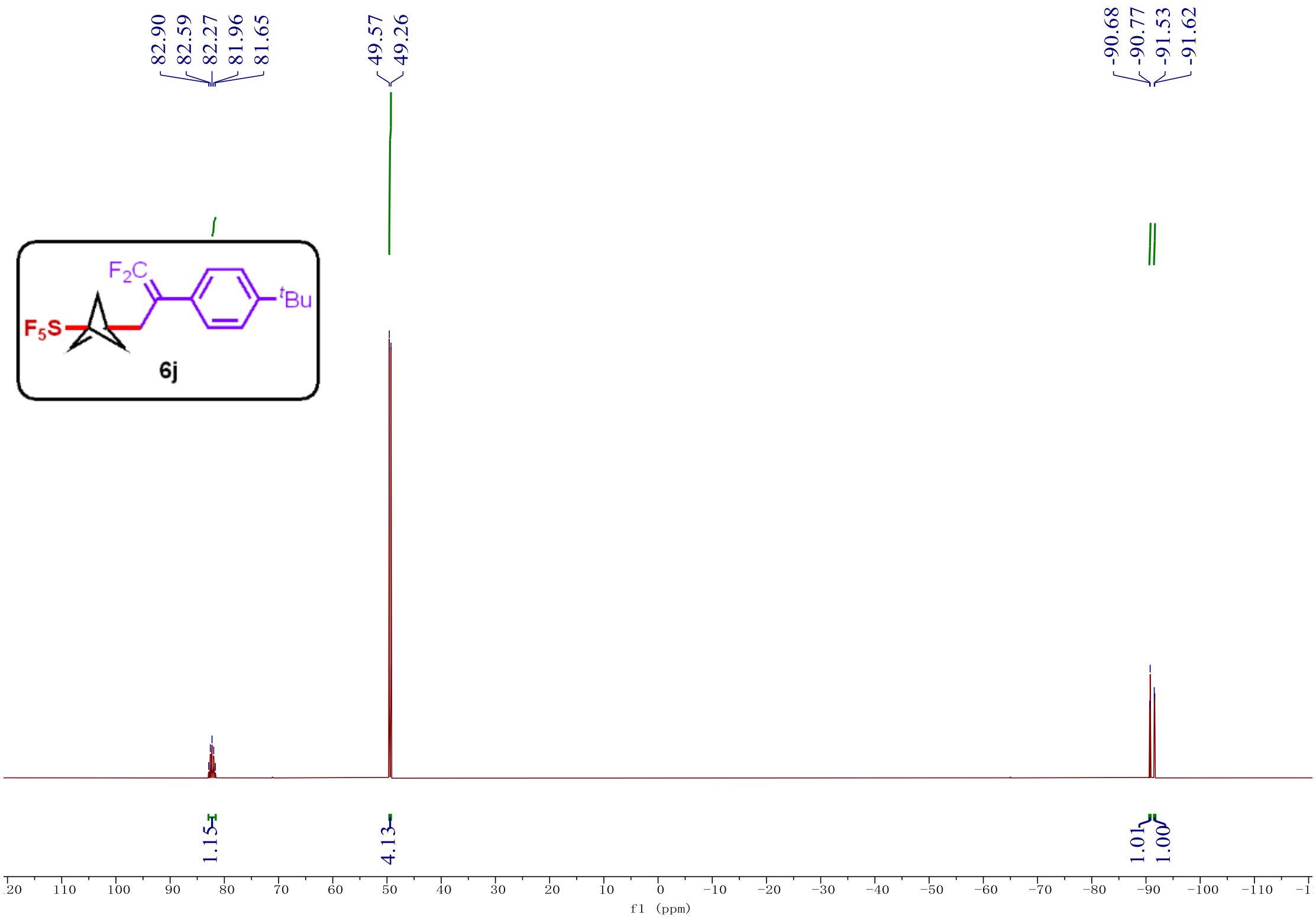

Supplementary Figure 204. ^{19}F NMR Spectrum of Compound 6j (471 MHz, DMSO, 25 °C)

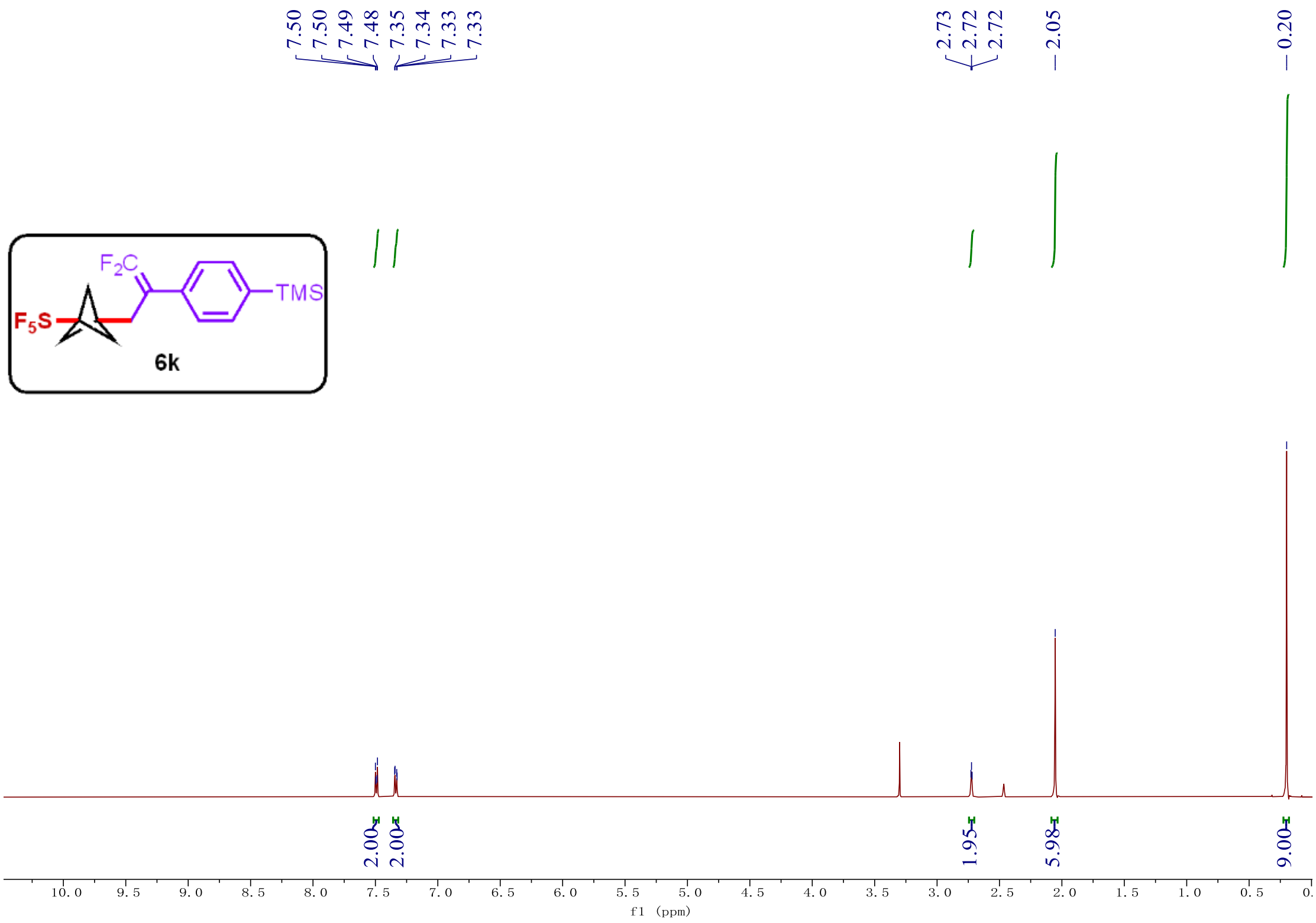

Supplementary Figure 205. ^{1}H NMR Spectrum of Compound 6k (500 MHz, DMSO, 25 ˚C)

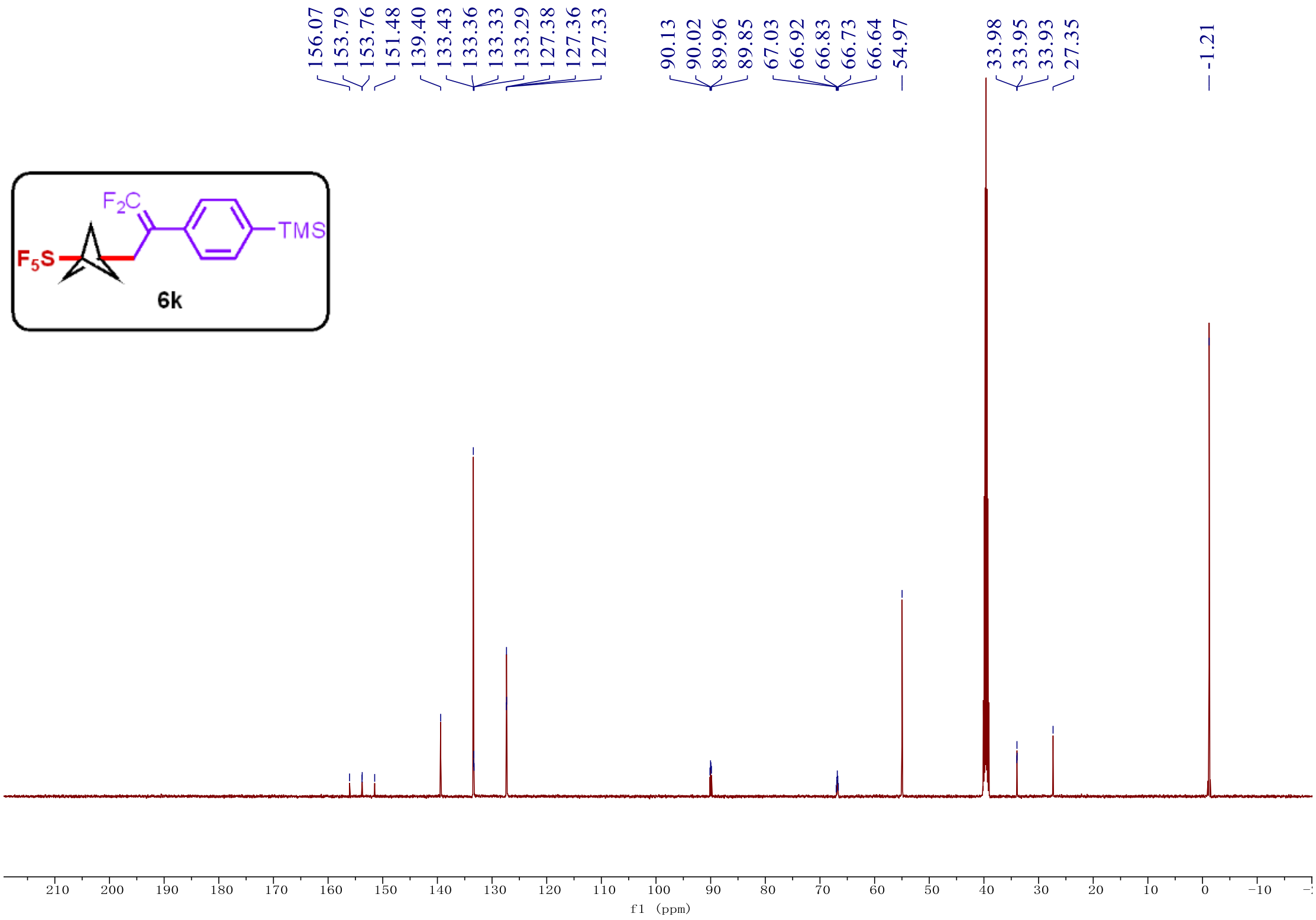

Supplementary Figure 206. ^{13}C NMR Spectrum of Compound 6k (126 MHz, DMSO, 25 ˚C)

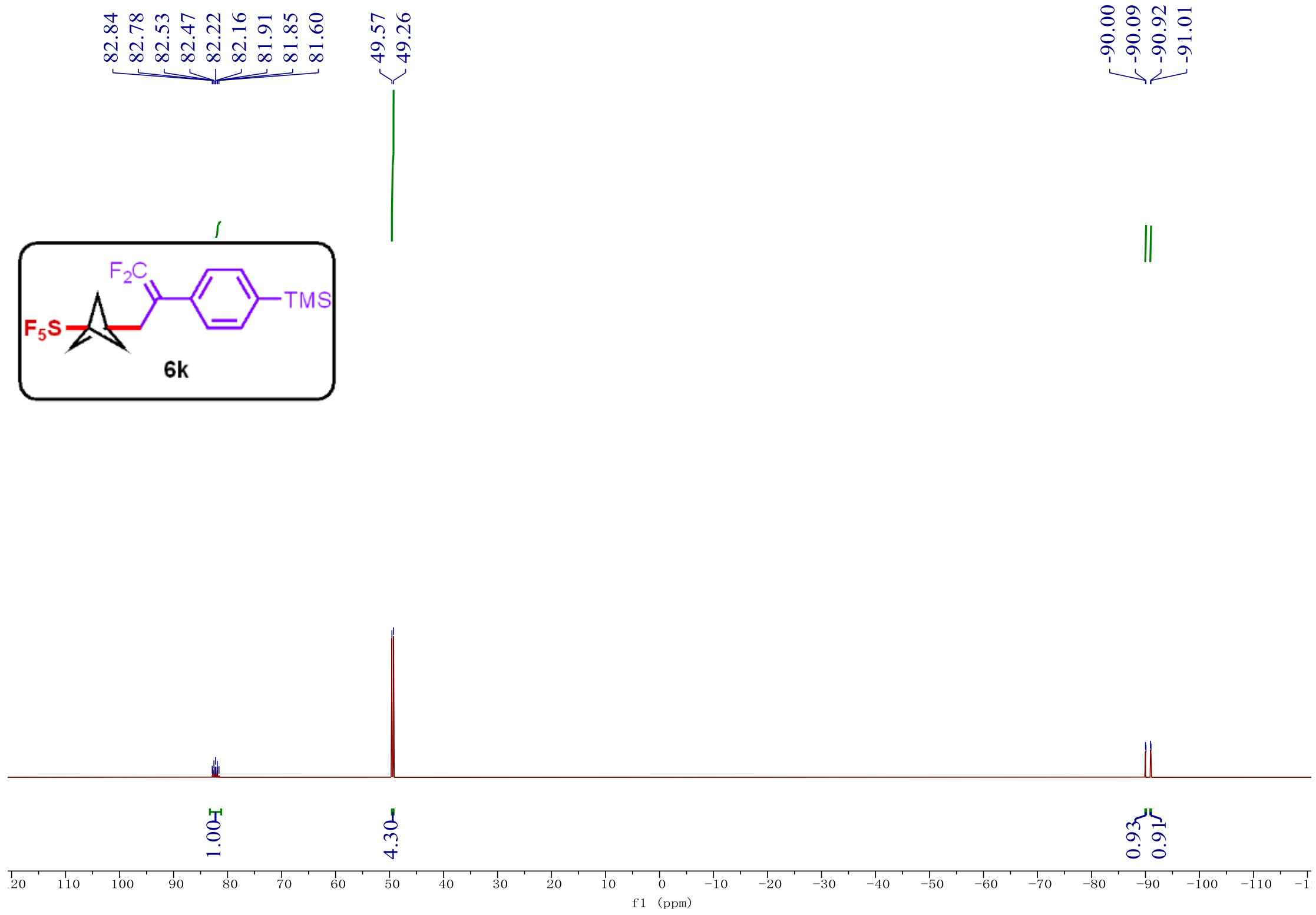

Supplementary Figure 207. ^{19}F **NMR Spectrum of Compound 6k (471 MHz, DMSO, 25 ˚C)**

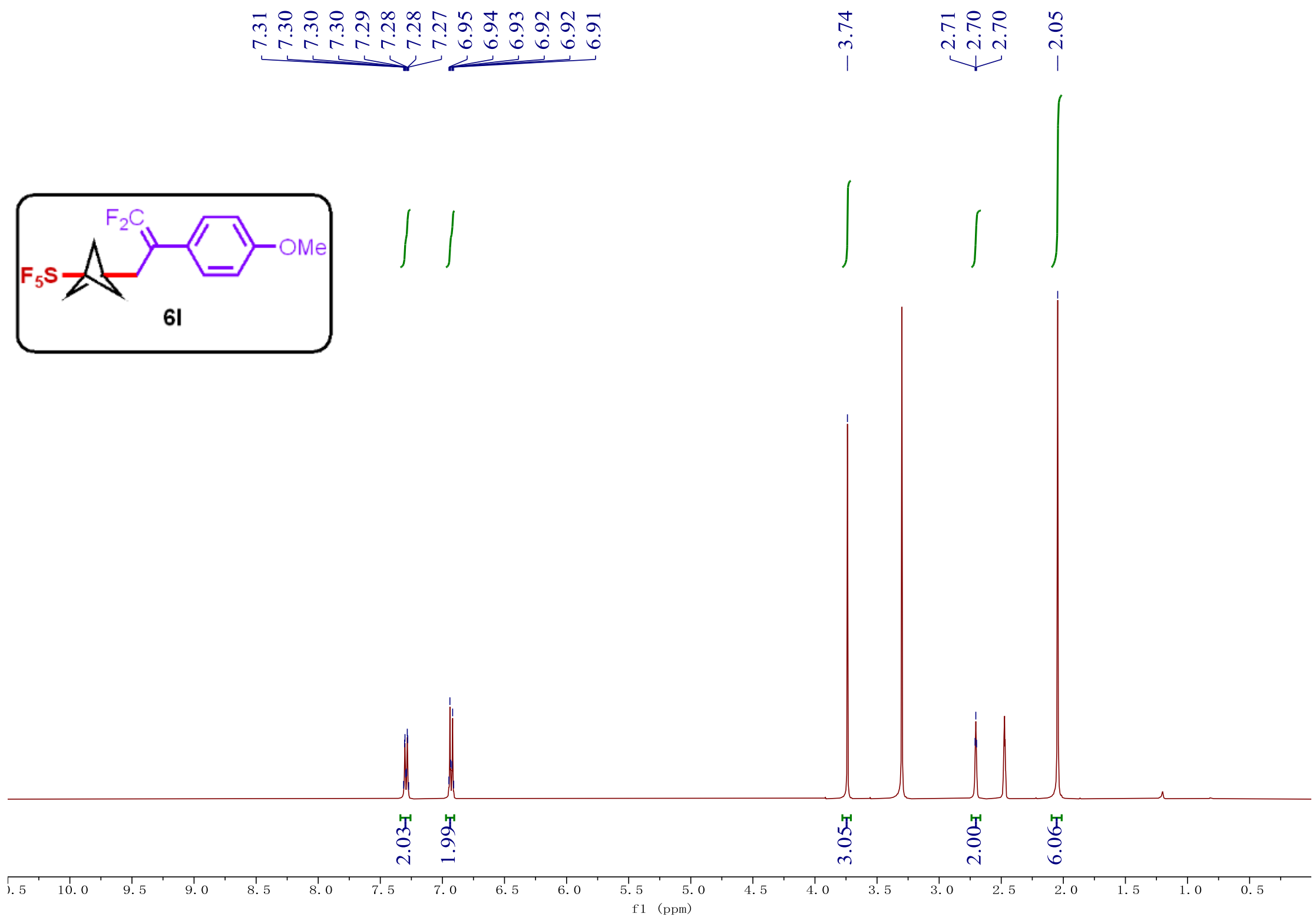

Supplementary Figure 208. ^{1}H **NMR Spectrum of Compound 6l (400 MHz, DMSO, 25 ˚C)**

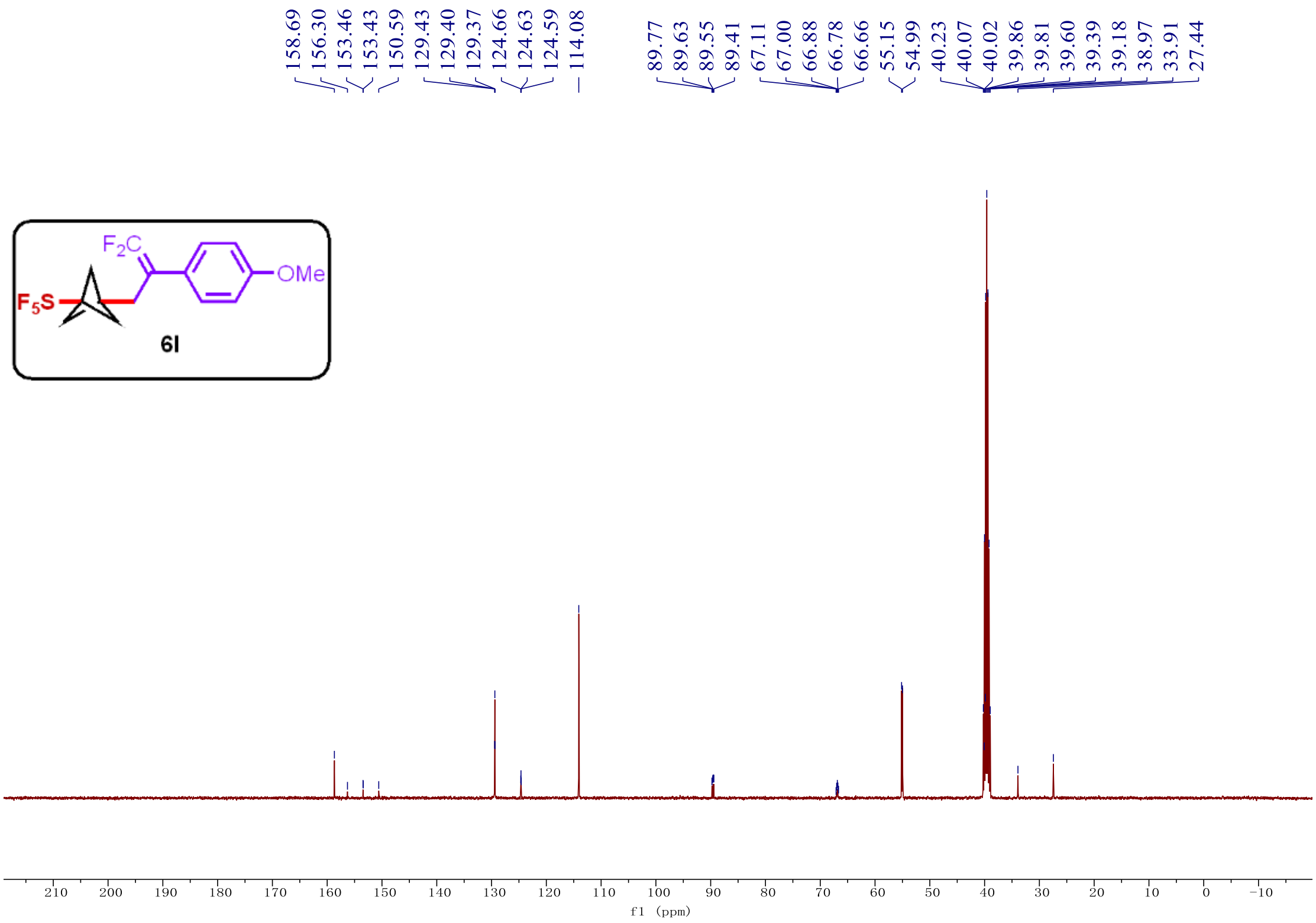

Supplementary Figure 209. ^{13}C NMR Spectrum of Compound 6l (101 MHz, DMSO, 25 ˚C)

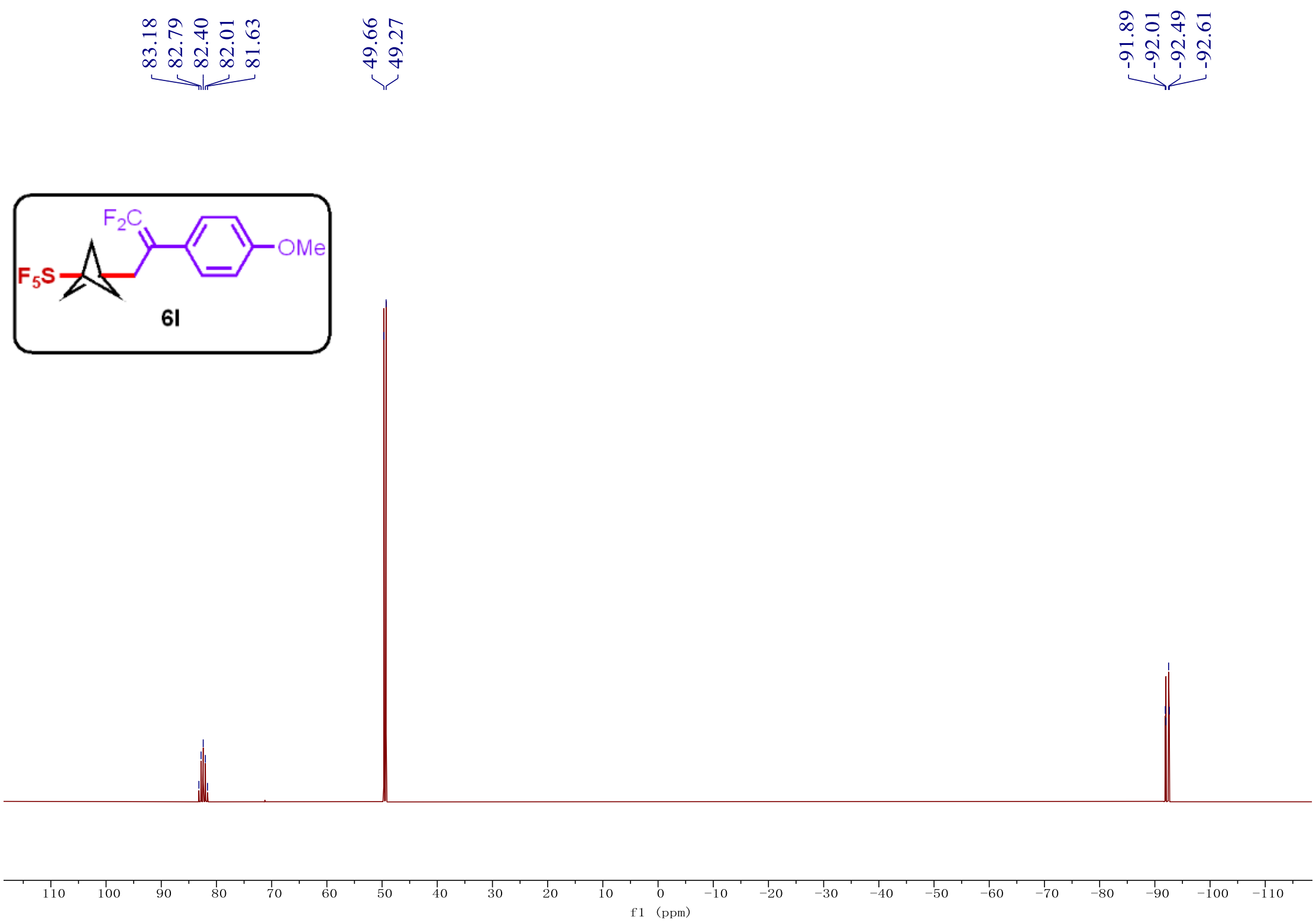

Supplementary Figure 210. ^{19}F NMR Spectrum of Compound 6l (376 MHz, DMSO, 25 ˚C)

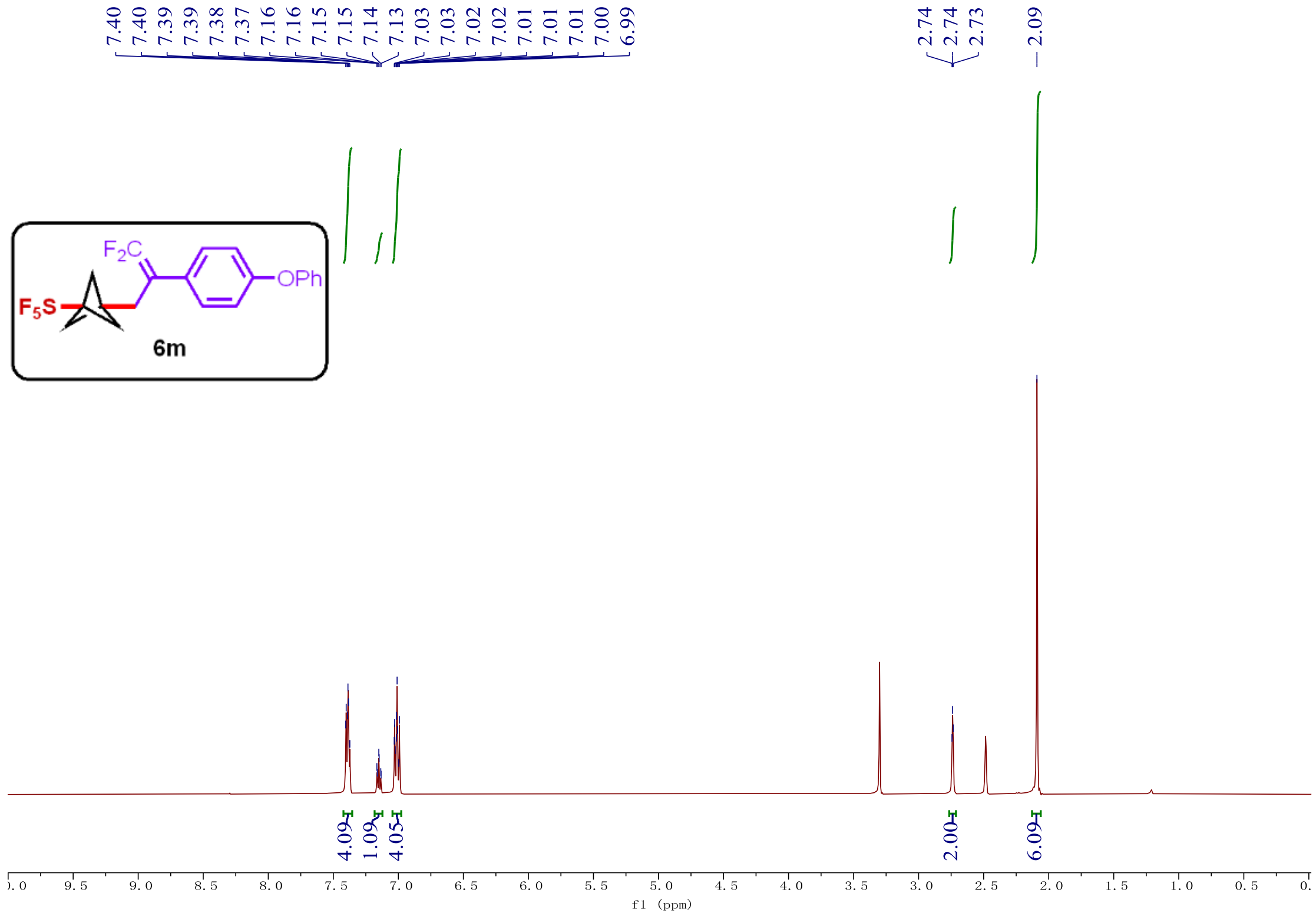

Supplementary Figure 211. ^{1}H NMR Spectrum of Compound 6m (500 MHz, DMSO, 25 °C)

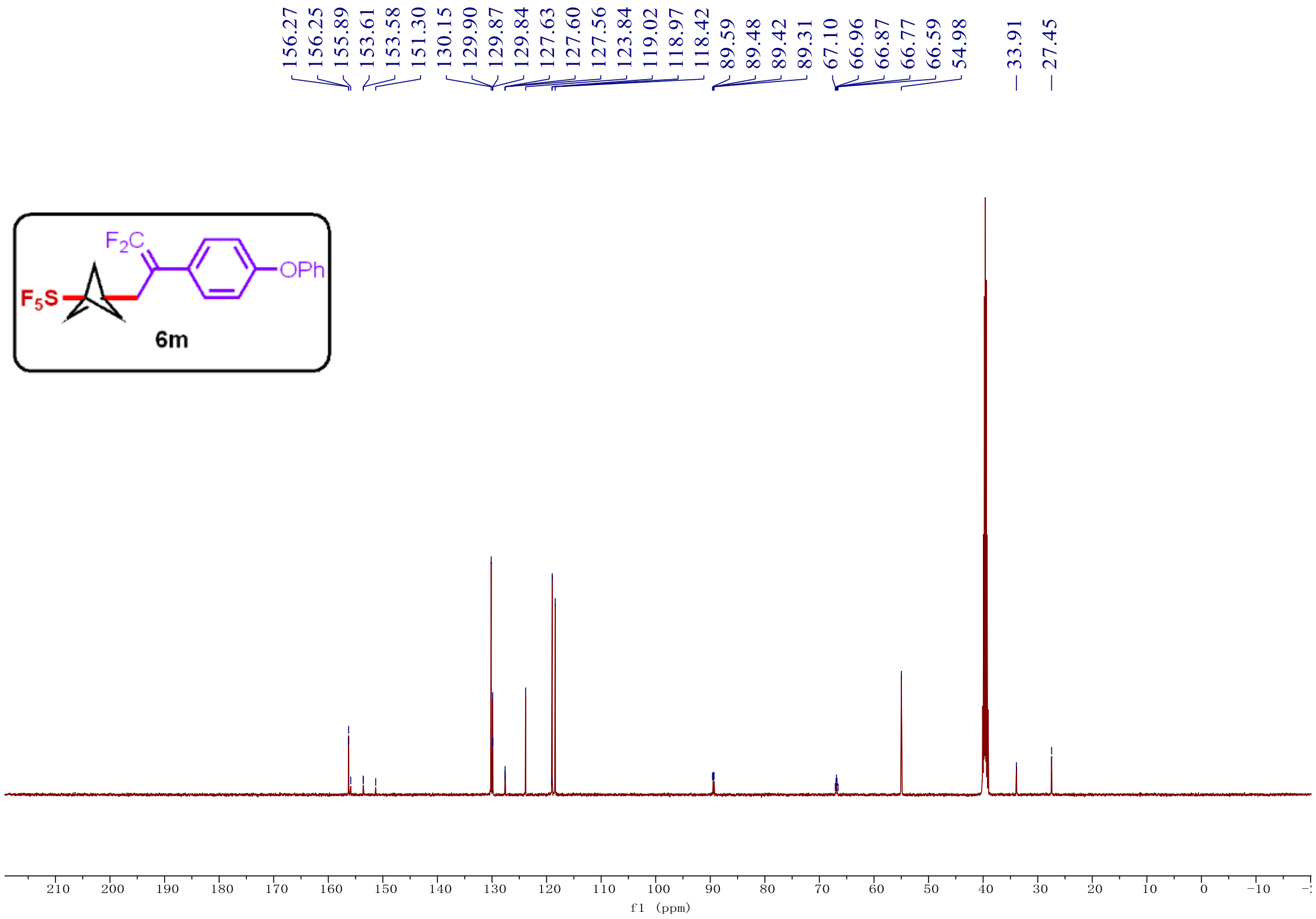

Supplementary Figure 212. ^{13}C NMR Spectrum of Compound 6m (126 MHz, DMSO, 25 °C)

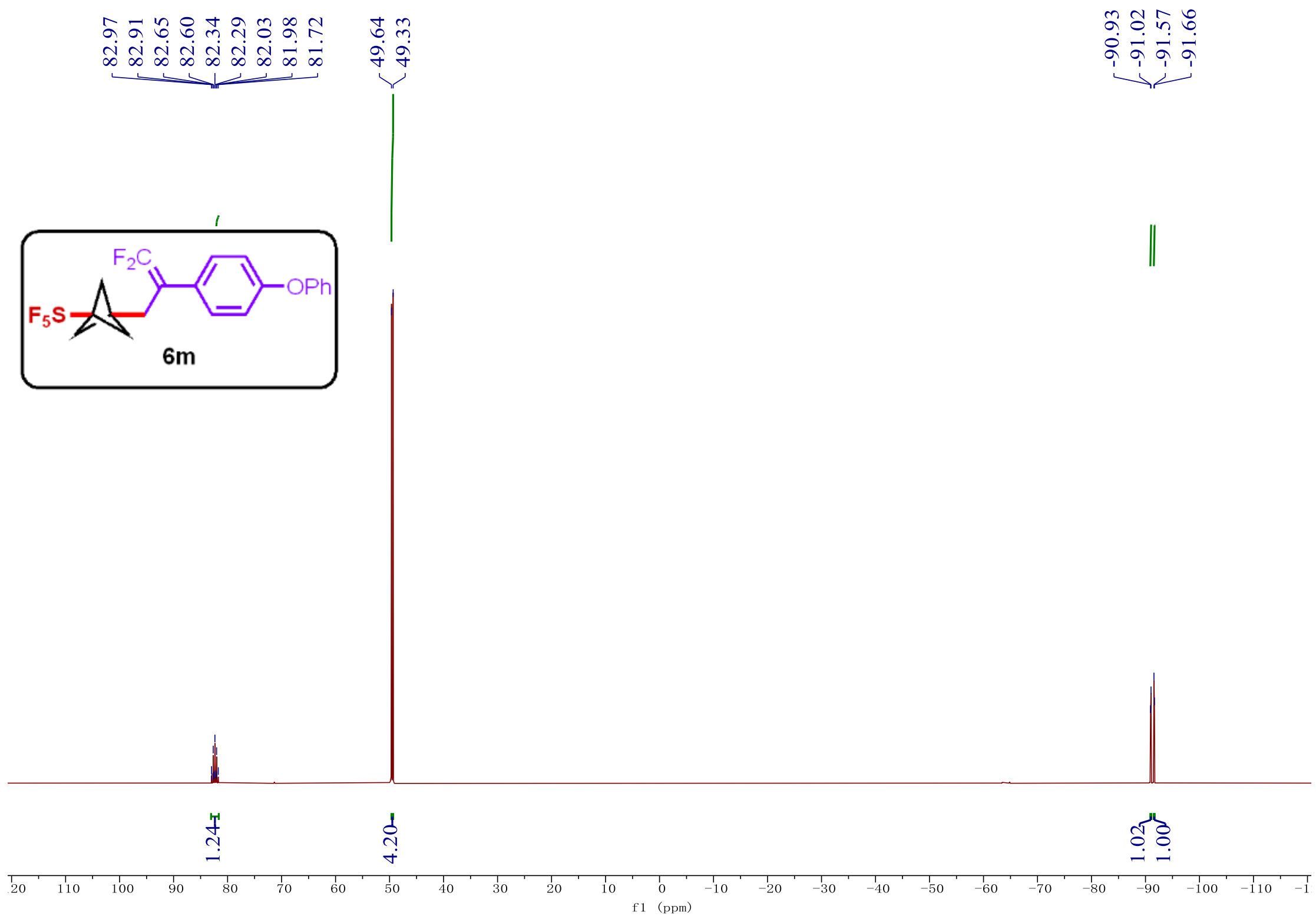

Supplementary Figure 213. ^{19}F NMR Spectrum of Compound 6m (471 MHz, DMSO, 25 °C)

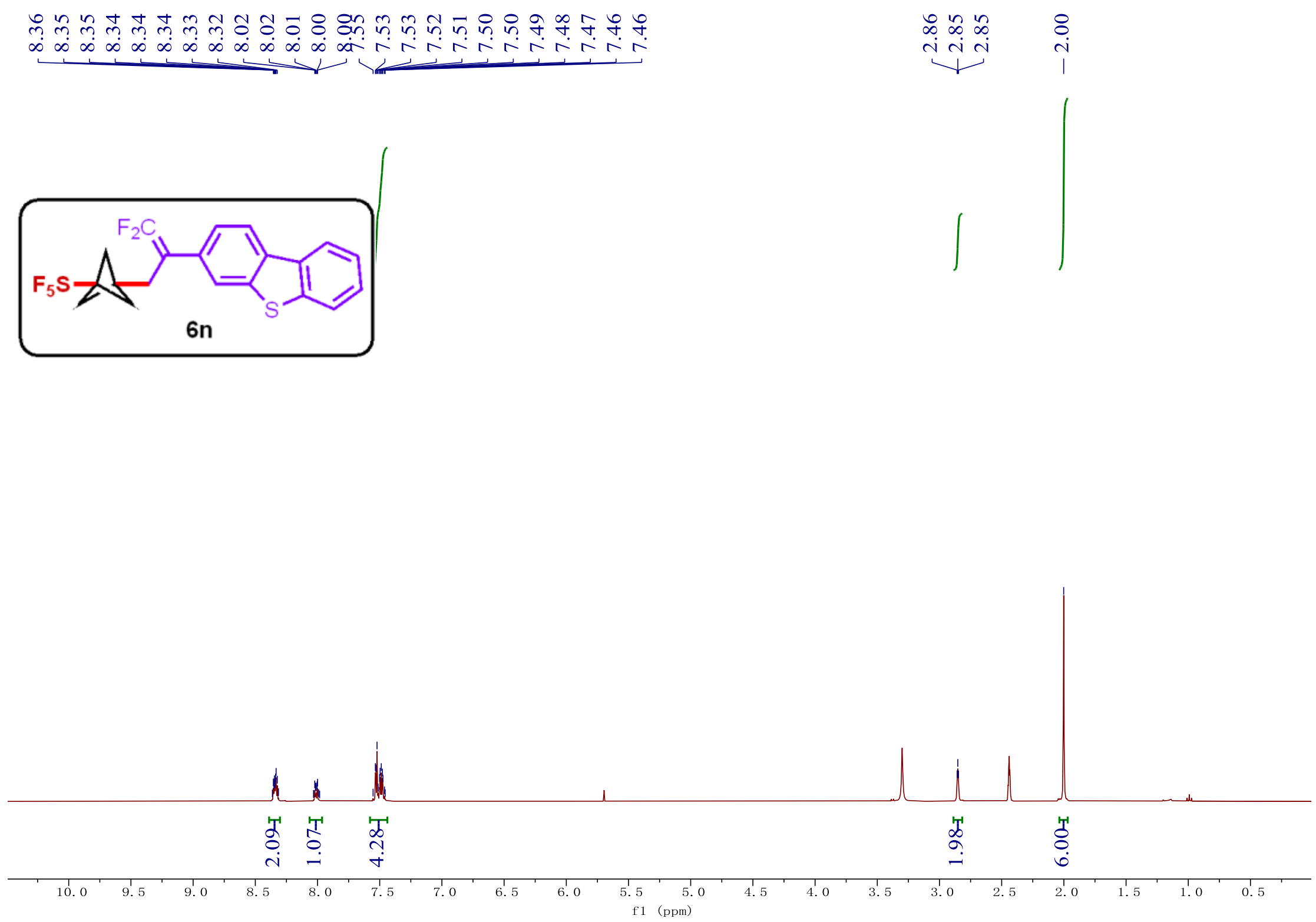

Supplementary Figure 214. ^{1}H NMR Spectrum of Compound 6n (400 MHz, DMSO, 25 °C)

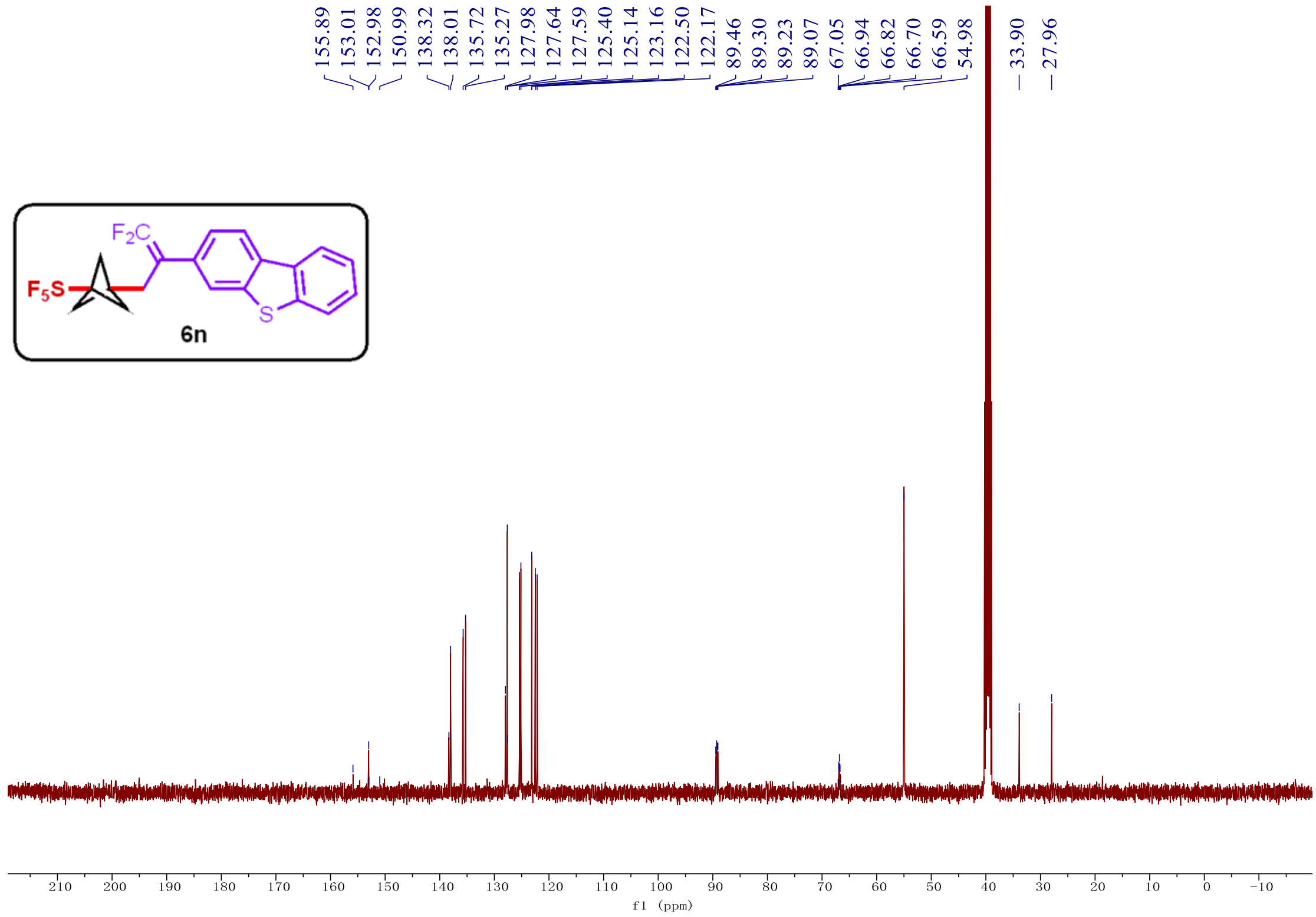

Supplementary Figure 215. ^{13}C NMR Spectrum of Compound 6n (101 MHz, DMSO, 25 °C)

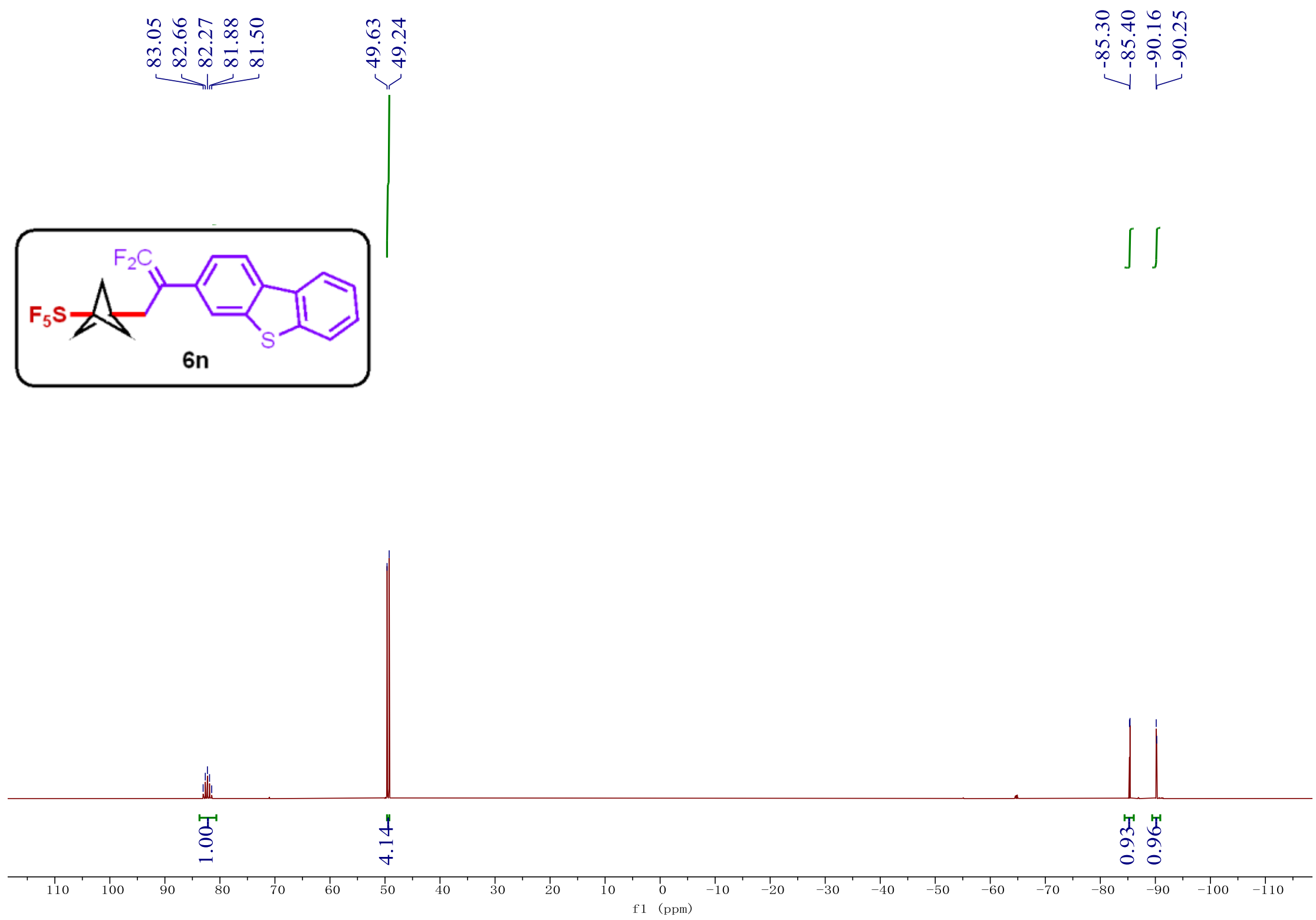

Supplementary Figure 216. ^{19}F NMR Spectrum of Compound 6n (376 MHz, DMSO, 25 °C)

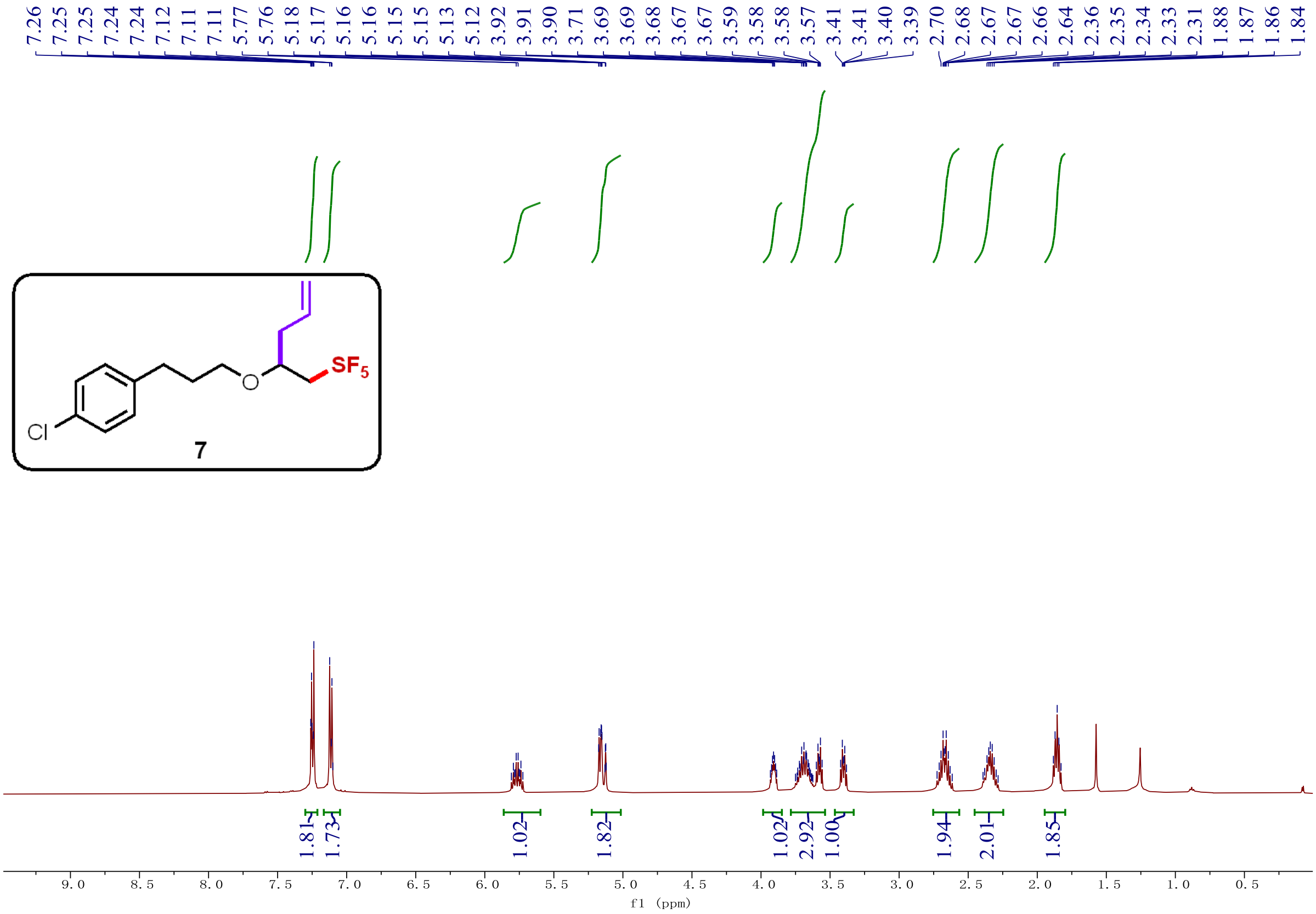

Supplementary Figure 217. ^{1}H NMR Spectrum of Compound 7 (500 MHz, $CDCl_3$, 25 °C)

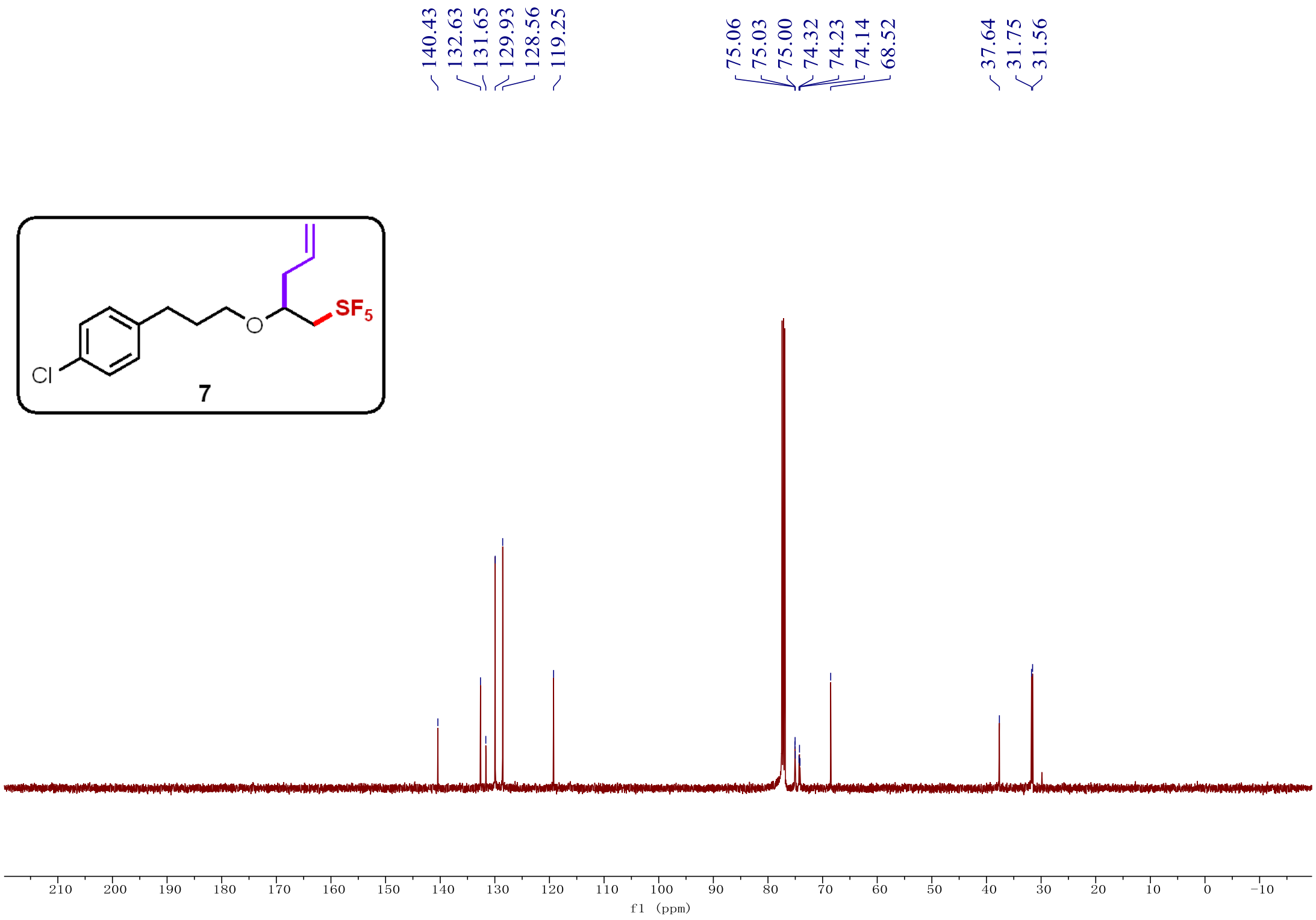

Supplementary Figure 218. ^{13}C NMR Spectrum of Compound 7 (126 MHz, $CDCl_3$, 25 °C)

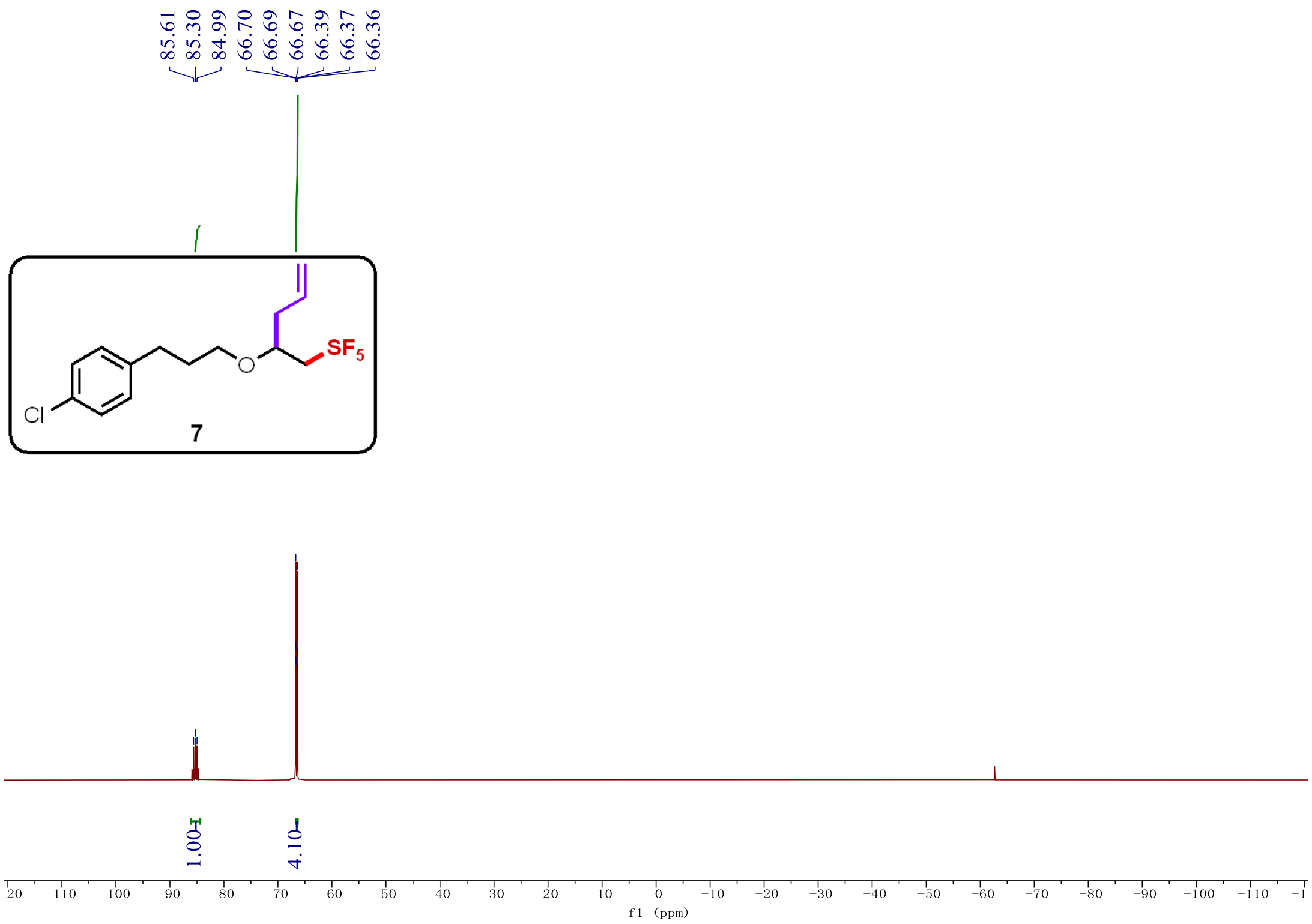

Supplementary Figure 219. ^{19}**F NMR Spectrum of Compound 7 (471 MHz, CDCl$_3$, 25 °C)**

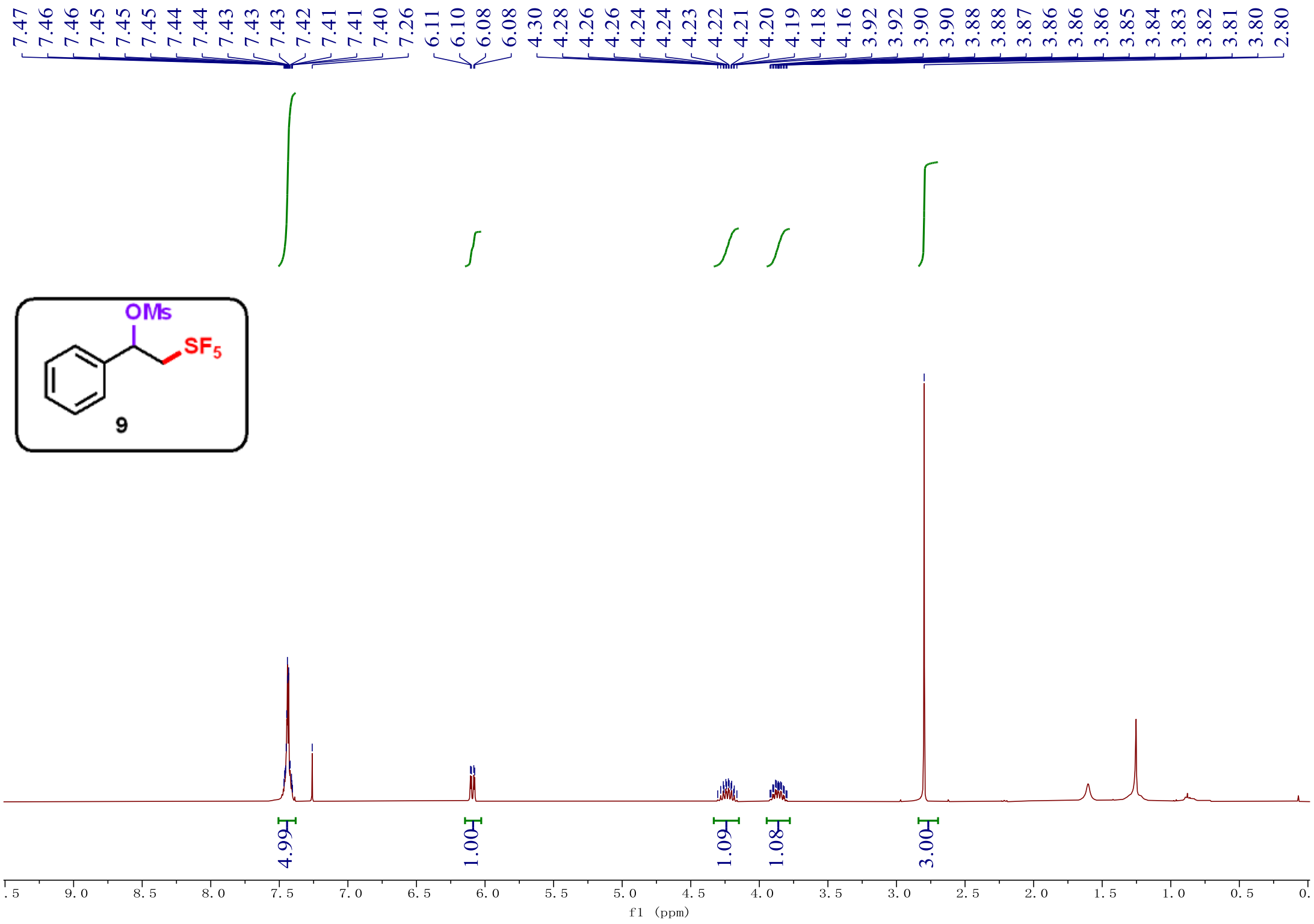

Supplementary Figure 220. ^{1}**H NMR Spectrum of Compound 9 (400 MHz, CDCl$_3$, 25 °C)**

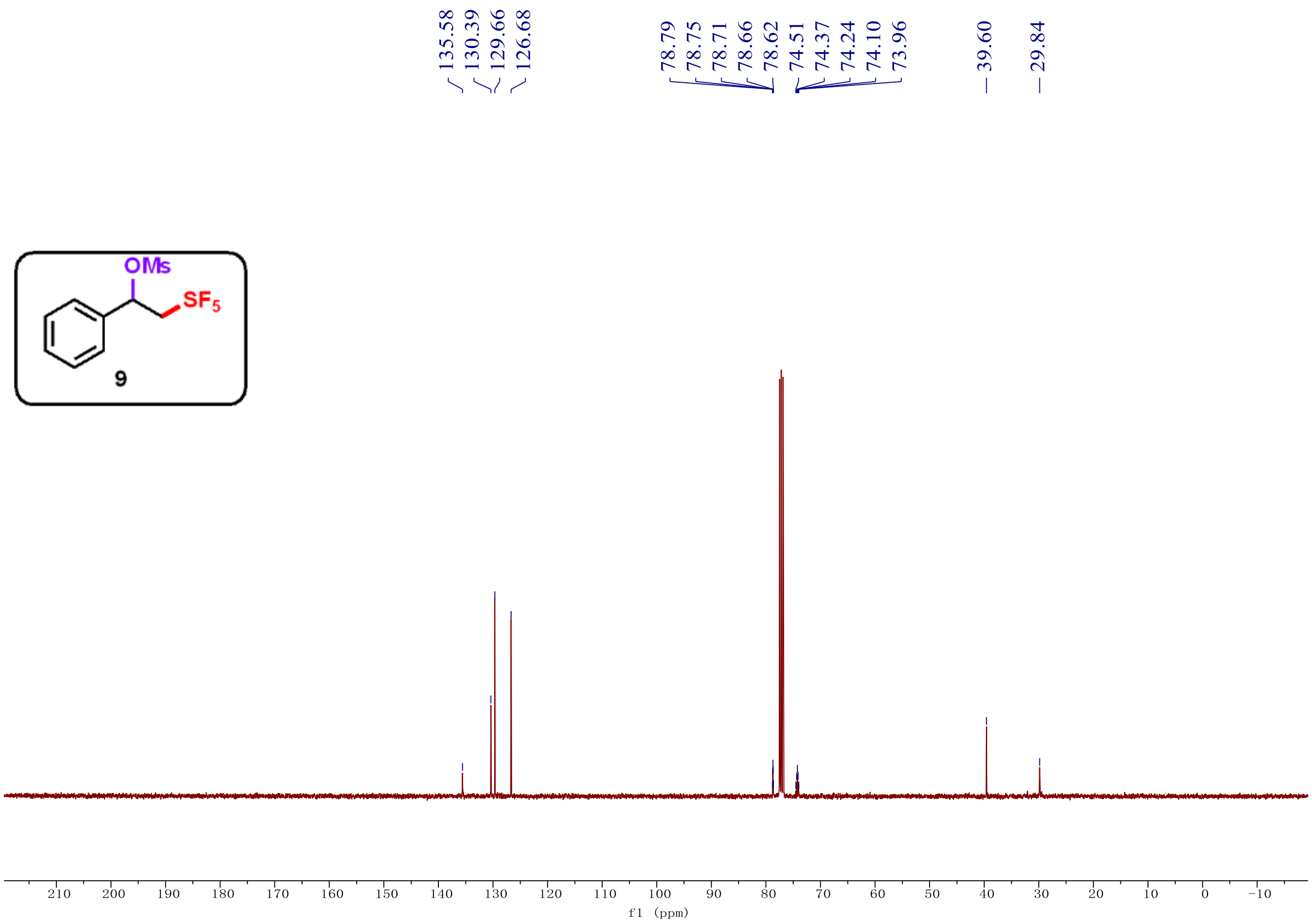

Supplementary Figure 221. ^{13}C **NMR Spectrum of Compound 9 (101 MHz,** $CDCl_3$**, 25 ˚C)**

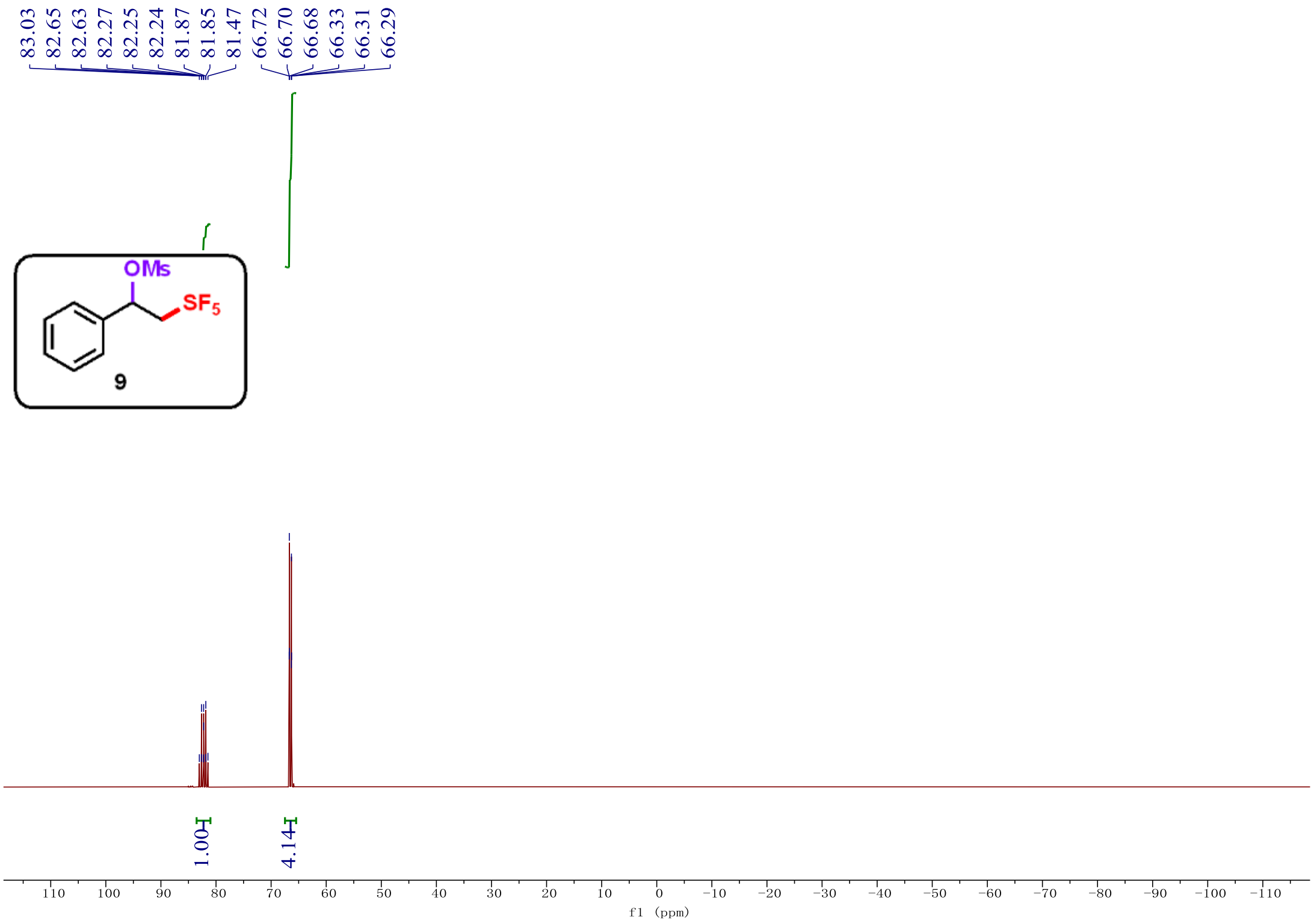

Supplementary Figure 222. ^{19}F **NMR Spectrum of Compound 9 (376 MHz,** $CDCl_3$**, 25 ˚C)**
